# Identifying molecular subgroups of patients with preeclampsia through bioinformatics

**DOI:** 10.3389/fcvm.2024.1367578

**Published:** 2024-06-03

**Authors:** Huijie Zhang, Jianglei Ma, Xueli Gao

**Affiliations:** ^1^ Department of Obstetrics, The Affiliated Yantai Yuhuangding Hospital of Qingdao University, Yantai, China; ^2^ Department of Infectious Diseases, Yantai Qishan Hospital, Yantai, China

**Keywords:** bioinformatics, gene, pregnancy, preeclampsia, subgroup

## Abstract

Preeclampsia (PE) is a pregnancy-related disorder associated with serious complications. Its molecular mechanisms remain undefined; hence, we aimed to identify molecular subgroups of patients with PE using bioinformatics to aid treatment strategies. R software was used to analyze gene expression data of 130 patients with PE and 138 healthy individuals from the Gene Expression Omnibus database. Patients with PE were divided into two molecular subgroups using the unsupervised clustering learning method. Clinical feature analysis of subgroups using weighted gene co-expression network analysis showed that the patients in subgroup I were primarily characterized by early onset of PE, severe symptoms at disease onset, and induced labor as the main delivery method. Patients in subgroup II primarily exhibited late PE onset, relatively mild symptoms, and natural delivery as the main delivery method. Gene Ontology and Kyoto Encyclopedia of Genes and Genomes pathway enrichment analyses revealed that the significant enrichment of calcium ion channels in subgroup II indicated the potential efficacy of calcium antagonists and magnesium sulfate therapy. In conclusion, the establishment of PE molecular subgroups can aid in diagnosing and treating PE.

## 1 Introduction

Preeclampsia (PE) is a pregnancy-related disorder characterized by elevated blood pressure that could be accompanied by proteinuria after 20 weeks of pregnancy (1). PE can cause serious complications, such as cerebral hemorrhage, pulmonary edema, acute kidney injury, liver dysfunction, hemolysis, seizures, and placental abruption (2). Furthermore, PE can result in premature delivery, fetal growth restriction, and an increased risk of fetal morbidity and mortality (1). Globally, PE occurs in 3%–5% of all pregnant women and is a crucial factor in maternal mortality (3). In the United States, 6.6% of pregnancy-related deaths are associated with gestational hypertension (1). Globally, approximately 760,000 maternal and 500,000 infant deaths are associated with PE annually, and the mortality rate of patients is approximately 10 times higher in low-income countries than in high-income countries (4). However, the pathogenesis of PE remains unclear.

Owing to the interaction between genetic, environmental, and immune factors, trophoblast invasion and spiral arteriole remodeling in the early stages of placental development can lead to uterine placental blood flow disorders (5). Under placental hypoxia, an imbalance in the expression of soluble factors can cause oxidative stress and inflammatory reactions, leading to systemic endothelial dysfunction and multisystem organ ischemia (1). As the delivery of the fetus is currently the only definitive treatment for PE, it poses a serious threat of increasing the risk of mortality for both pregnant women and newborns. The development of precision medicine has increased interest in identifying biomarkers for the diagnosis and treatment of PE. Over the past few decades, the importance of factors related to oxidative stress, inflammation, angiogenesis, and antiangiogenesis has improved the current understanding of the molecular pathogenesis of PE. Placental growth factor (PlGF) and soluble fibroid tyrosine kinase 1 (sFlt-1) are pro- and anti-angiogenic factors, respectively. In patients with PE, PlGF expression decreases and sFlt-1 expression increases (5). When a patient is <37 weeks pregnant and the sFlt-1:PlGF ratio is ≤38, the possibility of PE occurring within the next 4 weeks can be ruled out, with a predictive value of 99.3%. When the ratio is >38, the predictive accuracy of PE within 4 weeks is 36.7%, with a prediction sensitivity of 66.2% (6). In a recent study, genetic characteristics analysis using whole-exome sequencing in patients with severe PE identified subsets of protein interaction networks in these patients, suggesting that *LAMB2*, *PTK2*, *RAC1*, *QSOX1*, *FN1*, and *VCAM1* may be related to the pathogenesis of PE (7). Although introducing these biomarkers into clinical practice has yielded promising results, there are still significant unmet clinical needs; this may be because PE, including multiple subtypes, can lead to maternal and infant mortality and morbidity through various pathophysiological pathways. Approaches for correctly identifying and testing the subtype correlations are lacking (8).

Bioinformatics analysis is an important tool for analyzing and evaluating datasets in modern biomedical research. It can be used to predict protein structure, sequence alignment, protein–protein interaction (PPI), and other characteristics, and it is particularly useful for revealing the biochemical and functional roles of proteins (9). This study aimed to use unsupervised computational learning, weighted gene co-expression network analysis (WGCNA), Gene Ontology (GO) and Kyoto Encyclopedia of Genes and Genomes (KEGG) analyses to explore the entry points of the transcriptome dataset of patients with PE in the Gene Expression Omnibus (GEO) database, as well as to conduct bioinformatics analysis on these datasets to construct a molecular subgroup of these patients, providing new insights into the molecular mechanisms of PE. Effective identification of PE subtypes will contribute to the early prevention and diagnosis of PE and guide targeted treatment.

## 2 Methods

### 2.1 Downloading and processing of datasets

**Figure 1:**
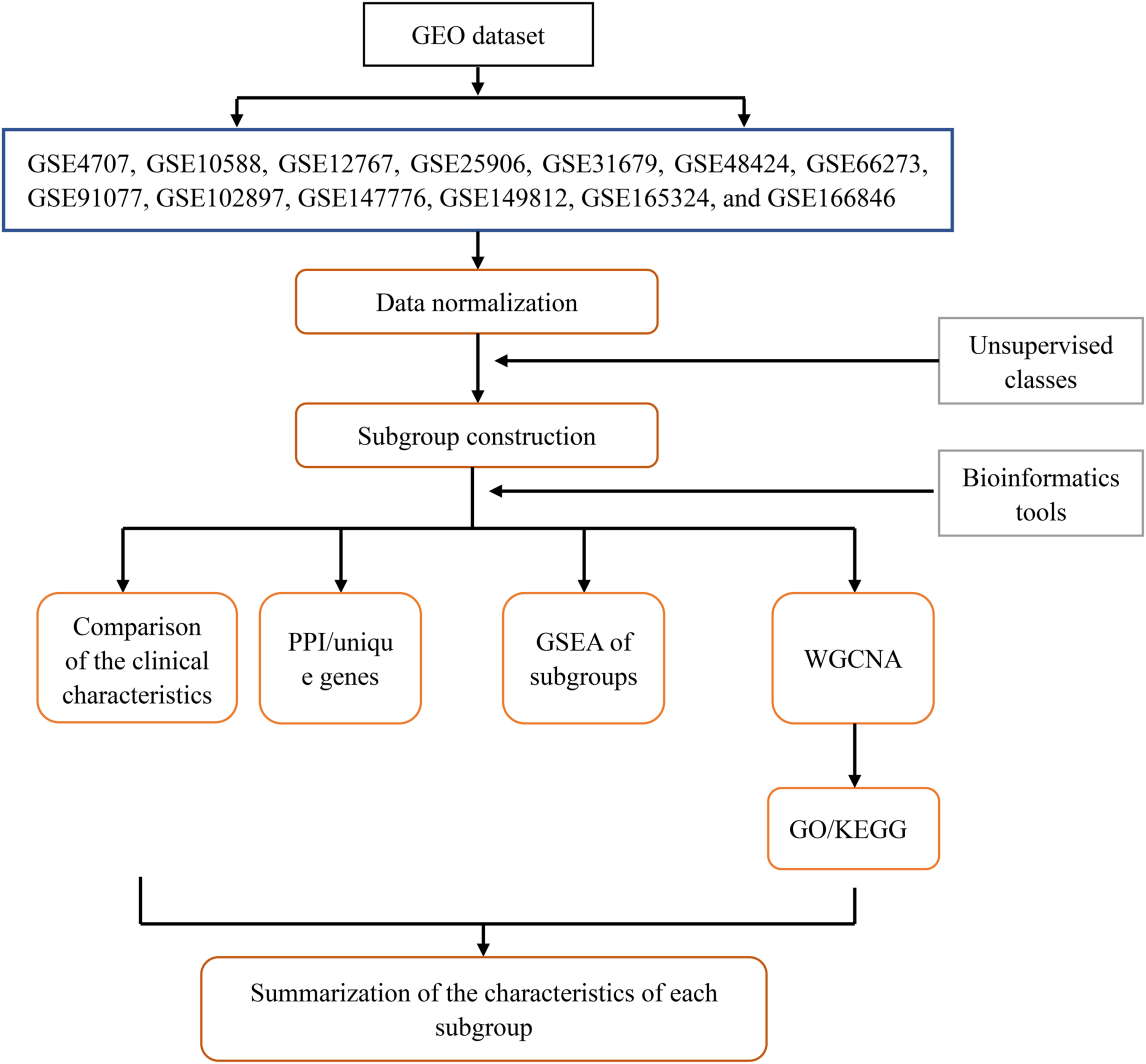
Flowchart of the research design.

We searched for “preeclampsia” in the GEO (https://www.ncbi.nlm.nih.gov/geo/) of the National Center for Biotechnology Information. We selected microarray datasets containing patients with PE and healthy controls (GSE4707, GSE10588, GSE12767, GSE25906, GSE31679, GSE48424, GSE66273, GSE91077, GSE102897, GSE147776, GSE149812, GSE165324, and GSE166846) and corresponding platform files (GPL1708, GPL2986, GPL570, GPL1602, GPL6947, GPL6480, GPL4133, GPL13497, GPL22120, GPL20844, GPL212844, GPL22120, and GPL22120) to download. Gene names on the dataset were annotated using Perl version 5.30.0 (https://www.perl.org/), and the “limma” and “sva” packages of R software (V 4.2.2) were used for principal component analysis (PCA) clustering batch correction to eliminate batch effects in samples (10). Specifically, the linear modeling method using the “limma” package was used to perform intragroup correction on each microarray dataset. Then, the “sva” package was used to integrate all samples in the datasets. As the data used in this study were sourced from the GEO public database, the contributors to the database obtained ethical approval and informed consent from each patient. Therefore, our study did not require further ethical or any other approval. The flowchart in Figure 1 shows the design for this study.

### 2.2 Constructing molecular subgroups of patients

Gene expression was analyzed after batch correction using the “limma” package of R software, and consensus clustering was conducted on genes and visual output heatmaps using the “ConsensusClusterPlus” package (11). Specifically, an unsupervised clustering learning method was used to cluster genes with the same genetic characteristics as those expressed in patients with PE. The outputs were visualized by grouping the number of clusters (defined as a parameter κ and set to 2–10), and the internal clustering of each group was evaluated. The higher the evaluation, the better the similarity in gene expression within the group. The subgroup with the highest consistent evaluation score was selected as the study subtype.

### 2.3 Identification of clinical features of patient subgroups

Based on the continuity of clinical features, the clinical characteristics of patients with PE were divided into discrete variables, including symptom severity (severe PE was defined as one or more of the following conditions: blood pressure above 160/110 mmHg, proteinuria above 5,000 mg/24 h, comorbidities with multiple system diseases, maternal seizures, stroke or fetal intrauterine growth restriction below the third percentile), delivery method, and sex of newborns; and continuous variables, including patient age and gestational week (early onset: gestational age <34 weeks; late onset: gestational age ≥34 weeks). The proportion of discrete variables in patients was analyzed using the “rstatix”, “ggplot2”, “ggpubr”, and “reshappe2” packages of the R software (12), and the mean and standard deviation values of continuous variables were examined using the “ggplot2” and “ggpubr” packages. Differences between groups were compared using a one-way analysis of variance.

### 2.4 Screening of subgroup-unique genes and construction of PPI networks

We used the “limma” package to screen differentially expressed genes (DEGs) between subgroups and control groups and within each subgroup. The intersecting genes of the two genes were the uniquely expressed genes of the subgroup. Unique genes in the subgroups were significantly differentially expressed only in specific subpopulations. The screening criteria were a mean filter of >0.2 and a corrected *p*-value of <0.05. To better analyze the characteristics of DEGs between subpopulations, we only selected the uniquely upregulated DEGs of each subpopulation. The top 10 unique DEGs from each subgroup were selected and imported into the STRING online tool (https://string-db.org/). A confidence level of 0.4 was selected, and a PPI network diagram was created to understand the relationship between protein linkages within each subgroup.

### 2.5 Gene set enrichment analysis (GSEA) of patient subgroups

To determine the unique DEGs among different subgroups and to evaluate whether the consistency of DEGs between subgroups and normal samples, the comparison files of the subgroups and control groups were converted into gene sets and gene list files using the Perl software. The files converted from each subgroup were inputted into GSEA version 5.32 (13), the running parameters were set to 5,000, and the remaining parameters were obtained from the software by default.

### 2.6 WGCNA of patient subgroups

We used the “WGCNA” package of R software to construct and visualize the WGCNA for subgroup-unique genes (14). First, we used the “limma” package to filter the corrected abnormal samples. A co-expression network was then constructed using the standard scale-free network analysis function, and the soft threshold power value β was calculated using the power function “pickSoftThreshold” to enhance the aggregation of gene expression (15). We drew a tree-like gene module diagram for co-expressed gene clustering. Pearson's correlation analysis was used to analyze the relationship among the delivery mode, symptom severity, sex of newborns, maternal age, and gestational week with the genetic modules to determine the relationship between clinical traits and genetic modules. The expression of gene modules obtained from the WGCNA in subgroups I and II was produced using the “heatmap” package of R software and was presented in the form of a heatmap (14). Finally, a visualized expression heatmap of gene modules in subgroups, which could indirectly reveal the enrichment of subgroups and clinical traits, was drawn using the “limma” and “pheatmap” packages of the R software.

### 2.7 GO and KEGG pathway analyses of gene modules

We analyzed the GO [biological process (BP), molecular function (MF), and cellular component (CC)] of the gene module using the “org.Hs.eg.db”, “clusterProfiler”, and “enrichplot” packages of R software (16). The KEGG pathway enrichment analysis was performed using the DAVID v6.8 online tool (https://david.ncifcrf.gov/) (17). The significantly expressed genes from each gene module were entered into the DAVID website to obtain significantly enriched KEGG pathways.

### 2.8 Statistical analyses

This study used R software version 4.2.2. Intergroup comparisons were conducted using a one-way analysis of variance. Continuous variables were analyzed using the SPSS 26.0 statistical software (IBM Corp., Armonk, NY, USA), and discrete variables were analyzed using the chi-squared test. The results are expressed as mean ± standard deviation. A *p*-value of <0.05 was considered statistically significant.

## 3 Results

### 3.1 Data processing and grouping

**Figure 2:**
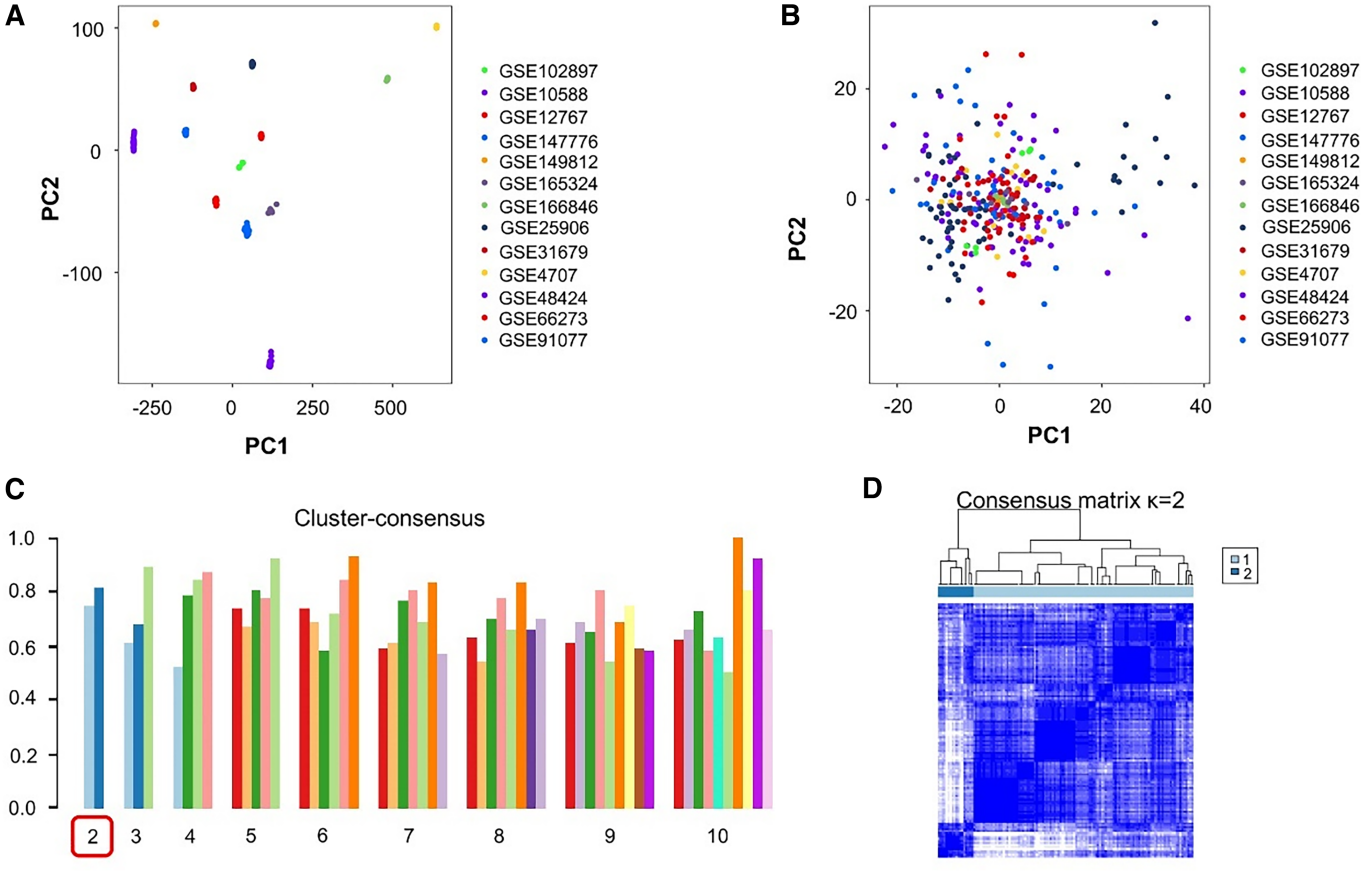
Processing and grouping of transcriptome datasets. (**A**) Pre-processing principal component analysis (PCA) visualization results, with different colors representing different datasets. (**B**) PCA visualization results after batch effect processing, in which the samples are centered and aggregated into a large set. (**C**) The consistency evaluation visualization results of clustering show that when clustering is set to 2, the subgroup scores are all higher. (**D**) When clustering is set to 2, the heatmap matrix shows that the darker the blue color inside the subgroup, the higher the similarity of gene expression.

After organizing all datasets, samples from 130 patients with PE and 138 healthy individuals were obtained. PCA-based visualization images showed that before the batch effects were eliminated, each dataset was located in a different region; there was no intersection between datasets (Figure 2A). After eliminating the batch effects, the PCA results showed that all samples were clustered into a single region, which could be regarded as a unified dataset (Figure 2B). After consensus clustering of genes in patients with PE, based on the set κ, nine clusters were obtained. The visualized clustering results showed that when the cluster was two, the consistency evaluation of each subgroup was at a high level (Figure 2C). The cluster correlation heatmap of subgroups showed high enrichment within each subgroup and low enrichment between subgroups (Figure 2D).

### 3.2 Clinical characteristics of patient subgroups

**Figure 3:**
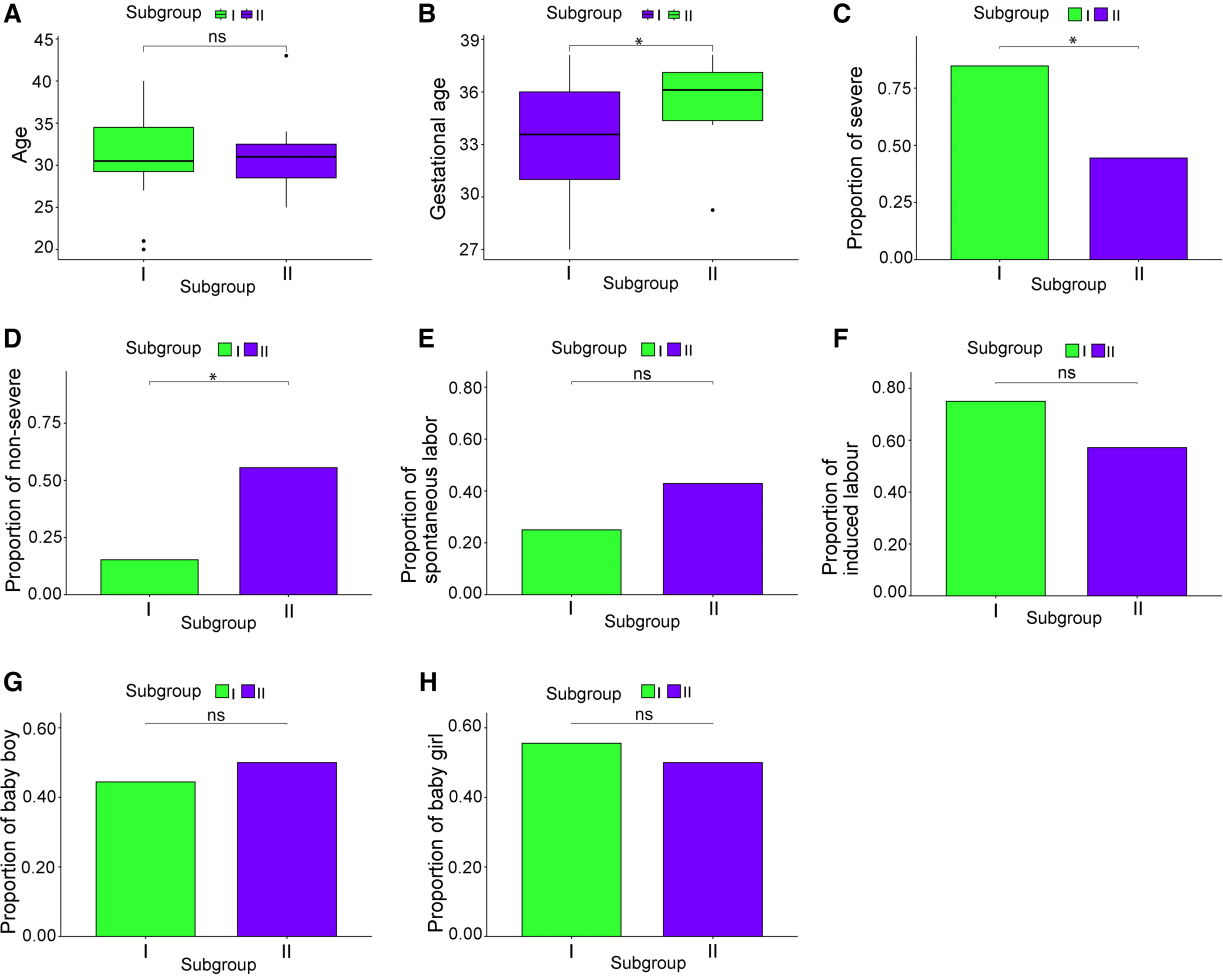
Clinical characteristics of the subgroups. (**A**) Patient age; (**B**) gestational week; (**C**) proportion of severe cases; (**D**) proportion of mild cases; (**E**) proportion of natural childbirth; (**F**) proportion of induced labor; (**G**) male infant ratio; (**H**) female infant ratio. *p < 0.05.

The clinical characteristics of 130 patients with PE (112 in subgroup I and 18 in subgroup II) were analyzed using the downloaded datasets (Supplementary Table S1). The mean and standard deviation values of age (years) and gestational week were 31.14 ± 5.13 and 33.30 ± 3.44, respectively, in subgroup I and 31.57 ± 5.77 and 35.44 ± 2.28, respectively, in subgroup II. Intergroup analysis showed no significant difference in age between subgroups I and II (Figure 3A), and the gestational week of subgroup I was significantly lower than that of subgroup II (*p* < 0.05; Figure 3B). The symptoms in subgroup I were more severe than those in subgroup II (*p *< 0.05; Figures 3C,D). Although more patients in subgroup I underwent induced labor based on clinical decision and those in subgroup II experienced spontaneous labor, no significant difference was found in the delivery methods (Figures 3E-F). In addition, no significant difference was observed in the sex of newborns between these two subgroups (Figures 3G-H).

### 3.3 Unique genes and PPI networks of patient subgroups

**Figure 4:**
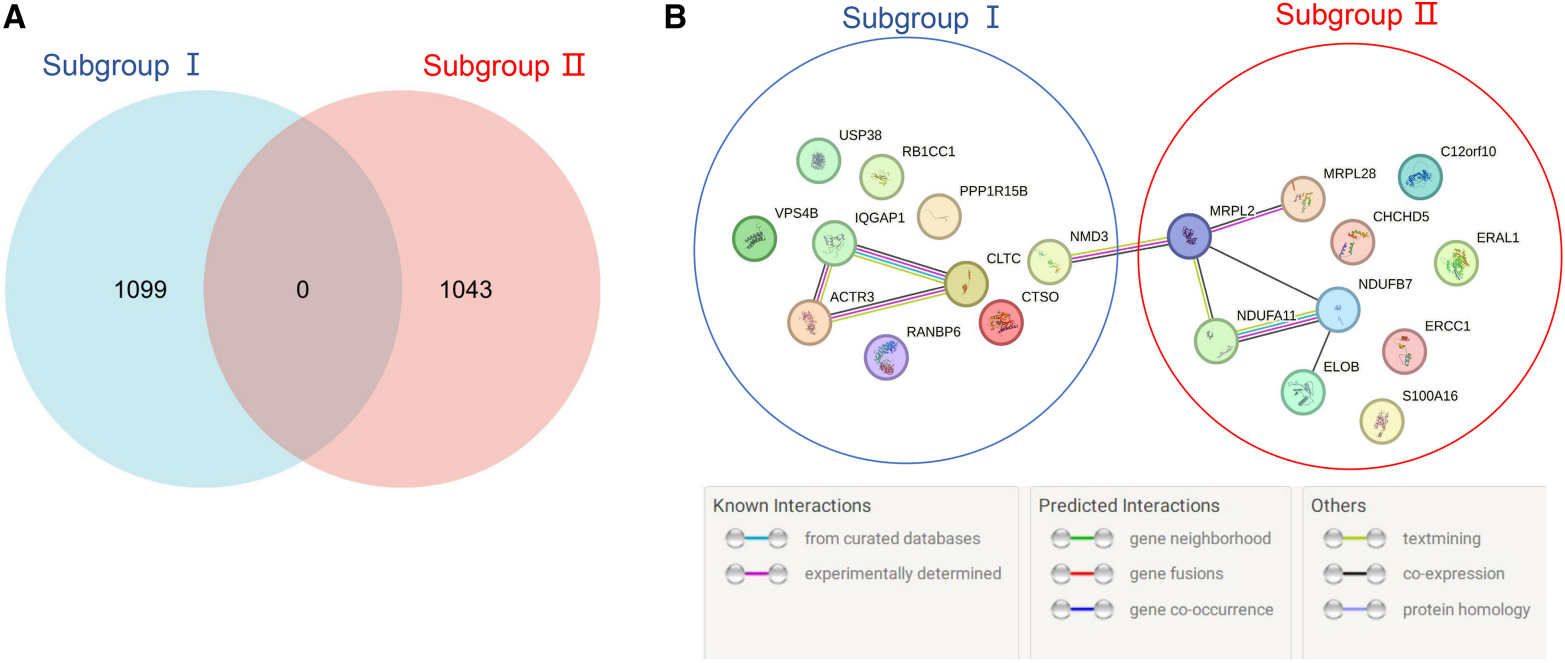
Venn diagram and PPI network of subgroup-unique differentially expressed genes (DEGs). (**A**) The blue and red areas represent all unique DEGs of subgroups I and II, and the middle region represents intersecting DEGs between subgroups. (**B**) The blue area represents the DEGs of subgroup I, the red area represents the DEGs of subgroup II, and different colored balls and lines represent the relationships between genes.

According to the screening criteria of a mean filter of >0.2 and corrected *p-*value of <0.05, 3,536 upregulated DEGs and 3,413 downregulated DEGs were identified between subgroup I and the control group (Supplementary Table S2); 3,162 upregulated DEGs and 3,787 downregulated DEGs were identified between subgroup II and the control group (Supplementary Table S3); and 3,168 upregulated DEGs and 3,781 downregulated DEGs were identified between subgroups I and II (Supplementary Table S4). After screening for upregulated DEGs and intersections, 1,099 and 1,043 unique DEGs were identified in subgroups I and II, respectively (Supplementary Table S5). The intersection results indicated no common unique DEGs among the subgroups (Figure 4A). The PPI network results showed 20 nodes, 9 protein pairs, and close interactions of DEGs within each subgroup (Figure 4B). Table 1 lists the number of nodes in the top 10 DEGs for each subgroup.

**Table 1:** PPI network nodes of the top 10 DEGs in the subgroups.

Subgroup	Gene	Nodes
Ⅰ	*IQGAP1*	7
	*USP38*	0
	*ACTR3*	6
	*RB1CC1*	0
	*NMD3*	3
	*PPP1R15B*	0
	*VPS4B*	0
	*RANBP6*	0
	*CLTC*	7
	*CTSO*	0
Ⅱ	*C12orf10*	0
	*CHCHD5*	0
	*ERAL1*	0
	*MRPL28*	2
	*MRPL2*	5
	*ERCC1*	0
	*S100A16*	0
	*TCEB2*	1
	*NDUFB7*	6
	*NDUFA11*	6

### 3.4 GSEA of patient subgroups

**Figure 5:**
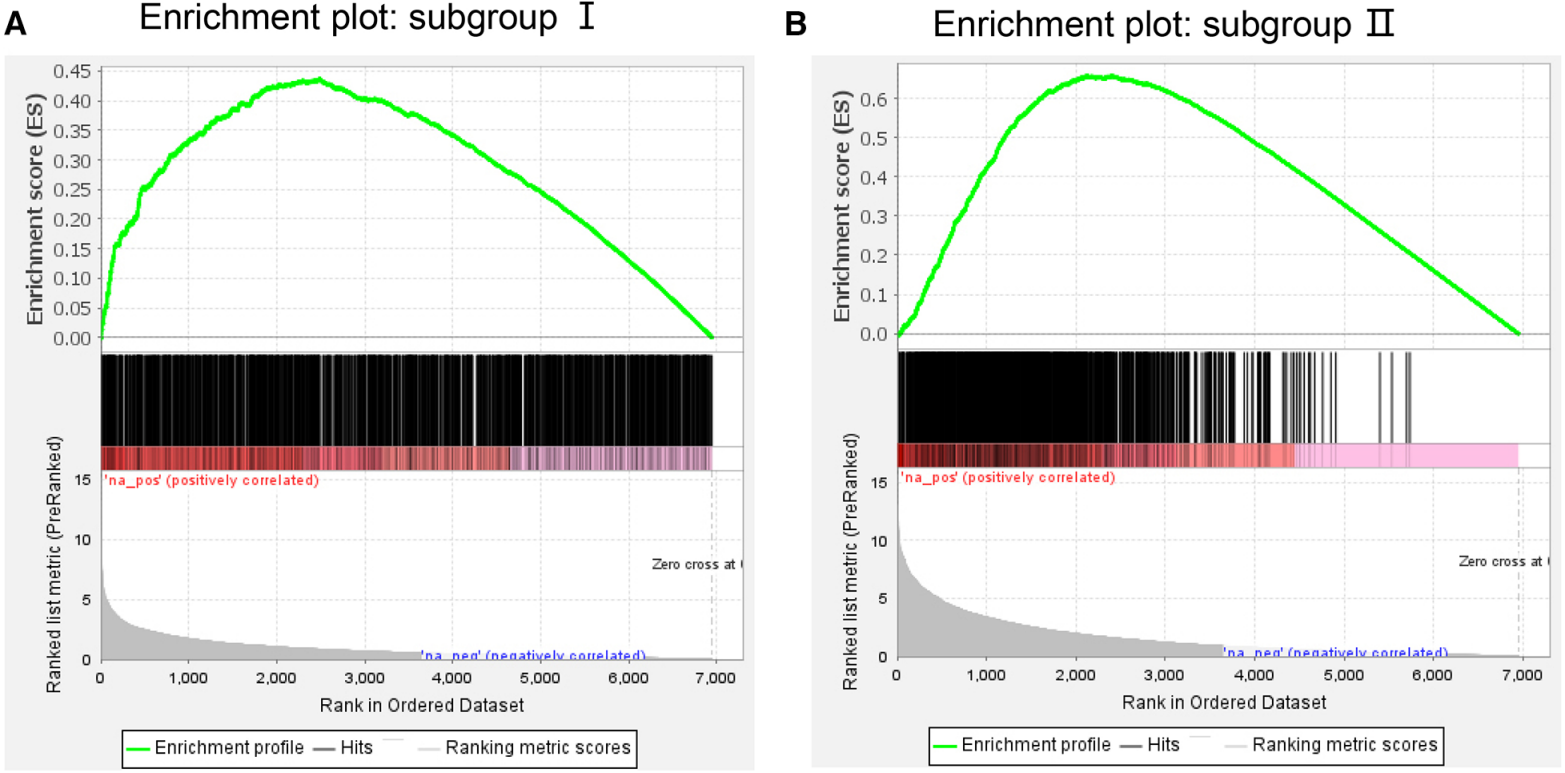
Gene Set enrichment analysis of (**A**) subgroup I and (**B**) subgroup II. The green line represents the score of the enrichment pathway. Each black line represents a differentially expressed gene, and the gray area represents the signal-to-noise ratio between the subgroup and control.

On the GSEA image, the black vertical lines represent the unique DEGs among subgroups, whereas the gray vertical lines represent the DEGs between subgroups and health control samples. Both sets of data were enriched on the left side of the image. Moreover, the *p*-value and error detection rate value were <0.05, indicating that the two sets of DEGs exhibited the same enrichment trend (Figure 5). GSEA results indicated that the unique DEGs among different subgroups, DEGs between subgroups, and health normal samples were consistent.

### 3.5 WGCNA in patients with PE

**Figure 6:**
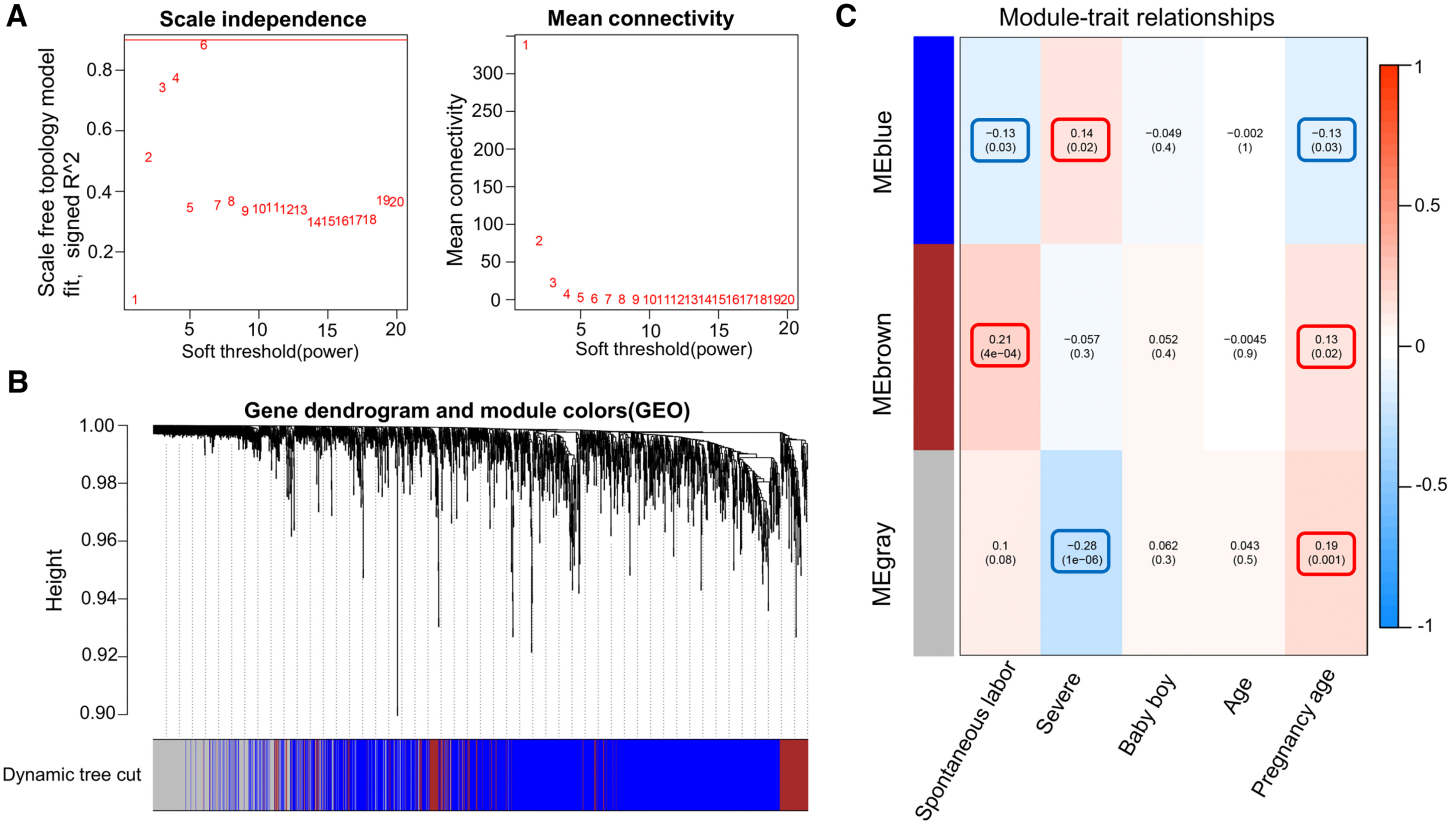
WGCNA of the dataset. (**A**) Different soft threshold power values in a standard scale-free network, with the red line indicating the optimal power value. The left figure shows the power value and scale-free fitting index. (**B**) Color gene module obtained by the dynamic tree graph method. (**C**) Heatmap of the correlation between color modules and clinical features is visualized using the “WGCNA” package of the R software (version 4.2.2).

**Figure 7:**
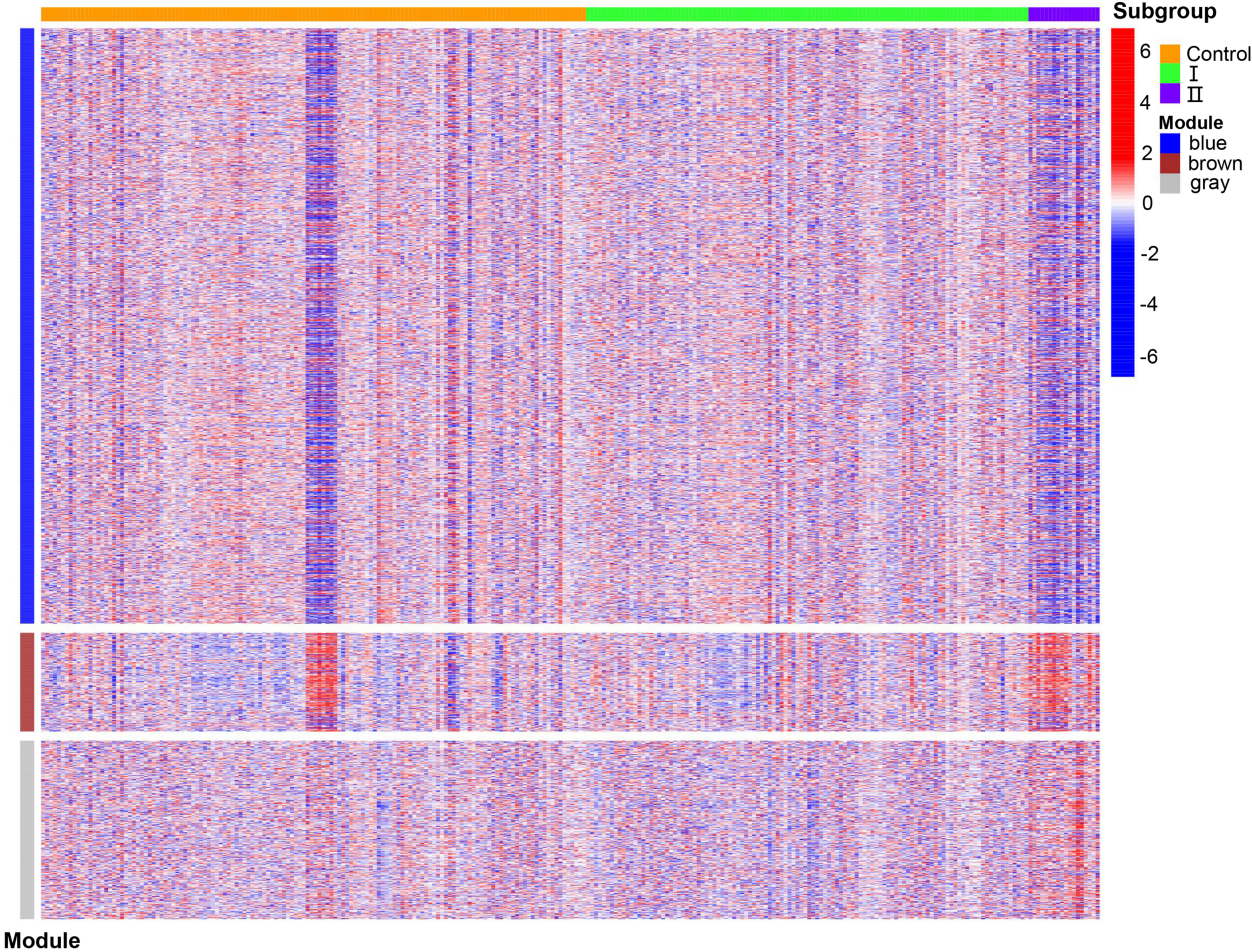
Enrichment heatmap of gene modules and subgroups representing different subgroups on the horizontal axis and different colored gene modules on the vertical axis. Red represents high expression, and blue represents low expression; the darker the color, the more significant it is.

WGCNA was performed on 6,949 genes in the processed samples. After excluding abnormal samples, the optimal soft threshold power value was selected as 6 (Figure 6A). According to the gene consensus expression tree, three colored gene modules were generated: blue (1,462 genes), brown (242 genes), and gray (438 genes; Figure 6B). The heatmap of gene modules and clinical traits showed a significant negative correlation (*p *= 0.03) between spontaneous labor and blue modules and a significant positive correlation (*p *< 0.001) with brown modules. Severe symptoms showed a significant positive correlation with the blue module (*p *= 0.02) and a significant negative correlation with the gray module (*p *< 0.001). Gestational week was significantly negatively correlated with the blue module (*p *= 0.03) and positively correlated with the brown (*p *= 0.02) and gray (*p *= 0.001) modules (Figure 6C). The enrichment heatmap of the gene modules and subgroups showed that the blue modules in subgroup I were highly expressed, whereas brown and gray modules were poorly expressed. Moreover, blue modules revealed low expression in subgroup II, whereas brown and gray modules showed high expression (Figure 7).

### 3.6 GO and KEGG

**Figure 8:**
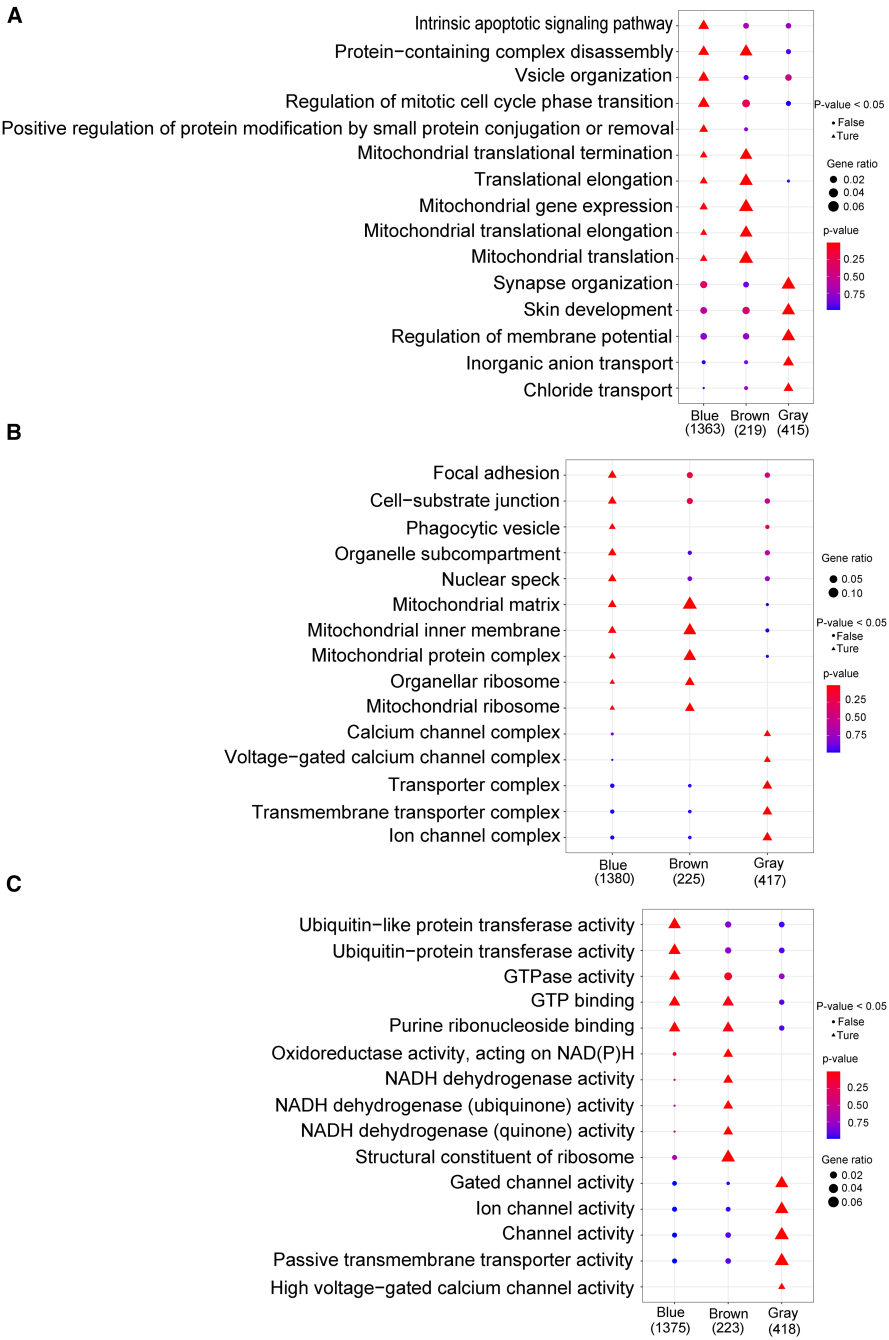
Go analysis. (**A**) Biological process; (**B**): cellular component; (**C**) molecular function. The horizontal axis represents different color gene modules, whereas the vertical axis represents terminology. The triangle expression is significantly enriched (*p *< 0.05), whereas the circle expression is not significant (*p *> 0.05); the larger the shape, the more significant the difference.

By analyzing the GO and KEGG enrichment pathways of gene modules, we observed that for BP, intrinsic apoptotic signaling pathways, vessel organization, regulation of mitotic cell cycle phase transition, and positive regulation of protein modification by small protein aggregation or removal were significantly enriched in the blue module (*p *< 0.05); synapse organization, skin development, regulation of membrane potential, inorganic anion transport, and chloride transport were significantly enriched in the gray module (*p *< 0.05; Figure 8A). For CC, focal adhesions, cell-substrate junctions, pharmacological vesicles, organelle subcompartments, and nuclear species were significantly enriched in the blue module (*p *< 0.05); the calcium channel, voltage-gated calcium channel, transporter, transmembrane transporter, and ion channel complexes were significantly enriched in the gray module (*p *< 0.05; Figure 8B). For MF, ubiquitin-like protein transfer, ubiquitin–protein transfer, and GTPase activities were significantly enriched in the blue module (*p *< 0.05); oxidoreductase activity with NAD(P)H as a donor and quinone or a similar compound as an acceptor, NADH dehydrogenase activity, and structural constitution of the ribosome were significantly enriched in the brown module (*p *< 0.05); and gated channel, ion channel, channel, passive transmembrane transporter, and high voltage-gated calcium channel activities were significantly enriched in the gray module (*p *< 0.05) (Figure 8C). In the KEGG enrichment pathway, signal pathways, such as those involved in small cell lung cancer, autophagy, legionellosis, shigellosis, glycosylphosphatidylinositol anchor biosynthesis, and the HIV-1 viral life cycle, and the p53 signaling pathway, were significantly enriched in the blue module (*p *< 0.05); ribosome components, metabolic pathways, oxidative physiology, reactive oxygen specifications, peroxisomes, and purine metabolism were significantly enriched in the brown module (*p *< 0.05); and the MAPK signaling pathway, maturity-onset diabetes of the young, calcium signaling pathway, GABAergic synapse, neuroactive ligand-receptor interaction, B-cell receptor signaling pathway, retrograde endocannabinoid signaling, phototransduction, taste transduction, and oxytocin signaling pathway were significantly enriched in the gray module (*p *< 0.05; Supplementary Table S6).

### 3.7 Characteristics of aggregated patient subgroups

To obtain the characteristics of each subgroup more intuitively, Table 2 summarizes the clinical characteristics, unique DEGs, and possible effective treatment methods and shows different clinical and molecular characteristics among the subgroups, indicating that patients with PE can be divided into two subgroups.

**Table 2:** Summary of characteristics of the subgroups.

Category	Characteristics
Subgroup I	
Clinical features	Younger gestational age, severe symptoms, induced labor, higher risk of fetal growth restriction or malformation
Unique DEGs	*IQGAP1*, *USP38*, *ACTR3*, *RB1CC1*, *NMD3*, *PPP1R15B*, *VPS4B*, *RANBP6*, *CLTC*, *CTSO*
Effective therapeutic method	Combined treatment
Subgroup II	
Clinical features	Older gestational age, mild symptoms, spontaneous labor
Unique gene	*C12orf10*, *CHCHD5*, *ERAL1*, *MRPL28*, *MRPL2*, *ERCC1*, *S100A16*, *TCEB2*, *NDUFB7*, *NDUFA11*
Effective therapeutic method	Calcium ion blocker, magnesium therapy

## 4 Discussion

Over the past few decades, extensive research has been conducted on PE. However, the subtypes of patients and therapeutic biomarkers for PE remain unclear. Identifying the molecular subpopulations of patients with PE is crucial for disease prevention and treatment. To the best of our knowledge, this study is the first to use bioinformatics methods to divide patients with PE into two molecular subgroups and to analyze the subgroups' clinical characteristics, unique DEGs, as well as GO and KEGG enrichment pathways for identifying the feasibility of grouping.

The clinical feature analysis of subgroup I showed that patients in this subgroup had a short gestational week and severe symptoms at the time of onset, which is related to abnormalities in the reactive oxygen species (ROS)-autophagy axis during pregnancy (18). ROS are products of aerobic energy metabolism and have critical antioxidant activities. Excessive ROS levels can lead to oxidative stress during early pregnancy. Autophagy induces trophoblasts to enter a hypoxic placental environment. Abnormal regulation of the ROS-autophagy axis leads to abnormal autophagy activity, thereby causing PE and intrauterine growth restriction. At the end of pregnancy, the ROS-autophagy interaction changes and is involved in the delivery process. In addition, WGCNA results showed that severe symptoms and lower gestational weeks were significantly positively correlated with the blue module, which was significantly overexpressed in subgroup I; this again indicates that patients in subgroup I had more severe symptoms. DEGs associated with lower gestational weeks and worsening symptoms helped the establishment of subgroup I. A previous study showed no significant correlation between the risk of PE and sex of newborns after analyzing the correlation between them (19). Furthermore, another study on the impact of fetal sex in high-risk premature pregnant women with PE showed no significant relationship between the sex of premature infants and the risk of PE (20). After comparing the sexes of newborns in the subgroups, no significant difference was found between these groups, indicating that the sex of newborns was not an independent factor in distinguishing the subgroups of patients with PE.

GTPase-activating protein 1 containing an IQ motif (IQGAP1) is a member of the IQGAP protein family and is evolutionarily conserved in eukaryotes. It was first recognized in human osteosarcoma tissues (21). Owing to its similarity in sequence to GTPase-activating protein (GAP), it is considered a type of GAP that inhibits the inherent GTPase activity of binding partners and stabilizes the active form of the G protein (22). GAP also participates in various biological activities, such as cell–cell adhesion, cytoskeleton dynamics, and cell invasion (23). Our study showed that IQGAP1 was significantly upregulated in subgroup I and had multiple protein pair nodes, indicating its critical role in PE occurrence and development. Placental hypoxia is one of the characteristics of PE. Studies have shown that IQGAP1 is upregulated in macrophages and endothelial cells of hypoxic tissue and can mediate neovascularization by regulating inflammatory cell infiltration and ROS production in hypoxic tissue (24). In hypoxic placental tissues and perivascular areas, the recruitment of endothelial cells or macrophages is impaired, which is related to reduced expression of vascular endothelial growth factor derived from macrophages, affecting angiogenesis and tissue repair in ischemic tissues. IQGAP1-dependent macrophage recruitment plays a crucial role in neovascularization, facilitating post-ischemic and hypoxia blood flow reconstruction and tissue repair. It is also considered a valuable therapeutic target for ischemic vascular diseases (24). In addition, as a scaffold protein for extracellular signal-regulated kinase (ERK), IQGAP1 regulates growth factor-stimulated ERK activity by binding to ERK2, mediates the binding of survival signals to cardiac mast cells, and promotes left ventricular remodeling after pressure overload (22). These findings suggest that IQGAP1 is a potential biomarker for PE.

In subgroup I, some molecules associated with fetal growth restriction exhibited significant differential upregulation. Phosphorylation of the protein phosphatase 1 regulatory subunit 15B (PPP1R15B) is a conserved cellular response to stress that inhibits overall gene translation and overexpression. This phosphorylation is crucial for embryonic development (25). Cells cannot transition from the G1 phase to the S phase following *PPP1R15B* silencing, leading to decreased proliferation and apoptosis (26). A homozygous mutation in the conserved region of the *PPP1R15B* gene (c.1972G>A; p. Arg658Cys) in twin brothers results in microcephaly, short stature, spinal cord dysplasia, and intellectual disabilities (27). Abdulkarim et al. (28) reported that after the R658C mutation in the *PPP1R15B* gene in two children, a conserved amino acid in the binding region of protein phosphatase-1 was affected, reducing PPP1R15B dephosphorylation and leading to β-cell apoptosis, small head disease, short stature, intellectual disability, and diabetes. Vacuolar protein sorting 4B (VPS4B) is a member of the ATPase protein family and a crucial component of the sorting complex that regulates membrane protein internalization and lysosomal degradation (29). VPS4B is closely related to dentin dysplasia pathogenesis (30, 31). VPS4B can participate in cell proliferation and act as a regulator of Wnt-β-catenin signaling in human gingival fibroblasts. The upstream transducer of the catenin signaling pathway promotes cementum formation. Mutations in the *β-catenin* gene cause a significant decrease in RNA transcription and protein function, leading to the loss of target cell function (30). In mice, homozygous deletion of VPS4B led to embryonic death during early pregnancy (29); this may be because VPS4B knockout, which interferes with RNA transcription in embryos, results in changes in the transcription levels of genes related to apoptosis, cell proliferation, and endocytosis; this affects mouse prenatal death. Clathrin heavy chain (CLTC), a component of clathrin, is a vesicular protein involved in intracellular transport and endocytosis. It is related to human neurological/developmental diseases, with mutations mainly causing phenotypes such as microcephaly, developmental delay, and intellectual impairment (32, 33). Based on these research results, we speculate that patients in subgroup 1 are more susceptible to fetal growth restriction or developmental abnormalities.

Mitochondria are double-membrane organelles in eukaryotic cells and the main sites of ATP production. They are involved in various cellular processes, including protein and fatty acid synthesis, calcium ion balance regulation, redox reactions, and cell apoptosis (34). Mitochondria are highly sensitive to hypoxic environments and undergo a series of reactions when exposed to adaptation or stress (35). The mother supplies oxygen and nutrition to the fetus via the placenta, which plays a crucial role in pregnancy. The maintenance of placental function depends highly on mitochondria-generated energy. PE originates from placental dysfunction (36). Therefore, mitochondrial abnormalities are the primary cause of placental dysfunction. Mitochondrial damage caused by bioactive factors released by the placenta can lead to endothelial dysfunction and increased maternal blood pressure (37). In subgroup Ⅱ, NADH:ubiquinone oxidoreductase subunit A11 (NDUFA11) is one of the subunits of mitochondrial respiratory chain complex I, which is crucial for maintaining the balance of mitochondrial energy metabolism and is involved in regulating mitochondrial respiratory function, cell apoptosis, and oxidative stress response (38). NDUFA11 deficiency is associated with various diseases, including NDUFA11-deficient mitochondrial disease and atherosclerotic disease (39). Another subunit of the mitochondrial respiratory chain complex I, NDUFB7, was significantly upregulated in subgroup II. Correia et al. (40) reported a patient with a dual-allele mutation (c.113-10C>G) in the NDUFB7 intron, which resulted in a significant decrease in NDUFB7 protein function and reduced complex I activity, leading to fetal intrauterine growth restriction, anemia, postpartum hypertrophic cardiomyopathy, and severe lactate acidosis. In addition, another unique gene in subgroup II, *Escherichia coli* Ras protein-like 1 (ERAL1), is also associated with mitochondrial ribosomal components (41). ERAL1 deficiency increases mitochondrial superoxide production, reduces mitochondrial membrane potential, inhibits cell growth, and induces apoptosis. These findings suggest that placental dysfunction is possibly the main target organ of damage in subgroup II patients.

In subgroup II, the GO and KEGG enrichment pathways significantly enriched calcium ion channel activity. Calcium channel blockers dilate small arteries and inhibit coronary vasospasms. The 2021 International Guidelines for the Treatment of Pregnancy-induced Hypertension have identified oral nifedipine (a calcium blocker) as a first-line treatment for PE (42). Magnesium therapy is most widely used to prevent seizures; the underlying mechanisms include blocking calcium ion channels, relaxing blood vessels, and altering neurotransmitter activity, which can significantly reduce maternal blood pressure (1). Therefore, calcium antagonists and magnesium therapy may be more effective for subgroup II patients.

The strengths of our research lie in the analysis of the microarray dataset of PE patients through multiple reliable bioinformatics methods, elucidating possible subtypes of PE patients from a molecular perspective and introducing the characteristics of various subtypes. However, this study has some limitations. First, this study mainly analyzed microarray datasets in public databases; the unique genes of each subgroup need to be validated through cytology, zoology, and even human tissue specimens. Second, the sample size included in this study was limited, and the downloaded patient information lacked clinical features such as blood pressure, urinary protein status, platelet count, and liver and kidney functions; thus, further evaluation of these clinical features in subgroups was impossible. Therefore, before these results are used in clinical practice, they need to be validated in a larger cohort to confirm the robustness of the results. In addition, the construction of molecular subgroups requires a combination of proteomics and metabolomics analysis to improve the accuracy of grouping.

## 5 Conclusion

In this study, we used transcriptome data to classify PE patients into two subgroups, analyze the differences between subgroups, and identify the unique DEGs for each subgroup. Our findings provide insights into the diagnosis and personalized treatment of PE.

## Data Availability

The datasets presented in this study can be found in online repositories. The names of the repository/repositories and accession number(s) can be found in the article/**Supplementary Material**.
