# Supplementary material for: Identifying molecular subgroups of patients with preeclampsia through bioinformatics
Source: Front Cardiovasc Med. 2024 Jun 3;11:1367578. doi: 10.3389/fcvm.2024.1367578 (PMC11180819; doi:10.3389/fcvm.2024.1367578)
Supplement: Supplementary file 1 [file Table1.docx]

**Supplementary Table S1.** Clinical characteristics of patients with preeclampsia

| Id | Spontaneous | Severe | Male | Age (years) | Weeks |
| --- | --- | --- | --- | --- | --- |
| GSM106276 |  | 0 |  |  |  |
| GSM106277 |  | 0 |  |  |  |
| GSM106278 |  | 0 |  |  |  |
| GSM106279 |  | 0 |  |  |  |
| GSM106280 |  | 0 |  |  |  |
| GSM106281 |  | 1 |  |  |  |
| GSM106282 |  | 1 |  |  |  |
| GSM106283 |  | 1 |  |  |  |
| GSM106284 |  | 1 |  |  |  |
| GSM106285 |  | 1 |  |  |  |
| GSM1177776 |  | 1 |  | 32 | 36 |
| GSM1177777 |  |  |  | 32 | 37 |
| GSM1177778 |  |  |  | 25 | 33 |
| GSM1177779 |  | 1 |  | 30 | 28 |
| GSM1177780 |  |  |  | 28 | 34 |
| GSM1177781 |  | 1 |  | 27 | 31 |
| GSM1177782 |  |  |  | 23 | 38 |
| GSM1177783 |  |  |  | 21 | 34 |
| GSM1177784 |  |  |  | 31 | 37 |
| GSM1177785 |  | 1 |  | 21 | 27 |
| GSM1177786 |  | 0 |  | 34 | 36 |
| GSM1177787 |  | 1 |  | 27 | 29 |
| GSM1177788 |  | 0 |  | 31 | 34 |
| GSM1177789 |  |  |  | 27 | 34 |
| GSM1177790 |  | 1 |  | 32 | 34 |
| GSM1177791 |  | 0 |  | 28 | 36 |
| GSM1177792 |  | 1 |  | 21 | 31 |
| GSM1177793 |  |  |  | 33 | 37 |
| GSM1177794 |  | 1 |  | 33 | 33 |
| GSM1177795 |  |  |  | 31 | 33 |
| GSM1177796 |  | 1 |  | 38 | 37 |
| GSM1177797 |  | 0 |  | 43 | 34 |
| GSM1177798 |  |  |  | 23 | 31 |
| GSM1177799 |  |  |  | 29 | 31 |
| GSM1177800 |  |  |  | 26 | 16 |
| GSM1177801 |  |  |  | 29 | 18 |
| GSM1177802 |  |  |  | 34 | 38 |
| GSM1177803 |  | 1 |  | 27 | 33 |
| GSM1177804 |  |  |  | 27 | 40 |
| GSM1177805 |  |  |  | 28 | 23 |
| GSM1177806 |  | 0 |  | 37 | 31 |
| GSM1177807 |  | 1 |  | 29 | 34 |
| GSM1177808 |  | 1 |  | 40 | 38 |
| GSM1177809 |  |  |  | 31 | 38 |
| GSM1177810 |  |  |  | 26 | 36 |
| GSM1177811 |  | 1 |  | 29 | 35 |
| GSM1618441 |  |  | 1 |  | 30.85 |
| GSM1618442 |  |  | 1 |  | 34 |
| GSM1618443 |  |  | 1 |  | 31 |
| GSM1618444 |  |  | 0 |  | 35.28 |
| GSM1618445 |  |  | 0 |  | 26.85 |
| GSM1618446 |  | 0 | 0 |  | 29.14 |
| GSM1618447 |  | 0 | 1 |  | 30.28 |
| GSM1618448 |  | 0 | 0 |  | 32.14 |
| GSM1618449 |  | 0 | 0 |  | 35 |
| GSM1618450 |  | 0 | 0 |  | 32.57 |
| GSM1618451 |  | 0 | 0 |  | 34.86 |
| GSM1618452 |  | 1 | 1 |  | 29.28 |
| GSM1618453 |  | 1 | 0 |  | 33.14 |
| GSM1618454 |  | 1 | 1 |  | 28.86 |
| GSM1618455 |  | 1 | 1 |  | 33.57 |
| GSM1618456 |  | 1 | 1 |  | 32 |
| GSM1618457 |  | 1 | 1 |  | 29.42 |
| GSM225481 |  | 1 |  |  |  |
| GSM225498 |  | 1 |  |  |  |
| GSM225499 |  | 1 |  |  |  |
| GSM225500 |  | 1 |  |  |  |
| GSM225501 |  | 1 |  |  |  |
| GSM225502 |  | 1 |  |  |  |
| GSM225503 |  | 1 |  |  |  |
| GSM225504 |  | 1 |  |  |  |
| GSM225505 |  | 1 |  |  |  |
| GSM225506 |  | 1 |  |  |  |
| GSM225507 |  | 1 |  |  |  |
| GSM225508 |  | 1 |  |  |  |
| GSM225509 |  | 1 |  |  |  |
| GSM225510 |  | 1 |  |  |  |
| GSM225511 |  | 1 |  |  |  |
| GSM225512 |  | 1 |  |  |  |
| GSM225513 |  | 1 |  |  |  |
| GSM2420587 |  |  |  | 37 | 39 |
| GSM2420588 |  |  |  | 34 | 40 |
| GSM2420589 |  |  |  | 34 | 40 |
| GSM2420590 |  |  |  | 31 | 39 |
| GSM2420591 |  |  |  | 31 | 39 |
| GSM2420592 |  |  |  | 39 | 38 |
| GSM2420593 |  |  |  | 39 | 38 |
| GSM2420594 |  |  |  | 36 | 38 |
| GSM2420595 |  |  |  | 41 | 40 |
| GSM2420596 |  |  |  | 41 | 40 |
| GSM2420597 |  |  |  | 38 | 39 |
| GSM2420598 |  |  |  | 38 | 39 |
| GSM2420599 |  | 1 |  | 21 | 38.1 |
| GSM2420600 |  | 1 |  | 36 | 36.4 |
| GSM2420601 |  | 1 |  | 36 | 36.4 |
| GSM2420602 |  | 1 |  | 35 | 36 |
| GSM2420603 |  | 1 |  | 35 | 36 |
| GSM2420604 |  | 1 |  | 31 | 37 |
| GSM2420605 |  | 1 |  | 31 | 37 |
| GSM2420606 |  | 1 |  | 38 | 38 |
| GSM2747897 |  | 1 |  | 30 |  |
| GSM2747898 |  | 1 |  | 30 |  |
| GSM2747899 |  | 1 |  | 30 |  |
| GSM2747900 |  |  |  | 30 |  |
| GSM2747901 |  |  |  | 30 |  |
| GSM2747902 |  |  |  | 30 |  |
| GSM4445711 |  | 1 |  | 30 |  |
| GSM4445712 |  | 1 |  | 33 |  |
| GSM4445713 |  | 1 |  | 38 |  |
| GSM4445714 |  | 1 |  | 30 |  |
| GSM4445715 |  | 1 |  | 31 |  |
| GSM4445716 |  | 1 |  | 27 |  |
| GSM4445717 |  | 1 |  | 25 |  |
| GSM4445718 |  | 1 |  | 40 |  |
| GSM4445719 |  | 1 |  | 30 |  |
| GSM4445720 |  | 1 |  | 29 |  |
| GSM4445721 |  | 1 |  | 30 |  |
| GSM4445722 |  | 1 |  | 20 |  |
| GSM4445723 |  | 1 |  | 33 |  |
| GSM4445724 |  |  |  | 31 |  |
| GSM4445725 |  |  |  | 30 |  |
| GSM4445726 |  |  |  | 25 |  |
| GSM4445727 |  |  |  | 24 |  |
| GSM4445728 |  |  |  | 25 |  |
| GSM4445729 |  |  |  | 37 |  |
| GSM4445730 |  |  |  | 34 |  |
| GSM4445731 |  |  |  | 39 |  |
| GSM4445732 |  |  |  | 25 |  |
| GSM4445733 |  |  |  | 30 |  |
| GSM4445734 |  |  |  | 26 |  |
| GSM4445735 |  |  |  | 18 |  |
| GSM4445736 |  |  |  | 28 |  |
| GSM4445737 |  |  |  | 28 |  |
| GSM4445738 |  |  |  | 27 |  |
| GSM5086567 |  |  |  | 32 |  |
| GSM5086568 |  |  |  | 28 |  |
| GSM5086569 |  |  |  | 29 |  |
| GSM5086570 |  |  |  | 30 |  |
| GSM5086571 |  |  |  | 32 |  |
| GSM5086572 |  |  |  | 33 |  |
| GSM635904 | 1 |  | 0 |  | 36 |
| GSM635905 | 0 |  | 0 |  | 37 |
| GSM635906 | 0 |  | 1 |  | 37 |
| GSM635907 | 0 |  | 1 |  | 34 |
| GSM635908 | 0 |  | 1 |  | 38 |
| GSM635909 | 1 |  | 0 |  | 37 |
| GSM635910 | 1 |  | 0 |  | 37 |
| GSM635911 | 1 |  | 1 |  | 39 |
| GSM635912 | 0 |  | 0 |  | 27 |
| GSM635913 | 1 |  | 1 |  | 38 |
| GSM635914 | 1 |  | 0 |  | 38 |
| GSM635915 | 0 |  | 1 |  | 34 |
| GSM635916 | 1 |  | 0 |  | 39 |
| GSM635917 | 1 |  | 0 |  | 37 |
| GSM635918 | 0 |  | 0 |  | 38 |
| GSM635919 | 0 |  | 1 |  | 39 |
| GSM635920 | 0 |  | 0 |  | 35 |
| GSM635921 | 1 |  | 1 |  | 37 |
| GSM635922 | 0 |  | 0 |  | 32 |
| GSM635923 | 0 |  | 1 |  | 34 |
| GSM635924 | 0 |  | 1 |  | 38 |
| GSM635925 | 1 |  | 1 |  | 37 |
| GSM635926 | 0 |  | 1 |  | 36 |
| GSM635927 | 0 |  | 0 |  | 40 |
| GSM635928 | 1 |  | 1 |  | 38 |
| GSM635929 | 0 |  | 1 |  | 39 |
| GSM635930 | 0 |  | 0 |  | 37 |
| GSM635931 | 1 |  | 0 |  | 34 |
| GSM635932 | 1 |  | 1 |  | 36 |
| GSM635933 | 1 |  | 0 |  | 38 |
| GSM635934 | 1 |  | 1 |  | 39 |
| GSM635935 | 1 |  | 1 |  | 38 |
| GSM635936 | 1 |  | 0 |  | 35 |
| GSM635937 | 0 |  | 1 |  | 29 |
| GSM635938 | 1 |  | 1 |  | 36 |
| GSM635939 | 1 |  | 1 |  | 39 |
| GSM635940 | 0 |  | 0 |  | 28 |
| GSM635941 | 0 |  | 1 |  | 39 |
| GSM635942 | 1 |  | 0 |  | 38 |
| GSM635943 | 1 |  | 0 |  | 32 |
| GSM635944 | 1 |  | 0 |  | 39 |
| GSM635945 | 1 |  | 1 |  | 34 |
| GSM635946 | 1 |  | 1 |  | 38 |
| GSM635947 | 0 |  | 0 |  | 33 |
| GSM635948 | 1 |  | 1 |  | 29 |
| GSM635949 | 0 |  | 0 |  | 40 |
| GSM635950 | 1 |  | 0 |  | 39 |
| GSM635951 | 0 |  | 0 |  | 27 |
| GSM635952 | 1 |  | 1 |  | 38 |
| GSM635953 | 1 |  | 1 |  | 39 |
| GSM635954 | 1 |  | 1 |  | 39 |
| GSM635955 | 1 |  | 0 |  | 39 |
| GSM635956 | 1 |  | 1 |  | 37 |
| GSM635957 | 1 |  | 1 |  | 35 |
| GSM635958 | 0 |  | 0 |  | 37 |
| GSM635959 | 1 |  | 1 |  | 38 |
| GSM635960 | 0 |  | 1 |  | 38 |
| GSM635961 | 1 |  | 0 |  | 38 |
| GSM635962 | 1 |  | 0 |  | 37 |
| GSM635963 | 1 |  | 0 |  | 38 |
