# Supplementary material for: Identifying molecular subgroups of patients with preeclampsia through bioinformatics
Source: Front Cardiovasc Med. 2024 Jun 3;11:1367578. doi: 10.3389/fcvm.2024.1367578 (PMC11180819; doi:10.3389/fcvm.2024.1367578)
Supplement: Supplementary file 2 [file Table2.docx]

**Supplementary Table S2.** Differential expression genes between subgroup I and control group

| Gene |  | meanDiff | AveExpr | t | P.Value | adj.P.Val | B |
| --- | --- | --- | --- | --- | --- | --- | --- |
| GCLM |  | -0.555 | 9.367 | -8.575 | 0.00E+00 | 0.00E+00 | 24.76 |
| LEP |  | 1.489 | 8.583 | 7.933 | 0.00E+00 | 0.00E+00 | 20.776 |
| GOT1 |  | -0.266 | 9.346 | -6.947 | 0.00E+00 | 0.00E+00 | 15.017 |
| CST6 |  | 0.727 | 8.064 | 6.734 | 0.00E+00 | 0.00E+00 | 13.841 |
| ENG |  | 0.535 | 10.195 | 6.445 | 0.00E+00 | 0.00E+00 | 12.287 |
| SSNA1 |  | 0.29 | 9.744 | 6.361 | 0.00E+00 | 0.00E+00 | 11.84 |
| MAP4K3 |  | -0.326 | 8.054 | -6.066 | 0.00E+00 | 0.00E+00 | 10.311 |
| FLT1 |  | 0.556 | 8.785 | 5.936 | 0.00E+00 | 0.00E+00 | 9.659 |
| CLDN1 |  | -0.729 | 7.616 | -5.926 | 0.00E+00 | 0.00E+00 | 9.606 |
| HMGCS1 |  | 0.356 | 8.322 | 5.872 | 0.00E+00 | 0.00E+00 | 9.344 |
| TGM1 |  | 0.42 | 6.4 | 5.867 | 0.00E+00 | 0.00E+00 | 9.319 |
| SERPINB2 |  | -0.565 | 9.997 | -5.779 | 0.00E+00 | 0.00E+00 | 8.877 |
| ISOC2 |  | 0.283 | 8.429 | 5.681 | 0.00E+00 | 0.00E+00 | 8.409 |
| PAPPA2 |  | 0.527 | 8.053 | 5.67 | 0.00E+00 | 0.00E+00 | 8.354 |
| RSU1 |  | -0.228 | 8.182 | -5.626 | 0.00E+00 | 0.00E+00 | 8.135 |
| PHPT1 |  | 0.22 | 11.089 | 5.556 | 0.00E+00 | 0.00E+00 | 7.809 |
| COMTD1 |  | 0.289 | 9.338 | 5.505 | 0.00E+00 | 0.00E+00 | 7.568 |
| INHA |  | 0.527 | 7.741 | 5.316 | 0.00E+00 | 0.00E+00 | 6.686 |
| ABCA7 |  | 0.333 | 7.23 | 5.295 | 0.00E+00 | 0.00E+00 | 6.59 |
| TCEAL8 |  | 0.209 | 10.058 | 5.27 | 0.00E+00 | 0.00E+00 | 6.478 |
| HMGN3 |  | 0.234 | 9.44 | 5.258 | 0.00E+00 | 0.00E+00 | 6.422 |
| AP1S1 |  | 0.337 | 7.951 | 5.199 | 0.00E+00 | 0.00E+00 | 6.152 |
| TREM1 |  | 0.557 | 7.89 | 5.175 | 0.00E+00 | 0.00E+00 | 6.045 |
| ARF5 |  | 0.199 | 10.808 | 5.096 | 0.00E+00 | 0.00E+00 | 5.692 |
| BMP6 |  | -0.346 | 8.488 | -5.092 | 0.00E+00 | 0.00E+00 | 5.67 |
| CNOT2 |  | -0.165 | 9.411 | -5.069 | 0.00E+00 | 0.00E+00 | 5.568 |
| RDH13 |  | 0.44 | 8.51 | 5.017 | 0.00E+00 | 0.00E+00 | 5.344 |
| MRPS30 |  | -0.209 | 8.417 | -5.011 | 0.00E+00 | 0.00E+00 | 5.314 |
| SASH1 |  | 0.33 | 9.258 | 4.996 | 0.00E+00 | 0.00E+00 | 5.253 |
| SLC23A2 |  | -0.28 | 7.539 | -4.987 | 0.00E+00 | 0.00E+00 | 5.21 |
| F13A1 |  | -0.352 | 10.446 | -4.957 | 0.00E+00 | 0.00E+00 | 5.077 |
| TUBAL3 |  | -0.34 | 5.678 | -4.928 | 0.00E+00 | 0.00E+00 | 4.953 |
| FAM50A |  | 0.179 | 10.53 | 4.898 | 0.00E+00 | 0.00E+00 | 4.828 |
| GPX2 |  | -0.357 | 6.08 | -4.854 | 0.00E+00 | 0.00E+00 | 4.634 |
| ZNF185 |  | -0.27 | 9.265 | -4.839 | 0.00E+00 | 0.00E+00 | 4.572 |
| CITED2 |  | 0.332 | 9.999 | 4.819 | 0.00E+00 | 0.00E+00 | 4.489 |
| MMP14 |  | 0.367 | 8.018 | 4.813 | 0.00E+00 | 0.00E+00 | 4.467 |
| ACO1 |  | -0.193 | 10.022 | -4.795 | 0.00E+00 | 0.00E+00 | 4.386 |
| CCNT2 |  | -0.228 | 7.498 | -4.785 | 0.00E+00 | 0.00E+00 | 4.342 |
| SCD |  | 0.32 | 8.575 | 4.778 | 0.00E+00 | 0.00E+00 | 4.318 |
| ZNF217 |  | -0.243 | 9.856 | -4.779 | 0.00E+00 | 0.00E+00 | 4.316 |
| CALML4 |  | -0.233 | 7.45 | -4.774 | 0.00E+00 | 0.00E+00 | 4.297 |
| PLAGL2 |  | -0.165 | 8.304 | -4.762 | 0.00E+00 | 0.00E+00 | 4.246 |
| PUM1 |  | -0.148 | 11.134 | -4.739 | 0.00E+00 | 0.00E+00 | 4.149 |
| CSF2 |  | -0.535 | 5.159 | -4.732 | 0.00E+00 | 0.00E+00 | 4.122 |
| ABHD8 |  | 0.272 | 8.926 | 4.729 | 0.00E+00 | 0.00E+00 | 4.113 |
| CDC42EP2 |  | -0.252 | 8.913 | -4.723 | 0.00E+00 | 0.00E+00 | 4.082 |
| SCARB1 |  | 0.246 | 11.128 | 4.702 | 0.00E+00 | 0.00E+00 | 4.001 |
| ACOXL |  | -0.473 | 5.656 | -4.65 | 0.00E+00 | 0.00E+00 | 3.779 |
| E2F6 |  | -0.186 | 8.256 | -4.608 | 0.00E+00 | 0.00E+00 | 3.609 |
| MID1IP1 |  | -0.252 | 8.549 | -4.59 | 0.00E+00 | 0.00E+00 | 3.533 |
| P4HA1 |  | 0.253 | 8.65 | 4.581 | 0.00E+00 | 0.00E+00 | 3.502 |
| C10orf10 |  | -0.418 | 9.253 | -4.578 | 0.00E+00 | 0.00E+00 | 3.485 |
| PMM1 |  | 0.184 | 9.098 | 4.572 | 0.00E+00 | 0.00E+00 | 3.467 |
| B3GALT4 |  | 0.255 | 8.806 | 4.549 | 0.00E+00 | 1.00E-03 | 3.372 |
| SDF2L1 |  | 0.253 | 11.366 | 4.547 | 0.00E+00 | 1.00E-03 | 3.365 |
| MIF |  | 0.183 | 13.57 | 4.538 | 0.00E+00 | 1.00E-03 | 3.326 |
| ARHGEF4 |  | 0.438 | 5.867 | 4.535 | 0.00E+00 | 1.00E-03 | 3.315 |
| C18orf8 |  | -0.196 | 9.215 | -4.536 | 0.00E+00 | 1.00E-03 | 3.315 |
| VNN3 |  | -0.434 | 6.145 | -4.522 | 0.00E+00 | 1.00E-03 | 3.26 |
| PROCR |  | 0.384 | 10.286 | 4.519 | 0.00E+00 | 1.00E-03 | 3.25 |
| NDRG1 |  | 0.366 | 10.694 | 4.515 | 0.00E+00 | 1.00E-03 | 3.236 |
| PSMA1 |  | -0.139 | 10.313 | -4.513 | 0.00E+00 | 1.00E-03 | 3.224 |
| PTPRB |  | -0.243 | 7.19 | -4.486 | 0.00E+00 | 1.00E-03 | 3.115 |
| NPL |  | -0.21 | 8.651 | -4.46 | 0.00E+00 | 1.00E-03 | 3.011 |
| BCL2 |  | -0.247 | 7.728 | -4.452 | 0.00E+00 | 1.00E-03 | 2.979 |
| POLR2H |  | 0.157 | 10.579 | 4.44 | 0.00E+00 | 1.00E-03 | 2.935 |
| GALK1 |  | 0.253 | 8.793 | 4.435 | 0.00E+00 | 1.00E-03 | 2.918 |
| FKBP2 |  | 0.206 | 11.219 | 4.432 | 0.00E+00 | 1.00E-03 | 2.904 |
| RAB11FIP3 |  | 0.209 | 8.529 | 4.432 | 0.00E+00 | 1.00E-03 | 2.903 |
| CNDP2 |  | -0.17 | 10.506 | -4.432 | 0.00E+00 | 1.00E-03 | 2.901 |
| CFDP1 |  | 0.182 | 9.976 | 4.425 | 0.00E+00 | 1.00E-03 | 2.875 |
| SSTR2 |  | -0.217 | 5.56 | -4.416 | 0.00E+00 | 1.00E-03 | 2.837 |
| THBS3 |  | 0.248 | 7.9 | 4.4 | 0.00E+00 | 1.00E-03 | 2.779 |
| PTN |  | 0.297 | 8.868 | 4.398 | 0.00E+00 | 1.00E-03 | 2.771 |
| TFG |  | -0.163 | 10.692 | -4.395 | 0.00E+00 | 1.00E-03 | 2.756 |
| BCAT2 |  | 0.243 | 8.577 | 4.373 | 0.00E+00 | 1.00E-03 | 2.671 |
| RGS20 |  | -0.339 | 6.223 | -4.373 | 0.00E+00 | 1.00E-03 | 2.667 |
| PUSL1 |  | 0.164 | 8.965 | 4.366 | 0.00E+00 | 1.00E-03 | 2.644 |
| CHCHD1 |  | 0.196 | 10.1 | 4.365 | 0.00E+00 | 1.00E-03 | 2.642 |
| GABARAPL2 |  | -0.17 | 11.397 | -4.361 | 0.00E+00 | 1.00E-03 | 2.622 |
| ILVBL |  | 0.224 | 10.635 | 4.353 | 0.00E+00 | 1.00E-03 | 2.593 |
| FSTL3 |  | 0.52 | 10.029 | 4.348 | 0.00E+00 | 1.00E-03 | 2.573 |
| SYNPO2L |  | -0.39 | 6.416 | -4.346 | 0.00E+00 | 1.00E-03 | 2.562 |
| DNM2 |  | 0.159 | 9.28 | 4.337 | 0.00E+00 | 1.00E-03 | 2.533 |
| ADAMTS3 |  | -0.34 | 5.997 | -4.289 | 0.00E+00 | 2.00E-03 | 2.342 |
| IGSF8 |  | 0.298 | 7.569 | 4.274 | 0.00E+00 | 2.00E-03 | 2.289 |
| PTGIR |  | -0.22 | 7.426 | -4.259 | 0.00E+00 | 2.00E-03 | 2.227 |
| TBC1D22A |  | 0.159 | 8.282 | 4.255 | 0.00E+00 | 2.00E-03 | 2.216 |
| SSR4 |  | 0.295 | 12.4 | 4.253 | 0.00E+00 | 2.00E-03 | 2.21 |
| AP1G2 |  | 0.228 | 8.942 | 4.252 | 0.00E+00 | 2.00E-03 | 2.203 |
| PPIG |  | 0.275 | 8.575 | 4.243 | 0.00E+00 | 2.00E-03 | 2.171 |
| AMOTL2 |  | 0.205 | 8.537 | 4.234 | 0.00E+00 | 2.00E-03 | 2.135 |
| ERO1L |  | 0.283 | 8.489 | 4.212 | 0.00E+00 | 2.00E-03 | 2.051 |
| BCL6 |  | 0.358 | 9.744 | 4.211 | 0.00E+00 | 2.00E-03 | 2.048 |
| LIMD1 |  | 0.29 | 6.633 | 4.204 | 0.00E+00 | 2.00E-03 | 2.022 |
| SLC1A1 |  | -0.267 | 6.769 | -4.201 | 0.00E+00 | 2.00E-03 | 2.007 |
| LSM7 |  | 0.203 | 11.155 | 4.192 | 0.00E+00 | 2.00E-03 | 1.977 |
| SNX27 |  | -0.169 | 9.743 | -4.191 | 0.00E+00 | 2.00E-03 | 1.971 |
| SERTAD4 |  | 0.402 | 6.978 | 4.183 | 0.00E+00 | 2.00E-03 | 1.944 |
| DNAJB2 |  | 0.168 | 9.58 | 4.177 | 0.00E+00 | 2.00E-03 | 1.921 |
| EPHB3 |  | 0.292 | 6.851 | 4.169 | 0.00E+00 | 2.00E-03 | 1.89 |
| TCEAL4 |  | 0.205 | 10.781 | 4.167 | 0.00E+00 | 2.00E-03 | 1.883 |
| RABAC1 |  | 0.138 | 12.423 | 4.159 | 0.00E+00 | 2.00E-03 | 1.853 |
| ANG |  | 0.289 | 8.26 | 4.159 | 0.00E+00 | 2.00E-03 | 1.852 |
| PRDX3 |  | -0.176 | 10.429 | -4.147 | 0.00E+00 | 3.00E-03 | 1.803 |
| MYL6 |  | 0.144 | 14.023 | 4.117 | 0.00E+00 | 3.00E-03 | 1.696 |
| IRF3 |  | 0.166 | 8.901 | 4.105 | 0.00E+00 | 3.00E-03 | 1.653 |
| ISOC1 |  | -0.176 | 9.461 | -4.09 | 0.00E+00 | 3.00E-03 | 1.593 |
| SLC25A5 |  | -0.157 | 12.823 | -4.083 | 0.00E+00 | 3.00E-03 | 1.567 |
| QPCT |  | 0.36 | 8.832 | 4.078 | 0.00E+00 | 3.00E-03 | 1.555 |
| CCPG1 |  | 0.2 | 8.027 | 4.074 | 0.00E+00 | 3.00E-03 | 1.538 |
| IER2 |  | 0.195 | 11.478 | 4.069 | 0.00E+00 | 3.00E-03 | 1.522 |
| TMEM45A |  | 0.417 | 8.783 | 4.068 | 0.00E+00 | 3.00E-03 | 1.516 |
| HM13 |  | -0.147 | 8.589 | -4.069 | 0.00E+00 | 3.00E-03 | 1.516 |
| NUBP2 |  | 0.165 | 8.583 | 4.067 | 0.00E+00 | 3.00E-03 | 1.513 |
| SLC11A1 |  | 0.213 | 7.014 | 4.065 | 0.00E+00 | 3.00E-03 | 1.504 |
| PHYHIP |  | 0.304 | 6.202 | 4.063 | 0.00E+00 | 3.00E-03 | 1.498 |
| LCP2 |  | -0.208 | 8.228 | -4.034 | 0.00E+00 | 4.00E-03 | 1.388 |
| SON |  | -0.141 | 8.869 | -4.032 | 0.00E+00 | 4.00E-03 | 1.381 |
| TRIM40 |  | -0.339 | 5.808 | -4.02 | 0.00E+00 | 4.00E-03 | 1.338 |
| LYST |  | 0.16 | 8.145 | 4.019 | 0.00E+00 | 4.00E-03 | 1.337 |
| GPT2 |  | 0.282 | 7.759 | 4.006 | 0.00E+00 | 4.00E-03 | 1.29 |
| STK38 |  | 0.218 | 9.212 | 4.005 | 0.00E+00 | 4.00E-03 | 1.286 |
| NSUN6 |  | 0.228 | 6.943 | 3.993 | 0.00E+00 | 4.00E-03 | 1.242 |
| PPP2CB |  | -0.17 | 9.831 | -3.991 | 0.00E+00 | 4.00E-03 | 1.233 |
| AICDA |  | -0.342 | 6.413 | -3.989 | 0.00E+00 | 4.00E-03 | 1.226 |
| ZNF281 |  | -0.161 | 9.697 | -3.977 | 0.00E+00 | 4.00E-03 | 1.181 |
| PSMF1 |  | -0.15 | 9.17 | -3.97 | 0.00E+00 | 5.00E-03 | 1.158 |
| DAPK3 |  | 0.171 | 9.265 | 3.968 | 0.00E+00 | 5.00E-03 | 1.155 |
| NMT2 |  | 0.247 | 7.951 | 3.961 | 0.00E+00 | 5.00E-03 | 1.127 |
| IGFBP5 |  | 0.282 | 8.839 | 3.96 | 0.00E+00 | 5.00E-03 | 1.126 |
| MAD2L1BP |  | -0.132 | 9.252 | -3.959 | 0.00E+00 | 5.00E-03 | 1.118 |
| HBD |  | -0.404 | 9.25 | -3.953 | 0.00E+00 | 5.00E-03 | 1.095 |
| FLNB |  | 0.261 | 9.283 | 3.95 | 0.00E+00 | 5.00E-03 | 1.087 |
| THAP6 |  | -0.2 | 7.34 | -3.946 | 0.00E+00 | 5.00E-03 | 1.071 |
| OCA2 |  | -0.219 | 5.805 | -3.943 | 0.00E+00 | 5.00E-03 | 1.061 |
| KMO |  | -0.277 | 7.906 | -3.942 | 0.00E+00 | 5.00E-03 | 1.055 |
| MAN1B1 |  | 0.144 | 9.396 | 3.938 | 0.00E+00 | 5.00E-03 | 1.044 |
| SLC4A1AP |  | -0.139 | 8.986 | -3.933 | 0.00E+00 | 5.00E-03 | 1.023 |
| TSNAX |  | -0.157 | 8.863 | -3.922 | 0.00E+00 | 5.00E-03 | 0.986 |
| GSTK1 |  | -0.155 | 10.924 | -3.922 | 0.00E+00 | 5.00E-03 | 0.985 |
| ATPIF1 |  | 0.165 | 11.205 | 3.918 | 0.00E+00 | 5.00E-03 | 0.975 |
| BAX |  | 0.169 | 8.546 | 3.905 | 0.00E+00 | 5.00E-03 | 0.929 |
| MRPL34 |  | 0.147 | 10.267 | 3.905 | 0.00E+00 | 5.00E-03 | 0.928 |
| MFAP5 |  | 0.306 | 9.209 | 3.899 | 0.00E+00 | 5.00E-03 | 0.908 |
| APLN |  | -0.289 | 8.442 | -3.898 | 0.00E+00 | 5.00E-03 | 0.899 |
| MTSS1 |  | -0.209 | 8.184 | -3.893 | 0.00E+00 | 5.00E-03 | 0.882 |
| YY1AP1 |  | -0.163 | 9.836 | -3.88 | 0.00E+00 | 6.00E-03 | 0.837 |
| FOS |  | 0.428 | 10.857 | 3.879 | 0.00E+00 | 6.00E-03 | 0.835 |
| CPT1C |  | 0.262 | 7.116 | 3.876 | 0.00E+00 | 6.00E-03 | 0.825 |
| C21orf2 |  | 0.204 | 7.577 | 3.871 | 0.00E+00 | 6.00E-03 | 0.809 |
| KIT |  | 0.262 | 7.365 | 3.866 | 0.00E+00 | 6.00E-03 | 0.792 |
| SEMA3C |  | -0.326 | 7.045 | -3.865 | 0.00E+00 | 6.00E-03 | 0.784 |
| SLC9A1 |  | -0.195 | 8.773 | -3.862 | 0.00E+00 | 6.00E-03 | 0.773 |
| NCOA2 |  | -0.216 | 6.653 | -3.856 | 0.00E+00 | 6.00E-03 | 0.754 |
| SMAD4 |  | -0.162 | 9.63 | -3.855 | 0.00E+00 | 6.00E-03 | 0.75 |
| PXK |  | -0.202 | 8.078 | -3.852 | 0.00E+00 | 6.00E-03 | 0.738 |
| SPON1 |  | -0.298 | 7.434 | -3.85 | 0.00E+00 | 6.00E-03 | 0.731 |
| CDK10 |  | 0.136 | 7.86 | 3.837 | 0.00E+00 | 6.00E-03 | 0.688 |
| LY6D |  | 0.349 | 6.81 | 3.834 | 0.00E+00 | 6.00E-03 | 0.68 |
| IRF2 |  | -0.169 | 7.349 | -3.827 | 0.00E+00 | 7.00E-03 | 0.651 |
| QPRT |  | 0.176 | 9.451 | 3.824 | 0.00E+00 | 7.00E-03 | 0.644 |
| CRIP1 |  | 0.223 | 10.651 | 3.816 | 0.00E+00 | 7.00E-03 | 0.617 |
| RTN4RL2 |  | -0.22 | 6.658 | -3.804 | 0.00E+00 | 7.00E-03 | 0.57 |
| KLRD1 |  | -0.236 | 6.559 | -3.802 | 0.00E+00 | 7.00E-03 | 0.565 |
| IREB2 |  | -0.159 | 8.326 | -3.799 | 0.00E+00 | 7.00E-03 | 0.553 |
| APBB2 |  | -0.17 | 7.187 | -3.796 | 0.00E+00 | 7.00E-03 | 0.543 |
| WDR47 |  | -0.199 | 7.272 | -3.796 | 0.00E+00 | 7.00E-03 | 0.542 |
| MAP4K5 |  | 0.204 | 8.468 | 3.786 | 0.00E+00 | 7.00E-03 | 0.513 |
| HMGB3 |  | 0.223 | 9.877 | 3.78 | 0.00E+00 | 7.00E-03 | 0.492 |
| CERK |  | 0.193 | 8.135 | 3.777 | 0.00E+00 | 7.00E-03 | 0.481 |
| IL27RA |  | 0.209 | 8.055 | 3.774 | 0.00E+00 | 8.00E-03 | 0.471 |
| TGIF2 |  | 0.224 | 6.951 | 3.772 | 0.00E+00 | 8.00E-03 | 0.464 |
| SMURF1 |  | -0.255 | 6.535 | -3.767 | 0.00E+00 | 8.00E-03 | 0.444 |
| FCER1G |  | -0.241 | 9.546 | -3.767 | 0.00E+00 | 8.00E-03 | 0.442 |
| ASAH1 |  | -0.2 | 9.375 | -3.766 | 0.00E+00 | 8.00E-03 | 0.441 |
| MTF1 |  | -0.159 | 8.301 | -3.76 | 0.00E+00 | 8.00E-03 | 0.419 |
| ULK1 |  | -0.17 | 9.878 | -3.757 | 0.00E+00 | 8.00E-03 | 0.41 |
| DVL2 |  | 0.184 | 8.807 | 3.747 | 0.00E+00 | 8.00E-03 | 0.38 |
| KRT23 |  | -0.224 | 10.302 | -3.745 | 0.00E+00 | 8.00E-03 | 0.367 |
| PNRC1 |  | -0.153 | 9.856 | -3.739 | 0.00E+00 | 8.00E-03 | 0.347 |
| RORA |  | -0.238 | 7.558 | -3.73 | 0.00E+00 | 8.00E-03 | 0.318 |
| SMARCC1 |  | 0.149 | 9.563 | 3.722 | 0.00E+00 | 9.00E-03 | 0.293 |
| APH1A |  | 0.175 | 9.065 | 3.715 | 0.00E+00 | 9.00E-03 | 0.269 |
| KCNJ8 |  | -0.285 | 7.144 | -3.704 | 0.00E+00 | 9.00E-03 | 0.23 |
| STX6 |  | 0.176 | 8.285 | 3.702 | 0.00E+00 | 9.00E-03 | 0.225 |
| TTC1 |  | 0.148 | 9.626 | 3.696 | 0.00E+00 | 9.00E-03 | 0.206 |
| NRCAM |  | -0.215 | 8.155 | -3.695 | 0.00E+00 | 9.00E-03 | 0.2 |
| MBD3 |  | 0.185 | 9.282 | 3.685 | 0.00E+00 | 1.00E-02 | 0.169 |
| GTF2H4 |  | 0.148 | 7.805 | 3.683 | 0.00E+00 | 1.00E-02 | 0.163 |
| NARF |  | 0.131 | 9.777 | 3.682 | 0.00E+00 | 1.00E-02 | 0.158 |
| PRKACG |  | -0.249 | 5.194 | -3.674 | 0.00E+00 | 1.00E-02 | 0.13 |
| IQGAP1 |  | 0.197 | 11.374 | 3.672 | 0.00E+00 | 1.00E-02 | 0.124 |
| PEX6 |  | 0.279 | 8.33 | 3.669 | 0.00E+00 | 1.00E-02 | 0.114 |
| LRIG3 |  | -0.199 | 7.094 | -3.665 | 0.00E+00 | 1.00E-02 | 0.098 |
| PREP |  | 0.164 | 8.964 | 3.664 | 0.00E+00 | 1.00E-02 | 0.097 |
| GNPDA1 |  | -0.173 | 9.236 | -3.663 | 0.00E+00 | 1.00E-02 | 0.091 |
| GNAS |  | -0.128 | 10.189 | -3.655 | 0.00E+00 | 1.00E-02 | 0.065 |
| GSN |  | 0.16 | 9.77 | 3.652 | 0.00E+00 | 1.00E-02 | 0.06 |
| ANK3 |  | -0.247 | 7.66 | -3.651 | 0.00E+00 | 1.00E-02 | 0.052 |
| FGF2 |  | -0.239 | 6.558 | -3.646 | 0.00E+00 | 1.10E-02 | 0.036 |
| GPR68 |  | -0.213 | 7.112 | -3.641 | 0.00E+00 | 1.10E-02 | 0.017 |
| GRIN2C |  | -0.217 | 6.668 | -3.64 | 0.00E+00 | 1.10E-02 | 0.015 |
| ARHGAP9 |  | -0.235 | 8.164 | -3.639 | 0.00E+00 | 1.10E-02 | 0.012 |
| MTIF2 |  | 0.161 | 7.586 | 3.634 | 0.00E+00 | 1.10E-02 | -0.001 |
| RAE1 |  | -0.096 | 9.118 | -3.622 | 0.00E+00 | 1.10E-02 | -0.045 |
| CHST11 |  | -0.156 | 8.262 | -3.61 | 0.00E+00 | 1.20E-02 | -0.086 |
| CRELD1 |  | 0.135 | 8.277 | 3.605 | 0.00E+00 | 1.20E-02 | -0.096 |
| LAP3 |  | -0.254 | 10.49 | -3.604 | 0.00E+00 | 1.20E-02 | -0.106 |
| RAB3IL1 |  | -0.263 | 7.793 | -3.603 | 0.00E+00 | 1.20E-02 | -0.109 |
| FABP6 |  | 0.197 | 5.093 | 3.599 | 0.00E+00 | 1.20E-02 | -0.116 |
| SIN3A |  | -0.129 | 9.361 | -3.591 | 0.00E+00 | 1.20E-02 | -0.147 |
| VNN1 |  | -0.281 | 6.825 | -3.579 | 0.00E+00 | 1.30E-02 | -0.185 |
| PGBD2 |  | 0.236 | 7.981 | 3.572 | 0.00E+00 | 1.30E-02 | -0.207 |
| FBXO28 |  | -0.123 | 8.714 | -3.569 | 0.00E+00 | 1.30E-02 | -0.219 |
| SYT1 |  | -0.226 | 5.983 | -3.567 | 0.00E+00 | 1.30E-02 | -0.227 |
| TKT |  | -0.2 | 12.293 | -3.564 | 0.00E+00 | 1.30E-02 | -0.236 |
| ZNF555 |  | -0.206 | 7.043 | -3.562 | 0.00E+00 | 1.30E-02 | -0.244 |
| PRKAB2 |  | 0.228 | 7.646 | 3.548 | 0.00E+00 | 1.40E-02 | -0.284 |
| BTBD3 |  | -0.205 | 8.706 | -3.547 | 0.00E+00 | 1.40E-02 | -0.291 |
| SDC4 |  | 0.197 | 8.855 | 3.54 | 0.00E+00 | 1.40E-02 | -0.31 |
| PHF21A |  | 0.137 | 8.666 | 3.54 | 0.00E+00 | 1.40E-02 | -0.312 |
| STMN1 |  | -0.159 | 8.381 | -3.536 | 0.00E+00 | 1.40E-02 | -0.328 |
| MTIF3 |  | 0.138 | 9.772 | 3.534 | 0.00E+00 | 1.40E-02 | -0.332 |
| CEBPA |  | 0.22 | 10.356 | 3.53 | 0.00E+00 | 1.50E-02 | -0.345 |
| INHBA |  | 0.407 | 9.387 | 3.529 | 0.00E+00 | 1.50E-02 | -0.347 |
| CDKAL1 |  | -0.178 | 7.177 | -3.523 | 0.00E+00 | 1.50E-02 | -0.368 |
| CD200R1 |  | -0.215 | 5.759 | -3.518 | 0.00E+00 | 1.50E-02 | -0.384 |
| LMNA |  | 0.224 | 10.522 | 3.51 | 0.00E+00 | 1.50E-02 | -0.408 |
| ADAMTS1 |  | 0.242 | 9.488 | 3.508 | 0.00E+00 | 1.50E-02 | -0.414 |
| MAP2K1 |  | -0.156 | 10.106 | -3.508 | 0.00E+00 | 1.50E-02 | -0.417 |
| STK11 |  | 0.177 | 8.513 | 3.505 | 0.00E+00 | 1.60E-02 | -0.425 |
| EIF4G3 |  | 0.173 | 7.909 | 3.503 | 0.00E+00 | 1.60E-02 | -0.43 |
| TAF4 |  | -0.16 | 7.824 | -3.503 | 0.00E+00 | 1.60E-02 | -0.435 |
| DPP8 |  | -0.11 | 9.115 | -3.493 | 0.00E+00 | 1.60E-02 | -0.467 |
| ITGAE |  | 0.165 | 8.95 | 3.487 | 0.00E+00 | 1.60E-02 | -0.481 |
| LIN7B |  | 0.184 | 7.526 | 3.484 | 0.00E+00 | 1.60E-02 | -0.491 |
| RUNX2 |  | 0.144 | 5.822 | 3.481 | 0.00E+00 | 1.70E-02 | -0.502 |
| STK36 |  | 0.138 | 7.584 | 3.48 | 0.00E+00 | 1.70E-02 | -0.502 |
| ARFGAP1 |  | 0.156 | 9.13 | 3.479 | 0.00E+00 | 1.70E-02 | -0.506 |
| HK2 |  | 0.288 | 7.321 | 3.475 | 0.00E+00 | 1.70E-02 | -0.518 |
| GSTA3 |  | -0.25 | 7.781 | -3.474 | 0.00E+00 | 1.70E-02 | -0.526 |
| ROM1 |  | 0.282 | 6.981 | 3.472 | 0.00E+00 | 1.70E-02 | -0.529 |
| GPR1 |  | -0.293 | 6.057 | -3.468 | 0.00E+00 | 1.70E-02 | -0.545 |
| LAIR2 |  | 0.456 | 7.327 | 3.464 | 0.00E+00 | 1.70E-02 | -0.554 |
| DPEP3 |  | -0.224 | 7.736 | -3.455 | 0.00E+00 | 1.80E-02 | -0.587 |
| BFSP2 |  | 0.197 | 5.431 | 3.449 | 0.00E+00 | 1.80E-02 | -0.602 |
| HMG20B |  | 0.164 | 9.61 | 3.447 | 0.00E+00 | 1.80E-02 | -0.61 |
| MLX |  | 0.11 | 8.411 | 3.446 | 0.00E+00 | 1.80E-02 | -0.612 |
| GNG5 |  | -0.145 | 10.855 | -3.445 | 0.00E+00 | 1.80E-02 | -0.619 |
| TPR |  | 0.151 | 8.997 | 3.444 | 0.00E+00 | 1.80E-02 | -0.62 |
| SLC9A5 |  | 0.194 | 6.624 | 3.441 | 0.00E+00 | 1.80E-02 | -0.629 |
| SLC15A4 |  | -0.134 | 8.646 | -3.438 | 0.00E+00 | 1.80E-02 | -0.641 |
| MGST3 |  | 0.159 | 11.674 | 3.433 | 0.00E+00 | 1.80E-02 | -0.654 |
| NUP153 |  | -0.14 | 9.999 | -3.428 | 0.00E+00 | 1.90E-02 | -0.672 |
| PRKCE |  | -0.182 | 7.282 | -3.425 | 0.00E+00 | 1.90E-02 | -0.683 |
| OGG1 |  | 0.132 | 7.174 | 3.418 | 0.00E+00 | 1.90E-02 | -0.7 |
| SLC16A4 |  | -0.224 | 9.351 | -3.416 | 0.00E+00 | 1.90E-02 | -0.71 |
| PRKAR2B |  | -0.199 | 8.29 | -3.41 | 0.00E+00 | 2.00E-02 | -0.728 |
| SNRPD3 |  | 0.152 | 9.144 | 3.407 | 0.00E+00 | 2.00E-02 | -0.734 |
| EPS8L1 |  | 0.191 | 9.011 | 3.406 | 0.00E+00 | 2.00E-02 | -0.74 |
| SNAPC3 |  | -0.122 | 7.419 | -3.398 | 0.00E+00 | 2.00E-02 | -0.767 |
| TMEFF2 |  | 0.188 | 5.761 | 3.394 | 0.00E+00 | 2.00E-02 | -0.775 |
| CARHSP1 |  | 0.203 | 11.101 | 3.394 | 0.00E+00 | 2.00E-02 | -0.775 |
| NR1D2 |  | -0.203 | 7.294 | -3.393 | 0.00E+00 | 2.00E-02 | -0.781 |
| GRM5 |  | -0.159 | 5.16 | -3.393 | 0.00E+00 | 2.00E-02 | -0.781 |
| MGST2 |  | 0.173 | 9.661 | 3.39 | 0.00E+00 | 2.00E-02 | -0.787 |
| FHOD1 |  | 0.153 | 8.881 | 3.389 | 0.00E+00 | 2.00E-02 | -0.791 |
| HCN4 |  | -0.218 | 7.399 | -3.384 | 0.00E+00 | 2.10E-02 | -0.81 |
| ITPR3 |  | 0.189 | 9.005 | 3.382 | 0.00E+00 | 2.10E-02 | -0.812 |
| CATSPER1 |  | 0.238 | 6.619 | 3.382 | 0.00E+00 | 2.10E-02 | -0.813 |
| PVRL3 |  | -0.254 | 9.668 | -3.382 | 0.00E+00 | 2.10E-02 | -0.816 |
| SNAI2 |  | -0.244 | 7.252 | -3.382 | 0.00E+00 | 2.10E-02 | -0.816 |
| NCOR2 |  | 0.189 | 10.615 | 3.38 | 0.00E+00 | 2.10E-02 | -0.819 |
| GNPTG |  | 0.143 | 10.485 | 3.378 | 0.00E+00 | 2.10E-02 | -0.824 |
| NEDD4 |  | -0.157 | 7.817 | -3.379 | 0.00E+00 | 2.10E-02 | -0.825 |
| ZFP28 |  | -0.179 | 5.399 | -3.367 | 0.00E+00 | 2.10E-02 | -0.862 |
| RPL37 |  | 0.126 | 10.607 | 3.365 | 0.00E+00 | 2.10E-02 | -0.865 |
| MMP11 |  | 0.244 | 10.454 | 3.365 | 0.00E+00 | 2.10E-02 | -0.866 |
| IRAK2 |  | -0.267 | 6.452 | -3.354 | 0.00E+00 | 2.20E-02 | -0.904 |
| TM6SF2 |  | -0.194 | 6.205 | -3.352 | 0.00E+00 | 2.20E-02 | -0.91 |
| CAMP |  | -0.332 | 7.33 | -3.351 | 0.00E+00 | 2.20E-02 | -0.912 |
| SMPDL3A |  | -0.201 | 9.11 | -3.351 | 0.00E+00 | 2.20E-02 | -0.914 |
| BCAT1 |  | 0.195 | 7.774 | 3.345 | 0.00E+00 | 2.30E-02 | -0.928 |
| STOM |  | -0.289 | 10.417 | -3.341 | 0.00E+00 | 2.30E-02 | -0.944 |
| DUSP4 |  | -0.23 | 8.803 | -3.338 | 0.00E+00 | 2.30E-02 | -0.952 |
| UROC1 |  | -0.153 | 5.238 | -3.337 | 0.00E+00 | 2.30E-02 | -0.954 |
| GABRR1 |  | -0.228 | 5.257 | -3.337 | 0.00E+00 | 2.30E-02 | -0.955 |
| RPS23 |  | 0.178 | 12.19 | 3.329 | 0.00E+00 | 2.30E-02 | -0.977 |
| POLR3GL |  | 0.126 | 9.972 | 3.326 | 1.00E-03 | 2.40E-02 | -0.986 |
| TACC2 |  | -0.164 | 8.47 | -3.327 | 1.00E-03 | 2.40E-02 | -0.988 |
| NOXA1 |  | 0.201 | 7.214 | 3.324 | 1.00E-03 | 2.40E-02 | -0.992 |
| ZNF593 |  | 0.171 | 9.891 | 3.322 | 1.00E-03 | 2.40E-02 | -0.999 |
| VIT |  | 0.277 | 5.885 | 3.321 | 1.00E-03 | 2.40E-02 | -1.002 |
| FZD7 |  | -0.173 | 7.163 | -3.32 | 1.00E-03 | 2.40E-02 | -1.007 |
| PPT1 |  | 0.176 | 10.929 | 3.315 | 1.00E-03 | 2.40E-02 | -1.018 |
| TAF6 |  | 0.147 | 9.417 | 3.313 | 1.00E-03 | 2.40E-02 | -1.024 |
| GFRA3 |  | -0.204 | 6.412 | -3.312 | 1.00E-03 | 2.40E-02 | -1.031 |
| CCNDBP1 |  | -0.158 | 10.469 | -3.307 | 1.00E-03 | 2.40E-02 | -1.046 |
| GTPBP3 |  | 0.139 | 8.248 | 3.301 | 1.00E-03 | 2.50E-02 | -1.061 |
| STX10 |  | 0.173 | 9.158 | 3.301 | 1.00E-03 | 2.50E-02 | -1.064 |
| SLC38A5 |  | -0.201 | 6.94 | -3.298 | 1.00E-03 | 2.50E-02 | -1.075 |
| GPR87 |  | 0.204 | 6.498 | 3.295 | 1.00E-03 | 2.50E-02 | -1.08 |
| F5 |  | -0.187 | 9.188 | -3.295 | 1.00E-03 | 2.50E-02 | -1.084 |
| MAGEL2 |  | -0.176 | 7.173 | -3.286 | 1.00E-03 | 2.60E-02 | -1.11 |
| FXYD3 |  | -0.192 | 7.433 | -3.273 | 1.00E-03 | 2.70E-02 | -1.151 |
| SLC25A27 |  | -0.192 | 5.537 | -3.271 | 1.00E-03 | 2.70E-02 | -1.156 |
| PANX2 |  | -0.194 | 6.532 | -3.27 | 1.00E-03 | 2.70E-02 | -1.158 |
| DAB2 |  | -0.189 | 11.853 | -3.268 | 1.00E-03 | 2.70E-02 | -1.165 |
| HN1 |  | 0.254 | 10.638 | 3.266 | 1.00E-03 | 2.70E-02 | -1.168 |
| DNAJB9 |  | -0.176 | 10.886 | -3.266 | 1.00E-03 | 2.70E-02 | -1.172 |
| NTRK2 |  | 0.161 | 5.291 | 3.26 | 1.00E-03 | 2.80E-02 | -1.186 |
| HTRA3 |  | -0.282 | 7.023 | -3.252 | 1.00E-03 | 2.80E-02 | -1.213 |
| DUSP16 |  | -0.163 | 7.6 | -3.248 | 1.00E-03 | 2.90E-02 | -1.226 |
| KRTCAP3 |  | 0.171 | 6.831 | 3.245 | 1.00E-03 | 2.90E-02 | -1.231 |
| PDLIM1 |  | -0.188 | 10.402 | -3.241 | 1.00E-03 | 2.90E-02 | -1.246 |
| SF3A1 |  | -0.125 | 8.796 | -3.237 | 1.00E-03 | 2.90E-02 | -1.258 |
| DLC1 |  | 0.155 | 7.529 | 3.235 | 1.00E-03 | 2.90E-02 | -1.26 |
| CTBP1 |  | 0.147 | 9.499 | 3.235 | 1.00E-03 | 2.90E-02 | -1.263 |
| MKNK1 |  | -0.141 | 8.91 | -3.233 | 1.00E-03 | 3.00E-02 | -1.269 |
| HDLBP |  | 0.132 | 8.913 | 3.229 | 1.00E-03 | 3.00E-02 | -1.279 |
| KATNB1 |  | -0.128 | 7.764 | -3.226 | 1.00E-03 | 3.00E-02 | -1.29 |
| OCRL |  | 0.153 | 8.95 | 3.225 | 1.00E-03 | 3.00E-02 | -1.292 |
| CYSLTR1 |  | -0.177 | 6.237 | -3.22 | 1.00E-03 | 3.00E-02 | -1.308 |
| MYO9A |  | -0.172 | 9.309 | -3.217 | 1.00E-03 | 3.10E-02 | -1.319 |
| C1GALT1C1 |  | 0.153 | 8.649 | 3.215 | 1.00E-03 | 3.10E-02 | -1.321 |
| PITPNC1 |  | -0.135 | 6.956 | -3.216 | 1.00E-03 | 3.10E-02 | -1.322 |
| GNG13 |  | -0.253 | 6.788 | -3.215 | 1.00E-03 | 3.10E-02 | -1.323 |
| TLR8 |  | -0.174 | 6.869 | -3.214 | 1.00E-03 | 3.10E-02 | -1.326 |
| RPS6KC1 |  | -0.131 | 8.05 | -3.213 | 1.00E-03 | 3.10E-02 | -1.33 |
| CPVL |  | 0.217 | 9.366 | 3.212 | 1.00E-03 | 3.10E-02 | -1.331 |
| FOXJ2 |  | 0.137 | 9.073 | 3.207 | 1.00E-03 | 3.10E-02 | -1.346 |
| SQRDL |  | -0.176 | 9.91 | -3.207 | 1.00E-03 | 3.10E-02 | -1.348 |
| CPE |  | 0.282 | 6.676 | 3.206 | 1.00E-03 | 3.10E-02 | -1.349 |
| GREM2 |  | 0.257 | 7.055 | 3.204 | 1.00E-03 | 3.10E-02 | -1.354 |
| ACP1 |  | -0.11 | 8.831 | -3.203 | 1.00E-03 | 3.10E-02 | -1.36 |
| CD53 |  | -0.199 | 8.408 | -3.2 | 1.00E-03 | 3.10E-02 | -1.367 |
| DHX32 |  | -0.142 | 9.238 | -3.199 | 1.00E-03 | 3.10E-02 | -1.371 |
| GRINA |  | -0.242 | 9.958 | -3.198 | 1.00E-03 | 3.10E-02 | -1.373 |
| FBXO30 |  | -0.124 | 8.837 | -3.197 | 1.00E-03 | 3.10E-02 | -1.377 |
| PPP1R12C |  | 0.163 | 9.293 | 3.195 | 1.00E-03 | 3.10E-02 | -1.381 |
| SERPINB9 |  | 0.201 | 8.095 | 3.194 | 1.00E-03 | 3.10E-02 | -1.383 |
| FBXO9 |  | -0.123 | 9.245 | -3.194 | 1.00E-03 | 3.10E-02 | -1.387 |
| SIRT3 |  | 0.098 | 7.345 | 3.192 | 1.00E-03 | 3.10E-02 | -1.388 |
| MAFB |  | -0.19 | 9.414 | -3.181 | 1.00E-03 | 3.30E-02 | -1.425 |
| COLEC10 |  | -0.197 | 5.696 | -3.18 | 1.00E-03 | 3.30E-02 | -1.427 |
| MICAL3 |  | 0.145 | 6.285 | 3.179 | 1.00E-03 | 3.30E-02 | -1.429 |
| TCEAL1 |  | 0.152 | 8.347 | 3.178 | 1.00E-03 | 3.30E-02 | -1.432 |
| SPTLC1 |  | -0.211 | 8.667 | -3.177 | 1.00E-03 | 3.30E-02 | -1.438 |
| KIAA0753 |  | 0.1 | 8.198 | 3.172 | 1.00E-03 | 3.30E-02 | -1.448 |
| ZNF317 |  | -0.131 | 8.115 | -3.173 | 1.00E-03 | 3.30E-02 | -1.448 |
| GPX3 |  | 0.251 | 9.345 | 3.167 | 1.00E-03 | 3.30E-02 | -1.462 |
| HSD17B1 |  | -0.268 | 10.571 | -3.167 | 1.00E-03 | 3.30E-02 | -1.467 |
| KCTD11 |  | 0.163 | 7.576 | 3.165 | 1.00E-03 | 3.30E-02 | -1.468 |
| SLC6A19 |  | -0.176 | 5.968 | -3.165 | 1.00E-03 | 3.30E-02 | -1.471 |
| SLC25A29 |  | 0.165 | 8.056 | 3.163 | 1.00E-03 | 3.40E-02 | -1.475 |
| TAPBP |  | 0.175 | 8.415 | 3.159 | 1.00E-03 | 3.40E-02 | -1.487 |
| FHL2 |  | 0.197 | 10.2 | 3.157 | 1.00E-03 | 3.40E-02 | -1.492 |
| FN1 |  | 0.377 | 8.012 | 3.156 | 1.00E-03 | 3.40E-02 | -1.496 |
| RAB38 |  | -0.17 | 7.942 | -3.153 | 1.00E-03 | 3.40E-02 | -1.507 |
| EXOSC10 |  | -0.101 | 9.93 | -3.151 | 1.00E-03 | 3.50E-02 | -1.514 |
| SEC24B |  | -0.127 | 8.523 | -3.149 | 1.00E-03 | 3.50E-02 | -1.52 |
| BRMS1 |  | 0.123 | 9.327 | 3.144 | 1.00E-03 | 3.50E-02 | -1.531 |
| CHRM1 |  | -0.215 | 5.949 | -3.141 | 1.00E-03 | 3.50E-02 | -1.541 |
| SPATA2 |  | -0.152 | 6.958 | -3.137 | 1.00E-03 | 3.60E-02 | -1.553 |
| MT1F |  | 0.293 | 8.13 | 3.136 | 1.00E-03 | 3.60E-02 | -1.553 |
| CLDN23 |  | -0.235 | 7.987 | -3.134 | 1.00E-03 | 3.60E-02 | -1.561 |
| SCARB2 |  | -0.189 | 10.273 | -3.131 | 1.00E-03 | 3.60E-02 | -1.57 |
| ANKRD1 |  | 0.209 | 5.647 | 3.129 | 1.00E-03 | 3.60E-02 | -1.572 |
| KHDRBS1 |  | 0.117 | 10.945 | 3.126 | 1.00E-03 | 3.60E-02 | -1.582 |
| ATP6V1D |  | 0.13 | 10.157 | 3.124 | 1.00E-03 | 3.60E-02 | -1.588 |
| LIPG |  | -0.181 | 8.278 | -3.125 | 1.00E-03 | 3.60E-02 | -1.59 |
| FGF9 |  | 0.203 | 5.728 | 3.123 | 1.00E-03 | 3.60E-02 | -1.591 |
| WNT8B |  | 0.187 | 4.853 | 3.122 | 1.00E-03 | 3.60E-02 | -1.593 |
| LGR5 |  | -0.204 | 5.72 | -3.122 | 2.00E-03 | 3.60E-02 | -1.596 |
| GPR135 |  | 0.141 | 6.622 | 3.121 | 2.00E-03 | 3.60E-02 | -1.596 |
| SLC17A5 |  | -0.15 | 8.518 | -3.122 | 2.00E-03 | 3.60E-02 | -1.597 |
| CHRNA10 |  | 0.163 | 6.321 | 3.12 | 2.00E-03 | 3.60E-02 | -1.601 |
| PRSS23 |  | 0.221 | 8.85 | 3.12 | 2.00E-03 | 3.60E-02 | -1.601 |
| COG6 |  | 0.118 | 7.799 | 3.12 | 2.00E-03 | 3.60E-02 | -1.601 |
| AKR1B10 |  | -0.196 | 5.897 | -3.118 | 2.00E-03 | 3.60E-02 | -1.608 |
| DPF3 |  | 0.14 | 5.894 | 3.115 | 2.00E-03 | 3.60E-02 | -1.613 |
| NFIX |  | -0.2 | 7.688 | -3.116 | 2.00E-03 | 3.60E-02 | -1.614 |
| MAT2B |  | -0.128 | 9.918 | -3.116 | 2.00E-03 | 3.60E-02 | -1.615 |
| KATNAL1 |  | 0.148 | 7.287 | 3.112 | 2.00E-03 | 3.70E-02 | -1.622 |
| EP300 |  | -0.163 | 8.984 | -3.113 | 2.00E-03 | 3.70E-02 | -1.622 |
| ZNF418 |  | -0.193 | 7.61 | -3.112 | 2.00E-03 | 3.70E-02 | -1.625 |
| UBE2L6 |  | -0.169 | 9.561 | -3.111 | 2.00E-03 | 3.70E-02 | -1.63 |
| EIF2B2 |  | -0.109 | 10.214 | -3.109 | 2.00E-03 | 3.70E-02 | -1.634 |
| TPSD1 |  | 0.208 | 5.555 | 3.108 | 2.00E-03 | 3.70E-02 | -1.634 |
| ENPP1 |  | -0.216 | 8.144 | -3.108 | 2.00E-03 | 3.70E-02 | -1.636 |
| ZNF319 |  | -0.176 | 7.39 | -3.108 | 2.00E-03 | 3.70E-02 | -1.637 |
| SGPL1 |  | -0.124 | 7.64 | -3.107 | 2.00E-03 | 3.70E-02 | -1.64 |
| KCNE3 |  | -0.18 | 7.132 | -3.104 | 2.00E-03 | 3.70E-02 | -1.65 |
| MANSC1 |  | -0.177 | 8.108 | -3.099 | 2.00E-03 | 3.70E-02 | -1.665 |
| HSD11B1 |  | -0.332 | 8.126 | -3.098 | 2.00E-03 | 3.70E-02 | -1.667 |
| TPI1 |  | 0.131 | 12.156 | 3.096 | 2.00E-03 | 3.70E-02 | -1.668 |
| SURF2 |  | 0.21 | 8.126 | 3.093 | 2.00E-03 | 3.80E-02 | -1.677 |
| CLN6 |  | -0.121 | 7.76 | -3.093 | 2.00E-03 | 3.80E-02 | -1.682 |
| APOL1 |  | -0.17 | 8.402 | -3.086 | 2.00E-03 | 3.90E-02 | -1.7 |
| RASGRP4 |  | -0.153 | 6.63 | -3.082 | 2.00E-03 | 3.90E-02 | -1.711 |
| DLEU1 |  | 0.123 | 7.226 | 3.078 | 2.00E-03 | 3.90E-02 | -1.722 |
| ESRRG |  | -0.161 | 7.132 | -3.075 | 2.00E-03 | 4.00E-02 | -1.733 |
| GANAB |  | 0.121 | 10.205 | 3.074 | 2.00E-03 | 4.00E-02 | -1.733 |
| NCF2 |  | -0.178 | 8.695 | -3.072 | 2.00E-03 | 4.00E-02 | -1.74 |
| TAS1R1 |  | -0.197 | 6.248 | -3.071 | 2.00E-03 | 4.00E-02 | -1.743 |
| HINT3 |  | -0.15 | 8.04 | -3.069 | 2.00E-03 | 4.00E-02 | -1.749 |
| LAMA2 |  | -0.201 | 8.749 | -3.066 | 2.00E-03 | 4.00E-02 | -1.758 |
| C21orf58 |  | -0.198 | 7.324 | -3.065 | 2.00E-03 | 4.00E-02 | -1.759 |
| MLF1 |  | 0.18 | 6.053 | 3.062 | 2.00E-03 | 4.10E-02 | -1.767 |
| MAP3K10 |  | 0.149 | 7.177 | 3.061 | 2.00E-03 | 4.10E-02 | -1.769 |
| DNAJC13 |  | -0.143 | 9.429 | -3.062 | 2.00E-03 | 4.10E-02 | -1.77 |
| C1orf43 |  | -0.115 | 10.071 | -3.06 | 2.00E-03 | 4.10E-02 | -1.776 |
| GRK5 |  | -0.13 | 8.67 | -3.058 | 2.00E-03 | 4.10E-02 | -1.781 |
| RNASEL |  | -0.152 | 7.934 | -3.056 | 2.00E-03 | 4.10E-02 | -1.785 |
| MRPL23 |  | 0.131 | 10.106 | 3.053 | 2.00E-03 | 4.10E-02 | -1.791 |
| CDKL2 |  | 0.135 | 4.832 | 3.051 | 2.00E-03 | 4.10E-02 | -1.797 |
| KBTBD7 |  | 0.148 | 8.069 | 3.051 | 2.00E-03 | 4.10E-02 | -1.798 |
| KCMF1 |  | -0.116 | 8.419 | -3.049 | 2.00E-03 | 4.20E-02 | -1.806 |
| XAGE3 |  | 0.167 | 9.19 | 3.047 | 2.00E-03 | 4.20E-02 | -1.81 |
| GNPAT |  | -0.102 | 9.517 | -3.038 | 2.00E-03 | 4.30E-02 | -1.838 |
| RBM4 |  | -0.095 | 8.639 | -3.037 | 2.00E-03 | 4.30E-02 | -1.839 |
| CORO1C |  | 0.18 | 9.801 | 3.036 | 2.00E-03 | 4.30E-02 | -1.84 |
| CRYGA |  | -0.174 | 5.895 | -3.036 | 2.00E-03 | 4.30E-02 | -1.843 |
| GCN1L1 |  | 0.169 | 10.348 | 3.032 | 2.00E-03 | 4.30E-02 | -1.85 |
| SLC35B4 |  | 0.158 | 7.645 | 3.031 | 2.00E-03 | 4.30E-02 | -1.853 |
| CANT1 |  | -0.149 | 9.371 | -3.032 | 2.00E-03 | 4.30E-02 | -1.853 |
| BCAP31 |  | 0.119 | 11.604 | 3.029 | 2.00E-03 | 4.30E-02 | -1.859 |
| NAP1L4 |  | 0.131 | 9.902 | 3.029 | 2.00E-03 | 4.30E-02 | -1.86 |
| FNDC3A |  | -0.235 | 9.346 | -3.029 | 2.00E-03 | 4.30E-02 | -1.861 |
| CA4 |  | 0.248 | 7.609 | 3.028 | 2.00E-03 | 4.30E-02 | -1.862 |
| SORCS2 |  | -0.187 | 5.449 | -3.028 | 2.00E-03 | 4.30E-02 | -1.864 |
| H2AFY |  | -0.109 | 10.684 | -3.027 | 2.00E-03 | 4.30E-02 | -1.868 |
| LSM1 |  | 0.143 | 10.559 | 3.026 | 2.00E-03 | 4.30E-02 | -1.869 |
| THAP1 |  | -0.11 | 8.313 | -3.026 | 2.00E-03 | 4.30E-02 | -1.871 |
| MRPL43 |  | 0.117 | 8.364 | 3.024 | 2.00E-03 | 4.30E-02 | -1.873 |
| FOSB |  | 0.273 | 8.209 | 3.017 | 2.00E-03 | 4.40E-02 | -1.892 |
| SMUG1 |  | 0.125 | 8.898 | 3.016 | 2.00E-03 | 4.40E-02 | -1.897 |
| MAPRE1 |  | -0.124 | 10.802 | -3.016 | 2.00E-03 | 4.40E-02 | -1.898 |
| HLCS |  | 0.135 | 7.436 | 3.014 | 2.00E-03 | 4.40E-02 | -1.901 |
| DNAJB12 |  | -0.108 | 8.865 | -3.014 | 2.00E-03 | 4.40E-02 | -1.904 |
| RBM6 |  | 0.168 | 8.768 | 3.013 | 2.00E-03 | 4.40E-02 | -1.905 |
| BRWD3 |  | 0.151 | 7.152 | 3.012 | 2.00E-03 | 4.40E-02 | -1.908 |
| DOCK8 |  | -0.158 | 8.303 | -3.012 | 2.00E-03 | 4.40E-02 | -1.909 |
| ZNF573 |  | 0.139 | 8.432 | 3.011 | 2.00E-03 | 4.40E-02 | -1.91 |
| CDKL3 |  | 0.17 | 6.366 | 3.01 | 2.00E-03 | 4.40E-02 | -1.913 |
| PAM |  | 0.196 | 10.83 | 3.01 | 2.00E-03 | 4.40E-02 | -1.914 |
| SNAP23 |  | -0.226 | 8.777 | -3.01 | 2.00E-03 | 4.40E-02 | -1.914 |
| MOSPD1 |  | -0.204 | 7.928 | -3.01 | 2.00E-03 | 4.40E-02 | -1.915 |
| ACVR2B |  | -0.142 | 7.811 | -3.01 | 2.00E-03 | 4.40E-02 | -1.916 |
| NR2F1 |  | -0.213 | 8.633 | -3.009 | 2.00E-03 | 4.40E-02 | -1.918 |
| STK11IP |  | 0.193 | 8.399 | 3.007 | 2.00E-03 | 4.40E-02 | -1.92 |
| COTL1 |  | 0.176 | 9.724 | 3.004 | 2.00E-03 | 4.40E-02 | -1.93 |
| STK17A |  | -0.17 | 7.416 | -3.005 | 2.00E-03 | 4.40E-02 | -1.93 |
| PRDX5 |  | 0.11 | 12.334 | 3 | 2.00E-03 | 4.40E-02 | -1.94 |
| EPB41L2 |  | -0.15 | 8.209 | -3.001 | 2.00E-03 | 4.40E-02 | -1.941 |
| CMAS |  | -0.139 | 9.467 | -3.001 | 2.00E-03 | 4.40E-02 | -1.941 |
| CETP |  | -0.244 | 7.211 | -2.994 | 3.00E-03 | 4.50E-02 | -1.96 |
| HOXD12 |  | 0.116 | 5.129 | 2.992 | 3.00E-03 | 4.50E-02 | -1.962 |
| GADD45G |  | 0.3 | 9.511 | 2.987 | 3.00E-03 | 4.60E-02 | -1.975 |
| ZBED4 |  | -0.119 | 7.449 | -2.986 | 3.00E-03 | 4.60E-02 | -1.982 |
| SNX2 |  | 0.141 | 10.082 | 2.984 | 3.00E-03 | 4.60E-02 | -1.985 |
| RBM17 |  | 0.094 | 9.851 | 2.982 | 3.00E-03 | 4.60E-02 | -1.989 |
| PPIB |  | 0.141 | 11.432 | 2.98 | 3.00E-03 | 4.70E-02 | -1.997 |
| EGR3 |  | 0.244 | 5.353 | 2.978 | 3.00E-03 | 4.70E-02 | -2.001 |
| HCRTR1 |  | -0.181 | 6.909 | -2.978 | 3.00E-03 | 4.70E-02 | -2.004 |
| PSMC6 |  | -0.144 | 10.585 | -2.978 | 3.00E-03 | 4.70E-02 | -2.005 |
| CNFN |  | -0.136 | 7.948 | -2.977 | 3.00E-03 | 4.70E-02 | -2.008 |
| SDSL |  | -0.221 | 8.42 | -2.976 | 3.00E-03 | 4.70E-02 | -2.009 |
| NVL |  | 0.156 | 8.677 | 2.973 | 3.00E-03 | 4.70E-02 | -2.014 |
| CES2 |  | -0.107 | 8.464 | -2.973 | 3.00E-03 | 4.70E-02 | -2.019 |
| PLEK |  | -0.156 | 8.648 | -2.967 | 3.00E-03 | 4.80E-02 | -2.035 |
| PREX1 |  | 0.153 | 9.063 | 2.965 | 3.00E-03 | 4.80E-02 | -2.038 |
| KCNK3 |  | -0.192 | 7.414 | -2.965 | 3.00E-03 | 4.80E-02 | -2.039 |
| UPP1 |  | -0.165 | 8.493 | -2.964 | 3.00E-03 | 4.80E-02 | -2.042 |
| RASAL2 |  | -0.143 | 6.97 | -2.963 | 3.00E-03 | 4.80E-02 | -2.045 |
| GCH1 |  | -0.174 | 7.198 | -2.961 | 3.00E-03 | 4.80E-02 | -2.05 |
| C9orf72 |  | -0.168 | 6.965 | -2.961 | 3.00E-03 | 4.80E-02 | -2.051 |
| PELI1 |  | -0.163 | 8.827 | -2.961 | 3.00E-03 | 4.80E-02 | -2.051 |
| STK25 |  | 0.099 | 9.602 | 2.959 | 3.00E-03 | 4.80E-02 | -2.054 |
| IRX3 |  | -0.215 | 7.038 | -2.96 | 3.00E-03 | 4.80E-02 | -2.055 |
| PRDX1 |  | 0.103 | 13.052 | 2.956 | 3.00E-03 | 4.80E-02 | -2.062 |
| LRRC25 |  | -0.206 | 7.032 | -2.956 | 3.00E-03 | 4.80E-02 | -2.065 |
| IQCE |  | 0.117 | 7.161 | 2.955 | 3.00E-03 | 4.80E-02 | -2.065 |
| PHLDA3 |  | 0.157 | 8.164 | 2.954 | 3.00E-03 | 4.80E-02 | -2.068 |
| MYT1 |  | 0.155 | 4.976 | 2.953 | 3.00E-03 | 4.80E-02 | -2.07 |
| GABRA3 |  | -0.183 | 5.74 | -2.954 | 3.00E-03 | 4.80E-02 | -2.07 |
| SYK |  | -0.164 | 7.741 | -2.954 | 3.00E-03 | 4.80E-02 | -2.071 |
| SCGB1A1 |  | -0.266 | 6.522 | -2.952 | 3.00E-03 | 4.80E-02 | -2.076 |
| KLF10 |  | -0.135 | 9.208 | -2.952 | 3.00E-03 | 4.80E-02 | -2.077 |
| SYNE1 |  | 0.132 | 6.57 | 2.948 | 3.00E-03 | 4.90E-02 | -2.085 |
| XG |  | 0.227 | 6.12 | 2.947 | 3.00E-03 | 4.90E-02 | -2.086 |
| NUBP1 |  | -0.169 | 9.137 | -2.946 | 3.00E-03 | 4.90E-02 | -2.091 |
| KBTBD4 |  | -0.108 | 7.841 | -2.946 | 3.00E-03 | 4.90E-02 | -2.091 |
| RQCD1 |  | -0.144 | 7.623 | -2.946 | 3.00E-03 | 4.90E-02 | -2.091 |
| RASL12 |  | -0.191 | 7.118 | -2.946 | 3.00E-03 | 4.90E-02 | -2.093 |
| SLC2A3 |  | 0.209 | 9.122 | 2.941 | 3.00E-03 | 4.90E-02 | -2.105 |
| TBL2 |  | -0.103 | 10.127 | -2.941 | 3.00E-03 | 4.90E-02 | -2.107 |
| SIGIRR |  | 0.117 | 8.343 | 2.939 | 3.00E-03 | 4.90E-02 | -2.11 |
| TBC1D14 |  | -0.137 | 9.179 | -2.937 | 3.00E-03 | 5.00E-02 | -2.116 |
| DCTD |  | -0.114 | 9.156 | -2.936 | 3.00E-03 | 5.00E-02 | -2.119 |
| CDC23 |  | -0.119 | 9.058 | -2.936 | 3.00E-03 | 5.00E-02 | -2.12 |
| STK31 |  | -0.2 | 5.406 | -2.935 | 3.00E-03 | 5.00E-02 | -2.122 |
| BTG2 |  | -0.178 | 9.941 | -2.935 | 3.00E-03 | 5.00E-02 | -2.124 |
| MS4A1 |  | 0.149 | 6.088 | 2.934 | 3.00E-03 | 5.00E-02 | -2.124 |
| COQ6 |  | -0.137 | 7.706 | -2.932 | 3.00E-03 | 5.00E-02 | -2.13 |
| MRPS18A |  | 0.113 | 9.262 | 2.931 | 3.00E-03 | 5.00E-02 | -2.132 |
| BEX1 |  | -0.284 | 7.893 | -2.931 | 3.00E-03 | 5.00E-02 | -2.133 |
| GLRX |  | 0.175 | 12.404 | 2.929 | 3.00E-03 | 5.00E-02 | -2.137 |
| CSF1R |  | -0.169 | 11.188 | -2.929 | 3.00E-03 | 5.00E-02 | -2.138 |
| UMOD |  | -0.172 | 7.102 | -2.928 | 3.00E-03 | 5.00E-02 | -2.143 |
| MAP3K15 |  | -0.165 | 4.868 | -2.924 | 3.00E-03 | 5.10E-02 | -2.152 |
| HCRT |  | -0.158 | 8.031 | -2.923 | 3.00E-03 | 5.10E-02 | -2.155 |
| CRISPLD1 |  | -0.212 | 5.633 | -2.921 | 3.00E-03 | 5.10E-02 | -2.16 |
| CCS |  | 0.106 | 9 | 2.918 | 3.00E-03 | 5.10E-02 | -2.165 |
| TRPM8 |  | -0.122 | 4.591 | -2.919 | 3.00E-03 | 5.10E-02 | -2.167 |
| NUDT9 |  | 0.121 | 8.941 | 2.917 | 3.00E-03 | 5.10E-02 | -2.168 |
| PLAU |  | -0.205 | 10.608 | -2.916 | 3.00E-03 | 5.10E-02 | -2.175 |
| ATP11C |  | 0.117 | 6.922 | 2.914 | 3.00E-03 | 5.10E-02 | -2.176 |
| SCARA3 |  | 0.159 | 8.53 | 2.912 | 3.00E-03 | 5.10E-02 | -2.181 |
| NDN |  | -0.215 | 8.245 | -2.913 | 3.00E-03 | 5.10E-02 | -2.182 |
| GDAP1 |  | -0.163 | 5.513 | -2.912 | 3.00E-03 | 5.10E-02 | -2.184 |
| STARD7 |  | 0.096 | 10.194 | 2.908 | 3.00E-03 | 5.20E-02 | -2.194 |
| SLC7A7 |  | -0.147 | 8.734 | -2.908 | 3.00E-03 | 5.20E-02 | -2.196 |
| BRSK2 |  | 0.23 | 6.042 | 2.905 | 3.00E-03 | 5.20E-02 | -2.2 |
| UBL5 |  | 0.118 | 11.886 | 2.902 | 4.00E-03 | 5.20E-02 | -2.21 |
| TLE6 |  | 0.21 | 6.921 | 2.901 | 4.00E-03 | 5.20E-02 | -2.213 |
| NNAT |  | 0.245 | 8.688 | 2.9 | 4.00E-03 | 5.20E-02 | -2.214 |
| WWP2 |  | -0.128 | 8.315 | -2.901 | 4.00E-03 | 5.20E-02 | -2.214 |
| CBFA2T3 |  | -0.157 | 7.442 | -2.901 | 4.00E-03 | 5.20E-02 | -2.216 |
| CCT5 |  | -0.138 | 8.47 | -2.9 | 4.00E-03 | 5.20E-02 | -2.218 |
| AGA |  | 0.126 | 8.604 | 2.898 | 4.00E-03 | 5.20E-02 | -2.219 |
| GPR62 |  | -0.174 | 5.629 | -2.899 | 4.00E-03 | 5.20E-02 | -2.221 |
| C11orf24 |  | 0.105 | 9.324 | 2.897 | 4.00E-03 | 5.20E-02 | -2.222 |
| GPSM3 |  | -0.146 | 8.954 | -2.898 | 4.00E-03 | 5.20E-02 | -2.223 |
| ACPP |  | -0.178 | 7.025 | -2.897 | 4.00E-03 | 5.20E-02 | -2.225 |
| CLEC2D |  | 0.135 | 7.577 | 2.895 | 4.00E-03 | 5.20E-02 | -2.227 |
| PCDHB13 |  | -0.246 | 6.866 | -2.896 | 4.00E-03 | 5.20E-02 | -2.228 |
| TXN2 |  | 0.097 | 9.332 | 2.895 | 4.00E-03 | 5.20E-02 | -2.23 |
| CLEC1A |  | -0.167 | 8.366 | -2.893 | 4.00E-03 | 5.30E-02 | -2.235 |
| EPHA5 |  | 0.156 | 5.091 | 2.892 | 4.00E-03 | 5.30E-02 | -2.237 |
| B3GAT3 |  | 0.124 | 8.528 | 2.887 | 4.00E-03 | 5.30E-02 | -2.25 |
| DPPA2 |  | -0.151 | 5.541 | -2.881 | 4.00E-03 | 5.50E-02 | -2.27 |
| SH3TC1 |  | 0.138 | 7.492 | 2.879 | 4.00E-03 | 5.50E-02 | -2.272 |
| ZNF92 |  | -0.145 | 7.448 | -2.878 | 4.00E-03 | 5.50E-02 | -2.277 |
| TM4SF5 |  | -0.228 | 6.822 | -2.877 | 4.00E-03 | 5.50E-02 | -2.28 |
| NR3C2 |  | -0.149 | 6.785 | -2.874 | 4.00E-03 | 5.50E-02 | -2.287 |
| PIP5KL1 |  | 0.221 | 6.619 | 2.872 | 4.00E-03 | 5.50E-02 | -2.291 |
| CDYL2 |  | -0.144 | 7.216 | -2.872 | 4.00E-03 | 5.50E-02 | -2.293 |
| MS4A3 |  | -0.222 | 5.549 | -2.872 | 4.00E-03 | 5.50E-02 | -2.294 |
| NKX2-3 |  | -0.175 | 5.954 | -2.868 | 4.00E-03 | 5.60E-02 | -2.304 |
| CALM1 |  | 0.146 | 11.264 | 2.864 | 4.00E-03 | 5.60E-02 | -2.311 |
| MRPL48 |  | 0.135 | 9.039 | 2.864 | 4.00E-03 | 5.60E-02 | -2.312 |
| SERPINB1 |  | 0.133 | 10.322 | 2.864 | 4.00E-03 | 5.60E-02 | -2.312 |
| RNF44 |  | -0.123 | 9.534 | -2.864 | 4.00E-03 | 5.60E-02 | -2.314 |
| ALOXE3 |  | -0.209 | 6.308 | -2.86 | 4.00E-03 | 5.70E-02 | -2.326 |
| ASPSCR1 |  | 0.185 | 8.213 | 2.856 | 4.00E-03 | 5.70E-02 | -2.334 |
| MRPL52 |  | 0.116 | 9.031 | 2.852 | 4.00E-03 | 5.80E-02 | -2.345 |
| EVL |  | 0.138 | 9.98 | 2.848 | 4.00E-03 | 5.80E-02 | -2.354 |
| AMPD3 |  | -0.127 | 7.551 | -2.849 | 4.00E-03 | 5.80E-02 | -2.355 |
| SAMHD1 |  | -0.151 | 7.443 | -2.846 | 4.00E-03 | 5.90E-02 | -2.361 |
| WDR45 |  | -0.128 | 9.495 | -2.843 | 4.00E-03 | 5.90E-02 | -2.37 |
| TXK |  | -0.251 | 7.811 | -2.841 | 4.00E-03 | 5.90E-02 | -2.375 |
| ALPL |  | -0.178 | 6.371 | -2.841 | 4.00E-03 | 5.90E-02 | -2.376 |
| KCTD4 |  | -0.261 | 5.392 | -2.839 | 4.00E-03 | 6.00E-02 | -2.38 |
| BZRAP1 |  | 0.171 | 6.226 | 2.837 | 4.00E-03 | 6.00E-02 | -2.383 |
| LETMD1 |  | -0.1 | 8.891 | -2.835 | 4.00E-03 | 6.00E-02 | -2.39 |
| SH3BP5 |  | 0.159 | 8.79 | 2.833 | 4.00E-03 | 6.00E-02 | -2.393 |
| SEC61A1 |  | 0.112 | 10.593 | 2.832 | 4.00E-03 | 6.00E-02 | -2.396 |
| TRIP10 |  | 0.176 | 8.956 | 2.832 | 4.00E-03 | 6.00E-02 | -2.397 |
| MBTD1 |  | -0.147 | 6.949 | -2.832 | 5.00E-03 | 6.00E-02 | -2.4 |
| DNMT3A |  | -0.163 | 7.626 | -2.831 | 5.00E-03 | 6.00E-02 | -2.402 |
| KCNC4 |  | -0.159 | 5.871 | -2.831 | 5.00E-03 | 6.00E-02 | -2.403 |
| LHFPL2 |  | -0.144 | 8.719 | -2.83 | 5.00E-03 | 6.00E-02 | -2.404 |
| ANXA5 |  | -0.109 | 13.111 | -2.83 | 5.00E-03 | 6.00E-02 | -2.406 |
| MARCKSL1 |  | -0.165 | 9.837 | -2.829 | 5.00E-03 | 6.00E-02 | -2.406 |
| HOXA13 |  | -0.206 | 6.826 | -2.829 | 5.00E-03 | 6.00E-02 | -2.407 |
| FOXK2 |  | 0.096 | 7.485 | 2.828 | 5.00E-03 | 6.00E-02 | -2.408 |
| SLC38A1 |  | -0.168 | 11.113 | -2.829 | 5.00E-03 | 6.00E-02 | -2.408 |
| NRIP1 |  | 0.217 | 9.707 | 2.827 | 5.00E-03 | 6.00E-02 | -2.409 |
| KLHL3 |  | -0.163 | 7.59 | -2.827 | 5.00E-03 | 6.00E-02 | -2.413 |
| EMD |  | 0.185 | 9.343 | 2.824 | 5.00E-03 | 6.00E-02 | -2.417 |
| RING1 |  | 0.101 | 10.226 | 2.823 | 5.00E-03 | 6.10E-02 | -2.422 |
| ATP5O |  | 0.107 | 12.58 | 2.822 | 5.00E-03 | 6.10E-02 | -2.424 |
| TRIM21 |  | -0.169 | 8.065 | -2.821 | 5.00E-03 | 6.10E-02 | -2.429 |
| PCTP |  | -0.126 | 9.815 | -2.82 | 5.00E-03 | 6.10E-02 | -2.43 |
| ADAT1 |  | -0.114 | 7.714 | -2.819 | 5.00E-03 | 6.10E-02 | -2.433 |
| DLG3 |  | 0.132 | 7.507 | 2.816 | 5.00E-03 | 6.10E-02 | -2.438 |
| SERPINB12 |  | 0.172 | 4.883 | 2.815 | 5.00E-03 | 6.10E-02 | -2.442 |
| PARVB |  | -0.163 | 7.22 | -2.814 | 5.00E-03 | 6.20E-02 | -2.447 |
| SUPT3H |  | -0.141 | 6.358 | -2.813 | 5.00E-03 | 6.20E-02 | -2.449 |
| OSTF1 |  | -0.106 | 10.808 | -2.812 | 5.00E-03 | 6.20E-02 | -2.452 |
| ADCY3 |  | -0.156 | 11.013 | -2.81 | 5.00E-03 | 6.20E-02 | -2.456 |
| RAB17 |  | 0.195 | 6.639 | 2.809 | 5.00E-03 | 6.20E-02 | -2.457 |
| OR7D2 |  | 0.226 | 4.871 | 2.808 | 5.00E-03 | 6.20E-02 | -2.459 |
| HTATSF1 |  | 0.106 | 9.631 | 2.807 | 5.00E-03 | 6.20E-02 | -2.462 |
| ARRDC3 |  | 0.205 | 8.776 | 2.806 | 5.00E-03 | 6.20E-02 | -2.466 |
| GPC6 |  | 0.147 | 5.824 | 2.804 | 5.00E-03 | 6.20E-02 | -2.47 |
| JAG1 |  | -0.161 | 8.857 | -2.805 | 5.00E-03 | 6.20E-02 | -2.471 |
| STK17B |  | -0.142 | 7.671 | -2.799 | 5.00E-03 | 6.30E-02 | -2.486 |
| S100A3 |  | 0.256 | 6.891 | 2.798 | 5.00E-03 | 6.30E-02 | -2.486 |
| LAMA1 |  | -0.143 | 5.593 | -2.798 | 5.00E-03 | 6.30E-02 | -2.488 |
| RPS24 |  | 0.157 | 12.373 | 2.794 | 5.00E-03 | 6.40E-02 | -2.496 |
| CTSC |  | -0.152 | 9.354 | -2.792 | 5.00E-03 | 6.40E-02 | -2.505 |
| NDUFS6 |  | 0.116 | 10.196 | 2.79 | 5.00E-03 | 6.40E-02 | -2.507 |
| DEPDC1B |  | -0.252 | 9.124 | -2.791 | 5.00E-03 | 6.40E-02 | -2.508 |
| SNRK |  | -0.146 | 8.033 | -2.79 | 5.00E-03 | 6.40E-02 | -2.51 |
| TFF1 |  | -0.153 | 6.441 | -2.79 | 5.00E-03 | 6.40E-02 | -2.511 |
| CHD3 |  | 0.131 | 8.187 | 2.786 | 5.00E-03 | 6.50E-02 | -2.519 |
| APOM |  | 0.152 | 6.764 | 2.784 | 5.00E-03 | 6.50E-02 | -2.523 |
| ALAS1 |  | -0.103 | 8.736 | -2.784 | 5.00E-03 | 6.50E-02 | -2.524 |
| CLPP |  | 0.11 | 10.216 | 2.782 | 5.00E-03 | 6.50E-02 | -2.528 |
| KCTD1 |  | -0.128 | 7.909 | -2.779 | 5.00E-03 | 6.60E-02 | -2.539 |
| SLC4A5 |  | -0.127 | 6.02 | -2.776 | 5.00E-03 | 6.60E-02 | -2.545 |
| PTPRD |  | -0.195 | 6.648 | -2.774 | 5.00E-03 | 6.70E-02 | -2.552 |
| HYDIN |  | -0.124 | 4.774 | -2.773 | 5.00E-03 | 6.70E-02 | -2.553 |
| LAMC2 |  | 0.193 | 6.609 | 2.771 | 5.00E-03 | 6.70E-02 | -2.556 |
| SPIN3 |  | 0.121 | 6.134 | 2.77 | 6.00E-03 | 6.70E-02 | -2.558 |
| RPL41 |  | 0.113 | 12.742 | 2.77 | 6.00E-03 | 6.70E-02 | -2.559 |
| STARD13 |  | -0.131 | 7.028 | -2.768 | 6.00E-03 | 6.70E-02 | -2.566 |
| AGPAT3 |  | -0.156 | 7.839 | -2.768 | 6.00E-03 | 6.70E-02 | -2.567 |
| PIGN |  | -0.142 | 7.664 | -2.768 | 6.00E-03 | 6.70E-02 | -2.567 |
| PDE1A |  | 0.147 | 5.206 | 2.765 | 6.00E-03 | 6.70E-02 | -2.571 |
| ARHGEF3 |  | 0.13 | 8.989 | 2.763 | 6.00E-03 | 6.70E-02 | -2.576 |
| NTHL1 |  | 0.129 | 8.092 | 2.763 | 6.00E-03 | 6.70E-02 | -2.576 |
| ASMT |  | -0.171 | 5.966 | -2.764 | 6.00E-03 | 6.70E-02 | -2.577 |
| KCNH1 |  | -0.204 | 5.103 | -2.764 | 6.00E-03 | 6.70E-02 | -2.578 |
| RAB27A |  | -0.12 | 7.885 | -2.762 | 6.00E-03 | 6.80E-02 | -2.581 |
| SBF2 |  | -0.1 | 7.627 | -2.761 | 6.00E-03 | 6.80E-02 | -2.585 |
| TMEM41A |  | -0.103 | 7.529 | -2.759 | 6.00E-03 | 6.80E-02 | -2.589 |
| SVIL |  | -0.142 | 7.746 | -2.759 | 6.00E-03 | 6.80E-02 | -2.59 |
| TIFA |  | -0.167 | 6.695 | -2.759 | 6.00E-03 | 6.80E-02 | -2.59 |
| BASP1 |  | 0.139 | 13.168 | 2.758 | 6.00E-03 | 6.80E-02 | -2.591 |
| TIMP3 |  | 0.248 | 9.986 | 2.758 | 6.00E-03 | 6.80E-02 | -2.591 |
| RBM5 |  | 0.098 | 10.955 | 2.757 | 6.00E-03 | 6.80E-02 | -2.592 |
| NR2E3 |  | -0.142 | 4.862 | -2.754 | 6.00E-03 | 6.80E-02 | -2.604 |
| CD9 |  | 0.207 | 9.835 | 2.752 | 6.00E-03 | 6.90E-02 | -2.606 |
| SNRPB |  | -0.092 | 11.197 | -2.749 | 6.00E-03 | 6.90E-02 | -2.617 |
| LSM3 |  | 0.118 | 10.526 | 2.747 | 6.00E-03 | 6.90E-02 | -2.619 |
| ALG5 |  | 0.138 | 10.447 | 2.747 | 6.00E-03 | 6.90E-02 | -2.619 |
| TMPRSS6 |  | 0.167 | 5.793 | 2.747 | 6.00E-03 | 6.90E-02 | -2.619 |
| NFKBIB |  | -0.108 | 7.794 | -2.747 | 6.00E-03 | 6.90E-02 | -2.62 |
| CX3CL1 |  | -0.219 | 6.638 | -2.747 | 6.00E-03 | 6.90E-02 | -2.62 |
| MAP4K2 |  | 0.129 | 9.418 | 2.743 | 6.00E-03 | 7.00E-02 | -2.629 |
| SELE |  | -0.215 | 5.492 | -2.741 | 6.00E-03 | 7.00E-02 | -2.636 |
| TNNC2 |  | 0.173 | 6.299 | 2.74 | 6.00E-03 | 7.00E-02 | -2.636 |
| ZNF10 |  | -0.123 | 6.993 | -2.738 | 6.00E-03 | 7.00E-02 | -2.644 |
| BRI3BP |  | 0.135 | 7.092 | 2.737 | 6.00E-03 | 7.00E-02 | -2.644 |
| DSG1 |  | -0.156 | 4.962 | -2.736 | 6.00E-03 | 7.10E-02 | -2.648 |
| GRPEL1 |  | -0.103 | 8.973 | -2.735 | 6.00E-03 | 7.10E-02 | -2.65 |
| LMNB1 |  | 0.168 | 8.304 | 2.734 | 6.00E-03 | 7.10E-02 | -2.651 |
| SLC13A4 |  | -0.172 | 8.407 | -2.733 | 6.00E-03 | 7.10E-02 | -2.658 |
| EIF4EBP2 |  | 0.087 | 10.413 | 2.732 | 6.00E-03 | 7.10E-02 | -2.658 |
| FOSL1 |  | -0.159 | 6.957 | -2.731 | 6.00E-03 | 7.10E-02 | -2.661 |
| KDELR1 |  | 0.14 | 10.024 | 2.728 | 6.00E-03 | 7.10E-02 | -2.666 |
| SLC19A2 |  | -0.181 | 8.905 | -2.728 | 6.00E-03 | 7.20E-02 | -2.67 |
| RNASE4 |  | 0.18 | 7.403 | 2.726 | 6.00E-03 | 7.20E-02 | -2.671 |
| XDH |  | -0.257 | 6.08 | -2.726 | 6.00E-03 | 7.20E-02 | -2.676 |
| NEK7 |  | -0.243 | 7.712 | -2.724 | 6.00E-03 | 7.20E-02 | -2.679 |
| ADAMTS5 |  | 0.217 | 8.521 | 2.718 | 7.00E-03 | 7.30E-02 | -2.692 |
| PLAGL1 |  | -0.149 | 9.426 | -2.718 | 7.00E-03 | 7.30E-02 | -2.696 |
| PLAUR |  | -0.175 | 8.464 | -2.716 | 7.00E-03 | 7.30E-02 | -2.699 |
| YTHDF2 |  | 0.071 | 10.741 | 2.714 | 7.00E-03 | 7.40E-02 | -2.703 |
| COASY |  | -0.137 | 10.636 | -2.713 | 7.00E-03 | 7.40E-02 | -2.707 |
| ABCC3 |  | -0.196 | 7.637 | -2.712 | 7.00E-03 | 7.40E-02 | -2.709 |
| NPC2 |  | 0.124 | 12.848 | 2.711 | 7.00E-03 | 7.40E-02 | -2.71 |
| CABYR |  | 0.162 | 5.806 | 2.71 | 7.00E-03 | 7.40E-02 | -2.711 |
| SLC16A2 |  | -0.161 | 7.771 | -2.711 | 7.00E-03 | 7.40E-02 | -2.713 |
| FMNL3 |  | -0.141 | 5.978 | -2.709 | 7.00E-03 | 7.40E-02 | -2.717 |
| BTBD8 |  | 0.128 | 4.777 | 2.706 | 7.00E-03 | 7.50E-02 | -2.722 |
| TRIM10 |  | -0.157 | 6.454 | -2.705 | 7.00E-03 | 7.50E-02 | -2.727 |
| IL11RA |  | 0.106 | 8.409 | 2.704 | 7.00E-03 | 7.50E-02 | -2.729 |
| ZSCAN1 |  | -0.173 | 6.339 | -2.704 | 7.00E-03 | 7.50E-02 | -2.729 |
| ADSSL1 |  | 0.126 | 7.56 | 2.702 | 7.00E-03 | 7.50E-02 | -2.734 |
| SYMPK |  | 0.168 | 9.631 | 2.699 | 7.00E-03 | 7.60E-02 | -2.74 |
| USP10 |  | -0.104 | 9.171 | -2.699 | 7.00E-03 | 7.60E-02 | -2.742 |
| RIN1 |  | 0.132 | 8.159 | 2.698 | 7.00E-03 | 7.60E-02 | -2.743 |
| PLEKHB2 |  | -0.11 | 9.352 | -2.699 | 7.00E-03 | 7.60E-02 | -2.743 |
| CIRH1A |  | -0.117 | 9.299 | -2.696 | 7.00E-03 | 7.60E-02 | -2.75 |
| PRPF4 |  | -0.094 | 8.998 | -2.694 | 7.00E-03 | 7.60E-02 | -2.755 |
| HSBP1 |  | 0.088 | 11.179 | 2.692 | 7.00E-03 | 7.70E-02 | -2.759 |
| ARID1A |  | -0.105 | 8.791 | -2.691 | 7.00E-03 | 7.70E-02 | -2.762 |
| CDK9 |  | 0.103 | 9.129 | 2.688 | 7.00E-03 | 7.70E-02 | -2.768 |
| GBP4 |  | -0.239 | 8.774 | -2.688 | 7.00E-03 | 7.70E-02 | -2.772 |
| FANCA |  | -0.135 | 7.257 | -2.685 | 7.00E-03 | 7.80E-02 | -2.777 |
| SCGB1D2 |  | 0.204 | 4.92 | 2.683 | 7.00E-03 | 7.80E-02 | -2.78 |
| GALM |  | 0.142 | 7.602 | 2.679 | 7.00E-03 | 7.90E-02 | -2.79 |
| SEMA5A |  | 0.183 | 7.274 | 2.679 | 7.00E-03 | 7.90E-02 | -2.791 |
| CIAPIN1 |  | -0.087 | 8.993 | -2.679 | 7.00E-03 | 7.90E-02 | -2.793 |
| PCSK9 |  | 0.222 | 5.442 | 2.678 | 7.00E-03 | 7.90E-02 | -2.794 |
| EMP2 |  | -0.152 | 8.352 | -2.677 | 7.00E-03 | 7.90E-02 | -2.799 |
| MSRA |  | -0.127 | 8.917 | -2.675 | 7.00E-03 | 7.90E-02 | -2.803 |
| IL1RAPL1 |  | 0.175 | 4.958 | 2.674 | 7.00E-03 | 7.90E-02 | -2.803 |
| POLRMT |  | 0.115 | 8.882 | 2.673 | 7.00E-03 | 7.90E-02 | -2.807 |
| PSMB1 |  | 0.08 | 13.269 | 2.671 | 8.00E-03 | 8.00E-02 | -2.81 |
| KIF3A |  | -0.127 | 7.429 | -2.672 | 8.00E-03 | 8.00E-02 | -2.812 |
| MAN2B2 |  | 0.174 | 8.534 | 2.666 | 8.00E-03 | 8.10E-02 | -2.824 |
| PVR |  | 0.161 | 6.976 | 2.664 | 8.00E-03 | 8.10E-02 | -2.828 |
| SACS |  | 0.145 | 6.788 | 2.664 | 8.00E-03 | 8.10E-02 | -2.829 |
| RNF11 |  | -0.168 | 10.602 | -2.664 | 8.00E-03 | 8.10E-02 | -2.83 |
| DAB1 |  | -0.164 | 6.44 | -2.664 | 8.00E-03 | 8.10E-02 | -2.831 |
| CXCL14 |  | -0.229 | 8.565 | -2.663 | 8.00E-03 | 8.10E-02 | -2.833 |
| OLR1 |  | 0.225 | 8.982 | 2.661 | 8.00E-03 | 8.10E-02 | -2.835 |
| CYGB |  | -0.165 | 7.288 | -2.662 | 8.00E-03 | 8.10E-02 | -2.836 |
| C1orf50 |  | 0.091 | 9.233 | 2.661 | 8.00E-03 | 8.10E-02 | -2.837 |
| PRLR |  | -0.147 | 7.441 | -2.66 | 8.00E-03 | 8.10E-02 | -2.84 |
| BCL7A |  | -0.138 | 6.741 | -2.66 | 8.00E-03 | 8.10E-02 | -2.84 |
| PQLC2 |  | -0.116 | 7.806 | -2.66 | 8.00E-03 | 8.10E-02 | -2.84 |
| FGFR3 |  | 0.144 | 7.023 | 2.658 | 8.00E-03 | 8.10E-02 | -2.843 |
| KLK10 |  | -0.14 | 4.986 | -2.658 | 8.00E-03 | 8.10E-02 | -2.845 |
| LNX2 |  | -0.124 | 7.993 | -2.655 | 8.00E-03 | 8.20E-02 | -2.853 |
| SSB |  | 0.118 | 10.199 | 2.649 | 8.00E-03 | 8.30E-02 | -2.865 |
| ACVR1C |  | -0.153 | 5.242 | -2.649 | 8.00E-03 | 8.30E-02 | -2.867 |
| YPEL2 |  | -0.132 | 8.248 | -2.649 | 8.00E-03 | 8.30E-02 | -2.869 |
| MAPK8IP2 |  | -0.143 | 6.008 | -2.648 | 8.00E-03 | 8.30E-02 | -2.871 |
| SIAH1 |  | -0.132 | 9.235 | -2.647 | 8.00E-03 | 8.30E-02 | -2.873 |
| SH3GLB2 |  | 0.163 | 9.479 | 2.644 | 8.00E-03 | 8.40E-02 | -2.878 |
| ZNF30 |  | 0.143 | 6.259 | 2.643 | 8.00E-03 | 8.40E-02 | -2.881 |
| TLN1 |  | -0.147 | 9.078 | -2.643 | 8.00E-03 | 8.40E-02 | -2.882 |
| IDH1 |  | -0.134 | 10.194 | -2.643 | 8.00E-03 | 8.40E-02 | -2.884 |
| PPM1A |  | -0.119 | 8.096 | -2.642 | 8.00E-03 | 8.40E-02 | -2.886 |
| ABAT |  | -0.132 | 7.036 | -2.639 | 8.00E-03 | 8.40E-02 | -2.892 |
| TAF10 |  | 0.109 | 10.205 | 2.635 | 8.00E-03 | 8.50E-02 | -2.899 |
| TM4SF4 |  | -0.176 | 4.801 | -2.635 | 8.00E-03 | 8.50E-02 | -2.902 |
| DNM1L |  | -0.103 | 8.275 | -2.635 | 8.00E-03 | 8.50E-02 | -2.903 |
| MID2 |  | -0.114 | 6.733 | -2.632 | 9.00E-03 | 8.60E-02 | -2.91 |
| PNMA1 |  | -0.117 | 9.519 | -2.63 | 9.00E-03 | 8.60E-02 | -2.914 |
| MOG |  | -0.13 | 5.285 | -2.63 | 9.00E-03 | 8.60E-02 | -2.915 |
| LRRFIP2 |  | 0.088 | 8.766 | 2.629 | 9.00E-03 | 8.60E-02 | -2.916 |
| CA1 |  | -0.259 | 7.708 | -2.629 | 9.00E-03 | 8.60E-02 | -2.917 |
| TNFRSF25 |  | 0.139 | 8.619 | 2.628 | 9.00E-03 | 8.60E-02 | -2.917 |
| LY6E |  | 0.17 | 11.007 | 2.628 | 9.00E-03 | 8.60E-02 | -2.917 |
| CHCHD5 |  | 0.133 | 8.969 | 2.628 | 9.00E-03 | 8.60E-02 | -2.918 |
| VPS29 |  | 0.1 | 10.558 | 2.628 | 9.00E-03 | 8.60E-02 | -2.918 |
| JDP2 |  | 0.164 | 8.466 | 2.625 | 9.00E-03 | 8.60E-02 | -2.925 |
| HFE |  | 0.106 | 6.345 | 2.62 | 9.00E-03 | 8.70E-02 | -2.937 |
| SPACA1 |  | -0.184 | 5.3 | -2.619 | 9.00E-03 | 8.80E-02 | -2.942 |
| AP4E1 |  | 0.11 | 7.268 | 2.613 | 9.00E-03 | 8.90E-02 | -2.955 |
| CCDC3 |  | -0.255 | 8.307 | -2.612 | 9.00E-03 | 8.90E-02 | -2.959 |
| UBE2I |  | -0.107 | 9.226 | -2.611 | 9.00E-03 | 8.90E-02 | -2.961 |
| MRPS5 |  | 0.089 | 9.428 | 2.61 | 9.00E-03 | 8.90E-02 | -2.963 |
| GTF3C3 |  | -0.086 | 8.463 | -2.604 | 9.00E-03 | 9.10E-02 | -2.978 |
| CDCA4 |  | -0.149 | 7.455 | -2.602 | 9.00E-03 | 9.10E-02 | -2.983 |
| MYCN |  | 0.195 | 7.47 | 2.596 | 9.00E-03 | 9.20E-02 | -2.996 |
| PERP |  | 0.186 | 9.893 | 2.595 | 9.00E-03 | 9.20E-02 | -2.999 |
| PSMA8 |  | 0.17 | 4.999 | 2.595 | 1.00E-02 | 9.20E-02 | -2.999 |
| SEMA3D |  | 0.175 | 5.385 | 2.594 | 1.00E-02 | 9.20E-02 | -3 |
| RTN4RL1 |  | -0.13 | 6.307 | -2.592 | 1.00E-02 | 9.30E-02 | -3.008 |
| FOXE3 |  | -0.148 | 6.55 | -2.59 | 1.00E-02 | 9.40E-02 | -3.013 |
| BAIAP2L1 |  | 0.116 | 7.149 | 2.588 | 1.00E-02 | 9.40E-02 | -3.015 |
| MTRR |  | -0.129 | 7.471 | -2.589 | 1.00E-02 | 9.40E-02 | -3.016 |
| NT5C |  | 0.109 | 9.989 | 2.587 | 1.00E-02 | 9.40E-02 | -3.017 |
| ENDOG |  | 0.172 | 8.841 | 2.587 | 1.00E-02 | 9.40E-02 | -3.019 |
| MTX1 |  | 0.098 | 9.556 | 2.587 | 1.00E-02 | 9.40E-02 | -3.019 |
| C20orf144 |  | -0.137 | 5.96 | -2.585 | 1.00E-02 | 9.40E-02 | -3.026 |
| PAFAH1B3 |  | 0.139 | 8.399 | 2.583 | 1.00E-02 | 9.40E-02 | -3.027 |
| RUVBL2 |  | 0.101 | 10.052 | 2.583 | 1.00E-02 | 9.40E-02 | -3.028 |
| RARA |  | -0.128 | 8.432 | -2.583 | 1.00E-02 | 9.40E-02 | -3.029 |
| MRPS9 |  | 0.12 | 9.114 | 2.582 | 1.00E-02 | 9.40E-02 | -3.03 |
| NDUFB2 |  | 0.117 | 12.29 | 2.581 | 1.00E-02 | 9.40E-02 | -3.031 |
| P2RY1 |  | -0.201 | 6.702 | -2.581 | 1.00E-02 | 9.40E-02 | -3.034 |
| TRIO |  | 0.127 | 7.995 | 2.58 | 1.00E-02 | 9.50E-02 | -3.035 |
| ADM |  | -0.175 | 13.615 | -2.58 | 1.00E-02 | 9.50E-02 | -3.037 |
| POFUT2 |  | 0.101 | 7.539 | 2.578 | 1.00E-02 | 9.50E-02 | -3.04 |
| BCL2L13 |  | -0.081 | 8.668 | -2.576 | 1.00E-02 | 9.50E-02 | -3.046 |
| RECQL4 |  | 0.141 | 7.365 | 2.575 | 1.00E-02 | 9.50E-02 | -3.047 |
| ZNF608 |  | 0.176 | 6.144 | 2.575 | 1.00E-02 | 9.50E-02 | -3.047 |
| LRP1 |  | 0.158 | 7.742 | 2.575 | 1.00E-02 | 9.50E-02 | -3.048 |
| VKORC1 |  | 0.107 | 9.619 | 2.573 | 1.00E-02 | 9.60E-02 | -3.052 |
| TGFBR2 |  | -0.121 | 8.637 | -2.571 | 1.00E-02 | 9.60E-02 | -3.059 |
| TMEM14B |  | 0.132 | 11.06 | 2.568 | 1.00E-02 | 9.60E-02 | -3.063 |
| MPP6 |  | 0.156 | 6.543 | 2.567 | 1.00E-02 | 9.60E-02 | -3.065 |
| ANKRD35 |  | 0.166 | 7.201 | 2.567 | 1.00E-02 | 9.60E-02 | -3.066 |
| SPI1 |  | -0.126 | 8.256 | -2.568 | 1.00E-02 | 9.60E-02 | -3.066 |
| NT5C1A |  | 0.133 | 5.169 | 2.567 | 1.00E-02 | 9.60E-02 | -3.067 |
| ARPC3 |  | 0.105 | 12.32 | 2.565 | 1.00E-02 | 9.70E-02 | -3.072 |
| NPM3 |  | 0.136 | 8.341 | 2.563 | 1.00E-02 | 9.70E-02 | -3.075 |
| IFNK |  | 0.142 | 4.829 | 2.563 | 1.00E-02 | 9.70E-02 | -3.075 |
| MARCO |  | 0.213 | 7.044 | 2.563 | 1.00E-02 | 9.70E-02 | -3.076 |
| PHYHIPL |  | -0.185 | 7.399 | -2.563 | 1.00E-02 | 9.70E-02 | -3.079 |
| CNOT4 |  | -0.076 | 7.184 | -2.561 | 1.10E-02 | 9.70E-02 | -3.082 |
| MAPK1 |  | -0.088 | 8.714 | -2.559 | 1.10E-02 | 9.80E-02 | -3.088 |
| PPP2R2D |  | -0.088 | 8.189 | -2.558 | 1.10E-02 | 9.80E-02 | -3.089 |
| NUP160 |  | -0.117 | 8.938 | -2.558 | 1.10E-02 | 9.80E-02 | -3.091 |
| GCNT1 |  | 0.144 | 7.351 | 2.556 | 1.10E-02 | 9.80E-02 | -3.093 |
| SUSD2 |  | 0.15 | 7.234 | 2.554 | 1.10E-02 | 9.80E-02 | -3.098 |
| GTF2H5 |  | 0.111 | 9.558 | 2.554 | 1.10E-02 | 9.80E-02 | -3.098 |
| RPL7 |  | 0.188 | 12.233 | 2.553 | 1.10E-02 | 9.80E-02 | -3.099 |
| GDAP2 |  | -0.08 | 7.37 | -2.554 | 1.10E-02 | 9.80E-02 | -3.099 |
| SIX5 |  | 0.122 | 9.138 | 2.553 | 1.10E-02 | 9.80E-02 | -3.099 |
| MRPL28 |  | 0.179 | 9.385 | 2.553 | 1.10E-02 | 9.80E-02 | -3.1 |
| SOD3 |  | 0.218 | 7.935 | 2.551 | 1.10E-02 | 9.90E-02 | -3.105 |
| CSAD |  | 0.16 | 6.863 | 2.55 | 1.10E-02 | 9.90E-02 | -3.107 |
| HCFC2 |  | 0.11 | 7.842 | 2.55 | 1.10E-02 | 9.90E-02 | -3.107 |
| WDR12 |  | -0.093 | 8.913 | -2.548 | 1.10E-02 | 9.90E-02 | -3.115 |
| PRM3 |  | -0.135 | 6.19 | -2.546 | 1.10E-02 | 1.00E-01 | -3.12 |
| ADRB3 |  | -0.187 | 6.662 | -2.545 | 1.10E-02 | 1.00E-01 | -3.12 |
| POU3F4 |  | -0.169 | 5.577 | -2.545 | 1.10E-02 | 1.00E-01 | -3.121 |
| PRKCDBP |  | -0.178 | 10.648 | -2.542 | 1.10E-02 | 1.00E-01 | -3.129 |
| RIPK4 |  | 0.123 | 6.909 | 2.541 | 1.10E-02 | 1.00E-01 | -3.129 |
| VASP |  | 0.159 | 9.668 | 2.54 | 1.10E-02 | 1.01E-01 | -3.131 |
| CENPE |  | 0.153 | 7.359 | 2.539 | 1.10E-02 | 1.01E-01 | -3.132 |
| HRC |  | -0.189 | 6.409 | -2.539 | 1.10E-02 | 1.01E-01 | -3.136 |
| TCEA3 |  | 0.159 | 8.06 | 2.537 | 1.10E-02 | 1.01E-01 | -3.138 |
| IL1B |  | -0.385 | 8.391 | -2.537 | 1.10E-02 | 1.01E-01 | -3.141 |
| INADL |  | 0.142 | 6.008 | 2.535 | 1.10E-02 | 1.01E-01 | -3.142 |
| NUDCD3 |  | 0.093 | 8.073 | 2.534 | 1.10E-02 | 1.01E-01 | -3.145 |
| CYBB |  | -0.138 | 8.663 | -2.535 | 1.10E-02 | 1.01E-01 | -3.145 |
| SYN2 |  | -0.1 | 6.083 | -2.534 | 1.10E-02 | 1.01E-01 | -3.146 |
| DMBX1 |  | -0.159 | 5 | -2.534 | 1.10E-02 | 1.01E-01 | -3.147 |
| PDAP1 |  | 0.1 | 7.957 | 2.532 | 1.10E-02 | 1.02E-01 | -3.15 |
| DLX4 |  | -0.156 | 7.423 | -2.532 | 1.10E-02 | 1.02E-01 | -3.152 |
| PLEKHF1 |  | -0.11 | 9.237 | -2.53 | 1.20E-02 | 1.02E-01 | -3.158 |
| ZNF521 |  | -0.152 | 7.252 | -2.528 | 1.20E-02 | 1.03E-01 | -3.162 |
| AURKC |  | -0.172 | 5.623 | -2.528 | 1.20E-02 | 1.03E-01 | -3.162 |
| GPX4 |  | 0.097 | 11.876 | 2.525 | 1.20E-02 | 1.03E-01 | -3.167 |
| RNF111 |  | -0.079 | 8.524 | -2.524 | 1.20E-02 | 1.03E-01 | -3.17 |
| ICAM5 |  | 0.163 | 6.01 | 2.522 | 1.20E-02 | 1.03E-01 | -3.173 |
| MED8 |  | -0.083 | 8.961 | -2.523 | 1.20E-02 | 1.03E-01 | -3.173 |
| SLC25A22 |  | 0.124 | 8.409 | 2.522 | 1.20E-02 | 1.03E-01 | -3.174 |
| HMGA1 |  | -0.129 | 9.151 | -2.521 | 1.20E-02 | 1.04E-01 | -3.178 |
| ETF1 |  | -0.104 | 10.214 | -2.519 | 1.20E-02 | 1.04E-01 | -3.184 |
| ATM |  | 0.122 | 6.202 | 2.517 | 1.20E-02 | 1.04E-01 | -3.186 |
| IRF8 |  | -0.14 | 7.185 | -2.518 | 1.20E-02 | 1.04E-01 | -3.187 |
| FKBP10 |  | 0.167 | 8.179 | 2.515 | 1.20E-02 | 1.05E-01 | -3.19 |
| KLF11 |  | -0.118 | 7.524 | -2.515 | 1.20E-02 | 1.05E-01 | -3.192 |
| OR12D2 |  | -0.138 | 4.898 | -2.514 | 1.20E-02 | 1.05E-01 | -3.194 |
| MGAT2 |  | -0.108 | 8.846 | -2.512 | 1.20E-02 | 1.05E-01 | -3.2 |
| ATP2B3 |  | -0.111 | 5.413 | -2.512 | 1.20E-02 | 1.05E-01 | -3.2 |
| APLP2 |  | 0.109 | 10.325 | 2.507 | 1.20E-02 | 1.06E-01 | -3.209 |
| CLC |  | 0.277 | 7.011 | 2.507 | 1.20E-02 | 1.06E-01 | -3.21 |
| COX4I2 |  | -0.166 | 8.497 | -2.507 | 1.20E-02 | 1.06E-01 | -3.211 |
| MAPRE3 |  | -0.171 | 6.722 | -2.505 | 1.20E-02 | 1.07E-01 | -3.217 |
| ZNF175 |  | 0.158 | 7.199 | 2.502 | 1.20E-02 | 1.07E-01 | -3.22 |
| MRRF |  | -0.09 | 7.734 | -2.503 | 1.20E-02 | 1.07E-01 | -3.22 |
| FZD4 |  | -0.121 | 8.41 | -2.503 | 1.20E-02 | 1.07E-01 | -3.221 |
| GSTM2 |  | 0.141 | 6.783 | 2.498 | 1.30E-02 | 1.08E-01 | -3.229 |
| MUC13 |  | 0.122 | 4.862 | 2.498 | 1.30E-02 | 1.08E-01 | -3.231 |
| DNMT1 |  | -0.123 | 10.603 | -2.498 | 1.30E-02 | 1.08E-01 | -3.232 |
| GNAZ |  | 0.122 | 6.795 | 2.497 | 1.30E-02 | 1.08E-01 | -3.233 |
| DPYD |  | -0.155 | 7.351 | -2.497 | 1.30E-02 | 1.08E-01 | -3.236 |
| LAMC3 |  | -0.193 | 9.145 | -2.496 | 1.30E-02 | 1.08E-01 | -3.237 |
| PPP1R8 |  | -0.08 | 8.27 | -2.496 | 1.30E-02 | 1.08E-01 | -3.237 |
| GMNN |  | 0.133 | 9.211 | 2.495 | 1.30E-02 | 1.08E-01 | -3.238 |
| INPP5B |  | 0.129 | 7.132 | 2.493 | 1.30E-02 | 1.08E-01 | -3.241 |
| CHPT1 |  | -0.162 | 9.178 | -2.494 | 1.30E-02 | 1.08E-01 | -3.242 |
| HYAL2 |  | 0.16 | 9.561 | 2.492 | 1.30E-02 | 1.08E-01 | -3.244 |
| ZNF540 |  | 0.138 | 5.996 | 2.492 | 1.30E-02 | 1.08E-01 | -3.245 |
| C1orf21 |  | 0.146 | 7.255 | 2.492 | 1.30E-02 | 1.08E-01 | -3.245 |
| LDLR |  | 0.15 | 10.114 | 2.491 | 1.30E-02 | 1.08E-01 | -3.245 |
| CLOCK |  | -0.109 | 7.884 | -2.491 | 1.30E-02 | 1.09E-01 | -3.249 |
| KLHL21 |  | -0.106 | 8.698 | -2.49 | 1.30E-02 | 1.09E-01 | -3.251 |
| SNX3 |  | -0.107 | 10.883 | -2.489 | 1.30E-02 | 1.09E-01 | -3.253 |
| ABHD6 |  | 0.103 | 7.596 | 2.488 | 1.30E-02 | 1.09E-01 | -3.254 |
| GTDC1 |  | -0.074 | 6.772 | -2.488 | 1.30E-02 | 1.09E-01 | -3.256 |
| APOA1BP |  | 0.096 | 10.003 | 2.486 | 1.30E-02 | 1.09E-01 | -3.257 |
| RAC2 |  | -0.123 | 9.457 | -2.486 | 1.30E-02 | 1.09E-01 | -3.26 |
| CD4 |  | -0.178 | 8.038 | -2.485 | 1.30E-02 | 1.10E-01 | -3.262 |
| RCHY1 |  | -0.107 | 7.888 | -2.484 | 1.30E-02 | 1.10E-01 | -3.265 |
| DPP7 |  | 0.116 | 9.069 | 2.48 | 1.30E-02 | 1.11E-01 | -3.272 |
| BNIPL |  | 0.152 | 5.601 | 2.478 | 1.30E-02 | 1.11E-01 | -3.277 |
| H2AFV |  | 0.084 | 9.017 | 2.477 | 1.30E-02 | 1.11E-01 | -3.28 |
| UTP14A |  | -0.145 | 7.561 | -2.476 | 1.30E-02 | 1.12E-01 | -3.284 |
| TCL1A |  | 0.167 | 7.048 | 2.475 | 1.30E-02 | 1.12E-01 | -3.284 |
| DPF1 |  | -0.115 | 5.71 | -2.475 | 1.30E-02 | 1.12E-01 | -3.286 |
| E2F7 |  | 0.159 | 6.384 | 2.474 | 1.30E-02 | 1.12E-01 | -3.286 |
| IPO13 |  | -0.104 | 7.468 | -2.474 | 1.40E-02 | 1.12E-01 | -3.289 |
| TNC |  | -0.253 | 6.65 | -2.473 | 1.40E-02 | 1.12E-01 | -3.292 |
| PRSS8 |  | 0.174 | 9.743 | 2.471 | 1.40E-02 | 1.12E-01 | -3.294 |
| ANGPTL6 |  | -0.169 | 6.752 | -2.471 | 1.40E-02 | 1.12E-01 | -3.295 |
| SIDT2 |  | 0.107 | 10.105 | 2.47 | 1.40E-02 | 1.12E-01 | -3.296 |
| MGAT5B |  | 0.114 | 5.667 | 2.465 | 1.40E-02 | 1.14E-01 | -3.307 |
| PCBP4 |  | 0.136 | 7.832 | 2.463 | 1.40E-02 | 1.14E-01 | -3.311 |
| MUSK |  | -0.131 | 6.183 | -2.463 | 1.40E-02 | 1.14E-01 | -3.314 |
| HIBCH |  | 0.116 | 8.922 | 2.462 | 1.40E-02 | 1.14E-01 | -3.315 |
| ANLN |  | 0.227 | 7.504 | 2.461 | 1.40E-02 | 1.14E-01 | -3.316 |
| DNAH5 |  | 0.117 | 4.994 | 2.461 | 1.40E-02 | 1.14E-01 | -3.316 |
| ZBTB5 |  | -0.089 | 8.715 | -2.461 | 1.40E-02 | 1.14E-01 | -3.319 |
| TMED1 |  | 0.107 | 9.269 | 2.459 | 1.40E-02 | 1.14E-01 | -3.321 |
| TTC13 |  | 0.1 | 7.957 | 2.459 | 1.40E-02 | 1.14E-01 | -3.321 |
| GALNT2 |  | 0.139 | 8.194 | 2.454 | 1.40E-02 | 1.16E-01 | -3.332 |
| GUCY1A3 |  | -0.143 | 7.88 | -2.454 | 1.40E-02 | 1.16E-01 | -3.335 |
| ZFHX4 |  | 0.145 | 5.054 | 2.453 | 1.40E-02 | 1.16E-01 | -3.335 |
| CABP5 |  | -0.17 | 6.852 | -2.453 | 1.40E-02 | 1.16E-01 | -3.336 |
| AKT2 |  | 0.126 | 7.176 | 2.452 | 1.40E-02 | 1.16E-01 | -3.337 |
| STS |  | 0.16 | 8.323 | 2.451 | 1.40E-02 | 1.16E-01 | -3.339 |
| CACNA1C |  | 0.101 | 6.661 | 2.451 | 1.40E-02 | 1.16E-01 | -3.34 |
| NETO1 |  | 0.093 | 5.134 | 2.451 | 1.40E-02 | 1.16E-01 | -3.34 |
| ABCD3 |  | -0.128 | 7.504 | -2.45 | 1.40E-02 | 1.16E-01 | -3.344 |
| CLEC4D |  | -0.179 | 6.374 | -2.45 | 1.40E-02 | 1.16E-01 | -3.344 |
| CXCL16 |  | -0.133 | 7.712 | -2.449 | 1.50E-02 | 1.16E-01 | -3.346 |
| NLN |  | -0.131 | 6.793 | -2.446 | 1.50E-02 | 1.17E-01 | -3.353 |
| MYOD1 |  | -0.169 | 6.651 | -2.446 | 1.50E-02 | 1.17E-01 | -3.353 |
| FBXO2 |  | 0.22 | 6.739 | 2.444 | 1.50E-02 | 1.17E-01 | -3.356 |
| HCK |  | -0.13 | 8.273 | -2.445 | 1.50E-02 | 1.17E-01 | -3.356 |
| CKMT2 |  | 0.152 | 5.088 | 2.442 | 1.50E-02 | 1.18E-01 | -3.359 |
| CHRM2 |  | 0.127 | 5.066 | 2.442 | 1.50E-02 | 1.18E-01 | -3.36 |
| FUT9 |  | 0.093 | 4.901 | 2.44 | 1.50E-02 | 1.18E-01 | -3.366 |
| GGH |  | 0.184 | 9.794 | 2.439 | 1.50E-02 | 1.18E-01 | -3.367 |
| PCDHB4 |  | -0.152 | 5.689 | -2.439 | 1.50E-02 | 1.18E-01 | -3.369 |
| DSCR8 |  | 0.137 | 7.83 | 2.438 | 1.50E-02 | 1.18E-01 | -3.37 |
| ZDHHC3 |  | 0.067 | 9.073 | 2.436 | 1.50E-02 | 1.19E-01 | -3.374 |
| ADIPOR1 |  | -0.111 | 11.018 | -2.436 | 1.50E-02 | 1.19E-01 | -3.376 |
| GJC1 |  | -0.119 | 7.875 | -2.434 | 1.50E-02 | 1.19E-01 | -3.38 |
| PAX7 |  | -0.125 | 5.967 | -2.434 | 1.50E-02 | 1.19E-01 | -3.38 |
| BACH1 |  | 0.107 | 6.974 | 2.43 | 1.50E-02 | 1.20E-01 | -3.388 |
| MPG |  | 0.1 | 8.629 | 2.427 | 1.50E-02 | 1.21E-01 | -3.394 |
| CAPG |  | 0.173 | 9.113 | 2.427 | 1.50E-02 | 1.21E-01 | -3.395 |
| NEUROD4 |  | 0.122 | 4.757 | 2.425 | 1.50E-02 | 1.21E-01 | -3.399 |
| C3orf14 |  | -0.131 | 7.155 | -2.425 | 1.60E-02 | 1.21E-01 | -3.401 |
| DNPEP |  | -0.102 | 8.739 | -2.425 | 1.60E-02 | 1.21E-01 | -3.402 |
| NIPBL |  | 0.12 | 8.488 | 2.424 | 1.60E-02 | 1.21E-01 | -3.402 |
| SEMA6B |  | -0.147 | 6.477 | -2.424 | 1.60E-02 | 1.21E-01 | -3.403 |
| PQBP1 |  | 0.067 | 8.601 | 2.423 | 1.60E-02 | 1.21E-01 | -3.404 |
| LHX2 |  | 0.169 | 6.556 | 2.422 | 1.60E-02 | 1.21E-01 | -3.405 |
| PRKCH |  | -0.1 | 9.33 | -2.423 | 1.60E-02 | 1.21E-01 | -3.406 |
| PRRX1 |  | -0.204 | 6.151 | -2.423 | 1.60E-02 | 1.21E-01 | -3.406 |
| NPDC1 |  | 0.155 | 9.265 | 2.422 | 1.60E-02 | 1.21E-01 | -3.407 |
| TTBK1 |  | -0.137 | 6.873 | -2.42 | 1.60E-02 | 1.22E-01 | -3.412 |
| PEX11A |  | 0.122 | 6.752 | 2.419 | 1.60E-02 | 1.22E-01 | -3.414 |
| APPBP2 |  | -0.106 | 8.36 | -2.419 | 1.60E-02 | 1.22E-01 | -3.414 |
| PLXNA3 |  | 0.15 | 7.959 | 2.418 | 1.60E-02 | 1.22E-01 | -3.416 |
| MPST |  | 0.093 | 9.517 | 2.416 | 1.60E-02 | 1.23E-01 | -3.419 |
| BAIAP2 |  | 0.094 | 7.335 | 2.415 | 1.60E-02 | 1.23E-01 | -3.422 |
| RNF128 |  | -0.142 | 5.955 | -2.415 | 1.60E-02 | 1.23E-01 | -3.425 |
| CYP2J2 |  | 0.18 | 8.645 | 2.413 | 1.60E-02 | 1.23E-01 | -3.426 |
| EBAG9 |  | -0.087 | 8.322 | -2.413 | 1.60E-02 | 1.23E-01 | -3.428 |
| NAT2 |  | 0.162 | 6.324 | 2.412 | 1.60E-02 | 1.23E-01 | -3.43 |
| ZDHHC12 |  | 0.112 | 8.83 | 2.41 | 1.60E-02 | 1.24E-01 | -3.433 |
| ARF3 |  | -0.117 | 9.075 | -2.41 | 1.60E-02 | 1.24E-01 | -3.434 |
| LMNB2 |  | 0.124 | 9.11 | 2.407 | 1.60E-02 | 1.24E-01 | -3.439 |
| NHLRC2 |  | -0.138 | 6.468 | -2.408 | 1.60E-02 | 1.24E-01 | -3.44 |
| ZMYND10 |  | 0.107 | 6.308 | 2.405 | 1.60E-02 | 1.25E-01 | -3.444 |
| TP53BP2 |  | -0.102 | 8.629 | -2.403 | 1.60E-02 | 1.26E-01 | -3.451 |
| IPP |  | 0.104 | 7.251 | 2.4 | 1.70E-02 | 1.26E-01 | -3.455 |
| ALDH7A1 |  | 0.121 | 8.754 | 2.399 | 1.70E-02 | 1.26E-01 | -3.457 |
| FYN |  | -0.116 | 8.817 | -2.399 | 1.70E-02 | 1.27E-01 | -3.46 |
| KARS |  | -0.086 | 9.944 | -2.398 | 1.70E-02 | 1.27E-01 | -3.462 |
| TLR4 |  | -0.147 | 7.898 | -2.398 | 1.70E-02 | 1.27E-01 | -3.464 |
| FXR1 |  | 0.105 | 8.798 | 2.395 | 1.70E-02 | 1.27E-01 | -3.467 |
| FRMD1 |  | -0.157 | 6.347 | -2.391 | 1.70E-02 | 1.29E-01 | -3.478 |
| GFER |  | 0.096 | 7.104 | 2.388 | 1.70E-02 | 1.29E-01 | -3.482 |
| PLXDC2 |  | 0.15 | 7.294 | 2.388 | 1.70E-02 | 1.29E-01 | -3.482 |
| HOXD1 |  | -0.137 | 5.348 | -2.389 | 1.70E-02 | 1.29E-01 | -3.483 |
| RDH8 |  | -0.141 | 5.372 | -2.389 | 1.70E-02 | 1.29E-01 | -3.483 |
| SLC15A1 |  | -0.155 | 5.33 | -2.388 | 1.70E-02 | 1.29E-01 | -3.484 |
| AMPH |  | 0.155 | 5.225 | 2.386 | 1.70E-02 | 1.29E-01 | -3.487 |
| SPCS3 |  | 0.109 | 9.668 | 2.385 | 1.70E-02 | 1.30E-01 | -3.489 |
| SLC28A3 |  | -0.154 | 4.935 | -2.386 | 1.70E-02 | 1.30E-01 | -3.489 |
| KIAA1586 |  | 0.138 | 6.132 | 2.384 | 1.70E-02 | 1.30E-01 | -3.492 |
| RASSF6 |  | -0.127 | 6.177 | -2.385 | 1.70E-02 | 1.30E-01 | -3.493 |
| SGTA |  | 0.186 | 9.011 | 2.381 | 1.70E-02 | 1.31E-01 | -3.499 |
| ACRV1 |  | -0.11 | 5.043 | -2.38 | 1.80E-02 | 1.31E-01 | -3.502 |
| SNRPC |  | -0.11 | 9.147 | -2.38 | 1.80E-02 | 1.31E-01 | -3.503 |
| RPS6KB1 |  | 0.095 | 8.401 | 2.377 | 1.80E-02 | 1.31E-01 | -3.507 |
| F2RL1 |  | 0.217 | 6.814 | 2.377 | 1.80E-02 | 1.31E-01 | -3.509 |
| SSBP4 |  | 0.094 | 8.946 | 2.376 | 1.80E-02 | 1.31E-01 | -3.509 |
| SLC24A3 |  | -0.155 | 7.459 | -2.376 | 1.80E-02 | 1.32E-01 | -3.512 |
| GOSR1 |  | -0.073 | 8.798 | -2.375 | 1.80E-02 | 1.32E-01 | -3.514 |
| KCNK13 |  | 0.127 | 5.747 | 2.369 | 1.80E-02 | 1.33E-01 | -3.525 |
| CPA3 |  | -0.203 | 6.533 | -2.37 | 1.80E-02 | 1.33E-01 | -3.526 |
| ITGAL |  | -0.127 | 6.917 | -2.37 | 1.80E-02 | 1.33E-01 | -3.526 |
| LPIN2 |  | -0.117 | 8.829 | -2.37 | 1.80E-02 | 1.33E-01 | -3.527 |
| ZNF552 |  | -0.098 | 7.145 | -2.369 | 1.80E-02 | 1.33E-01 | -3.528 |
| TBXAS1 |  | -0.128 | 7.327 | -2.369 | 1.80E-02 | 1.33E-01 | -3.528 |
| PHYH |  | 0.156 | 8.628 | 2.368 | 1.80E-02 | 1.33E-01 | -3.528 |
| HIPK3 |  | -0.132 | 6.941 | -2.368 | 1.80E-02 | 1.33E-01 | -3.53 |
| FUT5 |  | -0.148 | 5.588 | -2.367 | 1.80E-02 | 1.34E-01 | -3.532 |
| CYP3A43 |  | -0.159 | 5.578 | -2.365 | 1.80E-02 | 1.34E-01 | -3.537 |
| GABARAP |  | 0.099 | 10.889 | 2.364 | 1.80E-02 | 1.34E-01 | -3.537 |
| KCTD2 |  | 0.096 | 7.815 | 2.363 | 1.80E-02 | 1.34E-01 | -3.539 |
| TRPV5 |  | -0.147 | 5.59 | -2.363 | 1.80E-02 | 1.34E-01 | -3.54 |
| DNM1 |  | 0.152 | 6.83 | 2.362 | 1.80E-02 | 1.34E-01 | -3.54 |
| ZAR1 |  | -0.136 | 5.021 | -2.363 | 1.80E-02 | 1.34E-01 | -3.541 |
| SLC13A5 |  | -0.154 | 4.778 | -2.362 | 1.80E-02 | 1.34E-01 | -3.543 |
| C12orf10 |  | -0.112 | 9.515 | -2.362 | 1.80E-02 | 1.34E-01 | -3.544 |
| PFDN2 |  | 0.098 | 9.902 | 2.36 | 1.80E-02 | 1.34E-01 | -3.545 |
| CHPF |  | 0.168 | 9.252 | 2.36 | 1.80E-02 | 1.34E-01 | -3.545 |
| SAMD1 |  | 0.117 | 7.239 | 2.359 | 1.90E-02 | 1.34E-01 | -3.547 |
| DDX27 |  | 0.078 | 9.891 | 2.358 | 1.90E-02 | 1.35E-01 | -3.549 |
| SCML4 |  | -0.113 | 5.767 | -2.359 | 1.90E-02 | 1.35E-01 | -3.55 |
| COL6A1 |  | -0.16 | 10.569 | -2.359 | 1.90E-02 | 1.35E-01 | -3.551 |
| TRIB1 |  | -0.122 | 9.882 | -2.358 | 1.90E-02 | 1.35E-01 | -3.551 |
| ACVR1 |  | -0.101 | 9.462 | -2.356 | 1.90E-02 | 1.35E-01 | -3.557 |
| TFAP2E |  | -0.121 | 5.905 | -2.355 | 1.90E-02 | 1.35E-01 | -3.558 |
| FARP2 |  | 0.093 | 6.685 | 2.354 | 1.90E-02 | 1.35E-01 | -3.558 |
| RGS3 |  | 0.083 | 6.93 | 2.354 | 1.90E-02 | 1.35E-01 | -3.559 |
| PPIF |  | -0.108 | 8.712 | -2.353 | 1.90E-02 | 1.36E-01 | -3.564 |
| GDF9 |  | 0.147 | 5.128 | 2.352 | 1.90E-02 | 1.36E-01 | -3.564 |
| ESRRB |  | -0.14 | 5.314 | -2.352 | 1.90E-02 | 1.36E-01 | -3.566 |
| GNA15 |  | -0.122 | 7.096 | -2.351 | 1.90E-02 | 1.36E-01 | -3.567 |
| USP9X |  | -0.1 | 10.458 | -2.348 | 1.90E-02 | 1.37E-01 | -3.574 |
| REPIN1 |  | -0.085 | 9.106 | -2.348 | 1.90E-02 | 1.37E-01 | -3.574 |
| DLX5 |  | 0.148 | 7.548 | 2.347 | 1.90E-02 | 1.37E-01 | -3.575 |
| PRKD1 |  | -0.125 | 6.798 | -2.346 | 1.90E-02 | 1.37E-01 | -3.579 |
| CPEB3 |  | -0.119 | 6.78 | -2.346 | 1.90E-02 | 1.37E-01 | -3.579 |
| RPL3 |  | 0.11 | 13.961 | 2.344 | 1.90E-02 | 1.37E-01 | -3.581 |
| PNPLA4 |  | 0.116 | 6.791 | 2.344 | 1.90E-02 | 1.37E-01 | -3.582 |
| HECTD2 |  | 0.127 | 6.174 | 2.343 | 1.90E-02 | 1.37E-01 | -3.582 |
| KIAA0586 |  | 0.102 | 7.656 | 2.342 | 1.90E-02 | 1.38E-01 | -3.586 |
| KCNMB1 |  | -0.135 | 7.121 | -2.341 | 2.00E-02 | 1.39E-01 | -3.591 |
| NCOA6 |  | -0.09 | 9.693 | -2.339 | 2.00E-02 | 1.39E-01 | -3.594 |
| SRPK1 |  | -0.113 | 10.382 | -2.339 | 2.00E-02 | 1.39E-01 | -3.594 |
| SMARCA2 |  | 0.086 | 8.566 | 2.337 | 2.00E-02 | 1.39E-01 | -3.596 |
| HOXA1 |  | -0.129 | 5.013 | -2.337 | 2.00E-02 | 1.39E-01 | -3.598 |
| SLC24A4 |  | -0.12 | 6.199 | -2.337 | 2.00E-02 | 1.39E-01 | -3.599 |
| LIPT1 |  | -0.102 | 7.378 | -2.337 | 2.00E-02 | 1.39E-01 | -3.6 |
| AMOT |  | 0.119 | 7.803 | 2.335 | 2.00E-02 | 1.39E-01 | -3.601 |
| NONO |  | -0.083 | 10.208 | -2.336 | 2.00E-02 | 1.39E-01 | -3.602 |
| GMPR2 |  | 0.083 | 9.326 | 2.332 | 2.00E-02 | 1.40E-01 | -3.608 |
| KAZALD1 |  | 0.144 | 6.207 | 2.331 | 2.00E-02 | 1.40E-01 | -3.609 |
| RNASEH1 |  | 0.087 | 8.782 | 2.328 | 2.00E-02 | 1.41E-01 | -3.616 |
| ODC1 |  | -0.138 | 9.683 | -2.328 | 2.00E-02 | 1.41E-01 | -3.617 |
| PDLIM2 |  | 0.116 | 7.593 | 2.326 | 2.00E-02 | 1.42E-01 | -3.621 |
| FGD3 |  | -0.099 | 7.761 | -2.323 | 2.00E-02 | 1.43E-01 | -3.629 |
| IER3 |  | 0.178 | 10.873 | 2.322 | 2.10E-02 | 1.43E-01 | -3.63 |
| KRT15 |  | 0.143 | 7.265 | 2.32 | 2.10E-02 | 1.44E-01 | -3.634 |
| SP2 |  | -0.104 | 7.387 | -2.32 | 2.10E-02 | 1.44E-01 | -3.635 |
| ZNF415 |  | 0.144 | 7.061 | 2.318 | 2.10E-02 | 1.44E-01 | -3.637 |
| PRIMA1 |  | -0.133 | 6.453 | -2.316 | 2.10E-02 | 1.45E-01 | -3.645 |
| BRF2 |  | -0.083 | 8.394 | -2.316 | 2.10E-02 | 1.45E-01 | -3.645 |
| C6orf48 |  | 0.095 | 11.122 | 2.314 | 2.10E-02 | 1.45E-01 | -3.647 |
| CDS2 |  | -0.09 | 8.584 | -2.315 | 2.10E-02 | 1.45E-01 | -3.648 |
| RAB35 |  | 0.139 | 9.799 | 2.313 | 2.10E-02 | 1.45E-01 | -3.648 |
| METAP1 |  | -0.141 | 8.792 | -2.314 | 2.10E-02 | 1.45E-01 | -3.649 |
| GUCY1B3 |  | -0.167 | 7.462 | -2.314 | 2.10E-02 | 1.45E-01 | -3.65 |
| PWP1 |  | -0.078 | 10.237 | -2.314 | 2.10E-02 | 1.45E-01 | -3.65 |
| PPP1R14C |  | -0.188 | 6.94 | -2.312 | 2.10E-02 | 1.45E-01 | -3.652 |
| NDUFC1 |  | 0.092 | 11.199 | 2.311 | 2.10E-02 | 1.45E-01 | -3.654 |
| CDK8 |  | -0.132 | 7.261 | -2.312 | 2.10E-02 | 1.45E-01 | -3.654 |
| DZIP1 |  | 0.117 | 6.416 | 2.31 | 2.10E-02 | 1.45E-01 | -3.656 |
| GEMIN4 |  | 0.098 | 8.747 | 2.308 | 2.10E-02 | 1.46E-01 | -3.661 |
| CRABP1 |  | -0.183 | 6.636 | -2.306 | 2.10E-02 | 1.47E-01 | -3.667 |
| EIF4ENIF1 |  | -0.074 | 8.579 | -2.306 | 2.10E-02 | 1.47E-01 | -3.668 |
| TNFRSF12A |  | 0.144 | 10.65 | 2.301 | 2.20E-02 | 1.48E-01 | -3.675 |
| NYX |  | -0.149 | 7.642 | -2.302 | 2.20E-02 | 1.48E-01 | -3.676 |
| PALM2 |  | -0.113 | 5.08 | -2.3 | 2.20E-02 | 1.49E-01 | -3.679 |
| ZNF124 |  | -0.127 | 6.651 | -2.3 | 2.20E-02 | 1.49E-01 | -3.68 |
| GNL2 |  | 0.09 | 9.89 | 2.297 | 2.20E-02 | 1.49E-01 | -3.684 |
| RNPEPL1 |  | 0.098 | 9.19 | 2.297 | 2.20E-02 | 1.49E-01 | -3.684 |
| FHL3 |  | -0.166 | 7.901 | -2.296 | 2.20E-02 | 1.49E-01 | -3.688 |
| SPTBN2 |  | -0.165 | 5.962 | -2.296 | 2.20E-02 | 1.49E-01 | -3.689 |
| THBD |  | -0.149 | 9.434 | -2.296 | 2.20E-02 | 1.49E-01 | -3.689 |
| KCNK17 |  | -0.297 | 6.449 | -2.295 | 2.20E-02 | 1.50E-01 | -3.69 |
| ETV6 |  | -0.113 | 7.736 | -2.293 | 2.20E-02 | 1.50E-01 | -3.695 |
| GGA1 |  | 0.098 | 8.179 | 2.291 | 2.20E-02 | 1.50E-01 | -3.696 |
| LETM1 |  | 0.092 | 7.425 | 2.291 | 2.20E-02 | 1.50E-01 | -3.697 |
| P2RY4 |  | -0.137 | 6.011 | -2.291 | 2.20E-02 | 1.50E-01 | -3.698 |
| LAPTM5 |  | -0.117 | 9.59 | -2.291 | 2.20E-02 | 1.50E-01 | -3.698 |
| B3GALT6 |  | 0.096 | 9.211 | 2.289 | 2.20E-02 | 1.51E-01 | -3.701 |
| PCDH9 |  | 0.139 | 5.273 | 2.288 | 2.20E-02 | 1.51E-01 | -3.703 |
| CECR1 |  | -0.127 | 7.545 | -2.286 | 2.30E-02 | 1.52E-01 | -3.711 |
| IL12RB1 |  | -0.11 | 6.167 | -2.285 | 2.30E-02 | 1.52E-01 | -3.712 |
| DEFA4 |  | -0.26 | 6.848 | -2.285 | 2.30E-02 | 1.52E-01 | -3.712 |
| IL1RN |  | -0.134 | 6.186 | -2.284 | 2.30E-02 | 1.52E-01 | -3.713 |
| VRK3 |  | -0.072 | 8.262 | -2.284 | 2.30E-02 | 1.52E-01 | -3.714 |
| HRK |  | 0.16 | 6.191 | 2.283 | 2.30E-02 | 1.52E-01 | -3.714 |
| BNIP3L |  | 0.126 | 11.282 | 2.281 | 2.30E-02 | 1.53E-01 | -3.719 |
| JAM2 |  | -0.122 | 8.966 | -2.279 | 2.30E-02 | 1.54E-01 | -3.725 |
| EPS8L2 |  | 0.103 | 8.106 | 2.278 | 2.30E-02 | 1.54E-01 | -3.726 |
| MCEE |  | 0.099 | 8.907 | 2.277 | 2.30E-02 | 1.54E-01 | -3.726 |
| FLT3 |  | 0.157 | 6.035 | 2.277 | 2.30E-02 | 1.54E-01 | -3.728 |
| LARS |  | -0.097 | 9.364 | -2.277 | 2.30E-02 | 1.54E-01 | -3.729 |
| GPR20 |  | -0.161 | 6.729 | -2.277 | 2.30E-02 | 1.54E-01 | -3.729 |
| CDC42BPG |  | -0.139 | 6.798 | -2.276 | 2.30E-02 | 1.54E-01 | -3.73 |
| CDO1 |  | 0.149 | 9.08 | 2.275 | 2.30E-02 | 1.54E-01 | -3.732 |
| TRIM5 |  | -0.111 | 7.314 | -2.275 | 2.30E-02 | 1.54E-01 | -3.734 |
| ZNF80 |  | 0.123 | 5.167 | 2.273 | 2.30E-02 | 1.54E-01 | -3.737 |
| GYPC |  | -0.121 | 9.817 | -2.273 | 2.30E-02 | 1.54E-01 | -3.737 |
| CASC1 |  | 0.13 | 4.824 | 2.272 | 2.30E-02 | 1.54E-01 | -3.737 |
| DPF2 |  | -0.11 | 10.397 | -2.272 | 2.30E-02 | 1.55E-01 | -3.739 |
| CBX1 |  | 0.092 | 9.676 | 2.27 | 2.40E-02 | 1.55E-01 | -3.743 |
| VDAC1 |  | -0.101 | 10.872 | -2.271 | 2.40E-02 | 1.55E-01 | -3.743 |
| ZNF213 |  | -0.127 | 7.67 | -2.27 | 2.40E-02 | 1.55E-01 | -3.743 |
| DRAP1 |  | -0.104 | 11.326 | -2.27 | 2.40E-02 | 1.55E-01 | -3.744 |
| ZNF366 |  | -0.137 | 6.723 | -2.269 | 2.40E-02 | 1.55E-01 | -3.746 |
| HAS3 |  | -0.136 | 5.898 | -2.269 | 2.40E-02 | 1.55E-01 | -3.747 |
| KRT7 |  | 0.222 | 10.298 | 2.267 | 2.40E-02 | 1.55E-01 | -3.749 |
| COMMD6 |  | 0.121 | 8.523 | 2.263 | 2.40E-02 | 1.57E-01 | -3.756 |
| PTGS1 |  | -0.159 | 6.958 | -2.263 | 2.40E-02 | 1.57E-01 | -3.759 |
| STARD3 |  | 0.087 | 8.719 | 2.261 | 2.40E-02 | 1.57E-01 | -3.761 |
| HSPA2 |  | 0.147 | 9.55 | 2.26 | 2.40E-02 | 1.58E-01 | -3.764 |
| TTR |  | 0.228 | 5.715 | 2.258 | 2.40E-02 | 1.58E-01 | -3.767 |
| CD58 |  | -0.101 | 8.5 | -2.259 | 2.40E-02 | 1.58E-01 | -3.768 |
| MTRF1 |  | -0.105 | 7.204 | -2.258 | 2.40E-02 | 1.58E-01 | -3.769 |
| MANBAL |  | 0.081 | 9.417 | 2.257 | 2.40E-02 | 1.58E-01 | -3.769 |
| ZDHHC11 |  | 0.132 | 6.551 | 2.257 | 2.40E-02 | 1.58E-01 | -3.77 |
| NR2F6 |  | 0.152 | 9.692 | 2.257 | 2.40E-02 | 1.58E-01 | -3.77 |
| NKD2 |  | -0.132 | 8.222 | -2.257 | 2.40E-02 | 1.58E-01 | -3.771 |
| CTBP2 |  | -0.072 | 9.136 | -2.256 | 2.40E-02 | 1.58E-01 | -3.773 |
| HLF |  | -0.203 | 6.188 | -2.256 | 2.40E-02 | 1.58E-01 | -3.774 |
| LSM4 |  | 0.088 | 10.027 | 2.254 | 2.50E-02 | 1.58E-01 | -3.776 |
| TFAP2A |  | 0.149 | 7.82 | 2.252 | 2.50E-02 | 1.59E-01 | -3.78 |
| ATP10D |  | -0.111 | 8.436 | -2.253 | 2.50E-02 | 1.59E-01 | -3.781 |
| TRIM22 |  | -0.171 | 8.599 | -2.252 | 2.50E-02 | 1.59E-01 | -3.783 |
| FBXW5 |  | 0.074 | 9.286 | 2.249 | 2.50E-02 | 1.60E-01 | -3.788 |
| PODXL |  | 0.113 | 9.138 | 2.248 | 2.50E-02 | 1.60E-01 | -3.789 |
| COL13A1 |  | 0.161 | 6.474 | 2.247 | 2.50E-02 | 1.60E-01 | -3.79 |
| NEU4 |  | -0.13 | 5.198 | -2.248 | 2.50E-02 | 1.60E-01 | -3.792 |
| PLAC8 |  | 0.217 | 9.545 | 2.246 | 2.50E-02 | 1.61E-01 | -3.793 |
| CNTN6 |  | -0.154 | 5.727 | -2.246 | 2.50E-02 | 1.61E-01 | -3.794 |
| RTN1 |  | -0.144 | 6.399 | -2.244 | 2.50E-02 | 1.61E-01 | -3.799 |
| SLCO1C1 |  | -0.101 | 4.631 | -2.244 | 2.50E-02 | 1.61E-01 | -3.8 |
| ZNF584 |  | 0.111 | 6.688 | 2.242 | 2.50E-02 | 1.61E-01 | -3.801 |
| HTR7 |  | -0.112 | 6.618 | -2.243 | 2.50E-02 | 1.61E-01 | -3.802 |
| TOP1MT |  | 0.099 | 8.456 | 2.242 | 2.50E-02 | 1.61E-01 | -3.802 |
| TRHDE |  | 0.091 | 4.6 | 2.241 | 2.50E-02 | 1.62E-01 | -3.804 |
| FCHO2 |  | 0.135 | 7.808 | 2.24 | 2.50E-02 | 1.62E-01 | -3.806 |
| AADAT |  | 0.13 | 5.933 | 2.239 | 2.50E-02 | 1.62E-01 | -3.808 |
| MLH1 |  | -0.075 | 10.295 | -2.239 | 2.60E-02 | 1.62E-01 | -3.809 |
| LSS |  | -0.164 | 8.233 | -2.239 | 2.60E-02 | 1.62E-01 | -3.81 |
| MMP8 |  | -0.171 | 5.386 | -2.237 | 2.60E-02 | 1.63E-01 | -3.814 |
| SLC3A2 |  | -0.144 | 10.552 | -2.236 | 2.60E-02 | 1.63E-01 | -3.816 |
| OLFM1 |  | -0.115 | 5.587 | -2.234 | 2.60E-02 | 1.64E-01 | -3.822 |
| SNRPD2 |  | 0.098 | 11.342 | 2.232 | 2.60E-02 | 1.64E-01 | -3.822 |
| MAGEA10 |  | -0.158 | 7.371 | -2.233 | 2.60E-02 | 1.64E-01 | -3.823 |
| ZNF598 |  | -0.184 | 8.973 | -2.23 | 2.60E-02 | 1.65E-01 | -3.829 |
| UPP2 |  | -0.134 | 4.874 | -2.229 | 2.60E-02 | 1.65E-01 | -3.831 |
| RSBN1 |  | -0.109 | 8.079 | -2.229 | 2.60E-02 | 1.65E-01 | -3.831 |
| MBNL1 |  | 0.104 | 9.094 | 2.227 | 2.60E-02 | 1.66E-01 | -3.834 |
| MRPS7 |  | 0.085 | 9.741 | 2.226 | 2.60E-02 | 1.66E-01 | -3.836 |
| LRRC14 |  | 0.136 | 7.961 | 2.224 | 2.60E-02 | 1.66E-01 | -3.839 |
| CXXC1 |  | 0.111 | 8.888 | 2.224 | 2.70E-02 | 1.66E-01 | -3.84 |
| DCLRE1B |  | -0.125 | 6.819 | -2.224 | 2.70E-02 | 1.67E-01 | -3.842 |
| OR1C1 |  | 0.104 | 5.071 | 2.223 | 2.70E-02 | 1.67E-01 | -3.842 |
| NPHP3 |  | 0.096 | 8.163 | 2.222 | 2.70E-02 | 1.67E-01 | -3.844 |
| GRASP |  | 0.207 | 8.351 | 2.222 | 2.70E-02 | 1.67E-01 | -3.844 |
| NDRG2 |  | 0.148 | 8.563 | 2.221 | 2.70E-02 | 1.67E-01 | -3.846 |
| JRK |  | -0.111 | 6.473 | -2.222 | 2.70E-02 | 1.67E-01 | -3.846 |
| C18orf21 |  | -0.071 | 9.286 | -2.222 | 2.70E-02 | 1.67E-01 | -3.847 |
| ITPKB |  | -0.107 | 7.968 | -2.221 | 2.70E-02 | 1.67E-01 | -3.847 |
| FAM47A |  | 0.115 | 4.853 | 2.219 | 2.70E-02 | 1.67E-01 | -3.849 |
| TTN |  | -0.094 | 5.282 | -2.22 | 2.70E-02 | 1.67E-01 | -3.85 |
| ENSA |  | 0.098 | 8.431 | 2.217 | 2.70E-02 | 1.67E-01 | -3.854 |
| SDPR |  | 0.173 | 8.77 | 2.217 | 2.70E-02 | 1.67E-01 | -3.855 |
| SERPINB8 |  | -0.117 | 8.065 | -2.218 | 2.70E-02 | 1.67E-01 | -3.855 |
| QDPR |  | -0.115 | 8.936 | -2.217 | 2.70E-02 | 1.67E-01 | -3.855 |
| PRKCQ |  | -0.122 | 6.787 | -2.217 | 2.70E-02 | 1.67E-01 | -3.855 |
| MMP15 |  | 0.107 | 8.036 | 2.216 | 2.70E-02 | 1.67E-01 | -3.857 |
| DNAH7 |  | 0.124 | 5.14 | 2.215 | 2.70E-02 | 1.67E-01 | -3.858 |
| PPFIA2 |  | 0.108 | 4.699 | 2.215 | 2.70E-02 | 1.67E-01 | -3.858 |
| SLC2A10 |  | 0.111 | 7.407 | 2.214 | 2.70E-02 | 1.68E-01 | -3.86 |
| CLTA |  | -0.082 | 11.073 | -2.215 | 2.70E-02 | 1.68E-01 | -3.861 |
| NLGN2 |  | 0.116 | 7.581 | 2.213 | 2.70E-02 | 1.68E-01 | -3.862 |
| ZFP36L1 |  | 0.157 | 11.28 | 2.211 | 2.70E-02 | 1.69E-01 | -3.867 |
| ADAM28 |  | 0.12 | 6.156 | 2.211 | 2.70E-02 | 1.69E-01 | -3.867 |
| CD8A |  | -0.14 | 6.74 | -2.211 | 2.70E-02 | 1.69E-01 | -3.868 |
| NRXN3 |  | 0.142 | 5.784 | 2.21 | 2.70E-02 | 1.69E-01 | -3.869 |
| ODF2 |  | 0.075 | 7.705 | 2.208 | 2.80E-02 | 1.69E-01 | -3.873 |
| LRP5 |  | 0.137 | 7.449 | 2.208 | 2.80E-02 | 1.69E-01 | -3.874 |
| PRPH |  | -0.115 | 5.801 | -2.208 | 2.80E-02 | 1.69E-01 | -3.875 |
| C1QTNF1 |  | -0.141 | 7.757 | -2.208 | 2.80E-02 | 1.69E-01 | -3.875 |
| CLCN6 |  | 0.091 | 7.163 | 2.207 | 2.80E-02 | 1.69E-01 | -3.876 |
| WASF1 |  | -0.111 | 7.29 | -2.207 | 2.80E-02 | 1.69E-01 | -3.876 |
| RAD9A |  | 0.093 | 7.757 | 2.205 | 2.80E-02 | 1.70E-01 | -3.88 |
| OLFML2A |  | 0.151 | 6.502 | 2.205 | 2.80E-02 | 1.70E-01 | -3.88 |
| PTGDS |  | -0.159 | 9.805 | -2.204 | 2.80E-02 | 1.70E-01 | -3.884 |
| FSTL1 |  | 0.136 | 12.234 | 2.203 | 2.80E-02 | 1.70E-01 | -3.885 |
| DGCR14 |  | 0.123 | 7.326 | 2.202 | 2.80E-02 | 1.70E-01 | -3.885 |
| GUCY2F |  | -0.141 | 4.958 | -2.202 | 2.80E-02 | 1.70E-01 | -3.887 |
| TMEM14C |  | 0.084 | 11.651 | 2.201 | 2.80E-02 | 1.70E-01 | -3.887 |
| ETNK1 |  | -0.089 | 7.597 | -2.202 | 2.80E-02 | 1.70E-01 | -3.887 |
| STIM2 |  | 0.091 | 7.068 | 2.199 | 2.80E-02 | 1.71E-01 | -3.891 |
| B4GALT7 |  | 0.11 | 8.573 | 2.199 | 2.80E-02 | 1.71E-01 | -3.891 |
| SLC34A2 |  | -0.15 | 5.527 | -2.2 | 2.80E-02 | 1.71E-01 | -3.892 |
| IFI44 |  | 0.199 | 8.899 | 2.198 | 2.80E-02 | 1.71E-01 | -3.894 |
| CTRC |  | -0.101 | 6.712 | -2.197 | 2.80E-02 | 1.72E-01 | -3.899 |
| LY6K |  | 0.175 | 7.167 | 2.192 | 2.90E-02 | 1.73E-01 | -3.907 |
| PSG11 |  | 0.205 | 10.039 | 2.192 | 2.90E-02 | 1.73E-01 | -3.907 |
| AGRP |  | 0.146 | 5.715 | 2.19 | 2.90E-02 | 1.74E-01 | -3.91 |
| AP1S2 |  | -0.118 | 8.788 | -2.189 | 2.90E-02 | 1.75E-01 | -3.915 |
| SH3GLB1 |  | -0.08 | 10.392 | -2.189 | 2.90E-02 | 1.75E-01 | -3.915 |
| KCTD17 |  | -0.135 | 8.102 | -2.187 | 2.90E-02 | 1.75E-01 | -3.918 |
| PPAP2C |  | -0.194 | 7.435 | -2.187 | 2.90E-02 | 1.75E-01 | -3.918 |
| FUNDC1 |  | 0.078 | 8.819 | 2.185 | 2.90E-02 | 1.75E-01 | -3.921 |
| MRPL38 |  | 0.09 | 8.799 | 2.184 | 2.90E-02 | 1.76E-01 | -3.923 |
| RASGRF1 |  | 0.129 | 5.214 | 2.183 | 2.90E-02 | 1.76E-01 | -3.925 |
| CD36 |  | -0.127 | 9.336 | -2.183 | 3.00E-02 | 1.76E-01 | -3.928 |
| CLIC5 |  | -0.136 | 8.375 | -2.182 | 3.00E-02 | 1.76E-01 | -3.929 |
| PFKP |  | 0.178 | 8.942 | 2.181 | 3.00E-02 | 1.76E-01 | -3.93 |
| ELF5 |  | -0.124 | 5.476 | -2.18 | 3.00E-02 | 1.77E-01 | -3.934 |
| COL15A1 |  | -0.128 | 8.782 | -2.178 | 3.00E-02 | 1.78E-01 | -3.937 |
| BMP2K |  | -0.086 | 7.601 | -2.178 | 3.00E-02 | 1.78E-01 | -3.937 |
| NUP98 |  | -0.094 | 8.499 | -2.176 | 3.00E-02 | 1.79E-01 | -3.942 |
| ERMAP |  | -0.126 | 7.005 | -2.175 | 3.00E-02 | 1.79E-01 | -3.944 |
| FSTL5 |  | -0.128 | 4.793 | -2.173 | 3.00E-02 | 1.79E-01 | -3.947 |
| DEGS1 |  | 0.092 | 11.089 | 2.17 | 3.00E-02 | 1.80E-01 | -3.952 |
| TPX2 |  | 0.131 | 8.459 | 2.169 | 3.00E-02 | 1.80E-01 | -3.954 |
| RASA2 |  | -0.114 | 7.031 | -2.17 | 3.00E-02 | 1.80E-01 | -3.954 |
| C9orf3 |  | 0.086 | 7.511 | 2.168 | 3.10E-02 | 1.81E-01 | -3.956 |
| ITGB3BP |  | 0.105 | 8.282 | 2.166 | 3.10E-02 | 1.81E-01 | -3.959 |
| KNTC1 |  | 0.098 | 8.08 | 2.164 | 3.10E-02 | 1.82E-01 | -3.963 |
| PKIA |  | -0.138 | 7.262 | -2.165 | 3.10E-02 | 1.82E-01 | -3.964 |
| ELMO3 |  | 0.106 | 7.901 | 2.163 | 3.10E-02 | 1.82E-01 | -3.965 |
| SLC12A9 |  | 0.092 | 9.764 | 2.16 | 3.10E-02 | 1.83E-01 | -3.971 |
| MARCKS |  | 0.115 | 12.35 | 2.159 | 3.10E-02 | 1.83E-01 | -3.974 |
| LY86 |  | -0.112 | 8.112 | -2.16 | 3.10E-02 | 1.83E-01 | -3.974 |
| USMG5 |  | 0.156 | 9.47 | 2.159 | 3.10E-02 | 1.83E-01 | -3.974 |
| KCNMB3 |  | -0.12 | 6.347 | -2.16 | 3.10E-02 | 1.83E-01 | -3.974 |
| RRAD |  | 0.172 | 7.778 | 2.158 | 3.10E-02 | 1.84E-01 | -3.976 |
| PDE6A |  | -0.139 | 5.098 | -2.158 | 3.10E-02 | 1.84E-01 | -3.979 |
| BTBD6 |  | 0.077 | 10.493 | 2.157 | 3.10E-02 | 1.84E-01 | -3.979 |
| USP34 |  | -0.101 | 7.977 | -2.158 | 3.10E-02 | 1.84E-01 | -3.979 |
| SYN3 |  | 0.137 | 5.745 | 2.156 | 3.10E-02 | 1.84E-01 | -3.98 |
| SLC16A7 |  | 0.125 | 5.618 | 2.156 | 3.10E-02 | 1.84E-01 | -3.98 |
| XRCC5 |  | -0.071 | 10.618 | -2.156 | 3.20E-02 | 1.84E-01 | -3.981 |
| TBC1D20 |  | -0.114 | 7.84 | -2.156 | 3.20E-02 | 1.84E-01 | -3.982 |
| RNF126 |  | 0.083 | 9.186 | 2.154 | 3.20E-02 | 1.84E-01 | -3.984 |
| UGT8 |  | -0.112 | 5.253 | -2.154 | 3.20E-02 | 1.84E-01 | -3.986 |
| KCND2 |  | 0.224 | 5.273 | 2.153 | 3.20E-02 | 1.84E-01 | -3.987 |
| KIF2B |  | -0.199 | 5.096 | -2.153 | 3.20E-02 | 1.85E-01 | -3.988 |
| BGN |  | 0.27 | 8.787 | 2.151 | 3.20E-02 | 1.85E-01 | -3.991 |
| TNRC6A |  | 0.098 | 7.104 | 2.151 | 3.20E-02 | 1.85E-01 | -3.991 |
| DVL1 |  | 0.099 | 8.389 | 2.151 | 3.20E-02 | 1.85E-01 | -3.991 |
| TTC21A |  | 0.146 | 7.454 | 2.151 | 3.20E-02 | 1.85E-01 | -3.991 |
| VAC14 |  | 0.108 | 7.449 | 2.15 | 3.20E-02 | 1.85E-01 | -3.992 |
| DHX35 |  | -0.084 | 7.935 | -2.151 | 3.20E-02 | 1.85E-01 | -3.993 |
| POLD1 |  | 0.095 | 8.673 | 2.148 | 3.20E-02 | 1.85E-01 | -3.996 |
| SELT |  | 0.102 | 9.801 | 2.146 | 3.20E-02 | 1.86E-01 | -4.001 |
| STMN2 |  | -0.155 | 5.643 | -2.147 | 3.20E-02 | 1.86E-01 | -4.001 |
| ABCA12 |  | 0.149 | 6.009 | 2.143 | 3.20E-02 | 1.87E-01 | -4.006 |
| MOCS1 |  | -0.117 | 7.229 | -2.143 | 3.30E-02 | 1.87E-01 | -4.008 |
| SYNJ1 |  | -0.118 | 7.219 | -2.142 | 3.30E-02 | 1.88E-01 | -4.01 |
| E2F1 |  | 0.114 | 6.466 | 2.141 | 3.30E-02 | 1.88E-01 | -4.01 |
| IFNA4 |  | 0.131 | 5.672 | 2.139 | 3.30E-02 | 1.88E-01 | -4.014 |
| PRRG4 |  | -0.14 | 7.624 | -2.14 | 3.30E-02 | 1.88E-01 | -4.015 |
| KLF14 |  | -0.127 | 6.731 | -2.14 | 3.30E-02 | 1.88E-01 | -4.016 |
| SETMAR |  | -0.104 | 7.089 | -2.138 | 3.30E-02 | 1.89E-01 | -4.019 |
| NUP133 |  | 0.071 | 9.111 | 2.137 | 3.30E-02 | 1.89E-01 | -4.019 |
| LEPROT |  | -0.099 | 10.644 | -2.138 | 3.30E-02 | 1.89E-01 | -4.019 |
| MRPL47 |  | 0.096 | 9.228 | 2.137 | 3.30E-02 | 1.89E-01 | -4.019 |
| SPINT1 |  | -0.124 | 8.728 | -2.137 | 3.30E-02 | 1.89E-01 | -4.021 |
| PTPRR |  | 0.139 | 5.396 | 2.136 | 3.30E-02 | 1.89E-01 | -4.021 |
| E2F5 |  | -0.138 | 7.112 | -2.137 | 3.30E-02 | 1.89E-01 | -4.022 |
| SLC18A2 |  | -0.139 | 4.868 | -2.136 | 3.30E-02 | 1.89E-01 | -4.022 |
| SLC25A25 |  | -0.112 | 8.254 | -2.136 | 3.30E-02 | 1.89E-01 | -4.023 |
| ABTB1 |  | 0.112 | 8.605 | 2.134 | 3.30E-02 | 1.89E-01 | -4.025 |
| JMJD1C |  | -0.126 | 8.285 | -2.134 | 3.30E-02 | 1.89E-01 | -4.026 |
| HDC |  | -0.172 | 5.728 | -2.132 | 3.40E-02 | 1.90E-01 | -4.032 |
| ACD |  | 0.094 | 8.533 | 2.13 | 3.40E-02 | 1.91E-01 | -4.034 |
| ACRC |  | 0.118 | 7.063 | 2.129 | 3.40E-02 | 1.91E-01 | -4.035 |
| PDK2 |  | 0.149 | 7.91 | 2.128 | 3.40E-02 | 1.91E-01 | -4.037 |
| ALCAM |  | 0.132 | 8.06 | 2.127 | 3.40E-02 | 1.91E-01 | -4.038 |
| MLLT1 |  | 0.131 | 9.042 | 2.127 | 3.40E-02 | 1.91E-01 | -4.039 |
| MYH1 |  | 0.127 | 4.602 | 2.127 | 3.40E-02 | 1.91E-01 | -4.039 |
| ADAM9 |  | 0.104 | 7.842 | 2.126 | 3.40E-02 | 1.91E-01 | -4.042 |
| EIF2B1 |  | -0.07 | 10.179 | -2.126 | 3.40E-02 | 1.92E-01 | -4.043 |
| LDHA |  | 0.119 | 12.435 | 2.124 | 3.40E-02 | 1.92E-01 | -4.044 |
| VCP |  | -0.082 | 11.03 | -2.125 | 3.40E-02 | 1.92E-01 | -4.045 |
| MARVELD2 |  | 0.103 | 6.958 | 2.123 | 3.40E-02 | 1.92E-01 | -4.046 |
| IL15RA |  | -0.11 | 7.624 | -2.123 | 3.40E-02 | 1.92E-01 | -4.049 |
| PURG |  | 0.111 | 5.488 | 2.122 | 3.40E-02 | 1.92E-01 | -4.05 |
| KEAP1 |  | -0.083 | 9.073 | -2.122 | 3.40E-02 | 1.93E-01 | -4.051 |
| RFC1 |  | 0.098 | 8.022 | 2.12 | 3.40E-02 | 1.93E-01 | -4.052 |
| ATP5J2 |  | 0.106 | 10.981 | 2.118 | 3.50E-02 | 1.94E-01 | -4.057 |
| HMGCS2 |  | -0.219 | 6.066 | -2.119 | 3.50E-02 | 1.94E-01 | -4.058 |
| MAN1A1 |  | -0.139 | 8.265 | -2.119 | 3.50E-02 | 1.94E-01 | -4.058 |
| PCSK4 |  | 0.118 | 6.383 | 2.117 | 3.50E-02 | 1.94E-01 | -4.059 |
| F11R |  | 0.156 | 9.094 | 2.117 | 3.50E-02 | 1.94E-01 | -4.06 |
| ATP2A1 |  | 0.125 | 5.251 | 2.115 | 3.50E-02 | 1.94E-01 | -4.063 |
| MPP5 |  | -0.134 | 8.721 | -2.115 | 3.50E-02 | 1.94E-01 | -4.064 |
| FABP7 |  | 0.195 | 5.158 | 2.114 | 3.50E-02 | 1.94E-01 | -4.065 |
| PIGV |  | -0.078 | 8.751 | -2.115 | 3.50E-02 | 1.94E-01 | -4.065 |
| SEMA4D |  | -0.082 | 7.578 | -2.114 | 3.50E-02 | 1.94E-01 | -4.066 |
| ACTL6A |  | -0.072 | 10.088 | -2.113 | 3.50E-02 | 1.95E-01 | -4.069 |
| RGS6 |  | -0.109 | 5.5 | -2.112 | 3.50E-02 | 1.95E-01 | -4.071 |
| MCM3AP |  | 0.071 | 9.434 | 2.109 | 3.50E-02 | 1.96E-01 | -4.075 |
| KRTCAP2 |  | 0.098 | 11.811 | 2.108 | 3.50E-02 | 1.96E-01 | -4.078 |
| SYN1 |  | -0.117 | 6.608 | -2.109 | 3.50E-02 | 1.96E-01 | -4.078 |
| ACTL6B |  | -0.12 | 5.853 | -2.108 | 3.60E-02 | 1.97E-01 | -4.08 |
| INPP5E |  | 0.107 | 7.796 | 2.106 | 3.60E-02 | 1.97E-01 | -4.081 |
| AP2S1 |  | 0.073 | 11.7 | 2.105 | 3.60E-02 | 1.97E-01 | -4.083 |
| LSP1 |  | -0.104 | 8.538 | -2.104 | 3.60E-02 | 1.98E-01 | -4.087 |
| PFDN5 |  | 0.096 | 12.506 | 2.103 | 3.60E-02 | 1.98E-01 | -4.088 |
| AMHR2 |  | -0.114 | 4.703 | -2.104 | 3.60E-02 | 1.98E-01 | -4.088 |
| RAB21 |  | -0.085 | 9.14 | -2.103 | 3.60E-02 | 1.98E-01 | -4.09 |
| ALDH1L1 |  | 0.124 | 4.945 | 2.098 | 3.60E-02 | 1.99E-01 | -4.096 |
| CHSY1 |  | -0.123 | 11.727 | -2.099 | 3.60E-02 | 1.99E-01 | -4.096 |
| HSD17B2 |  | -0.161 | 9.96 | -2.098 | 3.60E-02 | 2.00E-01 | -4.098 |
| GFM2 |  | -0.126 | 8.005 | -2.098 | 3.60E-02 | 2.00E-01 | -4.098 |
| DGKG |  | -0.123 | 6.755 | -2.098 | 3.60E-02 | 2.00E-01 | -4.099 |
| POLB |  | -0.099 | 7.634 | -2.097 | 3.60E-02 | 2.00E-01 | -4.1 |
| LCN1 |  | -0.119 | 5.552 | -2.097 | 3.70E-02 | 2.00E-01 | -4.101 |
| SMOC1 |  | -0.131 | 6.441 | -2.097 | 3.70E-02 | 2.00E-01 | -4.101 |
| LLGL1 |  | 0.115 | 6.97 | 2.096 | 3.70E-02 | 2.00E-01 | -4.102 |
| HABP4 |  | 0.089 | 7.705 | 2.096 | 3.70E-02 | 2.00E-01 | -4.102 |
| NOX3 |  | 0.106 | 4.607 | 2.095 | 3.70E-02 | 2.00E-01 | -4.103 |
| HOMER2 |  | -0.139 | 5.806 | -2.096 | 3.70E-02 | 2.00E-01 | -4.103 |
| ARID3B |  | -0.083 | 9.679 | -2.095 | 3.70E-02 | 2.00E-01 | -4.105 |
| FMO1 |  | -0.122 | 6.164 | -2.094 | 3.70E-02 | 2.00E-01 | -4.107 |
| ST3GAL2 |  | -0.109 | 7.763 | -2.093 | 3.70E-02 | 2.00E-01 | -4.108 |
| FSCN1 |  | -0.101 | 10.63 | -2.093 | 3.70E-02 | 2.00E-01 | -4.109 |
| IQCC |  | 0.111 | 6.315 | 2.09 | 3.70E-02 | 2.01E-01 | -4.112 |
| TDG |  | -0.081 | 9.686 | -2.09 | 3.70E-02 | 2.01E-01 | -4.115 |
| CDC40 |  | -0.095 | 8.16 | -2.09 | 3.70E-02 | 2.01E-01 | -4.115 |
| PREB |  | -0.084 | 9.51 | -2.088 | 3.70E-02 | 2.02E-01 | -4.118 |
| RP1L1 |  | -0.131 | 4.976 | -2.088 | 3.70E-02 | 2.02E-01 | -4.118 |
| CXCL2 |  | -0.237 | 6.883 | -2.088 | 3.70E-02 | 2.02E-01 | -4.119 |
| KDELC1 |  | 0.106 | 7.688 | 2.086 | 3.70E-02 | 2.02E-01 | -4.121 |
| C20orf85 |  | 0.113 | 5.003 | 2.084 | 3.80E-02 | 2.03E-01 | -4.125 |
| RABEP2 |  | 0.09 | 8.043 | 2.083 | 3.80E-02 | 2.03E-01 | -4.126 |
| RGS10 |  | -0.108 | 10.282 | -2.084 | 3.80E-02 | 2.03E-01 | -4.127 |
| APRT |  | 0.086 | 10.686 | 2.083 | 3.80E-02 | 2.03E-01 | -4.127 |
| BBS7 |  | 0.114 | 6.039 | 2.083 | 3.80E-02 | 2.03E-01 | -4.128 |
| MAP7 |  | -0.082 | 7.469 | -2.083 | 3.80E-02 | 2.03E-01 | -4.129 |
| RCL1 |  | -0.105 | 8.096 | -2.083 | 3.80E-02 | 2.03E-01 | -4.13 |
| APOF |  | 0.088 | 4.728 | 2.079 | 3.80E-02 | 2.04E-01 | -4.134 |
| CATSPER3 |  | 0.124 | 5.748 | 2.077 | 3.80E-02 | 2.05E-01 | -4.138 |
| AKAP14 |  | -0.112 | 4.802 | -2.076 | 3.80E-02 | 2.06E-01 | -4.143 |
| ABCA1 |  | 0.146 | 8.793 | 2.073 | 3.90E-02 | 2.07E-01 | -4.146 |
| SH2D3A |  | 0.114 | 7.588 | 2.073 | 3.90E-02 | 2.07E-01 | -4.147 |
| IDH2 |  | 0.112 | 10.538 | 2.072 | 3.90E-02 | 2.07E-01 | -4.148 |
| ARID4B |  | -0.068 | 8.104 | -2.072 | 3.90E-02 | 2.07E-01 | -4.15 |
| ADCY2 |  | -0.125 | 5.551 | -2.071 | 3.90E-02 | 2.08E-01 | -4.152 |
| FANCD2 |  | 0.128 | 6.261 | 2.069 | 3.90E-02 | 2.08E-01 | -4.154 |
| DZIP3 |  | 0.086 | 6.774 | 2.069 | 3.90E-02 | 2.08E-01 | -4.154 |
| MYD88 |  | -0.13 | 8.674 | -2.069 | 3.90E-02 | 2.08E-01 | -4.156 |
| PSME3 |  | -0.079 | 9.158 | -2.069 | 3.90E-02 | 2.08E-01 | -4.156 |
| DBR1 |  | -0.086 | 7.328 | -2.068 | 3.90E-02 | 2.08E-01 | -4.158 |
| BIN1 |  | -0.106 | 9.417 | -2.068 | 3.90E-02 | 2.08E-01 | -4.158 |
| BAIAP3 |  | 0.13 | 6.208 | 2.067 | 3.90E-02 | 2.08E-01 | -4.158 |
| ARCN1 |  | -0.064 | 10.144 | -2.068 | 3.90E-02 | 2.08E-01 | -4.159 |
| SUV420H2 |  | 0.118 | 7.041 | 2.065 | 3.90E-02 | 2.09E-01 | -4.161 |
| GAP43 |  | -0.183 | 5.138 | -2.066 | 3.90E-02 | 2.09E-01 | -4.162 |
| SOD1 |  | -0.094 | 13.397 | -2.065 | 3.90E-02 | 2.09E-01 | -4.164 |
| FGF10 |  | -0.141 | 5.258 | -2.065 | 3.90E-02 | 2.09E-01 | -4.164 |
| GNRH1 |  | 0.112 | 6.743 | 2.064 | 3.90E-02 | 2.09E-01 | -4.164 |
| CLDN5 |  | 0.157 | 8.859 | 2.061 | 4.00E-02 | 2.10E-01 | -4.171 |
| PIK3R3 |  | 0.164 | 6.145 | 2.06 | 4.00E-02 | 2.10E-01 | -4.171 |
| CNNM4 |  | 0.109 | 8.22 | 2.055 | 4.00E-02 | 2.13E-01 | -4.183 |
| PLXNB1 |  | 0.111 | 7.563 | 2.054 | 4.00E-02 | 2.13E-01 | -4.183 |
| C21orf91 |  | -0.092 | 7.693 | -2.054 | 4.10E-02 | 2.13E-01 | -4.186 |
| TFDP2 |  | -0.097 | 8.319 | -2.053 | 4.10E-02 | 2.13E-01 | -4.188 |
| SLC5A5 |  | -0.119 | 5.602 | -2.053 | 4.10E-02 | 2.13E-01 | -4.188 |
| COX7A2 |  | 0.088 | 12.461 | 2.052 | 4.10E-02 | 2.13E-01 | -4.188 |
| EDEM1 |  | -0.083 | 9.047 | -2.052 | 4.10E-02 | 2.13E-01 | -4.188 |
| TNNC1 |  | -0.151 | 6.338 | -2.052 | 4.10E-02 | 2.13E-01 | -4.189 |
| SLC27A1 |  | 0.081 | 8.935 | 2.05 | 4.10E-02 | 2.14E-01 | -4.192 |
| DYM |  | -0.061 | 9.12 | -2.051 | 4.10E-02 | 2.14E-01 | -4.192 |
| TRAF2 |  | 0.107 | 7.956 | 2.049 | 4.10E-02 | 2.14E-01 | -4.193 |
| MRPS26 |  | 0.075 | 10.334 | 2.049 | 4.10E-02 | 2.14E-01 | -4.193 |
| MYO7B |  | 0.169 | 6.488 | 2.048 | 4.10E-02 | 2.14E-01 | -4.194 |
| MLF2 |  | 0.129 | 10.454 | 2.048 | 4.10E-02 | 2.14E-01 | -4.195 |
| FAM49A |  | -0.097 | 9.123 | -2.049 | 4.10E-02 | 2.14E-01 | -4.196 |
| XPO4 |  | -0.089 | 7.63 | -2.046 | 4.10E-02 | 2.15E-01 | -4.201 |
| FBXO32 |  | -0.131 | 8.324 | -2.046 | 4.10E-02 | 2.15E-01 | -4.201 |
| MRPS34 |  | 0.074 | 9.775 | 2.044 | 4.10E-02 | 2.15E-01 | -4.202 |
| UNC93B1 |  | -0.114 | 7.635 | -2.045 | 4.10E-02 | 2.15E-01 | -4.202 |
| TRUB1 |  | -0.12 | 7.276 | -2.045 | 4.10E-02 | 2.15E-01 | -4.203 |
| TRAF6 |  | -0.069 | 7.623 | -2.045 | 4.10E-02 | 2.15E-01 | -4.203 |
| IDH3A |  | -0.095 | 8.585 | -2.043 | 4.20E-02 | 2.16E-01 | -4.207 |
| CLCN2 |  | 0.11 | 6.234 | 2.041 | 4.20E-02 | 2.16E-01 | -4.209 |
| MRPL18 |  | -0.132 | 10.605 | -2.042 | 4.20E-02 | 2.16E-01 | -4.209 |
| GPR158 |  | -0.11 | 5.851 | -2.039 | 4.20E-02 | 2.18E-01 | -4.215 |
| EGLN3 |  | 0.192 | 6.956 | 2.037 | 4.20E-02 | 2.18E-01 | -4.216 |
| SLC7A2 |  | -0.179 | 7.204 | -2.037 | 4.20E-02 | 2.19E-01 | -4.219 |
| PAOX |  | 0.097 | 7.231 | 2.032 | 4.30E-02 | 2.20E-01 | -4.226 |
| HCST |  | -0.103 | 10.144 | -2.033 | 4.30E-02 | 2.20E-01 | -4.226 |
| POU2F2 |  | -0.096 | 7.325 | -2.032 | 4.30E-02 | 2.20E-01 | -4.228 |
| AHCY |  | -0.099 | 10.939 | -2.032 | 4.30E-02 | 2.20E-01 | -4.228 |
| RNF32 |  | -0.117 | 5.672 | -2.029 | 4.30E-02 | 2.22E-01 | -4.233 |
| CRYBB1 |  | -0.127 | 5.976 | -2.029 | 4.30E-02 | 2.22E-01 | -4.234 |
| SHC3 |  | 0.125 | 6.331 | 2.027 | 4.30E-02 | 2.22E-01 | -4.236 |
| MTRF1L |  | -0.065 | 7.595 | -2.027 | 4.30E-02 | 2.22E-01 | -4.238 |
| FBXO7 |  | -0.085 | 10.401 | -2.027 | 4.30E-02 | 2.22E-01 | -4.238 |
| PES1 |  | -0.091 | 7.54 | -2.026 | 4.30E-02 | 2.22E-01 | -4.24 |
| OR2C3 |  | -0.093 | 4.819 | -2.026 | 4.30E-02 | 2.22E-01 | -4.24 |
| CYP11B1 |  | -0.106 | 5.567 | -2.026 | 4.30E-02 | 2.22E-01 | -4.24 |
| ALOX5AP |  | -0.141 | 9.421 | -2.025 | 4.30E-02 | 2.22E-01 | -4.241 |
| SLC17A4 |  | 0.12 | 5.337 | 2.024 | 4.30E-02 | 2.22E-01 | -4.241 |
| NET1 |  | 0.102 | 9.891 | 2.024 | 4.30E-02 | 2.22E-01 | -4.241 |
| PRKAR1A |  | -0.153 | 10.195 | -2.025 | 4.30E-02 | 2.22E-01 | -4.241 |
| C3AR1 |  | -0.123 | 8.012 | -2.023 | 4.40E-02 | 2.23E-01 | -4.245 |
| OR1Q1 |  | 0.113 | 4.758 | 2.02 | 4.40E-02 | 2.24E-01 | -4.249 |
| SCN2B |  | 0.131 | 5.056 | 2.018 | 4.40E-02 | 2.24E-01 | -4.252 |
| DNASE1 |  | 0.097 | 6.129 | 2.017 | 4.40E-02 | 2.24E-01 | -4.254 |
| RPL14 |  | 0.112 | 12.208 | 2.017 | 4.40E-02 | 2.24E-01 | -4.254 |
| RPS6KL1 |  | 0.092 | 5.873 | 2.017 | 4.40E-02 | 2.24E-01 | -4.254 |
| EVI5 |  | -0.129 | 8.333 | -2.018 | 4.40E-02 | 2.24E-01 | -4.255 |
| LENG1 |  | -0.11 | 6.88 | -2.018 | 4.40E-02 | 2.24E-01 | -4.255 |
| PRSS33 |  | -0.099 | 6.094 | -2.017 | 4.40E-02 | 2.25E-01 | -4.257 |
| NODAL |  | -0.127 | 5.073 | -2.016 | 4.40E-02 | 2.25E-01 | -4.258 |
| ELL2 |  | 0.117 | 8.888 | 2.015 | 4.40E-02 | 2.25E-01 | -4.258 |
| ANKHD1 |  | -0.07 | 8.539 | -2.016 | 4.40E-02 | 2.25E-01 | -4.258 |
| DDX49 |  | 0.067 | 7.929 | 2.015 | 4.40E-02 | 2.25E-01 | -4.259 |
| BRD3 |  | 0.096 | 8.843 | 2.014 | 4.40E-02 | 2.25E-01 | -4.26 |
| AFP |  | -0.12 | 5.187 | -2.015 | 4.50E-02 | 2.25E-01 | -4.261 |
| MYO15A |  | 0.14 | 5.274 | 2.012 | 4.50E-02 | 2.26E-01 | -4.265 |
| MPO |  | -0.204 | 6.385 | -2.012 | 4.50E-02 | 2.26E-01 | -4.266 |
| IL17RC |  | 0.088 | 6.966 | 2.011 | 4.50E-02 | 2.26E-01 | -4.266 |
| PHLDA2 |  | 0.142 | 10.257 | 2.01 | 4.50E-02 | 2.26E-01 | -4.268 |
| CHST6 |  | -0.141 | 7.799 | -2.009 | 4.50E-02 | 2.27E-01 | -4.271 |
| SERPINA3 |  | 0.336 | 6.457 | 2.007 | 4.50E-02 | 2.27E-01 | -4.273 |
| LCMT2 |  | -0.077 | 8.365 | -2.008 | 4.50E-02 | 2.27E-01 | -4.274 |
| IFIT1 |  | 0.19 | 9.352 | 2.005 | 4.60E-02 | 2.28E-01 | -4.278 |
| CTCF |  | -0.065 | 9.283 | -2.005 | 4.60E-02 | 2.29E-01 | -4.28 |
| TNNI2 |  | 0.167 | 7.819 | 2.004 | 4.60E-02 | 2.29E-01 | -4.28 |
| SLC4A1 |  | -0.136 | 6.991 | -2.002 | 4.60E-02 | 2.30E-01 | -4.286 |
| RTN4R |  | 0.118 | 7.529 | 2.001 | 4.60E-02 | 2.30E-01 | -4.286 |
| PPP1R15A |  | 0.108 | 10.345 | 1.999 | 4.60E-02 | 2.30E-01 | -4.288 |
| INSIG1 |  | 0.129 | 10.267 | 1.999 | 4.60E-02 | 2.30E-01 | -4.289 |
| ADORA3 |  | 0.134 | 6.409 | 1.998 | 4.60E-02 | 2.31E-01 | -4.291 |
| PBX4 |  | 0.152 | 7.752 | 1.997 | 4.60E-02 | 2.31E-01 | -4.292 |
| CDC26 |  | 0.066 | 10.043 | 1.996 | 4.60E-02 | 2.32E-01 | -4.295 |
| MDH1 |  | 0.059 | 10.866 | 1.996 | 4.60E-02 | 2.32E-01 | -4.296 |
| GPS2 |  | -0.071 | 9.849 | -1.995 | 4.70E-02 | 2.32E-01 | -4.298 |
| WNT1 |  | 0.123 | 5.412 | 1.993 | 4.70E-02 | 2.33E-01 | -4.3 |
| ARMCX5 |  | 0.102 | 7.892 | 1.993 | 4.70E-02 | 2.33E-01 | -4.301 |
| AQP6 |  | 0.091 | 4.896 | 1.993 | 4.70E-02 | 2.33E-01 | -4.301 |
| CCR10 |  | -0.165 | 7.087 | -1.993 | 4.70E-02 | 2.33E-01 | -4.302 |
| ANGPTL7 |  | -0.126 | 4.996 | -1.992 | 4.70E-02 | 2.33E-01 | -4.304 |
| ALG2 |  | -0.059 | 8.463 | -1.992 | 4.70E-02 | 2.33E-01 | -4.304 |
| ATP6V1G3 |  | 0.119 | 5.064 | 1.99 | 4.70E-02 | 2.33E-01 | -4.306 |
| QTRTD1 |  | -0.078 | 8.239 | -1.991 | 4.70E-02 | 2.33E-01 | -4.306 |
| CCDC13 |  | -0.119 | 5.67 | -1.991 | 4.70E-02 | 2.33E-01 | -4.307 |
| OXR1 |  | 0.11 | 7.886 | 1.99 | 4.70E-02 | 2.33E-01 | -4.307 |
| SLC6A14 |  | -0.118 | 4.663 | -1.988 | 4.70E-02 | 2.34E-01 | -4.312 |
| ARL6IP5 |  | 0.092 | 11.677 | 1.987 | 4.70E-02 | 2.34E-01 | -4.313 |
| PDCD2 |  | -0.071 | 8.985 | -1.987 | 4.80E-02 | 2.34E-01 | -4.313 |
| MED6 |  | -0.063 | 9.025 | -1.987 | 4.80E-02 | 2.34E-01 | -4.313 |
| TGM3 |  | -0.138 | 5.894 | -1.987 | 4.80E-02 | 2.34E-01 | -4.314 |
| POFUT1 |  | -0.066 | 6.714 | -1.987 | 4.80E-02 | 2.34E-01 | -4.314 |
| MPP4 |  | -0.107 | 4.986 | -1.986 | 4.80E-02 | 2.34E-01 | -4.315 |
| ADCK1 |  | -0.124 | 6.66 | -1.984 | 4.80E-02 | 2.35E-01 | -4.319 |
| CTSK |  | -0.124 | 9.086 | -1.983 | 4.80E-02 | 2.36E-01 | -4.321 |
| WTAP |  | -0.084 | 8.455 | -1.983 | 4.80E-02 | 2.36E-01 | -4.322 |
| PCDHB12 |  | -0.131 | 5.214 | -1.983 | 4.80E-02 | 2.36E-01 | -4.322 |
| MPDU1 |  | 0.104 | 8.736 | 1.981 | 4.80E-02 | 2.36E-01 | -4.322 |
| WFDC11 |  | 0.102 | 4.646 | 1.979 | 4.80E-02 | 2.37E-01 | -4.327 |
| TYROBP |  | -0.116 | 10.509 | -1.979 | 4.80E-02 | 2.37E-01 | -4.329 |
| RASA1 |  | -0.111 | 10.237 | -1.979 | 4.80E-02 | 2.37E-01 | -4.329 |
| PIGA |  | -0.096 | 7.944 | -1.978 | 4.90E-02 | 2.37E-01 | -4.33 |
| PSMC1 |  | -0.069 | 11.673 | -1.978 | 4.90E-02 | 2.37E-01 | -4.332 |
| NFATC2IP |  | -0.083 | 7.625 | -1.977 | 4.90E-02 | 2.38E-01 | -4.333 |
| SDS |  | -0.108 | 6.13 | -1.976 | 4.90E-02 | 2.38E-01 | -4.335 |
| DLST |  | 0.103 | 8.69 | 1.975 | 4.90E-02 | 2.38E-01 | -4.335 |
| ZIC1 |  | -0.079 | 4.803 | -1.976 | 4.90E-02 | 2.38E-01 | -4.335 |
| LMO7 |  | 0.098 | 7.254 | 1.974 | 4.90E-02 | 2.38E-01 | -4.335 |
| HIST1H2AK |  | -0.102 | 8.182 | -1.975 | 4.90E-02 | 2.38E-01 | -4.337 |
| PLEKHH1 |  | 0.145 | 6.944 | 1.974 | 4.90E-02 | 2.38E-01 | -4.337 |
| ERCC1 |  | -0.064 | 9.198 | -1.974 | 4.90E-02 | 2.38E-01 | -4.338 |
| KCTD13 |  | -0.109 | 6.491 | -1.974 | 4.90E-02 | 2.38E-01 | -4.338 |
| MTA2 |  | 0.092 | 8.722 | 1.973 | 4.90E-02 | 2.38E-01 | -4.338 |
| PPIL3 |  | 0.09 | 9.013 | 1.973 | 4.90E-02 | 2.38E-01 | -4.339 |
| GPC2 |  | 0.098 | 6.607 | 1.972 | 4.90E-02 | 2.38E-01 | -4.339 |
| MDFI |  | 0.11 | 7.673 | 1.972 | 4.90E-02 | 2.38E-01 | -4.34 |
| RARRES2 |  | -0.171 | 8.496 | -1.972 | 4.90E-02 | 2.38E-01 | -4.341 |
| CSTB |  | 0.093 | 10.994 | 1.971 | 4.90E-02 | 2.38E-01 | -4.342 |
| GLP1R |  | 0.086 | 4.809 | 1.968 | 5.00E-02 | 2.39E-01 | -4.347 |
| IFI16 |  | -0.12 | 10.148 | -1.969 | 5.00E-02 | 2.39E-01 | -4.347 |
| LRRC31 |  | -0.093 | 5.389 | -1.969 | 5.00E-02 | 2.39E-01 | -4.348 |
| FRMD4A |  | 0.108 | 7.338 | 1.966 | 5.00E-02 | 2.40E-01 | -4.351 |
| PPIC |  | 0.097 | 9.38 | 1.964 | 5.00E-02 | 2.41E-01 | -4.354 |
| COX7B2 |  | -0.216 | 5.362 | -1.964 | 5.00E-02 | 2.41E-01 | -4.356 |
| FOXF1 |  | -0.124 | 8.287 | -1.964 | 5.00E-02 | 2.41E-01 | -4.356 |
| GTF2A1 |  | 0.115 | 6.763 | 1.963 | 5.00E-02 | 2.41E-01 | -4.356 |
| PAPPA |  | -0.2 | 10.612 | -1.964 | 5.00E-02 | 2.41E-01 | -4.357 |
| PPARA |  | 0.079 | 6.207 | 1.962 | 5.00E-02 | 2.41E-01 | -4.358 |
| RAB3B |  | -0.193 | 7.932 | -1.961 | 5.10E-02 | 2.42E-01 | -4.363 |
| S100A16 |  | -0.107 | 9.538 | -1.961 | 5.10E-02 | 2.42E-01 | -4.363 |
| ENPP4 |  | -0.122 | 7.515 | -1.96 | 5.10E-02 | 2.43E-01 | -4.364 |
| XRN2 |  | -0.119 | 8.745 | -1.959 | 5.10E-02 | 2.43E-01 | -4.366 |
| SPRR3 |  | -0.162 | 6.204 | -1.959 | 5.10E-02 | 2.43E-01 | -4.366 |
| RAB30 |  | 0.1 | 6.318 | 1.958 | 5.10E-02 | 2.43E-01 | -4.366 |
| SKI |  | 0.101 | 7.497 | 1.957 | 5.10E-02 | 2.43E-01 | -4.368 |
| ARFGEF2 |  | -0.084 | 7.689 | -1.958 | 5.10E-02 | 2.43E-01 | -4.368 |
| LYPLAL1 |  | -0.07 | 8.783 | -1.957 | 5.10E-02 | 2.43E-01 | -4.37 |
| RIPK1 |  | 0.077 | 8.794 | 1.955 | 5.10E-02 | 2.43E-01 | -4.371 |
| DHX15 |  | -0.084 | 11.038 | -1.956 | 5.10E-02 | 2.44E-01 | -4.372 |
| CCNL1 |  | -0.103 | 9.254 | -1.956 | 5.10E-02 | 2.44E-01 | -4.372 |
| PIM2 |  | 0.14 | 8.209 | 1.954 | 5.10E-02 | 2.44E-01 | -4.374 |
| A1BG |  | 0.118 | 7.002 | 1.954 | 5.10E-02 | 2.44E-01 | -4.374 |
| ICA1 |  | -0.098 | 7.669 | -1.955 | 5.10E-02 | 2.44E-01 | -4.374 |
| MYEF2 |  | 0.122 | 5.719 | 1.953 | 5.10E-02 | 2.44E-01 | -4.375 |
| PNKP |  | 0.104 | 8.915 | 1.952 | 5.10E-02 | 2.44E-01 | -4.377 |
| ROBO1 |  | 0.116 | 5.958 | 1.952 | 5.10E-02 | 2.44E-01 | -4.377 |
| PRG2 |  | 0.275 | 9.173 | 1.952 | 5.20E-02 | 2.44E-01 | -4.378 |
| DNAJC3 |  | 0.112 | 8.041 | 1.951 | 5.20E-02 | 2.44E-01 | -4.379 |
| PRKD3 |  | 0.079 | 7.696 | 1.951 | 5.20E-02 | 2.44E-01 | -4.379 |
| PCOLCE |  | -0.146 | 9.062 | -1.952 | 5.20E-02 | 2.44E-01 | -4.379 |
| RBM25 |  | 0.1 | 10.041 | 1.951 | 5.20E-02 | 2.44E-01 | -4.379 |
| PNLDC1 |  | -0.134 | 5.025 | -1.951 | 5.20E-02 | 2.44E-01 | -4.38 |
| PDCD7 |  | 0.078 | 9.581 | 1.95 | 5.20E-02 | 2.44E-01 | -4.382 |
| CHST10 |  | -0.117 | 6.841 | -1.951 | 5.20E-02 | 2.44E-01 | -4.382 |
| KLRC3 |  | 0.142 | 5.703 | 1.949 | 5.20E-02 | 2.44E-01 | -4.382 |
| IFNGR1 |  | -0.12 | 11.336 | -1.95 | 5.20E-02 | 2.44E-01 | -4.382 |
| IL17D |  | 0.15 | 5.931 | 1.947 | 5.20E-02 | 2.45E-01 | -4.386 |
| PPP2R2B |  | 0.122 | 7.282 | 1.947 | 5.20E-02 | 2.45E-01 | -4.387 |
| PDPK1 |  | -0.068 | 8.467 | -1.948 | 5.20E-02 | 2.45E-01 | -4.387 |
| SRP9 |  | -0.097 | 10.001 | -1.948 | 5.20E-02 | 2.45E-01 | -4.387 |
| INPP1 |  | -0.09 | 8.685 | -1.947 | 5.20E-02 | 2.45E-01 | -4.388 |
| MRPL24 |  | 0.089 | 10.211 | 1.945 | 5.20E-02 | 2.45E-01 | -4.39 |
| TUBGCP5 |  | 0.094 | 6.905 | 1.945 | 5.20E-02 | 2.45E-01 | -4.39 |
| CLCA2 |  | 0.116 | 5.137 | 1.945 | 5.20E-02 | 2.45E-01 | -4.391 |
| MYH14 |  | -0.105 | 7.253 | -1.944 | 5.30E-02 | 2.46E-01 | -4.394 |
| SCOC |  | 0.133 | 8.61 | 1.941 | 5.30E-02 | 2.47E-01 | -4.397 |
| PROP1 |  | -0.139 | 6.87 | -1.942 | 5.30E-02 | 2.47E-01 | -4.397 |
| CRYM |  | -0.143 | 6.39 | -1.941 | 5.30E-02 | 2.47E-01 | -4.399 |
| HLA-DPA1 |  | -0.181 | 8.853 | -1.941 | 5.30E-02 | 2.47E-01 | -4.4 |
| TBX5 |  | -0.115 | 5.658 | -1.941 | 5.30E-02 | 2.47E-01 | -4.4 |
| PRC1 |  | 0.116 | 9.314 | 1.938 | 5.30E-02 | 2.48E-01 | -4.403 |
| CRYGC |  | -0.095 | 4.647 | -1.939 | 5.30E-02 | 2.48E-01 | -4.403 |
| TNFRSF19 |  | 0.111 | 5.376 | 1.938 | 5.30E-02 | 2.48E-01 | -4.403 |
| INSL4 |  | 0.155 | 8.727 | 1.936 | 5.30E-02 | 2.48E-01 | -4.407 |
| CAPN2 |  | 0.1 | 10.492 | 1.936 | 5.30E-02 | 2.48E-01 | -4.408 |
| COL3A1 |  | 0.141 | 10.468 | 1.935 | 5.30E-02 | 2.48E-01 | -4.408 |
| TNFRSF1B |  | -0.125 | 8.568 | -1.936 | 5.40E-02 | 2.49E-01 | -4.409 |
| CXCR6 |  | 0.147 | 7.232 | 1.934 | 5.40E-02 | 2.49E-01 | -4.411 |
| DOCK2 |  | -0.105 | 7.735 | -1.934 | 5.40E-02 | 2.49E-01 | -4.411 |
| SMYD5 |  | -0.077 | 7.556 | -1.934 | 5.40E-02 | 2.49E-01 | -4.413 |
| MAP1LC3C |  | -0.214 | 5.994 | -1.932 | 5.40E-02 | 2.50E-01 | -4.415 |
| RP2 |  | -0.09 | 9.212 | -1.931 | 5.40E-02 | 2.50E-01 | -4.417 |
| SRPX2 |  | -0.121 | 8.149 | -1.931 | 5.40E-02 | 2.50E-01 | -4.417 |
| DLG5 |  | -0.137 | 8.911 | -1.931 | 5.40E-02 | 2.50E-01 | -4.417 |
| RAB5B |  | -0.102 | 9.339 | -1.931 | 5.40E-02 | 2.50E-01 | -4.418 |
| CEBPG |  | 0.095 | 9.51 | 1.93 | 5.40E-02 | 2.50E-01 | -4.419 |
| FDPS |  | 0.069 | 10.66 | 1.928 | 5.40E-02 | 2.50E-01 | -4.421 |
| GPM6A |  | -0.118 | 5.141 | -1.929 | 5.40E-02 | 2.50E-01 | -4.421 |
| NOXO1 |  | -0.115 | 6.073 | -1.929 | 5.40E-02 | 2.50E-01 | -4.422 |
| TBX22 |  | 0.08 | 4.585 | 1.928 | 5.40E-02 | 2.50E-01 | -4.422 |
| PTPRA |  | 0.06 | 9.498 | 1.927 | 5.40E-02 | 2.50E-01 | -4.423 |
| ABHD1 |  | -0.1 | 5.658 | -1.928 | 5.50E-02 | 2.50E-01 | -4.423 |
| FBXO6 |  | -0.12 | 7.114 | -1.928 | 5.50E-02 | 2.50E-01 | -4.424 |
| HTR1D |  | 0.1 | 5.815 | 1.927 | 5.50E-02 | 2.50E-01 | -4.424 |
| DOK6 |  | -0.118 | 6.392 | -1.926 | 5.50E-02 | 2.51E-01 | -4.426 |
| C9orf24 |  | 0.098 | 5.809 | 1.924 | 5.50E-02 | 2.51E-01 | -4.428 |
| SOX2 |  | 0.101 | 5.059 | 1.924 | 5.50E-02 | 2.51E-01 | -4.428 |
| BCL7C |  | 0.081 | 8.124 | 1.924 | 5.50E-02 | 2.51E-01 | -4.429 |
| SRF |  | 0.106 | 9.051 | 1.923 | 5.50E-02 | 2.51E-01 | -4.43 |
| CSRP2 |  | 0.114 | 9.804 | 1.923 | 5.50E-02 | 2.51E-01 | -4.431 |
| TIPARP |  | 0.085 | 8.5 | 1.923 | 5.50E-02 | 2.51E-01 | -4.431 |
| RBM10 |  | 0.098 | 9.59 | 1.922 | 5.50E-02 | 2.51E-01 | -4.432 |
| NCDN |  | -0.087 | 6.882 | -1.923 | 5.50E-02 | 2.51E-01 | -4.432 |
| TMEM2 |  | -0.097 | 10.256 | -1.923 | 5.50E-02 | 2.51E-01 | -4.432 |
| SLC16A9 |  | -0.139 | 5.936 | -1.921 | 5.50E-02 | 2.52E-01 | -4.435 |
| CLASP2 |  | -0.11 | 7.164 | -1.92 | 5.50E-02 | 2.53E-01 | -4.437 |
| NDST1 |  | 0.093 | 8.525 | 1.918 | 5.60E-02 | 2.53E-01 | -4.439 |
| STAT5B |  | -0.11 | 9.435 | -1.918 | 5.60E-02 | 2.53E-01 | -4.441 |
| SENP7 |  | -0.104 | 7.33 | -1.918 | 5.60E-02 | 2.53E-01 | -4.441 |
| EPDR1 |  | -0.155 | 7.793 | -1.918 | 5.60E-02 | 2.53E-01 | -4.442 |
| TCN2 |  | 0.122 | 7.102 | 1.916 | 5.60E-02 | 2.54E-01 | -4.443 |
| SAE1 |  | 0.057 | 10.846 | 1.915 | 5.60E-02 | 2.54E-01 | -4.445 |
| HIST1H2BO |  | 0.11 | 8.302 | 1.915 | 5.60E-02 | 2.54E-01 | -4.446 |
| SPTBN1 |  | -0.11 | 7.61 | -1.914 | 5.60E-02 | 2.55E-01 | -4.449 |
| ZCCHC14 |  | 0.078 | 8.249 | 1.913 | 5.60E-02 | 2.55E-01 | -4.449 |
| LMAN2L |  | 0.07 | 9.244 | 1.913 | 5.60E-02 | 2.55E-01 | -4.449 |
| BDKRB1 |  | -0.159 | 5.979 | -1.913 | 5.60E-02 | 2.55E-01 | -4.45 |
| PELP1 |  | 0.07 | 9.586 | 1.911 | 5.70E-02 | 2.56E-01 | -4.453 |
| FUT11 |  | -0.088 | 7.595 | -1.908 | 5.70E-02 | 2.58E-01 | -4.46 |
| AQP3 |  | 0.159 | 7.159 | 1.907 | 5.70E-02 | 2.58E-01 | -4.46 |
| CHST7 |  | -0.129 | 8.378 | -1.908 | 5.70E-02 | 2.58E-01 | -4.46 |
| ICAM3 |  | -0.098 | 9.229 | -1.907 | 5.70E-02 | 2.58E-01 | -4.462 |
| CD37 |  | -0.115 | 7.358 | -1.906 | 5.70E-02 | 2.58E-01 | -4.463 |
| SMOC2 |  | -0.143 | 7.102 | -1.906 | 5.70E-02 | 2.58E-01 | -4.463 |
| TBC1D7 |  | 0.078 | 8.876 | 1.905 | 5.70E-02 | 2.58E-01 | -4.463 |
| RASGRP2 |  | 0.106 | 8.496 | 1.905 | 5.70E-02 | 2.58E-01 | -4.464 |
| PDC |  | -0.12 | 4.744 | -1.905 | 5.70E-02 | 2.58E-01 | -4.465 |
| C1QL1 |  | 0.125 | 5.807 | 1.902 | 5.80E-02 | 2.59E-01 | -4.469 |
| PHC3 |  | -0.096 | 7.461 | -1.903 | 5.80E-02 | 2.59E-01 | -4.469 |
| PARN |  | 0.063 | 8.546 | 1.9 | 5.80E-02 | 2.60E-01 | -4.472 |
| GOLGA3 |  | 0.075 | 9.191 | 1.9 | 5.80E-02 | 2.60E-01 | -4.473 |
| ASPM |  | 0.142 | 7.825 | 1.899 | 5.80E-02 | 2.60E-01 | -4.474 |
| CNGB1 |  | -0.086 | 5.6 | -1.9 | 5.80E-02 | 2.60E-01 | -4.474 |
| REM1 |  | -0.113 | 5.525 | -1.9 | 5.80E-02 | 2.60E-01 | -4.475 |
| RBKS |  | 0.105 | 6.983 | 1.898 | 5.80E-02 | 2.60E-01 | -4.475 |
| AMMECR1 |  | 0.113 | 7.513 | 1.898 | 5.80E-02 | 2.60E-01 | -4.475 |
| INPPL1 |  | 0.086 | 9.681 | 1.898 | 5.80E-02 | 2.60E-01 | -4.477 |
| SSBP3 |  | -0.119 | 7.354 | -1.899 | 5.80E-02 | 2.60E-01 | -4.477 |
| BRS3 |  | 0.094 | 5.005 | 1.897 | 5.80E-02 | 2.60E-01 | -4.477 |
| RHOB |  | 0.174 | 9.3 | 1.896 | 5.90E-02 | 2.60E-01 | -4.479 |
| SEC61B |  | 0.072 | 11.969 | 1.896 | 5.90E-02 | 2.60E-01 | -4.479 |
| AES |  | 0.105 | 10.903 | 1.895 | 5.90E-02 | 2.61E-01 | -4.481 |
| ZNF140 |  | 0.09 | 6.863 | 1.895 | 5.90E-02 | 2.61E-01 | -4.482 |
| TPTE |  | 0.142 | 5.727 | 1.895 | 5.90E-02 | 2.61E-01 | -4.482 |
| P2RY10 |  | -0.123 | 5.819 | -1.895 | 5.90E-02 | 2.61E-01 | -4.484 |
| KRTAP5-9 |  | -0.129 | 6.817 | -1.893 | 5.90E-02 | 2.62E-01 | -4.487 |
| CDON |  | -0.094 | 5.623 | -1.892 | 5.90E-02 | 2.62E-01 | -4.489 |
| ZBP1 |  | 0.102 | 6.272 | 1.891 | 5.90E-02 | 2.62E-01 | -4.489 |
| FGF18 |  | 0.112 | 5.608 | 1.889 | 5.90E-02 | 2.63E-01 | -4.491 |
| LASP1 |  | 0.098 | 11.513 | 1.889 | 5.90E-02 | 2.63E-01 | -4.492 |
| USP2 |  | -0.076 | 6.065 | -1.889 | 6.00E-02 | 2.63E-01 | -4.494 |
| SLC27A2 |  | -0.153 | 8.189 | -1.889 | 6.00E-02 | 2.63E-01 | -4.494 |
| NRGN |  | 0.121 | 8.375 | 1.887 | 6.00E-02 | 2.63E-01 | -4.495 |
| CSF3R |  | -0.158 | 10.08 | -1.888 | 6.00E-02 | 2.63E-01 | -4.495 |
| FANK1 |  | 0.107 | 6.137 | 1.887 | 6.00E-02 | 2.63E-01 | -4.496 |
| ASCL2 |  | 0.182 | 8.113 | 1.886 | 6.00E-02 | 2.63E-01 | -4.497 |
| ATOX1 |  | 0.079 | 10.958 | 1.886 | 6.00E-02 | 2.63E-01 | -4.497 |
| IL7 |  | -0.147 | 5.323 | -1.886 | 6.00E-02 | 2.64E-01 | -4.499 |
| COIL |  | 0.061 | 8.772 | 1.885 | 6.00E-02 | 2.64E-01 | -4.5 |
| NFKB1 |  | -0.086 | 10.323 | -1.885 | 6.00E-02 | 2.64E-01 | -4.5 |
| MRPL35 |  | -0.083 | 8.31 | -1.885 | 6.00E-02 | 2.64E-01 | -4.5 |
| STRA6 |  | -0.101 | 8.936 | -1.885 | 6.00E-02 | 2.64E-01 | -4.501 |
| ASB1 |  | 0.088 | 7.859 | 1.883 | 6.00E-02 | 2.64E-01 | -4.502 |
| SLC39A1 |  | 0.071 | 10.59 | 1.883 | 6.00E-02 | 2.64E-01 | -4.503 |
| SCRN2 |  | 0.112 | 7.616 | 1.882 | 6.00E-02 | 2.64E-01 | -4.504 |
| CGRRF1 |  | -0.094 | 7.923 | -1.882 | 6.00E-02 | 2.65E-01 | -4.506 |
| CASP1 |  | -0.115 | 9.274 | -1.882 | 6.10E-02 | 2.65E-01 | -4.506 |
| SLC1A6 |  | 0.168 | 5.783 | 1.881 | 6.10E-02 | 2.65E-01 | -4.507 |
| IL17F |  | -0.126 | 5.926 | -1.881 | 6.10E-02 | 2.65E-01 | -4.508 |
| CHST8 |  | -0.147 | 6.199 | -1.88 | 6.10E-02 | 2.65E-01 | -4.509 |
| SLA |  | -0.109 | 8.403 | -1.879 | 6.10E-02 | 2.66E-01 | -4.512 |
| SLC25A19 |  | 0.089 | 7.797 | 1.877 | 6.10E-02 | 2.66E-01 | -4.513 |
| ARG2 |  | -0.169 | 7.211 | -1.877 | 6.10E-02 | 2.67E-01 | -4.516 |
| PLTP |  | -0.137 | 9.632 | -1.876 | 6.10E-02 | 2.67E-01 | -4.518 |
| FKBP4 |  | -0.081 | 9.197 | -1.875 | 6.10E-02 | 2.67E-01 | -4.518 |
| ZCCHC6 |  | 0.085 | 8.448 | 1.874 | 6.10E-02 | 2.67E-01 | -4.518 |
| CLPS |  | -0.123 | 6.015 | -1.875 | 6.10E-02 | 2.67E-01 | -4.518 |
| NAALADL1 |  | 0.106 | 6.083 | 1.874 | 6.20E-02 | 2.67E-01 | -4.519 |
| CHRNB1 |  | 0.105 | 6.918 | 1.872 | 6.20E-02 | 2.69E-01 | -4.523 |
| CNOT1 |  | -0.081 | 7.971 | -1.872 | 6.20E-02 | 2.69E-01 | -4.524 |
| SHQ1 |  | -0.082 | 7.398 | -1.872 | 6.20E-02 | 2.69E-01 | -4.525 |
| DCLRE1C |  | -0.065 | 7.81 | -1.872 | 6.20E-02 | 2.69E-01 | -4.525 |
| SMCR8 |  | -0.072 | 6.717 | -1.871 | 6.20E-02 | 2.69E-01 | -4.526 |
| BAG1 |  | -0.076 | 9.164 | -1.87 | 6.20E-02 | 2.69E-01 | -4.527 |
| ATP10A |  | 0.084 | 6.249 | 1.869 | 6.20E-02 | 2.69E-01 | -4.528 |
| MAP2K7 |  | -0.087 | 7.576 | -1.869 | 6.20E-02 | 2.70E-01 | -4.53 |
| FZD1 |  | -0.114 | 8.1 | -1.868 | 6.20E-02 | 2.70E-01 | -4.531 |
| CSTF2 |  | -0.072 | 8.9 | -1.868 | 6.20E-02 | 2.70E-01 | -4.531 |
| SOCS4 |  | -0.114 | 7.23 | -1.867 | 6.30E-02 | 2.70E-01 | -4.533 |
| DDR2 |  | -0.105 | 6.627 | -1.866 | 6.30E-02 | 2.71E-01 | -4.535 |
| GIMAP4 |  | -0.116 | 9.276 | -1.865 | 6.30E-02 | 2.71E-01 | -4.536 |
| GPHN |  | -0.096 | 6.9 | -1.864 | 6.30E-02 | 2.71E-01 | -4.538 |
| NDST2 |  | 0.055 | 7.944 | 1.863 | 6.30E-02 | 2.72E-01 | -4.539 |
| ATP6AP1 |  | 0.117 | 11.536 | 1.862 | 6.30E-02 | 2.72E-01 | -4.541 |
| STRN3 |  | -0.091 | 8.42 | -1.862 | 6.30E-02 | 2.72E-01 | -4.542 |
| ZNF576 |  | 0.072 | 7.39 | 1.86 | 6.30E-02 | 2.73E-01 | -4.543 |
| CD52 |  | 0.15 | 8.153 | 1.859 | 6.40E-02 | 2.73E-01 | -4.545 |
| MBNL3 |  | -0.095 | 8.833 | -1.86 | 6.40E-02 | 2.73E-01 | -4.545 |
| YWHAB |  | -0.061 | 12.128 | -1.86 | 6.40E-02 | 2.73E-01 | -4.546 |
| CCL19 |  | -0.094 | 5.954 | -1.859 | 6.40E-02 | 2.73E-01 | -4.548 |
| OR3A2 |  | -0.112 | 4.974 | -1.859 | 6.40E-02 | 2.73E-01 | -4.548 |
| TNPO3 |  | 0.08 | 8.281 | 1.856 | 6.40E-02 | 2.74E-01 | -4.55 |
| TRIM47 |  | -0.095 | 7.648 | -1.856 | 6.40E-02 | 2.75E-01 | -4.553 |
| TCEB2 |  | 0.078 | 11.006 | 1.854 | 6.40E-02 | 2.75E-01 | -4.555 |
| LRRN3 |  | -0.146 | 6.834 | -1.854 | 6.40E-02 | 2.75E-01 | -4.556 |
| IDI1 |  | 0.1 | 9.244 | 1.853 | 6.50E-02 | 2.75E-01 | -4.557 |
| ARHGEF2 |  | -0.082 | 9.223 | -1.853 | 6.50E-02 | 2.75E-01 | -4.557 |
| GCM2 |  | -0.092 | 4.61 | -1.852 | 6.50E-02 | 2.76E-01 | -4.559 |
| PSMB8 |  | -0.089 | 9.617 | -1.852 | 6.50E-02 | 2.76E-01 | -4.56 |
| MS4A4A |  | -0.114 | 8.365 | -1.852 | 6.50E-02 | 2.76E-01 | -4.56 |
| PLEKHM1 |  | -0.072 | 8.331 | -1.852 | 6.50E-02 | 2.76E-01 | -4.56 |
| USF2 |  | 0.083 | 8.689 | 1.85 | 6.50E-02 | 2.76E-01 | -4.561 |
| MAN2C1 |  | 0.084 | 8.65 | 1.85 | 6.50E-02 | 2.76E-01 | -4.562 |
| NDUFS7 |  | 0.079 | 10.188 | 1.849 | 6.50E-02 | 2.76E-01 | -4.563 |
| WNT2 |  | -0.161 | 7.894 | -1.85 | 6.50E-02 | 2.76E-01 | -4.563 |
| UMODL1 |  | -0.114 | 4.829 | -1.849 | 6.50E-02 | 2.76E-01 | -4.564 |
| MS4A6A |  | -0.113 | 8.767 | -1.848 | 6.50E-02 | 2.77E-01 | -4.566 |
| HTR3A |  | -0.119 | 5.722 | -1.848 | 6.50E-02 | 2.77E-01 | -4.566 |
| ORAOV1 |  | 0.067 | 7.801 | 1.846 | 6.50E-02 | 2.77E-01 | -4.568 |
| APCS |  | -0.136 | 5.817 | -1.847 | 6.50E-02 | 2.77E-01 | -4.569 |
| MRPS15 |  | -0.073 | 10.529 | -1.847 | 6.60E-02 | 2.77E-01 | -4.569 |
| PRKAA2 |  | 0.098 | 5.147 | 1.845 | 6.60E-02 | 2.77E-01 | -4.569 |
| RASGRP3 |  | 0.105 | 7.011 | 1.845 | 6.60E-02 | 2.77E-01 | -4.569 |
| CSTL1 |  | -0.1 | 5.053 | -1.846 | 6.60E-02 | 2.77E-01 | -4.57 |
| SH2D4A |  | 0.134 | 7.019 | 1.845 | 6.60E-02 | 2.77E-01 | -4.571 |
| SLC9A6 |  | -0.088 | 7.388 | -1.846 | 6.60E-02 | 2.77E-01 | -4.571 |
| POLR2C |  | -0.064 | 9.419 | -1.845 | 6.60E-02 | 2.77E-01 | -4.571 |
| FBXO17 |  | -0.104 | 7.286 | -1.845 | 6.60E-02 | 2.77E-01 | -4.572 |
| HCLS1 |  | -0.084 | 10.56 | -1.844 | 6.60E-02 | 2.77E-01 | -4.573 |
| DDI1 |  | -0.105 | 5.026 | -1.844 | 6.60E-02 | 2.77E-01 | -4.573 |
| TAT |  | 0.083 | 4.919 | 1.843 | 6.60E-02 | 2.77E-01 | -4.574 |
| YAP1 |  | 0.118 | 8.899 | 1.843 | 6.60E-02 | 2.77E-01 | -4.574 |
| ABCF3 |  | -0.081 | 8.629 | -1.843 | 6.60E-02 | 2.77E-01 | -4.575 |
| UCHL5 |  | 0.062 | 8.175 | 1.841 | 6.60E-02 | 2.78E-01 | -4.577 |
| HSF1 |  | 0.055 | 9.753 | 1.84 | 6.60E-02 | 2.78E-01 | -4.578 |
| PLVAP |  | -0.117 | 8.301 | -1.841 | 6.60E-02 | 2.78E-01 | -4.578 |
| TNR |  | 0.1 | 5.582 | 1.84 | 6.60E-02 | 2.78E-01 | -4.579 |
| GLA |  | 0.075 | 10.599 | 1.84 | 6.60E-02 | 2.78E-01 | -4.579 |
| USP32 |  | -0.075 | 7.337 | -1.84 | 6.60E-02 | 2.78E-01 | -4.58 |
| GPR65 |  | -0.108 | 6.939 | -1.84 | 6.60E-02 | 2.78E-01 | -4.58 |
| FBN2 |  | -0.114 | 10.431 | -1.84 | 6.70E-02 | 2.78E-01 | -4.581 |
| MAGEA8 |  | -0.123 | 7.966 | -1.84 | 6.70E-02 | 2.78E-01 | -4.581 |
| C9orf84 |  | -0.121 | 4.869 | -1.838 | 6.70E-02 | 2.78E-01 | -4.584 |
| EFNA1 |  | 0.104 | 9.504 | 1.837 | 6.70E-02 | 2.78E-01 | -4.584 |
| GAS2L2 |  | -0.095 | 6.04 | -1.838 | 6.70E-02 | 2.78E-01 | -4.584 |
| ANGPTL2 |  | -0.133 | 7.686 | -1.837 | 6.70E-02 | 2.79E-01 | -4.586 |
| SCN4A |  | -0.124 | 5.552 | -1.837 | 6.70E-02 | 2.79E-01 | -4.586 |
| DEDD |  | -0.079 | 8.15 | -1.837 | 6.70E-02 | 2.79E-01 | -4.586 |
| PF4 |  | 0.125 | 8.033 | 1.835 | 6.70E-02 | 2.79E-01 | -4.587 |
| PACS2 |  | 0.094 | 7.31 | 1.835 | 6.70E-02 | 2.79E-01 | -4.588 |
| NOTCH2NL |  | -0.113 | 8.618 | -1.836 | 6.70E-02 | 2.79E-01 | -4.588 |
| CCK |  | -0.284 | 7.939 | -1.833 | 6.70E-02 | 2.80E-01 | -4.592 |
| TM7SF2 |  | 0.102 | 8.023 | 1.832 | 6.70E-02 | 2.80E-01 | -4.592 |
| NDEL1 |  | 0.086 | 10.022 | 1.831 | 6.80E-02 | 2.81E-01 | -4.595 |
| ABCC11 |  | 0.115 | 5.285 | 1.831 | 6.80E-02 | 2.81E-01 | -4.595 |
| RPL3L |  | -0.11 | 6.382 | -1.83 | 6.80E-02 | 2.81E-01 | -4.597 |
| AKAP10 |  | -0.107 | 7.779 | -1.83 | 6.80E-02 | 2.81E-01 | -4.598 |
| MLLT4 |  | 0.095 | 6.588 | 1.828 | 6.80E-02 | 2.82E-01 | -4.6 |
| CDH6 |  | -0.15 | 5.487 | -1.828 | 6.80E-02 | 2.82E-01 | -4.602 |
| SNAPC2 |  | -0.076 | 9.741 | -1.827 | 6.80E-02 | 2.82E-01 | -4.602 |
| COX6B2 |  | 0.108 | 6.547 | 1.826 | 6.80E-02 | 2.83E-01 | -4.603 |
| SCGB2A2 |  | 0.095 | 4.67 | 1.826 | 6.90E-02 | 2.83E-01 | -4.604 |
| RANBP3 |  | 0.078 | 7.59 | 1.825 | 6.90E-02 | 2.83E-01 | -4.604 |
| DDX25 |  | 0.104 | 4.8 | 1.825 | 6.90E-02 | 2.83E-01 | -4.606 |
| STARD8 |  | -0.089 | 8.538 | -1.825 | 6.90E-02 | 2.83E-01 | -4.607 |
| CDH5 |  | -0.099 | 8.969 | -1.825 | 6.90E-02 | 2.83E-01 | -4.608 |
| COPA |  | -0.08 | 10.152 | -1.824 | 6.90E-02 | 2.83E-01 | -4.608 |
| SLCO4C1 |  | 0.107 | 5.472 | 1.823 | 6.90E-02 | 2.83E-01 | -4.609 |
| ANGPTL4 |  | -0.153 | 9.076 | -1.823 | 6.90E-02 | 2.84E-01 | -4.61 |
| ACTN1 |  | 0.096 | 11.457 | 1.821 | 6.90E-02 | 2.84E-01 | -4.611 |
| PDK3 |  | -0.095 | 7.715 | -1.821 | 6.90E-02 | 2.85E-01 | -4.614 |
| NDUFB3 |  | 0.087 | 10.282 | 1.82 | 6.90E-02 | 2.85E-01 | -4.614 |
| IGF1R |  | 0.078 | 8.018 | 1.82 | 6.90E-02 | 2.85E-01 | -4.614 |
| STYK1 |  | -0.129 | 6.822 | -1.82 | 6.90E-02 | 2.85E-01 | -4.615 |
| SRPR |  | -0.08 | 10.046 | -1.82 | 7.00E-02 | 2.85E-01 | -4.616 |
| GOT2 |  | -0.063 | 10.503 | -1.819 | 7.00E-02 | 2.85E-01 | -4.617 |
| TGFBI |  | -0.117 | 12.269 | -1.819 | 7.00E-02 | 2.85E-01 | -4.617 |
| SBF1 |  | 0.071 | 9.009 | 1.818 | 7.00E-02 | 2.85E-01 | -4.618 |
| SEC61A2 |  | -0.086 | 6.66 | -1.819 | 7.00E-02 | 2.85E-01 | -4.618 |
| FBXO11 |  | 0.076 | 7.873 | 1.818 | 7.00E-02 | 2.85E-01 | -4.618 |
| RARRES3 |  | 0.127 | 9.754 | 1.817 | 7.00E-02 | 2.85E-01 | -4.618 |
| UHRF1 |  | 0.096 | 7.953 | 1.817 | 7.00E-02 | 2.85E-01 | -4.618 |
| SLC22A14 |  | -0.106 | 6.637 | -1.817 | 7.00E-02 | 2.85E-01 | -4.62 |
| TUBB |  | 0.1 | 11.061 | 1.816 | 7.00E-02 | 2.85E-01 | -4.62 |
| CCNB1 |  | 0.103 | 8.546 | 1.816 | 7.00E-02 | 2.85E-01 | -4.621 |
| CHML |  | -0.105 | 7.287 | -1.816 | 7.00E-02 | 2.85E-01 | -4.622 |
| ZNF587 |  | -0.074 | 7.954 | -1.815 | 7.00E-02 | 2.86E-01 | -4.624 |
| UBE2N |  | 0.085 | 9.085 | 1.814 | 7.00E-02 | 2.86E-01 | -4.625 |
| PHLDA1 |  | -0.102 | 7.287 | -1.814 | 7.00E-02 | 2.86E-01 | -4.626 |
| CHRNA4 |  | -0.11 | 6.32 | -1.814 | 7.00E-02 | 2.86E-01 | -4.626 |
| TARSL2 |  | 0.106 | 6.463 | 1.813 | 7.00E-02 | 2.86E-01 | -4.626 |
| UST |  | -0.11 | 6.745 | -1.814 | 7.00E-02 | 2.86E-01 | -4.626 |
| TOMM20 |  | 0.076 | 10.565 | 1.812 | 7.10E-02 | 2.86E-01 | -4.627 |
| FAM46A |  | -0.111 | 11.859 | -1.813 | 7.10E-02 | 2.86E-01 | -4.628 |
| RNF13 |  | -0.086 | 9.74 | -1.812 | 7.10E-02 | 2.86E-01 | -4.629 |
| EIF4E |  | 0.06 | 7.841 | 1.811 | 7.10E-02 | 2.86E-01 | -4.629 |
| ZDHHC4 |  | -0.057 | 8.738 | -1.811 | 7.10E-02 | 2.86E-01 | -4.63 |
| FIBCD1 |  | -0.113 | 7.851 | -1.811 | 7.10E-02 | 2.86E-01 | -4.63 |
| KLF3 |  | -0.09 | 8.736 | -1.811 | 7.10E-02 | 2.86E-01 | -4.631 |
| ZCCHC2 |  | 0.089 | 7.771 | 1.81 | 7.10E-02 | 2.86E-01 | -4.631 |
| VPS39 |  | -0.074 | 8.557 | -1.811 | 7.10E-02 | 2.86E-01 | -4.631 |
| TM9SF4 |  | -0.063 | 10.216 | -1.81 | 7.10E-02 | 2.86E-01 | -4.632 |
| ZNF614 |  | 0.097 | 7.381 | 1.808 | 7.10E-02 | 2.87E-01 | -4.635 |
| RTEL1 |  | 0.078 | 8.483 | 1.807 | 7.10E-02 | 2.87E-01 | -4.636 |
| RPS19 |  | 0.059 | 13.994 | 1.807 | 7.10E-02 | 2.87E-01 | -4.637 |
| SLC12A5 |  | -0.09 | 5.022 | -1.807 | 7.10E-02 | 2.87E-01 | -4.637 |
| LTB4R2 |  | -0.085 | 6.393 | -1.807 | 7.10E-02 | 2.87E-01 | -4.637 |
| EPS15L1 |  | -0.074 | 7.584 | -1.807 | 7.10E-02 | 2.87E-01 | -4.638 |
| ZNF234 |  | -0.093 | 7.849 | -1.807 | 7.20E-02 | 2.87E-01 | -4.638 |
| WBSCR22 |  | 0.083 | 8.986 | 1.806 | 7.20E-02 | 2.87E-01 | -4.638 |
| KIF5B |  | -0.094 | 9.354 | -1.806 | 7.20E-02 | 2.87E-01 | -4.639 |
| CGA |  | 0.144 | 10.789 | 1.805 | 7.20E-02 | 2.87E-01 | -4.639 |
| GLIS2 |  | -0.111 | 7.483 | -1.805 | 7.20E-02 | 2.87E-01 | -4.641 |
| IQCB1 |  | -0.07 | 8.158 | -1.805 | 7.20E-02 | 2.88E-01 | -4.641 |
| GUCA1B |  | -0.094 | 5.749 | -1.805 | 7.20E-02 | 2.88E-01 | -4.642 |
| STAT2 |  | 0.083 | 9.794 | 1.803 | 7.20E-02 | 2.88E-01 | -4.643 |
| RB1CC1 |  | 0.085 | 9.703 | 1.803 | 7.20E-02 | 2.88E-01 | -4.643 |
| SUFU |  | -0.095 | 6.534 | -1.803 | 7.20E-02 | 2.88E-01 | -4.645 |
| LAMA3 |  | 0.137 | 7.246 | 1.802 | 7.20E-02 | 2.88E-01 | -4.645 |
| SULF1 |  | 0.158 | 6.97 | 1.802 | 7.20E-02 | 2.88E-01 | -4.645 |
| PTGFRN |  | 0.131 | 7.414 | 1.801 | 7.20E-02 | 2.88E-01 | -4.646 |
| AP4S1 |  | 0.146 | 6.749 | 1.8 | 7.20E-02 | 2.89E-01 | -4.648 |
| RNF168 |  | 0.093 | 6.309 | 1.799 | 7.30E-02 | 2.89E-01 | -4.65 |
| P2RY6 |  | -0.111 | 7.887 | -1.799 | 7.30E-02 | 2.90E-01 | -4.651 |
| BRMS1L |  | -0.09 | 7.178 | -1.799 | 7.30E-02 | 2.90E-01 | -4.652 |
| ATAD3B |  | 0.08 | 7.819 | 1.797 | 7.30E-02 | 2.90E-01 | -4.653 |
| TEX101 |  | -0.12 | 5.328 | -1.797 | 7.30E-02 | 2.90E-01 | -4.655 |
| GPHA2 |  | -0.107 | 5.699 | -1.796 | 7.30E-02 | 2.91E-01 | -4.656 |
| CBX5 |  | -0.069 | 8.503 | -1.795 | 7.30E-02 | 2.91E-01 | -4.659 |
| NGEF |  | 0.114 | 5.542 | 1.794 | 7.30E-02 | 2.91E-01 | -4.659 |
| GBX2 |  | 0.122 | 6.004 | 1.793 | 7.40E-02 | 2.92E-01 | -4.66 |
| CABP7 |  | 0.144 | 5.632 | 1.791 | 7.40E-02 | 2.92E-01 | -4.663 |
| SLCO3A1 |  | -0.082 | 6.569 | -1.792 | 7.40E-02 | 2.92E-01 | -4.663 |
| DDB1 |  | 0.086 | 10.594 | 1.79 | 7.40E-02 | 2.93E-01 | -4.665 |
| THUMPD2 |  | 0.068 | 7.54 | 1.789 | 7.40E-02 | 2.93E-01 | -4.666 |
| ABCD1 |  | 0.094 | 7.801 | 1.789 | 7.40E-02 | 2.93E-01 | -4.666 |
| NDUFA8 |  | 0.063 | 11.665 | 1.789 | 7.40E-02 | 2.93E-01 | -4.667 |
| CEL |  | 0.088 | 6.282 | 1.789 | 7.40E-02 | 2.93E-01 | -4.667 |
| DRG1 |  | -0.076 | 10.311 | -1.789 | 7.40E-02 | 2.94E-01 | -4.669 |
| LTBP1 |  | 0.127 | 8.328 | 1.786 | 7.50E-02 | 2.94E-01 | -4.671 |
| PHGDH |  | 0.127 | 9.605 | 1.786 | 7.50E-02 | 2.94E-01 | -4.671 |
| ASF1B |  | -0.098 | 8.36 | -1.786 | 7.50E-02 | 2.95E-01 | -4.674 |
| SEC23B |  | -0.066 | 9.308 | -1.785 | 7.50E-02 | 2.96E-01 | -4.676 |
| KRTAP4-4 |  | -0.083 | 4.721 | -1.784 | 7.50E-02 | 2.96E-01 | -4.677 |
| COPG2 |  | 0.093 | 7.561 | 1.783 | 7.50E-02 | 2.96E-01 | -4.678 |
| NRD1 |  | 0.062 | 11.045 | 1.781 | 7.50E-02 | 2.96E-01 | -4.68 |
| LZTFL1 |  | 0.094 | 7.563 | 1.781 | 7.50E-02 | 2.96E-01 | -4.68 |
| PCK2 |  | -0.091 | 7.714 | -1.782 | 7.60E-02 | 2.96E-01 | -4.681 |
| ABHD10 |  | 0.075 | 7.074 | 1.78 | 7.60E-02 | 2.97E-01 | -4.682 |
| GEM |  | 0.145 | 6.526 | 1.78 | 7.60E-02 | 2.97E-01 | -4.682 |
| DEAF1 |  | 0.062 | 8.39 | 1.778 | 7.60E-02 | 2.98E-01 | -4.686 |
| COL5A1 |  | 0.108 | 9.195 | 1.777 | 7.60E-02 | 2.98E-01 | -4.687 |
| ACTR6 |  | 0.092 | 9.188 | 1.777 | 7.60E-02 | 2.98E-01 | -4.688 |
| ASCC1 |  | -0.092 | 8.054 | -1.775 | 7.70E-02 | 3.00E-01 | -4.691 |
| DDAH1 |  | 0.116 | 7.718 | 1.774 | 7.70E-02 | 3.00E-01 | -4.692 |
| SLC4A8 |  | 0.092 | 5.468 | 1.774 | 7.70E-02 | 3.00E-01 | -4.693 |
| RAB2B |  | -0.103 | 8.752 | -1.774 | 7.70E-02 | 3.00E-01 | -4.693 |
| AUH |  | -0.079 | 9.275 | -1.774 | 7.70E-02 | 3.00E-01 | -4.694 |
| CP |  | 0.162 | 5.663 | 1.772 | 7.70E-02 | 3.00E-01 | -4.695 |
| CSTF3 |  | 0.052 | 8.576 | 1.772 | 7.70E-02 | 3.00E-01 | -4.696 |
| SFI1 |  | 0.072 | 7.957 | 1.771 | 7.70E-02 | 3.00E-01 | -4.697 |
| CDH4 |  | 0.112 | 5.11 | 1.771 | 7.70E-02 | 3.00E-01 | -4.697 |
| LRRTM2 |  | 0.1 | 5.246 | 1.771 | 7.70E-02 | 3.00E-01 | -4.698 |
| TGDS |  | 0.072 | 8.609 | 1.771 | 7.70E-02 | 3.00E-01 | -4.698 |
| GTF2IRD1 |  | -0.081 | 7.006 | -1.771 | 7.70E-02 | 3.01E-01 | -4.699 |
| ZNF510 |  | 0.071 | 5.714 | 1.77 | 7.70E-02 | 3.01E-01 | -4.7 |
| FTL |  | -0.07 | 14.275 | -1.77 | 7.80E-02 | 3.01E-01 | -4.701 |
| SPATA13 |  | -0.117 | 7.374 | -1.77 | 7.80E-02 | 3.01E-01 | -4.701 |
| SNRPE |  | 0.077 | 8.987 | 1.768 | 7.80E-02 | 3.01E-01 | -4.702 |
| FKRP |  | -0.091 | 7.827 | -1.769 | 7.80E-02 | 3.01E-01 | -4.703 |
| SLC6A13 |  | 0.07 | 6.202 | 1.768 | 7.80E-02 | 3.01E-01 | -4.703 |
| PON1 |  | -0.094 | 4.963 | -1.769 | 7.80E-02 | 3.01E-01 | -4.703 |
| PTPRF |  | 0.117 | 9.662 | 1.767 | 7.80E-02 | 3.02E-01 | -4.705 |
| PECR |  | 0.088 | 6.943 | 1.766 | 7.80E-02 | 3.02E-01 | -4.706 |
| CLDN15 |  | 0.085 | 6.895 | 1.765 | 7.80E-02 | 3.02E-01 | -4.707 |
| METRNL |  | 0.095 | 10.761 | 1.765 | 7.80E-02 | 3.02E-01 | -4.707 |
| GRM4 |  | 0.13 | 5.304 | 1.764 | 7.80E-02 | 3.03E-01 | -4.709 |
| HMGB2 |  | 0.125 | 9.557 | 1.762 | 7.90E-02 | 3.03E-01 | -4.712 |
| SIRPB1 |  | -0.097 | 6.205 | -1.763 | 7.90E-02 | 3.04E-01 | -4.713 |
| C6 |  | -0.136 | 5.371 | -1.762 | 7.90E-02 | 3.04E-01 | -4.715 |
| PPM1D |  | -0.088 | 8.906 | -1.762 | 7.90E-02 | 3.04E-01 | -4.715 |
| BAHD1 |  | 0.057 | 8.979 | 1.76 | 7.90E-02 | 3.04E-01 | -4.716 |
| EYA1 |  | -0.087 | 5.089 | -1.76 | 7.90E-02 | 3.05E-01 | -4.717 |
| MRPL22 |  | 0.076 | 10.337 | 1.759 | 7.90E-02 | 3.05E-01 | -4.718 |
| NUP214 |  | -0.091 | 8.566 | -1.759 | 7.90E-02 | 3.05E-01 | -4.719 |
| ITGB2 |  | -0.098 | 9.354 | -1.759 | 7.90E-02 | 3.05E-01 | -4.719 |
| TSHB |  | -0.093 | 5.797 | -1.758 | 7.90E-02 | 3.05E-01 | -4.72 |
| PRPS1 |  | -0.083 | 8.705 | -1.757 | 8.00E-02 | 3.06E-01 | -4.722 |
| RRM1 |  | 0.07 | 10.457 | 1.756 | 8.00E-02 | 3.06E-01 | -4.722 |
| SLC6A1 |  | 0.099 | 5.193 | 1.755 | 8.00E-02 | 3.06E-01 | -4.725 |
| GLS |  | 0.086 | 8.176 | 1.754 | 8.00E-02 | 3.07E-01 | -4.726 |
| NIPSNAP3B |  | -0.102 | 6.352 | -1.754 | 8.00E-02 | 3.07E-01 | -4.727 |
| PTP4A2 |  | 0.071 | 9.3 | 1.753 | 8.00E-02 | 3.07E-01 | -4.727 |
| RER1 |  | 0.055 | 9.872 | 1.752 | 8.00E-02 | 3.08E-01 | -4.73 |
| PIR |  | -0.134 | 8.711 | -1.751 | 8.10E-02 | 3.09E-01 | -4.732 |
| TIGD7 |  | 0.106 | 6.612 | 1.75 | 8.10E-02 | 3.09E-01 | -4.733 |
| NES |  | -0.109 | 8.173 | -1.751 | 8.10E-02 | 3.09E-01 | -4.733 |
| ARL1 |  | 0.081 | 9.609 | 1.749 | 8.10E-02 | 3.09E-01 | -4.734 |
| ALDH5A1 |  | -0.112 | 6.928 | -1.75 | 8.10E-02 | 3.09E-01 | -4.734 |
| RARRES1 |  | 0.152 | 6.488 | 1.748 | 8.10E-02 | 3.09E-01 | -4.735 |
| LGMN |  | -0.104 | 10.457 | -1.749 | 8.10E-02 | 3.09E-01 | -4.735 |
| ACP2 |  | 0.089 | 8.477 | 1.747 | 8.10E-02 | 3.09E-01 | -4.737 |
| UNC13D |  | -0.12 | 7.613 | -1.748 | 8.10E-02 | 3.09E-01 | -4.737 |
| CDC42EP5 |  | -0.095 | 10.702 | -1.748 | 8.10E-02 | 3.09E-01 | -4.737 |
| ATRX |  | -0.064 | 7.557 | -1.747 | 8.10E-02 | 3.09E-01 | -4.739 |
| TUBG1 |  | -0.054 | 9.174 | -1.746 | 8.20E-02 | 3.09E-01 | -4.741 |
| PLA2G2F |  | 0.086 | 5.492 | 1.745 | 8.20E-02 | 3.09E-01 | -4.741 |
| STX1A |  | 0.093 | 7.47 | 1.745 | 8.20E-02 | 3.09E-01 | -4.741 |
| TRAM2 |  | 0.083 | 10.12 | 1.745 | 8.20E-02 | 3.09E-01 | -4.741 |
| KCNN2 |  | -0.125 | 5.68 | -1.746 | 8.20E-02 | 3.09E-01 | -4.742 |
| EDN3 |  | 0.107 | 5.009 | 1.744 | 8.20E-02 | 3.09E-01 | -4.742 |
| MINPP1 |  | -0.122 | 8.641 | -1.745 | 8.20E-02 | 3.09E-01 | -4.742 |
| EGF |  | -0.117 | 5.012 | -1.745 | 8.20E-02 | 3.09E-01 | -4.742 |
| EPHA2 |  | 0.097 | 7.703 | 1.743 | 8.20E-02 | 3.10E-01 | -4.743 |
| NDUFV1 |  | 0.065 | 10.961 | 1.743 | 8.20E-02 | 3.10E-01 | -4.744 |
| CLIC6 |  | -0.115 | 5.141 | -1.743 | 8.20E-02 | 3.10E-01 | -4.745 |
| CD79A |  | -0.115 | 7.933 | -1.743 | 8.20E-02 | 3.10E-01 | -4.746 |
| LAMP1 |  | 0.104 | 12.333 | 1.742 | 8.20E-02 | 3.10E-01 | -4.746 |
| ZNF253 |  | -0.084 | 7.371 | -1.743 | 8.20E-02 | 3.10E-01 | -4.746 |
| SH3BGRL |  | -0.102 | 9.81 | -1.743 | 8.20E-02 | 3.10E-01 | -4.747 |
| PTGER2 |  | -0.102 | 7.713 | -1.742 | 8.20E-02 | 3.10E-01 | -4.747 |
| PARP10 |  | 0.075 | 8.326 | 1.741 | 8.20E-02 | 3.10E-01 | -4.747 |
| ATP1B1 |  | -0.102 | 9.525 | -1.742 | 8.20E-02 | 3.10E-01 | -4.747 |
| DUSP3 |  | -0.074 | 9.884 | -1.742 | 8.20E-02 | 3.10E-01 | -4.748 |
| GSTO1 |  | -0.103 | 11.881 | -1.741 | 8.30E-02 | 3.10E-01 | -4.749 |
| ADCY8 |  | -0.091 | 4.531 | -1.74 | 8.30E-02 | 3.11E-01 | -4.751 |
| PLAG1 |  | 0.088 | 7.829 | 1.738 | 8.30E-02 | 3.11E-01 | -4.752 |
| TMEM35 |  | 0.119 | 5.585 | 1.738 | 8.30E-02 | 3.11E-01 | -4.752 |
| RPS4X |  | -0.079 | 13.173 | -1.739 | 8.30E-02 | 3.11E-01 | -4.753 |
| PRSS21 |  | -0.097 | 5.754 | -1.738 | 8.30E-02 | 3.11E-01 | -4.755 |
| NUP62 |  | -0.057 | 10.567 | -1.737 | 8.30E-02 | 3.11E-01 | -4.755 |
| TRIM63 |  | -0.133 | 5.749 | -1.737 | 8.30E-02 | 3.11E-01 | -4.755 |
| ECEL1 |  | -0.105 | 5.14 | -1.736 | 8.30E-02 | 3.12E-01 | -4.757 |
| TMSB10 |  | 0.07 | 13.882 | 1.735 | 8.30E-02 | 3.12E-01 | -4.757 |
| ADAMTS19 |  | 0.106 | 6.285 | 1.734 | 8.40E-02 | 3.12E-01 | -4.759 |
| HBP1 |  | -0.096 | 9.556 | -1.734 | 8.40E-02 | 3.12E-01 | -4.76 |
| PEX11G |  | 0.113 | 6.345 | 1.733 | 8.40E-02 | 3.12E-01 | -4.76 |
| CPNE8 |  | 0.092 | 7.489 | 1.733 | 8.40E-02 | 3.12E-01 | -4.761 |
| LEPROTL1 |  | 0.097 | 10.502 | 1.732 | 8.40E-02 | 3.12E-01 | -4.762 |
| EDIL3 |  | 0.126 | 6.102 | 1.732 | 8.40E-02 | 3.12E-01 | -4.762 |
| DDX31 |  | -0.078 | 6.172 | -1.733 | 8.40E-02 | 3.12E-01 | -4.762 |
| RENBP |  | -0.114 | 6.969 | -1.733 | 8.40E-02 | 3.12E-01 | -4.762 |
| PDE9A |  | 0.089 | 7.007 | 1.731 | 8.40E-02 | 3.13E-01 | -4.764 |
| SLC1A2 |  | -0.133 | 6.241 | -1.731 | 8.40E-02 | 3.13E-01 | -4.765 |
| TFPI |  | 0.12 | 10.588 | 1.73 | 8.40E-02 | 3.13E-01 | -4.765 |
| IL9 |  | -0.085 | 4.766 | -1.731 | 8.40E-02 | 3.13E-01 | -4.766 |
| GNB1 |  | -0.071 | 11.681 | -1.73 | 8.40E-02 | 3.13E-01 | -4.767 |
| HACE1 |  | -0.089 | 8.214 | -1.729 | 8.50E-02 | 3.14E-01 | -4.769 |
| PLCB4 |  | 0.105 | 6.024 | 1.728 | 8.50E-02 | 3.14E-01 | -4.769 |
| KPTN |  | 0.084 | 7.161 | 1.727 | 8.50E-02 | 3.14E-01 | -4.77 |
| KLHDC4 |  | -0.069 | 7.635 | -1.727 | 8.50E-02 | 3.15E-01 | -4.772 |
| BOLL |  | 0.067 | 5.166 | 1.726 | 8.50E-02 | 3.15E-01 | -4.772 |
| MRPL49 |  | 0.096 | 9.498 | 1.726 | 8.50E-02 | 3.15E-01 | -4.773 |
| COL4A5 |  | 0.109 | 7.904 | 1.725 | 8.50E-02 | 3.15E-01 | -4.774 |
| GUCA2B |  | 0.122 | 5.203 | 1.725 | 8.50E-02 | 3.15E-01 | -4.774 |
| BCAS4 |  | 0.079 | 7.54 | 1.724 | 8.50E-02 | 3.15E-01 | -4.775 |
| SLC22A15 |  | 0.094 | 6.704 | 1.724 | 8.50E-02 | 3.15E-01 | -4.775 |
| SNX5 |  | 0.059 | 9.897 | 1.721 | 8.60E-02 | 3.18E-01 | -4.781 |
| CYP26B1 |  | 0.131 | 6.472 | 1.72 | 8.60E-02 | 3.18E-01 | -4.782 |
| SHFM1 |  | 0.07 | 11.156 | 1.719 | 8.60E-02 | 3.18E-01 | -4.784 |
| ETHE1 |  | 0.074 | 8.834 | 1.719 | 8.60E-02 | 3.18E-01 | -4.784 |
| RABL3 |  | -0.074 | 7.74 | -1.719 | 8.60E-02 | 3.19E-01 | -4.786 |
| TOMM40 |  | 0.075 | 8.946 | 1.717 | 8.70E-02 | 3.19E-01 | -4.787 |
| PPP4R1L |  | -0.102 | 5.58 | -1.718 | 8.70E-02 | 3.19E-01 | -4.788 |
| PRDM2 |  | -0.043 | 7.687 | -1.714 | 8.70E-02 | 3.21E-01 | -4.793 |
| IVD |  | -0.08 | 7.659 | -1.714 | 8.70E-02 | 3.21E-01 | -4.794 |
| TXNIP |  | -0.091 | 12.867 | -1.713 | 8.80E-02 | 3.22E-01 | -4.795 |
| CSMD1 |  | -0.122 | 5.73 | -1.712 | 8.80E-02 | 3.22E-01 | -4.796 |
| UPF3B |  | 0.079 | 8.1 | 1.711 | 8.80E-02 | 3.22E-01 | -4.797 |
| CACNB2 |  | -0.1 | 4.916 | -1.711 | 8.80E-02 | 3.23E-01 | -4.798 |
| PABPN1 |  | 0.054 | 10.699 | 1.709 | 8.80E-02 | 3.23E-01 | -4.8 |
| MUC7 |  | -0.105 | 5.056 | -1.71 | 8.80E-02 | 3.23E-01 | -4.8 |
| PGR |  | 0.113 | 5.835 | 1.708 | 8.80E-02 | 3.23E-01 | -4.802 |
| PPL |  | 0.138 | 7.601 | 1.704 | 8.90E-02 | 3.26E-01 | -4.809 |
| TSPYL5 |  | 0.107 | 7.108 | 1.703 | 8.90E-02 | 3.27E-01 | -4.81 |
| SPTBN5 |  | 0.115 | 5.701 | 1.702 | 8.90E-02 | 3.27E-01 | -4.811 |
| CTTNBP2 |  | 0.094 | 5.726 | 1.702 | 8.90E-02 | 3.27E-01 | -4.812 |
| YKT6 |  | 0.054 | 8.526 | 1.701 | 9.00E-02 | 3.27E-01 | -4.812 |
| LIN7A |  | -0.127 | 6.945 | -1.701 | 9.00E-02 | 3.27E-01 | -4.814 |
| KLK13 |  | -0.069 | 4.988 | -1.701 | 9.00E-02 | 3.27E-01 | -4.814 |
| PLCG2 |  | -0.093 | 8.425 | -1.701 | 9.00E-02 | 3.27E-01 | -4.815 |
| SSTR4 |  | -0.104 | 5.27 | -1.701 | 9.00E-02 | 3.27E-01 | -4.815 |
| BCL9L |  | -0.089 | 7.59 | -1.7 | 9.00E-02 | 3.28E-01 | -4.816 |
| ALPP |  | -0.163 | 11.758 | -1.7 | 9.00E-02 | 3.28E-01 | -4.816 |
| PAX3 |  | -0.081 | 4.947 | -1.699 | 9.00E-02 | 3.28E-01 | -4.817 |
| TNFRSF10C |  | 0.094 | 7.673 | 1.698 | 9.00E-02 | 3.28E-01 | -4.818 |
| CLTC |  | -0.087 | 9.435 | -1.698 | 9.00E-02 | 3.28E-01 | -4.819 |
| RAMP3 |  | -0.154 | 6.459 | -1.698 | 9.00E-02 | 3.28E-01 | -4.82 |
| NFE2L2 |  | -0.075 | 9.57 | -1.698 | 9.00E-02 | 3.28E-01 | -4.82 |
| PTPRJ |  | -0.094 | 7.316 | -1.697 | 9.00E-02 | 3.29E-01 | -4.821 |
| PADI4 |  | -0.099 | 5.777 | -1.697 | 9.10E-02 | 3.29E-01 | -4.821 |
| FMOD |  | -0.117 | 8.182 | -1.696 | 9.10E-02 | 3.29E-01 | -4.822 |
| PLA2G7 |  | -0.178 | 7.851 | -1.696 | 9.10E-02 | 3.29E-01 | -4.823 |
| DCP2 |  | 0.09 | 9.646 | 1.695 | 9.10E-02 | 3.29E-01 | -4.823 |
| GPX5 |  | -0.109 | 5.898 | -1.696 | 9.10E-02 | 3.29E-01 | -4.823 |
| SNCG |  | -0.092 | 6.642 | -1.696 | 9.10E-02 | 3.29E-01 | -4.823 |
| TNFRSF11A |  | -0.096 | 5.925 | -1.695 | 9.10E-02 | 3.29E-01 | -4.824 |
| SYNGR3 |  | -0.132 | 8.052 | -1.695 | 9.10E-02 | 3.29E-01 | -4.825 |
| ANXA3 |  | 0.13 | 10.205 | 1.693 | 9.10E-02 | 3.29E-01 | -4.826 |
| OLIG1 |  | -0.106 | 6.031 | -1.694 | 9.10E-02 | 3.29E-01 | -4.826 |
| CDH19 |  | 0.091 | 6.022 | 1.693 | 9.10E-02 | 3.29E-01 | -4.826 |
| IKBKG |  | 0.091 | 8.951 | 1.693 | 9.10E-02 | 3.29E-01 | -4.826 |
| NID2 |  | -0.128 | 7.715 | -1.693 | 9.10E-02 | 3.29E-01 | -4.827 |
| IL5RA |  | -0.107 | 5.274 | -1.693 | 9.10E-02 | 3.29E-01 | -4.828 |
| DYRK2 |  | -0.079 | 8.103 | -1.693 | 9.10E-02 | 3.29E-01 | -4.828 |
| RAB33A |  | 0.095 | 6.844 | 1.691 | 9.10E-02 | 3.29E-01 | -4.828 |
| GLTSCR2 |  | 0.073 | 12.049 | 1.69 | 9.20E-02 | 3.30E-01 | -4.83 |
| SPP1 |  | -0.182 | 10.261 | -1.691 | 9.20E-02 | 3.30E-01 | -4.831 |
| BCKDHB |  | -0.107 | 6.935 | -1.69 | 9.20E-02 | 3.30E-01 | -4.832 |
| KCTD3 |  | -0.096 | 9.099 | -1.688 | 9.20E-02 | 3.31E-01 | -4.835 |
| PPP2R5D |  | 0.058 | 8.676 | 1.686 | 9.20E-02 | 3.32E-01 | -4.837 |
| PRR3 |  | 0.063 | 7.211 | 1.686 | 9.20E-02 | 3.32E-01 | -4.838 |
| UTS2R |  | -0.158 | 8.725 | -1.687 | 9.20E-02 | 3.32E-01 | -4.838 |
| IGF1 |  | -0.104 | 7.407 | -1.686 | 9.30E-02 | 3.32E-01 | -4.839 |
| TPM2 |  | 0.096 | 10.219 | 1.685 | 9.30E-02 | 3.32E-01 | -4.839 |
| POU1F1 |  | -0.08 | 4.626 | -1.686 | 9.30E-02 | 3.32E-01 | -4.839 |
| FTSJ2 |  | 0.051 | 8.273 | 1.683 | 9.30E-02 | 3.33E-01 | -4.842 |
| PPP2R5A |  | 0.085 | 9.086 | 1.683 | 9.30E-02 | 3.33E-01 | -4.843 |
| CAD |  | -0.075 | 8.188 | -1.683 | 9.30E-02 | 3.33E-01 | -4.843 |
| SPRED1 |  | -0.092 | 8.212 | -1.683 | 9.30E-02 | 3.33E-01 | -4.843 |
| PPP1R12A |  | 0.101 | 8.717 | 1.682 | 9.30E-02 | 3.33E-01 | -4.844 |
| ARID4A |  | 0.068 | 7.433 | 1.682 | 9.30E-02 | 3.33E-01 | -4.844 |
| GEMIN5 |  | -0.081 | 7.534 | -1.683 | 9.30E-02 | 3.33E-01 | -4.844 |
| UNC5D |  | -0.085 | 4.549 | -1.682 | 9.30E-02 | 3.33E-01 | -4.845 |
| SDK1 |  | 0.116 | 5.364 | 1.68 | 9.40E-02 | 3.33E-01 | -4.847 |
| NRL |  | 0.105 | 6.018 | 1.68 | 9.40E-02 | 3.33E-01 | -4.847 |
| BRF1 |  | -0.059 | 8.002 | -1.681 | 9.40E-02 | 3.33E-01 | -4.847 |
| FBLN5 |  | -0.098 | 8.696 | -1.681 | 9.40E-02 | 3.33E-01 | -4.848 |
| LZIC |  | 0.108 | 8.677 | 1.679 | 9.40E-02 | 3.33E-01 | -4.848 |
| TRADD |  | 0.056 | 9.552 | 1.679 | 9.40E-02 | 3.33E-01 | -4.848 |
| ABCF2 |  | -0.055 | 8.027 | -1.68 | 9.40E-02 | 3.33E-01 | -4.848 |
| PDCD6IP |  | -0.054 | 9.147 | -1.679 | 9.40E-02 | 3.34E-01 | -4.85 |
| GMEB2 |  | 0.094 | 8.408 | 1.677 | 9.40E-02 | 3.34E-01 | -4.851 |
| ZDHHC13 |  | -0.08 | 7.731 | -1.677 | 9.40E-02 | 3.35E-01 | -4.853 |
| MCCC2 |  | -0.077 | 7.666 | -1.677 | 9.40E-02 | 3.35E-01 | -4.854 |
| GIPR |  | -0.116 | 6.631 | -1.677 | 9.40E-02 | 3.35E-01 | -4.854 |
| C6orf165 |  | 0.088 | 4.961 | 1.675 | 9.40E-02 | 3.35E-01 | -4.854 |
| EGR1 |  | 0.184 | 9.552 | 1.675 | 9.50E-02 | 3.35E-01 | -4.856 |
| PLA2R1 |  | 0.084 | 5.66 | 1.673 | 9.50E-02 | 3.36E-01 | -4.857 |
| PTPRT |  | -0.106 | 5.744 | -1.674 | 9.50E-02 | 3.36E-01 | -4.857 |
| RAB7B |  | 0.085 | 6.43 | 1.672 | 9.50E-02 | 3.37E-01 | -4.86 |
| AMACR |  | -0.111 | 6.578 | -1.673 | 9.50E-02 | 3.37E-01 | -4.86 |
| FMO2 |  | 0.102 | 5.182 | 1.671 | 9.50E-02 | 3.37E-01 | -4.861 |
| KLF12 |  | 0.094 | 6.672 | 1.671 | 9.50E-02 | 3.37E-01 | -4.862 |
| SRI |  | 0.066 | 9.071 | 1.671 | 9.50E-02 | 3.37E-01 | -4.862 |
| E4F1 |  | 0.065 | 9.177 | 1.67 | 9.60E-02 | 3.37E-01 | -4.863 |
| SSH3 |  | -0.079 | 7.955 | -1.671 | 9.60E-02 | 3.37E-01 | -4.863 |
| CLCA1 |  | 0.102 | 4.862 | 1.669 | 9.60E-02 | 3.37E-01 | -4.864 |
| POLE3 |  | -0.087 | 10.05 | -1.67 | 9.60E-02 | 3.37E-01 | -4.864 |
| FAP |  | -0.125 | 8.189 | -1.67 | 9.60E-02 | 3.37E-01 | -4.864 |
| CYR61 |  | 0.111 | 10.2 | 1.668 | 9.60E-02 | 3.37E-01 | -4.866 |
| ERN1 |  | -0.065 | 6.897 | -1.669 | 9.60E-02 | 3.37E-01 | -4.866 |
| CUEDC1 |  | 0.09 | 7.814 | 1.668 | 9.60E-02 | 3.37E-01 | -4.866 |
| P2RY12 |  | -0.12 | 5.632 | -1.669 | 9.60E-02 | 3.37E-01 | -4.867 |
| PUS1 |  | 0.079 | 8.557 | 1.667 | 9.60E-02 | 3.37E-01 | -4.867 |
| TNFSF10 |  | 0.14 | 9.798 | 1.667 | 9.60E-02 | 3.37E-01 | -4.868 |
| AK7 |  | 0.101 | 4.988 | 1.665 | 9.70E-02 | 3.39E-01 | -4.871 |
| LILRB3 |  | -0.09 | 8.852 | -1.665 | 9.70E-02 | 3.39E-01 | -4.873 |
| PLCXD2 |  | -0.126 | 6.943 | -1.665 | 9.70E-02 | 3.39E-01 | -4.873 |
| PIGO |  | -0.084 | 8.481 | -1.664 | 9.70E-02 | 3.39E-01 | -4.874 |
| PYCR1 |  | 0.112 | 7.471 | 1.663 | 9.70E-02 | 3.39E-01 | -4.874 |
| TRAF4 |  | 0.065 | 7.9 | 1.663 | 9.70E-02 | 3.39E-01 | -4.874 |
| BMP4 |  | 0.111 | 7.221 | 1.662 | 9.70E-02 | 3.40E-01 | -4.875 |
| MLNR |  | 0.091 | 4.755 | 1.662 | 9.70E-02 | 3.40E-01 | -4.876 |
| PIK3CB |  | 0.102 | 7.754 | 1.661 | 9.70E-02 | 3.40E-01 | -4.877 |
| EXOSC2 |  | -0.06 | 7.739 | -1.662 | 9.70E-02 | 3.40E-01 | -4.877 |
| KCNAB1 |  | -0.099 | 5.188 | -1.662 | 9.70E-02 | 3.40E-01 | -4.877 |
| SLC29A1 |  | -0.098 | 9.019 | -1.661 | 9.80E-02 | 3.40E-01 | -4.879 |
| RUNDC1 |  | -0.081 | 8.025 | -1.661 | 9.80E-02 | 3.40E-01 | -4.879 |
| USP44 |  | -0.115 | 5.378 | -1.66 | 9.80E-02 | 3.40E-01 | -4.88 |
| ARID1B |  | -0.06 | 7.045 | -1.66 | 9.80E-02 | 3.41E-01 | -4.881 |
| BMP8A |  | 0.119 | 5.566 | 1.658 | 9.80E-02 | 3.41E-01 | -4.881 |
| CNP |  | -0.056 | 8.173 | -1.659 | 9.80E-02 | 3.41E-01 | -4.882 |
| WSB1 |  | -0.093 | 10.542 | -1.658 | 9.80E-02 | 3.41E-01 | -4.883 |
| EIF4A2 |  | 0.085 | 11.437 | 1.657 | 9.80E-02 | 3.41E-01 | -4.883 |
| NEK6 |  | 0.076 | 8.457 | 1.657 | 9.80E-02 | 3.41E-01 | -4.884 |
| HIST1H2AM |  | -0.088 | 7.856 | -1.657 | 9.80E-02 | 3.41E-01 | -4.884 |
| LILRA1 |  | -0.1 | 6.609 | -1.656 | 9.90E-02 | 3.42E-01 | -4.886 |
| RAB14 |  | 0.053 | 8.045 | 1.654 | 9.90E-02 | 3.43E-01 | -4.888 |
| XRN1 |  | 0.079 | 8.321 | 1.653 | 9.90E-02 | 3.43E-01 | -4.89 |
| MAPKAP1 |  | -0.066 | 8.329 | -1.653 | 9.90E-02 | 3.43E-01 | -4.891 |
| FBXO33 |  | 0.064 | 9.036 | 1.652 | 9.90E-02 | 3.43E-01 | -4.891 |
| PDLIM3 |  | -0.094 | 6.324 | -1.653 | 9.90E-02 | 3.44E-01 | -4.892 |
| PFKFB3 |  | -0.096 | 8.096 | -1.652 | 9.90E-02 | 3.44E-01 | -4.892 |
| FMR1 |  | -0.061 | 8.553 | -1.651 | 1.00E-01 | 3.44E-01 | -4.894 |
| R3HDML |  | -0.106 | 5.505 | -1.651 | 1.00E-01 | 3.44E-01 | -4.895 |
| RANBP17 |  | -0.1 | 5.923 | -1.65 | 1.00E-01 | 3.45E-01 | -4.896 |
| PNMT |  | 0.119 | 7.285 | 1.647 | 1.00E-01 | 3.46E-01 | -4.899 |
| MRPL1 |  | 0.082 | 8.461 | 1.647 | 1.00E-01 | 3.46E-01 | -4.9 |
| DHX29 |  | 0.071 | 9.326 | 1.646 | 1.00E-01 | 3.46E-01 | -4.9 |
| MB |  | 0.104 | 5.022 | 1.645 | 1.01E-01 | 3.47E-01 | -4.903 |
| RBBP8 |  | -0.075 | 7.937 | -1.645 | 1.01E-01 | 3.47E-01 | -4.904 |
| PDHA2 |  | 0.079 | 4.667 | 1.643 | 1.01E-01 | 3.48E-01 | -4.905 |
| PAK1 |  | -0.111 | 9.383 | -1.643 | 1.01E-01 | 3.48E-01 | -4.907 |
| PTPRG |  | 0.072 | 6.683 | 1.641 | 1.01E-01 | 3.49E-01 | -4.908 |
| USP12 |  | 0.094 | 7.184 | 1.641 | 1.01E-01 | 3.49E-01 | -4.909 |
| ROBO4 |  | -0.076 | 7.731 | -1.641 | 1.02E-01 | 3.49E-01 | -4.909 |
| MPPE1 |  | 0.056 | 8.308 | 1.64 | 1.02E-01 | 3.49E-01 | -4.91 |
| DCPS |  | -0.07 | 8.865 | -1.64 | 1.02E-01 | 3.49E-01 | -4.911 |
| PGLYRP1 |  | -0.127 | 6.555 | -1.64 | 1.02E-01 | 3.49E-01 | -4.911 |
| ACLY |  | -0.068 | 10.294 | -1.64 | 1.02E-01 | 3.49E-01 | -4.911 |
| CREB3 |  | 0.066 | 8.478 | 1.637 | 1.02E-01 | 3.51E-01 | -4.915 |
| USP4 |  | -0.047 | 9.266 | -1.637 | 1.02E-01 | 3.51E-01 | -4.917 |
| SLAMF8 |  | -0.116 | 7.204 | -1.636 | 1.03E-01 | 3.51E-01 | -4.917 |
| TIMM9 |  | -0.066 | 9.697 | -1.636 | 1.03E-01 | 3.51E-01 | -4.917 |
| STAP2 |  | -0.112 | 7.123 | -1.636 | 1.03E-01 | 3.52E-01 | -4.918 |
| KRTAP3-1 |  | -0.118 | 6.141 | -1.635 | 1.03E-01 | 3.52E-01 | -4.919 |
| PDZRN3 |  | -0.08 | 6.533 | -1.635 | 1.03E-01 | 3.52E-01 | -4.919 |
| BHLHB9 |  | 0.091 | 6.346 | 1.634 | 1.03E-01 | 3.52E-01 | -4.92 |
| PDILT |  | 0.111 | 4.772 | 1.634 | 1.03E-01 | 3.52E-01 | -4.92 |
| ANKRA2 |  | -0.066 | 8.705 | -1.634 | 1.03E-01 | 3.52E-01 | -4.92 |
| PSMB9 |  | -0.117 | 9.022 | -1.634 | 1.03E-01 | 3.52E-01 | -4.921 |
| VGLL4 |  | 0.06 | 8.848 | 1.633 | 1.03E-01 | 3.52E-01 | -4.921 |
| CCT2 |  | -0.069 | 11.557 | -1.633 | 1.03E-01 | 3.53E-01 | -4.923 |
| CLYBL |  | 0.077 | 5.849 | 1.631 | 1.03E-01 | 3.53E-01 | -4.924 |
| MYO18B |  | -0.098 | 4.864 | -1.632 | 1.04E-01 | 3.53E-01 | -4.924 |
| ROR1 |  | 0.077 | 6.253 | 1.631 | 1.04E-01 | 3.53E-01 | -4.924 |
| CBLL1 |  | -0.06 | 8.034 | -1.631 | 1.04E-01 | 3.53E-01 | -4.925 |
| NOX1 |  | -0.079 | 5.245 | -1.631 | 1.04E-01 | 3.53E-01 | -4.926 |
| GNL3 |  | 0.077 | 9.443 | 1.63 | 1.04E-01 | 3.53E-01 | -4.926 |
| SRMS |  | 0.103 | 5.438 | 1.628 | 1.04E-01 | 3.54E-01 | -4.929 |
| PPP1R12B |  | -0.075 | 7.509 | -1.629 | 1.04E-01 | 3.54E-01 | -4.929 |
| SGCG |  | 0.084 | 4.71 | 1.627 | 1.04E-01 | 3.54E-01 | -4.93 |
| RFXAP |  | -0.092 | 7.092 | -1.627 | 1.04E-01 | 3.54E-01 | -4.931 |
| TSGA10 |  | -0.093 | 6.059 | -1.627 | 1.05E-01 | 3.54E-01 | -4.931 |
| RNMT |  | 0.069 | 8.284 | 1.626 | 1.05E-01 | 3.55E-01 | -4.932 |
| B2M |  | 0.084 | 14.01 | 1.626 | 1.05E-01 | 3.55E-01 | -4.932 |
| SLC22A6 |  | -0.089 | 5.382 | -1.626 | 1.05E-01 | 3.55E-01 | -4.934 |
| ATE1 |  | -0.083 | 6.45 | -1.625 | 1.05E-01 | 3.55E-01 | -4.934 |
| WDR18 |  | 0.083 | 10.011 | 1.624 | 1.05E-01 | 3.55E-01 | -4.934 |
| C20orf27 |  | -0.076 | 8.916 | -1.625 | 1.05E-01 | 3.55E-01 | -4.935 |
| SLC12A1 |  | -0.087 | 4.909 | -1.625 | 1.05E-01 | 3.55E-01 | -4.935 |
| IK |  | 0.054 | 10.845 | 1.623 | 1.05E-01 | 3.55E-01 | -4.936 |
| AIM1L |  | -0.096 | 6.995 | -1.624 | 1.05E-01 | 3.55E-01 | -4.936 |
| LRRTM4 |  | -0.086 | 4.785 | -1.624 | 1.05E-01 | 3.55E-01 | -4.937 |
| SLC29A3 |  | -0.081 | 7.633 | -1.623 | 1.05E-01 | 3.55E-01 | -4.938 |
| CYP3A5 |  | -0.106 | 5.559 | -1.623 | 1.05E-01 | 3.55E-01 | -4.938 |
| SYT11 |  | 0.085 | 7.575 | 1.622 | 1.05E-01 | 3.55E-01 | -4.938 |
| CKM |  | 0.114 | 5.657 | 1.621 | 1.06E-01 | 3.56E-01 | -4.939 |
| CD6 |  | -0.106 | 6.617 | -1.622 | 1.06E-01 | 3.56E-01 | -4.94 |
| NOL8 |  | 0.079 | 9.213 | 1.619 | 1.06E-01 | 3.57E-01 | -4.943 |
| KCNIP2 |  | 0.08 | 5.404 | 1.618 | 1.06E-01 | 3.57E-01 | -4.945 |
| CREB3L4 |  | 0.05 | 7.936 | 1.618 | 1.06E-01 | 3.57E-01 | -4.945 |
| P2RY13 |  | -0.103 | 6.628 | -1.619 | 1.06E-01 | 3.57E-01 | -4.945 |
| ANXA1 |  | 0.099 | 13.476 | 1.617 | 1.06E-01 | 3.57E-01 | -4.945 |
| BUB1B |  | 0.122 | 6.763 | 1.616 | 1.07E-01 | 3.58E-01 | -4.947 |
| NCKIPSD |  | 0.068 | 7.212 | 1.616 | 1.07E-01 | 3.58E-01 | -4.947 |
| CPSF3 |  | 0.063 | 9.357 | 1.616 | 1.07E-01 | 3.58E-01 | -4.947 |
| PTGES |  | 0.133 | 7.943 | 1.615 | 1.07E-01 | 3.58E-01 | -4.948 |
| EYA2 |  | -0.088 | 6.192 | -1.616 | 1.07E-01 | 3.58E-01 | -4.949 |
| NR1H2 |  | 0.087 | 8.972 | 1.615 | 1.07E-01 | 3.58E-01 | -4.949 |
| SIM2 |  | -0.067 | 5.196 | -1.615 | 1.07E-01 | 3.58E-01 | -4.95 |
| HAGHL |  | 0.099 | 6.781 | 1.612 | 1.08E-01 | 3.60E-01 | -4.953 |
| CDCA7 |  | -0.09 | 7.636 | -1.612 | 1.08E-01 | 3.60E-01 | -4.954 |
| MC5R |  | -0.114 | 5.711 | -1.612 | 1.08E-01 | 3.60E-01 | -4.955 |
| BRPF3 |  | -0.082 | 7.608 | -1.612 | 1.08E-01 | 3.60E-01 | -4.955 |
| DDB2 |  | 0.063 | 8.414 | 1.611 | 1.08E-01 | 3.60E-01 | -4.955 |
| SCN7A |  | -0.079 | 5.756 | -1.611 | 1.08E-01 | 3.60E-01 | -4.956 |
| PFKFB4 |  | 0.115 | 9.124 | 1.61 | 1.08E-01 | 3.60E-01 | -4.956 |
| ADAM30 |  | 0.09 | 5.254 | 1.61 | 1.08E-01 | 3.60E-01 | -4.957 |
| ARIH1 |  | -0.056 | 8.267 | -1.611 | 1.08E-01 | 3.60E-01 | -4.957 |
| PCCA |  | 0.092 | 9.362 | 1.609 | 1.08E-01 | 3.60E-01 | -4.958 |
| COX7B |  | 0.081 | 10.925 | 1.609 | 1.08E-01 | 3.60E-01 | -4.958 |
| SPESP1 |  | -0.171 | 7.029 | -1.609 | 1.08E-01 | 3.61E-01 | -4.959 |
| CHD1 |  | 0.103 | 8.826 | 1.608 | 1.08E-01 | 3.61E-01 | -4.959 |
| MTNR1B |  | 0.094 | 4.809 | 1.607 | 1.09E-01 | 3.61E-01 | -4.961 |
| CD2AP |  | 0.088 | 7.735 | 1.607 | 1.09E-01 | 3.61E-01 | -4.962 |
| BIVM |  | 0.078 | 7.499 | 1.606 | 1.09E-01 | 3.61E-01 | -4.962 |
| PCDHGA8 |  | -0.079 | 6.047 | -1.607 | 1.09E-01 | 3.62E-01 | -4.963 |
| FJX1 |  | 0.122 | 8.102 | 1.605 | 1.09E-01 | 3.62E-01 | -4.964 |
| FKBPL |  | -0.084 | 7.012 | -1.606 | 1.09E-01 | 3.62E-01 | -4.964 |
| ANKRD11 |  | -0.062 | 8.464 | -1.605 | 1.09E-01 | 3.62E-01 | -4.966 |
| SOS1 |  | -0.09 | 6.617 | -1.605 | 1.09E-01 | 3.62E-01 | -4.966 |
| CD1D |  | -0.084 | 6 | -1.604 | 1.09E-01 | 3.62E-01 | -4.967 |
| BECN1 |  | 0.05 | 9.666 | 1.603 | 1.09E-01 | 3.62E-01 | -4.967 |
| ZNF513 |  | 0.067 | 7.89 | 1.603 | 1.10E-01 | 3.62E-01 | -4.967 |
| NR2C2 |  | -0.068 | 7.703 | -1.604 | 1.10E-01 | 3.62E-01 | -4.967 |
| TMIE |  | 0.105 | 5.47 | 1.603 | 1.10E-01 | 3.62E-01 | -4.968 |
| FUBP1 |  | -0.068 | 8.295 | -1.603 | 1.10E-01 | 3.63E-01 | -4.969 |
| HOXC10 |  | -0.087 | 5.116 | -1.602 | 1.10E-01 | 3.63E-01 | -4.97 |
| DHX9 |  | 0.076 | 8.853 | 1.599 | 1.10E-01 | 3.64E-01 | -4.973 |
| ATP5G1 |  | 0.063 | 10.197 | 1.599 | 1.11E-01 | 3.65E-01 | -4.974 |
| TFPI2 |  | -0.154 | 11.425 | -1.599 | 1.11E-01 | 3.65E-01 | -4.974 |
| CASQ2 |  | -0.101 | 4.848 | -1.599 | 1.11E-01 | 3.65E-01 | -4.975 |
| TRIM23 |  | -0.09 | 6.873 | -1.599 | 1.11E-01 | 3.65E-01 | -4.975 |
| PYGM |  | -0.105 | 6.587 | -1.597 | 1.11E-01 | 3.66E-01 | -4.978 |
| CACNG7 |  | -0.079 | 5.219 | -1.596 | 1.11E-01 | 3.66E-01 | -4.979 |
| L3MBTL2 |  | 0.057 | 7.582 | 1.595 | 1.11E-01 | 3.66E-01 | -4.98 |
| SESN2 |  | -0.062 | 8.036 | -1.596 | 1.11E-01 | 3.66E-01 | -4.98 |
| HMGN2 |  | -0.057 | 11.046 | -1.595 | 1.12E-01 | 3.67E-01 | -4.981 |
| SURF4 |  | 0.067 | 10.978 | 1.594 | 1.12E-01 | 3.67E-01 | -4.981 |
| COL5A3 |  | -0.112 | 6.913 | -1.595 | 1.12E-01 | 3.67E-01 | -4.982 |
| FBXO24 |  | 0.072 | 5.842 | 1.593 | 1.12E-01 | 3.67E-01 | -4.982 |
| COL9A1 |  | 0.091 | 5.363 | 1.593 | 1.12E-01 | 3.67E-01 | -4.983 |
| TBCC |  | -0.053 | 9.068 | -1.593 | 1.12E-01 | 3.67E-01 | -4.984 |
| TNFRSF13C |  | 0.08 | 6.395 | 1.592 | 1.12E-01 | 3.67E-01 | -4.985 |
| PCSK6 |  | 0.112 | 6.697 | 1.591 | 1.12E-01 | 3.68E-01 | -4.986 |
| SFPQ |  | -0.08 | 9.256 | -1.592 | 1.12E-01 | 3.68E-01 | -4.986 |
| RALA |  | 0.082 | 9.805 | 1.589 | 1.13E-01 | 3.69E-01 | -4.989 |
| CYP2B6 |  | 0.075 | 6.317 | 1.589 | 1.13E-01 | 3.69E-01 | -4.989 |
| CYHR1 |  | 0.063 | 7.89 | 1.588 | 1.13E-01 | 3.69E-01 | -4.99 |
| RNF139 |  | -0.065 | 8.709 | -1.589 | 1.13E-01 | 3.69E-01 | -4.99 |
| C14orf1 |  | -0.08 | 6.884 | -1.589 | 1.13E-01 | 3.69E-01 | -4.99 |
| THPO |  | -0.077 | 5.147 | -1.589 | 1.13E-01 | 3.69E-01 | -4.991 |
| PMF1 |  | -0.061 | 9.049 | -1.587 | 1.13E-01 | 3.70E-01 | -4.993 |
| NFAT5 |  | -0.078 | 9.425 | -1.587 | 1.13E-01 | 3.70E-01 | -4.993 |
| PAPOLA |  | 0.084 | 9.788 | 1.586 | 1.13E-01 | 3.70E-01 | -4.993 |
| H1FX |  | 0.079 | 9.519 | 1.585 | 1.13E-01 | 3.70E-01 | -4.994 |
| ST6GALNAC5 |  | -0.131 | 5.695 | -1.586 | 1.14E-01 | 3.70E-01 | -4.994 |
| NPHP1 |  | 0.071 | 6.164 | 1.584 | 1.14E-01 | 3.70E-01 | -4.996 |
| WDHD1 |  | 0.076 | 6.631 | 1.584 | 1.14E-01 | 3.70E-01 | -4.996 |
| TP53I3 |  | 0.09 | 8.373 | 1.584 | 1.14E-01 | 3.70E-01 | -4.996 |
| THAP9 |  | -0.076 | 5.902 | -1.585 | 1.14E-01 | 3.70E-01 | -4.997 |
| SERPINA12 |  | -0.107 | 5.121 | -1.583 | 1.14E-01 | 3.71E-01 | -4.999 |
| POLD3 |  | -0.056 | 8.19 | -1.583 | 1.14E-01 | 3.71E-01 | -5 |
| TAS2R1 |  | 0.07 | 4.903 | 1.582 | 1.14E-01 | 3.71E-01 | -5 |
| WDR5 |  | 0.054 | 7.709 | 1.581 | 1.15E-01 | 3.72E-01 | -5.001 |
| IGSF11 |  | 0.094 | 5.03 | 1.58 | 1.15E-01 | 3.72E-01 | -5.002 |
| CAPN10 |  | 0.062 | 6.756 | 1.58 | 1.15E-01 | 3.72E-01 | -5.002 |
| CSN1S1 |  | -0.102 | 5.297 | -1.581 | 1.15E-01 | 3.72E-01 | -5.002 |
| LACE1 |  | -0.077 | 6.437 | -1.58 | 1.15E-01 | 3.72E-01 | -5.003 |
| CA8 |  | -0.091 | 6.629 | -1.579 | 1.15E-01 | 3.73E-01 | -5.005 |
| MRPL17 |  | 0.061 | 9.725 | 1.578 | 1.15E-01 | 3.73E-01 | -5.006 |
| ITM2C |  | 0.089 | 10.044 | 1.577 | 1.15E-01 | 3.73E-01 | -5.006 |
| MCC |  | 0.1 | 5.875 | 1.576 | 1.16E-01 | 3.74E-01 | -5.008 |
| GADD45A |  | -0.129 | 9.222 | -1.577 | 1.16E-01 | 3.74E-01 | -5.008 |
| DECR1 |  | -0.097 | 9.553 | -1.576 | 1.16E-01 | 3.74E-01 | -5.009 |
| USP20 |  | 0.075 | 7.932 | 1.575 | 1.16E-01 | 3.74E-01 | -5.009 |
| RASAL1 |  | -0.113 | 6.241 | -1.576 | 1.16E-01 | 3.74E-01 | -5.01 |
| LEF1 |  | -0.093 | 7.478 | -1.576 | 1.16E-01 | 3.74E-01 | -5.01 |
| CD151 |  | 0.089 | 10.214 | 1.574 | 1.16E-01 | 3.74E-01 | -5.011 |
| AP4B1 |  | 0.062 | 7.896 | 1.574 | 1.16E-01 | 3.74E-01 | -5.012 |
| UBE2A |  | -0.06 | 10.542 | -1.575 | 1.16E-01 | 3.74E-01 | -5.012 |
| TPM4 |  | 0.068 | 10.7 | 1.573 | 1.16E-01 | 3.74E-01 | -5.012 |
| ARMC1 |  | 0.064 | 9.043 | 1.573 | 1.16E-01 | 3.74E-01 | -5.013 |
| HERC5 |  | 0.11 | 8.922 | 1.573 | 1.16E-01 | 3.74E-01 | -5.013 |
| NKIRAS1 |  | -0.1 | 7.4 | -1.574 | 1.16E-01 | 3.74E-01 | -5.013 |
| TAF11 |  | 0.068 | 8 | 1.572 | 1.17E-01 | 3.74E-01 | -5.014 |
| RASGRF2 |  | 0.104 | 6.152 | 1.572 | 1.17E-01 | 3.74E-01 | -5.014 |
| NOLC1 |  | -0.063 | 8.608 | -1.573 | 1.17E-01 | 3.74E-01 | -5.015 |
| DOK3 |  | -0.077 | 7.358 | -1.573 | 1.17E-01 | 3.74E-01 | -5.015 |
| KHDRBS2 |  | 0.117 | 5.282 | 1.571 | 1.17E-01 | 3.75E-01 | -5.016 |
| DCK |  | -0.091 | 7.806 | -1.571 | 1.17E-01 | 3.75E-01 | -5.017 |
| TRPS1 |  | 0.082 | 6.713 | 1.568 | 1.17E-01 | 3.76E-01 | -5.02 |
| SAMD3 |  | 0.089 | 5.799 | 1.567 | 1.18E-01 | 3.77E-01 | -5.021 |
| MESDC1 |  | -0.067 | 8.991 | -1.568 | 1.18E-01 | 3.77E-01 | -5.022 |
| TUSC5 |  | -0.091 | 5.19 | -1.568 | 1.18E-01 | 3.77E-01 | -5.022 |
| RECQL5 |  | 0.072 | 6.169 | 1.566 | 1.18E-01 | 3.77E-01 | -5.023 |
| MS4A6E |  | -0.088 | 5.236 | -1.566 | 1.18E-01 | 3.78E-01 | -5.024 |
| GNAI1 |  | -0.094 | 8.375 | -1.565 | 1.18E-01 | 3.79E-01 | -5.026 |
| TDRD7 |  | -0.063 | 9.054 | -1.565 | 1.19E-01 | 3.79E-01 | -5.027 |
| CORO7 |  | 0.065 | 8.803 | 1.563 | 1.19E-01 | 3.79E-01 | -5.028 |
| TMEM14A |  | 0.076 | 8.705 | 1.562 | 1.19E-01 | 3.79E-01 | -5.029 |
| DLGAP1 |  | 0.084 | 5.001 | 1.562 | 1.19E-01 | 3.79E-01 | -5.029 |
| GDF15 |  | -0.115 | 12.209 | -1.563 | 1.19E-01 | 3.79E-01 | -5.029 |
| RNF4 |  | -0.097 | 9.974 | -1.563 | 1.19E-01 | 3.79E-01 | -5.03 |
| ZNF335 |  | 0.064 | 8.073 | 1.561 | 1.19E-01 | 3.79E-01 | -5.03 |
| MMP26 |  | -0.081 | 4.709 | -1.562 | 1.19E-01 | 3.79E-01 | -5.03 |
| CASP8 |  | -0.081 | 6.939 | -1.562 | 1.19E-01 | 3.79E-01 | -5.031 |
| RPL36 |  | 0.065 | 12.032 | 1.56 | 1.19E-01 | 3.80E-01 | -5.032 |
| FUT10 |  | 0.084 | 6.197 | 1.56 | 1.19E-01 | 3.80E-01 | -5.033 |
| RYR2 |  | 0.095 | 5.399 | 1.56 | 1.19E-01 | 3.80E-01 | -5.033 |
| C8orf4 |  | 0.13 | 8.385 | 1.559 | 1.20E-01 | 3.80E-01 | -5.033 |
| CTGF |  | 0.15 | 10.324 | 1.558 | 1.20E-01 | 3.80E-01 | -5.035 |
| HIST3H3 |  | 0.06 | 9.428 | 1.557 | 1.20E-01 | 3.80E-01 | -5.036 |
| ARPC2 |  | 0.045 | 11.853 | 1.557 | 1.20E-01 | 3.80E-01 | -5.036 |
| EPHA3 |  | -0.079 | 5.627 | -1.558 | 1.20E-01 | 3.80E-01 | -5.036 |
| CACNA1I |  | -0.078 | 6.541 | -1.558 | 1.20E-01 | 3.80E-01 | -5.037 |
| SMAD9 |  | 0.076 | 6.751 | 1.557 | 1.20E-01 | 3.80E-01 | -5.037 |
| TAS2R5 |  | 0.083 | 5.65 | 1.554 | 1.21E-01 | 3.82E-01 | -5.04 |
| PPP1R10 |  | 0.058 | 9.235 | 1.554 | 1.21E-01 | 3.82E-01 | -5.041 |
| SPTA1 |  | -0.134 | 6.523 | -1.554 | 1.21E-01 | 3.82E-01 | -5.042 |
| CASP7 |  | 0.07 | 8.563 | 1.553 | 1.21E-01 | 3.82E-01 | -5.042 |
| NR5A2 |  | -0.075 | 5.328 | -1.554 | 1.21E-01 | 3.83E-01 | -5.043 |
| LUC7L |  | 0.057 | 9.016 | 1.551 | 1.21E-01 | 3.84E-01 | -5.045 |
| TSN |  | 0.075 | 8.12 | 1.551 | 1.22E-01 | 3.84E-01 | -5.046 |
| FAT4 |  | 0.087 | 6.092 | 1.55 | 1.22E-01 | 3.84E-01 | -5.047 |
| HIST1H2AC |  | -0.106 | 8.624 | -1.551 | 1.22E-01 | 3.84E-01 | -5.047 |
| MMP2 |  | 0.092 | 7.199 | 1.55 | 1.22E-01 | 3.84E-01 | -5.047 |
| GTPBP4 |  | 0.084 | 10.06 | 1.55 | 1.22E-01 | 3.84E-01 | -5.048 |
| PLCXD1 |  | -0.099 | 8 | -1.549 | 1.22E-01 | 3.85E-01 | -5.049 |
| TCL1B |  | 0.11 | 7.355 | 1.548 | 1.22E-01 | 3.85E-01 | -5.049 |
| ITGA6 |  | -0.084 | 9.327 | -1.548 | 1.22E-01 | 3.85E-01 | -5.052 |
| RAC1 |  | 0.044 | 10.505 | 1.547 | 1.23E-01 | 3.85E-01 | -5.052 |
| MAP3K5 |  | -0.092 | 8.534 | -1.547 | 1.23E-01 | 3.86E-01 | -5.052 |
| GARS |  | 0.076 | 9.599 | 1.546 | 1.23E-01 | 3.86E-01 | -5.053 |
| LPPR4 |  | 0.115 | 5.759 | 1.546 | 1.23E-01 | 3.86E-01 | -5.053 |
| CLEC4A |  | -0.087 | 7.6 | -1.547 | 1.23E-01 | 3.86E-01 | -5.053 |
| NPTXR |  | -0.1 | 5.688 | -1.546 | 1.23E-01 | 3.86E-01 | -5.054 |
| PCDHB6 |  | -0.087 | 4.797 | -1.546 | 1.23E-01 | 3.86E-01 | -5.054 |
| LRPAP1 |  | 0.068 | 10.416 | 1.545 | 1.23E-01 | 3.86E-01 | -5.055 |
| APBB1 |  | -0.082 | 7.152 | -1.545 | 1.23E-01 | 3.86E-01 | -5.055 |
| PHLDB2 |  | -0.088 | 8.679 | -1.545 | 1.23E-01 | 3.86E-01 | -5.056 |
| SEC23A |  | -0.074 | 8.669 | -1.544 | 1.23E-01 | 3.87E-01 | -5.057 |
| BCL2L11 |  | -0.063 | 6.961 | -1.544 | 1.23E-01 | 3.87E-01 | -5.058 |
| NSD1 |  | -0.049 | 7.355 | -1.544 | 1.24E-01 | 3.87E-01 | -5.058 |
| ELOVL6 |  | 0.084 | 6.772 | 1.542 | 1.24E-01 | 3.87E-01 | -5.059 |
| PIWIL2 |  | -0.084 | 5.351 | -1.542 | 1.24E-01 | 3.88E-01 | -5.061 |
| FBXO40 |  | -0.065 | 4.864 | -1.541 | 1.24E-01 | 3.88E-01 | -5.062 |
| GPR35 |  | -0.064 | 6.541 | -1.54 | 1.24E-01 | 3.89E-01 | -5.063 |
| KYNU |  | -0.129 | 8.65 | -1.539 | 1.25E-01 | 3.89E-01 | -5.065 |
| UBP1 |  | 0.068 | 9.295 | 1.537 | 1.25E-01 | 3.90E-01 | -5.066 |
| TJP2 |  | -0.074 | 8.643 | -1.538 | 1.25E-01 | 3.90E-01 | -5.067 |
| ZNF230 |  | -0.077 | 6.363 | -1.538 | 1.25E-01 | 3.90E-01 | -5.067 |
| IMPDH2 |  | 0.068 | 10.692 | 1.536 | 1.25E-01 | 3.90E-01 | -5.067 |
| DUOX2 |  | -0.1 | 5.191 | -1.537 | 1.25E-01 | 3.90E-01 | -5.067 |
| RPL38 |  | -0.053 | 14.506 | -1.537 | 1.25E-01 | 3.90E-01 | -5.067 |
| VPS33A |  | -0.057 | 8.53 | -1.537 | 1.25E-01 | 3.90E-01 | -5.068 |
| KLHL2 |  | -0.086 | 8.36 | -1.536 | 1.25E-01 | 3.90E-01 | -5.069 |
| TNNT1 |  | 0.143 | 6.924 | 1.535 | 1.25E-01 | 3.90E-01 | -5.069 |
| DDO |  | -0.094 | 5.623 | -1.535 | 1.26E-01 | 3.90E-01 | -5.07 |
| PEX14 |  | 0.07 | 9.465 | 1.534 | 1.26E-01 | 3.90E-01 | -5.07 |
| ARMC8 |  | -0.054 | 7.88 | -1.535 | 1.26E-01 | 3.90E-01 | -5.071 |
| FXYD7 |  | 0.084 | 6.094 | 1.534 | 1.26E-01 | 3.90E-01 | -5.071 |
| GPR12 |  | -0.103 | 6.184 | -1.535 | 1.26E-01 | 3.90E-01 | -5.071 |
| HIVEP2 |  | -0.091 | 8.474 | -1.534 | 1.26E-01 | 3.90E-01 | -5.071 |
| MYH13 |  | -0.1 | 5.399 | -1.534 | 1.26E-01 | 3.90E-01 | -5.072 |
| CTHRC1 |  | -0.119 | 8.894 | -1.534 | 1.26E-01 | 3.90E-01 | -5.072 |
| MAGEB1 |  | -0.097 | 5.288 | -1.533 | 1.26E-01 | 3.90E-01 | -5.073 |
| ANKMY1 |  | 0.075 | 6.138 | 1.532 | 1.26E-01 | 3.90E-01 | -5.073 |
| CACYBP |  | 0.057 | 8.189 | 1.531 | 1.26E-01 | 3.91E-01 | -5.075 |
| ARL11 |  | 0.077 | 6.003 | 1.53 | 1.27E-01 | 3.91E-01 | -5.076 |
| DPT |  | -0.122 | 7.655 | -1.531 | 1.27E-01 | 3.91E-01 | -5.077 |
| HIST1H4I |  | 0.099 | 6.942 | 1.53 | 1.27E-01 | 3.91E-01 | -5.077 |
| GPRC5D |  | -0.103 | 5.571 | -1.531 | 1.27E-01 | 3.91E-01 | -5.077 |
| SSTR1 |  | -0.142 | 6.222 | -1.53 | 1.27E-01 | 3.91E-01 | -5.078 |
| ADAMTSL2 |  | 0.093 | 6.219 | 1.529 | 1.27E-01 | 3.91E-01 | -5.078 |
| HDAC4 |  | 0.068 | 7.891 | 1.529 | 1.27E-01 | 3.92E-01 | -5.078 |
| ATP2A2 |  | -0.064 | 8.606 | -1.529 | 1.27E-01 | 3.92E-01 | -5.079 |
| CHRNA3 |  | 0.057 | 4.927 | 1.527 | 1.27E-01 | 3.92E-01 | -5.08 |
| EPAS1 |  | -0.086 | 10.9 | -1.528 | 1.27E-01 | 3.92E-01 | -5.081 |
| GPR174 |  | 0.087 | 5.019 | 1.527 | 1.27E-01 | 3.92E-01 | -5.081 |
| BIRC6 |  | 0.06 | 8.361 | 1.527 | 1.27E-01 | 3.92E-01 | -5.081 |
| NUDC |  | 0.068 | 10.371 | 1.527 | 1.27E-01 | 3.92E-01 | -5.081 |
| CHD5 |  | -0.071 | 4.871 | -1.527 | 1.28E-01 | 3.92E-01 | -5.082 |
| RRS1 |  | 0.071 | 9.163 | 1.525 | 1.28E-01 | 3.93E-01 | -5.084 |
| EXOSC9 |  | 0.074 | 9.368 | 1.524 | 1.28E-01 | 3.93E-01 | -5.085 |
| RHOV |  | -0.102 | 7.104 | -1.525 | 1.28E-01 | 3.94E-01 | -5.086 |
| MGAM |  | -0.084 | 5.942 | -1.523 | 1.29E-01 | 3.95E-01 | -5.089 |
| AKAP8L |  | 0.06 | 9.303 | 1.521 | 1.29E-01 | 3.95E-01 | -5.089 |
| CAV3 |  | -0.1 | 5.289 | -1.521 | 1.29E-01 | 3.96E-01 | -5.091 |
| MMP3 |  | 0.113 | 5.626 | 1.52 | 1.29E-01 | 3.96E-01 | -5.091 |
| MKKS |  | 0.054 | 8.377 | 1.52 | 1.29E-01 | 3.96E-01 | -5.092 |
| UGT2B15 |  | -0.093 | 5.575 | -1.521 | 1.29E-01 | 3.96E-01 | -5.092 |
| HILS1 |  | 0.1 | 5.033 | 1.519 | 1.29E-01 | 3.96E-01 | -5.092 |
| S100A1 |  | 0.111 | 5.505 | 1.518 | 1.30E-01 | 3.96E-01 | -5.094 |
| NMBR |  | -0.096 | 4.741 | -1.519 | 1.30E-01 | 3.96E-01 | -5.094 |
| SIDT1 |  | -0.092 | 6.083 | -1.518 | 1.30E-01 | 3.97E-01 | -5.095 |
| RAB9A |  | 0.073 | 10.515 | 1.517 | 1.30E-01 | 3.97E-01 | -5.095 |
| MYO1A |  | -0.101 | 6.006 | -1.518 | 1.30E-01 | 3.97E-01 | -5.095 |
| SERPIND1 |  | 0.102 | 5.677 | 1.516 | 1.30E-01 | 3.97E-01 | -5.096 |
| ADHFE1 |  | -0.1 | 8.919 | -1.517 | 1.30E-01 | 3.97E-01 | -5.096 |
| TCP10L |  | 0.079 | 5.746 | 1.515 | 1.30E-01 | 3.98E-01 | -5.098 |
| LPPR2 |  | -0.074 | 7.556 | -1.515 | 1.31E-01 | 3.98E-01 | -5.1 |
| CPSF6 |  | -0.074 | 7.806 | -1.515 | 1.31E-01 | 3.98E-01 | -5.1 |
| PHF2 |  | -0.047 | 8.876 | -1.515 | 1.31E-01 | 3.98E-01 | -5.1 |
| PSD2 |  | -0.094 | 4.841 | -1.514 | 1.31E-01 | 3.99E-01 | -5.102 |
| ZCCHC3 |  | -0.074 | 7.829 | -1.513 | 1.31E-01 | 3.99E-01 | -5.102 |
| PMFBP1 |  | 0.083 | 5.276 | 1.512 | 1.31E-01 | 3.99E-01 | -5.102 |
| FMNL2 |  | -0.073 | 8.393 | -1.513 | 1.31E-01 | 3.99E-01 | -5.103 |
| KHK |  | 0.08 | 5.926 | 1.511 | 1.31E-01 | 3.99E-01 | -5.103 |
| SOAT1 |  | -0.088 | 7.575 | -1.512 | 1.31E-01 | 3.99E-01 | -5.104 |
| PI3 |  | -0.123 | 6.621 | -1.512 | 1.31E-01 | 3.99E-01 | -5.104 |
| RAB24 |  | 0.056 | 9.309 | 1.511 | 1.31E-01 | 3.99E-01 | -5.104 |
| GPR160 |  | -0.094 | 7.406 | -1.512 | 1.31E-01 | 3.99E-01 | -5.105 |
| PPFIA1 |  | -0.063 | 8.572 | -1.511 | 1.32E-01 | 3.99E-01 | -5.105 |
| STAT5A |  | -0.096 | 8.287 | -1.511 | 1.32E-01 | 3.99E-01 | -5.105 |
| MSX1 |  | -0.094 | 7.717 | -1.511 | 1.32E-01 | 3.99E-01 | -5.106 |
| TTYH3 |  | -0.086 | 8.793 | -1.511 | 1.32E-01 | 3.99E-01 | -5.106 |
| SYT4 |  | 0.07 | 4.633 | 1.509 | 1.32E-01 | 3.99E-01 | -5.106 |
| CDH18 |  | 0.111 | 4.952 | 1.509 | 1.32E-01 | 3.99E-01 | -5.107 |
| NINJ2 |  | -0.11 | 7.268 | -1.51 | 1.32E-01 | 3.99E-01 | -5.107 |
| PROK1 |  | 0.14 | 6.313 | 1.508 | 1.32E-01 | 3.99E-01 | -5.108 |
| OR12D3 |  | 0.096 | 4.946 | 1.508 | 1.32E-01 | 3.99E-01 | -5.108 |
| PARVA |  | -0.068 | 8.371 | -1.509 | 1.32E-01 | 3.99E-01 | -5.108 |
| ZNF574 |  | -0.088 | 7.685 | -1.508 | 1.32E-01 | 4.00E-01 | -5.109 |
| EEF1A2 |  | 0.104 | 5.664 | 1.507 | 1.33E-01 | 4.00E-01 | -5.11 |
| PGLYRP2 |  | -0.078 | 4.891 | -1.507 | 1.33E-01 | 4.00E-01 | -5.11 |
| ADAMTS6 |  | -0.128 | 6.716 | -1.507 | 1.33E-01 | 4.00E-01 | -5.111 |
| RIOK3 |  | 0.081 | 9.797 | 1.505 | 1.33E-01 | 4.00E-01 | -5.112 |
| GDNF |  | 0.071 | 5.669 | 1.505 | 1.33E-01 | 4.00E-01 | -5.112 |
| ARAF |  | 0.069 | 8.748 | 1.505 | 1.33E-01 | 4.00E-01 | -5.112 |
| DALRD3 |  | 0.059 | 7.984 | 1.505 | 1.33E-01 | 4.00E-01 | -5.113 |
| TRIT1 |  | 0.083 | 9.066 | 1.505 | 1.33E-01 | 4.00E-01 | -5.113 |
| DHCR7 |  | 0.094 | 8.663 | 1.504 | 1.33E-01 | 4.01E-01 | -5.114 |
| NUDT2 |  | 0.068 | 8.849 | 1.504 | 1.33E-01 | 4.01E-01 | -5.114 |
| MINK1 |  | -0.103 | 6.86 | -1.504 | 1.33E-01 | 4.01E-01 | -5.116 |
| GPR37 |  | -0.08 | 6.665 | -1.503 | 1.34E-01 | 4.02E-01 | -5.117 |
| SOAT2 |  | 0.118 | 5.902 | 1.502 | 1.34E-01 | 4.02E-01 | -5.117 |
| TSLP |  | 0.085 | 4.766 | 1.5 | 1.34E-01 | 4.03E-01 | -5.119 |
| SAFB |  | 0.091 | 9.41 | 1.5 | 1.34E-01 | 4.03E-01 | -5.12 |
| CXCL5 |  | -0.148 | 6.677 | -1.501 | 1.34E-01 | 4.03E-01 | -5.12 |
| PSMA4 |  | -0.068 | 10.849 | -1.501 | 1.34E-01 | 4.03E-01 | -5.12 |
| FBXO38 |  | -0.054 | 8.798 | -1.5 | 1.35E-01 | 4.03E-01 | -5.122 |
| PLA2G5 |  | -0.097 | 7.964 | -1.499 | 1.35E-01 | 4.03E-01 | -5.122 |
| ASB2 |  | -0.156 | 7.305 | -1.499 | 1.35E-01 | 4.03E-01 | -5.123 |
| GPR161 |  | -0.072 | 6.441 | -1.499 | 1.35E-01 | 4.03E-01 | -5.123 |
| FXN |  | -0.073 | 6.87 | -1.497 | 1.35E-01 | 4.04E-01 | -5.125 |
| BTAF1 |  | -0.088 | 8.561 | -1.497 | 1.35E-01 | 4.04E-01 | -5.126 |
| C15orf27 |  | -0.105 | 6.234 | -1.497 | 1.35E-01 | 4.04E-01 | -5.126 |
| FOLR1 |  | 0.081 | 8.552 | 1.495 | 1.36E-01 | 4.05E-01 | -5.127 |
| IFNW1 |  | 0.079 | 4.813 | 1.495 | 1.36E-01 | 4.05E-01 | -5.127 |
| LHFP |  | -0.084 | 10.207 | -1.496 | 1.36E-01 | 4.05E-01 | -5.127 |
| ARL6IP6 |  | -0.074 | 9.574 | -1.495 | 1.36E-01 | 4.05E-01 | -5.129 |
| KCNG1 |  | -0.099 | 7.245 | -1.495 | 1.36E-01 | 4.05E-01 | -5.129 |
| PTGER1 |  | 0.092 | 5.336 | 1.494 | 1.36E-01 | 4.05E-01 | -5.129 |
| SGK2 |  | -0.08 | 5.583 | -1.493 | 1.36E-01 | 4.06E-01 | -5.131 |
| TFEB |  | 0.078 | 8.459 | 1.492 | 1.36E-01 | 4.06E-01 | -5.132 |
| ZNF77 |  | -0.062 | 7.574 | -1.492 | 1.37E-01 | 4.07E-01 | -5.133 |
| IRX1 |  | -0.102 | 5.813 | -1.491 | 1.37E-01 | 4.07E-01 | -5.133 |
| ASB6 |  | -0.054 | 7.57 | -1.491 | 1.37E-01 | 4.07E-01 | -5.133 |
| HSPA5 |  | 0.066 | 10.685 | 1.49 | 1.37E-01 | 4.07E-01 | -5.134 |
| GRID2 |  | -0.087 | 5.218 | -1.491 | 1.37E-01 | 4.07E-01 | -5.134 |
| PRKAR2A |  | 0.073 | 7.107 | 1.49 | 1.37E-01 | 4.07E-01 | -5.135 |
| DHFRL1 |  | 0.074 | 6.871 | 1.488 | 1.37E-01 | 4.07E-01 | -5.137 |
| BID |  | -0.068 | 8.782 | -1.489 | 1.37E-01 | 4.07E-01 | -5.137 |
| SNX13 |  | -0.077 | 8.439 | -1.489 | 1.37E-01 | 4.07E-01 | -5.138 |
| MBD2 |  | 0.056 | 8.61 | 1.488 | 1.37E-01 | 4.07E-01 | -5.138 |
| FRZB |  | -0.134 | 8.851 | -1.488 | 1.37E-01 | 4.07E-01 | -5.138 |
| SRRM2 |  | 0.069 | 9.775 | 1.487 | 1.38E-01 | 4.07E-01 | -5.138 |
| MAPK6 |  | 0.088 | 8.887 | 1.487 | 1.38E-01 | 4.08E-01 | -5.138 |
| CROCC |  | 0.058 | 6.635 | 1.487 | 1.38E-01 | 4.08E-01 | -5.139 |
| MBP |  | 0.073 | 6.184 | 1.486 | 1.38E-01 | 4.08E-01 | -5.14 |
| PPP2R5E |  | 0.059 | 9.822 | 1.485 | 1.38E-01 | 4.09E-01 | -5.142 |
| PTPN23 |  | -0.062 | 9.367 | -1.485 | 1.38E-01 | 4.09E-01 | -5.142 |
| UPF2 |  | 0.052 | 8.529 | 1.484 | 1.38E-01 | 4.09E-01 | -5.143 |
| THRSP |  | 0.076 | 4.833 | 1.484 | 1.38E-01 | 4.09E-01 | -5.143 |
| MYOCD |  | -0.088 | 5.434 | -1.484 | 1.39E-01 | 4.09E-01 | -5.144 |
| SPRED2 |  | -0.082 | 6.705 | -1.484 | 1.39E-01 | 4.09E-01 | -5.144 |
| SLC6A15 |  | -0.067 | 5.291 | -1.484 | 1.39E-01 | 4.09E-01 | -5.144 |
| STK16 |  | 0.058 | 7.384 | 1.483 | 1.39E-01 | 4.09E-01 | -5.144 |
| PDE7B |  | -0.096 | 5.833 | -1.483 | 1.39E-01 | 4.10E-01 | -5.146 |
| GPR45 |  | -0.117 | 6.046 | -1.482 | 1.39E-01 | 4.10E-01 | -5.147 |
| DOCK1 |  | -0.072 | 8.146 | -1.482 | 1.39E-01 | 4.10E-01 | -5.147 |
| SLC25A28 |  | -0.049 | 9.888 | -1.481 | 1.39E-01 | 4.10E-01 | -5.148 |
| HPS4 |  | 0.045 | 7.606 | 1.48 | 1.40E-01 | 4.11E-01 | -5.149 |
| VRK2 |  | -0.05 | 7.881 | -1.48 | 1.40E-01 | 4.11E-01 | -5.149 |
| ZBTB24 |  | 0.063 | 7.962 | 1.479 | 1.40E-01 | 4.11E-01 | -5.149 |
| EN1 |  | 0.089 | 4.847 | 1.479 | 1.40E-01 | 4.11E-01 | -5.15 |
| DHRS9 |  | -0.1 | 7.202 | -1.48 | 1.40E-01 | 4.11E-01 | -5.15 |
| NSUN5 |  | 0.07 | 8.974 | 1.478 | 1.40E-01 | 4.11E-01 | -5.151 |
| ILKAP |  | 0.05 | 8.237 | 1.478 | 1.40E-01 | 4.11E-01 | -5.152 |
| HAS1 |  | -0.092 | 5.185 | -1.478 | 1.40E-01 | 4.11E-01 | -5.152 |
| TCEB1 |  | -0.047 | 10.364 | -1.477 | 1.41E-01 | 4.12E-01 | -5.154 |
| CD160 |  | 0.095 | 5.672 | 1.475 | 1.41E-01 | 4.13E-01 | -5.156 |
| CDK5R1 |  | -0.073 | 6.413 | -1.475 | 1.41E-01 | 4.13E-01 | -5.157 |
| BRAP |  | -0.069 | 6.895 | -1.475 | 1.41E-01 | 4.13E-01 | -5.157 |
| ALDOC |  | -0.112 | 7.319 | -1.475 | 1.41E-01 | 4.13E-01 | -5.157 |
| ZNF34 |  | 0.056 | 6.799 | 1.474 | 1.41E-01 | 4.13E-01 | -5.157 |
| RPP14 |  | 0.059 | 7.981 | 1.474 | 1.41E-01 | 4.13E-01 | -5.157 |
| PTGIS |  | 0.099 | 5.801 | 1.473 | 1.41E-01 | 4.13E-01 | -5.158 |
| DLL3 |  | 0.076 | 5.386 | 1.473 | 1.41E-01 | 4.13E-01 | -5.158 |
| ANAPC10 |  | 0.062 | 8.584 | 1.473 | 1.41E-01 | 4.13E-01 | -5.158 |
| HAPLN1 |  | -0.121 | 7.82 | -1.474 | 1.41E-01 | 4.13E-01 | -5.158 |
| POP4 |  | 0.044 | 9.672 | 1.472 | 1.42E-01 | 4.13E-01 | -5.159 |
| PRCC |  | 0.059 | 8.045 | 1.472 | 1.42E-01 | 4.13E-01 | -5.159 |
| LLGL2 |  | 0.072 | 7.366 | 1.472 | 1.42E-01 | 4.13E-01 | -5.16 |
| RECK |  | 0.075 | 7.826 | 1.472 | 1.42E-01 | 4.13E-01 | -5.16 |
| LAPTM4B |  | -0.073 | 9.514 | -1.472 | 1.42E-01 | 4.13E-01 | -5.161 |
| HEXA |  | -0.058 | 8.505 | -1.472 | 1.42E-01 | 4.13E-01 | -5.161 |
| MYLK2 |  | -0.087 | 5.568 | -1.471 | 1.42E-01 | 4.13E-01 | -5.162 |
| MTMR6 |  | -0.087 | 7.701 | -1.471 | 1.42E-01 | 4.13E-01 | -5.162 |
| FGF3 |  | -0.101 | 7.14 | -1.471 | 1.42E-01 | 4.14E-01 | -5.163 |
| MDC1 |  | 0.059 | 8.968 | 1.468 | 1.43E-01 | 4.15E-01 | -5.165 |
| QKI |  | -0.08 | 7.412 | -1.469 | 1.43E-01 | 4.15E-01 | -5.165 |
| NUDT5 |  | 0.063 | 10.698 | 1.467 | 1.43E-01 | 4.15E-01 | -5.166 |
| SPO11 |  | -0.086 | 4.754 | -1.468 | 1.43E-01 | 4.15E-01 | -5.166 |
| PTEN |  | -0.07 | 10.037 | -1.468 | 1.43E-01 | 4.15E-01 | -5.167 |
| TBX3 |  | -0.088 | 8.148 | -1.467 | 1.43E-01 | 4.15E-01 | -5.167 |
| HOXA5 |  | 0.097 | 6.835 | 1.466 | 1.43E-01 | 4.16E-01 | -5.168 |
| EYA3 |  | 0.051 | 6.23 | 1.465 | 1.44E-01 | 4.16E-01 | -5.169 |
| GRK1 |  | -0.077 | 5.212 | -1.466 | 1.44E-01 | 4.16E-01 | -5.169 |
| C20orf96 |  | 0.099 | 5.527 | 1.465 | 1.44E-01 | 4.16E-01 | -5.17 |
| WFDC5 |  | -0.079 | 5.722 | -1.465 | 1.44E-01 | 4.16E-01 | -5.17 |
| C9orf41 |  | -0.075 | 5.651 | -1.465 | 1.44E-01 | 4.16E-01 | -5.171 |
| CXCL10 |  | -0.251 | 6.706 | -1.465 | 1.44E-01 | 4.16E-01 | -5.171 |
| GUCY2D |  | 0.066 | 4.824 | 1.464 | 1.44E-01 | 4.16E-01 | -5.171 |
| AQP5 |  | -0.069 | 6.224 | -1.464 | 1.44E-01 | 4.17E-01 | -5.173 |
| ARMC4 |  | 0.092 | 5.362 | 1.462 | 1.44E-01 | 4.17E-01 | -5.173 |
| MPHOSPH10 |  | 0.066 | 9.261 | 1.461 | 1.45E-01 | 4.17E-01 | -5.174 |
| FIP1L1 |  | 0.089 | 9.131 | 1.461 | 1.45E-01 | 4.18E-01 | -5.175 |
| LIMK2 |  | -0.068 | 9.584 | -1.462 | 1.45E-01 | 4.18E-01 | -5.175 |
| ARL2 |  | 0.055 | 10.463 | 1.46 | 1.45E-01 | 4.18E-01 | -5.176 |
| LOR |  | 0.096 | 5.089 | 1.46 | 1.45E-01 | 4.18E-01 | -5.176 |
| TEX14 |  | 0.071 | 5.249 | 1.46 | 1.45E-01 | 4.18E-01 | -5.176 |
| PTPRN2 |  | -0.064 | 6.424 | -1.46 | 1.45E-01 | 4.18E-01 | -5.178 |
| PTPN22 |  | 0.085 | 5.689 | 1.459 | 1.45E-01 | 4.18E-01 | -5.178 |
| GLB1L |  | -0.083 | 7.259 | -1.459 | 1.45E-01 | 4.18E-01 | -5.179 |
| HOXB5 |  | -0.081 | 6.847 | -1.459 | 1.45E-01 | 4.18E-01 | -5.179 |
| NAPB |  | 0.077 | 6.887 | 1.458 | 1.46E-01 | 4.18E-01 | -5.18 |
| APOL2 |  | -0.068 | 9.284 | -1.459 | 1.46E-01 | 4.18E-01 | -5.18 |
| TLR9 |  | 0.087 | 5.994 | 1.458 | 1.46E-01 | 4.18E-01 | -5.18 |
| NARFL |  | -0.077 | 7.96 | -1.458 | 1.46E-01 | 4.18E-01 | -5.18 |
| TMEM27 |  | -0.117 | 5.746 | -1.458 | 1.46E-01 | 4.18E-01 | -5.18 |
| WDR19 |  | 0.063 | 7.035 | 1.456 | 1.46E-01 | 4.19E-01 | -5.181 |
| RASSF1 |  | 0.049 | 8.639 | 1.456 | 1.46E-01 | 4.19E-01 | -5.182 |
| BCCIP |  | 0.058 | 8.098 | 1.455 | 1.46E-01 | 4.19E-01 | -5.183 |
| PXMP4 |  | 0.057 | 7.277 | 1.454 | 1.46E-01 | 4.20E-01 | -5.184 |
| ANXA11 |  | 0.055 | 9.095 | 1.454 | 1.47E-01 | 4.20E-01 | -5.185 |
| BAZ2B |  | -0.077 | 8.543 | -1.454 | 1.47E-01 | 4.21E-01 | -5.186 |
| SLC25A24 |  | -0.081 | 8.324 | -1.453 | 1.47E-01 | 4.21E-01 | -5.188 |
| EIF1AX |  | -0.084 | 9.997 | -1.453 | 1.47E-01 | 4.21E-01 | -5.188 |
| AK2 |  | -0.044 | 9.003 | -1.452 | 1.47E-01 | 4.21E-01 | -5.188 |
| CA11 |  | 0.082 | 6.54 | 1.451 | 1.47E-01 | 4.21E-01 | -5.188 |
| TYK2 |  | 0.069 | 8.783 | 1.451 | 1.47E-01 | 4.21E-01 | -5.189 |
| IRS1 |  | 0.08 | 7.541 | 1.45 | 1.48E-01 | 4.21E-01 | -5.19 |
| WDR5B |  | 0.068 | 6.427 | 1.45 | 1.48E-01 | 4.21E-01 | -5.19 |
| TRIM52 |  | -0.063 | 7.797 | -1.45 | 1.48E-01 | 4.22E-01 | -5.192 |
| EDA |  | -0.065 | 5.928 | -1.45 | 1.48E-01 | 4.22E-01 | -5.192 |
| ZNF3 |  | -0.04 | 7.626 | -1.45 | 1.48E-01 | 4.22E-01 | -5.192 |
| PCBP1 |  | 0.071 | 12.592 | 1.448 | 1.48E-01 | 4.23E-01 | -5.193 |
| STAC2 |  | 0.096 | 5.8 | 1.447 | 1.49E-01 | 4.23E-01 | -5.195 |
| ARRDC1 |  | 0.068 | 8.513 | 1.446 | 1.49E-01 | 4.24E-01 | -5.196 |
| CDK5R2 |  | -0.118 | 7.095 | -1.447 | 1.49E-01 | 4.24E-01 | -5.196 |
| STIP1 |  | 0.079 | 9.616 | 1.446 | 1.49E-01 | 4.24E-01 | -5.196 |
| ZNF468 |  | -0.09 | 7.796 | -1.446 | 1.49E-01 | 4.24E-01 | -5.197 |
| GALNT10 |  | -0.066 | 7.267 | -1.446 | 1.49E-01 | 4.24E-01 | -5.197 |
| MC1R |  | 0.099 | 8.942 | 1.445 | 1.49E-01 | 4.24E-01 | -5.198 |
| NUMB |  | 0.054 | 9.553 | 1.445 | 1.49E-01 | 4.24E-01 | -5.198 |
| UBE2G2 |  | 0.059 | 8.553 | 1.444 | 1.49E-01 | 4.24E-01 | -5.198 |
| RPS14 |  | 0.055 | 13.006 | 1.444 | 1.49E-01 | 4.24E-01 | -5.198 |
| FGFR1 |  | 0.061 | 7.066 | 1.444 | 1.49E-01 | 4.24E-01 | -5.199 |
| CDK5RAP3 |  | 0.09 | 9.111 | 1.444 | 1.49E-01 | 4.24E-01 | -5.199 |
| ELAVL4 |  | 0.08 | 5.063 | 1.443 | 1.50E-01 | 4.24E-01 | -5.2 |
| ATP5S |  | 0.058 | 8.025 | 1.442 | 1.50E-01 | 4.24E-01 | -5.201 |
| ZNF345 |  | 0.07 | 6.628 | 1.442 | 1.50E-01 | 4.24E-01 | -5.201 |
| GRIK2 |  | 0.068 | 5.197 | 1.442 | 1.50E-01 | 4.24E-01 | -5.201 |
| UFD1L |  | -0.051 | 9.741 | -1.443 | 1.50E-01 | 4.24E-01 | -5.201 |
| CECR6 |  | -0.096 | 6.702 | -1.443 | 1.50E-01 | 4.25E-01 | -5.202 |
| GCDH |  | 0.068 | 7.927 | 1.441 | 1.50E-01 | 4.25E-01 | -5.202 |
| JTB |  | 0.043 | 11.723 | 1.439 | 1.51E-01 | 4.26E-01 | -5.205 |
| PAIP2 |  | -0.061 | 10.125 | -1.44 | 1.51E-01 | 4.26E-01 | -5.206 |
| NTSR2 |  | 0.083 | 4.999 | 1.439 | 1.51E-01 | 4.26E-01 | -5.206 |
| ABCA9 |  | -0.082 | 6.514 | -1.439 | 1.51E-01 | 4.26E-01 | -5.206 |
| ARMC2 |  | 0.081 | 5.713 | 1.438 | 1.51E-01 | 4.26E-01 | -5.207 |
| ZFX |  | 0.068 | 7.052 | 1.438 | 1.51E-01 | 4.26E-01 | -5.207 |
| OR10H2 |  | -0.107 | 6.947 | -1.439 | 1.51E-01 | 4.26E-01 | -5.207 |
| PRKAA1 |  | 0.048 | 7.862 | 1.437 | 1.51E-01 | 4.27E-01 | -5.208 |
| HOXD11 |  | 0.091 | 5.645 | 1.437 | 1.51E-01 | 4.27E-01 | -5.208 |
| USP3 |  | -0.065 | 8.822 | -1.437 | 1.52E-01 | 4.28E-01 | -5.21 |
| NUDT6 |  | 0.071 | 7.068 | 1.435 | 1.52E-01 | 4.28E-01 | -5.211 |
| CHDH |  | -0.07 | 5.731 | -1.436 | 1.52E-01 | 4.28E-01 | -5.211 |
| PKP4 |  | 0.073 | 7.059 | 1.434 | 1.52E-01 | 4.28E-01 | -5.212 |
| TNMD |  | 0.09 | 5.905 | 1.434 | 1.52E-01 | 4.28E-01 | -5.212 |
| CCR1 |  | -0.088 | 7.711 | -1.435 | 1.52E-01 | 4.28E-01 | -5.213 |
| DCDC2 |  | -0.102 | 5.198 | -1.434 | 1.52E-01 | 4.29E-01 | -5.214 |
| WFDC1 |  | -0.122 | 8.552 | -1.433 | 1.53E-01 | 4.29E-01 | -5.215 |
| SNX14 |  | 0.074 | 9.353 | 1.432 | 1.53E-01 | 4.29E-01 | -5.215 |
| ARHGEF12 |  | -0.086 | 7.439 | -1.432 | 1.53E-01 | 4.29E-01 | -5.216 |
| MYNN |  | -0.062 | 7.319 | -1.432 | 1.53E-01 | 4.30E-01 | -5.217 |
| RABGAP1L |  | 0.061 | 7.425 | 1.43 | 1.53E-01 | 4.30E-01 | -5.218 |
| SLC6A17 |  | -0.103 | 5.88 | -1.43 | 1.53E-01 | 4.30E-01 | -5.219 |
| WNK4 |  | -0.074 | 5.708 | -1.43 | 1.54E-01 | 4.30E-01 | -5.219 |
| BTNL3 |  | -0.178 | 5.458 | -1.43 | 1.54E-01 | 4.30E-01 | -5.219 |
| MASP2 |  | -0.061 | 5.432 | -1.429 | 1.54E-01 | 4.31E-01 | -5.22 |
| RHEB |  | 0.061 | 9.992 | 1.427 | 1.54E-01 | 4.31E-01 | -5.221 |
| COMMD3 |  | 0.045 | 10.868 | 1.427 | 1.54E-01 | 4.31E-01 | -5.222 |
| PELI2 |  | 0.064 | 7.854 | 1.427 | 1.54E-01 | 4.31E-01 | -5.222 |
| SKIL |  | 0.076 | 7.224 | 1.427 | 1.54E-01 | 4.31E-01 | -5.222 |
| SCNN1D |  | 0.083 | 6.577 | 1.427 | 1.54E-01 | 4.31E-01 | -5.222 |
| GLI4 |  | 0.075 | 7.221 | 1.425 | 1.55E-01 | 4.32E-01 | -5.224 |
| KHSRP |  | -0.063 | 9.734 | -1.426 | 1.55E-01 | 4.32E-01 | -5.224 |
| TNFRSF4 |  | 0.087 | 6.989 | 1.425 | 1.55E-01 | 4.32E-01 | -5.225 |
| LGALS14 |  | -0.129 | 9.045 | -1.426 | 1.55E-01 | 4.32E-01 | -5.225 |
| EIF5 |  | -0.067 | 8.765 | -1.425 | 1.55E-01 | 4.33E-01 | -5.227 |
| EMP3 |  | 0.076 | 10.165 | 1.423 | 1.55E-01 | 4.33E-01 | -5.227 |
| THRB |  | 0.082 | 5.429 | 1.423 | 1.55E-01 | 4.33E-01 | -5.227 |
| DDX42 |  | 0.052 | 9.474 | 1.422 | 1.56E-01 | 4.33E-01 | -5.228 |
| PITPNB |  | -0.077 | 10.048 | -1.423 | 1.56E-01 | 4.33E-01 | -5.229 |
| ART5 |  | 0.114 | 5.575 | 1.422 | 1.56E-01 | 4.33E-01 | -5.229 |
| ELK4 |  | -0.075 | 6.467 | -1.422 | 1.56E-01 | 4.34E-01 | -5.231 |
| RAD52 |  | -0.059 | 6.542 | -1.421 | 1.56E-01 | 4.34E-01 | -5.231 |
| NPB |  | -0.113 | 7.171 | -1.421 | 1.56E-01 | 4.35E-01 | -5.232 |
| ANXA7 |  | -0.043 | 9.612 | -1.42 | 1.56E-01 | 4.35E-01 | -5.233 |
| POLR3A |  | -0.05 | 7.818 | -1.419 | 1.57E-01 | 4.35E-01 | -5.234 |
| ID2 |  | -0.091 | 10.107 | -1.419 | 1.57E-01 | 4.35E-01 | -5.234 |
| RPS10 |  | 0.058 | 13.661 | 1.417 | 1.57E-01 | 4.36E-01 | -5.236 |
| RAB15 |  | -0.078 | 8.41 | -1.417 | 1.57E-01 | 4.36E-01 | -5.236 |
| ANAPC4 |  | 0.07 | 9.797 | 1.416 | 1.57E-01 | 4.36E-01 | -5.237 |
| KCNA10 |  | -0.088 | 4.866 | -1.417 | 1.57E-01 | 4.36E-01 | -5.237 |
| LIPH |  | 0.081 | 5.166 | 1.416 | 1.57E-01 | 4.36E-01 | -5.237 |
| ATRNL1 |  | -0.077 | 5.219 | -1.417 | 1.57E-01 | 4.36E-01 | -5.237 |
| ATP1B2 |  | -0.1 | 7.326 | -1.416 | 1.58E-01 | 4.36E-01 | -5.238 |
| SCGB3A2 |  | 0.088 | 6.589 | 1.415 | 1.58E-01 | 4.36E-01 | -5.238 |
| DNAJB8 |  | -0.091 | 5.165 | -1.416 | 1.58E-01 | 4.36E-01 | -5.239 |
| FBXO31 |  | -0.076 | 7.687 | -1.416 | 1.58E-01 | 4.36E-01 | -5.239 |
| TRIM14 |  | 0.063 | 7.152 | 1.415 | 1.58E-01 | 4.36E-01 | -5.239 |
| NPPA |  | -0.12 | 5.328 | -1.415 | 1.58E-01 | 4.36E-01 | -5.239 |
| MMAA |  | -0.053 | 6.795 | -1.415 | 1.58E-01 | 4.36E-01 | -5.239 |
| DNAJC14 |  | -0.086 | 7.41 | -1.415 | 1.58E-01 | 4.36E-01 | -5.24 |
| NMNAT3 |  | 0.094 | 5.885 | 1.412 | 1.58E-01 | 4.37E-01 | -5.242 |
| NEUROD6 |  | 0.072 | 4.66 | 1.412 | 1.59E-01 | 4.37E-01 | -5.242 |
| ENTPD5 |  | 0.083 | 6.001 | 1.412 | 1.59E-01 | 4.37E-01 | -5.243 |
| PSEN2 |  | -0.058 | 8.499 | -1.412 | 1.59E-01 | 4.37E-01 | -5.243 |
| FBXL22 |  | 0.094 | 5.379 | 1.411 | 1.59E-01 | 4.37E-01 | -5.243 |
| UQCRB |  | 0.083 | 8.621 | 1.411 | 1.59E-01 | 4.37E-01 | -5.243 |
| NEU1 |  | 0.051 | 10.104 | 1.411 | 1.59E-01 | 4.37E-01 | -5.244 |
| MTMR9 |  | -0.073 | 7.863 | -1.412 | 1.59E-01 | 4.37E-01 | -5.244 |
| CISH |  | -0.095 | 8.928 | -1.411 | 1.59E-01 | 4.37E-01 | -5.245 |
| G0S2 |  | -0.161 | 8.001 | -1.411 | 1.59E-01 | 4.37E-01 | -5.245 |
| SNX9 |  | 0.076 | 7.828 | 1.41 | 1.59E-01 | 4.37E-01 | -5.245 |
| APBA2 |  | -0.083 | 5.884 | -1.411 | 1.59E-01 | 4.37E-01 | -5.245 |
| SLC27A6 |  | -0.118 | 6.484 | -1.411 | 1.59E-01 | 4.37E-01 | -5.245 |
| NGFRAP1 |  | 0.086 | 11.869 | 1.41 | 1.59E-01 | 4.37E-01 | -5.245 |
| RNF7 |  | 0.048 | 10.013 | 1.409 | 1.59E-01 | 4.37E-01 | -5.246 |
| ZNF79 |  | -0.065 | 7.167 | -1.41 | 1.59E-01 | 4.37E-01 | -5.246 |
| DUOX1 |  | 0.087 | 6.388 | 1.409 | 1.60E-01 | 4.38E-01 | -5.247 |
| CTSH |  | 0.091 | 9.868 | 1.408 | 1.60E-01 | 4.38E-01 | -5.248 |
| LIFR |  | 0.11 | 7.889 | 1.407 | 1.60E-01 | 4.39E-01 | -5.249 |
| MEOX1 |  | 0.103 | 5.404 | 1.405 | 1.60E-01 | 4.40E-01 | -5.251 |
| ZNF136 |  | -0.042 | 7.371 | -1.406 | 1.60E-01 | 4.40E-01 | -5.251 |
| RHBDL2 |  | 0.079 | 5.33 | 1.404 | 1.61E-01 | 4.40E-01 | -5.252 |
| BSN |  | -0.078 | 5.115 | -1.405 | 1.61E-01 | 4.40E-01 | -5.252 |
| BMP15 |  | 0.081 | 4.87 | 1.404 | 1.61E-01 | 4.40E-01 | -5.252 |
| MEST |  | -0.1 | 9.757 | -1.405 | 1.61E-01 | 4.40E-01 | -5.253 |
| SIPA1L1 |  | 0.078 | 9.38 | 1.404 | 1.61E-01 | 4.40E-01 | -5.253 |
| SEMA3F |  | 0.091 | 7.35 | 1.403 | 1.61E-01 | 4.40E-01 | -5.254 |
| MYO1C |  | 0.07 | 9.9 | 1.403 | 1.61E-01 | 4.40E-01 | -5.254 |
| SIGLEC7 |  | -0.077 | 6.358 | -1.404 | 1.61E-01 | 4.40E-01 | -5.254 |
| TGFBR3 |  | -0.075 | 10.016 | -1.404 | 1.61E-01 | 4.40E-01 | -5.254 |
| INHBC |  | -0.088 | 6.198 | -1.403 | 1.61E-01 | 4.40E-01 | -5.255 |
| KNG1 |  | -0.069 | 5.202 | -1.403 | 1.62E-01 | 4.40E-01 | -5.256 |
| SUMF2 |  | 0.094 | 9.208 | 1.401 | 1.62E-01 | 4.40E-01 | -5.256 |
| DUSP15 |  | -0.077 | 6.519 | -1.402 | 1.62E-01 | 4.40E-01 | -5.256 |
| DLL4 |  | -0.089 | 5.924 | -1.402 | 1.62E-01 | 4.40E-01 | -5.257 |
| TAS2R8 |  | -0.078 | 4.629 | -1.402 | 1.62E-01 | 4.40E-01 | -5.257 |
| SUPV3L1 |  | -0.056 | 8.343 | -1.402 | 1.62E-01 | 4.40E-01 | -5.257 |
| SLC2A13 |  | 0.053 | 5.703 | 1.401 | 1.62E-01 | 4.40E-01 | -5.257 |
| PGD |  | -0.086 | 10.039 | -1.402 | 1.62E-01 | 4.40E-01 | -5.257 |
| MAPK3 |  | 0.076 | 9.428 | 1.4 | 1.62E-01 | 4.41E-01 | -5.258 |
| PRODH2 |  | -0.088 | 5.026 | -1.4 | 1.62E-01 | 4.41E-01 | -5.259 |
| TOB1 |  | 0.082 | 8.228 | 1.399 | 1.62E-01 | 4.41E-01 | -5.26 |
| LRRC18 |  | -0.094 | 5.48 | -1.4 | 1.62E-01 | 4.41E-01 | -5.26 |
| ELF1 |  | -0.096 | 9.315 | -1.399 | 1.63E-01 | 4.42E-01 | -5.261 |
| AOAH |  | -0.076 | 7.115 | -1.398 | 1.63E-01 | 4.42E-01 | -5.262 |
| ATP8B4 |  | -0.086 | 7.338 | -1.398 | 1.63E-01 | 4.42E-01 | -5.262 |
| ABTB2 |  | -0.098 | 7.671 | -1.398 | 1.63E-01 | 4.42E-01 | -5.262 |
| TAS2R7 |  | -0.082 | 4.726 | -1.398 | 1.63E-01 | 4.42E-01 | -5.262 |
| TOM1L1 |  | -0.079 | 6.159 | -1.397 | 1.63E-01 | 4.42E-01 | -5.263 |
| SRC |  | -0.075 | 6.974 | -1.397 | 1.63E-01 | 4.42E-01 | -5.264 |
| KIF11 |  | 0.098 | 7.788 | 1.395 | 1.63E-01 | 4.42E-01 | -5.264 |
| ARID3A |  | 0.084 | 10.157 | 1.395 | 1.64E-01 | 4.43E-01 | -5.265 |
| TPM3 |  | -0.043 | 9.142 | -1.396 | 1.64E-01 | 4.43E-01 | -5.265 |
| RPS7 |  | -0.066 | 12.233 | -1.395 | 1.64E-01 | 4.43E-01 | -5.266 |
| HAND1 |  | -0.108 | 5.817 | -1.395 | 1.64E-01 | 4.43E-01 | -5.267 |
| MYOG |  | -0.098 | 6.881 | -1.394 | 1.64E-01 | 4.44E-01 | -5.268 |
| GABRD |  | -0.098 | 5.071 | -1.393 | 1.64E-01 | 4.44E-01 | -5.269 |
| BRCA2 |  | -0.072 | 5.895 | -1.393 | 1.64E-01 | 4.44E-01 | -5.269 |
| ZNF529 |  | -0.064 | 7.507 | -1.393 | 1.65E-01 | 4.44E-01 | -5.269 |
| SF1 |  | -0.052 | 9.191 | -1.393 | 1.65E-01 | 4.44E-01 | -5.269 |
| ST14 |  | 0.065 | 8.154 | 1.391 | 1.65E-01 | 4.45E-01 | -5.271 |
| WDR53 |  | 0.044 | 7.996 | 1.39 | 1.65E-01 | 4.45E-01 | -5.271 |
| RAI1 |  | -0.065 | 7.135 | -1.391 | 1.65E-01 | 4.45E-01 | -5.272 |
| JUN |  | 0.134 | 9.292 | 1.389 | 1.65E-01 | 4.45E-01 | -5.272 |
| TCEAL7 |  | -0.088 | 5.845 | -1.389 | 1.66E-01 | 4.46E-01 | -5.275 |
| IDS |  | 0.056 | 8.933 | 1.387 | 1.66E-01 | 4.46E-01 | -5.275 |
| CETN2 |  | 0.06 | 10.049 | 1.387 | 1.66E-01 | 4.46E-01 | -5.275 |
| COMMD10 |  | -0.068 | 9.117 | -1.388 | 1.66E-01 | 4.46E-01 | -5.276 |
| BIRC3 |  | 0.137 | 7.591 | 1.387 | 1.66E-01 | 4.46E-01 | -5.276 |
| MAST2 |  | 0.072 | 7.332 | 1.386 | 1.66E-01 | 4.46E-01 | -5.277 |
| INPP4B |  | 0.087 | 6.555 | 1.386 | 1.66E-01 | 4.46E-01 | -5.277 |
| AP1S3 |  | 0.097 | 5.953 | 1.386 | 1.66E-01 | 4.46E-01 | -5.277 |
| SNX8 |  | -0.08 | 8.386 | -1.387 | 1.66E-01 | 4.46E-01 | -5.277 |
| PEX12 |  | -0.067 | 7.202 | -1.387 | 1.66E-01 | 4.46E-01 | -5.277 |
| OXCT2 |  | 0.094 | 5.96 | 1.385 | 1.66E-01 | 4.46E-01 | -5.278 |
| TPO |  | 0.081 | 5.278 | 1.385 | 1.67E-01 | 4.46E-01 | -5.278 |
| DGKE |  | 0.064 | 5.291 | 1.384 | 1.67E-01 | 4.47E-01 | -5.279 |
| EFTUD1 |  | 0.069 | 7.099 | 1.384 | 1.67E-01 | 4.47E-01 | -5.28 |
| PCNA |  | -0.062 | 9.765 | -1.384 | 1.67E-01 | 4.47E-01 | -5.28 |
| PPEF2 |  | -0.1 | 4.545 | -1.384 | 1.67E-01 | 4.47E-01 | -5.281 |
| MESP1 |  | 0.088 | 6.464 | 1.383 | 1.67E-01 | 4.47E-01 | -5.281 |
| AOC3 |  | -0.111 | 8.163 | -1.384 | 1.67E-01 | 4.47E-01 | -5.281 |
| ANAPC11 |  | 0.043 | 10.604 | 1.383 | 1.67E-01 | 4.47E-01 | -5.282 |
| CYP11B2 |  | -0.083 | 5.541 | -1.383 | 1.67E-01 | 4.47E-01 | -5.282 |
| PAK7 |  | 0.063 | 4.831 | 1.382 | 1.68E-01 | 4.47E-01 | -5.282 |
| DSCR3 |  | 0.054 | 8.566 | 1.382 | 1.68E-01 | 4.47E-01 | -5.282 |
| KLK14 |  | -0.077 | 5.854 | -1.382 | 1.68E-01 | 4.48E-01 | -5.284 |
| SLC15A3 |  | 0.082 | 10.135 | 1.381 | 1.68E-01 | 4.48E-01 | -5.284 |
| ST3GAL4 |  | -0.105 | 9.097 | -1.382 | 1.68E-01 | 4.48E-01 | -5.284 |
| FAM20C |  | -0.077 | 8.127 | -1.382 | 1.68E-01 | 4.48E-01 | -5.284 |
| PPP3CB |  | 0.054 | 9.369 | 1.379 | 1.68E-01 | 4.49E-01 | -5.286 |
| ETS1 |  | -0.068 | 8.356 | -1.38 | 1.68E-01 | 4.49E-01 | -5.286 |
| DNER |  | -0.097 | 5.361 | -1.38 | 1.69E-01 | 4.49E-01 | -5.287 |
| MAGEA11 |  | -0.088 | 6.018 | -1.379 | 1.69E-01 | 4.49E-01 | -5.287 |
| GABRP |  | -0.096 | 6.979 | -1.379 | 1.69E-01 | 4.49E-01 | -5.287 |
| EMID1 |  | -0.09 | 6.281 | -1.379 | 1.69E-01 | 4.49E-01 | -5.287 |
| LHX4 |  | 0.106 | 4.987 | 1.378 | 1.69E-01 | 4.49E-01 | -5.288 |
| UXS1 |  | 0.056 | 8.643 | 1.378 | 1.69E-01 | 4.49E-01 | -5.288 |
| GFRA4 |  | -0.079 | 7.036 | -1.379 | 1.69E-01 | 4.49E-01 | -5.288 |
| NOTCH1 |  | -0.072 | 7.957 | -1.378 | 1.69E-01 | 4.49E-01 | -5.289 |
| CTBS |  | -0.059 | 9.238 | -1.378 | 1.69E-01 | 4.49E-01 | -5.289 |
| LOX |  | -0.107 | 7.055 | -1.377 | 1.69E-01 | 4.49E-01 | -5.29 |
| ZPBP2 |  | -0.058 | 4.471 | -1.377 | 1.69E-01 | 4.49E-01 | -5.29 |
| ZBTB33 |  | 0.072 | 7.864 | 1.375 | 1.70E-01 | 4.49E-01 | -5.291 |
| GIF |  | -0.073 | 4.782 | -1.376 | 1.70E-01 | 4.49E-01 | -5.291 |
| ENPEP |  | 0.071 | 6.429 | 1.375 | 1.70E-01 | 4.49E-01 | -5.291 |
| DPYSL4 |  | -0.116 | 6.302 | -1.376 | 1.70E-01 | 4.49E-01 | -5.292 |
| TALDO1 |  | -0.049 | 11.407 | -1.376 | 1.70E-01 | 4.49E-01 | -5.292 |
| SF3A2 |  | -0.09 | 9.969 | -1.376 | 1.70E-01 | 4.49E-01 | -5.292 |
| RAPGEF3 |  | 0.068 | 7.55 | 1.373 | 1.70E-01 | 4.50E-01 | -5.294 |
| SLCO2B1 |  | -0.065 | 7.694 | -1.374 | 1.70E-01 | 4.50E-01 | -5.294 |
| N4BP2 |  | -0.074 | 8.086 | -1.374 | 1.70E-01 | 4.50E-01 | -5.294 |
| LGALS2 |  | 0.112 | 6.781 | 1.373 | 1.70E-01 | 4.50E-01 | -5.294 |
| TM4SF1 |  | -0.09 | 10.467 | -1.373 | 1.71E-01 | 4.51E-01 | -5.295 |
| EIF4E2 |  | 0.047 | 10.579 | 1.372 | 1.71E-01 | 4.51E-01 | -5.296 |
| SFN |  | 0.117 | 6.957 | 1.371 | 1.71E-01 | 4.51E-01 | -5.297 |
| ADCYAP1 |  | 0.077 | 5.347 | 1.371 | 1.71E-01 | 4.51E-01 | -5.297 |
| MYOZ3 |  | 0.075 | 6.431 | 1.37 | 1.71E-01 | 4.51E-01 | -5.298 |
| PEX5 |  | -0.059 | 8.279 | -1.371 | 1.71E-01 | 4.51E-01 | -5.298 |
| PRRX2 |  | -0.078 | 7.183 | -1.371 | 1.71E-01 | 4.52E-01 | -5.298 |
| GKN1 |  | 0.194 | 6.236 | 1.369 | 1.71E-01 | 4.52E-01 | -5.299 |
| PSMC2 |  | -0.049 | 10.943 | -1.37 | 1.72E-01 | 4.52E-01 | -5.299 |
| IGBP1 |  | 0.05 | 9.926 | 1.369 | 1.72E-01 | 4.52E-01 | -5.299 |
| PCDH12 |  | -0.086 | 7.053 | -1.368 | 1.72E-01 | 4.53E-01 | -5.302 |
| DYRK3 |  | 0.096 | 6.576 | 1.366 | 1.72E-01 | 4.53E-01 | -5.303 |
| CNGA1 |  | -0.115 | 6.178 | -1.367 | 1.73E-01 | 4.53E-01 | -5.303 |
| ZNF546 |  | -0.067 | 5.553 | -1.367 | 1.73E-01 | 4.53E-01 | -5.304 |
| MMP24 |  | 0.092 | 5.878 | 1.366 | 1.73E-01 | 4.53E-01 | -5.304 |
| PHC1 |  | 0.052 | 7.499 | 1.366 | 1.73E-01 | 4.53E-01 | -5.304 |
| DCBLD2 |  | 0.068 | 7.355 | 1.365 | 1.73E-01 | 4.53E-01 | -5.304 |
| EFNA2 |  | -0.09 | 5.959 | -1.365 | 1.73E-01 | 4.54E-01 | -5.305 |
| TLR6 |  | -0.073 | 6.279 | -1.365 | 1.73E-01 | 4.54E-01 | -5.306 |
| SMARCA4 |  | -0.048 | 10.805 | -1.365 | 1.73E-01 | 4.54E-01 | -5.306 |
| TRPM3 |  | 0.058 | 6.225 | 1.363 | 1.73E-01 | 4.54E-01 | -5.307 |
| GNAQ |  | -0.057 | 8.65 | -1.364 | 1.73E-01 | 4.54E-01 | -5.307 |
| NFRKB |  | -0.062 | 6.907 | -1.363 | 1.74E-01 | 4.55E-01 | -5.308 |
| WNT10B |  | 0.097 | 6.107 | 1.361 | 1.74E-01 | 4.56E-01 | -5.31 |
| MST1R |  | 0.078 | 5.905 | 1.361 | 1.74E-01 | 4.56E-01 | -5.31 |
| KLK2 |  | 0.071 | 5.511 | 1.361 | 1.74E-01 | 4.56E-01 | -5.31 |
| BCOR |  | -0.061 | 7.138 | -1.362 | 1.74E-01 | 4.56E-01 | -5.31 |
| NOX4 |  | 0.079 | 5.971 | 1.359 | 1.75E-01 | 4.57E-01 | -5.313 |
| VIPR1 |  | -0.096 | 6.952 | -1.36 | 1.75E-01 | 4.57E-01 | -5.313 |
| GULP1 |  | -0.091 | 8.724 | -1.359 | 1.75E-01 | 4.57E-01 | -5.314 |
| DDIT4 |  | -0.142 | 9.998 | -1.359 | 1.75E-01 | 4.57E-01 | -5.314 |
| TPCN2 |  | -0.076 | 7.278 | -1.358 | 1.75E-01 | 4.58E-01 | -5.316 |
| STOML3 |  | 0.068 | 5.049 | 1.357 | 1.75E-01 | 4.58E-01 | -5.316 |
| DDX20 |  | -0.048 | 7.967 | -1.357 | 1.76E-01 | 4.58E-01 | -5.317 |
| BFSP1 |  | 0.086 | 6.673 | 1.356 | 1.76E-01 | 4.58E-01 | -5.317 |
| SMPDL3B |  | 0.103 | 5.848 | 1.356 | 1.76E-01 | 4.58E-01 | -5.317 |
| FEZ1 |  | -0.064 | 7.317 | -1.356 | 1.76E-01 | 4.58E-01 | -5.317 |
| KCNC2 |  | -0.055 | 4.665 | -1.356 | 1.76E-01 | 4.58E-01 | -5.318 |
| ZNF354C |  | -0.082 | 5.171 | -1.356 | 1.76E-01 | 4.58E-01 | -5.318 |
| ACTR1A |  | 0.059 | 10.212 | 1.354 | 1.76E-01 | 4.58E-01 | -5.319 |
| CHIC2 |  | -0.063 | 9.342 | -1.355 | 1.76E-01 | 4.58E-01 | -5.319 |
| P2RX7 |  | -0.086 | 6.916 | -1.354 | 1.76E-01 | 4.58E-01 | -5.32 |
| TNFRSF8 |  | -0.112 | 6.894 | -1.354 | 1.76E-01 | 4.58E-01 | -5.32 |
| HOXA10 |  | -0.082 | 7.442 | -1.354 | 1.77E-01 | 4.58E-01 | -5.32 |
| ASF1A |  | 0.075 | 7.403 | 1.353 | 1.77E-01 | 4.58E-01 | -5.32 |
| SF3B4 |  | -0.105 | 8.244 | -1.354 | 1.77E-01 | 4.58E-01 | -5.321 |
| GPR26 |  | -0.076 | 5.172 | -1.354 | 1.77E-01 | 4.58E-01 | -5.321 |
| TBX6 |  | -0.077 | 5.053 | -1.353 | 1.77E-01 | 4.58E-01 | -5.321 |
| CCND3 |  | 0.059 | 10.267 | 1.352 | 1.77E-01 | 4.58E-01 | -5.321 |
| ELOVL3 |  | -0.096 | 5.689 | -1.353 | 1.77E-01 | 4.58E-01 | -5.321 |
| EXOSC7 |  | 0.046 | 9.309 | 1.352 | 1.77E-01 | 4.58E-01 | -5.321 |
| CACNA1F |  | -0.071 | 5.694 | -1.353 | 1.77E-01 | 4.59E-01 | -5.322 |
| ZCCHC9 |  | -0.054 | 9.466 | -1.352 | 1.77E-01 | 4.59E-01 | -5.323 |
| ZNRF2 |  | 0.051 | 7.333 | 1.35 | 1.77E-01 | 4.59E-01 | -5.323 |
| B3GNT6 |  | -0.08 | 6.69 | -1.351 | 1.77E-01 | 4.59E-01 | -5.324 |
| FNDC5 |  | 0.094 | 5.228 | 1.35 | 1.78E-01 | 4.60E-01 | -5.324 |
| HBE1 |  | 0.113 | 6.059 | 1.35 | 1.78E-01 | 4.60E-01 | -5.325 |
| SCN4B |  | -0.13 | 6.28 | -1.35 | 1.78E-01 | 4.60E-01 | -5.325 |
| IL3RA |  | -0.098 | 7.152 | -1.35 | 1.78E-01 | 4.60E-01 | -5.326 |
| FGFRL1 |  | 0.079 | 6.983 | 1.348 | 1.78E-01 | 4.60E-01 | -5.326 |
| MCTS1 |  | 0.073 | 8.994 | 1.348 | 1.78E-01 | 4.60E-01 | -5.327 |
| GRIA4 |  | 0.097 | 5.173 | 1.348 | 1.78E-01 | 4.60E-01 | -5.327 |
| LAG3 |  | 0.104 | 7.05 | 1.347 | 1.78E-01 | 4.60E-01 | -5.327 |
| ADAM22 |  | -0.055 | 5.011 | -1.348 | 1.78E-01 | 4.60E-01 | -5.328 |
| INSM1 |  | -0.093 | 5.183 | -1.348 | 1.78E-01 | 4.60E-01 | -5.328 |
| SLC4A7 |  | 0.082 | 7.139 | 1.346 | 1.79E-01 | 4.61E-01 | -5.33 |
| AURKB |  | -0.085 | 7.607 | -1.345 | 1.79E-01 | 4.62E-01 | -5.331 |
| SPIB |  | 0.069 | 6.135 | 1.344 | 1.79E-01 | 4.62E-01 | -5.331 |
| KAAG1 |  | 0.081 | 5.34 | 1.344 | 1.80E-01 | 4.63E-01 | -5.332 |
| DBH |  | -0.095 | 5.595 | -1.344 | 1.80E-01 | 4.63E-01 | -5.333 |
| FAM47B |  | -0.085 | 4.61 | -1.344 | 1.80E-01 | 4.63E-01 | -5.333 |
| CAST |  | -0.05 | 10.088 | -1.344 | 1.80E-01 | 4.63E-01 | -5.334 |
| ELAVL2 |  | -0.072 | 4.976 | -1.343 | 1.80E-01 | 4.63E-01 | -5.334 |
| CREG1 |  | -0.073 | 11.105 | -1.343 | 1.80E-01 | 4.63E-01 | -5.334 |
| RBX1 |  | 0.066 | 11.415 | 1.341 | 1.80E-01 | 4.64E-01 | -5.336 |
| MCM2 |  | -0.064 | 9.68 | -1.341 | 1.81E-01 | 4.65E-01 | -5.337 |
| RAD9B |  | 0.074 | 4.929 | 1.339 | 1.81E-01 | 4.65E-01 | -5.338 |
| ATRN |  | -0.042 | 8.28 | -1.34 | 1.81E-01 | 4.65E-01 | -5.338 |
| CLCN1 |  | -0.097 | 6.186 | -1.339 | 1.81E-01 | 4.65E-01 | -5.339 |
| ETV4 |  | 0.104 | 6.626 | 1.338 | 1.81E-01 | 4.65E-01 | -5.339 |
| PPHLN1 |  | -0.044 | 8.073 | -1.339 | 1.81E-01 | 4.65E-01 | -5.34 |
| NINJ1 |  | -0.064 | 11.73 | -1.338 | 1.82E-01 | 4.66E-01 | -5.341 |
| HIRIP3 |  | 0.056 | 7.815 | 1.336 | 1.82E-01 | 4.66E-01 | -5.342 |
| IL11 |  | -0.142 | 5.895 | -1.337 | 1.82E-01 | 4.66E-01 | -5.342 |
| RPL27A |  | 0.092 | 9.739 | 1.335 | 1.82E-01 | 4.67E-01 | -5.343 |
| CDH16 |  | 0.096 | 5.531 | 1.334 | 1.83E-01 | 4.67E-01 | -5.344 |
| OR3A3 |  | -0.081 | 5.773 | -1.335 | 1.83E-01 | 4.67E-01 | -5.344 |
| PPP1R13B |  | 0.066 | 7.432 | 1.334 | 1.83E-01 | 4.67E-01 | -5.344 |
| VPS13A |  | 0.055 | 7.521 | 1.334 | 1.83E-01 | 4.67E-01 | -5.345 |
| INE1 |  | 0.085 | 6.406 | 1.334 | 1.83E-01 | 4.67E-01 | -5.345 |
| SERPINI1 |  | -0.121 | 7.01 | -1.333 | 1.83E-01 | 4.68E-01 | -5.347 |
| APOBEC1 |  | 0.072 | 4.697 | 1.331 | 1.84E-01 | 4.69E-01 | -5.349 |
| AKAP8 |  | 0.059 | 9.403 | 1.33 | 1.84E-01 | 4.70E-01 | -5.35 |
| COL2A1 |  | -0.063 | 5.731 | -1.329 | 1.85E-01 | 4.71E-01 | -5.353 |
| LRCH3 |  | 0.05 | 7.1 | 1.327 | 1.85E-01 | 4.72E-01 | -5.354 |
| STAT1 |  | -0.094 | 9.788 | -1.328 | 1.85E-01 | 4.72E-01 | -5.354 |
| CCNJ |  | -0.058 | 7.343 | -1.327 | 1.85E-01 | 4.72E-01 | -5.354 |
| GAS2L1 |  | 0.061 | 7.657 | 1.326 | 1.85E-01 | 4.72E-01 | -5.354 |
| POLDIP2 |  | -0.039 | 9.428 | -1.326 | 1.86E-01 | 4.73E-01 | -5.356 |
| VAT1 |  | 0.058 | 9.48 | 1.325 | 1.86E-01 | 4.73E-01 | -5.356 |
| SPHK2 |  | -0.079 | 8.821 | -1.326 | 1.86E-01 | 4.73E-01 | -5.357 |
| MARS2 |  | -0.07 | 5.771 | -1.324 | 1.86E-01 | 4.74E-01 | -5.358 |
| SNX16 |  | 0.08 | 6.766 | 1.322 | 1.87E-01 | 4.74E-01 | -5.359 |
| VSIG1 |  | 0.071 | 5.55 | 1.322 | 1.87E-01 | 4.75E-01 | -5.36 |
| PHF10 |  | 0.055 | 9.448 | 1.322 | 1.87E-01 | 4.75E-01 | -5.36 |
| PHACTR4 |  | -0.048 | 8.24 | -1.322 | 1.87E-01 | 4.75E-01 | -5.361 |
| CST3 |  | 0.064 | 11.122 | 1.321 | 1.87E-01 | 4.75E-01 | -5.362 |
| CYP4F11 |  | 0.06 | 4.757 | 1.321 | 1.87E-01 | 4.75E-01 | -5.362 |
| KPNA1 |  | -0.056 | 8.291 | -1.321 | 1.87E-01 | 4.75E-01 | -5.362 |
| USP29 |  | 0.066 | 4.731 | 1.32 | 1.87E-01 | 4.75E-01 | -5.362 |
| RPS17 |  | 0.061 | 12.791 | 1.32 | 1.87E-01 | 4.75E-01 | -5.362 |
| ASTN2 |  | -0.072 | 6.759 | -1.321 | 1.87E-01 | 4.75E-01 | -5.362 |
| MEIS2 |  | -0.057 | 6.878 | -1.321 | 1.87E-01 | 4.75E-01 | -5.363 |
| ZNF568 |  | -0.063 | 5.673 | -1.319 | 1.88E-01 | 4.76E-01 | -5.365 |
| SPRR1B |  | 0.093 | 5.7 | 1.317 | 1.88E-01 | 4.76E-01 | -5.366 |
| PPM1F |  | 0.061 | 9.711 | 1.317 | 1.88E-01 | 4.77E-01 | -5.366 |
| NKX3-1 |  | -0.084 | 6.417 | -1.317 | 1.89E-01 | 4.77E-01 | -5.367 |
| ADD1 |  | -0.055 | 9.447 | -1.316 | 1.89E-01 | 4.78E-01 | -5.368 |
| C1R |  | -0.075 | 8.693 | -1.316 | 1.89E-01 | 4.78E-01 | -5.368 |
| TNFRSF10D |  | 0.09 | 6.31 | 1.315 | 1.89E-01 | 4.78E-01 | -5.369 |
| POLR3B |  | 0.05 | 8.899 | 1.315 | 1.89E-01 | 4.78E-01 | -5.369 |
| MX1 |  | 0.131 | 10.957 | 1.315 | 1.89E-01 | 4.78E-01 | -5.369 |
| UQCRFS1 |  | -0.043 | 11.815 | -1.315 | 1.89E-01 | 4.78E-01 | -5.37 |
| ABCF1 |  | -0.049 | 10.484 | -1.313 | 1.90E-01 | 4.79E-01 | -5.372 |
| FAM3A |  | 0.066 | 8.795 | 1.312 | 1.90E-01 | 4.80E-01 | -5.373 |
| SLK |  | -0.066 | 9.339 | -1.313 | 1.90E-01 | 4.80E-01 | -5.373 |
| HES4 |  | -0.104 | 10.453 | -1.312 | 1.90E-01 | 4.80E-01 | -5.373 |
| MEIS3 |  | 0.077 | 5.941 | 1.311 | 1.90E-01 | 4.80E-01 | -5.373 |
| ECHS1 |  | 0.06 | 11.091 | 1.31 | 1.91E-01 | 4.80E-01 | -5.375 |
| RLN2 |  | 0.089 | 5.678 | 1.31 | 1.91E-01 | 4.80E-01 | -5.375 |
| PTPRN |  | -0.09 | 5.208 | -1.311 | 1.91E-01 | 4.80E-01 | -5.375 |
| GTF3C5 |  | 0.048 | 9.025 | 1.31 | 1.91E-01 | 4.80E-01 | -5.376 |
| CSNK1G3 |  | -0.066 | 7.748 | -1.31 | 1.91E-01 | 4.80E-01 | -5.376 |
| LGALS4 |  | 0.075 | 6.008 | 1.309 | 1.91E-01 | 4.80E-01 | -5.376 |
| FER |  | 0.057 | 6.979 | 1.308 | 1.91E-01 | 4.80E-01 | -5.377 |
| PER3 |  | -0.066 | 6.316 | -1.309 | 1.91E-01 | 4.80E-01 | -5.378 |
| ECM2 |  | -0.068 | 5.228 | -1.309 | 1.91E-01 | 4.80E-01 | -5.378 |
| SLC17A1 |  | -0.075 | 4.808 | -1.309 | 1.91E-01 | 4.80E-01 | -5.378 |
| SLC13A2 |  | 0.066 | 5.842 | 1.308 | 1.91E-01 | 4.80E-01 | -5.378 |
| HOXD8 |  | 0.089 | 5.957 | 1.308 | 1.92E-01 | 4.80E-01 | -5.378 |
| MRPL37 |  | 0.057 | 10.203 | 1.306 | 1.92E-01 | 4.81E-01 | -5.379 |
| MFAP3 |  | -0.087 | 7.791 | -1.307 | 1.92E-01 | 4.82E-01 | -5.381 |
| C7orf33 |  | -0.078 | 4.821 | -1.306 | 1.92E-01 | 4.82E-01 | -5.381 |
| MICA |  | 0.076 | 8.161 | 1.305 | 1.92E-01 | 4.82E-01 | -5.381 |
| MRO |  | -0.066 | 4.945 | -1.306 | 1.92E-01 | 4.82E-01 | -5.381 |
| FIGNL1 |  | -0.076 | 6.926 | -1.305 | 1.93E-01 | 4.83E-01 | -5.383 |
| KRT3 |  | -0.095 | 7.451 | -1.304 | 1.93E-01 | 4.83E-01 | -5.384 |
| FBXO22 |  | 0.055 | 7.395 | 1.303 | 1.93E-01 | 4.83E-01 | -5.384 |
| EEF1A1 |  | 0.122 | 14.21 | 1.302 | 1.93E-01 | 4.84E-01 | -5.385 |
| SLC35D2 |  | -0.049 | 8.123 | -1.303 | 1.94E-01 | 4.84E-01 | -5.385 |
| FHL5 |  | -0.098 | 5.651 | -1.302 | 1.94E-01 | 4.84E-01 | -5.387 |
| DUSP6 |  | -0.079 | 9.689 | -1.301 | 1.94E-01 | 4.85E-01 | -5.387 |
| FOXA2 |  | -0.072 | 5.392 | -1.301 | 1.94E-01 | 4.85E-01 | -5.387 |
| XYLT1 |  | -0.062 | 6.58 | -1.301 | 1.94E-01 | 4.85E-01 | -5.388 |
| BAALC |  | 0.059 | 7.236 | 1.3 | 1.94E-01 | 4.85E-01 | -5.388 |
| SRM |  | -0.062 | 9.024 | -1.299 | 1.95E-01 | 4.86E-01 | -5.39 |
| HPS1 |  | 0.068 | 7.77 | 1.298 | 1.95E-01 | 4.86E-01 | -5.39 |
| EMILIN1 |  | -0.113 | 9.229 | -1.298 | 1.95E-01 | 4.86E-01 | -5.391 |
| TMPRSS5 |  | 0.078 | 5.964 | 1.297 | 1.95E-01 | 4.86E-01 | -5.391 |
| CSNK1G1 |  | -0.043 | 6.704 | -1.298 | 1.95E-01 | 4.86E-01 | -5.391 |
| CHRNA2 |  | -0.072 | 5.248 | -1.298 | 1.95E-01 | 4.86E-01 | -5.392 |
| TDRD5 |  | 0.054 | 4.702 | 1.296 | 1.95E-01 | 4.86E-01 | -5.392 |
| CDH26 |  | -0.067 | 5.614 | -1.297 | 1.96E-01 | 4.87E-01 | -5.393 |
| PRDM11 |  | -0.074 | 6.125 | -1.296 | 1.96E-01 | 4.87E-01 | -5.393 |
| ADRA2C |  | 0.098 | 8.022 | 1.295 | 1.96E-01 | 4.87E-01 | -5.393 |
| PCF11 |  | 0.078 | 7.115 | 1.295 | 1.96E-01 | 4.87E-01 | -5.394 |
| LOXL1 |  | -0.091 | 8.559 | -1.296 | 1.96E-01 | 4.87E-01 | -5.394 |
| KIAA0141 |  | -0.041 | 8.845 | -1.295 | 1.96E-01 | 4.87E-01 | -5.394 |
| CIB1 |  | 0.054 | 10.733 | 1.294 | 1.96E-01 | 4.87E-01 | -5.395 |
| S100Z |  | 0.075 | 6.422 | 1.294 | 1.96E-01 | 4.87E-01 | -5.395 |
| SLITRK1 |  | -0.073 | 4.867 | -1.294 | 1.97E-01 | 4.88E-01 | -5.396 |
| PLEKHB1 |  | 0.073 | 5.808 | 1.293 | 1.97E-01 | 4.88E-01 | -5.397 |
| UBE2D4 |  | -0.055 | 8.373 | -1.293 | 1.97E-01 | 4.88E-01 | -5.397 |
| CXorf21 |  | 0.085 | 6.453 | 1.292 | 1.97E-01 | 4.88E-01 | -5.398 |
| BMPER |  | -0.085 | 5.571 | -1.292 | 1.97E-01 | 4.89E-01 | -5.399 |
| FOXP1 |  | 0.04 | 8.777 | 1.291 | 1.97E-01 | 4.89E-01 | -5.399 |
| NSFL1C |  | -0.058 | 9.267 | -1.291 | 1.98E-01 | 4.89E-01 | -5.4 |
| AGL |  | -0.072 | 8.485 | -1.291 | 1.98E-01 | 4.89E-01 | -5.4 |
| MIA2 |  | 0.061 | 4.661 | 1.29 | 1.98E-01 | 4.89E-01 | -5.4 |
| PC |  | -0.07 | 7.732 | -1.291 | 1.98E-01 | 4.89E-01 | -5.4 |
| CNIH2 |  | 0.067 | 6.332 | 1.289 | 1.98E-01 | 4.89E-01 | -5.401 |
| OSBPL10 |  | 0.068 | 8.355 | 1.289 | 1.98E-01 | 4.89E-01 | -5.401 |
| ATF5 |  | 0.088 | 8.191 | 1.289 | 1.98E-01 | 4.89E-01 | -5.401 |
| STC2 |  | 0.115 | 7.162 | 1.289 | 1.98E-01 | 4.89E-01 | -5.402 |
| NUP43 |  | -0.049 | 8.32 | -1.288 | 1.98E-01 | 4.90E-01 | -5.403 |
| PSEN1 |  | -0.054 | 8.422 | -1.288 | 1.99E-01 | 4.90E-01 | -5.403 |
| YES1 |  | 0.083 | 9.309 | 1.287 | 1.99E-01 | 4.90E-01 | -5.404 |
| TAS2R50 |  | -0.086 | 5.067 | -1.287 | 1.99E-01 | 4.90E-01 | -5.404 |
| KLK6 |  | 0.075 | 5.852 | 1.286 | 1.99E-01 | 4.91E-01 | -5.405 |
| SGCZ |  | -0.06 | 4.565 | -1.286 | 1.99E-01 | 4.91E-01 | -5.406 |
| MAP2K5 |  | -0.05 | 7.193 | -1.286 | 1.99E-01 | 4.91E-01 | -5.406 |
| NTF3 |  | 0.083 | 6.077 | 1.284 | 1.99E-01 | 4.91E-01 | -5.407 |
| CAPZB |  | 0.044 | 9.953 | 1.284 | 2.00E-01 | 4.92E-01 | -5.408 |
| EPN3 |  | -0.088 | 6.766 | -1.285 | 2.00E-01 | 4.92E-01 | -5.408 |
| SCN11A |  | -0.097 | 5.2 | -1.284 | 2.00E-01 | 4.92E-01 | -5.409 |
| DFFB |  | 0.058 | 6.896 | 1.28 | 2.01E-01 | 4.94E-01 | -5.412 |
| PIGQ |  | 0.062 | 8.168 | 1.28 | 2.01E-01 | 4.94E-01 | -5.412 |
| FOXF2 |  | -0.068 | 7.349 | -1.281 | 2.01E-01 | 4.94E-01 | -5.413 |
| HIRA |  | 0.07 | 8.478 | 1.279 | 2.01E-01 | 4.94E-01 | -5.413 |
| MYO5C |  | -0.091 | 6.141 | -1.28 | 2.01E-01 | 4.94E-01 | -5.413 |
| KLF2 |  | 0.074 | 11.356 | 1.279 | 2.01E-01 | 4.94E-01 | -5.414 |
| RGS8 |  | -0.052 | 4.858 | -1.28 | 2.01E-01 | 4.94E-01 | -5.414 |
| BET1L |  | -0.056 | 8.283 | -1.28 | 2.02E-01 | 4.94E-01 | -5.414 |
| RBM23 |  | -0.066 | 9.742 | -1.279 | 2.02E-01 | 4.94E-01 | -5.414 |
| CBS |  | -0.1 | 8.085 | -1.279 | 2.02E-01 | 4.94E-01 | -5.414 |
| UBQLN4 |  | -0.073 | 8.156 | -1.279 | 2.02E-01 | 4.94E-01 | -5.414 |
| BCKDHA |  | 0.067 | 9.089 | 1.277 | 2.02E-01 | 4.94E-01 | -5.415 |
| CRH |  | 0.142 | 10.439 | 1.277 | 2.02E-01 | 4.94E-01 | -5.415 |
| ADPRH |  | -0.094 | 6.987 | -1.278 | 2.02E-01 | 4.94E-01 | -5.416 |
| RANBP6 |  | -0.07 | 7.549 | -1.278 | 2.02E-01 | 4.94E-01 | -5.416 |
| LENEP |  | -0.084 | 5.051 | -1.278 | 2.02E-01 | 4.95E-01 | -5.416 |
| SGPP2 |  | 0.062 | 4.98 | 1.275 | 2.03E-01 | 4.95E-01 | -5.418 |
| DISP2 |  | -0.095 | 6.56 | -1.276 | 2.03E-01 | 4.95E-01 | -5.418 |
| TRIM6 |  | 0.075 | 7.363 | 1.275 | 2.03E-01 | 4.95E-01 | -5.418 |
| MAG |  | 0.089 | 6.245 | 1.275 | 2.03E-01 | 4.95E-01 | -5.418 |
| HOXB2 |  | -0.087 | 7.907 | -1.275 | 2.03E-01 | 4.95E-01 | -5.419 |
| CARD14 |  | -0.065 | 6.188 | -1.275 | 2.03E-01 | 4.95E-01 | -5.419 |
| GUCA1A |  | -0.082 | 5.279 | -1.275 | 2.03E-01 | 4.95E-01 | -5.42 |
| TIMP1 |  | -0.081 | 12.203 | -1.275 | 2.03E-01 | 4.95E-01 | -5.42 |
| DYRK1B |  | -0.075 | 8.14 | -1.275 | 2.03E-01 | 4.95E-01 | -5.42 |
| ZNF407 |  | -0.05 | 6.763 | -1.274 | 2.03E-01 | 4.96E-01 | -5.42 |
| CRNKL1 |  | -0.054 | 7.774 | -1.274 | 2.04E-01 | 4.96E-01 | -5.421 |
| GALC |  | -0.086 | 7.755 | -1.273 | 2.04E-01 | 4.96E-01 | -5.421 |
| NARS |  | -0.073 | 10.888 | -1.273 | 2.04E-01 | 4.96E-01 | -5.422 |
| LY75 |  | -0.077 | 6.483 | -1.273 | 2.04E-01 | 4.96E-01 | -5.422 |
| GOSR2 |  | -0.038 | 8.22 | -1.273 | 2.04E-01 | 4.96E-01 | -5.422 |
| NDUFS1 |  | -0.041 | 9.208 | -1.273 | 2.04E-01 | 4.96E-01 | -5.422 |
| RTKN |  | -0.064 | 6.91 | -1.272 | 2.04E-01 | 4.96E-01 | -5.423 |
| PYY |  | -0.068 | 5.683 | -1.272 | 2.04E-01 | 4.96E-01 | -5.423 |
| GNA12 |  | -0.056 | 8.521 | -1.272 | 2.04E-01 | 4.96E-01 | -5.423 |
| TIRAP |  | 0.044 | 6.523 | 1.271 | 2.04E-01 | 4.96E-01 | -5.424 |
| EWSR1 |  | -0.047 | 9.606 | -1.27 | 2.05E-01 | 4.97E-01 | -5.425 |
| EHD3 |  | 0.081 | 8.131 | 1.269 | 2.05E-01 | 4.97E-01 | -5.425 |
| SH3KBP1 |  | -0.053 | 9.825 | -1.27 | 2.05E-01 | 4.97E-01 | -5.426 |
| CREB5 |  | -0.066 | 8 | -1.269 | 2.05E-01 | 4.98E-01 | -5.426 |
| CPB1 |  | 0.099 | 6.312 | 1.268 | 2.05E-01 | 4.98E-01 | -5.427 |
| CKAP2 |  | 0.056 | 7.595 | 1.268 | 2.05E-01 | 4.98E-01 | -5.427 |
| CLEC11A |  | -0.081 | 8.689 | -1.268 | 2.06E-01 | 4.98E-01 | -5.428 |
| SLC30A10 |  | -0.082 | 4.816 | -1.267 | 2.06E-01 | 4.99E-01 | -5.429 |
| XBP1 |  | -0.059 | 11.647 | -1.267 | 2.06E-01 | 4.99E-01 | -5.429 |
| FMO5 |  | -0.059 | 5.812 | -1.266 | 2.06E-01 | 4.99E-01 | -5.43 |
| HIC2 |  | -0.05 | 8.567 | -1.266 | 2.07E-01 | 5.00E-01 | -5.431 |
| MRPS11 |  | 0.049 | 9.126 | 1.264 | 2.07E-01 | 5.00E-01 | -5.431 |
| CCNE1 |  | 0.072 | 8.889 | 1.264 | 2.07E-01 | 5.00E-01 | -5.432 |
| SCGB3A1 |  | -0.115 | 7.071 | -1.264 | 2.07E-01 | 5.00E-01 | -5.433 |
| MT1E |  | -0.078 | 9.16 | -1.264 | 2.07E-01 | 5.00E-01 | -5.433 |
| CPEB1 |  | 0.076 | 5.415 | 1.263 | 2.07E-01 | 5.01E-01 | -5.433 |
| HSPB2 |  | 0.069 | 7.309 | 1.262 | 2.07E-01 | 5.01E-01 | -5.434 |
| FBXL4 |  | 0.053 | 7.341 | 1.262 | 2.07E-01 | 5.01E-01 | -5.434 |
| DACT1 |  | -0.083 | 7.344 | -1.263 | 2.07E-01 | 5.01E-01 | -5.434 |
| DLG2 |  | -0.061 | 5.302 | -1.263 | 2.08E-01 | 5.01E-01 | -5.434 |
| KCNK16 |  | -0.068 | 5.052 | -1.262 | 2.08E-01 | 5.01E-01 | -5.435 |
| HOXB9 |  | 0.085 | 6.067 | 1.26 | 2.08E-01 | 5.01E-01 | -5.436 |
| NR4A1 |  | 0.092 | 6.306 | 1.26 | 2.08E-01 | 5.02E-01 | -5.437 |
| SFXN2 |  | 0.064 | 6.746 | 1.259 | 2.09E-01 | 5.02E-01 | -5.438 |
| RAB3C |  | 0.079 | 5.049 | 1.259 | 2.09E-01 | 5.02E-01 | -5.438 |
| ZFYVE21 |  | 0.049 | 10.657 | 1.258 | 2.09E-01 | 5.03E-01 | -5.439 |
| GP5 |  | -0.057 | 5.373 | -1.258 | 2.09E-01 | 5.04E-01 | -5.44 |
| ALDH3A2 |  | 0.046 | 7.854 | 1.256 | 2.09E-01 | 5.04E-01 | -5.441 |
| KCNH4 |  | -0.079 | 5.797 | -1.256 | 2.10E-01 | 5.04E-01 | -5.442 |
| GRB10 |  | -0.076 | 8.619 | -1.256 | 2.10E-01 | 5.05E-01 | -5.443 |
| TTF1 |  | 0.054 | 7.585 | 1.255 | 2.10E-01 | 5.05E-01 | -5.443 |
| RAB31 |  | -0.05 | 11.265 | -1.255 | 2.10E-01 | 5.05E-01 | -5.444 |
| MAP1B |  | -0.082 | 7.203 | -1.255 | 2.10E-01 | 5.05E-01 | -5.444 |
| PNOC |  | 0.078 | 5.729 | 1.253 | 2.11E-01 | 5.05E-01 | -5.444 |
| CNOT7 |  | -0.04 | 9.343 | -1.254 | 2.11E-01 | 5.05E-01 | -5.444 |
| ST3GAL3 |  | -0.07 | 6.619 | -1.254 | 2.11E-01 | 5.05E-01 | -5.444 |
| BCAR1 |  | -0.057 | 8.845 | -1.254 | 2.11E-01 | 5.05E-01 | -5.445 |
| DOCK10 |  | -0.073 | 7.39 | -1.254 | 2.11E-01 | 5.05E-01 | -5.445 |
| LGI2 |  | -0.079 | 5.463 | -1.254 | 2.11E-01 | 5.05E-01 | -5.445 |
| APEX2 |  | -0.061 | 8.733 | -1.253 | 2.11E-01 | 5.05E-01 | -5.446 |
| TM9SF1 |  | -0.048 | 9.619 | -1.253 | 2.11E-01 | 5.05E-01 | -5.446 |
| MEGF11 |  | -0.075 | 5.235 | -1.253 | 2.11E-01 | 5.05E-01 | -5.446 |
| SOX6 |  | 0.087 | 5.693 | 1.252 | 2.11E-01 | 5.05E-01 | -5.446 |
| NDP |  | 0.158 | 5.751 | 1.252 | 2.11E-01 | 5.05E-01 | -5.446 |
| SLC2A6 |  | -0.08 | 7.915 | -1.253 | 2.11E-01 | 5.05E-01 | -5.447 |
| TP53RK |  | -0.047 | 7.73 | -1.253 | 2.11E-01 | 5.05E-01 | -5.447 |
| TIAM1 |  | -0.071 | 7.567 | -1.252 | 2.11E-01 | 5.05E-01 | -5.447 |
| CA5A |  | -0.068 | 5.695 | -1.251 | 2.12E-01 | 5.06E-01 | -5.448 |
| DHRS2 |  | -0.112 | 7.264 | -1.251 | 2.12E-01 | 5.06E-01 | -5.449 |
| NPAS1 |  | 0.078 | 5.777 | 1.25 | 2.12E-01 | 5.06E-01 | -5.449 |
| TRPC5 |  | -0.07 | 4.79 | -1.25 | 2.12E-01 | 5.06E-01 | -5.449 |
| ATP5D |  | 0.048 | 10.208 | 1.248 | 2.12E-01 | 5.07E-01 | -5.451 |
| GAL |  | -0.106 | 6.298 | -1.249 | 2.12E-01 | 5.07E-01 | -5.451 |
| SCFD1 |  | 0.058 | 9.267 | 1.248 | 2.13E-01 | 5.07E-01 | -5.451 |
| CYP4F2 |  | -0.072 | 5.711 | -1.249 | 2.13E-01 | 5.07E-01 | -5.451 |
| DNAH8 |  | 0.065 | 4.93 | 1.248 | 2.13E-01 | 5.07E-01 | -5.451 |
| ATCAY |  | -0.078 | 5.793 | -1.248 | 2.13E-01 | 5.07E-01 | -5.452 |
| DNASE1L1 |  | 0.038 | 8.729 | 1.247 | 2.13E-01 | 5.07E-01 | -5.452 |
| GAS7 |  | -0.058 | 7.122 | -1.247 | 2.13E-01 | 5.07E-01 | -5.453 |
| SV2B |  | 0.115 | 6.252 | 1.246 | 2.13E-01 | 5.07E-01 | -5.453 |
| MRPS18C |  | 0.059 | 9.393 | 1.246 | 2.13E-01 | 5.07E-01 | -5.453 |
| PCYT1A |  | -0.044 | 7.357 | -1.247 | 2.13E-01 | 5.07E-01 | -5.454 |
| CYLD |  | 0.053 | 7.597 | 1.245 | 2.13E-01 | 5.07E-01 | -5.454 |
| TSKS |  | 0.118 | 6.899 | 1.245 | 2.13E-01 | 5.07E-01 | -5.454 |
| BZW1 |  | 0.045 | 9.26 | 1.245 | 2.13E-01 | 5.07E-01 | -5.454 |
| IRF1 |  | -0.076 | 9.444 | -1.246 | 2.14E-01 | 5.07E-01 | -5.455 |
| PBOV1 |  | -0.066 | 5.417 | -1.245 | 2.14E-01 | 5.07E-01 | -5.455 |
| DMP1 |  | 0.071 | 4.725 | 1.244 | 2.14E-01 | 5.07E-01 | -5.455 |
| PKN3 |  | 0.077 | 6.922 | 1.244 | 2.14E-01 | 5.07E-01 | -5.456 |
| SLC39A7 |  | 0.081 | 8.927 | 1.244 | 2.14E-01 | 5.07E-01 | -5.456 |
| GPR55 |  | 0.066 | 5.115 | 1.244 | 2.14E-01 | 5.07E-01 | -5.456 |
| DENR |  | -0.048 | 9.329 | -1.245 | 2.14E-01 | 5.07E-01 | -5.456 |
| UVRAG |  | -0.055 | 7.856 | -1.244 | 2.14E-01 | 5.07E-01 | -5.456 |
| TMLHE |  | -0.052 | 7.02 | -1.244 | 2.14E-01 | 5.07E-01 | -5.457 |
| SYP |  | -0.079 | 4.835 | -1.243 | 2.15E-01 | 5.08E-01 | -5.457 |
| RCE1 |  | -0.054 | 7.747 | -1.243 | 2.15E-01 | 5.08E-01 | -5.458 |
| TFPT |  | -0.044 | 9.273 | -1.243 | 2.15E-01 | 5.08E-01 | -5.458 |
| ANKRD10 |  | -0.074 | 8.801 | -1.243 | 2.15E-01 | 5.08E-01 | -5.458 |
| SERPINA6 |  | -0.086 | 6.166 | -1.242 | 2.15E-01 | 5.08E-01 | -5.459 |
| POLE |  | -0.059 | 7.136 | -1.242 | 2.15E-01 | 5.08E-01 | -5.459 |
| BLNK |  | 0.061 | 7.241 | 1.24 | 2.15E-01 | 5.09E-01 | -5.46 |
| NUDT13 |  | -0.072 | 6.309 | -1.24 | 2.16E-01 | 5.09E-01 | -5.461 |
| NASP |  | 0.046 | 9.002 | 1.239 | 2.16E-01 | 5.09E-01 | -5.462 |
| DCTN1 |  | 0.059 | 8.251 | 1.239 | 2.16E-01 | 5.09E-01 | -5.462 |
| CCL13 |  | -0.078 | 6.512 | -1.24 | 2.16E-01 | 5.09E-01 | -5.462 |
| PNPLA2 |  | 0.102 | 8.765 | 1.239 | 2.16E-01 | 5.09E-01 | -5.462 |
| F12 |  | 0.094 | 7.298 | 1.237 | 2.16E-01 | 5.10E-01 | -5.463 |
| PLOD1 |  | 0.072 | 9.67 | 1.237 | 2.16E-01 | 5.10E-01 | -5.463 |
| PPP1R7 |  | 0.044 | 9.773 | 1.237 | 2.16E-01 | 5.10E-01 | -5.464 |
| CHCHD7 |  | 0.043 | 8.821 | 1.236 | 2.17E-01 | 5.11E-01 | -5.465 |
| FBXW2 |  | -0.047 | 7.03 | -1.237 | 2.17E-01 | 5.11E-01 | -5.465 |
| RND3 |  | -0.103 | 8.752 | -1.237 | 2.17E-01 | 5.11E-01 | -5.465 |
| CRBN |  | -0.055 | 9.14 | -1.236 | 2.17E-01 | 5.11E-01 | -5.466 |
| ZNF350 |  | 0.053 | 8.381 | 1.235 | 2.17E-01 | 5.11E-01 | -5.466 |
| GUCY1B2 |  | 0.096 | 5.91 | 1.235 | 2.17E-01 | 5.11E-01 | -5.466 |
| PUS3 |  | -0.053 | 8.491 | -1.236 | 2.17E-01 | 5.11E-01 | -5.466 |
| MYRIP |  | -0.088 | 5.303 | -1.234 | 2.18E-01 | 5.12E-01 | -5.468 |
| SNX1 |  | 0.065 | 8.963 | 1.233 | 2.18E-01 | 5.12E-01 | -5.468 |
| PSAT1 |  | -0.094 | 7.686 | -1.233 | 2.18E-01 | 5.12E-01 | -5.469 |
| DCBLD1 |  | -0.057 | 6.837 | -1.232 | 2.19E-01 | 5.14E-01 | -5.471 |
| MYH7 |  | 0.072 | 5.171 | 1.23 | 2.19E-01 | 5.14E-01 | -5.472 |
| IRX5 |  | 0.1 | 5.236 | 1.23 | 2.19E-01 | 5.14E-01 | -5.472 |
| LAMB1 |  | 0.092 | 9.172 | 1.229 | 2.19E-01 | 5.14E-01 | -5.473 |
| CTNS |  | -0.07 | 7.201 | -1.23 | 2.19E-01 | 5.14E-01 | -5.473 |
| TMEM17 |  | -0.081 | 6.181 | -1.229 | 2.20E-01 | 5.14E-01 | -5.474 |
| LSM2 |  | 0.039 | 10.435 | 1.228 | 2.20E-01 | 5.14E-01 | -5.474 |
| KRT4 |  | -0.077 | 5.855 | -1.229 | 2.20E-01 | 5.14E-01 | -5.474 |
| THBS4 |  | -0.088 | 5.571 | -1.229 | 2.20E-01 | 5.14E-01 | -5.474 |
| MRPL16 |  | 0.044 | 10.16 | 1.226 | 2.21E-01 | 5.16E-01 | -5.477 |
| ADAM15 |  | 0.065 | 9.113 | 1.226 | 2.21E-01 | 5.16E-01 | -5.477 |
| IBSP |  | 0.069 | 5.055 | 1.225 | 2.21E-01 | 5.17E-01 | -5.478 |
| ELSPBP1 |  | 0.069 | 5.445 | 1.224 | 2.22E-01 | 5.18E-01 | -5.48 |
| HIST1H2BB |  | 0.083 | 7.744 | 1.224 | 2.22E-01 | 5.18E-01 | -5.48 |
| DIRAS3 |  | 0.07 | 5.153 | 1.223 | 2.22E-01 | 5.18E-01 | -5.48 |
| MRPL44 |  | -0.057 | 8.672 | -1.224 | 2.22E-01 | 5.18E-01 | -5.48 |
| PKDREJ |  | 0.061 | 4.712 | 1.223 | 2.22E-01 | 5.18E-01 | -5.481 |
| MMRN2 |  | -0.073 | 7.739 | -1.224 | 2.22E-01 | 5.18E-01 | -5.481 |
| ENO1 |  | 0.049 | 12.092 | 1.222 | 2.22E-01 | 5.18E-01 | -5.481 |
| TPPP |  | 0.078 | 5.691 | 1.221 | 2.23E-01 | 5.19E-01 | -5.483 |
| SPG20 |  | 0.064 | 8.018 | 1.221 | 2.23E-01 | 5.19E-01 | -5.483 |
| LHX9 |  | 0.058 | 4.669 | 1.22 | 2.23E-01 | 5.19E-01 | -5.483 |
| DMXL1 |  | 0.065 | 6.606 | 1.22 | 2.23E-01 | 5.19E-01 | -5.484 |
| OXER1 |  | -0.082 | 7.325 | -1.221 | 2.23E-01 | 5.19E-01 | -5.484 |
| MATR3 |  | 0.062 | 9.911 | 1.219 | 2.23E-01 | 5.19E-01 | -5.485 |
| LTB4R |  | 0.055 | 6.981 | 1.219 | 2.23E-01 | 5.19E-01 | -5.485 |
| WDR41 |  | 0.054 | 8.195 | 1.219 | 2.23E-01 | 5.20E-01 | -5.486 |
| KCNK15 |  | -0.077 | 7.226 | -1.219 | 2.23E-01 | 5.20E-01 | -5.486 |
| IL2RG |  | -0.061 | 7.004 | -1.219 | 2.24E-01 | 5.20E-01 | -5.486 |
| DEFA6 |  | -0.06 | 4.786 | -1.219 | 2.24E-01 | 5.20E-01 | -5.487 |
| INPP5D |  | -0.049 | 8.702 | -1.218 | 2.24E-01 | 5.20E-01 | -5.488 |
| S100P |  | -0.084 | 12.698 | -1.218 | 2.24E-01 | 5.20E-01 | -5.488 |
| PRPF18 |  | 0.051 | 8.793 | 1.217 | 2.24E-01 | 5.20E-01 | -5.488 |
| C19orf18 |  | 0.066 | 6.522 | 1.216 | 2.24E-01 | 5.21E-01 | -5.489 |
| POLR2G |  | 0.043 | 10.955 | 1.215 | 2.25E-01 | 5.21E-01 | -5.489 |
| NAPG |  | 0.048 | 8.685 | 1.215 | 2.25E-01 | 5.21E-01 | -5.489 |
| SPTAN1 |  | -0.068 | 10.206 | -1.216 | 2.25E-01 | 5.21E-01 | -5.49 |
| GAS1 |  | -0.118 | 8.55 | -1.215 | 2.25E-01 | 5.22E-01 | -5.491 |
| DNAJC7 |  | 0.048 | 9.794 | 1.214 | 2.25E-01 | 5.22E-01 | -5.491 |
| WDR48 |  | -0.056 | 9.103 | -1.214 | 2.25E-01 | 5.22E-01 | -5.492 |
| COL16A1 |  | 0.076 | 7.847 | 1.213 | 2.26E-01 | 5.22E-01 | -5.492 |
| PER2 |  | -0.063 | 7.033 | -1.212 | 2.26E-01 | 5.23E-01 | -5.494 |
| LCMT1 |  | 0.044 | 9.832 | 1.211 | 2.26E-01 | 5.23E-01 | -5.494 |
| TFAP2C |  | -0.091 | 7.663 | -1.212 | 2.26E-01 | 5.24E-01 | -5.495 |
| WDR37 |  | -0.048 | 8.482 | -1.211 | 2.27E-01 | 5.24E-01 | -5.495 |
| ACRBP |  | -0.066 | 6.806 | -1.211 | 2.27E-01 | 5.24E-01 | -5.495 |
| C19orf24 |  | 0.053 | 9.123 | 1.21 | 2.27E-01 | 5.24E-01 | -5.495 |
| SPATA5L1 |  | 0.042 | 7.976 | 1.21 | 2.27E-01 | 5.24E-01 | -5.496 |
| SPAG9 |  | -0.08 | 7.904 | -1.21 | 2.27E-01 | 5.24E-01 | -5.497 |
| CLDN17 |  | -0.069 | 5.08 | -1.209 | 2.27E-01 | 5.25E-01 | -5.497 |
| ZNF473 |  | 0.057 | 6.462 | 1.208 | 2.27E-01 | 5.25E-01 | -5.498 |
| SLCO1A2 |  | -0.059 | 4.906 | -1.209 | 2.27E-01 | 5.25E-01 | -5.498 |
| CBLN2 |  | 0.075 | 5.079 | 1.208 | 2.28E-01 | 5.25E-01 | -5.498 |
| MFAP2 |  | 0.098 | 8.84 | 1.207 | 2.28E-01 | 5.25E-01 | -5.499 |
| WDTC1 |  | -0.083 | 7.086 | -1.208 | 2.28E-01 | 5.25E-01 | -5.499 |
| COMMD5 |  | -0.036 | 8.202 | -1.208 | 2.28E-01 | 5.25E-01 | -5.499 |
| NXPH4 |  | -0.081 | 6.74 | -1.208 | 2.28E-01 | 5.25E-01 | -5.499 |
| GAN |  | -0.054 | 6.435 | -1.207 | 2.28E-01 | 5.25E-01 | -5.5 |
| SH3BP4 |  | 0.071 | 8.62 | 1.206 | 2.28E-01 | 5.26E-01 | -5.501 |
| TIMELESS |  | -0.05 | 8.313 | -1.206 | 2.29E-01 | 5.26E-01 | -5.501 |
| PARP1 |  | -0.048 | 10.364 | -1.206 | 2.29E-01 | 5.26E-01 | -5.502 |
| PROX1 |  | -0.063 | 4.884 | -1.205 | 2.29E-01 | 5.26E-01 | -5.502 |
| CTSD |  | 0.071 | 10.49 | 1.203 | 2.29E-01 | 5.27E-01 | -5.503 |
| SLC5A8 |  | 0.08 | 5.125 | 1.202 | 2.30E-01 | 5.28E-01 | -5.505 |
| CLDN14 |  | -0.067 | 6.524 | -1.203 | 2.30E-01 | 5.28E-01 | -5.505 |
| DUSP23 |  | 0.065 | 10.008 | 1.201 | 2.30E-01 | 5.28E-01 | -5.506 |
| GP9 |  | -0.066 | 7.596 | -1.202 | 2.30E-01 | 5.28E-01 | -5.506 |
| CTNNA1 |  | 0.047 | 10.956 | 1.201 | 2.30E-01 | 5.28E-01 | -5.506 |
| NAV1 |  | 0.068 | 7.292 | 1.2 | 2.30E-01 | 5.28E-01 | -5.507 |
| SLBP |  | -0.047 | 10.343 | -1.201 | 2.31E-01 | 5.28E-01 | -5.507 |
| FETUB |  | -0.076 | 5.296 | -1.201 | 2.31E-01 | 5.28E-01 | -5.507 |
| OSBP2 |  | -0.089 | 6.906 | -1.201 | 2.31E-01 | 5.28E-01 | -5.508 |
| KRT5 |  | 0.124 | 5.119 | 1.199 | 2.31E-01 | 5.28E-01 | -5.508 |
| MT3 |  | -0.065 | 7.247 | -1.2 | 2.31E-01 | 5.28E-01 | -5.508 |
| REPS1 |  | 0.04 | 9.088 | 1.199 | 2.31E-01 | 5.28E-01 | -5.508 |
| TMEM9 |  | 0.051 | 8.648 | 1.198 | 2.31E-01 | 5.29E-01 | -5.509 |
| RNF157 |  | -0.073 | 5.619 | -1.199 | 2.31E-01 | 5.29E-01 | -5.509 |
| CYP2C18 |  | -0.062 | 4.863 | -1.198 | 2.32E-01 | 5.30E-01 | -5.51 |
| CBR4 |  | 0.054 | 8.026 | 1.197 | 2.32E-01 | 5.30E-01 | -5.51 |
| RBL1 |  | -0.058 | 6.012 | -1.198 | 2.32E-01 | 5.30E-01 | -5.511 |
| CD109 |  | 0.08 | 6.711 | 1.196 | 2.32E-01 | 5.30E-01 | -5.511 |
| FOXJ3 |  | 0.063 | 10.721 | 1.196 | 2.32E-01 | 5.31E-01 | -5.512 |
| F3 |  | -0.094 | 8.134 | -1.196 | 2.32E-01 | 5.31E-01 | -5.513 |
| SMOX |  | -0.088 | 8.363 | -1.196 | 2.33E-01 | 5.31E-01 | -5.513 |
| ID1 |  | 0.077 | 10.02 | 1.195 | 2.33E-01 | 5.31E-01 | -5.513 |
| NUDCD2 |  | 0.059 | 9.457 | 1.195 | 2.33E-01 | 5.31E-01 | -5.513 |
| SLC23A1 |  | 0.065 | 5.099 | 1.194 | 2.33E-01 | 5.31E-01 | -5.514 |
| TAF1B |  | 0.048 | 8.167 | 1.194 | 2.33E-01 | 5.31E-01 | -5.514 |
| GADD45GIP1 |  | 0.049 | 9.322 | 1.194 | 2.33E-01 | 5.31E-01 | -5.514 |
| ASPH |  | -0.061 | 8.148 | -1.194 | 2.33E-01 | 5.31E-01 | -5.515 |
| RGAG1 |  | 0.064 | 5.44 | 1.193 | 2.33E-01 | 5.31E-01 | -5.515 |
| DPAGT1 |  | -0.078 | 8.41 | -1.194 | 2.33E-01 | 5.31E-01 | -5.515 |
| SPACA3 |  | -0.074 | 6.412 | -1.194 | 2.33E-01 | 5.31E-01 | -5.515 |
| FANCC |  | -0.048 | 7.078 | -1.194 | 2.33E-01 | 5.31E-01 | -5.516 |
| RFC5 |  | -0.061 | 7.917 | -1.193 | 2.33E-01 | 5.31E-01 | -5.516 |
| TBP |  | -0.037 | 8.824 | -1.193 | 2.34E-01 | 5.31E-01 | -5.516 |
| F2RL2 |  | 0.083 | 5.388 | 1.192 | 2.34E-01 | 5.31E-01 | -5.516 |
| SHMT2 |  | 0.053 | 9.858 | 1.192 | 2.34E-01 | 5.31E-01 | -5.516 |
| MUM1 |  | 0.052 | 8.263 | 1.192 | 2.34E-01 | 5.31E-01 | -5.516 |
| ARRB1 |  | -0.062 | 8.304 | -1.192 | 2.34E-01 | 5.31E-01 | -5.517 |
| ISYNA1 |  | 0.061 | 10.063 | 1.191 | 2.34E-01 | 5.31E-01 | -5.517 |
| ZDHHC19 |  | -0.069 | 5.459 | -1.192 | 2.34E-01 | 5.31E-01 | -5.517 |
| CHD4 |  | 0.041 | 9.878 | 1.191 | 2.34E-01 | 5.31E-01 | -5.518 |
| AGTR2 |  | 0.062 | 4.784 | 1.19 | 2.35E-01 | 5.32E-01 | -5.519 |
| MRVI1 |  | 0.085 | 8.137 | 1.188 | 2.35E-01 | 5.33E-01 | -5.521 |
| PLSCR1 |  | -0.09 | 9.526 | -1.189 | 2.35E-01 | 5.33E-01 | -5.521 |
| GPR6 |  | 0.062 | 5.828 | 1.188 | 2.35E-01 | 5.33E-01 | -5.521 |
| SERPINF1 |  | -0.061 | 10.363 | -1.189 | 2.35E-01 | 5.33E-01 | -5.521 |
| ITGB1 |  | -0.048 | 10.325 | -1.188 | 2.36E-01 | 5.33E-01 | -5.522 |
| TAP2 |  | -0.067 | 6.492 | -1.188 | 2.36E-01 | 5.33E-01 | -5.522 |
| NAP1L1 |  | 0.065 | 9.394 | 1.186 | 2.36E-01 | 5.33E-01 | -5.523 |
| KIAA0232 |  | 0.065 | 8.486 | 1.186 | 2.36E-01 | 5.33E-01 | -5.523 |
| GALNT6 |  | 0.087 | 7.66 | 1.186 | 2.36E-01 | 5.33E-01 | -5.523 |
| LAMP3 |  | -0.094 | 6.713 | -1.187 | 2.36E-01 | 5.33E-01 | -5.523 |
| KCNA1 |  | 0.049 | 4.723 | 1.185 | 2.36E-01 | 5.34E-01 | -5.524 |
| CRIM1 |  | -0.068 | 8.669 | -1.186 | 2.36E-01 | 5.34E-01 | -5.524 |
| RPS15A |  | 0.048 | 13.397 | 1.184 | 2.37E-01 | 5.34E-01 | -5.525 |
| NELL1 |  | -0.061 | 5.158 | -1.185 | 2.37E-01 | 5.34E-01 | -5.525 |
| PIGF |  | 0.045 | 8.765 | 1.184 | 2.37E-01 | 5.34E-01 | -5.526 |
| TRAP1 |  | -0.045 | 8.914 | -1.185 | 2.37E-01 | 5.34E-01 | -5.526 |
| TNK2 |  | 0.05 | 8.172 | 1.184 | 2.37E-01 | 5.34E-01 | -5.526 |
| GPC4 |  | 0.079 | 8.32 | 1.184 | 2.37E-01 | 5.34E-01 | -5.526 |
| SDCBP |  | -0.063 | 11.149 | -1.184 | 2.37E-01 | 5.34E-01 | -5.527 |
| CYP2A7 |  | -0.085 | 5.128 | -1.184 | 2.37E-01 | 5.34E-01 | -5.527 |
| ADCK4 |  | 0.067 | 6.558 | 1.182 | 2.38E-01 | 5.35E-01 | -5.528 |
| HS3ST1 |  | 0.092 | 6.374 | 1.182 | 2.38E-01 | 5.35E-01 | -5.528 |
| CS |  | -0.045 | 8.801 | -1.183 | 2.38E-01 | 5.35E-01 | -5.528 |
| BLCAP |  | 0.061 | 8.989 | 1.181 | 2.38E-01 | 5.35E-01 | -5.529 |
| LCE1B |  | 0.077 | 4.94 | 1.181 | 2.38E-01 | 5.35E-01 | -5.529 |
| GDI2 |  | 0.055 | 10.753 | 1.181 | 2.38E-01 | 5.36E-01 | -5.529 |
| CGREF1 |  | 0.073 | 5.48 | 1.18 | 2.38E-01 | 5.36E-01 | -5.53 |
| APEX1 |  | -0.047 | 10.02 | -1.18 | 2.39E-01 | 5.36E-01 | -5.531 |
| MAGEB6 |  | -0.065 | 4.695 | -1.18 | 2.39E-01 | 5.36E-01 | -5.531 |
| TBX21 |  | 0.081 | 6.546 | 1.179 | 2.39E-01 | 5.36E-01 | -5.531 |
| ITGA2 |  | -0.075 | 5.666 | -1.179 | 2.39E-01 | 5.36E-01 | -5.532 |
| WDR44 |  | 0.048 | 7.666 | 1.178 | 2.39E-01 | 5.36E-01 | -5.532 |
| MXI1 |  | 0.074 | 8.097 | 1.178 | 2.39E-01 | 5.36E-01 | -5.532 |
| ATP6V1C1 |  | 0.06 | 8.851 | 1.178 | 2.39E-01 | 5.36E-01 | -5.532 |
| TNFRSF10B |  | 0.055 | 8.681 | 1.177 | 2.39E-01 | 5.37E-01 | -5.533 |
| ZDHHC6 |  | 0.047 | 8.591 | 1.177 | 2.40E-01 | 5.37E-01 | -5.533 |
| LRRC17 |  | 0.079 | 5.337 | 1.177 | 2.40E-01 | 5.37E-01 | -5.533 |
| ABI1 |  | -0.045 | 8.545 | -1.178 | 2.40E-01 | 5.37E-01 | -5.533 |
| PLOD3 |  | 0.061 | 10.265 | 1.176 | 2.40E-01 | 5.37E-01 | -5.534 |
| CYP4X1 |  | -0.082 | 5.804 | -1.177 | 2.40E-01 | 5.37E-01 | -5.535 |
| LILRA2 |  | -0.072 | 6.567 | -1.177 | 2.40E-01 | 5.37E-01 | -5.535 |
| ACIN1 |  | 0.055 | 9.324 | 1.176 | 2.40E-01 | 5.37E-01 | -5.535 |
| RASSF5 |  | -0.046 | 6.776 | -1.176 | 2.40E-01 | 5.38E-01 | -5.536 |
| MARK2 |  | -0.047 | 7.875 | -1.175 | 2.41E-01 | 5.38E-01 | -5.536 |
| PSMD14 |  | -0.057 | 10.538 | -1.175 | 2.41E-01 | 5.38E-01 | -5.537 |
| AHR |  | -0.076 | 10.75 | -1.175 | 2.41E-01 | 5.38E-01 | -5.537 |
| TIMM8A |  | 0.047 | 7.506 | 1.174 | 2.41E-01 | 5.38E-01 | -5.537 |
| PPIL2 |  | 0.06 | 7.025 | 1.173 | 2.41E-01 | 5.38E-01 | -5.538 |
| CDK6 |  | 0.054 | 9.26 | 1.173 | 2.41E-01 | 5.38E-01 | -5.538 |
| ALG1 |  | -0.073 | 7.504 | -1.174 | 2.41E-01 | 5.38E-01 | -5.538 |
| HSPB8 |  | -0.081 | 9.089 | -1.174 | 2.41E-01 | 5.38E-01 | -5.538 |
| KIAA1524 |  | -0.075 | 5.486 | -1.173 | 2.42E-01 | 5.39E-01 | -5.539 |
| EFNA4 |  | 0.056 | 7.753 | 1.171 | 2.42E-01 | 5.39E-01 | -5.54 |
| PRKAG3 |  | 0.083 | 5.758 | 1.171 | 2.42E-01 | 5.39E-01 | -5.54 |
| RB1 |  | -0.068 | 9.267 | -1.171 | 2.42E-01 | 5.40E-01 | -5.541 |
| SURF6 |  | 0.062 | 7.569 | 1.17 | 2.42E-01 | 5.40E-01 | -5.541 |
| OPRD1 |  | -0.082 | 5.81 | -1.171 | 2.42E-01 | 5.40E-01 | -5.541 |
| SLAMF9 |  | 0.063 | 6.647 | 1.17 | 2.43E-01 | 5.40E-01 | -5.542 |
| BMP10 |  | 0.064 | 4.969 | 1.169 | 2.43E-01 | 5.40E-01 | -5.542 |
| VPS18 |  | 0.079 | 9.454 | 1.168 | 2.43E-01 | 5.41E-01 | -5.543 |
| LPAL2 |  | -0.054 | 4.816 | -1.169 | 2.43E-01 | 5.41E-01 | -5.544 |
| PFDN4 |  | 0.079 | 8.215 | 1.168 | 2.43E-01 | 5.41E-01 | -5.544 |
| MANBA |  | -0.059 | 9.175 | -1.168 | 2.44E-01 | 5.41E-01 | -5.544 |
| CHI3L2 |  | 0.114 | 6.637 | 1.167 | 2.44E-01 | 5.41E-01 | -5.544 |
| ZBTB37 |  | 0.056 | 5.89 | 1.167 | 2.44E-01 | 5.41E-01 | -5.545 |
| CDH10 |  | 0.063 | 4.876 | 1.166 | 2.44E-01 | 5.42E-01 | -5.546 |
| KCTD5 |  | -0.043 | 8.728 | -1.166 | 2.44E-01 | 5.42E-01 | -5.547 |
| DDX24 |  | 0.054 | 9.178 | 1.165 | 2.45E-01 | 5.43E-01 | -5.547 |
| FASTK |  | 0.047 | 9.905 | 1.164 | 2.45E-01 | 5.43E-01 | -5.548 |
| NOL7 |  | 0.042 | 11.678 | 1.163 | 2.45E-01 | 5.43E-01 | -5.549 |
| SPAM1 |  | -0.06 | 4.858 | -1.164 | 2.45E-01 | 5.43E-01 | -5.549 |
| HES5 |  | 0.07 | 6.859 | 1.163 | 2.45E-01 | 5.43E-01 | -5.549 |
| FGF19 |  | -0.054 | 5.565 | -1.164 | 2.45E-01 | 5.43E-01 | -5.549 |
| CHRND |  | -0.071 | 5.352 | -1.164 | 2.45E-01 | 5.43E-01 | -5.549 |
| YTHDF1 |  | 0.038 | 10.107 | 1.163 | 2.45E-01 | 5.43E-01 | -5.549 |
| CENPF |  | 0.071 | 7.797 | 1.162 | 2.45E-01 | 5.43E-01 | -5.55 |
| ITLN1 |  | -0.088 | 6.638 | -1.163 | 2.46E-01 | 5.44E-01 | -5.551 |
| GCM1 |  | -0.092 | 8.497 | -1.161 | 2.46E-01 | 5.45E-01 | -5.552 |
| HS3ST4 |  | 0.053 | 5.31 | 1.16 | 2.47E-01 | 5.45E-01 | -5.553 |
| PAK6 |  | 0.067 | 7.393 | 1.16 | 2.47E-01 | 5.45E-01 | -5.553 |
| C2 |  | -0.07 | 8.034 | -1.161 | 2.47E-01 | 5.45E-01 | -5.553 |
| RNPS1 |  | -0.036 | 9.878 | -1.16 | 2.47E-01 | 5.45E-01 | -5.553 |
| BRD9 |  | 0.039 | 8.201 | 1.159 | 2.47E-01 | 5.45E-01 | -5.553 |
| ALG3 |  | -0.054 | 9.377 | -1.16 | 2.47E-01 | 5.45E-01 | -5.554 |
| NUBPL |  | 0.053 | 7.364 | 1.159 | 2.47E-01 | 5.45E-01 | -5.554 |
| CAP1 |  | -0.044 | 11.949 | -1.159 | 2.47E-01 | 5.45E-01 | -5.554 |
| EGFR |  | 0.059 | 8.522 | 1.158 | 2.47E-01 | 5.45E-01 | -5.555 |
| PRDM4 |  | 0.036 | 9.161 | 1.157 | 2.47E-01 | 5.45E-01 | -5.555 |
| MMP16 |  | -0.053 | 5.118 | -1.158 | 2.48E-01 | 5.46E-01 | -5.556 |
| SOX15 |  | 0.066 | 5.546 | 1.157 | 2.48E-01 | 5.46E-01 | -5.556 |
| TEP1 |  | -0.052 | 7.323 | -1.157 | 2.48E-01 | 5.46E-01 | -5.556 |
| B3GALNT2 |  | 0.048 | 7.356 | 1.156 | 2.48E-01 | 5.46E-01 | -5.557 |
| GALNT12 |  | 0.06 | 6.232 | 1.154 | 2.49E-01 | 5.47E-01 | -5.559 |
| LHFPL5 |  | -0.075 | 6.026 | -1.155 | 2.49E-01 | 5.47E-01 | -5.559 |
| CCL20 |  | -0.098 | 5.341 | -1.155 | 2.49E-01 | 5.48E-01 | -5.559 |
| CYB561D2 |  | 0.046 | 8.962 | 1.153 | 2.49E-01 | 5.48E-01 | -5.56 |
| MMP9 |  | -0.097 | 8.324 | -1.154 | 2.49E-01 | 5.48E-01 | -5.56 |
| GMFB |  | 0.064 | 8.935 | 1.152 | 2.50E-01 | 5.48E-01 | -5.561 |
| TUSC2 |  | 0.032 | 9.289 | 1.152 | 2.50E-01 | 5.48E-01 | -5.561 |
| C6orf1 |  | -0.043 | 9.298 | -1.153 | 2.50E-01 | 5.48E-01 | -5.562 |
| BCKDK |  | 0.044 | 10.177 | 1.151 | 2.50E-01 | 5.48E-01 | -5.562 |
| ATP2C1 |  | -0.051 | 8.618 | -1.152 | 2.50E-01 | 5.48E-01 | -5.562 |
| SYTL1 |  | 0.063 | 7.45 | 1.151 | 2.50E-01 | 5.49E-01 | -5.562 |
| SLAMF6 |  | -0.064 | 5.365 | -1.151 | 2.50E-01 | 5.49E-01 | -5.563 |
| RREB1 |  | -0.045 | 6.647 | -1.151 | 2.51E-01 | 5.49E-01 | -5.564 |
| NOG |  | 0.123 | 7.457 | 1.149 | 2.51E-01 | 5.50E-01 | -5.564 |
| TBC1D4 |  | 0.055 | 8.299 | 1.149 | 2.51E-01 | 5.50E-01 | -5.565 |
| GNGT2 |  | 0.065 | 6.075 | 1.149 | 2.51E-01 | 5.50E-01 | -5.565 |
| RBM24 |  | -0.074 | 5.659 | -1.15 | 2.51E-01 | 5.50E-01 | -5.565 |
| DIO1 |  | -0.069 | 5.093 | -1.149 | 2.51E-01 | 5.50E-01 | -5.566 |
| FAM19A4 |  | -0.088 | 4.883 | -1.149 | 2.51E-01 | 5.50E-01 | -5.566 |
| ZNF222 |  | -0.064 | 6.689 | -1.149 | 2.51E-01 | 5.50E-01 | -5.566 |
| SLC12A7 |  | -0.059 | 7.867 | -1.149 | 2.51E-01 | 5.50E-01 | -5.566 |
| SSBP1 |  | -0.055 | 10.23 | -1.149 | 2.51E-01 | 5.50E-01 | -5.566 |
| ITGB7 |  | -0.069 | 6.912 | -1.148 | 2.52E-01 | 5.50E-01 | -5.566 |
| THRAP3 |  | 0.042 | 9.046 | 1.147 | 2.52E-01 | 5.50E-01 | -5.566 |
| PDE4DIP |  | -0.046 | 7.609 | -1.148 | 2.52E-01 | 5.50E-01 | -5.567 |
| SLC4A10 |  | -0.091 | 5.225 | -1.148 | 2.52E-01 | 5.50E-01 | -5.567 |
| ARHGAP21 |  | 0.046 | 8.902 | 1.145 | 2.52E-01 | 5.51E-01 | -5.568 |
| DYX1C1 |  | 0.064 | 5.882 | 1.145 | 2.53E-01 | 5.51E-01 | -5.569 |
| FKBP5 |  | -0.108 | 7.828 | -1.146 | 2.53E-01 | 5.51E-01 | -5.569 |
| ZNF559 |  | 0.08 | 7.326 | 1.145 | 2.53E-01 | 5.51E-01 | -5.569 |
| ZNF485 |  | -0.069 | 5.794 | -1.145 | 2.53E-01 | 5.51E-01 | -5.57 |
| SLITRK3 |  | -0.069 | 4.709 | -1.144 | 2.53E-01 | 5.52E-01 | -5.571 |
| CLK2 |  | -0.069 | 7.725 | -1.144 | 2.53E-01 | 5.52E-01 | -5.571 |
| NFE2L3 |  | -0.091 | 9.038 | -1.143 | 2.54E-01 | 5.53E-01 | -5.572 |
| RIMS4 |  | -0.068 | 5.826 | -1.143 | 2.54E-01 | 5.53E-01 | -5.572 |
| HIST1H1T |  | -0.091 | 5.467 | -1.143 | 2.54E-01 | 5.53E-01 | -5.573 |
| FKBP14 |  | 0.058 | 8.004 | 1.141 | 2.54E-01 | 5.53E-01 | -5.573 |
| MITF |  | 0.049 | 6.848 | 1.141 | 2.54E-01 | 5.53E-01 | -5.574 |
| NIT1 |  | -0.034 | 8.532 | -1.142 | 2.54E-01 | 5.53E-01 | -5.574 |
| SFTPB |  | 0.053 | 5.287 | 1.14 | 2.55E-01 | 5.53E-01 | -5.574 |
| SOST |  | 0.08 | 5.356 | 1.14 | 2.55E-01 | 5.53E-01 | -5.575 |
| SMAD6 |  | 0.079 | 8.305 | 1.14 | 2.55E-01 | 5.53E-01 | -5.575 |
| PYCARD |  | -0.066 | 9.807 | -1.14 | 2.55E-01 | 5.53E-01 | -5.575 |
| CYTL1 |  | -0.092 | 7.543 | -1.14 | 2.55E-01 | 5.53E-01 | -5.575 |
| RNF130 |  | -0.048 | 10.065 | -1.14 | 2.55E-01 | 5.53E-01 | -5.575 |
| RPAP1 |  | 0.054 | 8.414 | 1.139 | 2.55E-01 | 5.53E-01 | -5.575 |
| C9orf40 |  | 0.07 | 7.812 | 1.139 | 2.55E-01 | 5.53E-01 | -5.575 |
| BCAS1 |  | 0.069 | 4.918 | 1.138 | 2.55E-01 | 5.53E-01 | -5.576 |
| NUP37 |  | 0.044 | 9.462 | 1.138 | 2.55E-01 | 5.53E-01 | -5.576 |
| OR4D2 |  | -0.067 | 5.988 | -1.139 | 2.55E-01 | 5.53E-01 | -5.576 |
| CDC42BPB |  | -0.047 | 9.655 | -1.139 | 2.56E-01 | 5.53E-01 | -5.577 |
| PARP9 |  | 0.061 | 8.887 | 1.137 | 2.56E-01 | 5.53E-01 | -5.577 |
| RAG1 |  | 0.068 | 5.399 | 1.137 | 2.56E-01 | 5.53E-01 | -5.577 |
| AHI1 |  | -0.06 | 6.846 | -1.138 | 2.56E-01 | 5.53E-01 | -5.577 |
| CUTC |  | 0.046 | 8.519 | 1.137 | 2.56E-01 | 5.53E-01 | -5.578 |
| RAB8B |  | 0.059 | 9.05 | 1.137 | 2.56E-01 | 5.53E-01 | -5.578 |
| FKBP6 |  | -0.067 | 5.029 | -1.137 | 2.56E-01 | 5.54E-01 | -5.578 |
| NPAS2 |  | -0.063 | 6.936 | -1.137 | 2.56E-01 | 5.54E-01 | -5.579 |
| DCX |  | -0.08 | 5.797 | -1.137 | 2.56E-01 | 5.54E-01 | -5.579 |
| LRRC20 |  | 0.067 | 6.876 | 1.136 | 2.56E-01 | 5.54E-01 | -5.579 |
| DSCR9 |  | 0.073 | 4.983 | 1.136 | 2.56E-01 | 5.54E-01 | -5.579 |
| NUP205 |  | -0.043 | 9.911 | -1.136 | 2.57E-01 | 5.54E-01 | -5.579 |
| PCCB |  | -0.05 | 9.33 | -1.136 | 2.57E-01 | 5.54E-01 | -5.579 |
| XAB2 |  | -0.075 | 7.331 | -1.135 | 2.57E-01 | 5.55E-01 | -5.581 |
| HSF2 |  | -0.055 | 8.179 | -1.134 | 2.57E-01 | 5.55E-01 | -5.582 |
| CCNI |  | 0.051 | 13.134 | 1.133 | 2.57E-01 | 5.55E-01 | -5.582 |
| C4BPB |  | 0.083 | 6.792 | 1.133 | 2.58E-01 | 5.55E-01 | -5.582 |
| CPA2 |  | 0.064 | 5.067 | 1.132 | 2.58E-01 | 5.56E-01 | -5.583 |
| UNC119 |  | -0.051 | 8.06 | -1.133 | 2.58E-01 | 5.56E-01 | -5.583 |
| SLC17A3 |  | 0.064 | 5.127 | 1.132 | 2.58E-01 | 5.56E-01 | -5.583 |
| POMC |  | 0.089 | 7.183 | 1.131 | 2.58E-01 | 5.56E-01 | -5.584 |
| SERTAD3 |  | -0.059 | 8.676 | -1.132 | 2.58E-01 | 5.56E-01 | -5.584 |
| RLN1 |  | 0.071 | 5.216 | 1.13 | 2.59E-01 | 5.56E-01 | -5.585 |
| ZNF563 |  | 0.063 | 5.587 | 1.13 | 2.59E-01 | 5.56E-01 | -5.585 |
| SRGAP1 |  | 0.061 | 6.802 | 1.13 | 2.59E-01 | 5.56E-01 | -5.585 |
| OTOP2 |  | 0.071 | 5.861 | 1.129 | 2.59E-01 | 5.57E-01 | -5.586 |
| CXXC4 |  | -0.079 | 5.966 | -1.13 | 2.59E-01 | 5.57E-01 | -5.586 |
| IMPDH1 |  | -0.048 | 9.414 | -1.129 | 2.59E-01 | 5.57E-01 | -5.587 |
| CAPZA3 |  | -0.067 | 4.691 | -1.129 | 2.60E-01 | 5.57E-01 | -5.587 |
| DST |  | -0.05 | 6.6 | -1.129 | 2.60E-01 | 5.57E-01 | -5.587 |
| ZNF148 |  | -0.042 | 9.203 | -1.129 | 2.60E-01 | 5.57E-01 | -5.587 |
| NFX1 |  | -0.032 | 7.928 | -1.129 | 2.60E-01 | 5.57E-01 | -5.588 |
| TCEB3B |  | -0.061 | 4.769 | -1.129 | 2.60E-01 | 5.57E-01 | -5.588 |
| ARHGDIB |  | -0.073 | 11.985 | -1.129 | 2.60E-01 | 5.57E-01 | -5.588 |
| FBXL8 |  | 0.05 | 6.801 | 1.127 | 2.60E-01 | 5.58E-01 | -5.589 |
| NUPL2 |  | 0.047 | 8.295 | 1.126 | 2.60E-01 | 5.58E-01 | -5.589 |
| PLCB2 |  | -0.06 | 7.18 | -1.127 | 2.61E-01 | 5.58E-01 | -5.59 |
| CDKN2C |  | -0.059 | 7.923 | -1.127 | 2.61E-01 | 5.58E-01 | -5.59 |
| MTHFD1L |  | 0.057 | 7.88 | 1.126 | 2.61E-01 | 5.58E-01 | -5.59 |
| DOLPP1 |  | -0.065 | 8.285 | -1.127 | 2.61E-01 | 5.58E-01 | -5.59 |
| SLC25A23 |  | -0.054 | 8.259 | -1.127 | 2.61E-01 | 5.58E-01 | -5.59 |
| COPS2 |  | 0.058 | 9.01 | 1.125 | 2.61E-01 | 5.58E-01 | -5.59 |
| API5 |  | -0.042 | 9.089 | -1.126 | 2.61E-01 | 5.58E-01 | -5.591 |
| KIAA0513 |  | 0.06 | 8.127 | 1.124 | 2.61E-01 | 5.58E-01 | -5.591 |
| HOOK2 |  | 0.051 | 8.797 | 1.124 | 2.61E-01 | 5.58E-01 | -5.591 |
| CST9L |  | 0.054 | 4.722 | 1.124 | 2.61E-01 | 5.58E-01 | -5.592 |
| ATP1B3 |  | -0.06 | 10.851 | -1.125 | 2.62E-01 | 5.58E-01 | -5.592 |
| DAO |  | -0.06 | 5.027 | -1.125 | 2.62E-01 | 5.58E-01 | -5.592 |
| CAPZA1 |  | -0.089 | 9.089 | -1.124 | 2.62E-01 | 5.59E-01 | -5.593 |
| MSI2 |  | 0.053 | 8.021 | 1.122 | 2.62E-01 | 5.59E-01 | -5.594 |
| SSRP1 |  | 0.046 | 11.214 | 1.122 | 2.62E-01 | 5.59E-01 | -5.594 |
| AP2B1 |  | 0.062 | 9.024 | 1.122 | 2.62E-01 | 5.59E-01 | -5.594 |
| ECHDC1 |  | -0.047 | 8.279 | -1.121 | 2.63E-01 | 5.60E-01 | -5.596 |
| XPNPEP2 |  | -0.068 | 5.929 | -1.121 | 2.63E-01 | 5.60E-01 | -5.596 |
| MMP17 |  | 0.09 | 7.377 | 1.12 | 2.63E-01 | 5.60E-01 | -5.596 |
| MRPL30 |  | 0.037 | 8.466 | 1.12 | 2.63E-01 | 5.60E-01 | -5.596 |
| HERC6 |  | 0.076 | 7.094 | 1.12 | 2.63E-01 | 5.60E-01 | -5.596 |
| NOVA1 |  | 0.055 | 5.324 | 1.12 | 2.63E-01 | 5.60E-01 | -5.596 |
| CLPX |  | -0.039 | 9.2 | -1.121 | 2.63E-01 | 5.60E-01 | -5.596 |
| MYCBP2 |  | -0.047 | 9.221 | -1.121 | 2.63E-01 | 5.60E-01 | -5.597 |
| SLC6A18 |  | 0.077 | 6.25 | 1.119 | 2.64E-01 | 5.60E-01 | -5.597 |
| LYAR |  | 0.05 | 8.915 | 1.119 | 2.64E-01 | 5.60E-01 | -5.598 |
| LILRB1 |  | -0.073 | 6.916 | -1.12 | 2.64E-01 | 5.60E-01 | -5.598 |
| TPM1 |  | 0.075 | 9.855 | 1.118 | 2.64E-01 | 5.60E-01 | -5.598 |
| SSBP2 |  | -0.064 | 8.543 | -1.119 | 2.64E-01 | 5.61E-01 | -5.598 |
| NBEA |  | -0.074 | 6.056 | -1.118 | 2.64E-01 | 5.61E-01 | -5.599 |
| BAIAP2L2 |  | 0.061 | 6.616 | 1.116 | 2.65E-01 | 5.62E-01 | -5.6 |
| GNG11 |  | -0.06 | 12.315 | -1.117 | 2.65E-01 | 5.62E-01 | -5.6 |
| RAB10 |  | 0.036 | 10.939 | 1.116 | 2.65E-01 | 5.62E-01 | -5.601 |
| PMCHL1 |  | 0.06 | 5.349 | 1.115 | 2.65E-01 | 5.62E-01 | -5.601 |
| EMCN |  | 0.076 | 5.566 | 1.114 | 2.65E-01 | 5.63E-01 | -5.602 |
| LOH12CR1 |  | -0.063 | 7.582 | -1.115 | 2.66E-01 | 5.63E-01 | -5.602 |
| SOX21 |  | -0.062 | 5.482 | -1.115 | 2.66E-01 | 5.63E-01 | -5.602 |
| RNF135 |  | -0.037 | 8.775 | -1.115 | 2.66E-01 | 5.63E-01 | -5.602 |
| ANAPC2 |  | 0.051 | 6.928 | 1.114 | 2.66E-01 | 5.63E-01 | -5.603 |
| RPE65 |  | 0.072 | 4.792 | 1.114 | 2.66E-01 | 5.63E-01 | -5.603 |
| FDFT1 |  | 0.05 | 10.94 | 1.113 | 2.66E-01 | 5.63E-01 | -5.603 |
| ABCA4 |  | -0.071 | 5.463 | -1.114 | 2.66E-01 | 5.63E-01 | -5.603 |
| GLIPR1 |  | -0.09 | 8.681 | -1.114 | 2.66E-01 | 5.63E-01 | -5.604 |
| MPDZ |  | 0.044 | 8.17 | 1.113 | 2.66E-01 | 5.63E-01 | -5.604 |
| KCNK2 |  | 0.058 | 4.696 | 1.112 | 2.66E-01 | 5.63E-01 | -5.604 |
| USHBP1 |  | -0.055 | 5.887 | -1.113 | 2.66E-01 | 5.63E-01 | -5.604 |
| CASP9 |  | -0.045 | 8.513 | -1.113 | 2.67E-01 | 5.63E-01 | -5.605 |
| DOT1L |  | 0.042 | 6.624 | 1.112 | 2.67E-01 | 5.63E-01 | -5.605 |
| GUCA1C |  | -0.07 | 4.898 | -1.113 | 2.67E-01 | 5.63E-01 | -5.605 |
| GYG2 |  | -0.082 | 5.9 | -1.112 | 2.67E-01 | 5.63E-01 | -5.606 |
| PSMD8 |  | 0.047 | 10.661 | 1.11 | 2.67E-01 | 5.64E-01 | -5.606 |
| ITGB5 |  | 0.061 | 9.416 | 1.11 | 2.67E-01 | 5.64E-01 | -5.607 |
| ENTPD4 |  | 0.055 | 8.059 | 1.109 | 2.68E-01 | 5.65E-01 | -5.608 |
| ZNF557 |  | 0.051 | 7.558 | 1.109 | 2.68E-01 | 5.65E-01 | -5.608 |
| SCARF2 |  | -0.064 | 8.332 | -1.109 | 2.68E-01 | 5.65E-01 | -5.608 |
| FCAR |  | -0.054 | 5.95 | -1.109 | 2.68E-01 | 5.65E-01 | -5.609 |
| SECTM1 |  | -0.058 | 7.795 | -1.108 | 2.69E-01 | 5.65E-01 | -5.61 |
| HCCS |  | 0.04 | 8.213 | 1.105 | 2.70E-01 | 5.67E-01 | -5.612 |
| RFC3 |  | -0.054 | 7.229 | -1.105 | 2.70E-01 | 5.67E-01 | -5.613 |
| NDUFB1 |  | 0.051 | 9.948 | 1.104 | 2.70E-01 | 5.67E-01 | -5.613 |
| SELL |  | 0.078 | 6.902 | 1.104 | 2.70E-01 | 5.67E-01 | -5.613 |
| SERPINE1 |  | 0.111 | 10.688 | 1.104 | 2.70E-01 | 5.67E-01 | -5.613 |
| POLR2A |  | -0.062 | 9.931 | -1.104 | 2.70E-01 | 5.68E-01 | -5.614 |
| SHANK1 |  | -0.061 | 6.553 | -1.104 | 2.70E-01 | 5.68E-01 | -5.614 |
| OMD |  | 0.108 | 5.288 | 1.102 | 2.71E-01 | 5.68E-01 | -5.615 |
| RAB40C |  | -0.084 | 7.869 | -1.103 | 2.71E-01 | 5.68E-01 | -5.615 |
| ZRANB3 |  | 0.048 | 5.64 | 1.102 | 2.71E-01 | 5.68E-01 | -5.615 |
| THBS1 |  | -0.095 | 8.847 | -1.103 | 2.71E-01 | 5.68E-01 | -5.615 |
| CD86 |  | -0.116 | 7.161 | -1.103 | 2.71E-01 | 5.68E-01 | -5.615 |
| NRXN1 |  | -0.056 | 4.833 | -1.103 | 2.71E-01 | 5.68E-01 | -5.616 |
| MAPK8IP3 |  | 0.073 | 7.596 | 1.101 | 2.71E-01 | 5.68E-01 | -5.616 |
| TST |  | 0.052 | 10.879 | 1.101 | 2.71E-01 | 5.68E-01 | -5.616 |
| PBX3 |  | 0.045 | 8.265 | 1.101 | 2.71E-01 | 5.68E-01 | -5.616 |
| EEF2 |  | -0.049 | 13.129 | -1.101 | 2.72E-01 | 5.69E-01 | -5.618 |
| CD300A |  | -0.063 | 7.372 | -1.101 | 2.72E-01 | 5.69E-01 | -5.618 |
| CAMTA1 |  | 0.055 | 6.619 | 1.099 | 2.72E-01 | 5.70E-01 | -5.619 |
| KIF17 |  | 0.071 | 6.74 | 1.099 | 2.72E-01 | 5.70E-01 | -5.619 |
| CHGB |  | 0.067 | 5.118 | 1.099 | 2.72E-01 | 5.70E-01 | -5.619 |
| KLHL20 |  | 0.051 | 8.214 | 1.098 | 2.72E-01 | 5.70E-01 | -5.619 |
| SLC14A1 |  | -0.082 | 6.209 | -1.099 | 2.72E-01 | 5.70E-01 | -5.619 |
| SSTR5 |  | -0.062 | 5.837 | -1.099 | 2.72E-01 | 5.70E-01 | -5.619 |
| HS3ST2 |  | -0.087 | 5.281 | -1.099 | 2.73E-01 | 5.70E-01 | -5.62 |
| CYP24A1 |  | -0.07 | 5.568 | -1.099 | 2.73E-01 | 5.70E-01 | -5.62 |
| SLC22A8 |  | -0.074 | 5.507 | -1.098 | 2.73E-01 | 5.70E-01 | -5.62 |
| GABRA4 |  | -0.069 | 5.261 | -1.098 | 2.73E-01 | 5.70E-01 | -5.621 |
| ASNS |  | 0.069 | 9.114 | 1.097 | 2.73E-01 | 5.70E-01 | -5.621 |
| RILP |  | -0.062 | 7.811 | -1.098 | 2.73E-01 | 5.70E-01 | -5.621 |
| ILF2 |  | -0.055 | 10.401 | -1.097 | 2.73E-01 | 5.70E-01 | -5.621 |
| RUFY2 |  | -0.062 | 6.495 | -1.097 | 2.73E-01 | 5.70E-01 | -5.621 |
| AGPS |  | -0.042 | 8.232 | -1.097 | 2.73E-01 | 5.70E-01 | -5.621 |
| TSTA3 |  | 0.058 | 9.377 | 1.096 | 2.74E-01 | 5.70E-01 | -5.622 |
| CAMK2B |  | 0.045 | 5.133 | 1.095 | 2.74E-01 | 5.70E-01 | -5.622 |
| TMEM39B |  | -0.049 | 8.888 | -1.096 | 2.74E-01 | 5.70E-01 | -5.622 |
| ZNF586 |  | 0.043 | 7.6 | 1.095 | 2.74E-01 | 5.70E-01 | -5.622 |
| PCGF3 |  | -0.04 | 7.691 | -1.096 | 2.74E-01 | 5.70E-01 | -5.622 |
| IL18BP |  | -0.053 | 7.723 | -1.096 | 2.74E-01 | 5.70E-01 | -5.623 |
| EGFL6 |  | -0.065 | 9.268 | -1.096 | 2.74E-01 | 5.70E-01 | -5.623 |
| TRAM1 |  | 0.055 | 11.489 | 1.095 | 2.74E-01 | 5.70E-01 | -5.623 |
| GPD1L |  | 0.052 | 9.165 | 1.094 | 2.74E-01 | 5.70E-01 | -5.623 |
| ATXN7L2 |  | 0.058 | 8.46 | 1.093 | 2.74E-01 | 5.71E-01 | -5.624 |
| OSBPL7 |  | 0.056 | 6.991 | 1.093 | 2.75E-01 | 5.71E-01 | -5.625 |
| CFTR |  | -0.074 | 5.365 | -1.093 | 2.75E-01 | 5.71E-01 | -5.625 |
| SNN |  | -0.053 | 7.772 | -1.093 | 2.75E-01 | 5.71E-01 | -5.626 |
| NDUFB5 |  | -0.037 | 10.43 | -1.092 | 2.75E-01 | 5.72E-01 | -5.626 |
| AREG |  | 0.081 | 8.182 | 1.091 | 2.75E-01 | 5.72E-01 | -5.627 |
| BRIP1 |  | 0.053 | 5.226 | 1.091 | 2.76E-01 | 5.72E-01 | -5.627 |
| RNGTT |  | 0.062 | 8.213 | 1.091 | 2.76E-01 | 5.72E-01 | -5.627 |
| TBC1D13 |  | 0.044 | 9.7 | 1.09 | 2.76E-01 | 5.72E-01 | -5.627 |
| SALL2 |  | -0.073 | 7.136 | -1.091 | 2.76E-01 | 5.72E-01 | -5.628 |
| TBR1 |  | -0.046 | 4.847 | -1.091 | 2.76E-01 | 5.72E-01 | -5.628 |
| OR2H1 |  | -0.053 | 5.835 | -1.091 | 2.76E-01 | 5.72E-01 | -5.628 |
| NDUFV2 |  | 0.037 | 11.319 | 1.089 | 2.76E-01 | 5.72E-01 | -5.629 |
| CCR8 |  | 0.059 | 4.754 | 1.089 | 2.76E-01 | 5.72E-01 | -5.629 |
| EME1 |  | 0.05 | 6.553 | 1.089 | 2.76E-01 | 5.72E-01 | -5.629 |
| AKAP6 |  | -0.056 | 5.331 | -1.09 | 2.76E-01 | 5.72E-01 | -5.629 |
| PTPN21 |  | 0.047 | 7.653 | 1.088 | 2.77E-01 | 5.72E-01 | -5.63 |
| MTAP |  | 0.037 | 8.008 | 1.088 | 2.77E-01 | 5.72E-01 | -5.63 |
| ADAM17 |  | 0.037 | 8.908 | 1.088 | 2.77E-01 | 5.72E-01 | -5.63 |
| FCN1 |  | -0.083 | 7.991 | -1.089 | 2.77E-01 | 5.72E-01 | -5.63 |
| WWP1 |  | 0.049 | 9.384 | 1.087 | 2.77E-01 | 5.73E-01 | -5.631 |
| PDE7A |  | 0.064 | 6.583 | 1.087 | 2.77E-01 | 5.73E-01 | -5.631 |
| CIZ1 |  | -0.044 | 8.403 | -1.087 | 2.78E-01 | 5.73E-01 | -5.632 |
| GLT8D2 |  | 0.057 | 6.609 | 1.084 | 2.79E-01 | 5.75E-01 | -5.634 |
| RAB33B |  | 0.069 | 7.326 | 1.084 | 2.79E-01 | 5.75E-01 | -5.635 |
| DMRT1 |  | -0.091 | 5.127 | -1.084 | 2.79E-01 | 5.75E-01 | -5.635 |
| UBE4A |  | 0.044 | 10.049 | 1.083 | 2.79E-01 | 5.76E-01 | -5.635 |
| ADPRHL2 |  | 0.037 | 9.134 | 1.083 | 2.79E-01 | 5.76E-01 | -5.635 |
| STX7 |  | -0.055 | 8.671 | -1.084 | 2.79E-01 | 5.76E-01 | -5.635 |
| FOXE1 |  | -0.061 | 5.431 | -1.083 | 2.80E-01 | 5.76E-01 | -5.636 |
| GYPE |  | -0.074 | 6.345 | -1.083 | 2.80E-01 | 5.76E-01 | -5.636 |
| SLAMF7 |  | -0.1 | 6.675 | -1.082 | 2.80E-01 | 5.76E-01 | -5.637 |
| ASXL2 |  | -0.042 | 9.015 | -1.082 | 2.80E-01 | 5.76E-01 | -5.637 |
| CSN3 |  | 0.056 | 4.841 | 1.081 | 2.80E-01 | 5.76E-01 | -5.638 |
| ASB17 |  | 0.082 | 4.714 | 1.081 | 2.80E-01 | 5.76E-01 | -5.638 |
| RCOR3 |  | -0.042 | 8.735 | -1.082 | 2.80E-01 | 5.76E-01 | -5.638 |
| WFIKKN2 |  | 0.052 | 5.027 | 1.08 | 2.80E-01 | 5.77E-01 | -5.638 |
| HCRTR2 |  | 0.055 | 5.02 | 1.08 | 2.80E-01 | 5.77E-01 | -5.638 |
| COL11A1 |  | 0.068 | 5.785 | 1.079 | 2.81E-01 | 5.77E-01 | -5.639 |
| SLC39A14 |  | -0.058 | 7.366 | -1.08 | 2.81E-01 | 5.77E-01 | -5.639 |
| GNL1 |  | 0.059 | 8.057 | 1.079 | 2.81E-01 | 5.77E-01 | -5.639 |
| CCNF |  | 0.05 | 7.004 | 1.078 | 2.81E-01 | 5.78E-01 | -5.64 |
| CD7 |  | -0.069 | 6.663 | -1.079 | 2.81E-01 | 5.78E-01 | -5.64 |
| POLDIP3 |  | -0.038 | 9.372 | -1.078 | 2.82E-01 | 5.78E-01 | -5.641 |
| KATNAL2 |  | 0.059 | 5.061 | 1.077 | 2.82E-01 | 5.78E-01 | -5.641 |
| EPX |  | 0.059 | 4.839 | 1.076 | 2.82E-01 | 5.79E-01 | -5.642 |
| AIPL1 |  | -0.045 | 4.814 | -1.077 | 2.82E-01 | 5.79E-01 | -5.642 |
| TSPYL6 |  | 0.066 | 4.991 | 1.076 | 2.82E-01 | 5.79E-01 | -5.642 |
| UCP1 |  | 0.056 | 4.79 | 1.076 | 2.82E-01 | 5.79E-01 | -5.643 |
| PIK3R4 |  | -0.048 | 8.266 | -1.077 | 2.82E-01 | 5.79E-01 | -5.643 |
| CXorf36 |  | -0.053 | 6.108 | -1.076 | 2.83E-01 | 5.79E-01 | -5.643 |
| NOL6 |  | -0.054 | 7.59 | -1.076 | 2.83E-01 | 5.79E-01 | -5.643 |
| PGAM2 |  | -0.061 | 5.845 | -1.076 | 2.83E-01 | 5.79E-01 | -5.644 |
| AP3M2 |  | -0.049 | 8.298 | -1.075 | 2.83E-01 | 5.79E-01 | -5.644 |
| ST5 |  | -0.063 | 8.043 | -1.075 | 2.83E-01 | 5.79E-01 | -5.644 |
| MEGF10 |  | -0.058 | 5.103 | -1.075 | 2.83E-01 | 5.79E-01 | -5.644 |
| PAFAH1B2 |  | -0.045 | 8.198 | -1.075 | 2.83E-01 | 5.79E-01 | -5.644 |
| TK1 |  | 0.08 | 10.183 | 1.074 | 2.83E-01 | 5.79E-01 | -5.644 |
| FBXL17 |  | -0.053 | 7.316 | -1.074 | 2.83E-01 | 5.79E-01 | -5.645 |
| UTY |  | -0.113 | 5.531 | -1.074 | 2.83E-01 | 5.79E-01 | -5.645 |
| CPNE2 |  | -0.072 | 8.06 | -1.074 | 2.83E-01 | 5.79E-01 | -5.645 |
| SOSTDC1 |  | 0.075 | 4.975 | 1.072 | 2.84E-01 | 5.80E-01 | -5.646 |
| ADPRHL1 |  | 0.057 | 5.947 | 1.072 | 2.84E-01 | 5.80E-01 | -5.647 |
| BANF1 |  | 0.044 | 8.842 | 1.072 | 2.84E-01 | 5.80E-01 | -5.647 |
| CD1A |  | 0.054 | 5.312 | 1.071 | 2.84E-01 | 5.80E-01 | -5.647 |
| RGS17 |  | 0.076 | 5.076 | 1.071 | 2.84E-01 | 5.80E-01 | -5.648 |
| H2AFJ |  | 0.052 | 9.176 | 1.071 | 2.85E-01 | 5.80E-01 | -5.648 |
| ZCCHC5 |  | -0.063 | 4.871 | -1.071 | 2.85E-01 | 5.80E-01 | -5.648 |
| PDXK |  | 0.044 | 9.285 | 1.07 | 2.85E-01 | 5.80E-01 | -5.648 |
| LARGE |  | -0.073 | 6.876 | -1.071 | 2.85E-01 | 5.80E-01 | -5.649 |
| PKLR |  | -0.062 | 6.171 | -1.07 | 2.85E-01 | 5.81E-01 | -5.65 |
| ITGBL1 |  | 0.069 | 6.91 | 1.069 | 2.85E-01 | 5.81E-01 | -5.65 |
| CNKSR1 |  | -0.055 | 6.949 | -1.069 | 2.86E-01 | 5.81E-01 | -5.65 |
| PMPCB |  | -0.032 | 9.652 | -1.069 | 2.86E-01 | 5.81E-01 | -5.65 |
| PCBP3 |  | -0.075 | 5.245 | -1.069 | 2.86E-01 | 5.81E-01 | -5.65 |
| DOCK6 |  | 0.066 | 7.75 | 1.067 | 2.86E-01 | 5.82E-01 | -5.651 |
| NSUN4 |  | 0.033 | 8.183 | 1.067 | 2.86E-01 | 5.82E-01 | -5.652 |
| APCDD1 |  | -0.093 | 8.364 | -1.067 | 2.86E-01 | 5.82E-01 | -5.652 |
| RPL12 |  | -0.034 | 13.9 | -1.067 | 2.86E-01 | 5.82E-01 | -5.652 |
| CSNK1A1 |  | -0.035 | 9.511 | -1.067 | 2.87E-01 | 5.82E-01 | -5.652 |
| FKBP8 |  | 0.065 | 9.084 | 1.066 | 2.87E-01 | 5.82E-01 | -5.653 |
| ZNF256 |  | -0.044 | 7.995 | -1.066 | 2.87E-01 | 5.83E-01 | -5.654 |
| APEH |  | 0.041 | 9.959 | 1.065 | 2.87E-01 | 5.83E-01 | -5.654 |
| C3orf18 |  | 0.059 | 7.637 | 1.064 | 2.87E-01 | 5.83E-01 | -5.654 |
| PACS1 |  | -0.066 | 7.981 | -1.065 | 2.87E-01 | 5.83E-01 | -5.654 |
| CDR1 |  | 0.052 | 4.605 | 1.064 | 2.88E-01 | 5.83E-01 | -5.655 |
| HGFAC |  | -0.058 | 6.878 | -1.065 | 2.88E-01 | 5.83E-01 | -5.655 |
| RPUSD2 |  | -0.037 | 9.295 | -1.064 | 2.88E-01 | 5.83E-01 | -5.655 |
| VPS4A |  | 0.043 | 9.574 | 1.063 | 2.88E-01 | 5.83E-01 | -5.656 |
| RPP38 |  | -0.032 | 8.303 | -1.064 | 2.88E-01 | 5.83E-01 | -5.656 |
| OTC |  | 0.062 | 4.678 | 1.062 | 2.88E-01 | 5.84E-01 | -5.656 |
| GPD2 |  | 0.043 | 7.095 | 1.061 | 2.89E-01 | 5.84E-01 | -5.657 |
| PSKH1 |  | 0.05 | 8.006 | 1.061 | 2.89E-01 | 5.84E-01 | -5.657 |
| PHTF2 |  | 0.065 | 7.331 | 1.061 | 2.89E-01 | 5.85E-01 | -5.658 |
| SPCS1 |  | 0.035 | 11.67 | 1.061 | 2.89E-01 | 5.85E-01 | -5.658 |
| WFDC8 |  | -0.055 | 5.003 | -1.061 | 2.89E-01 | 5.85E-01 | -5.659 |
| ZNF16 |  | -0.045 | 6.72 | -1.061 | 2.90E-01 | 5.85E-01 | -5.659 |
| HEBP1 |  | -0.048 | 10.772 | -1.06 | 2.90E-01 | 5.85E-01 | -5.66 |
| CHRNB2 |  | -0.053 | 5.559 | -1.06 | 2.90E-01 | 5.85E-01 | -5.66 |
| LMX1B |  | -0.049 | 5.868 | -1.06 | 2.90E-01 | 5.85E-01 | -5.66 |
| RUVBL1 |  | -0.046 | 7.974 | -1.059 | 2.90E-01 | 5.86E-01 | -5.661 |
| CPAMD8 |  | -0.058 | 7.104 | -1.058 | 2.91E-01 | 5.86E-01 | -5.662 |
| GZMK |  | 0.101 | 6.315 | 1.057 | 2.91E-01 | 5.86E-01 | -5.662 |
| HBB |  | -0.063 | 12.314 | -1.058 | 2.91E-01 | 5.86E-01 | -5.662 |
| TBC1D8 |  | -0.052 | 7.722 | -1.058 | 2.91E-01 | 5.86E-01 | -5.662 |
| NRAP |  | 0.046 | 4.884 | 1.056 | 2.91E-01 | 5.87E-01 | -5.663 |
| UPB1 |  | -0.057 | 5.791 | -1.057 | 2.91E-01 | 5.87E-01 | -5.663 |
| LRCH2 |  | -0.062 | 6.634 | -1.057 | 2.91E-01 | 5.87E-01 | -5.663 |
| ADSL |  | -0.041 | 10.082 | -1.057 | 2.91E-01 | 5.87E-01 | -5.663 |
| CHUK |  | 0.054 | 8.653 | 1.055 | 2.92E-01 | 5.87E-01 | -5.664 |
| MAFF |  | -0.09 | 9.38 | -1.056 | 2.92E-01 | 5.87E-01 | -5.664 |
| OSBPL5 |  | 0.056 | 8.159 | 1.054 | 2.92E-01 | 5.87E-01 | -5.665 |
| ELMO1 |  | -0.072 | 7.337 | -1.055 | 2.92E-01 | 5.87E-01 | -5.665 |
| LUZP4 |  | 0.064 | 4.72 | 1.054 | 2.92E-01 | 5.87E-01 | -5.665 |
| PPIE |  | 0.043 | 8.185 | 1.054 | 2.92E-01 | 5.87E-01 | -5.665 |
| PI15 |  | -0.082 | 5.418 | -1.055 | 2.92E-01 | 5.87E-01 | -5.665 |
| SRD5A2 |  | 0.062 | 4.861 | 1.053 | 2.92E-01 | 5.87E-01 | -5.665 |
| COMMD9 |  | -0.044 | 8.723 | -1.054 | 2.92E-01 | 5.87E-01 | -5.666 |
| CENPH |  | -0.052 | 7.277 | -1.054 | 2.93E-01 | 5.87E-01 | -5.666 |
| GBA2 |  | -0.041 | 7.816 | -1.054 | 2.93E-01 | 5.87E-01 | -5.666 |
| SS18L2 |  | 0.044 | 10.255 | 1.053 | 2.93E-01 | 5.88E-01 | -5.666 |
| ZNF165 |  | -0.06 | 7.745 | -1.053 | 2.93E-01 | 5.88E-01 | -5.667 |
| TCTE3 |  | -0.062 | 5.37 | -1.053 | 2.93E-01 | 5.88E-01 | -5.667 |
| PAPSS1 |  | 0.062 | 8.836 | 1.052 | 2.93E-01 | 5.88E-01 | -5.667 |
| KLHL17 |  | 0.055 | 7.47 | 1.051 | 2.93E-01 | 5.88E-01 | -5.668 |
| ZDHHC17 |  | -0.041 | 8.218 | -1.052 | 2.93E-01 | 5.88E-01 | -5.668 |
| EGR2 |  | 0.103 | 6.273 | 1.051 | 2.94E-01 | 5.88E-01 | -5.668 |
| MPV17 |  | 0.039 | 9.517 | 1.049 | 2.94E-01 | 5.89E-01 | -5.67 |
| LYNX1 |  | 0.06 | 7.178 | 1.049 | 2.94E-01 | 5.89E-01 | -5.67 |
| SCAMP3 |  | -0.041 | 10.177 | -1.049 | 2.95E-01 | 5.90E-01 | -5.671 |
| CHRNB3 |  | -0.06 | 5.481 | -1.049 | 2.95E-01 | 5.90E-01 | -5.671 |
| RRM2 |  | -0.07 | 7.881 | -1.049 | 2.95E-01 | 5.90E-01 | -5.671 |
| NAP1L2 |  | 0.063 | 5.484 | 1.048 | 2.95E-01 | 5.90E-01 | -5.671 |
| GIP |  | 0.063 | 5.079 | 1.048 | 2.95E-01 | 5.90E-01 | -5.671 |
| SCNN1A |  | -0.084 | 5.733 | -1.048 | 2.95E-01 | 5.90E-01 | -5.672 |
| TEAD4 |  | 0.036 | 7.596 | 1.047 | 2.96E-01 | 5.90E-01 | -5.672 |
| RIBC2 |  | 0.079 | 5.81 | 1.046 | 2.96E-01 | 5.90E-01 | -5.673 |
| SYNGR1 |  | 0.05 | 7.58 | 1.046 | 2.96E-01 | 5.90E-01 | -5.673 |
| NR1H4 |  | -0.096 | 5.097 | -1.047 | 2.96E-01 | 5.90E-01 | -5.673 |
| AGTRAP |  | -0.056 | 8.219 | -1.047 | 2.96E-01 | 5.90E-01 | -5.673 |
| PIK3AP1 |  | 0.091 | 7.213 | 1.046 | 2.96E-01 | 5.90E-01 | -5.673 |
| SART3 |  | -0.039 | 7.643 | -1.046 | 2.96E-01 | 5.91E-01 | -5.674 |
| PGM2L1 |  | 0.059 | 6.881 | 1.045 | 2.96E-01 | 5.91E-01 | -5.674 |
| SERTAD2 |  | 0.044 | 9.815 | 1.044 | 2.97E-01 | 5.91E-01 | -5.674 |
| SF3B2 |  | -0.036 | 11.51 | -1.045 | 2.97E-01 | 5.91E-01 | -5.675 |
| GPRASP1 |  | -0.056 | 7.093 | -1.045 | 2.97E-01 | 5.91E-01 | -5.675 |
| SDC2 |  | -0.072 | 7.254 | -1.045 | 2.97E-01 | 5.91E-01 | -5.675 |
| SEC24D |  | -0.063 | 9.081 | -1.044 | 2.97E-01 | 5.91E-01 | -5.676 |
| PTGS2 |  | -0.11 | 6.541 | -1.044 | 2.97E-01 | 5.92E-01 | -5.676 |
| DAB2IP |  | 0.052 | 7.153 | 1.042 | 2.98E-01 | 5.92E-01 | -5.677 |
| NGLY1 |  | 0.038 | 9.517 | 1.042 | 2.98E-01 | 5.92E-01 | -5.677 |
| ACVR1B |  | -0.055 | 6.495 | -1.043 | 2.98E-01 | 5.92E-01 | -5.677 |
| RPS15 |  | 0.04 | 12.007 | 1.042 | 2.98E-01 | 5.92E-01 | -5.677 |
| SLC43A1 |  | -0.045 | 7.538 | -1.042 | 2.98E-01 | 5.92E-01 | -5.678 |
| C14orf142 |  | 0.059 | 7.591 | 1.04 | 2.99E-01 | 5.93E-01 | -5.679 |
| KLF7 |  | 0.061 | 6.818 | 1.04 | 2.99E-01 | 5.93E-01 | -5.679 |
| SGOL1 |  | -0.054 | 6.24 | -1.041 | 2.99E-01 | 5.93E-01 | -5.679 |
| RP1 |  | -0.055 | 4.739 | -1.04 | 2.99E-01 | 5.93E-01 | -5.68 |
| PNPLA5 |  | -0.063 | 6.159 | -1.04 | 2.99E-01 | 5.94E-01 | -5.68 |
| RNF26 |  | 0.055 | 9.142 | 1.038 | 3.00E-01 | 5.95E-01 | -5.681 |
| ARHGEF18 |  | 0.038 | 9.849 | 1.037 | 3.00E-01 | 5.95E-01 | -5.682 |
| ZNF623 |  | -0.042 | 6.516 | -1.038 | 3.00E-01 | 5.95E-01 | -5.682 |
| KRT24 |  | 0.123 | 5.952 | 1.036 | 3.00E-01 | 5.95E-01 | -5.683 |
| NMU |  | 0.087 | 6.834 | 1.036 | 3.00E-01 | 5.95E-01 | -5.683 |
| REST |  | 0.051 | 6.93 | 1.036 | 3.00E-01 | 5.95E-01 | -5.683 |
| PRM1 |  | -0.067 | 5.601 | -1.037 | 3.00E-01 | 5.95E-01 | -5.683 |
| CASP4 |  | -0.036 | 11.029 | -1.037 | 3.01E-01 | 5.95E-01 | -5.683 |
| ADAMTS2 |  | 0.071 | 6.277 | 1.036 | 3.01E-01 | 5.95E-01 | -5.683 |
| BCL10 |  | -0.054 | 6.808 | -1.037 | 3.01E-01 | 5.95E-01 | -5.683 |
| NRAS |  | -0.042 | 8.359 | -1.036 | 3.01E-01 | 5.95E-01 | -5.683 |
| NEBL |  | 0.095 | 6.494 | 1.035 | 3.01E-01 | 5.95E-01 | -5.684 |
| KIAA0907 |  | -0.042 | 9.147 | -1.036 | 3.01E-01 | 5.95E-01 | -5.684 |
| SALL1 |  | -0.069 | 5.532 | -1.035 | 3.01E-01 | 5.96E-01 | -5.685 |
| TIMP2 |  | 0.053 | 12.252 | 1.034 | 3.02E-01 | 5.96E-01 | -5.685 |
| TRIM26 |  | -0.042 | 8.745 | -1.035 | 3.02E-01 | 5.96E-01 | -5.685 |
| SCYL2 |  | -0.059 | 8.99 | -1.034 | 3.02E-01 | 5.96E-01 | -5.685 |
| EEF1E1 |  | 0.057 | 8.609 | 1.033 | 3.02E-01 | 5.96E-01 | -5.686 |
| BCL2L2 |  | 0.038 | 9.577 | 1.033 | 3.02E-01 | 5.96E-01 | -5.686 |
| TIGD5 |  | 0.04 | 8.365 | 1.032 | 3.02E-01 | 5.97E-01 | -5.687 |
| B3GNT5 |  | -0.069 | 8.47 | -1.033 | 3.03E-01 | 5.97E-01 | -5.687 |
| TAF2 |  | -0.057 | 8.447 | -1.032 | 3.03E-01 | 5.97E-01 | -5.688 |
| CD5 |  | -0.084 | 6.1 | -1.032 | 3.03E-01 | 5.97E-01 | -5.688 |
| DEPDC5 |  | -0.038 | 7.207 | -1.031 | 3.03E-01 | 5.97E-01 | -5.688 |
| BHMT2 |  | -0.076 | 5.214 | -1.031 | 3.03E-01 | 5.97E-01 | -5.689 |
| SULT1E1 |  | -0.066 | 5.217 | -1.031 | 3.03E-01 | 5.97E-01 | -5.689 |
| NMNAT2 |  | -0.071 | 6 | -1.031 | 3.03E-01 | 5.97E-01 | -5.689 |
| PGM2 |  | -0.053 | 9.733 | -1.03 | 3.04E-01 | 5.97E-01 | -5.689 |
| ZBTB10 |  | -0.058 | 6.169 | -1.03 | 3.04E-01 | 5.97E-01 | -5.69 |
| TFCP2 |  | -0.04 | 8.299 | -1.03 | 3.04E-01 | 5.97E-01 | -5.69 |
| GPR52 |  | 0.061 | 5.057 | 1.029 | 3.04E-01 | 5.97E-01 | -5.69 |
| GHITM |  | -0.037 | 10.697 | -1.03 | 3.04E-01 | 5.97E-01 | -5.69 |
| NTAN1 |  | -0.038 | 8.796 | -1.03 | 3.04E-01 | 5.97E-01 | -5.69 |
| TNFSF9 |  | 0.053 | 6.245 | 1.028 | 3.04E-01 | 5.97E-01 | -5.69 |
| FOXN1 |  | -0.063 | 6.162 | -1.029 | 3.04E-01 | 5.97E-01 | -5.691 |
| DMAP1 |  | 0.046 | 9.008 | 1.028 | 3.04E-01 | 5.97E-01 | -5.691 |
| NUDT16L1 |  | 0.045 | 8.918 | 1.027 | 3.05E-01 | 5.98E-01 | -5.692 |
| RBP7 |  | -0.07 | 8.214 | -1.026 | 3.05E-01 | 6.00E-01 | -5.693 |
| HAVCR2 |  | -0.054 | 7.529 | -1.026 | 3.06E-01 | 6.00E-01 | -5.694 |
| HECW1 |  | 0.046 | 4.749 | 1.024 | 3.06E-01 | 6.00E-01 | -5.694 |
| ZBTB11 |  | 0.06 | 8.593 | 1.024 | 3.06E-01 | 6.00E-01 | -5.695 |
| TMCC1 |  | 0.05 | 7.92 | 1.024 | 3.06E-01 | 6.00E-01 | -5.695 |
| ZNF32 |  | -0.049 | 7.535 | -1.025 | 3.06E-01 | 6.00E-01 | -5.695 |
| CD22 |  | 0.068 | 5.8 | 1.023 | 3.06E-01 | 6.00E-01 | -5.695 |
| ZNF132 |  | -0.055 | 6.397 | -1.024 | 3.06E-01 | 6.00E-01 | -5.695 |
| ERO1LB |  | -0.056 | 6.397 | -1.024 | 3.07E-01 | 6.01E-01 | -5.696 |
| VWF |  | 0.051 | 9.223 | 1.022 | 3.07E-01 | 6.01E-01 | -5.697 |
| POU4F1 |  | -0.06 | 4.866 | -1.023 | 3.07E-01 | 6.01E-01 | -5.697 |
| PDCD4 |  | 0.063 | 9.499 | 1.021 | 3.07E-01 | 6.02E-01 | -5.697 |
| CYP4Z1 |  | -0.057 | 4.72 | -1.022 | 3.08E-01 | 6.02E-01 | -5.698 |
| ADRA1A |  | -0.05 | 5.277 | -1.022 | 3.08E-01 | 6.02E-01 | -5.698 |
| SLC7A1 |  | -0.073 | 8.162 | -1.021 | 3.08E-01 | 6.02E-01 | -5.699 |
| SLC34A1 |  | -0.063 | 5.846 | -1.021 | 3.08E-01 | 6.02E-01 | -5.699 |
| ZNF100 |  | 0.06 | 6.559 | 1.019 | 3.08E-01 | 6.03E-01 | -5.7 |
| CREB3L2 |  | -0.042 | 11.127 | -1.02 | 3.09E-01 | 6.03E-01 | -5.7 |
| RAPGEFL1 |  | -0.062 | 6.077 | -1.019 | 3.09E-01 | 6.03E-01 | -5.7 |
| AOC2 |  | -0.047 | 6.036 | -1.019 | 3.09E-01 | 6.03E-01 | -5.701 |
| PRF1 |  | -0.067 | 6.725 | -1.019 | 3.09E-01 | 6.03E-01 | -5.701 |
| UBAP1 |  | -0.036 | 9.917 | -1.017 | 3.10E-01 | 6.05E-01 | -5.703 |
| ABCG4 |  | 0.054 | 5.732 | 1.016 | 3.10E-01 | 6.05E-01 | -5.703 |
| NSUN2 |  | 0.044 | 10.101 | 1.015 | 3.10E-01 | 6.05E-01 | -5.703 |
| RASL10B |  | -0.075 | 6.267 | -1.016 | 3.10E-01 | 6.05E-01 | -5.703 |
| MYF6 |  | -0.051 | 4.856 | -1.016 | 3.10E-01 | 6.05E-01 | -5.704 |
| ZFYVE26 |  | -0.039 | 8.365 | -1.016 | 3.10E-01 | 6.05E-01 | -5.704 |
| GTF2E2 |  | -0.036 | 9.71 | -1.015 | 3.11E-01 | 6.05E-01 | -5.704 |
| S100A9 |  | 0.068 | 10.382 | 1.014 | 3.11E-01 | 6.05E-01 | -5.705 |
| IDH3G |  | -0.033 | 9.071 | -1.015 | 3.11E-01 | 6.05E-01 | -5.705 |
| SLCO4A1 |  | 0.121 | 8.164 | 1.014 | 3.11E-01 | 6.05E-01 | -5.705 |
| ABCB11 |  | 0.048 | 4.792 | 1.013 | 3.11E-01 | 6.06E-01 | -5.705 |
| INSL5 |  | -0.06 | 5.004 | -1.014 | 3.11E-01 | 6.06E-01 | -5.706 |
| PGLYRP3 |  | 0.052 | 5.136 | 1.013 | 3.11E-01 | 6.06E-01 | -5.706 |
| SPRR4 |  | -0.061 | 5.366 | -1.013 | 3.12E-01 | 6.06E-01 | -5.707 |
| PTBP2 |  | 0.052 | 8.055 | 1.011 | 3.12E-01 | 6.07E-01 | -5.707 |
| KIAA0556 |  | 0.046 | 7.248 | 1.011 | 3.12E-01 | 6.07E-01 | -5.708 |
| BAP1 |  | -0.048 | 8.647 | -1.01 | 3.13E-01 | 6.08E-01 | -5.709 |
| RPS29 |  | 0.045 | 13.374 | 1.009 | 3.13E-01 | 6.08E-01 | -5.71 |
| POLR3D |  | -0.039 | 8.397 | -1.01 | 3.13E-01 | 6.08E-01 | -5.71 |
| C1RL |  | -0.052 | 7.045 | -1.01 | 3.13E-01 | 6.08E-01 | -5.71 |
| ETFA |  | -0.04 | 10.465 | -1.01 | 3.13E-01 | 6.08E-01 | -5.71 |
| MERTK |  | 0.049 | 7.791 | 1.008 | 3.14E-01 | 6.08E-01 | -5.71 |
| AGXT2 |  | -0.063 | 4.895 | -1.009 | 3.14E-01 | 6.09E-01 | -5.711 |
| CPNE7 |  | -0.071 | 6.611 | -1.009 | 3.14E-01 | 6.09E-01 | -5.711 |
| GRIN2A |  | -0.049 | 5.062 | -1.008 | 3.14E-01 | 6.09E-01 | -5.711 |
| KCNN4 |  | 0.062 | 9.734 | 1.007 | 3.14E-01 | 6.09E-01 | -5.711 |
| CTRL |  | -0.053 | 6.168 | -1.008 | 3.14E-01 | 6.09E-01 | -5.711 |
| TLR1 |  | -0.057 | 7.581 | -1.008 | 3.14E-01 | 6.09E-01 | -5.712 |
| MRPL50 |  | 0.044 | 9.433 | 1.007 | 3.14E-01 | 6.09E-01 | -5.712 |
| OPN3 |  | -0.058 | 8.842 | -1.007 | 3.14E-01 | 6.09E-01 | -5.712 |
| HIST1H2AJ |  | 0.048 | 8.628 | 1.006 | 3.15E-01 | 6.09E-01 | -5.712 |
| CAPNS2 |  | -0.055 | 6.438 | -1.007 | 3.15E-01 | 6.09E-01 | -5.713 |
| PCDHB9 |  | 0.054 | 5.616 | 1.006 | 3.15E-01 | 6.09E-01 | -5.713 |
| SORD |  | -0.053 | 6.796 | -1.006 | 3.15E-01 | 6.09E-01 | -5.713 |
| LAMB4 |  | -0.075 | 5.913 | -1.006 | 3.15E-01 | 6.09E-01 | -5.713 |
| CLTCL1 |  | -0.059 | 6.49 | -1.006 | 3.15E-01 | 6.09E-01 | -5.713 |
| NIPA1 |  | -0.061 | 6.869 | -1.006 | 3.15E-01 | 6.09E-01 | -5.714 |
| EPHA10 |  | -0.043 | 5.147 | -1.006 | 3.15E-01 | 6.09E-01 | -5.714 |
| NF2 |  | -0.038 | 7.57 | -1.005 | 3.16E-01 | 6.10E-01 | -5.714 |
| TP73 |  | -0.053 | 5.463 | -1.004 | 3.16E-01 | 6.10E-01 | -5.715 |
| HLA-DOA |  | -0.057 | 6.414 | -1.003 | 3.16E-01 | 6.11E-01 | -5.716 |
| IKBKB |  | 0.048 | 7.611 | 1.002 | 3.17E-01 | 6.11E-01 | -5.716 |
| BANK1 |  | 0.06 | 6.211 | 1.002 | 3.17E-01 | 6.11E-01 | -5.716 |
| TBCD |  | 0.045 | 9.031 | 1.002 | 3.17E-01 | 6.11E-01 | -5.717 |
| BMP1 |  | 0.07 | 7.845 | 1.002 | 3.17E-01 | 6.11E-01 | -5.717 |
| ATP1A4 |  | 0.075 | 6.693 | 1.001 | 3.17E-01 | 6.11E-01 | -5.717 |
| SOCS3 |  | -0.069 | 7.962 | -1.002 | 3.17E-01 | 6.11E-01 | -5.717 |
| ADCK2 |  | -0.038 | 8.707 | -1.002 | 3.17E-01 | 6.11E-01 | -5.718 |
| CRIPT |  | 0.046 | 8.606 | 1 | 3.17E-01 | 6.11E-01 | -5.718 |
| ANKRD17 |  | -0.035 | 8.29 | -1.001 | 3.18E-01 | 6.11E-01 | -5.718 |
| LRP10 |  | -0.051 | 10.398 | -1.001 | 3.18E-01 | 6.11E-01 | -5.718 |
| CRHBP |  | 0.09 | 6.61 | 0.999 | 3.18E-01 | 6.12E-01 | -5.719 |
| CTSG |  | -0.109 | 7.214 | -1 | 3.18E-01 | 6.12E-01 | -5.719 |
| CTDSPL |  | 0.061 | 9.087 | 0.999 | 3.18E-01 | 6.12E-01 | -5.719 |
| MTHFD2 |  | 0.06 | 8.884 | 0.998 | 3.18E-01 | 6.12E-01 | -5.72 |
| FGD2 |  | -0.041 | 6.427 | -0.999 | 3.18E-01 | 6.12E-01 | -5.72 |
| REG4 |  | -0.043 | 5.115 | -0.998 | 3.19E-01 | 6.12E-01 | -5.721 |
| GSK3A |  | 0.041 | 9.126 | 0.997 | 3.19E-01 | 6.12E-01 | -5.721 |
| GJA8 |  | 0.066 | 4.817 | 0.997 | 3.19E-01 | 6.12E-01 | -5.721 |
| IQSEC2 |  | 0.041 | 7.935 | 0.997 | 3.19E-01 | 6.12E-01 | -5.721 |
| TNFRSF14 |  | -0.051 | 9.027 | -0.998 | 3.19E-01 | 6.12E-01 | -5.721 |
| ARMCX6 |  | 0.046 | 9.22 | 0.997 | 3.19E-01 | 6.12E-01 | -5.721 |
| ZNF24 |  | -0.034 | 9.169 | -0.998 | 3.19E-01 | 6.12E-01 | -5.721 |
| EPSTI1 |  | 0.08 | 8.624 | 0.996 | 3.19E-01 | 6.13E-01 | -5.722 |
| WDFY2 |  | 0.037 | 7.645 | 0.996 | 3.20E-01 | 6.13E-01 | -5.722 |
| XPO1 |  | 0.05 | 10.249 | 0.995 | 3.20E-01 | 6.13E-01 | -5.723 |
| TUB |  | -0.054 | 6.765 | -0.996 | 3.20E-01 | 6.13E-01 | -5.723 |
| CDH13 |  | -0.064 | 6.123 | -0.996 | 3.20E-01 | 6.13E-01 | -5.723 |
| DGAT1 |  | -0.038 | 8.239 | -0.995 | 3.20E-01 | 6.13E-01 | -5.724 |
| CSRP3 |  | 0.042 | 4.444 | 0.994 | 3.20E-01 | 6.13E-01 | -5.724 |
| MTA1 |  | -0.042 | 9.282 | -0.995 | 3.20E-01 | 6.13E-01 | -5.724 |
| PSMC3 |  | -0.038 | 10.933 | -0.995 | 3.20E-01 | 6.13E-01 | -5.724 |
| ILDR1 |  | -0.066 | 8.2 | -0.994 | 3.21E-01 | 6.14E-01 | -5.724 |
| LHX5 |  | -0.054 | 4.896 | -0.994 | 3.21E-01 | 6.14E-01 | -5.725 |
| HIST1H4A |  | 0.058 | 5.82 | 0.993 | 3.21E-01 | 6.14E-01 | -5.725 |
| BCAN |  | -0.05 | 5.52 | -0.994 | 3.21E-01 | 6.14E-01 | -5.725 |
| CD84 |  | -0.056 | 6.622 | -0.993 | 3.21E-01 | 6.14E-01 | -5.726 |
| GPC5 |  | -0.056 | 4.951 | -0.993 | 3.22E-01 | 6.14E-01 | -5.726 |
| FGD1 |  | -0.054 | 7.368 | -0.992 | 3.22E-01 | 6.15E-01 | -5.727 |
| HSF2BP |  | 0.055 | 5.456 | 0.99 | 3.22E-01 | 6.15E-01 | -5.728 |
| PLCB1 |  | -0.046 | 7.554 | -0.99 | 3.23E-01 | 6.16E-01 | -5.729 |
| ARHGEF11 |  | -0.058 | 6.773 | -0.99 | 3.23E-01 | 6.16E-01 | -5.729 |
| TRHR |  | -0.07 | 5.042 | -0.989 | 3.23E-01 | 6.17E-01 | -5.73 |
| ABCB4 |  | 0.077 | 5.34 | 0.987 | 3.24E-01 | 6.17E-01 | -5.73 |
| ENOSF1 |  | -0.047 | 9.249 | -0.988 | 3.24E-01 | 6.17E-01 | -5.73 |
| CCR9 |  | -0.082 | 5.157 | -0.988 | 3.24E-01 | 6.17E-01 | -5.731 |
| DSC3 |  | -0.053 | 6.498 | -0.988 | 3.24E-01 | 6.17E-01 | -5.731 |
| FGL2 |  | -0.067 | 7.903 | -0.987 | 3.24E-01 | 6.17E-01 | -5.731 |
| FLCN |  | -0.039 | 7.282 | -0.987 | 3.24E-01 | 6.17E-01 | -5.731 |
| PDRG1 |  | 0.034 | 9.233 | 0.986 | 3.24E-01 | 6.18E-01 | -5.732 |
| FMN2 |  | 0.056 | 5.379 | 0.986 | 3.24E-01 | 6.18E-01 | -5.732 |
| CSF3 |  | 0.083 | 5.305 | 0.986 | 3.25E-01 | 6.18E-01 | -5.732 |
| EPHB4 |  | 0.061 | 8.356 | 0.985 | 3.25E-01 | 6.18E-01 | -5.733 |
| FGF20 |  | -0.052 | 5.233 | -0.985 | 3.25E-01 | 6.19E-01 | -5.733 |
| SYT2 |  | -0.047 | 5.528 | -0.985 | 3.26E-01 | 6.19E-01 | -5.734 |
| SIM1 |  | 0.054 | 5.404 | 0.983 | 3.26E-01 | 6.19E-01 | -5.734 |
| KDELR2 |  | -0.036 | 11.235 | -0.984 | 3.26E-01 | 6.19E-01 | -5.735 |
| OGFRL1 |  | 0.047 | 9.248 | 0.982 | 3.26E-01 | 6.20E-01 | -5.735 |
| TWSG1 |  | 0.064 | 7.892 | 0.981 | 3.27E-01 | 6.20E-01 | -5.736 |
| AKAP13 |  | -0.051 | 8.417 | -0.982 | 3.27E-01 | 6.20E-01 | -5.736 |
| SOX18 |  | -0.067 | 8.11 | -0.981 | 3.27E-01 | 6.21E-01 | -5.737 |
| SERPINA4 |  | 0.062 | 4.781 | 0.98 | 3.27E-01 | 6.21E-01 | -5.737 |
| SPATS2 |  | 0.049 | 7.228 | 0.98 | 3.27E-01 | 6.21E-01 | -5.737 |
| SFRP2 |  | 0.075 | 5.665 | 0.98 | 3.27E-01 | 6.21E-01 | -5.737 |
| C6orf203 |  | 0.042 | 8.118 | 0.98 | 3.28E-01 | 6.21E-01 | -5.738 |
| DYSF |  | -0.063 | 10.018 | -0.98 | 3.28E-01 | 6.21E-01 | -5.738 |
| ACY3 |  | -0.058 | 6.422 | -0.98 | 3.28E-01 | 6.21E-01 | -5.738 |
| GNB5 |  | -0.053 | 6.478 | -0.98 | 3.28E-01 | 6.21E-01 | -5.738 |
| MAT1A |  | 0.061 | 5.158 | 0.978 | 3.28E-01 | 6.21E-01 | -5.739 |
| SH2D2A |  | 0.055 | 6.838 | 0.978 | 3.28E-01 | 6.21E-01 | -5.739 |
| FOXP2 |  | -0.036 | 4.799 | -0.978 | 3.28E-01 | 6.22E-01 | -5.739 |
| KIAA1407 |  | 0.059 | 6.572 | 0.977 | 3.29E-01 | 6.22E-01 | -5.74 |
| SYT12 |  | -0.052 | 5.924 | -0.978 | 3.29E-01 | 6.22E-01 | -5.74 |
| CD1C |  | 0.053 | 6.052 | 0.977 | 3.29E-01 | 6.22E-01 | -5.74 |
| ZMAT2 |  | 0.044 | 10.167 | 0.976 | 3.29E-01 | 6.22E-01 | -5.741 |
| AMD1 |  | 0.06 | 10.855 | 0.976 | 3.29E-01 | 6.23E-01 | -5.741 |
| IL1R1 |  | 0.069 | 9.071 | 0.976 | 3.29E-01 | 6.23E-01 | -5.741 |
| RPL23A |  | -0.039 | 11.645 | -0.976 | 3.30E-01 | 6.23E-01 | -5.742 |
| ZNF645 |  | 0.054 | 4.887 | 0.974 | 3.30E-01 | 6.23E-01 | -5.742 |
| VSIG4 |  | -0.091 | 9.227 | -0.974 | 3.31E-01 | 6.24E-01 | -5.743 |
| NISCH |  | 0.049 | 10.03 | 0.973 | 3.31E-01 | 6.24E-01 | -5.744 |
| ARHGEF1 |  | 0.05 | 7.594 | 0.973 | 3.31E-01 | 6.24E-01 | -5.744 |
| WBP4 |  | 0.06 | 8.569 | 0.973 | 3.31E-01 | 6.24E-01 | -5.744 |
| HMP19 |  | 0.067 | 5.66 | 0.973 | 3.31E-01 | 6.24E-01 | -5.744 |
| RAD51 |  | -0.061 | 7.941 | -0.974 | 3.31E-01 | 6.24E-01 | -5.744 |
| GPR101 |  | 0.052 | 4.802 | 0.972 | 3.31E-01 | 6.24E-01 | -5.744 |
| HIVEP3 |  | 0.038 | 6.896 | 0.971 | 3.32E-01 | 6.25E-01 | -5.746 |
| SLC35A3 |  | 0.048 | 8.042 | 0.971 | 3.32E-01 | 6.26E-01 | -5.746 |
| H6PD |  | 0.06 | 6.946 | 0.97 | 3.32E-01 | 6.26E-01 | -5.746 |
| DTYMK |  | 0.038 | 8.885 | 0.97 | 3.32E-01 | 6.26E-01 | -5.747 |
| SH3RF2 |  | -0.054 | 5.549 | -0.971 | 3.32E-01 | 6.26E-01 | -5.747 |
| EPRS |  | -0.044 | 10.625 | -0.97 | 3.32E-01 | 6.26E-01 | -5.747 |
| TAF5L |  | 0.046 | 6.518 | 0.969 | 3.33E-01 | 6.26E-01 | -5.748 |
| PLXDC1 |  | -0.054 | 6.404 | -0.969 | 3.33E-01 | 6.26E-01 | -5.748 |
| ZNF596 |  | -0.052 | 5.91 | -0.969 | 3.33E-01 | 6.26E-01 | -5.748 |
| PPOX |  | -0.041 | 8.84 | -0.969 | 3.33E-01 | 6.26E-01 | -5.748 |
| ZDHHC8 |  | -0.055 | 9.574 | -0.969 | 3.33E-01 | 6.26E-01 | -5.748 |
| ACSL6 |  | 0.041 | 5.24 | 0.968 | 3.33E-01 | 6.26E-01 | -5.748 |
| EDAR |  | -0.062 | 5.6 | -0.969 | 3.33E-01 | 6.26E-01 | -5.749 |
| RRAGD |  | -0.065 | 6.983 | -0.969 | 3.33E-01 | 6.26E-01 | -5.749 |
| DSG3 |  | -0.064 | 4.768 | -0.968 | 3.33E-01 | 6.26E-01 | -5.749 |
| CCR2 |  | -0.048 | 6.142 | -0.968 | 3.34E-01 | 6.26E-01 | -5.749 |
| SRGAP3 |  | 0.054 | 5.172 | 0.967 | 3.34E-01 | 6.26E-01 | -5.749 |
| BICD1 |  | 0.057 | 6.529 | 0.967 | 3.34E-01 | 6.26E-01 | -5.749 |
| MDGA1 |  | 0.066 | 5.95 | 0.967 | 3.34E-01 | 6.26E-01 | -5.749 |
| SNTG2 |  | 0.06 | 4.819 | 0.967 | 3.34E-01 | 6.26E-01 | -5.75 |
| LRRC2 |  | 0.063 | 5.517 | 0.966 | 3.34E-01 | 6.26E-01 | -5.75 |
| HIPK1 |  | 0.046 | 7.191 | 0.966 | 3.34E-01 | 6.26E-01 | -5.75 |
| MCFD2 |  | 0.047 | 9.398 | 0.966 | 3.34E-01 | 6.26E-01 | -5.751 |
| IL1RAPL2 |  | 0.055 | 5.284 | 0.966 | 3.34E-01 | 6.26E-01 | -5.751 |
| SSH1 |  | 0.041 | 7.122 | 0.965 | 3.35E-01 | 6.26E-01 | -5.751 |
| GNB4 |  | 0.066 | 8.091 | 0.965 | 3.35E-01 | 6.26E-01 | -5.751 |
| TRIM7 |  | 0.051 | 6.007 | 0.964 | 3.35E-01 | 6.27E-01 | -5.752 |
| SST |  | -0.075 | 5.013 | -0.965 | 3.35E-01 | 6.27E-01 | -5.752 |
| POU4F2 |  | -0.071 | 5.699 | -0.965 | 3.35E-01 | 6.27E-01 | -5.752 |
| C1QBP |  | -0.036 | 11.606 | -0.964 | 3.36E-01 | 6.28E-01 | -5.753 |
| CLNS1A |  | 0.033 | 10.556 | 0.963 | 3.36E-01 | 6.28E-01 | -5.753 |
| NCR2 |  | -0.062 | 5.826 | -0.964 | 3.36E-01 | 6.28E-01 | -5.753 |
| SPATA12 |  | 0.059 | 5.165 | 0.962 | 3.36E-01 | 6.28E-01 | -5.754 |
| SLC27A5 |  | 0.054 | 7.364 | 0.962 | 3.36E-01 | 6.28E-01 | -5.754 |
| ZNF189 |  | -0.04 | 7.962 | -0.963 | 3.36E-01 | 6.28E-01 | -5.754 |
| IPO8 |  | -0.058 | 7.675 | -0.962 | 3.37E-01 | 6.28E-01 | -5.755 |
| ARHGAP5 |  | 0.04 | 7.638 | 0.961 | 3.37E-01 | 6.28E-01 | -5.755 |
| ALOX12 |  | -0.067 | 6.099 | -0.962 | 3.37E-01 | 6.29E-01 | -5.755 |
| CRB1 |  | -0.048 | 4.814 | -0.962 | 3.37E-01 | 6.29E-01 | -5.755 |
| MS4A12 |  | -0.062 | 4.718 | -0.961 | 3.37E-01 | 6.29E-01 | -5.756 |
| C1orf35 |  | 0.036 | 9.562 | 0.96 | 3.37E-01 | 6.29E-01 | -5.756 |
| GTF2F2 |  | 0.037 | 9.811 | 0.959 | 3.38E-01 | 6.29E-01 | -5.756 |
| UBQLN3 |  | -0.063 | 5.682 | -0.96 | 3.38E-01 | 6.30E-01 | -5.757 |
| TWIST2 |  | 0.064 | 7.357 | 0.958 | 3.38E-01 | 6.30E-01 | -5.757 |
| HPSE2 |  | -0.058 | 5.261 | -0.959 | 3.38E-01 | 6.30E-01 | -5.758 |
| TNFRSF13B |  | 0.063 | 5.43 | 0.958 | 3.38E-01 | 6.30E-01 | -5.758 |
| SYT3 |  | 0.058 | 6.417 | 0.957 | 3.39E-01 | 6.30E-01 | -5.759 |
| ITGAM |  | -0.053 | 7.834 | -0.958 | 3.39E-01 | 6.30E-01 | -5.759 |
| YARS |  | -0.039 | 9.346 | -0.958 | 3.39E-01 | 6.30E-01 | -5.759 |
| EIF4B |  | 0.034 | 10.527 | 0.957 | 3.39E-01 | 6.30E-01 | -5.759 |
| DDX4 |  | 0.066 | 4.622 | 0.957 | 3.39E-01 | 6.30E-01 | -5.759 |
| TREM2 |  | 0.066 | 6.263 | 0.957 | 3.39E-01 | 6.30E-01 | -5.759 |
| HRH1 |  | -0.049 | 6.519 | -0.957 | 3.39E-01 | 6.30E-01 | -5.759 |
| DIRC2 |  | -0.05 | 10.396 | -0.957 | 3.39E-01 | 6.30E-01 | -5.759 |
| PDE10A |  | -0.035 | 5.556 | -0.956 | 3.40E-01 | 6.31E-01 | -5.76 |
| HPSE |  | -0.087 | 7.502 | -0.956 | 3.40E-01 | 6.31E-01 | -5.76 |
| SRY |  | 0.052 | 4.879 | 0.955 | 3.40E-01 | 6.31E-01 | -5.761 |
| ALDH1A2 |  | 0.102 | 5.782 | 0.955 | 3.40E-01 | 6.31E-01 | -5.761 |
| FBXO34 |  | -0.038 | 8.625 | -0.956 | 3.40E-01 | 6.31E-01 | -5.761 |
| KIF12 |  | 0.065 | 5.09 | 0.954 | 3.40E-01 | 6.31E-01 | -5.761 |
| PNMA3 |  | -0.059 | 5.619 | -0.955 | 3.40E-01 | 6.31E-01 | -5.761 |
| TEX12 |  | 0.061 | 4.765 | 0.954 | 3.40E-01 | 6.31E-01 | -5.761 |
| MFHAS1 |  | 0.049 | 7.172 | 0.953 | 3.41E-01 | 6.32E-01 | -5.762 |
| G6PC2 |  | 0.051 | 5.543 | 0.952 | 3.41E-01 | 6.32E-01 | -5.763 |
| RTTN |  | -0.045 | 6.852 | -0.953 | 3.41E-01 | 6.32E-01 | -5.763 |
| PKMYT1 |  | -0.056 | 8.206 | -0.953 | 3.41E-01 | 6.32E-01 | -5.763 |
| FAM19A5 |  | -0.08 | 5.087 | -0.952 | 3.42E-01 | 6.33E-01 | -5.764 |
| CTDSP1 |  | 0.034 | 11.054 | 0.951 | 3.42E-01 | 6.33E-01 | -5.764 |
| TRIM11 |  | -0.036 | 8.579 | -0.952 | 3.42E-01 | 6.33E-01 | -5.764 |
| PAN3 |  | -0.043 | 8.373 | -0.952 | 3.42E-01 | 6.33E-01 | -5.764 |
| H2AFY2 |  | -0.062 | 6.901 | -0.951 | 3.42E-01 | 6.33E-01 | -5.765 |
| CPLX2 |  | -0.051 | 5.856 | -0.951 | 3.42E-01 | 6.33E-01 | -5.765 |
| NAGS |  | -0.051 | 7.059 | -0.95 | 3.43E-01 | 6.33E-01 | -5.766 |
| CHKB |  | -0.042 | 8.779 | -0.95 | 3.43E-01 | 6.33E-01 | -5.766 |
| LAIR1 |  | -0.05 | 8.074 | -0.95 | 3.43E-01 | 6.33E-01 | -5.766 |
| HCFC1 |  | -0.049 | 8.504 | -0.95 | 3.43E-01 | 6.33E-01 | -5.766 |
| AIG1 |  | -0.051 | 8.118 | -0.949 | 3.43E-01 | 6.33E-01 | -5.766 |
| RCOR2 |  | 0.054 | 7.715 | 0.948 | 3.43E-01 | 6.33E-01 | -5.766 |
| BNC1 |  | -0.045 | 6.149 | -0.949 | 3.43E-01 | 6.33E-01 | -5.766 |
| SH3YL1 |  | 0.063 | 7.659 | 0.948 | 3.43E-01 | 6.33E-01 | -5.767 |
| WNT8A |  | 0.048 | 4.651 | 0.948 | 3.43E-01 | 6.33E-01 | -5.767 |
| FZD2 |  | -0.064 | 8.439 | -0.949 | 3.43E-01 | 6.33E-01 | -5.767 |
| PRPF39 |  | 0.044 | 7.857 | 0.948 | 3.43E-01 | 6.33E-01 | -5.767 |
| MOS |  | -0.057 | 6.665 | -0.949 | 3.43E-01 | 6.33E-01 | -5.767 |
| HEBP2 |  | 0.057 | 11.257 | 0.947 | 3.44E-01 | 6.34E-01 | -5.768 |
| PLXNB3 |  | 0.056 | 5.289 | 0.947 | 3.44E-01 | 6.34E-01 | -5.768 |
| SERPINA1 |  | -0.058 | 7.776 | -0.947 | 3.44E-01 | 6.34E-01 | -5.768 |
| SLC22A9 |  | -0.05 | 4.936 | -0.947 | 3.44E-01 | 6.34E-01 | -5.769 |
| PSMD12 |  | 0.04 | 8.934 | 0.946 | 3.44E-01 | 6.34E-01 | -5.769 |
| SRP72 |  | -0.046 | 9.972 | -0.947 | 3.44E-01 | 6.34E-01 | -5.769 |
| SOX4 |  | 0.06 | 7.743 | 0.946 | 3.44E-01 | 6.34E-01 | -5.769 |
| UBE2C |  | 0.056 | 8.757 | 0.946 | 3.44E-01 | 6.34E-01 | -5.769 |
| CLSPN |  | -0.062 | 5.618 | -0.946 | 3.45E-01 | 6.34E-01 | -5.769 |
| FSHB |  | 0.046 | 4.828 | 0.945 | 3.45E-01 | 6.34E-01 | -5.77 |
| SCGN |  | -0.097 | 6.215 | -0.946 | 3.45E-01 | 6.34E-01 | -5.77 |
| TCF4 |  | 0.047 | 9.771 | 0.945 | 3.45E-01 | 6.34E-01 | -5.77 |
| RANBP2 |  | 0.04 | 8.264 | 0.944 | 3.45E-01 | 6.34E-01 | -5.77 |
| SEZ6 |  | -0.059 | 5.502 | -0.945 | 3.45E-01 | 6.34E-01 | -5.77 |
| ZDHHC7 |  | 0.03 | 10.477 | 0.944 | 3.45E-01 | 6.34E-01 | -5.771 |
| CCBE1 |  | 0.04 | 5.18 | 0.944 | 3.45E-01 | 6.34E-01 | -5.771 |
| ERBB2 |  | 0.04 | 8.873 | 0.944 | 3.46E-01 | 6.34E-01 | -5.771 |
| PDGFC |  | 0.067 | 7.797 | 0.943 | 3.46E-01 | 6.34E-01 | -5.771 |
| CA2 |  | -0.093 | 7.779 | -0.944 | 3.46E-01 | 6.35E-01 | -5.771 |
| PHACTR2 |  | 0.041 | 9.52 | 0.942 | 3.46E-01 | 6.35E-01 | -5.772 |
| ALG9 |  | -0.042 | 8.462 | -0.943 | 3.46E-01 | 6.35E-01 | -5.772 |
| OR10H1 |  | -0.055 | 5.934 | -0.943 | 3.46E-01 | 6.35E-01 | -5.772 |
| RRBP1 |  | 0.067 | 9.579 | 0.942 | 3.47E-01 | 6.35E-01 | -5.773 |
| HNF4A |  | -0.042 | 4.795 | -0.942 | 3.47E-01 | 6.35E-01 | -5.773 |
| GUCY2C |  | 0.055 | 4.769 | 0.941 | 3.47E-01 | 6.35E-01 | -5.773 |
| CABP1 |  | -0.052 | 5.048 | -0.942 | 3.47E-01 | 6.35E-01 | -5.773 |
| FAM53C |  | -0.039 | 9.166 | -0.942 | 3.47E-01 | 6.35E-01 | -5.773 |
| MAP6 |  | 0.051 | 5.824 | 0.941 | 3.47E-01 | 6.35E-01 | -5.773 |
| IL1RAP |  | 0.059 | 8.449 | 0.94 | 3.47E-01 | 6.35E-01 | -5.774 |
| UBE2R2 |  | 0.032 | 8.612 | 0.94 | 3.47E-01 | 6.35E-01 | -5.774 |
| IDI2 |  | 0.059 | 5.227 | 0.94 | 3.47E-01 | 6.35E-01 | -5.774 |
| DISC1 |  | 0.035 | 6.578 | 0.94 | 3.48E-01 | 6.35E-01 | -5.774 |
| TRH |  | 0.05 | 5.32 | 0.939 | 3.48E-01 | 6.36E-01 | -5.775 |
| SLC6A11 |  | 0.042 | 4.862 | 0.938 | 3.48E-01 | 6.36E-01 | -5.776 |
| PARD6A |  | 0.054 | 6.596 | 0.938 | 3.48E-01 | 6.36E-01 | -5.776 |
| NRG1 |  | 0.043 | 5.94 | 0.937 | 3.49E-01 | 6.37E-01 | -5.776 |
| SLC25A10 |  | 0.049 | 6.68 | 0.937 | 3.49E-01 | 6.37E-01 | -5.777 |
| CASP6 |  | -0.038 | 8.108 | -0.937 | 3.49E-01 | 6.37E-01 | -5.777 |
| ZNF184 |  | -0.042 | 7.31 | -0.937 | 3.49E-01 | 6.37E-01 | -5.778 |
| CD83 |  | 0.065 | 7.69 | 0.935 | 3.50E-01 | 6.38E-01 | -5.778 |
| RBM7 |  | -0.061 | 8.706 | -0.936 | 3.50E-01 | 6.38E-01 | -5.778 |
| PFKFB2 |  | -0.039 | 7.2 | -0.936 | 3.50E-01 | 6.38E-01 | -5.778 |
| ARPC1A |  | 0.044 | 11.091 | 0.935 | 3.50E-01 | 6.38E-01 | -5.779 |
| IL13 |  | 0.049 | 4.752 | 0.935 | 3.50E-01 | 6.38E-01 | -5.779 |
| C14orf93 |  | 0.039 | 8.16 | 0.935 | 3.50E-01 | 6.38E-01 | -5.779 |
| NOL3 |  | 0.047 | 8.427 | 0.934 | 3.50E-01 | 6.38E-01 | -5.779 |
| POLE2 |  | 0.053 | 6.841 | 0.934 | 3.50E-01 | 6.38E-01 | -5.779 |
| COX11 |  | 0.031 | 7.612 | 0.934 | 3.51E-01 | 6.38E-01 | -5.78 |
| CACNG2 |  | 0.044 | 4.871 | 0.933 | 3.51E-01 | 6.38E-01 | -5.78 |
| PSMA3 |  | -0.042 | 11.255 | -0.934 | 3.51E-01 | 6.39E-01 | -5.78 |
| DPYS |  | 0.046 | 5.062 | 0.932 | 3.51E-01 | 6.39E-01 | -5.781 |
| CTNNBIP1 |  | 0.045 | 8.958 | 0.932 | 3.51E-01 | 6.39E-01 | -5.781 |
| DCTN3 |  | 0.033 | 10.115 | 0.932 | 3.51E-01 | 6.39E-01 | -5.781 |
| SH3GL1 |  | 0.037 | 10.081 | 0.932 | 3.52E-01 | 6.39E-01 | -5.781 |
| ST7L |  | 0.042 | 6.516 | 0.932 | 3.52E-01 | 6.39E-01 | -5.781 |
| ELOVL4 |  | -0.07 | 6.511 | -0.933 | 3.52E-01 | 6.39E-01 | -5.781 |
| CTNNBL1 |  | -0.031 | 9.962 | -0.932 | 3.52E-01 | 6.39E-01 | -5.782 |
| RPLP2 |  | 0.031 | 14.381 | 0.931 | 3.52E-01 | 6.39E-01 | -5.782 |
| SPATA8 |  | -0.053 | 5.495 | -0.931 | 3.52E-01 | 6.39E-01 | -5.783 |
| STARD3NL |  | -0.05 | 9.671 | -0.931 | 3.52E-01 | 6.39E-01 | -5.783 |
| KLF13 |  | -0.038 | 8.949 | -0.931 | 3.52E-01 | 6.39E-01 | -5.783 |
| PTTG1 |  | 0.063 | 9.527 | 0.93 | 3.53E-01 | 6.40E-01 | -5.783 |
| FCGBP |  | -0.081 | 8.348 | -0.93 | 3.53E-01 | 6.40E-01 | -5.783 |
| CPD |  | -0.047 | 9.526 | -0.93 | 3.53E-01 | 6.40E-01 | -5.784 |
| SLC28A2 |  | -0.082 | 5.529 | -0.93 | 3.53E-01 | 6.40E-01 | -5.784 |
| LMX1A |  | -0.055 | 5.185 | -0.93 | 3.53E-01 | 6.40E-01 | -5.784 |
| DNAL4 |  | -0.052 | 8.973 | -0.93 | 3.53E-01 | 6.40E-01 | -5.784 |
| ZNF101 |  | 0.03 | 7.6 | 0.928 | 3.53E-01 | 6.40E-01 | -5.784 |
| COPB2 |  | -0.028 | 11.46 | -0.929 | 3.53E-01 | 6.40E-01 | -5.785 |
| SYT7 |  | 0.047 | 6.634 | 0.928 | 3.54E-01 | 6.40E-01 | -5.785 |
| PRSS3 |  | -0.067 | 6.826 | -0.928 | 3.54E-01 | 6.40E-01 | -5.786 |
| LHFPL3 |  | -0.053 | 4.963 | -0.927 | 3.54E-01 | 6.41E-01 | -5.786 |
| MYOZ1 |  | 0.069 | 5.883 | 0.926 | 3.55E-01 | 6.41E-01 | -5.787 |
| DHRS7 |  | -0.044 | 9.869 | -0.926 | 3.55E-01 | 6.42E-01 | -5.787 |
| CRTAP |  | -0.041 | 7.56 | -0.926 | 3.55E-01 | 6.42E-01 | -5.787 |
| EPB41L3 |  | -0.045 | 9.276 | -0.926 | 3.55E-01 | 6.42E-01 | -5.788 |
| PLCL2 |  | -0.06 | 9.374 | -0.926 | 3.55E-01 | 6.42E-01 | -5.788 |
| RPS6 |  | 0.034 | 13.508 | 0.924 | 3.55E-01 | 6.42E-01 | -5.788 |
| FAAH |  | -0.054 | 6.577 | -0.925 | 3.55E-01 | 6.42E-01 | -5.788 |
| ACTL7A |  | -0.057 | 5.79 | -0.925 | 3.56E-01 | 6.42E-01 | -5.788 |
| SPARCL1 |  | -0.074 | 9.128 | -0.924 | 3.56E-01 | 6.43E-01 | -5.789 |
| RBPMS |  | 0.039 | 7.533 | 0.923 | 3.56E-01 | 6.43E-01 | -5.789 |
| MCOLN3 |  | -0.066 | 6 | -0.924 | 3.56E-01 | 6.43E-01 | -5.79 |
| FBXW7 |  | 0.031 | 8.364 | 0.922 | 3.57E-01 | 6.43E-01 | -5.79 |
| EIF1AY |  | -0.177 | 6.996 | -0.923 | 3.57E-01 | 6.43E-01 | -5.79 |
| CEACAM4 |  | -0.078 | 7.487 | -0.922 | 3.57E-01 | 6.43E-01 | -5.791 |
| GRB14 |  | -0.049 | 7.742 | -0.922 | 3.57E-01 | 6.43E-01 | -5.791 |
| FXR2 |  | 0.056 | 7.761 | 0.921 | 3.57E-01 | 6.43E-01 | -5.791 |
| HCP5 |  | -0.061 | 7.839 | -0.922 | 3.57E-01 | 6.43E-01 | -5.791 |
| CRYBB2 |  | -0.069 | 7.158 | -0.921 | 3.58E-01 | 6.44E-01 | -5.792 |
| SPHK1 |  | -0.058 | 7.817 | -0.921 | 3.58E-01 | 6.44E-01 | -5.792 |
| SLC35B3 |  | -0.045 | 8.668 | -0.921 | 3.58E-01 | 6.44E-01 | -5.792 |
| CDT1 |  | 0.061 | 8.087 | 0.92 | 3.58E-01 | 6.44E-01 | -5.792 |
| LAMC1 |  | 0.055 | 11.095 | 0.92 | 3.58E-01 | 6.44E-01 | -5.792 |
| HAPLN4 |  | -0.055 | 5.371 | -0.92 | 3.58E-01 | 6.44E-01 | -5.793 |
| SPRY1 |  | 0.055 | 7.877 | 0.919 | 3.58E-01 | 6.44E-01 | -5.793 |
| SLC6A7 |  | 0.048 | 5.16 | 0.919 | 3.58E-01 | 6.44E-01 | -5.793 |
| BTG3 |  | -0.052 | 10.088 | -0.92 | 3.58E-01 | 6.44E-01 | -5.793 |
| ADH1C |  | -0.061 | 5.829 | -0.919 | 3.59E-01 | 6.44E-01 | -5.794 |
| RANBP10 |  | -0.05 | 8.635 | -0.919 | 3.59E-01 | 6.44E-01 | -5.794 |
| SPINK5 |  | 0.058 | 6.055 | 0.918 | 3.59E-01 | 6.44E-01 | -5.794 |
| F2R |  | 0.084 | 7.572 | 0.918 | 3.59E-01 | 6.44E-01 | -5.794 |
| ZNF547 |  | 0.042 | 5.629 | 0.917 | 3.59E-01 | 6.45E-01 | -5.794 |
| SUPT5H |  | 0.041 | 9.188 | 0.917 | 3.59E-01 | 6.45E-01 | -5.795 |
| HIPK4 |  | -0.056 | 5.25 | -0.917 | 3.60E-01 | 6.46E-01 | -5.796 |
| ABCC2 |  | 0.055 | 6.147 | 0.915 | 3.60E-01 | 6.46E-01 | -5.796 |
| IL2RA |  | -0.065 | 6.225 | -0.916 | 3.60E-01 | 6.46E-01 | -5.796 |
| DDX1 |  | 0.034 | 11.328 | 0.915 | 3.60E-01 | 6.46E-01 | -5.797 |
| FGFBP1 |  | -0.104 | 5.879 | -0.915 | 3.61E-01 | 6.46E-01 | -5.797 |
| SEMA6D |  | -0.045 | 6.132 | -0.915 | 3.61E-01 | 6.46E-01 | -5.797 |
| TAX1BP1 |  | 0.046 | 11.605 | 0.914 | 3.61E-01 | 6.46E-01 | -5.797 |
| IL4R |  | 0.041 | 9.704 | 0.914 | 3.61E-01 | 6.46E-01 | -5.797 |
| CORO1B |  | -0.045 | 8.866 | -0.915 | 3.61E-01 | 6.46E-01 | -5.797 |
| AVPR1A |  | -0.06 | 4.874 | -0.915 | 3.61E-01 | 6.46E-01 | -5.797 |
| SFTPD |  | -0.051 | 5.994 | -0.915 | 3.61E-01 | 6.46E-01 | -5.798 |
| MELK |  | 0.061 | 7.928 | 0.913 | 3.61E-01 | 6.46E-01 | -5.798 |
| DMC1 |  | -0.05 | 5.274 | -0.914 | 3.61E-01 | 6.46E-01 | -5.798 |
| VILL |  | 0.041 | 7.156 | 0.913 | 3.62E-01 | 6.46E-01 | -5.798 |
| UBE3C |  | 0.034 | 8.282 | 0.912 | 3.62E-01 | 6.46E-01 | -5.799 |
| ST8SIA1 |  | 0.056 | 5.501 | 0.912 | 3.62E-01 | 6.46E-01 | -5.799 |
| PPP2R3A |  | -0.038 | 7.526 | -0.913 | 3.62E-01 | 6.46E-01 | -5.799 |
| IL4 |  | 0.056 | 4.852 | 0.912 | 3.62E-01 | 6.46E-01 | -5.799 |
| KCNJ16 |  | 0.095 | 6.008 | 0.912 | 3.62E-01 | 6.46E-01 | -5.799 |
| TNFSF18 |  | -0.053 | 4.778 | -0.913 | 3.62E-01 | 6.46E-01 | -5.799 |
| GNPNAT1 |  | 0.042 | 7.379 | 0.911 | 3.62E-01 | 6.46E-01 | -5.8 |
| GLRB |  | -0.05 | 5.673 | -0.912 | 3.62E-01 | 6.46E-01 | -5.8 |
| PDE4B |  | 0.055 | 6.985 | 0.911 | 3.62E-01 | 6.46E-01 | -5.8 |
| GJB4 |  | -0.049 | 5.861 | -0.912 | 3.63E-01 | 6.46E-01 | -5.8 |
| PLA2G2E |  | 0.05 | 4.956 | 0.91 | 3.63E-01 | 6.46E-01 | -5.8 |
| HSPB7 |  | 0.042 | 5.711 | 0.91 | 3.63E-01 | 6.46E-01 | -5.8 |
| SLC5A2 |  | -0.051 | 6.873 | -0.911 | 3.63E-01 | 6.47E-01 | -5.801 |
| KCNB1 |  | 0.056 | 5.184 | 0.909 | 3.63E-01 | 6.47E-01 | -5.801 |
| USP13 |  | -0.045 | 7.714 | -0.91 | 3.63E-01 | 6.47E-01 | -5.802 |
| AIRE |  | -0.041 | 6.368 | -0.91 | 3.63E-01 | 6.47E-01 | -5.802 |
| MS4A5 |  | -0.057 | 4.979 | -0.91 | 3.64E-01 | 6.47E-01 | -5.802 |
| COL9A2 |  | 0.057 | 6.022 | 0.908 | 3.64E-01 | 6.48E-01 | -5.802 |
| COL23A1 |  | -0.057 | 6.409 | -0.909 | 3.64E-01 | 6.48E-01 | -5.803 |
| RRH |  | -0.057 | 6.051 | -0.908 | 3.64E-01 | 6.48E-01 | -5.803 |
| TCERG1 |  | -0.036 | 8.514 | -0.908 | 3.65E-01 | 6.48E-01 | -5.804 |
| DOK1 |  | 0.039 | 7.932 | 0.906 | 3.65E-01 | 6.48E-01 | -5.804 |
| KPNA6 |  | -0.044 | 9.712 | -0.907 | 3.65E-01 | 6.48E-01 | -5.804 |
| GLS2 |  | -0.052 | 5.887 | -0.907 | 3.65E-01 | 6.48E-01 | -5.804 |
| RALBP1 |  | 0.038 | 9.903 | 0.906 | 3.65E-01 | 6.48E-01 | -5.804 |
| ZNF382 |  | 0.061 | 5.619 | 0.906 | 3.65E-01 | 6.48E-01 | -5.804 |
| DSP |  | 0.068 | 9.345 | 0.906 | 3.65E-01 | 6.48E-01 | -5.804 |
| CDC14A |  | -0.049 | 6.066 | -0.907 | 3.65E-01 | 6.48E-01 | -5.805 |
| AKR1C4 |  | 0.04 | 4.948 | 0.905 | 3.66E-01 | 6.49E-01 | -5.805 |
| MTHFD1 |  | 0.038 | 9.176 | 0.904 | 3.66E-01 | 6.49E-01 | -5.806 |
| MMP20 |  | -0.05 | 4.7 | -0.905 | 3.66E-01 | 6.49E-01 | -5.806 |
| CAV2 |  | -0.052 | 8.857 | -0.905 | 3.66E-01 | 6.49E-01 | -5.806 |
| PLS3 |  | -0.052 | 8.99 | -0.904 | 3.67E-01 | 6.49E-01 | -5.807 |
| NPM2 |  | 0.052 | 5.909 | 0.903 | 3.67E-01 | 6.49E-01 | -5.807 |
| SLC22A18 |  | 0.05 | 8.153 | 0.903 | 3.67E-01 | 6.49E-01 | -5.807 |
| FGF23 |  | 0.048 | 4.883 | 0.903 | 3.67E-01 | 6.49E-01 | -5.807 |
| GALE |  | 0.052 | 7.863 | 0.903 | 3.67E-01 | 6.49E-01 | -5.807 |
| PRPF8 |  | -0.042 | 10.704 | -0.903 | 3.67E-01 | 6.49E-01 | -5.807 |
| CDC25A |  | -0.053 | 7.076 | -0.903 | 3.67E-01 | 6.49E-01 | -5.807 |
| TMEM33 |  | -0.04 | 7.654 | -0.903 | 3.67E-01 | 6.50E-01 | -5.808 |
| BLVRB |  | 0.049 | 10.499 | 0.901 | 3.68E-01 | 6.50E-01 | -5.809 |
| BRCA1 |  | 0.041 | 7.259 | 0.901 | 3.68E-01 | 6.50E-01 | -5.809 |
| IFI44L |  | 0.105 | 8.274 | 0.901 | 3.68E-01 | 6.50E-01 | -5.809 |
| SERPINB5 |  | -0.048 | 5.123 | -0.901 | 3.68E-01 | 6.50E-01 | -5.809 |
| S100A14 |  | 0.076 | 5.443 | 0.899 | 3.69E-01 | 6.51E-01 | -5.81 |
| MRPL33 |  | 0.038 | 10.818 | 0.899 | 3.69E-01 | 6.51E-01 | -5.81 |
| DLGAP4 |  | 0.045 | 8.378 | 0.899 | 3.69E-01 | 6.51E-01 | -5.81 |
| KCNJ15 |  | -0.049 | 6.955 | -0.9 | 3.69E-01 | 6.51E-01 | -5.81 |
| LAMB3 |  | -0.074 | 7.733 | -0.899 | 3.69E-01 | 6.51E-01 | -5.811 |
| CCL1 |  | 0.043 | 5.582 | 0.898 | 3.69E-01 | 6.51E-01 | -5.811 |
| SENP2 |  | 0.053 | 8.509 | 0.898 | 3.69E-01 | 6.51E-01 | -5.811 |
| FZD5 |  | -0.06 | 6.979 | -0.899 | 3.69E-01 | 6.51E-01 | -5.811 |
| PNLIPRP2 |  | 0.049 | 5.008 | 0.898 | 3.69E-01 | 6.51E-01 | -5.811 |
| LRBA |  | 0.035 | 7.897 | 0.897 | 3.70E-01 | 6.51E-01 | -5.812 |
| RAD51C |  | 0.035 | 8.516 | 0.897 | 3.70E-01 | 6.51E-01 | -5.812 |
| KRTAP4-5 |  | -0.054 | 5.102 | -0.898 | 3.70E-01 | 6.51E-01 | -5.812 |
| SPAG16 |  | 0.039 | 6.289 | 0.897 | 3.70E-01 | 6.51E-01 | -5.812 |
| SMAD2 |  | 0.026 | 7.821 | 0.896 | 3.70E-01 | 6.52E-01 | -5.813 |
| MYO10 |  | 0.045 | 8.285 | 0.896 | 3.70E-01 | 6.52E-01 | -5.813 |
| DDX23 |  | 0.032 | 10.056 | 0.896 | 3.71E-01 | 6.52E-01 | -5.813 |
| COMMD2 |  | -0.04 | 8.396 | -0.897 | 3.71E-01 | 6.52E-01 | -5.813 |
| NAPA |  | -0.051 | 8.548 | -0.897 | 3.71E-01 | 6.52E-01 | -5.813 |
| DNAJC4 |  | 0.039 | 7.796 | 0.895 | 3.71E-01 | 6.52E-01 | -5.813 |
| TGFBR1 |  | 0.05 | 6.659 | 0.895 | 3.71E-01 | 6.52E-01 | -5.814 |
| ASB4 |  | 0.046 | 5.535 | 0.895 | 3.71E-01 | 6.52E-01 | -5.814 |
| CSMD3 |  | -0.055 | 4.729 | -0.895 | 3.71E-01 | 6.53E-01 | -5.814 |
| CLEC12A |  | -0.062 | 6.249 | -0.894 | 3.72E-01 | 6.53E-01 | -5.815 |
| SORCS1 |  | -0.043 | 4.894 | -0.894 | 3.72E-01 | 6.53E-01 | -5.815 |
| SDHD |  | -0.044 | 10.28 | -0.894 | 3.72E-01 | 6.53E-01 | -5.815 |
| ZNF337 |  | 0.032 | 7.792 | 0.893 | 3.72E-01 | 6.53E-01 | -5.816 |
| KLF8 |  | 0.05 | 7.102 | 0.892 | 3.72E-01 | 6.54E-01 | -5.816 |
| AP1M2 |  | -0.058 | 7.802 | -0.893 | 3.72E-01 | 6.54E-01 | -5.816 |
| AKAP5 |  | -0.051 | 5.006 | -0.893 | 3.73E-01 | 6.54E-01 | -5.816 |
| LETM2 |  | 0.046 | 6.096 | 0.892 | 3.73E-01 | 6.54E-01 | -5.816 |
| TGM4 |  | -0.048 | 4.841 | -0.893 | 3.73E-01 | 6.54E-01 | -5.817 |
| PIK3C2A |  | -0.073 | 7.757 | -0.892 | 3.73E-01 | 6.54E-01 | -5.817 |
| CLEC2B |  | 0.068 | 7.97 | 0.891 | 3.73E-01 | 6.54E-01 | -5.817 |
| CHCHD6 |  | 0.041 | 7.614 | 0.89 | 3.73E-01 | 6.54E-01 | -5.818 |
| CACNA1B |  | -0.047 | 5.638 | -0.891 | 3.74E-01 | 6.54E-01 | -5.818 |
| PRDM13 |  | -0.05 | 5.015 | -0.891 | 3.74E-01 | 6.54E-01 | -5.818 |
| SOX1 |  | -0.045 | 5.635 | -0.89 | 3.74E-01 | 6.54E-01 | -5.818 |
| RBAK |  | -0.056 | 6.222 | -0.89 | 3.74E-01 | 6.54E-01 | -5.818 |
| ADRB2 |  | -0.057 | 8.605 | -0.89 | 3.74E-01 | 6.55E-01 | -5.819 |
| CHI3L1 |  | -0.087 | 7.296 | -0.89 | 3.74E-01 | 6.55E-01 | -5.819 |
| ARHGAP26 |  | 0.05 | 8.025 | 0.888 | 3.75E-01 | 6.55E-01 | -5.82 |
| RPP30 |  | -0.032 | 6.81 | -0.889 | 3.75E-01 | 6.55E-01 | -5.82 |
| MMP12 |  | 0.105 | 5.836 | 0.887 | 3.75E-01 | 6.55E-01 | -5.82 |
| HEY2 |  | -0.047 | 5.908 | -0.888 | 3.75E-01 | 6.55E-01 | -5.82 |
| CNTN4 |  | 0.044 | 5.368 | 0.887 | 3.75E-01 | 6.55E-01 | -5.82 |
| AMFR |  | -0.056 | 9.757 | -0.888 | 3.75E-01 | 6.55E-01 | -5.821 |
| FTSJ1 |  | -0.036 | 8.317 | -0.888 | 3.75E-01 | 6.55E-01 | -5.821 |
| GAGE1 |  | -0.037 | 4.93 | -0.888 | 3.75E-01 | 6.55E-01 | -5.821 |
| TARS |  | 0.054 | 10.322 | 0.886 | 3.76E-01 | 6.56E-01 | -5.821 |
| SH3GL3 |  | -0.051 | 6.296 | -0.886 | 3.76E-01 | 6.56E-01 | -5.822 |
| IL7R |  | 0.07 | 7.974 | 0.885 | 3.76E-01 | 6.56E-01 | -5.822 |
| USP26 |  | 0.045 | 4.786 | 0.885 | 3.76E-01 | 6.56E-01 | -5.822 |
| GLI3 |  | -0.044 | 8.575 | -0.886 | 3.76E-01 | 6.56E-01 | -5.822 |
| BOK |  | 0.057 | 8.003 | 0.884 | 3.77E-01 | 6.56E-01 | -5.823 |
| MAP1LC3B |  | -0.04 | 11.01 | -0.885 | 3.77E-01 | 6.56E-01 | -5.823 |
| CYP2W1 |  | -0.066 | 8.379 | -0.885 | 3.77E-01 | 6.56E-01 | -5.823 |
| LAPTM4A |  | -0.044 | 12.319 | -0.885 | 3.77E-01 | 6.57E-01 | -5.823 |
| VPS4B |  | 0.037 | 10.344 | 0.883 | 3.77E-01 | 6.57E-01 | -5.823 |
| PKD2 |  | 0.043 | 8.653 | 0.882 | 3.78E-01 | 6.57E-01 | -5.824 |
| IFITM3 |  | -0.051 | 13.707 | -0.883 | 3.78E-01 | 6.57E-01 | -5.825 |
| KIAA0101 |  | 0.057 | 8.318 | 0.882 | 3.78E-01 | 6.57E-01 | -5.825 |
| RUFY1 |  | 0.036 | 9.809 | 0.882 | 3.78E-01 | 6.57E-01 | -5.825 |
| CNTN2 |  | -0.047 | 5.046 | -0.883 | 3.78E-01 | 6.57E-01 | -5.825 |
| BACH2 |  | 0.044 | 6.216 | 0.881 | 3.78E-01 | 6.58E-01 | -5.825 |
| TUBGCP3 |  | -0.035 | 7.455 | -0.882 | 3.78E-01 | 6.58E-01 | -5.826 |
| KCNH8 |  | 0.063 | 5.155 | 0.88 | 3.79E-01 | 6.58E-01 | -5.826 |
| CDCA8 |  | -0.06 | 8.372 | -0.881 | 3.79E-01 | 6.59E-01 | -5.827 |
| KCND3 |  | -0.039 | 5.783 | -0.88 | 3.79E-01 | 6.59E-01 | -5.827 |
| FAIM2 |  | 0.053 | 5.77 | 0.879 | 3.80E-01 | 6.59E-01 | -5.827 |
| NAGA |  | 0.036 | 8.52 | 0.879 | 3.80E-01 | 6.59E-01 | -5.828 |
| PSTPIP2 |  | -0.054 | 7.506 | -0.88 | 3.80E-01 | 6.59E-01 | -5.828 |
| CHRNA5 |  | 0.056 | 5.796 | 0.879 | 3.80E-01 | 6.59E-01 | -5.828 |
| PRND |  | 0.049 | 4.86 | 0.878 | 3.80E-01 | 6.59E-01 | -5.828 |
| SUMF1 |  | -0.034 | 9.758 | -0.879 | 3.80E-01 | 6.59E-01 | -5.828 |
| IL22RA2 |  | -0.063 | 5.767 | -0.879 | 3.80E-01 | 6.59E-01 | -5.828 |
| MAP3K14 |  | 0.049 | 7.65 | 0.877 | 3.80E-01 | 6.59E-01 | -5.829 |
| XYLB |  | -0.045 | 5.165 | -0.878 | 3.80E-01 | 6.59E-01 | -5.829 |
| DTNA |  | -0.038 | 6.201 | -0.878 | 3.81E-01 | 6.59E-01 | -5.829 |
| PDE3A |  | -0.041 | 5.649 | -0.878 | 3.81E-01 | 6.59E-01 | -5.829 |
| A4GNT |  | 0.046 | 4.989 | 0.876 | 3.81E-01 | 6.60E-01 | -5.83 |
| CYYR1 |  | 0.058 | 7.446 | 0.875 | 3.82E-01 | 6.60E-01 | -5.831 |
| OR51B2 |  | 0.048 | 4.838 | 0.875 | 3.82E-01 | 6.60E-01 | -5.831 |
| CAPN11 |  | 0.054 | 5.499 | 0.875 | 3.82E-01 | 6.60E-01 | -5.831 |
| SBNO1 |  | 0.037 | 8.203 | 0.875 | 3.82E-01 | 6.60E-01 | -5.831 |
| PDCL2 |  | -0.039 | 4.6 | -0.876 | 3.82E-01 | 6.60E-01 | -5.831 |
| RAPGEF6 |  | 0.033 | 7.862 | 0.875 | 3.82E-01 | 6.60E-01 | -5.831 |
| GRN |  | 0.052 | 13.02 | 0.874 | 3.82E-01 | 6.61E-01 | -5.831 |
| ANXA13 |  | -0.062 | 4.887 | -0.875 | 3.82E-01 | 6.61E-01 | -5.831 |
| EPHX2 |  | 0.054 | 8.394 | 0.874 | 3.82E-01 | 6.61E-01 | -5.832 |
| TIMM17A |  | -0.034 | 9.18 | -0.874 | 3.83E-01 | 6.62E-01 | -5.833 |
| NAV3 |  | -0.042 | 6.688 | -0.873 | 3.83E-01 | 6.62E-01 | -5.833 |
| ZNF311 |  | 0.046 | 5.333 | 0.872 | 3.83E-01 | 6.62E-01 | -5.834 |
| C6orf47 |  | 0.038 | 8.265 | 0.872 | 3.83E-01 | 6.62E-01 | -5.834 |
| RIOK2 |  | 0.045 | 8.014 | 0.871 | 3.84E-01 | 6.62E-01 | -5.834 |
| ADK |  | 0.037 | 8.998 | 0.871 | 3.84E-01 | 6.62E-01 | -5.834 |
| PTCD2 |  | 0.046 | 6.366 | 0.871 | 3.84E-01 | 6.62E-01 | -5.834 |
| RHOA |  | 0.035 | 11.975 | 0.871 | 3.84E-01 | 6.62E-01 | -5.834 |
| PKP1 |  | 0.061 | 5.686 | 0.87 | 3.84E-01 | 6.62E-01 | -5.834 |
| IFIT5 |  | -0.043 | 8.495 | -0.871 | 3.84E-01 | 6.62E-01 | -5.835 |
| ERAL1 |  | -0.036 | 9.327 | -0.871 | 3.84E-01 | 6.62E-01 | -5.835 |
| TOB2 |  | 0.043 | 7.883 | 0.87 | 3.84E-01 | 6.62E-01 | -5.835 |
| EPS8L3 |  | -0.064 | 6.065 | -0.87 | 3.85E-01 | 6.63E-01 | -5.835 |
| NXPH2 |  | -0.042 | 4.803 | -0.87 | 3.85E-01 | 6.63E-01 | -5.836 |
| FUT3 |  | 0.071 | 5.378 | 0.869 | 3.85E-01 | 6.63E-01 | -5.836 |
| NFATC1 |  | 0.039 | 6.837 | 0.868 | 3.85E-01 | 6.63E-01 | -5.836 |
| CNN1 |  | 0.088 | 8.301 | 0.868 | 3.85E-01 | 6.63E-01 | -5.836 |
| PKNOX1 |  | 0.046 | 7.706 | 0.868 | 3.85E-01 | 6.63E-01 | -5.837 |
| EME2 |  | 0.04 | 7.194 | 0.867 | 3.86E-01 | 6.63E-01 | -5.837 |
| IL18RAP |  | -0.061 | 6.951 | -0.868 | 3.86E-01 | 6.63E-01 | -5.837 |
| SLC2A11 |  | 0.06 | 7.622 | 0.867 | 3.86E-01 | 6.63E-01 | -5.837 |
| AMIGO2 |  | -0.059 | 7.633 | -0.868 | 3.86E-01 | 6.63E-01 | -5.837 |
| PRSS16 |  | -0.046 | 6.738 | -0.867 | 3.86E-01 | 6.64E-01 | -5.838 |
| ZBTB16 |  | -0.084 | 7.786 | -0.867 | 3.87E-01 | 6.64E-01 | -5.838 |
| WNK2 |  | 0.061 | 5.453 | 0.866 | 3.87E-01 | 6.64E-01 | -5.839 |
| C1D |  | 0.04 | 8.38 | 0.865 | 3.87E-01 | 6.64E-01 | -5.839 |
| NRM |  | -0.035 | 8.354 | -0.866 | 3.87E-01 | 6.64E-01 | -5.839 |
| BATF |  | -0.051 | 7.426 | -0.866 | 3.87E-01 | 6.64E-01 | -5.839 |
| KCNIP4 |  | -0.048 | 5.25 | -0.866 | 3.87E-01 | 6.64E-01 | -5.839 |
| BMF |  | -0.04 | 7.303 | -0.866 | 3.87E-01 | 6.64E-01 | -5.839 |
| CUL2 |  | -0.048 | 8.834 | -0.866 | 3.87E-01 | 6.64E-01 | -5.839 |
| CEBPB |  | 0.042 | 11.969 | 0.864 | 3.87E-01 | 6.64E-01 | -5.839 |
| FBXO15 |  | 0.051 | 5.871 | 0.864 | 3.87E-01 | 6.64E-01 | -5.839 |
| CACNA1S |  | -0.058 | 5.566 | -0.865 | 3.88E-01 | 6.64E-01 | -5.84 |
| GPR21 |  | -0.05 | 5.136 | -0.865 | 3.88E-01 | 6.64E-01 | -5.84 |
| RIBC1 |  | 0.034 | 5.14 | 0.863 | 3.88E-01 | 6.64E-01 | -5.84 |
| MAML2 |  | 0.047 | 6.855 | 0.863 | 3.88E-01 | 6.64E-01 | -5.84 |
| DDOST |  | 0.032 | 11.868 | 0.862 | 3.88E-01 | 6.65E-01 | -5.841 |
| SLC6A2 |  | -0.057 | 7.608 | -0.863 | 3.89E-01 | 6.65E-01 | -5.841 |
| GBGT1 |  | 0.051 | 7.554 | 0.862 | 3.89E-01 | 6.66E-01 | -5.842 |
| RDH10 |  | -0.052 | 7.851 | -0.862 | 3.89E-01 | 6.66E-01 | -5.842 |
| PEX7 |  | 0.031 | 7.977 | 0.861 | 3.89E-01 | 6.66E-01 | -5.842 |
| FGD6 |  | 0.05 | 7.199 | 0.861 | 3.89E-01 | 6.66E-01 | -5.842 |
| RAB37 |  | -0.043 | 7.455 | -0.861 | 3.90E-01 | 6.66E-01 | -5.843 |
| CDX2 |  | -0.055 | 4.71 | -0.86 | 3.90E-01 | 6.67E-01 | -5.844 |
| COL19A1 |  | 0.054 | 5.23 | 0.859 | 3.90E-01 | 6.67E-01 | -5.844 |
| NEU2 |  | -0.053 | 4.978 | -0.86 | 3.91E-01 | 6.67E-01 | -5.844 |
| CXorf38 |  | 0.036 | 7.806 | 0.859 | 3.91E-01 | 6.67E-01 | -5.844 |
| CD34 |  | -0.045 | 7.629 | -0.859 | 3.91E-01 | 6.67E-01 | -5.844 |
| GPR173 |  | -0.045 | 5.943 | -0.859 | 3.91E-01 | 6.67E-01 | -5.845 |
| NSDHL |  | 0.035 | 8.901 | 0.858 | 3.91E-01 | 6.67E-01 | -5.845 |
| EXTL2 |  | 0.046 | 8.28 | 0.858 | 3.91E-01 | 6.67E-01 | -5.845 |
| RS1 |  | -0.059 | 4.873 | -0.859 | 3.91E-01 | 6.67E-01 | -5.845 |
| ANP32E |  | 0.038 | 7.526 | 0.858 | 3.91E-01 | 6.67E-01 | -5.845 |
| LOC81691 |  | -0.058 | 6.11 | -0.858 | 3.91E-01 | 6.67E-01 | -5.845 |
| SHC1 |  | -0.032 | 9.593 | -0.858 | 3.91E-01 | 6.67E-01 | -5.845 |
| FZR1 |  | 0.036 | 7.596 | 0.857 | 3.91E-01 | 6.67E-01 | -5.846 |
| EIF5B |  | -0.04 | 8.398 | -0.858 | 3.92E-01 | 6.67E-01 | -5.846 |
| BST1 |  | -0.039 | 8.075 | -0.857 | 3.92E-01 | 6.68E-01 | -5.846 |
| GNB2 |  | 0.041 | 9.97 | 0.856 | 3.92E-01 | 6.68E-01 | -5.847 |
| GJB1 |  | -0.056 | 5.126 | -0.857 | 3.92E-01 | 6.68E-01 | -5.847 |
| CDC27 |  | 0.032 | 7.792 | 0.855 | 3.92E-01 | 6.68E-01 | -5.847 |
| BAG4 |  | 0.039 | 7.162 | 0.855 | 3.93E-01 | 6.68E-01 | -5.847 |
| S100G |  | 0.047 | 4.85 | 0.855 | 3.93E-01 | 6.68E-01 | -5.847 |
| TIE1 |  | -0.059 | 7.651 | -0.855 | 3.93E-01 | 6.69E-01 | -5.848 |
| NAALAD2 |  | 0.047 | 6.313 | 0.854 | 3.93E-01 | 6.69E-01 | -5.848 |
| PDE8B |  | 0.059 | 8.143 | 0.853 | 3.93E-01 | 6.69E-01 | -5.849 |
| ZAN |  | -0.048 | 5.784 | -0.854 | 3.94E-01 | 6.69E-01 | -5.849 |
| GPR153 |  | -0.041 | 8.121 | -0.854 | 3.94E-01 | 6.69E-01 | -5.849 |
| APOL6 |  | -0.056 | 6.682 | -0.853 | 3.94E-01 | 6.69E-01 | -5.849 |
| RFT1 |  | -0.03 | 6.754 | -0.853 | 3.94E-01 | 6.69E-01 | -5.85 |
| KCNQ1 |  | -0.033 | 7.354 | -0.853 | 3.94E-01 | 6.69E-01 | -5.85 |
| EPHA7 |  | 0.033 | 4.769 | 0.852 | 3.94E-01 | 6.69E-01 | -5.85 |
| PSG2 |  | 0.091 | 11.768 | 0.852 | 3.94E-01 | 6.69E-01 | -5.85 |
| RRAGA |  | 0.026 | 11.918 | 0.852 | 3.94E-01 | 6.69E-01 | -5.85 |
| LDHAL6B |  | 0.044 | 4.703 | 0.852 | 3.94E-01 | 6.69E-01 | -5.85 |
| SLC12A3 |  | -0.051 | 5.451 | -0.852 | 3.95E-01 | 6.69E-01 | -5.85 |
| EBF2 |  | 0.05 | 4.863 | 0.851 | 3.95E-01 | 6.69E-01 | -5.85 |
| ZNF384 |  | 0.028 | 9.21 | 0.851 | 3.95E-01 | 6.69E-01 | -5.851 |
| MGC50722 |  | 0.055 | 5.011 | 0.851 | 3.95E-01 | 6.69E-01 | -5.851 |
| CYB5R2 |  | 0.055 | 7.925 | 0.85 | 3.95E-01 | 6.69E-01 | -5.851 |
| ATP6V0C |  | -0.033 | 12.203 | -0.851 | 3.95E-01 | 6.69E-01 | -5.851 |
| ATIC |  | -0.036 | 10.617 | -0.851 | 3.95E-01 | 6.69E-01 | -5.851 |
| ELP3 |  | -0.031 | 8.651 | -0.851 | 3.96E-01 | 6.69E-01 | -5.852 |
| HARS2 |  | -0.034 | 8.799 | -0.851 | 3.96E-01 | 6.69E-01 | -5.852 |
| FRMD3 |  | -0.067 | 6.609 | -0.85 | 3.96E-01 | 6.70E-01 | -5.852 |
| VN1R5 |  | 0.05 | 4.944 | 0.849 | 3.96E-01 | 6.70E-01 | -5.852 |
| TRIP12 |  | -0.05 | 9.572 | -0.85 | 3.96E-01 | 6.70E-01 | -5.852 |
| LZTS2 |  | 0.036 | 9.067 | 0.849 | 3.96E-01 | 6.70E-01 | -5.852 |
| UPK1A |  | 0.045 | 5.192 | 0.848 | 3.96E-01 | 6.70E-01 | -5.853 |
| ATP5L |  | 0.035 | 12.046 | 0.848 | 3.97E-01 | 6.70E-01 | -5.853 |
| MAP3K8 |  | 0.052 | 8.873 | 0.848 | 3.97E-01 | 6.70E-01 | -5.853 |
| GPAM |  | 0.042 | 6.44 | 0.847 | 3.97E-01 | 6.70E-01 | -5.854 |
| PDCD1LG2 |  | -0.064 | 5.774 | -0.848 | 3.97E-01 | 6.70E-01 | -5.854 |
| BAG5 |  | -0.027 | 8.316 | -0.848 | 3.97E-01 | 6.70E-01 | -5.854 |
| VHL |  | 0.036 | 9.796 | 0.846 | 3.97E-01 | 6.71E-01 | -5.854 |
| ATP5B |  | -0.026 | 12.632 | -0.847 | 3.97E-01 | 6.71E-01 | -5.854 |
| C1QTNF7 |  | 0.052 | 5.895 | 0.846 | 3.98E-01 | 6.71E-01 | -5.855 |
| SCPEP1 |  | 0.043 | 8.738 | 0.846 | 3.98E-01 | 6.71E-01 | -5.855 |
| GRK6 |  | -0.035 | 8.253 | -0.845 | 3.99E-01 | 6.72E-01 | -5.856 |
| PPIL4 |  | -0.031 | 7.484 | -0.845 | 3.99E-01 | 6.72E-01 | -5.856 |
| KCNJ14 |  | 0.041 | 6.589 | 0.844 | 3.99E-01 | 6.72E-01 | -5.856 |
| GPR3 |  | 0.043 | 5.972 | 0.843 | 3.99E-01 | 6.72E-01 | -5.857 |
| POSTN |  | -0.064 | 8.095 | -0.844 | 3.99E-01 | 6.72E-01 | -5.857 |
| DHX36 |  | -0.033 | 9.628 | -0.843 | 4.00E-01 | 6.73E-01 | -5.858 |
| WDR4 |  | 0.05 | 7.296 | 0.842 | 4.00E-01 | 6.73E-01 | -5.858 |
| CREB3L3 |  | -0.046 | 5.674 | -0.842 | 4.00E-01 | 6.74E-01 | -5.858 |
| WEE1 |  | 0.056 | 8.465 | 0.841 | 4.00E-01 | 6.74E-01 | -5.858 |
| AK3 |  | 0.038 | 9.305 | 0.841 | 4.00E-01 | 6.74E-01 | -5.859 |
| NME1 |  | -0.047 | 10.203 | -0.842 | 4.01E-01 | 6.74E-01 | -5.859 |
| GRIN1 |  | -0.047 | 6.801 | -0.841 | 4.01E-01 | 6.74E-01 | -5.859 |
| MPI |  | -0.038 | 8.129 | -0.841 | 4.01E-01 | 6.74E-01 | -5.86 |
| PRM2 |  | 0.051 | 5.257 | 0.839 | 4.01E-01 | 6.74E-01 | -5.86 |
| PDCD10 |  | -0.046 | 9.709 | -0.84 | 4.01E-01 | 6.74E-01 | -5.86 |
| MRPL11 |  | 0.034 | 8.737 | 0.839 | 4.01E-01 | 6.74E-01 | -5.86 |
| PSMD3 |  | -0.034 | 9.795 | -0.839 | 4.02E-01 | 6.75E-01 | -5.861 |
| PPP1R3D |  | -0.037 | 8.502 | -0.839 | 4.02E-01 | 6.75E-01 | -5.861 |
| ASB11 |  | 0.05 | 4.81 | 0.838 | 4.02E-01 | 6.75E-01 | -5.861 |
| FGF17 |  | 0.041 | 5.192 | 0.838 | 4.02E-01 | 6.75E-01 | -5.861 |
| GAL3ST4 |  | -0.049 | 6.979 | -0.838 | 4.03E-01 | 6.75E-01 | -5.862 |
| C10orf32 |  | -0.038 | 9.338 | -0.837 | 4.03E-01 | 6.76E-01 | -5.862 |
| CLEC4F |  | -0.054 | 5.094 | -0.837 | 4.03E-01 | 6.76E-01 | -5.863 |
| MBD6 |  | -0.052 | 10.061 | -0.837 | 4.03E-01 | 6.76E-01 | -5.863 |
| TLX3 |  | -0.05 | 5.244 | -0.837 | 4.03E-01 | 6.76E-01 | -5.863 |
| TRAM1L1 |  | 0.05 | 5.265 | 0.836 | 4.03E-01 | 6.76E-01 | -5.863 |
| PLEKHF2 |  | -0.039 | 9.305 | -0.835 | 4.04E-01 | 6.77E-01 | -5.864 |
| MGLL |  | 0.042 | 9.872 | 0.834 | 4.04E-01 | 6.77E-01 | -5.864 |
| C6orf106 |  | -0.036 | 8.814 | -0.835 | 4.04E-01 | 6.77E-01 | -5.864 |
| PDE6G |  | 0.05 | 6.818 | 0.834 | 4.04E-01 | 6.77E-01 | -5.864 |
| SLC17A6 |  | -0.037 | 4.62 | -0.834 | 4.05E-01 | 6.77E-01 | -5.865 |
| ALX3 |  | -0.051 | 5.555 | -0.834 | 4.05E-01 | 6.77E-01 | -5.865 |
| AIM2 |  | 0.054 | 6.866 | 0.833 | 4.05E-01 | 6.77E-01 | -5.865 |
| PSMA7 |  | -0.03 | 10.166 | -0.834 | 4.05E-01 | 6.77E-01 | -5.865 |
| RHPN2 |  | 0.055 | 7.453 | 0.832 | 4.06E-01 | 6.78E-01 | -5.866 |
| HIST1H1C |  | 0.069 | 11.578 | 0.831 | 4.06E-01 | 6.79E-01 | -5.867 |
| PSMD7 |  | -0.027 | 11.514 | -0.831 | 4.06E-01 | 6.79E-01 | -5.867 |
| CUL1 |  | -0.029 | 9.74 | -0.831 | 4.06E-01 | 6.79E-01 | -5.867 |
| GPR107 |  | -0.037 | 8.145 | -0.831 | 4.06E-01 | 6.79E-01 | -5.867 |
| NGB |  | -0.048 | 5.585 | -0.831 | 4.07E-01 | 6.79E-01 | -5.868 |
| CRMP1 |  | -0.048 | 6.377 | -0.831 | 4.07E-01 | 6.79E-01 | -5.868 |
| TRIM15 |  | 0.036 | 5.745 | 0.83 | 4.07E-01 | 6.79E-01 | -5.868 |
| ATP8A1 |  | 0.059 | 6.128 | 0.83 | 4.07E-01 | 6.79E-01 | -5.868 |
| TRIM56 |  | -0.04 | 9.382 | -0.83 | 4.07E-01 | 6.79E-01 | -5.868 |
| ZDHHC14 |  | 0.041 | 8.081 | 0.829 | 4.07E-01 | 6.79E-01 | -5.868 |
| PRKRIR |  | -0.033 | 9.821 | -0.83 | 4.07E-01 | 6.79E-01 | -5.868 |
| POLR3F |  | -0.036 | 7.641 | -0.83 | 4.07E-01 | 6.79E-01 | -5.869 |
| OSTM1 |  | 0.037 | 8.509 | 0.828 | 4.08E-01 | 6.79E-01 | -5.869 |
| MAGI1 |  | 0.034 | 5.982 | 0.828 | 4.08E-01 | 6.80E-01 | -5.869 |
| NDUFA7 |  | 0.032 | 10.784 | 0.827 | 4.08E-01 | 6.80E-01 | -5.87 |
| MYBPC2 |  | -0.063 | 5.512 | -0.827 | 4.09E-01 | 6.81E-01 | -5.87 |
| SLC29A4 |  | 0.045 | 6.248 | 0.826 | 4.09E-01 | 6.81E-01 | -5.871 |
| GRIK5 |  | -0.045 | 6.239 | -0.827 | 4.09E-01 | 6.81E-01 | -5.871 |
| TMEM39A |  | 0.03 | 8.625 | 0.826 | 4.09E-01 | 6.81E-01 | -5.871 |
| KLHL12 |  | -0.032 | 8.023 | -0.827 | 4.09E-01 | 6.81E-01 | -5.871 |
| CACNA1D |  | -0.053 | 5.123 | -0.826 | 4.09E-01 | 6.81E-01 | -5.872 |
| INS |  | -0.047 | 5.994 | -0.826 | 4.10E-01 | 6.81E-01 | -5.872 |
| PTCHD1 |  | -0.047 | 4.785 | -0.825 | 4.10E-01 | 6.81E-01 | -5.872 |
| SOX11 |  | -0.04 | 4.86 | -0.825 | 4.10E-01 | 6.81E-01 | -5.872 |
| CAPNS1 |  | 0.041 | 10.932 | 0.824 | 4.10E-01 | 6.81E-01 | -5.872 |
| MED31 |  | 0.046 | 6.404 | 0.824 | 4.10E-01 | 6.81E-01 | -5.872 |
| ALPI |  | -0.056 | 5.545 | -0.825 | 4.10E-01 | 6.81E-01 | -5.872 |
| SAP30 |  | 0.043 | 8.928 | 0.823 | 4.11E-01 | 6.82E-01 | -5.873 |
| KLF9 |  | 0.066 | 9.195 | 0.823 | 4.11E-01 | 6.82E-01 | -5.873 |
| RPA4 |  | -0.062 | 7.318 | -0.824 | 4.11E-01 | 6.82E-01 | -5.873 |
| DISP1 |  | -0.04 | 7.092 | -0.823 | 4.11E-01 | 6.82E-01 | -5.873 |
| DUSP13 |  | 0.049 | 5.876 | 0.822 | 4.11E-01 | 6.82E-01 | -5.874 |
| FADS2 |  | 0.047 | 8.406 | 0.822 | 4.11E-01 | 6.82E-01 | -5.874 |
| PRKD2 |  | -0.038 | 9.082 | -0.823 | 4.11E-01 | 6.82E-01 | -5.874 |
| FBXO21 |  | -0.027 | 10.127 | -0.822 | 4.12E-01 | 6.82E-01 | -5.875 |
| DEF6 |  | 0.039 | 7.85 | 0.821 | 4.12E-01 | 6.82E-01 | -5.875 |
| RTP1 |  | -0.043 | 5.021 | -0.822 | 4.12E-01 | 6.83E-01 | -5.875 |
| NCK1 |  | -0.049 | 8.921 | -0.821 | 4.12E-01 | 6.83E-01 | -5.875 |
| E2F2 |  | 0.052 | 8.397 | 0.82 | 4.12E-01 | 6.83E-01 | -5.875 |
| COMMD1 |  | 0.037 | 10.125 | 0.82 | 4.12E-01 | 6.83E-01 | -5.875 |
| ABCG8 |  | 0.039 | 4.89 | 0.82 | 4.12E-01 | 6.83E-01 | -5.875 |
| PTER |  | -0.05 | 6.981 | -0.82 | 4.13E-01 | 6.83E-01 | -5.876 |
| RHOT1 |  | -0.036 | 7.418 | -0.82 | 4.13E-01 | 6.83E-01 | -5.876 |
| GALNT9 |  | -0.045 | 5.429 | -0.82 | 4.13E-01 | 6.83E-01 | -5.876 |
| TYRO3 |  | -0.042 | 7.892 | -0.82 | 4.13E-01 | 6.83E-01 | -5.877 |
| C15orf26 |  | -0.058 | 5.481 | -0.819 | 4.13E-01 | 6.84E-01 | -5.877 |
| CRLF2 |  | 0.046 | 5.331 | 0.818 | 4.14E-01 | 6.84E-01 | -5.877 |
| CDC25B |  | -0.04 | 9.298 | -0.818 | 4.14E-01 | 6.84E-01 | -5.877 |
| OSBPL2 |  | -0.029 | 9.093 | -0.818 | 4.14E-01 | 6.84E-01 | -5.878 |
| FRMPD1 |  | 0.06 | 5.244 | 0.816 | 4.15E-01 | 6.85E-01 | -5.879 |
| CPNE5 |  | 0.051 | 7.21 | 0.815 | 4.15E-01 | 6.85E-01 | -5.879 |
| MAPK13 |  | -0.047 | 8.452 | -0.816 | 4.15E-01 | 6.85E-01 | -5.879 |
| GIT2 |  | -0.028 | 8.26 | -0.816 | 4.15E-01 | 6.86E-01 | -5.88 |
| WNT16 |  | -0.054 | 4.941 | -0.815 | 4.15E-01 | 6.86E-01 | -5.88 |
| RBBP9 |  | 0.03 | 6.369 | 0.814 | 4.16E-01 | 6.86E-01 | -5.88 |
| VTI1A |  | 0.032 | 7.268 | 0.814 | 4.16E-01 | 6.86E-01 | -5.88 |
| RPE |  | 0.032 | 7.663 | 0.814 | 4.16E-01 | 6.86E-01 | -5.88 |
| EDA2R |  | 0.06 | 5.391 | 0.813 | 4.16E-01 | 6.86E-01 | -5.881 |
| NOVA2 |  | -0.072 | 7.782 | -0.814 | 4.16E-01 | 6.86E-01 | -5.881 |
| IFIH1 |  | 0.053 | 9.409 | 0.812 | 4.16E-01 | 6.86E-01 | -5.881 |
| COL7A1 |  | -0.055 | 8.184 | -0.813 | 4.17E-01 | 6.86E-01 | -5.881 |
| SYCP1 |  | -0.047 | 5.389 | -0.813 | 4.17E-01 | 6.86E-01 | -5.882 |
| ZNF71 |  | 0.035 | 6.965 | 0.812 | 4.17E-01 | 6.86E-01 | -5.882 |
| FGFR1OP2 |  | 0.035 | 8.166 | 0.812 | 4.17E-01 | 6.86E-01 | -5.882 |
| SMARCA5 |  | 0.031 | 8.77 | 0.812 | 4.17E-01 | 6.86E-01 | -5.882 |
| GZMM |  | -0.044 | 7.453 | -0.813 | 4.17E-01 | 6.86E-01 | -5.882 |
| HIPK2 |  | 0.04 | 7.788 | 0.812 | 4.17E-01 | 6.86E-01 | -5.882 |
| EHD1 |  | 0.039 | 11.115 | 0.811 | 4.17E-01 | 6.86E-01 | -5.882 |
| MMP10 |  | 0.074 | 5.653 | 0.811 | 4.17E-01 | 6.86E-01 | -5.882 |
| CREBL2 |  | -0.031 | 8.176 | -0.812 | 4.17E-01 | 6.86E-01 | -5.882 |
| PODXL2 |  | 0.04 | 5.794 | 0.811 | 4.17E-01 | 6.86E-01 | -5.883 |
| SDF2 |  | 0.031 | 9.999 | 0.811 | 4.18E-01 | 6.87E-01 | -5.883 |
| HIST1H1D |  | 0.064 | 7.397 | 0.81 | 4.18E-01 | 6.87E-01 | -5.883 |
| CYP8B1 |  | -0.045 | 5.755 | -0.81 | 4.19E-01 | 6.87E-01 | -5.884 |
| GMPPA |  | 0.036 | 8.064 | 0.809 | 4.19E-01 | 6.87E-01 | -5.884 |
| KCTD16 |  | 0.037 | 4.751 | 0.809 | 4.19E-01 | 6.87E-01 | -5.884 |
| AIP |  | 0.026 | 9.939 | 0.809 | 4.19E-01 | 6.87E-01 | -5.884 |
| SEMA4F |  | 0.045 | 6.428 | 0.808 | 4.19E-01 | 6.88E-01 | -5.885 |
| BTN3A2 |  | 0.076 | 8.807 | 0.808 | 4.19E-01 | 6.88E-01 | -5.885 |
| DOCK7 |  | -0.037 | 7.642 | -0.808 | 4.20E-01 | 6.88E-01 | -5.886 |
| ASRGL1 |  | -0.047 | 7.39 | -0.808 | 4.20E-01 | 6.88E-01 | -5.886 |
| PABPC5 |  | 0.045 | 5.26 | 0.807 | 4.20E-01 | 6.88E-01 | -5.886 |
| CD72 |  | 0.054 | 6.638 | 0.807 | 4.20E-01 | 6.88E-01 | -5.886 |
| DLL1 |  | -0.058 | 7.251 | -0.807 | 4.20E-01 | 6.89E-01 | -5.886 |
| WDR36 |  | 0.031 | 8.15 | 0.805 | 4.21E-01 | 6.90E-01 | -5.887 |
| ZNF511 |  | -0.038 | 8.677 | -0.805 | 4.21E-01 | 6.90E-01 | -5.888 |
| APOD |  | -0.08 | 8.166 | -0.805 | 4.21E-01 | 6.90E-01 | -5.888 |
| GPR32 |  | 0.056 | 5.922 | 0.804 | 4.21E-01 | 6.90E-01 | -5.888 |
| RIC3 |  | 0.049 | 5.334 | 0.803 | 4.22E-01 | 6.90E-01 | -5.888 |
| ECE1 |  | 0.053 | 7.965 | 0.803 | 4.22E-01 | 6.90E-01 | -5.888 |
| ADRA1B |  | 0.058 | 5.55 | 0.803 | 4.22E-01 | 6.90E-01 | -5.889 |
| PGBD5 |  | -0.043 | 5.592 | -0.804 | 4.22E-01 | 6.90E-01 | -5.889 |
| VSNL1 |  | -0.06 | 6.134 | -0.804 | 4.22E-01 | 6.90E-01 | -5.889 |
| PITPNM2 |  | 0.037 | 6.135 | 0.802 | 4.22E-01 | 6.91E-01 | -5.889 |
| SEC14L3 |  | -0.04 | 4.8 | -0.803 | 4.22E-01 | 6.91E-01 | -5.889 |
| KRT1 |  | 0.085 | 7.911 | 0.802 | 4.23E-01 | 6.91E-01 | -5.89 |
| IL3 |  | 0.051 | 5.055 | 0.801 | 4.23E-01 | 6.91E-01 | -5.89 |
| CRLF1 |  | 0.079 | 7.42 | 0.801 | 4.23E-01 | 6.91E-01 | -5.89 |
| TBC1D17 |  | 0.043 | 8.255 | 0.801 | 4.23E-01 | 6.91E-01 | -5.89 |
| ESM1 |  | 0.051 | 5.354 | 0.801 | 4.23E-01 | 6.91E-01 | -5.89 |
| MYL4 |  | -0.057 | 7.168 | -0.802 | 4.23E-01 | 6.91E-01 | -5.89 |
| CDH8 |  | -0.034 | 4.759 | -0.802 | 4.23E-01 | 6.91E-01 | -5.89 |
| ENTPD2 |  | 0.048 | 6.542 | 0.801 | 4.23E-01 | 6.91E-01 | -5.891 |
| LDHD |  | 0.054 | 6.756 | 0.8 | 4.23E-01 | 6.91E-01 | -5.891 |
| LRRC28 |  | 0.032 | 7.139 | 0.8 | 4.24E-01 | 6.91E-01 | -5.891 |
| LRP8 |  | -0.066 | 8.053 | -0.801 | 4.24E-01 | 6.91E-01 | -5.891 |
| NCL |  | 0.033 | 11.156 | 0.8 | 4.24E-01 | 6.91E-01 | -5.891 |
| CHEK2 |  | -0.039 | 6.968 | -0.8 | 4.24E-01 | 6.91E-01 | -5.892 |
| SENP3 |  | 0.029 | 8.039 | 0.799 | 4.24E-01 | 6.91E-01 | -5.892 |
| GALNT5 |  | 0.055 | 5.131 | 0.799 | 4.24E-01 | 6.91E-01 | -5.892 |
| GPR146 |  | -0.058 | 7.149 | -0.8 | 4.24E-01 | 6.91E-01 | -5.892 |
| GAD1 |  | -0.046 | 5.425 | -0.799 | 4.25E-01 | 6.92E-01 | -5.892 |
| CDCA2 |  | 0.056 | 6.691 | 0.798 | 4.25E-01 | 6.92E-01 | -5.893 |
| ADAM19 |  | -0.053 | 8.632 | -0.798 | 4.25E-01 | 6.92E-01 | -5.893 |
| JAK3 |  | -0.038 | 7.081 | -0.797 | 4.26E-01 | 6.93E-01 | -5.894 |
| ELF3 |  | 0.057 | 7.984 | 0.795 | 4.26E-01 | 6.94E-01 | -5.895 |
| ZNF660 |  | -0.06 | 5.465 | -0.796 | 4.26E-01 | 6.94E-01 | -5.895 |
| IQGAP3 |  | -0.051 | 7.499 | -0.796 | 4.26E-01 | 6.94E-01 | -5.895 |
| TMED6 |  | -0.058 | 5.549 | -0.796 | 4.27E-01 | 6.94E-01 | -5.895 |
| TCOF1 |  | -0.03 | 7.928 | -0.796 | 4.27E-01 | 6.94E-01 | -5.895 |
| DHX57 |  | 0.033 | 6.473 | 0.794 | 4.27E-01 | 6.94E-01 | -5.896 |
| IPO4 |  | 0.035 | 8.113 | 0.794 | 4.27E-01 | 6.94E-01 | -5.896 |
| TRIM2 |  | -0.045 | 7.058 | -0.794 | 4.28E-01 | 6.95E-01 | -5.897 |
| ADAMTS17 |  | 0.041 | 5.278 | 0.793 | 4.28E-01 | 6.95E-01 | -5.897 |
| NUDT15 |  | 0.045 | 7.73 | 0.792 | 4.28E-01 | 6.95E-01 | -5.897 |
| INSM2 |  | -0.038 | 4.621 | -0.792 | 4.29E-01 | 6.96E-01 | -5.898 |
| TRIM3 |  | -0.039 | 6.147 | -0.792 | 4.29E-01 | 6.96E-01 | -5.898 |
| ADRB1 |  | -0.039 | 7.666 | -0.792 | 4.29E-01 | 6.96E-01 | -5.898 |
| FBXO18 |  | 0.027 | 8.564 | 0.791 | 4.29E-01 | 6.96E-01 | -5.898 |
| DFNB31 |  | 0.041 | 5.646 | 0.79 | 4.29E-01 | 6.96E-01 | -5.898 |
| CDSN |  | -0.069 | 6.131 | -0.791 | 4.30E-01 | 6.97E-01 | -5.899 |
| MACF1 |  | 0.044 | 8.05 | 0.789 | 4.30E-01 | 6.97E-01 | -5.899 |
| DHFR |  | -0.043 | 7.777 | -0.79 | 4.30E-01 | 6.97E-01 | -5.899 |
| ETV5 |  | -0.052 | 8.245 | -0.79 | 4.30E-01 | 6.97E-01 | -5.9 |
| COL25A1 |  | 0.033 | 4.749 | 0.788 | 4.30E-01 | 6.97E-01 | -5.9 |
| DNAJC10 |  | -0.027 | 8.685 | -0.788 | 4.31E-01 | 6.98E-01 | -5.901 |
| DCTN4 |  | 0.042 | 9.743 | 0.786 | 4.32E-01 | 6.99E-01 | -5.901 |
| TNIP3 |  | 0.049 | 4.867 | 0.786 | 4.32E-01 | 6.99E-01 | -5.901 |
| SOX7 |  | -0.048 | 7.814 | -0.787 | 4.32E-01 | 6.99E-01 | -5.902 |
| PTK2 |  | -0.029 | 9.33 | -0.787 | 4.32E-01 | 6.99E-01 | -5.902 |
| TCF7L2 |  | 0.049 | 7.18 | 0.785 | 4.32E-01 | 6.99E-01 | -5.902 |
| EXOSC5 |  | 0.034 | 7.616 | 0.785 | 4.32E-01 | 6.99E-01 | -5.902 |
| ITPR2 |  | 0.032 | 7.66 | 0.785 | 4.33E-01 | 6.99E-01 | -5.903 |
| CHN1 |  | -0.049 | 8.748 | -0.786 | 4.33E-01 | 6.99E-01 | -5.903 |
| BLOC1S2 |  | -0.033 | 8.737 | -0.785 | 4.33E-01 | 6.99E-01 | -5.903 |
| TARBP2 |  | 0.029 | 8.98 | 0.784 | 4.33E-01 | 6.99E-01 | -5.903 |
| ODF1 |  | 0.034 | 4.767 | 0.784 | 4.33E-01 | 7.00E-01 | -5.904 |
| TFF2 |  | -0.043 | 5.526 | -0.784 | 4.33E-01 | 7.00E-01 | -5.904 |
| UMPS |  | -0.032 | 8.793 | -0.784 | 4.34E-01 | 7.00E-01 | -5.904 |
| HIST1H4H |  | -0.055 | 8.283 | -0.783 | 4.34E-01 | 7.00E-01 | -5.904 |
| BMP3 |  | 0.032 | 4.762 | 0.782 | 4.34E-01 | 7.00E-01 | -5.904 |
| G6PC3 |  | -0.058 | 8.526 | -0.783 | 4.34E-01 | 7.00E-01 | -5.905 |
| SLC35A5 |  | 0.042 | 9.199 | 0.782 | 4.34E-01 | 7.00E-01 | -5.905 |
| PSMA5 |  | 0.03 | 11.301 | 0.782 | 4.34E-01 | 7.00E-01 | -5.905 |
| MYADM |  | -0.032 | 9.469 | -0.782 | 4.35E-01 | 7.01E-01 | -5.906 |
| HEY1 |  | 0.058 | 8.744 | 0.78 | 4.35E-01 | 7.02E-01 | -5.906 |
| TP53INP2 |  | -0.045 | 7.197 | -0.781 | 4.36E-01 | 7.02E-01 | -5.907 |
| CLCN7 |  | 0.044 | 9.058 | 0.78 | 4.36E-01 | 7.02E-01 | -5.907 |
| FGB |  | 0.06 | 4.998 | 0.779 | 4.36E-01 | 7.02E-01 | -5.907 |
| LYZL4 |  | 0.045 | 4.851 | 0.779 | 4.36E-01 | 7.02E-01 | -5.907 |
| NOSTRIN |  | 0.044 | 7.655 | 0.779 | 4.36E-01 | 7.02E-01 | -5.907 |
| VBP1 |  | 0.042 | 9.34 | 0.778 | 4.36E-01 | 7.02E-01 | -5.907 |
| STRN |  | 0.038 | 6.716 | 0.778 | 4.36E-01 | 7.02E-01 | -5.908 |
| FSCN2 |  | 0.058 | 5.532 | 0.778 | 4.37E-01 | 7.02E-01 | -5.908 |
| MKLN1 |  | 0.03 | 9.186 | 0.778 | 4.37E-01 | 7.02E-01 | -5.908 |
| PTP4A3 |  | -0.039 | 7.533 | -0.779 | 4.37E-01 | 7.02E-01 | -5.908 |
| MAML1 |  | -0.027 | 9.092 | -0.778 | 4.37E-01 | 7.02E-01 | -5.908 |
| KIF20A |  | 0.061 | 7.721 | 0.777 | 4.37E-01 | 7.02E-01 | -5.908 |
| SCGB2A1 |  | 0.063 | 4.742 | 0.776 | 4.38E-01 | 7.03E-01 | -5.909 |
| TMEM26 |  | 0.041 | 5.231 | 0.776 | 4.38E-01 | 7.03E-01 | -5.909 |
| ZNF425 |  | 0.037 | 6.665 | 0.775 | 4.38E-01 | 7.04E-01 | -5.91 |
| SMPD1 |  | 0.038 | 8.532 | 0.775 | 4.38E-01 | 7.04E-01 | -5.91 |
| LACTB2 |  | -0.04 | 8.09 | -0.776 | 4.38E-01 | 7.04E-01 | -5.91 |
| ATAD2 |  | -0.033 | 7.812 | -0.776 | 4.38E-01 | 7.04E-01 | -5.91 |
| SSSCA1 |  | 0.03 | 9.281 | 0.774 | 4.39E-01 | 7.04E-01 | -5.911 |
| LAMA4 |  | -0.046 | 8.013 | -0.774 | 4.39E-01 | 7.04E-01 | -5.911 |
| ZNF496 |  | 0.039 | 6.442 | 0.773 | 4.39E-01 | 7.04E-01 | -5.911 |
| GGA3 |  | -0.022 | 8.937 | -0.774 | 4.39E-01 | 7.04E-01 | -5.911 |
| ZNF354B |  | -0.049 | 7.239 | -0.774 | 4.39E-01 | 7.04E-01 | -5.911 |
| POLR1C |  | -0.028 | 8.787 | -0.774 | 4.40E-01 | 7.04E-01 | -5.912 |
| THOP1 |  | 0.051 | 10.418 | 0.773 | 4.40E-01 | 7.04E-01 | -5.912 |
| MAPK8IP1 |  | -0.072 | 6.294 | -0.774 | 4.40E-01 | 7.04E-01 | -5.912 |
| LTK |  | -0.044 | 7.398 | -0.774 | 4.40E-01 | 7.04E-01 | -5.912 |
| PLA1A |  | -0.045 | 6.444 | -0.773 | 4.40E-01 | 7.05E-01 | -5.912 |
| FNBP1 |  | -0.032 | 9.559 | -0.773 | 4.40E-01 | 7.05E-01 | -5.912 |
| CLEC3A |  | 0.044 | 4.75 | 0.771 | 4.40E-01 | 7.05E-01 | -5.913 |
| TAS2R3 |  | -0.046 | 4.86 | -0.772 | 4.41E-01 | 7.05E-01 | -5.913 |
| DMBT1 |  | 0.035 | 4.823 | 0.77 | 4.41E-01 | 7.06E-01 | -5.913 |
| TBC1D19 |  | -0.042 | 6.738 | -0.771 | 4.41E-01 | 7.06E-01 | -5.914 |
| REN |  | -0.066 | 6.263 | -0.771 | 4.41E-01 | 7.06E-01 | -5.914 |
| SLC41A1 |  | -0.037 | 7.07 | -0.77 | 4.42E-01 | 7.06E-01 | -5.914 |
| GSPT1 |  | -0.043 | 9.806 | -0.77 | 4.42E-01 | 7.06E-01 | -5.914 |
| SLC25A14 |  | 0.029 | 8.607 | 0.769 | 4.42E-01 | 7.06E-01 | -5.914 |
| CDH24 |  | -0.036 | 7.225 | -0.769 | 4.42E-01 | 7.06E-01 | -5.915 |
| CRABP2 |  | -0.053 | 7.569 | -0.769 | 4.42E-01 | 7.06E-01 | -5.915 |
| NPR2 |  | -0.036 | 7.074 | -0.769 | 4.42E-01 | 7.06E-01 | -5.915 |
| B3GNT4 |  | -0.05 | 5.278 | -0.769 | 4.42E-01 | 7.06E-01 | -5.915 |
| ARV1 |  | 0.027 | 8.374 | 0.768 | 4.43E-01 | 7.06E-01 | -5.915 |
| DDX46 |  | 0.031 | 8.167 | 0.768 | 4.43E-01 | 7.06E-01 | -5.915 |
| SLC25A3 |  | -0.023 | 11.821 | -0.768 | 4.43E-01 | 7.07E-01 | -5.916 |
| PTDSS1 |  | -0.033 | 10.511 | -0.768 | 4.43E-01 | 7.07E-01 | -5.916 |
| EFHD2 |  | 0.036 | 10.933 | 0.766 | 4.43E-01 | 7.07E-01 | -5.916 |
| CYP2C8 |  | 0.035 | 5.184 | 0.766 | 4.44E-01 | 7.07E-01 | -5.917 |
| C9orf114 |  | -0.029 | 7.698 | -0.766 | 4.44E-01 | 7.08E-01 | -5.917 |
| FBXO36 |  | 0.037 | 5.875 | 0.765 | 4.44E-01 | 7.08E-01 | -5.918 |
| TMC7 |  | -0.045 | 6.304 | -0.765 | 4.45E-01 | 7.08E-01 | -5.918 |
| PTPDC1 |  | 0.037 | 6.523 | 0.764 | 4.45E-01 | 7.08E-01 | -5.918 |
| BBS1 |  | -0.031 | 7.598 | -0.765 | 4.45E-01 | 7.08E-01 | -5.918 |
| CYBA |  | -0.037 | 10.234 | -0.764 | 4.45E-01 | 7.09E-01 | -5.919 |
| GNPDA2 |  | 0.047 | 7.047 | 0.763 | 4.45E-01 | 7.09E-01 | -5.919 |
| ALS2CL |  | 0.034 | 6.852 | 0.763 | 4.45E-01 | 7.09E-01 | -5.919 |
| FASN |  | 0.058 | 8.835 | 0.762 | 4.46E-01 | 7.09E-01 | -5.919 |
| INSIG2 |  | -0.038 | 8.355 | -0.763 | 4.46E-01 | 7.09E-01 | -5.919 |
| PHKB |  | -0.027 | 9.391 | -0.763 | 4.46E-01 | 7.09E-01 | -5.92 |
| APOA4 |  | 0.04 | 5 | 0.762 | 4.46E-01 | 7.09E-01 | -5.92 |
| CPSF1 |  | -0.046 | 8.338 | -0.762 | 4.47E-01 | 7.10E-01 | -5.92 |
| IL23A |  | 0.058 | 6.46 | 0.761 | 4.47E-01 | 7.10E-01 | -5.92 |
| PPP2R2C |  | -0.047 | 5.348 | -0.761 | 4.47E-01 | 7.10E-01 | -5.921 |
| OCIAD1 |  | 0.028 | 10.412 | 0.76 | 4.47E-01 | 7.10E-01 | -5.921 |
| CYP2E1 |  | 0.037 | 5.201 | 0.76 | 4.47E-01 | 7.10E-01 | -5.921 |
| COPS8 |  | -0.028 | 8.761 | -0.76 | 4.47E-01 | 7.10E-01 | -5.921 |
| OXGR1 |  | 0.056 | 5.397 | 0.759 | 4.47E-01 | 7.10E-01 | -5.921 |
| PUM2 |  | -0.038 | 9.562 | -0.76 | 4.48E-01 | 7.10E-01 | -5.922 |
| FLT3LG |  | -0.045 | 6.728 | -0.759 | 4.48E-01 | 7.11E-01 | -5.922 |
| SETDB1 |  | -0.027 | 8.959 | -0.758 | 4.49E-01 | 7.12E-01 | -5.923 |
| EXT1 |  | 0.035 | 8.848 | 0.757 | 4.49E-01 | 7.12E-01 | -5.923 |
| UBB |  | -0.022 | 14.22 | -0.758 | 4.49E-01 | 7.12E-01 | -5.923 |
| ITGA3 |  | 0.043 | 7.88 | 0.757 | 4.49E-01 | 7.12E-01 | -5.923 |
| ZNF585B |  | 0.031 | 6.877 | 0.756 | 4.49E-01 | 7.12E-01 | -5.924 |
| FANCL |  | -0.03 | 8.664 | -0.757 | 4.49E-01 | 7.12E-01 | -5.924 |
| SLC41A3 |  | 0.027 | 8.757 | 0.756 | 4.49E-01 | 7.12E-01 | -5.924 |
| BCL9 |  | 0.048 | 6.214 | 0.755 | 4.50E-01 | 7.13E-01 | -5.925 |
| ZNF212 |  | -0.027 | 9.069 | -0.756 | 4.50E-01 | 7.13E-01 | -5.925 |
| FOXC1 |  | -0.047 | 8.67 | -0.756 | 4.50E-01 | 7.13E-01 | -5.925 |
| SFXN5 |  | -0.044 | 7.024 | -0.756 | 4.50E-01 | 7.13E-01 | -5.925 |
| PAICS |  | 0.032 | 9.787 | 0.754 | 4.50E-01 | 7.13E-01 | -5.925 |
| GLI1 |  | 0.056 | 5.817 | 0.754 | 4.51E-01 | 7.13E-01 | -5.925 |
| TCF15 |  | -0.049 | 7.13 | -0.755 | 4.51E-01 | 7.13E-01 | -5.926 |
| TIGD3 |  | -0.042 | 5.976 | -0.754 | 4.51E-01 | 7.13E-01 | -5.926 |
| BARD1 |  | 0.032 | 7.951 | 0.753 | 4.51E-01 | 7.13E-01 | -5.926 |
| MVP |  | -0.036 | 11.105 | -0.754 | 4.51E-01 | 7.13E-01 | -5.926 |
| ZNF208 |  | -0.05 | 6.312 | -0.754 | 4.51E-01 | 7.13E-01 | -5.926 |
| PDGFB |  | -0.056 | 7.405 | -0.754 | 4.51E-01 | 7.13E-01 | -5.926 |
| MATN1 |  | 0.036 | 5.492 | 0.753 | 4.52E-01 | 7.13E-01 | -5.926 |
| SIX3 |  | -0.048 | 5.195 | -0.753 | 4.52E-01 | 7.13E-01 | -5.927 |
| CCL8 |  | 0.086 | 6.563 | 0.752 | 4.52E-01 | 7.13E-01 | -5.927 |
| LRRC4 |  | -0.047 | 6.385 | -0.753 | 4.52E-01 | 7.13E-01 | -5.927 |
| SUMO1 |  | 0.029 | 8.677 | 0.752 | 4.52E-01 | 7.13E-01 | -5.927 |
| TPP2 |  | -0.029 | 8.206 | -0.752 | 4.52E-01 | 7.13E-01 | -5.927 |
| SPEN |  | -0.042 | 9.399 | -0.752 | 4.52E-01 | 7.13E-01 | -5.927 |
| PANX1 |  | 0.037 | 7.819 | 0.751 | 4.52E-01 | 7.14E-01 | -5.927 |
| FAH |  | -0.044 | 8.001 | -0.752 | 4.53E-01 | 7.14E-01 | -5.928 |
| PAPLN |  | 0.049 | 6.585 | 0.751 | 4.53E-01 | 7.14E-01 | -5.928 |
| TINF2 |  | -0.026 | 10.799 | -0.751 | 4.53E-01 | 7.14E-01 | -5.928 |
| ENO3 |  | -0.04 | 7.441 | -0.751 | 4.53E-01 | 7.14E-01 | -5.928 |
| PPA2 |  | 0.038 | 8.994 | 0.75 | 4.53E-01 | 7.14E-01 | -5.928 |
| PGRMC2 |  | -0.032 | 11.875 | -0.751 | 4.53E-01 | 7.14E-01 | -5.928 |
| CHIA |  | -0.055 | 5.743 | -0.751 | 4.53E-01 | 7.14E-01 | -5.929 |
| RTN2 |  | -0.046 | 8.231 | -0.75 | 4.54E-01 | 7.14E-01 | -5.929 |
| AHSA1 |  | -0.029 | 10.43 | -0.75 | 4.54E-01 | 7.14E-01 | -5.929 |
| DHDDS |  | 0.031 | 7.441 | 0.748 | 4.54E-01 | 7.15E-01 | -5.93 |
| SMARCD2 |  | 0.037 | 9.247 | 0.748 | 4.54E-01 | 7.15E-01 | -5.93 |
| FBP2 |  | 0.036 | 4.549 | 0.747 | 4.55E-01 | 7.15E-01 | -5.93 |
| ZIM3 |  | 0.031 | 4.638 | 0.747 | 4.55E-01 | 7.15E-01 | -5.931 |
| GIMAP2 |  | -0.039 | 8.354 | -0.748 | 4.55E-01 | 7.15E-01 | -5.931 |
| AP3B1 |  | -0.029 | 9.877 | -0.748 | 4.55E-01 | 7.15E-01 | -5.931 |
| FBXO3 |  | -0.041 | 7.391 | -0.747 | 4.55E-01 | 7.15E-01 | -5.931 |
| AKAP9 |  | -0.034 | 7.441 | -0.747 | 4.56E-01 | 7.15E-01 | -5.931 |
| PPP1R13L |  | 0.048 | 8.975 | 0.746 | 4.56E-01 | 7.15E-01 | -5.931 |
| ITGAX |  | 0.04 | 6.635 | 0.746 | 4.56E-01 | 7.15E-01 | -5.931 |
| ANAPC5 |  | -0.025 | 10.754 | -0.746 | 4.56E-01 | 7.15E-01 | -5.932 |
| ANK2 |  | -0.041 | 5.393 | -0.746 | 4.56E-01 | 7.15E-01 | -5.932 |
| PLCZ1 |  | 0.048 | 4.552 | 0.745 | 4.56E-01 | 7.15E-01 | -5.932 |
| PKD2L2 |  | 0.035 | 4.971 | 0.745 | 4.56E-01 | 7.15E-01 | -5.932 |
| AKNA |  | 0.029 | 7.076 | 0.745 | 4.56E-01 | 7.15E-01 | -5.932 |
| TBRG4 |  | 0.027 | 8.597 | 0.745 | 4.56E-01 | 7.15E-01 | -5.932 |
| GZMB |  | -0.073 | 7.532 | -0.746 | 4.56E-01 | 7.15E-01 | -5.932 |
| ARHGEF17 |  | -0.037 | 8.433 | -0.746 | 4.56E-01 | 7.15E-01 | -5.932 |
| ICOS |  | -0.049 | 5.955 | -0.745 | 4.57E-01 | 7.16E-01 | -5.932 |
| GOLGA5 |  | 0.032 | 9.853 | 0.744 | 4.57E-01 | 7.16E-01 | -5.933 |
| AK5 |  | 0.044 | 5.569 | 0.744 | 4.57E-01 | 7.16E-01 | -5.933 |
| C8A |  | -0.038 | 5.044 | -0.745 | 4.57E-01 | 7.16E-01 | -5.933 |
| C20orf141 |  | 0.05 | 9.093 | 0.743 | 4.57E-01 | 7.16E-01 | -5.933 |
| EXOSC6 |  | 0.043 | 7.967 | 0.743 | 4.58E-01 | 7.16E-01 | -5.934 |
| SLC1A7 |  | -0.035 | 6.451 | -0.743 | 4.58E-01 | 7.16E-01 | -5.934 |
| BIRC5 |  | 0.054 | 8.408 | 0.742 | 4.58E-01 | 7.16E-01 | -5.934 |
| MYO3B |  | -0.03 | 4.984 | -0.743 | 4.58E-01 | 7.16E-01 | -5.934 |
| NEK3 |  | 0.033 | 7.551 | 0.742 | 4.58E-01 | 7.17E-01 | -5.934 |
| PCDHB1 |  | -0.047 | 5.156 | -0.743 | 4.58E-01 | 7.17E-01 | -5.934 |
| RAG2 |  | -0.035 | 4.708 | -0.742 | 4.59E-01 | 7.17E-01 | -5.935 |
| ASB16 |  | -0.047 | 6.841 | -0.741 | 4.59E-01 | 7.17E-01 | -5.935 |
| TAF1 |  | 0.027 | 7.225 | 0.74 | 4.59E-01 | 7.17E-01 | -5.935 |
| ZNF548 |  | 0.03 | 7.241 | 0.74 | 4.59E-01 | 7.17E-01 | -5.935 |
| FARS2 |  | -0.029 | 8.45 | -0.741 | 4.59E-01 | 7.17E-01 | -5.936 |
| NDUFB6 |  | 0.028 | 10.288 | 0.74 | 4.59E-01 | 7.17E-01 | -5.936 |
| PTPRE |  | 0.03 | 7.677 | 0.739 | 4.60E-01 | 7.17E-01 | -5.936 |
| PDE1C |  | 0.039 | 5.544 | 0.739 | 4.60E-01 | 7.17E-01 | -5.936 |
| GABRA1 |  | -0.035 | 4.897 | -0.74 | 4.60E-01 | 7.18E-01 | -5.936 |
| CNOT6 |  | -0.034 | 8.645 | -0.74 | 4.60E-01 | 7.18E-01 | -5.936 |
| VPS13C |  | -0.027 | 7.233 | -0.74 | 4.60E-01 | 7.18E-01 | -5.936 |
| PLEKHA8 |  | -0.04 | 6.16 | -0.739 | 4.60E-01 | 7.18E-01 | -5.937 |
| RAPGEF4 |  | -0.045 | 5.36 | -0.739 | 4.61E-01 | 7.18E-01 | -5.937 |
| RNF133 |  | 0.028 | 4.534 | 0.737 | 4.61E-01 | 7.18E-01 | -5.938 |
| ZSWIM3 |  | 0.031 | 7.108 | 0.737 | 4.61E-01 | 7.18E-01 | -5.938 |
| ADNP |  | -0.025 | 9.376 | -0.738 | 4.61E-01 | 7.18E-01 | -5.938 |
| COPZ2 |  | 0.045 | 8.125 | 0.736 | 4.61E-01 | 7.18E-01 | -5.938 |
| RPL15 |  | -0.027 | 12.103 | -0.737 | 4.61E-01 | 7.18E-01 | -5.938 |
| USP28 |  | -0.042 | 5.951 | -0.737 | 4.61E-01 | 7.18E-01 | -5.938 |
| SLITRK2 |  | 0.03 | 4.957 | 0.736 | 4.61E-01 | 7.18E-01 | -5.938 |
| RBM15 |  | 0.032 | 7.541 | 0.736 | 4.62E-01 | 7.18E-01 | -5.938 |
| NME4 |  | -0.042 | 9.624 | -0.736 | 4.62E-01 | 7.19E-01 | -5.939 |
| HLA-DRA |  | -0.07 | 9.458 | -0.736 | 4.63E-01 | 7.19E-01 | -5.939 |
| ANTXR1 |  | -0.047 | 7.846 | -0.735 | 4.63E-01 | 7.19E-01 | -5.939 |
| RBM14 |  | 0.028 | 9.319 | 0.734 | 4.63E-01 | 7.19E-01 | -5.939 |
| P2RX4 |  | 0.039 | 9.35 | 0.734 | 4.63E-01 | 7.19E-01 | -5.94 |
| EFHD1 |  | 0.052 | 9.964 | 0.734 | 4.63E-01 | 7.19E-01 | -5.94 |
| PIAS4 |  | 0.04 | 8.048 | 0.734 | 4.63E-01 | 7.19E-01 | -5.94 |
| WNT7A |  | 0.058 | 6.956 | 0.734 | 4.63E-01 | 7.19E-01 | -5.94 |
| PTK6 |  | -0.039 | 6.379 | -0.734 | 4.63E-01 | 7.19E-01 | -5.94 |
| SLC36A4 |  | 0.032 | 7.932 | 0.733 | 4.63E-01 | 7.20E-01 | -5.94 |
| FABP4 |  | 0.054 | 7.847 | 0.733 | 4.64E-01 | 7.20E-01 | -5.94 |
| TP53BP1 |  | -0.028 | 8.945 | -0.733 | 4.64E-01 | 7.20E-01 | -5.941 |
| HHLA1 |  | -0.047 | 5.267 | -0.733 | 4.64E-01 | 7.20E-01 | -5.941 |
| CPT1A |  | -0.032 | 7.544 | -0.733 | 4.64E-01 | 7.20E-01 | -5.941 |
| NRIP2 |  | -0.048 | 5.743 | -0.733 | 4.64E-01 | 7.20E-01 | -5.941 |
| MLN |  | -0.042 | 5.175 | -0.732 | 4.64E-01 | 7.20E-01 | -5.942 |
| PRAP1 |  | 0.043 | 6.368 | 0.731 | 4.65E-01 | 7.20E-01 | -5.942 |
| SMAD7 |  | -0.036 | 9.125 | -0.732 | 4.65E-01 | 7.20E-01 | -5.942 |
| KCNE2 |  | 0.049 | 5.168 | 0.731 | 4.65E-01 | 7.20E-01 | -5.942 |
| DCN |  | -0.056 | 9.973 | -0.731 | 4.65E-01 | 7.20E-01 | -5.942 |
| RBM11 |  | 0.045 | 5.277 | 0.73 | 4.65E-01 | 7.21E-01 | -5.943 |
| CEBPZ |  | 0.037 | 9.716 | 0.73 | 4.65E-01 | 7.21E-01 | -5.943 |
| WDR1 |  | 0.029 | 10.6 | 0.729 | 4.66E-01 | 7.21E-01 | -5.943 |
| ATP2B2 |  | 0.033 | 4.869 | 0.729 | 4.66E-01 | 7.21E-01 | -5.943 |
| AP2A1 |  | -0.051 | 8.011 | -0.728 | 4.67E-01 | 7.22E-01 | -5.944 |
| SH2D3C |  | -0.043 | 8.142 | -0.728 | 4.67E-01 | 7.22E-01 | -5.944 |
| PKHD1 |  | 0.025 | 4.949 | 0.727 | 4.67E-01 | 7.22E-01 | -5.945 |
| TREH |  | 0.042 | 4.821 | 0.727 | 4.67E-01 | 7.23E-01 | -5.945 |
| DCLRE1A |  | -0.034 | 7.874 | -0.727 | 4.68E-01 | 7.23E-01 | -5.945 |
| SLC39A5 |  | -0.045 | 5.82 | -0.727 | 4.68E-01 | 7.23E-01 | -5.945 |
| RHCG |  | -0.041 | 5.536 | -0.726 | 4.68E-01 | 7.24E-01 | -5.946 |
| PLA2G3 |  | 0.034 | 4.927 | 0.725 | 4.69E-01 | 7.24E-01 | -5.946 |
| CACNB3 |  | 0.041 | 7.576 | 0.724 | 4.69E-01 | 7.24E-01 | -5.946 |
| CD200 |  | -0.057 | 8.377 | -0.725 | 4.69E-01 | 7.24E-01 | -5.947 |
| TRIB2 |  | -0.039 | 7.897 | -0.725 | 4.69E-01 | 7.24E-01 | -5.947 |
| ING5 |  | 0.027 | 6.863 | 0.724 | 4.69E-01 | 7.24E-01 | -5.947 |
| HERC1 |  | 0.027 | 9.171 | 0.724 | 4.69E-01 | 7.24E-01 | -5.947 |
| ABCG1 |  | 0.04 | 8.229 | 0.723 | 4.69E-01 | 7.24E-01 | -5.947 |
| DHTKD1 |  | 0.031 | 8.011 | 0.723 | 4.69E-01 | 7.24E-01 | -5.947 |
| GPR84 |  | -0.044 | 6.751 | -0.724 | 4.69E-01 | 7.24E-01 | -5.947 |
| RABIF |  | 0.03 | 7.075 | 0.723 | 4.69E-01 | 7.24E-01 | -5.947 |
| MEF2C |  | 0.045 | 8.185 | 0.723 | 4.70E-01 | 7.24E-01 | -5.947 |
| FILIP1 |  | -0.05 | 6.205 | -0.723 | 4.70E-01 | 7.24E-01 | -5.948 |
| DTX3L |  | -0.043 | 7.937 | -0.723 | 4.70E-01 | 7.25E-01 | -5.948 |
| DNTTIP1 |  | 0.021 | 10.3 | 0.721 | 4.71E-01 | 7.25E-01 | -5.949 |
| DEK |  | 0.042 | 10.812 | 0.721 | 4.71E-01 | 7.25E-01 | -5.949 |
| ABCC13 |  | 0.04 | 5.816 | 0.721 | 4.71E-01 | 7.25E-01 | -5.949 |
| DAPK1 |  | 0.04 | 9.097 | 0.72 | 4.71E-01 | 7.25E-01 | -5.949 |
| GNAT1 |  | 0.033 | 4.808 | 0.72 | 4.71E-01 | 7.26E-01 | -5.95 |
| CARS |  | 0.031 | 8.524 | 0.72 | 4.72E-01 | 7.26E-01 | -5.95 |
| LIPF |  | 0.036 | 4.859 | 0.72 | 4.72E-01 | 7.26E-01 | -5.95 |
| ST6GALNAC4 |  | -0.046 | 7.735 | -0.72 | 4.72E-01 | 7.26E-01 | -5.95 |
| ZNF595 |  | -0.038 | 7.505 | -0.72 | 4.72E-01 | 7.26E-01 | -5.95 |
| TJP3 |  | -0.041 | 6.934 | -0.72 | 4.72E-01 | 7.26E-01 | -5.95 |
| CAV1 |  | -0.041 | 11.046 | -0.718 | 4.73E-01 | 7.27E-01 | -5.951 |
| EVI2A |  | 0.037 | 7.579 | 0.717 | 4.73E-01 | 7.27E-01 | -5.951 |
| MADD |  | -0.027 | 8.687 | -0.717 | 4.74E-01 | 7.28E-01 | -5.952 |
| LRRN1 |  | 0.058 | 5.589 | 0.716 | 4.74E-01 | 7.28E-01 | -5.952 |
| RBMS2 |  | -0.036 | 8.657 | -0.717 | 4.74E-01 | 7.28E-01 | -5.953 |
| TCF12 |  | 0.027 | 8.386 | 0.715 | 4.74E-01 | 7.28E-01 | -5.953 |
| CCT4 |  | -0.025 | 10.621 | -0.716 | 4.74E-01 | 7.28E-01 | -5.953 |
| OR51E2 |  | -0.045 | 5.781 | -0.716 | 4.75E-01 | 7.28E-01 | -5.953 |
| GYS1 |  | -0.046 | 8.255 | -0.715 | 4.75E-01 | 7.28E-01 | -5.953 |
| EIF5A2 |  | -0.043 | 6.475 | -0.715 | 4.75E-01 | 7.28E-01 | -5.953 |
| EPB41 |  | -0.041 | 6.356 | -0.715 | 4.75E-01 | 7.28E-01 | -5.953 |
| RPL31 |  | -0.039 | 12.612 | -0.715 | 4.75E-01 | 7.28E-01 | -5.954 |
| NAV2 |  | -0.034 | 6.484 | -0.713 | 4.76E-01 | 7.30E-01 | -5.955 |
| ZNF346 |  | 0.04 | 6.695 | 0.712 | 4.76E-01 | 7.30E-01 | -5.955 |
| KCNK1 |  | 0.056 | 7.086 | 0.712 | 4.76E-01 | 7.30E-01 | -5.955 |
| GABRA2 |  | -0.038 | 4.715 | -0.713 | 4.76E-01 | 7.30E-01 | -5.955 |
| FUT8 |  | 0.033 | 8.815 | 0.712 | 4.77E-01 | 7.30E-01 | -5.955 |
| OIP5 |  | 0.051 | 7.474 | 0.711 | 4.77E-01 | 7.30E-01 | -5.956 |
| NOS1 |  | 0.032 | 5.436 | 0.711 | 4.77E-01 | 7.30E-01 | -5.956 |
| GLDC |  | -0.039 | 8.91 | -0.711 | 4.77E-01 | 7.30E-01 | -5.956 |
| CDKL1 |  | -0.042 | 6.473 | -0.711 | 4.77E-01 | 7.30E-01 | -5.956 |
| EIF2S1 |  | 0.025 | 8.75 | 0.71 | 4.77E-01 | 7.30E-01 | -5.956 |
| NUP155 |  | 0.024 | 8.201 | 0.71 | 4.77E-01 | 7.30E-01 | -5.956 |
| RXRG |  | -0.037 | 4.982 | -0.711 | 4.78E-01 | 7.30E-01 | -5.956 |
| ITCH |  | 0.025 | 8.174 | 0.71 | 4.78E-01 | 7.30E-01 | -5.956 |
| PAIP1 |  | 0.021 | 8.794 | 0.71 | 4.78E-01 | 7.30E-01 | -5.956 |
| ELAVL1 |  | -0.025 | 7.888 | -0.711 | 4.78E-01 | 7.30E-01 | -5.957 |
| DNMT3B |  | 0.036 | 6.145 | 0.709 | 4.78E-01 | 7.30E-01 | -5.957 |
| AACS |  | 0.036 | 8.423 | 0.709 | 4.78E-01 | 7.31E-01 | -5.957 |
| ELF2 |  | -0.03 | 8.02 | -0.709 | 4.79E-01 | 7.31E-01 | -5.957 |
| MPZL1 |  | 0.03 | 8.984 | 0.708 | 4.79E-01 | 7.31E-01 | -5.958 |
| SSX3 |  | -0.053 | 5.507 | -0.709 | 4.79E-01 | 7.31E-01 | -5.958 |
| CD96 |  | 0.039 | 5.846 | 0.708 | 4.79E-01 | 7.31E-01 | -5.958 |
| NIPSNAP1 |  | 0.032 | 8.761 | 0.707 | 4.79E-01 | 7.31E-01 | -5.958 |
| ACOX1 |  | 0.027 | 8.402 | 0.707 | 4.79E-01 | 7.31E-01 | -5.958 |
| ACSL5 |  | -0.034 | 7.612 | -0.708 | 4.79E-01 | 7.31E-01 | -5.958 |
| CANX |  | -0.028 | 9.799 | -0.708 | 4.79E-01 | 7.31E-01 | -5.958 |
| OR51B5 |  | -0.038 | 5.243 | -0.708 | 4.79E-01 | 7.31E-01 | -5.958 |
| WDR7 |  | 0.025 | 7.854 | 0.706 | 4.80E-01 | 7.31E-01 | -5.959 |
| SLC22A16 |  | 0.047 | 5.961 | 0.706 | 4.80E-01 | 7.31E-01 | -5.959 |
| CYP46A1 |  | -0.039 | 4.913 | -0.707 | 4.80E-01 | 7.31E-01 | -5.959 |
| JRKL |  | 0.03 | 7.237 | 0.706 | 4.80E-01 | 7.31E-01 | -5.959 |
| ADCY6 |  | 0.039 | 7.432 | 0.706 | 4.80E-01 | 7.31E-01 | -5.959 |
| MEN1 |  | 0.04 | 7.269 | 0.705 | 4.81E-01 | 7.32E-01 | -5.96 |
| LRRC15 |  | -0.08 | 5.796 | -0.706 | 4.81E-01 | 7.32E-01 | -5.96 |
| KIAA2018 |  | 0.038 | 6.625 | 0.704 | 4.81E-01 | 7.32E-01 | -5.96 |
| ESD |  | -0.027 | 9.925 | -0.704 | 4.82E-01 | 7.33E-01 | -5.961 |
| GFRA1 |  | -0.034 | 6.075 | -0.704 | 4.82E-01 | 7.33E-01 | -5.961 |
| TAOK2 |  | 0.033 | 8.336 | 0.703 | 4.82E-01 | 7.33E-01 | -5.961 |
| FOXQ1 |  | 0.049 | 7.869 | 0.703 | 4.82E-01 | 7.33E-01 | -5.961 |
| TBC1D1 |  | -0.025 | 8.926 | -0.704 | 4.82E-01 | 7.33E-01 | -5.961 |
| MICAL1 |  | -0.04 | 9.191 | -0.704 | 4.82E-01 | 7.33E-01 | -5.961 |
| OR2J2 |  | 0.035 | 4.865 | 0.702 | 4.82E-01 | 7.33E-01 | -5.961 |
| ITK |  | -0.045 | 6.009 | -0.703 | 4.82E-01 | 7.33E-01 | -5.962 |
| PEMT |  | -0.036 | 8.08 | -0.703 | 4.83E-01 | 7.33E-01 | -5.962 |
| DIRAS2 |  | 0.055 | 5.031 | 0.702 | 4.83E-01 | 7.33E-01 | -5.962 |
| ZFPM2 |  | 0.038 | 6.151 | 0.701 | 4.83E-01 | 7.33E-01 | -5.963 |
| PPP1R9B |  | -0.037 | 7.56 | -0.702 | 4.83E-01 | 7.33E-01 | -5.963 |
| TAF1A |  | -0.033 | 7.028 | -0.702 | 4.83E-01 | 7.33E-01 | -5.963 |
| TEC |  | -0.034 | 5.56 | -0.701 | 4.84E-01 | 7.35E-01 | -5.963 |
| WNT4 |  | 0.056 | 5.876 | 0.697 | 4.86E-01 | 7.37E-01 | -5.965 |
| HS6ST3 |  | 0.031 | 5.022 | 0.696 | 4.86E-01 | 7.37E-01 | -5.966 |
| MTMR1 |  | -0.029 | 7.948 | -0.697 | 4.86E-01 | 7.37E-01 | -5.966 |
| FGF5 |  | -0.031 | 4.854 | -0.697 | 4.86E-01 | 7.38E-01 | -5.966 |
| SPATA9 |  | 0.039 | 6.063 | 0.695 | 4.87E-01 | 7.38E-01 | -5.966 |
| ARHGAP22 |  | 0.036 | 7.342 | 0.694 | 4.88E-01 | 7.39E-01 | -5.967 |
| ROPN1L |  | 0.037 | 6.702 | 0.693 | 4.88E-01 | 7.39E-01 | -5.968 |
| KCNC3 |  | 0.032 | 6.444 | 0.693 | 4.88E-01 | 7.39E-01 | -5.968 |
| NEXN |  | 0.051 | 7.957 | 0.693 | 4.88E-01 | 7.39E-01 | -5.968 |
| FCGR2B |  | 0.035 | 9.269 | 0.692 | 4.89E-01 | 7.40E-01 | -5.968 |
| CRYZL1 |  | 0.022 | 7.824 | 0.692 | 4.89E-01 | 7.40E-01 | -5.968 |
| SIGLEC5 |  | 0.036 | 6.233 | 0.692 | 4.89E-01 | 7.40E-01 | -5.968 |
| COL6A2 |  | -0.05 | 8.894 | -0.693 | 4.89E-01 | 7.40E-01 | -5.969 |
| MRPL21 |  | 0.026 | 10.044 | 0.691 | 4.89E-01 | 7.40E-01 | -5.969 |
| FGL1 |  | -0.037 | 6.655 | -0.692 | 4.89E-01 | 7.40E-01 | -5.969 |
| WASL |  | 0.041 | 9.231 | 0.691 | 4.89E-01 | 7.40E-01 | -5.969 |
| DDX58 |  | -0.046 | 8.523 | -0.692 | 4.89E-01 | 7.40E-01 | -5.969 |
| PDK1 |  | 0.038 | 6.978 | 0.691 | 4.89E-01 | 7.40E-01 | -5.969 |
| ATXN2L |  | 0.025 | 8.166 | 0.691 | 4.90E-01 | 7.40E-01 | -5.969 |
| ZMYND12 |  | 0.044 | 5.651 | 0.69 | 4.90E-01 | 7.40E-01 | -5.97 |
| C1orf52 |  | 0.023 | 9.182 | 0.689 | 4.90E-01 | 7.40E-01 | -5.97 |
| PTH |  | 0.039 | 4.557 | 0.689 | 4.90E-01 | 7.40E-01 | -5.97 |
| TBXA2R |  | -0.029 | 7.417 | -0.69 | 4.90E-01 | 7.40E-01 | -5.97 |
| TRIM46 |  | -0.033 | 6.329 | -0.69 | 4.90E-01 | 7.40E-01 | -5.97 |
| IL1RL2 |  | 0.041 | 5.41 | 0.689 | 4.90E-01 | 7.40E-01 | -5.97 |
| MOGAT3 |  | -0.04 | 5.552 | -0.69 | 4.91E-01 | 7.40E-01 | -5.97 |
| FBXO44 |  | 0.035 | 6.466 | 0.689 | 4.91E-01 | 7.40E-01 | -5.971 |
| SNX24 |  | -0.026 | 8.62 | -0.69 | 4.91E-01 | 7.40E-01 | -5.971 |
| NFAM1 |  | -0.029 | 6.765 | -0.689 | 4.91E-01 | 7.41E-01 | -5.971 |
| CENPJ |  | 0.03 | 7.655 | 0.688 | 4.92E-01 | 7.41E-01 | -5.971 |
| SS18L1 |  | -0.029 | 7.717 | -0.688 | 4.92E-01 | 7.41E-01 | -5.972 |
| PHOSPHO1 |  | -0.045 | 7.377 | -0.688 | 4.92E-01 | 7.41E-01 | -5.972 |
| ZNF644 |  | 0.039 | 7.793 | 0.686 | 4.92E-01 | 7.41E-01 | -5.972 |
| ZBED2 |  | 0.067 | 5.678 | 0.686 | 4.93E-01 | 7.41E-01 | -5.972 |
| PRDX2 |  | 0.037 | 9.54 | 0.686 | 4.93E-01 | 7.41E-01 | -5.973 |
| ZNF502 |  | 0.032 | 7.605 | 0.686 | 4.93E-01 | 7.41E-01 | -5.973 |
| PTPRS |  | 0.032 | 5.988 | 0.686 | 4.93E-01 | 7.41E-01 | -5.973 |
| CCL21 |  | -0.05 | 6.12 | -0.686 | 4.93E-01 | 7.42E-01 | -5.973 |
| LENG8 |  | -0.035 | 7.297 | -0.686 | 4.93E-01 | 7.42E-01 | -5.973 |
| POLA2 |  | -0.03 | 7.942 | -0.686 | 4.93E-01 | 7.42E-01 | -5.973 |
| PLSCR2 |  | 0.033 | 4.735 | 0.684 | 4.94E-01 | 7.42E-01 | -5.974 |
| ARHGAP1 |  | -0.037 | 9.921 | -0.685 | 4.94E-01 | 7.42E-01 | -5.974 |
| SNAP91 |  | 0.038 | 5.164 | 0.684 | 4.94E-01 | 7.42E-01 | -5.974 |
| CLCA4 |  | -0.035 | 4.61 | -0.684 | 4.94E-01 | 7.43E-01 | -5.974 |
| CCT6B |  | 0.041 | 6.258 | 0.683 | 4.94E-01 | 7.43E-01 | -5.974 |
| RNASET2 |  | 0.036 | 10.11 | 0.682 | 4.95E-01 | 7.44E-01 | -5.975 |
| PARP3 |  | -0.042 | 6.516 | -0.682 | 4.95E-01 | 7.44E-01 | -5.976 |
| SNRPF |  | 0.037 | 10.824 | 0.681 | 4.96E-01 | 7.44E-01 | -5.976 |
| COX5B |  | 0.026 | 12.369 | 0.681 | 4.96E-01 | 7.44E-01 | -5.976 |
| C11orf30 |  | -0.025 | 7.826 | -0.681 | 4.96E-01 | 7.44E-01 | -5.976 |
| FCHSD2 |  | -0.024 | 8.498 | -0.681 | 4.96E-01 | 7.44E-01 | -5.976 |
| SIRT6 |  | -0.027 | 7.908 | -0.681 | 4.96E-01 | 7.45E-01 | -5.976 |
| MRPL4 |  | 0.023 | 8.905 | 0.68 | 4.96E-01 | 7.45E-01 | -5.977 |
| ZGPAT |  | 0.032 | 8.808 | 0.678 | 4.98E-01 | 7.46E-01 | -5.978 |
| CDX4 |  | -0.033 | 4.902 | -0.679 | 4.98E-01 | 7.46E-01 | -5.978 |
| GATA3 |  | 0.03 | 9.411 | 0.677 | 4.98E-01 | 7.46E-01 | -5.978 |
| STK32A |  | -0.034 | 4.815 | -0.678 | 4.98E-01 | 7.46E-01 | -5.978 |
| EZH2 |  | 0.031 | 7.818 | 0.677 | 4.98E-01 | 7.46E-01 | -5.978 |
| JAK2 |  | -0.033 | 8.292 | -0.678 | 4.98E-01 | 7.46E-01 | -5.978 |
| RAB1A |  | 0.03 | 10.524 | 0.677 | 4.98E-01 | 7.46E-01 | -5.979 |
| TAF5 |  | -0.036 | 7.357 | -0.678 | 4.98E-01 | 7.46E-01 | -5.979 |
| PNRC2 |  | -0.028 | 9.681 | -0.678 | 4.98E-01 | 7.46E-01 | -5.979 |
| TEX13B |  | -0.044 | 5.455 | -0.676 | 4.99E-01 | 7.47E-01 | -5.98 |
| ACAD8 |  | 0.03 | 7.369 | 0.675 | 4.99E-01 | 7.47E-01 | -5.98 |
| APOL4 |  | 0.038 | 6.156 | 0.675 | 4.99E-01 | 7.47E-01 | -5.98 |
| BLVRA |  | 0.049 | 10.271 | 0.674 | 5.00E-01 | 7.47E-01 | -5.98 |
| BMP2 |  | 0.068 | 7.375 | 0.674 | 5.00E-01 | 7.47E-01 | -5.98 |
| ZRANB1 |  | 0.037 | 8.109 | 0.674 | 5.00E-01 | 7.47E-01 | -5.98 |
| RERE |  | 0.032 | 7.807 | 0.674 | 5.00E-01 | 7.48E-01 | -5.98 |
| ZNF304 |  | -0.034 | 6.788 | -0.675 | 5.00E-01 | 7.48E-01 | -5.981 |
| CPEB2 |  | -0.042 | 6.188 | -0.674 | 5.00E-01 | 7.48E-01 | -5.981 |
| YPEL1 |  | 0.031 | 6.958 | 0.673 | 5.01E-01 | 7.48E-01 | -5.981 |
| CLK1 |  | 0.044 | 9.307 | 0.673 | 5.01E-01 | 7.48E-01 | -5.981 |
| TACR3 |  | -0.041 | 4.843 | -0.674 | 5.01E-01 | 7.48E-01 | -5.981 |
| ST13 |  | 0.026 | 10.097 | 0.672 | 5.01E-01 | 7.48E-01 | -5.982 |
| COL4A1 |  | -0.047 | 11.189 | -0.673 | 5.01E-01 | 7.49E-01 | -5.982 |
| ZNF343 |  | -0.032 | 6.327 | -0.672 | 5.02E-01 | 7.49E-01 | -5.982 |
| FASLG |  | 0.046 | 5.73 | 0.671 | 5.02E-01 | 7.49E-01 | -5.982 |
| TAF1L |  | -0.047 | 6.538 | -0.672 | 5.02E-01 | 7.49E-01 | -5.982 |
| HSPA12B |  | 0.031 | 7.55 | 0.671 | 5.02E-01 | 7.49E-01 | -5.982 |
| PPBP |  | 0.051 | 8.199 | 0.67 | 5.03E-01 | 7.50E-01 | -5.983 |
| AGER |  | -0.036 | 6.879 | -0.671 | 5.03E-01 | 7.50E-01 | -5.983 |
| HIST1H2BJ |  | -0.042 | 7.432 | -0.671 | 5.03E-01 | 7.50E-01 | -5.983 |
| UBR2 |  | -0.026 | 8.494 | -0.67 | 5.03E-01 | 7.50E-01 | -5.984 |
| DDX3Y |  | -0.122 | 6.734 | -0.669 | 5.04E-01 | 7.50E-01 | -5.984 |
| LUM |  | 0.048 | 10.28 | 0.668 | 5.04E-01 | 7.50E-01 | -5.984 |
| TRIB3 |  | 0.035 | 9.117 | 0.668 | 5.04E-01 | 7.50E-01 | -5.984 |
| SLC39A13 |  | 0.022 | 9.885 | 0.668 | 5.04E-01 | 7.50E-01 | -5.984 |
| BSND |  | -0.04 | 4.938 | -0.669 | 5.04E-01 | 7.51E-01 | -5.984 |
| PURB |  | 0.037 | 8.985 | 0.667 | 5.05E-01 | 7.51E-01 | -5.985 |
| KCNJ6 |  | 0.034 | 4.993 | 0.667 | 5.05E-01 | 7.51E-01 | -5.985 |
| BIN3 |  | -0.023 | 8.169 | -0.668 | 5.05E-01 | 7.51E-01 | -5.985 |
| MRPL42 |  | 0.028 | 8.345 | 0.666 | 5.05E-01 | 7.51E-01 | -5.985 |
| HLA-DMB |  | 0.043 | 8.764 | 0.666 | 5.05E-01 | 7.51E-01 | -5.985 |
| PIK3C2G |  | 0.029 | 4.666 | 0.666 | 5.05E-01 | 7.51E-01 | -5.985 |
| ASXL1 |  | -0.031 | 8.905 | -0.667 | 5.05E-01 | 7.51E-01 | -5.986 |
| FOXL2 |  | 0.046 | 6.38 | 0.666 | 5.05E-01 | 7.51E-01 | -5.986 |
| DUSP11 |  | -0.026 | 8.632 | -0.666 | 5.06E-01 | 7.51E-01 | -5.986 |
| KIF21A |  | 0.045 | 7.838 | 0.665 | 5.06E-01 | 7.51E-01 | -5.986 |
| SOCS5 |  | -0.034 | 7.641 | -0.665 | 5.06E-01 | 7.52E-01 | -5.987 |
| CAMLG |  | 0.03 | 9.005 | 0.664 | 5.06E-01 | 7.52E-01 | -5.987 |
| LDOC1L |  | -0.029 | 8.051 | -0.665 | 5.07E-01 | 7.52E-01 | -5.987 |
| TEAD1 |  | -0.039 | 8.554 | -0.664 | 5.07E-01 | 7.52E-01 | -5.987 |
| LY6G5C |  | 0.033 | 6.2 | 0.663 | 5.07E-01 | 7.52E-01 | -5.987 |
| OR51E1 |  | -0.04 | 5.8 | -0.664 | 5.07E-01 | 7.52E-01 | -5.987 |
| CDC42EP3 |  | -0.035 | 7.958 | -0.664 | 5.07E-01 | 7.52E-01 | -5.987 |
| MASP1 |  | 0.026 | 5.387 | 0.662 | 5.08E-01 | 7.52E-01 | -5.988 |
| WBSCR17 |  | 0.052 | 6.475 | 0.662 | 5.08E-01 | 7.52E-01 | -5.988 |
| EFHC2 |  | 0.041 | 5.477 | 0.662 | 5.08E-01 | 7.52E-01 | -5.988 |
| DNAJB4 |  | 0.051 | 7.541 | 0.662 | 5.08E-01 | 7.53E-01 | -5.988 |
| ACACA |  | 0.034 | 6.67 | 0.662 | 5.08E-01 | 7.53E-01 | -5.988 |
| TARDBP |  | 0.021 | 8.933 | 0.661 | 5.08E-01 | 7.53E-01 | -5.988 |
| MAPK12 |  | -0.035 | 6.84 | -0.662 | 5.08E-01 | 7.53E-01 | -5.988 |
| ATF7IP2 |  | 0.035 | 6.937 | 0.661 | 5.08E-01 | 7.53E-01 | -5.989 |
| HGD |  | -0.042 | 5.968 | -0.662 | 5.09E-01 | 7.53E-01 | -5.989 |
| ERBB2IP |  | 0.033 | 8.851 | 0.66 | 5.09E-01 | 7.53E-01 | -5.989 |
| HBS1L |  | 0.035 | 6.173 | 0.66 | 5.09E-01 | 7.53E-01 | -5.989 |
| VN1R4 |  | -0.036 | 4.985 | -0.661 | 5.09E-01 | 7.53E-01 | -5.989 |
| PIGH |  | -0.033 | 9.066 | -0.661 | 5.09E-01 | 7.53E-01 | -5.99 |
| LAS1L |  | -0.031 | 9.434 | -0.66 | 5.09E-01 | 7.53E-01 | -5.99 |
| DNTT |  | -0.033 | 5.026 | -0.66 | 5.10E-01 | 7.54E-01 | -5.99 |
| PCP4 |  | 0.046 | 6.785 | 0.659 | 5.10E-01 | 7.54E-01 | -5.99 |
| SLC7A6 |  | 0.031 | 7.633 | 0.659 | 5.10E-01 | 7.54E-01 | -5.99 |
| ZNF227 |  | 0.03 | 7.179 | 0.658 | 5.10E-01 | 7.54E-01 | -5.99 |
| GAB3 |  | 0.03 | 6.82 | 0.658 | 5.10E-01 | 7.54E-01 | -5.991 |
| SLC4A3 |  | 0.039 | 6.755 | 0.658 | 5.10E-01 | 7.54E-01 | -5.991 |
| FAHD1 |  | 0.03 | 7.938 | 0.658 | 5.11E-01 | 7.54E-01 | -5.991 |
| TUSC1 |  | 0.042 | 8.205 | 0.658 | 5.11E-01 | 7.54E-01 | -5.991 |
| APOC1 |  | 0.051 | 7.719 | 0.657 | 5.11E-01 | 7.54E-01 | -5.991 |
| SPRY2 |  | -0.036 | 8.353 | -0.657 | 5.12E-01 | 7.55E-01 | -5.992 |
| CIRBP |  | -0.024 | 10.939 | -0.656 | 5.12E-01 | 7.55E-01 | -5.992 |
| RAB40B |  | 0.033 | 7.552 | 0.655 | 5.12E-01 | 7.55E-01 | -5.992 |
| SF3A3 |  | 0.024 | 9.971 | 0.654 | 5.13E-01 | 7.56E-01 | -5.993 |
| CLEC1B |  | -0.049 | 6.13 | -0.655 | 5.13E-01 | 7.56E-01 | -5.993 |
| PTTG2 |  | -0.041 | 7.63 | -0.655 | 5.13E-01 | 7.56E-01 | -5.993 |
| GPR155 |  | -0.026 | 7.321 | -0.655 | 5.13E-01 | 7.56E-01 | -5.993 |
| PCBP2 |  | -0.028 | 10.853 | -0.654 | 5.13E-01 | 7.56E-01 | -5.994 |
| ZAP70 |  | 0.046 | 6.363 | 0.652 | 5.14E-01 | 7.57E-01 | -5.994 |
| FZD6 |  | 0.054 | 7.466 | 0.652 | 5.14E-01 | 7.57E-01 | -5.994 |
| C21orf33 |  | -0.025 | 10.079 | -0.653 | 5.14E-01 | 7.57E-01 | -5.994 |
| DDX51 |  | 0.032 | 7.801 | 0.651 | 5.14E-01 | 7.57E-01 | -5.995 |
| GNLY |  | -0.07 | 6.827 | -0.652 | 5.15E-01 | 7.57E-01 | -5.995 |
| FAM49B |  | -0.032 | 8.946 | -0.652 | 5.15E-01 | 7.57E-01 | -5.995 |
| MYH3 |  | 0.038 | 6.28 | 0.651 | 5.15E-01 | 7.57E-01 | -5.995 |
| TTK |  | 0.051 | 7.274 | 0.651 | 5.15E-01 | 7.57E-01 | -5.995 |
| FAM19A2 |  | 0.046 | 5.709 | 0.651 | 5.15E-01 | 7.57E-01 | -5.995 |
| GRIN2D |  | 0.038 | 8.369 | 0.65 | 5.15E-01 | 7.58E-01 | -5.995 |
| OPA3 |  | -0.03 | 7.479 | -0.651 | 5.15E-01 | 7.58E-01 | -5.996 |
| ATP6V1E1 |  | 0.026 | 11.078 | 0.65 | 5.16E-01 | 7.58E-01 | -5.996 |
| KRT6B |  | -0.03 | 5.275 | -0.65 | 5.16E-01 | 7.58E-01 | -5.996 |
| ICK |  | -0.046 | 8.07 | -0.65 | 5.16E-01 | 7.58E-01 | -5.996 |
| FTHL17 |  | 0.049 | 8.262 | 0.649 | 5.16E-01 | 7.58E-01 | -5.996 |
| PARP14 |  | 0.043 | 8.499 | 0.649 | 5.16E-01 | 7.58E-01 | -5.996 |
| EPC1 |  | -0.031 | 8.105 | -0.65 | 5.16E-01 | 7.58E-01 | -5.996 |
| PRKCG |  | -0.035 | 5.619 | -0.65 | 5.16E-01 | 7.58E-01 | -5.996 |
| PIPOX |  | 0.06 | 6.923 | 0.648 | 5.17E-01 | 7.58E-01 | -5.997 |
| CYP2S1 |  | -0.042 | 6.295 | -0.648 | 5.18E-01 | 7.59E-01 | -5.998 |
| PFKL |  | 0.043 | 9.097 | 0.646 | 5.18E-01 | 7.59E-01 | -5.998 |
| CNOT10 |  | 0.025 | 8.743 | 0.646 | 5.18E-01 | 7.60E-01 | -5.998 |
| AKT1S1 |  | 0.039 | 7.863 | 0.646 | 5.18E-01 | 7.60E-01 | -5.998 |
| RPL26L1 |  | -0.036 | 10.42 | -0.646 | 5.18E-01 | 7.60E-01 | -5.998 |
| CBR3 |  | -0.045 | 7.629 | -0.646 | 5.19E-01 | 7.60E-01 | -5.999 |
| TAF6L |  | -0.032 | 8.019 | -0.646 | 5.19E-01 | 7.60E-01 | -5.999 |
| ALK |  | 0.025 | 4.816 | 0.645 | 5.19E-01 | 7.60E-01 | -5.999 |
| TAS2R40 |  | -0.036 | 5.115 | -0.645 | 5.19E-01 | 7.60E-01 | -5.999 |
| ATP8A2 |  | 0.045 | 5.752 | 0.644 | 5.19E-01 | 7.60E-01 | -5.999 |
| LRRC29 |  | -0.04 | 5.685 | -0.645 | 5.19E-01 | 7.60E-01 | -5.999 |
| ZNF639 |  | -0.022 | 7.538 | -0.645 | 5.20E-01 | 7.60E-01 | -6 |
| YEATS4 |  | 0.034 | 8.203 | 0.643 | 5.20E-01 | 7.61E-01 | -6 |
| TMPRSS3 |  | -0.05 | 5.264 | -0.643 | 5.20E-01 | 7.61E-01 | -6 |
| REL |  | -0.026 | 7.421 | -0.643 | 5.20E-01 | 7.61E-01 | -6 |
| HIST1H3I |  | 0.046 | 7.259 | 0.642 | 5.20E-01 | 7.61E-01 | -6 |
| CCKAR |  | 0.029 | 4.985 | 0.642 | 5.21E-01 | 7.61E-01 | -6.001 |
| ABCB1 |  | -0.061 | 7.63 | -0.643 | 5.21E-01 | 7.61E-01 | -6.001 |
| GPR88 |  | 0.034 | 5.106 | 0.642 | 5.21E-01 | 7.61E-01 | -6.001 |
| ETFDH |  | -0.032 | 9.417 | -0.642 | 5.21E-01 | 7.62E-01 | -6.001 |
| LDHB |  | -0.053 | 11.856 | -0.642 | 5.21E-01 | 7.62E-01 | -6.001 |
| KCNA6 |  | -0.042 | 5.195 | -0.642 | 5.22E-01 | 7.62E-01 | -6.001 |
| FLRT3 |  | -0.073 | 5.862 | -0.641 | 5.22E-01 | 7.62E-01 | -6.002 |
| FABP3 |  | -0.042 | 5.568 | -0.64 | 5.22E-01 | 7.62E-01 | -6.002 |
| PTPN9 |  | -0.031 | 6.968 | -0.64 | 5.23E-01 | 7.62E-01 | -6.002 |
| LPL |  | 0.077 | 8.044 | 0.639 | 5.23E-01 | 7.62E-01 | -6.002 |
| GCKR |  | 0.031 | 4.887 | 0.639 | 5.23E-01 | 7.62E-01 | -6.002 |
| HPX |  | 0.038 | 5.615 | 0.639 | 5.23E-01 | 7.62E-01 | -6.002 |
| RBM15B |  | 0.025 | 7.722 | 0.639 | 5.23E-01 | 7.62E-01 | -6.003 |
| CXCL9 |  | -0.087 | 6.117 | -0.639 | 5.23E-01 | 7.62E-01 | -6.003 |
| RPS6KA1 |  | 0.033 | 7.699 | 0.638 | 5.23E-01 | 7.62E-01 | -6.003 |
| IL13RA1 |  | 0.029 | 9.754 | 0.638 | 5.23E-01 | 7.62E-01 | -6.003 |
| HOXB6 |  | -0.03 | 7.018 | -0.639 | 5.23E-01 | 7.63E-01 | -6.003 |
| ZNF17 |  | 0.027 | 7.035 | 0.638 | 5.24E-01 | 7.63E-01 | -6.003 |
| YTHDF3 |  | -0.04 | 8.456 | -0.638 | 5.24E-01 | 7.63E-01 | -6.004 |
| STATH |  | -0.034 | 4.73 | -0.638 | 5.24E-01 | 7.63E-01 | -6.004 |
| B3GALT1 |  | 0.033 | 4.943 | 0.637 | 5.24E-01 | 7.63E-01 | -6.004 |
| PEPD |  | -0.035 | 10.208 | -0.637 | 5.24E-01 | 7.63E-01 | -6.004 |
| CILP |  | 0.065 | 6.581 | 0.636 | 5.24E-01 | 7.63E-01 | -6.004 |
| GPC1 |  | 0.037 | 9.325 | 0.636 | 5.25E-01 | 7.63E-01 | -6.004 |
| NCOR1 |  | -0.026 | 7.941 | -0.637 | 5.25E-01 | 7.63E-01 | -6.004 |
| ALS2CR12 |  | -0.037 | 5.186 | -0.637 | 5.25E-01 | 7.63E-01 | -6.005 |
| ARHGEF9 |  | 0.026 | 7.188 | 0.635 | 5.25E-01 | 7.63E-01 | -6.005 |
| ZNF197 |  | 0.03 | 6.961 | 0.635 | 5.25E-01 | 7.63E-01 | -6.005 |
| TYSND1 |  | 0.022 | 7.662 | 0.635 | 5.25E-01 | 7.63E-01 | -6.005 |
| FBXO4 |  | -0.033 | 6.754 | -0.635 | 5.26E-01 | 7.64E-01 | -6.005 |
| HIST1H4D |  | -0.046 | 8.168 | -0.635 | 5.26E-01 | 7.64E-01 | -6.005 |
| TRMT1 |  | -0.036 | 8.94 | -0.635 | 5.26E-01 | 7.64E-01 | -6.005 |
| FBXL20 |  | -0.023 | 8.254 | -0.635 | 5.26E-01 | 7.64E-01 | -6.005 |
| AMICA1 |  | 0.04 | 7.263 | 0.634 | 5.26E-01 | 7.64E-01 | -6.006 |
| MYO9B |  | 0.03 | 8.189 | 0.634 | 5.26E-01 | 7.64E-01 | -6.006 |
| LEPR |  | 0.037 | 6.331 | 0.632 | 5.27E-01 | 7.65E-01 | -6.006 |
| BSG |  | 0.036 | 10.52 | 0.632 | 5.27E-01 | 7.65E-01 | -6.007 |
| CCND1 |  | -0.04 | 9.426 | -0.633 | 5.27E-01 | 7.65E-01 | -6.007 |
| TGM2 |  | -0.074 | 9.844 | -0.633 | 5.27E-01 | 7.65E-01 | -6.007 |
| XPO7 |  | 0.034 | 8.636 | 0.631 | 5.28E-01 | 7.65E-01 | -6.007 |
| PPIL6 |  | -0.034 | 5.202 | -0.631 | 5.28E-01 | 7.66E-01 | -6.008 |
| CACNG6 |  | -0.045 | 5.981 | -0.631 | 5.28E-01 | 7.66E-01 | -6.008 |
| HOXC4 |  | 0.027 | 6.147 | 0.63 | 5.28E-01 | 7.66E-01 | -6.008 |
| ISG20 |  | 0.049 | 10.19 | 0.63 | 5.28E-01 | 7.66E-01 | -6.008 |
| GPR25 |  | 0.046 | 6.898 | 0.63 | 5.29E-01 | 7.66E-01 | -6.008 |
| TLN2 |  | 0.036 | 6.823 | 0.629 | 5.29E-01 | 7.66E-01 | -6.009 |
| TUFM |  | -0.031 | 10.616 | -0.63 | 5.29E-01 | 7.66E-01 | -6.009 |
| KREMEN1 |  | -0.029 | 7.181 | -0.63 | 5.29E-01 | 7.66E-01 | -6.009 |
| WBP11 |  | -0.024 | 8.685 | -0.629 | 5.30E-01 | 7.67E-01 | -6.009 |
| BCL11B |  | -0.032 | 5.692 | -0.629 | 5.30E-01 | 7.67E-01 | -6.009 |
| C21orf59 |  | 0.021 | 9.196 | 0.627 | 5.30E-01 | 7.67E-01 | -6.01 |
| BRAF |  | 0.03 | 7.317 | 0.627 | 5.31E-01 | 7.67E-01 | -6.01 |
| HRAS |  | -0.028 | 8.492 | -0.627 | 5.31E-01 | 7.68E-01 | -6.01 |
| POGZ |  | 0.024 | 8.379 | 0.625 | 5.31E-01 | 7.68E-01 | -6.011 |
| PTX3 |  | -0.08 | 7.333 | -0.626 | 5.32E-01 | 7.68E-01 | -6.011 |
| MARK1 |  | 0.029 | 5.607 | 0.625 | 5.32E-01 | 7.68E-01 | -6.011 |
| SNPH |  | 0.041 | 6.51 | 0.625 | 5.32E-01 | 7.68E-01 | -6.011 |
| ZNF543 |  | 0.029 | 6.375 | 0.625 | 5.32E-01 | 7.68E-01 | -6.011 |
| PQLC1 |  | -0.026 | 9.449 | -0.626 | 5.32E-01 | 7.68E-01 | -6.011 |
| CXADR |  | -0.039 | 5.141 | -0.625 | 5.32E-01 | 7.68E-01 | -6.011 |
| PTCRA |  | 0.031 | 7.485 | 0.624 | 5.32E-01 | 7.68E-01 | -6.011 |
| SLIT1 |  | 0.024 | 5.215 | 0.624 | 5.32E-01 | 7.68E-01 | -6.012 |
| HOXC8 |  | 0.038 | 6.299 | 0.624 | 5.32E-01 | 7.68E-01 | -6.012 |
| TPD52L2 |  | 0.032 | 9.71 | 0.624 | 5.32E-01 | 7.68E-01 | -6.012 |
| INHBE |  | -0.047 | 6.003 | -0.625 | 5.32E-01 | 7.68E-01 | -6.012 |
| RPL22 |  | 0.029 | 10.987 | 0.624 | 5.33E-01 | 7.68E-01 | -6.012 |
| SESTD1 |  | 0.03 | 7.526 | 0.624 | 5.33E-01 | 7.68E-01 | -6.012 |
| MKL1 |  | -0.03 | 7.901 | -0.624 | 5.33E-01 | 7.68E-01 | -6.012 |
| KIAA0922 |  | 0.029 | 6.926 | 0.623 | 5.33E-01 | 7.68E-01 | -6.012 |
| DHCR24 |  | 0.044 | 8.516 | 0.623 | 5.33E-01 | 7.68E-01 | -6.012 |
| PTGES2 |  | 0.029 | 8.287 | 0.622 | 5.34E-01 | 7.68E-01 | -6.013 |
| HTN1 |  | -0.036 | 4.71 | -0.623 | 5.34E-01 | 7.68E-01 | -6.013 |
| PHACTR1 |  | 0.033 | 5.846 | 0.622 | 5.34E-01 | 7.68E-01 | -6.013 |
| CYP1A1 |  | 0.072 | 5.788 | 0.622 | 5.34E-01 | 7.68E-01 | -6.013 |
| UBE2J2 |  | 0.028 | 7.919 | 0.622 | 5.34E-01 | 7.68E-01 | -6.013 |
| ZNF287 |  | 0.038 | 5.619 | 0.621 | 5.34E-01 | 7.68E-01 | -6.013 |
| SLC3A1 |  | 0.034 | 5.094 | 0.621 | 5.34E-01 | 7.68E-01 | -6.013 |
| RAB11FIP4 |  | 0.029 | 7.474 | 0.621 | 5.34E-01 | 7.68E-01 | -6.013 |
| GNG3 |  | 0.039 | 4.956 | 0.621 | 5.34E-01 | 7.68E-01 | -6.013 |
| SYNJ2 |  | 0.031 | 7.22 | 0.62 | 5.35E-01 | 7.70E-01 | -6.014 |
| HTR5A |  | 0.028 | 4.681 | 0.62 | 5.35E-01 | 7.70E-01 | -6.014 |
| GLRA1 |  | 0.035 | 4.833 | 0.618 | 5.36E-01 | 7.71E-01 | -6.015 |
| C22orf23 |  | 0.034 | 5.116 | 0.617 | 5.37E-01 | 7.72E-01 | -6.016 |
| CYP2C19 |  | -0.03 | 4.943 | -0.618 | 5.37E-01 | 7.72E-01 | -6.016 |
| TRIM38 |  | 0.025 | 8.254 | 0.617 | 5.37E-01 | 7.72E-01 | -6.016 |
| LIX1 |  | -0.045 | 5.241 | -0.617 | 5.37E-01 | 7.72E-01 | -6.016 |
| ZBTB17 |  | -0.026 | 8.64 | -0.617 | 5.38E-01 | 7.72E-01 | -6.016 |
| PTAFR |  | 0.03 | 7.866 | 0.616 | 5.38E-01 | 7.72E-01 | -6.016 |
| TBC1D21 |  | -0.039 | 4.834 | -0.617 | 5.38E-01 | 7.72E-01 | -6.017 |
| TFR2 |  | -0.035 | 6.345 | -0.616 | 5.38E-01 | 7.72E-01 | -6.017 |
| MTCH1 |  | -0.019 | 12.372 | -0.616 | 5.38E-01 | 7.72E-01 | -6.017 |
| ASIP |  | -0.034 | 6.182 | -0.615 | 5.39E-01 | 7.73E-01 | -6.017 |
| SLC13A3 |  | 0.039 | 7.174 | 0.614 | 5.39E-01 | 7.73E-01 | -6.018 |
| XRCC3 |  | -0.032 | 7.476 | -0.615 | 5.39E-01 | 7.73E-01 | -6.018 |
| C4BPA |  | -0.065 | 6.2 | -0.614 | 5.39E-01 | 7.73E-01 | -6.018 |
| DCD |  | 0.054 | 4.987 | 0.612 | 5.40E-01 | 7.74E-01 | -6.019 |
| CD79B |  | -0.041 | 6.641 | -0.612 | 5.41E-01 | 7.75E-01 | -6.019 |
| ARVCF |  | 0.035 | 6.322 | 0.611 | 5.41E-01 | 7.75E-01 | -6.019 |
| BRSK1 |  | 0.037 | 5.639 | 0.61 | 5.41E-01 | 7.75E-01 | -6.02 |
| SHOX2 |  | -0.035 | 6.255 | -0.611 | 5.42E-01 | 7.75E-01 | -6.02 |
| SLC30A9 |  | 0.029 | 8.77 | 0.61 | 5.42E-01 | 7.75E-01 | -6.02 |
| HSD17B8 |  | 0.027 | 8.216 | 0.61 | 5.42E-01 | 7.75E-01 | -6.02 |
| PLCD1 |  | 0.029 | 8.108 | 0.61 | 5.42E-01 | 7.76E-01 | -6.02 |
| C1GALT1 |  | -0.038 | 8.641 | -0.61 | 5.42E-01 | 7.76E-01 | -6.02 |
| PHF21B |  | -0.027 | 4.999 | -0.61 | 5.42E-01 | 7.76E-01 | -6.02 |
| TYRP1 |  | 0.03 | 4.679 | 0.608 | 5.43E-01 | 7.76E-01 | -6.021 |
| RPL29 |  | -0.023 | 11.388 | -0.609 | 5.43E-01 | 7.76E-01 | -6.021 |
| PLEKHH2 |  | -0.044 | 5.818 | -0.609 | 5.43E-01 | 7.76E-01 | -6.021 |
| UPK3A |  | 0.035 | 5.498 | 0.608 | 5.43E-01 | 7.76E-01 | -6.021 |
| UCK1 |  | 0.02 | 8.784 | 0.608 | 5.43E-01 | 7.76E-01 | -6.021 |
| VAMP1 |  | 0.034 | 5.89 | 0.607 | 5.44E-01 | 7.77E-01 | -6.022 |
| RAB11A |  | 0.033 | 9.922 | 0.607 | 5.44E-01 | 7.77E-01 | -6.022 |
| SLC35D1 |  | -0.034 | 6.788 | -0.608 | 5.44E-01 | 7.77E-01 | -6.022 |
| GGA2 |  | -0.029 | 8.078 | -0.606 | 5.45E-01 | 7.78E-01 | -6.023 |
| LITAF |  | 0.035 | 11.171 | 0.605 | 5.45E-01 | 7.78E-01 | -6.023 |
| ZNF85 |  | 0.037 | 7.529 | 0.605 | 5.45E-01 | 7.78E-01 | -6.023 |
| SCNN1B |  | -0.054 | 6.907 | -0.606 | 5.45E-01 | 7.78E-01 | -6.023 |
| CCNG1 |  | 0.036 | 9.418 | 0.604 | 5.45E-01 | 7.78E-01 | -6.023 |
| EIF4G2 |  | -0.032 | 12.501 | -0.605 | 5.45E-01 | 7.78E-01 | -6.023 |
| XPA |  | 0.021 | 7.91 | 0.604 | 5.45E-01 | 7.78E-01 | -6.023 |
| AP3B2 |  | 0.047 | 5.708 | 0.604 | 5.45E-01 | 7.78E-01 | -6.023 |
| TTC12 |  | 0.032 | 6.909 | 0.604 | 5.46E-01 | 7.78E-01 | -6.023 |
| LZTR1 |  | 0.023 | 9.356 | 0.602 | 5.47E-01 | 7.79E-01 | -6.025 |
| CD59 |  | -0.033 | 10.308 | -0.603 | 5.47E-01 | 7.79E-01 | -6.025 |
| ETV7 |  | -0.055 | 6.693 | -0.602 | 5.47E-01 | 7.79E-01 | -6.025 |
| OR1D2 |  | 0.036 | 5.827 | 0.601 | 5.47E-01 | 7.79E-01 | -6.025 |
| ATXN10 |  | 0.036 | 8.635 | 0.601 | 5.48E-01 | 7.79E-01 | -6.025 |
| DONSON |  | 0.021 | 7.921 | 0.601 | 5.48E-01 | 7.79E-01 | -6.025 |
| RIPK3 |  | 0.026 | 7.055 | 0.601 | 5.48E-01 | 7.79E-01 | -6.025 |
| C10orf107 |  | 0.049 | 5.25 | 0.6 | 5.48E-01 | 7.79E-01 | -6.025 |
| MICB |  | -0.031 | 7.946 | -0.601 | 5.48E-01 | 7.79E-01 | -6.026 |
| PLA2G2A |  | 0.052 | 8.787 | 0.6 | 5.48E-01 | 7.79E-01 | -6.026 |
| PPP5C |  | -0.031 | 6.865 | -0.601 | 5.48E-01 | 7.79E-01 | -6.026 |
| ANP32A |  | 0.022 | 8.761 | 0.6 | 5.48E-01 | 7.79E-01 | -6.026 |
| AGT |  | 0.045 | 5.665 | 0.6 | 5.48E-01 | 7.79E-01 | -6.026 |
| ADAMTS13 |  | -0.025 | 6.282 | -0.6 | 5.49E-01 | 7.79E-01 | -6.026 |
| STAG2 |  | 0.032 | 9.107 | 0.599 | 5.49E-01 | 7.79E-01 | -6.026 |
| THRA |  | 0.028 | 7.628 | 0.599 | 5.49E-01 | 7.79E-01 | -6.026 |
| PKD2L1 |  | -0.039 | 5.546 | -0.6 | 5.49E-01 | 7.79E-01 | -6.026 |
| GABRB3 |  | -0.033 | 4.926 | -0.6 | 5.49E-01 | 7.79E-01 | -6.026 |
| ELAVL3 |  | 0.029 | 5.311 | 0.599 | 5.49E-01 | 7.79E-01 | -6.026 |
| WISP1 |  | 0.029 | 4.899 | 0.599 | 5.49E-01 | 7.79E-01 | -6.026 |
| CACNB1 |  | 0.027 | 5.695 | 0.598 | 5.49E-01 | 7.79E-01 | -6.027 |
| TSGA10IP |  | -0.038 | 5.449 | -0.599 | 5.49E-01 | 7.79E-01 | -6.027 |
| RAP2A |  | -0.042 | 8.358 | -0.599 | 5.50E-01 | 7.79E-01 | -6.027 |
| TPMT |  | 0.029 | 7.379 | 0.598 | 5.50E-01 | 7.79E-01 | -6.027 |
| UTF1 |  | -0.036 | 7.657 | -0.599 | 5.50E-01 | 7.79E-01 | -6.027 |
| PTGER3 |  | 0.03 | 5.224 | 0.597 | 5.50E-01 | 7.79E-01 | -6.027 |
| ANKH |  | 0.03 | 6.924 | 0.597 | 5.50E-01 | 7.79E-01 | -6.027 |
| CDKN1C |  | -0.05 | 11.468 | -0.598 | 5.50E-01 | 7.79E-01 | -6.027 |
| SP4 |  | 0.029 | 6.492 | 0.597 | 5.50E-01 | 7.79E-01 | -6.027 |
| SCT |  | 0.026 | 6.568 | 0.597 | 5.50E-01 | 7.79E-01 | -6.027 |
| SMO |  | -0.036 | 7.316 | -0.598 | 5.50E-01 | 7.79E-01 | -6.027 |
| SOX3 |  | 0.04 | 5.882 | 0.597 | 5.50E-01 | 7.79E-01 | -6.027 |
| ARMC6 |  | -0.031 | 8.152 | -0.598 | 5.51E-01 | 7.79E-01 | -6.028 |
| NETO2 |  | -0.04 | 7.087 | -0.597 | 5.51E-01 | 7.79E-01 | -6.028 |
| IGSF1 |  | -0.035 | 5.399 | -0.597 | 5.51E-01 | 7.79E-01 | -6.028 |
| TRIM42 |  | -0.036 | 5.082 | -0.597 | 5.51E-01 | 7.79E-01 | -6.028 |
| IL31RA |  | -0.032 | 5.117 | -0.597 | 5.51E-01 | 7.79E-01 | -6.028 |
| NR5A1 |  | -0.041 | 5.576 | -0.597 | 5.51E-01 | 7.79E-01 | -6.028 |
| ZBED3 |  | 0.037 | 7.719 | 0.596 | 5.51E-01 | 7.79E-01 | -6.028 |
| RUNX3 |  | -0.04 | 6.725 | -0.596 | 5.52E-01 | 7.79E-01 | -6.029 |
| ZDHHC15 |  | 0.027 | 4.932 | 0.595 | 5.52E-01 | 7.79E-01 | -6.029 |
| USP38 |  | -0.036 | 8.691 | -0.596 | 5.52E-01 | 7.79E-01 | -6.029 |
| DNM3 |  | 0.033 | 5.935 | 0.595 | 5.52E-01 | 7.79E-01 | -6.029 |
| FMO4 |  | -0.027 | 6.63 | -0.596 | 5.52E-01 | 7.79E-01 | -6.029 |
| NKAP |  | 0.033 | 8.357 | 0.595 | 5.52E-01 | 7.79E-01 | -6.029 |
| SFXN1 |  | 0.029 | 7.102 | 0.594 | 5.52E-01 | 7.79E-01 | -6.029 |
| SCARF1 |  | -0.032 | 7.349 | -0.595 | 5.52E-01 | 7.79E-01 | -6.029 |
| QPCTL |  | 0.035 | 5.685 | 0.594 | 5.52E-01 | 7.79E-01 | -6.029 |
| EEF1D |  | 0.02 | 11.432 | 0.594 | 5.52E-01 | 7.79E-01 | -6.029 |
| ZNF195 |  | 0.035 | 8.039 | 0.594 | 5.52E-01 | 7.79E-01 | -6.029 |
| CSNK2A1 |  | -0.024 | 8.274 | -0.595 | 5.52E-01 | 7.79E-01 | -6.029 |
| PIGR |  | -0.036 | 5.071 | -0.594 | 5.53E-01 | 7.79E-01 | -6.029 |
| DHX40 |  | -0.029 | 6.775 | -0.594 | 5.53E-01 | 7.79E-01 | -6.03 |
| ANXA6 |  | 0.039 | 9.037 | 0.593 | 5.53E-01 | 7.79E-01 | -6.03 |
| FLOT2 |  | 0.034 | 9.79 | 0.593 | 5.53E-01 | 7.79E-01 | -6.03 |
| HAO2 |  | 0.029 | 4.731 | 0.593 | 5.53E-01 | 7.79E-01 | -6.03 |
| ACAT2 |  | 0.029 | 8.845 | 0.593 | 5.53E-01 | 7.79E-01 | -6.03 |
| VGLL1 |  | 0.029 | 8.486 | 0.592 | 5.53E-01 | 7.79E-01 | -6.03 |
| ERG |  | 0.032 | 6.266 | 0.592 | 5.54E-01 | 7.79E-01 | -6.03 |
| ZFYVE27 |  | 0.024 | 8.746 | 0.592 | 5.54E-01 | 7.79E-01 | -6.03 |
| LZTS1 |  | -0.037 | 6.987 | -0.593 | 5.54E-01 | 7.79E-01 | -6.03 |
| CA14 |  | 0.035 | 6.21 | 0.592 | 5.54E-01 | 7.79E-01 | -6.03 |
| SHPRH |  | 0.027 | 6.743 | 0.592 | 5.54E-01 | 7.79E-01 | -6.03 |
| PRRG3 |  | -0.035 | 5.591 | -0.592 | 5.54E-01 | 7.80E-01 | -6.031 |
| RFX2 |  | 0.031 | 6.926 | 0.591 | 5.54E-01 | 7.80E-01 | -6.031 |
| LGI3 |  | 0.032 | 6.227 | 0.591 | 5.54E-01 | 7.80E-01 | -6.031 |
| GFOD1 |  | -0.03 | 8.282 | -0.591 | 5.55E-01 | 7.80E-01 | -6.031 |
| SEPP1 |  | -0.044 | 9.937 | -0.591 | 5.55E-01 | 7.80E-01 | -6.031 |
| DMRT2 |  | -0.038 | 4.823 | -0.59 | 5.55E-01 | 7.81E-01 | -6.032 |
| PTPRZ1 |  | 0.029 | 4.941 | 0.589 | 5.56E-01 | 7.81E-01 | -6.032 |
| BUB3 |  | -0.019 | 9.685 | -0.59 | 5.56E-01 | 7.81E-01 | -6.032 |
| BBS2 |  | 0.025 | 9.009 | 0.589 | 5.56E-01 | 7.81E-01 | -6.032 |
| SNCB |  | -0.041 | 6.816 | -0.589 | 5.56E-01 | 7.81E-01 | -6.032 |
| UAP1L1 |  | -0.032 | 7.422 | -0.589 | 5.56E-01 | 7.81E-01 | -6.033 |
| ZCCHC12 |  | -0.034 | 5.133 | -0.589 | 5.56E-01 | 7.81E-01 | -6.033 |
| HAX1 |  | 0.019 | 9.987 | 0.587 | 5.57E-01 | 7.81E-01 | -6.033 |
| ZFR |  | 0.022 | 9.718 | 0.587 | 5.57E-01 | 7.81E-01 | -6.033 |
| FGF21 |  | -0.035 | 4.969 | -0.588 | 5.57E-01 | 7.81E-01 | -6.033 |
| IQCF1 |  | -0.028 | 4.821 | -0.588 | 5.57E-01 | 7.81E-01 | -6.033 |
| BAMBI |  | 0.04 | 10.163 | 0.587 | 5.57E-01 | 7.81E-01 | -6.033 |
| POP5 |  | 0.023 | 8.804 | 0.587 | 5.57E-01 | 7.81E-01 | -6.033 |
| CR2 |  | 0.033 | 5.898 | 0.587 | 5.57E-01 | 7.81E-01 | -6.033 |
| PRX |  | 0.026 | 6.338 | 0.586 | 5.58E-01 | 7.82E-01 | -6.034 |
| TRIM44 |  | 0.024 | 9.209 | 0.586 | 5.58E-01 | 7.82E-01 | -6.034 |
| TRPV4 |  | 0.029 | 7.057 | 0.586 | 5.58E-01 | 7.82E-01 | -6.034 |
| MTO1 |  | -0.02 | 8.085 | -0.586 | 5.58E-01 | 7.82E-01 | -6.034 |
| SMAD5 |  | 0.038 | 7.377 | 0.585 | 5.58E-01 | 7.82E-01 | -6.034 |
| CUEDC2 |  | -0.021 | 9.733 | -0.586 | 5.58E-01 | 7.82E-01 | -6.034 |
| GEMIN6 |  | 0.03 | 8.943 | 0.584 | 5.59E-01 | 7.83E-01 | -6.035 |
| HDAC6 |  | 0.022 | 7.922 | 0.583 | 5.59E-01 | 7.83E-01 | -6.035 |
| NANOG |  | 0.036 | 5.618 | 0.583 | 5.60E-01 | 7.83E-01 | -6.035 |
| CLIC1 |  | 0.018 | 12.208 | 0.583 | 5.60E-01 | 7.83E-01 | -6.035 |
| MAGEE1 |  | 0.026 | 5.919 | 0.582 | 5.60E-01 | 7.84E-01 | -6.036 |
| GSTO2 |  | 0.038 | 5.922 | 0.582 | 5.60E-01 | 7.84E-01 | -6.036 |
| CYP7B1 |  | -0.046 | 5.728 | -0.582 | 5.61E-01 | 7.84E-01 | -6.036 |
| PDCD6 |  | 0.018 | 11.183 | 0.581 | 5.61E-01 | 7.84E-01 | -6.037 |
| EPB41L5 |  | -0.033 | 6.506 | -0.582 | 5.61E-01 | 7.84E-01 | -6.037 |
| M6PR |  | -0.023 | 9.738 | -0.581 | 5.61E-01 | 7.85E-01 | -6.037 |
| PIGK |  | 0.029 | 7.956 | 0.58 | 5.62E-01 | 7.85E-01 | -6.037 |
| MDM4 |  | 0.029 | 7.227 | 0.58 | 5.62E-01 | 7.85E-01 | -6.037 |
| LAT |  | 0.025 | 7.917 | 0.579 | 5.62E-01 | 7.85E-01 | -6.037 |
| HDHD2 |  | 0.023 | 9.664 | 0.579 | 5.62E-01 | 7.85E-01 | -6.038 |
| IL10RB |  | 0.028 | 10.143 | 0.578 | 5.63E-01 | 7.86E-01 | -6.038 |
| GRM3 |  | 0.03 | 4.613 | 0.578 | 5.63E-01 | 7.86E-01 | -6.038 |
| KCNA4 |  | 0.04 | 5.192 | 0.578 | 5.63E-01 | 7.86E-01 | -6.038 |
| IFIT3 |  | -0.047 | 9.157 | -0.578 | 5.64E-01 | 7.87E-01 | -6.039 |
| MED12 |  | -0.031 | 8.214 | -0.576 | 5.65E-01 | 7.88E-01 | -6.04 |
| NAB2 |  | -0.034 | 7.814 | -0.576 | 5.65E-01 | 7.88E-01 | -6.04 |
| ZNF619 |  | 0.027 | 5.871 | 0.575 | 5.65E-01 | 7.88E-01 | -6.04 |
| CNNM3 |  | 0.021 | 8.497 | 0.574 | 5.65E-01 | 7.88E-01 | -6.04 |
| MOGAT1 |  | 0.037 | 4.901 | 0.574 | 5.65E-01 | 7.88E-01 | -6.04 |
| ITGB8 |  | 0.034 | 6.003 | 0.573 | 5.66E-01 | 7.89E-01 | -6.041 |
| FAM24B |  | 0.035 | 6.247 | 0.573 | 5.66E-01 | 7.89E-01 | -6.041 |
| SP7 |  | 0.029 | 4.973 | 0.573 | 5.67E-01 | 7.89E-01 | -6.041 |
| RNASE1 |  | -0.033 | 10.249 | -0.574 | 5.67E-01 | 7.89E-01 | -6.041 |
| RAB28 |  | -0.028 | 7.675 | -0.573 | 5.67E-01 | 7.89E-01 | -6.041 |
| USP11 |  | -0.028 | 9.09 | -0.573 | 5.67E-01 | 7.89E-01 | -6.041 |
| SERPINB6 |  | 0.03 | 9.858 | 0.572 | 5.67E-01 | 7.89E-01 | -6.041 |
| KLF16 |  | 0.031 | 7.781 | 0.572 | 5.67E-01 | 7.89E-01 | -6.042 |
| NDRG4 |  | -0.039 | 6.98 | -0.572 | 5.67E-01 | 7.89E-01 | -6.042 |
| MLLT6 |  | 0.03 | 8.045 | 0.571 | 5.68E-01 | 7.89E-01 | -6.042 |
| SORBS1 |  | -0.028 | 7.501 | -0.572 | 5.68E-01 | 7.89E-01 | -6.042 |
| IMPACT |  | -0.022 | 7.617 | -0.572 | 5.68E-01 | 7.89E-01 | -6.042 |
| RXRB |  | -0.024 | 9.376 | -0.572 | 5.68E-01 | 7.89E-01 | -6.042 |
| KCNK7 |  | 0.037 | 7.298 | 0.571 | 5.68E-01 | 7.89E-01 | -6.042 |
| SLC31A1 |  | -0.026 | 8.466 | -0.571 | 5.68E-01 | 7.90E-01 | -6.042 |
| PSCA |  | 0.061 | 6.719 | 0.57 | 5.68E-01 | 7.90E-01 | -6.043 |
| P2RX2 |  | -0.038 | 7.406 | -0.57 | 5.69E-01 | 7.90E-01 | -6.043 |
| BTBD7 |  | -0.02 | 7.862 | -0.57 | 5.69E-01 | 7.90E-01 | -6.043 |
| SCN9A |  | -0.038 | 5.978 | -0.57 | 5.69E-01 | 7.90E-01 | -6.043 |
| ITPK1 |  | -0.029 | 7.991 | -0.569 | 5.69E-01 | 7.90E-01 | -6.044 |
| KCNA7 |  | -0.033 | 4.702 | -0.569 | 5.70E-01 | 7.90E-01 | -6.044 |
| ZNF334 |  | 0.032 | 5.704 | 0.568 | 5.70E-01 | 7.90E-01 | -6.044 |
| FGF22 |  | 0.03 | 5.132 | 0.568 | 5.70E-01 | 7.90E-01 | -6.044 |
| BRD4 |  | -0.027 | 7.463 | -0.569 | 5.70E-01 | 7.90E-01 | -6.044 |
| CDC42SE2 |  | -0.03 | 7.908 | -0.568 | 5.70E-01 | 7.90E-01 | -6.044 |
| PRL |  | 0.101 | 6.871 | 0.567 | 5.70E-01 | 7.90E-01 | -6.044 |
| CD226 |  | 0.04 | 5.375 | 0.567 | 5.70E-01 | 7.90E-01 | -6.044 |
| CDC16 |  | 0.02 | 10.705 | 0.567 | 5.70E-01 | 7.90E-01 | -6.044 |
| PACSIN1 |  | -0.037 | 6.034 | -0.568 | 5.70E-01 | 7.91E-01 | -6.044 |
| GCA |  | -0.033 | 10.011 | -0.568 | 5.71E-01 | 7.91E-01 | -6.044 |
| EPHX1 |  | -0.037 | 7.472 | -0.568 | 5.71E-01 | 7.91E-01 | -6.044 |
| SDAD1 |  | -0.034 | 8.447 | -0.567 | 5.71E-01 | 7.91E-01 | -6.045 |
| ABCA8 |  | -0.043 | 6.386 | -0.567 | 5.71E-01 | 7.91E-01 | -6.045 |
| IGSF10 |  | -0.047 | 5.683 | -0.566 | 5.72E-01 | 7.91E-01 | -6.045 |
| PRKG1 |  | 0.025 | 5.788 | 0.565 | 5.72E-01 | 7.91E-01 | -6.046 |
| FOXA3 |  | 0.032 | 5.521 | 0.565 | 5.72E-01 | 7.91E-01 | -6.046 |
| MYH11 |  | -0.046 | 8.854 | -0.565 | 5.72E-01 | 7.91E-01 | -6.046 |
| BARX2 |  | -0.03 | 5.488 | -0.565 | 5.72E-01 | 7.91E-01 | -6.046 |
| AGTR1 |  | -0.034 | 9.098 | -0.565 | 5.72E-01 | 7.91E-01 | -6.046 |
| ARG1 |  | 0.048 | 6.139 | 0.564 | 5.72E-01 | 7.91E-01 | -6.046 |
| MAF1 |  | -0.029 | 9.358 | -0.565 | 5.72E-01 | 7.91E-01 | -6.046 |
| PIAS2 |  | -0.025 | 6.843 | -0.564 | 5.73E-01 | 7.92E-01 | -6.046 |
| ATP2B4 |  | 0.028 | 8.112 | 0.563 | 5.73E-01 | 7.92E-01 | -6.046 |
| LCN6 |  | -0.027 | 5.137 | -0.563 | 5.74E-01 | 7.93E-01 | -6.047 |
| KRT8 |  | 0.03 | 10 | 0.562 | 5.74E-01 | 7.93E-01 | -6.047 |
| DNAH9 |  | 0.036 | 5.156 | 0.562 | 5.74E-01 | 7.93E-01 | -6.047 |
| KCNJ9 |  | 0.025 | 5.443 | 0.562 | 5.74E-01 | 7.93E-01 | -6.047 |
| PKIG |  | 0.023 | 9.254 | 0.561 | 5.74E-01 | 7.93E-01 | -6.047 |
| WDR31 |  | 0.031 | 5.08 | 0.561 | 5.74E-01 | 7.93E-01 | -6.047 |
| TDRKH |  | -0.029 | 6.398 | -0.562 | 5.75E-01 | 7.93E-01 | -6.048 |
| FCN2 |  | -0.027 | 5.391 | -0.561 | 5.75E-01 | 7.94E-01 | -6.048 |
| CLTB |  | 0.026 | 8.482 | 0.56 | 5.75E-01 | 7.94E-01 | -6.048 |
| VTI1B |  | 0.031 | 10.403 | 0.559 | 5.75E-01 | 7.94E-01 | -6.048 |
| USP14 |  | 0.035 | 8.127 | 0.559 | 5.76E-01 | 7.94E-01 | -6.048 |
| GGN |  | -0.026 | 6.514 | -0.56 | 5.76E-01 | 7.94E-01 | -6.049 |
| HIST1H2BA |  | -0.029 | 4.897 | -0.56 | 5.76E-01 | 7.94E-01 | -6.049 |
| AVPR1B |  | -0.042 | 4.919 | -0.559 | 5.76E-01 | 7.94E-01 | -6.049 |
| FUCA2 |  | -0.027 | 9.652 | -0.559 | 5.76E-01 | 7.94E-01 | -6.049 |
| MNAT1 |  | 0.019 | 8.655 | 0.558 | 5.77E-01 | 7.95E-01 | -6.049 |
| ATAD1 |  | -0.035 | 9.656 | -0.558 | 5.77E-01 | 7.95E-01 | -6.049 |
| CAMK1D |  | 0.022 | 7.332 | 0.557 | 5.77E-01 | 7.95E-01 | -6.05 |
| SPPL2B |  | -0.027 | 7.812 | -0.558 | 5.77E-01 | 7.95E-01 | -6.05 |
| ARHGAP24 |  | -0.031 | 7.015 | -0.558 | 5.77E-01 | 7.95E-01 | -6.05 |
| ADD3 |  | -0.04 | 8.785 | -0.557 | 5.78E-01 | 7.95E-01 | -6.05 |
| SLC9A3R1 |  | 0.026 | 10.396 | 0.556 | 5.78E-01 | 7.95E-01 | -6.05 |
| GTPBP1 |  | 0.022 | 7.802 | 0.556 | 5.78E-01 | 7.95E-01 | -6.05 |
| PAPOLG |  | 0.025 | 6.998 | 0.555 | 5.78E-01 | 7.95E-01 | -6.05 |
| ATP6V0B |  | -0.026 | 11.706 | -0.556 | 5.79E-01 | 7.96E-01 | -6.051 |
| MAOA |  | 0.046 | 10.671 | 0.555 | 5.79E-01 | 7.96E-01 | -6.051 |
| NRG4 |  | 0.031 | 5.166 | 0.554 | 5.79E-01 | 7.96E-01 | -6.051 |
| DEFB119 |  | -0.025 | 4.762 | -0.555 | 5.79E-01 | 7.96E-01 | -6.051 |
| GZMH |  | 0.051 | 6.944 | 0.554 | 5.79E-01 | 7.96E-01 | -6.051 |
| ZNF142 |  | -0.027 | 7.775 | -0.555 | 5.79E-01 | 7.96E-01 | -6.051 |
| NFKBIL1 |  | -0.033 | 7.827 | -0.554 | 5.80E-01 | 7.96E-01 | -6.052 |
| MFNG |  | -0.022 | 9.488 | -0.554 | 5.80E-01 | 7.96E-01 | -6.052 |
| SP3 |  | -0.029 | 8.896 | -0.554 | 5.80E-01 | 7.96E-01 | -6.052 |
| AKR7A2 |  | -0.02 | 10.564 | -0.554 | 5.80E-01 | 7.96E-01 | -6.052 |
| ZPBP |  | -0.031 | 4.932 | -0.553 | 5.80E-01 | 7.96E-01 | -6.052 |
| PPP1R3F |  | -0.026 | 6.82 | -0.553 | 5.80E-01 | 7.96E-01 | -6.052 |
| RHOBTB3 |  | 0.048 | 9.98 | 0.552 | 5.81E-01 | 7.96E-01 | -6.052 |
| CPNE6 |  | -0.032 | 5.427 | -0.553 | 5.81E-01 | 7.96E-01 | -6.052 |
| MRPL19 |  | -0.029 | 9.122 | -0.553 | 5.81E-01 | 7.96E-01 | -6.052 |
| RASIP1 |  | -0.035 | 9.173 | -0.553 | 5.81E-01 | 7.96E-01 | -6.052 |
| ZNF408 |  | 0.032 | 6.681 | 0.551 | 5.81E-01 | 7.97E-01 | -6.053 |
| DDX17 |  | -0.038 | 9.632 | -0.552 | 5.81E-01 | 7.97E-01 | -6.053 |
| LHCGR |  | -0.028 | 4.691 | -0.552 | 5.82E-01 | 7.97E-01 | -6.053 |
| POU4F3 |  | -0.032 | 4.786 | -0.551 | 5.82E-01 | 7.97E-01 | -6.053 |
| GPR78 |  | -0.041 | 7.223 | -0.551 | 5.82E-01 | 7.97E-01 | -6.053 |
| HBZ |  | 0.063 | 7.33 | 0.55 | 5.82E-01 | 7.97E-01 | -6.053 |
| ITGB1BP1 |  | 0.024 | 9.189 | 0.549 | 5.83E-01 | 7.97E-01 | -6.054 |
| RGS14 |  | 0.029 | 6.488 | 0.549 | 5.83E-01 | 7.97E-01 | -6.054 |
| RND1 |  | 0.036 | 5.049 | 0.549 | 5.83E-01 | 7.97E-01 | -6.054 |
| SELENBP1 |  | -0.04 | 9.251 | -0.55 | 5.83E-01 | 7.97E-01 | -6.054 |
| HOMER1 |  | -0.032 | 7.358 | -0.55 | 5.83E-01 | 7.97E-01 | -6.054 |
| TUBGCP2 |  | -0.022 | 8.809 | -0.55 | 5.83E-01 | 7.97E-01 | -6.054 |
| COG4 |  | 0.022 | 8.392 | 0.548 | 5.83E-01 | 7.97E-01 | -6.054 |
| CH25H |  | 0.043 | 6.061 | 0.548 | 5.83E-01 | 7.97E-01 | -6.054 |
| SEMA3E |  | -0.044 | 4.774 | -0.549 | 5.83E-01 | 7.97E-01 | -6.054 |
| LIPC |  | -0.036 | 5.443 | -0.548 | 5.84E-01 | 7.98E-01 | -6.055 |
| ALDH1B1 |  | -0.028 | 6.635 | -0.548 | 5.84E-01 | 7.98E-01 | -6.055 |
| PYGB |  | 0.023 | 8.892 | 0.546 | 5.85E-01 | 7.99E-01 | -6.055 |
| CPN1 |  | -0.036 | 5.78 | -0.547 | 5.85E-01 | 7.99E-01 | -6.056 |
| ZNF491 |  | -0.03 | 6.08 | -0.546 | 5.85E-01 | 7.99E-01 | -6.056 |
| PITX2 |  | 0.035 | 7.878 | 0.545 | 5.85E-01 | 7.99E-01 | -6.056 |
| CENPB |  | 0.025 | 9.506 | 0.545 | 5.85E-01 | 7.99E-01 | -6.056 |
| RBM22 |  | -0.028 | 10.322 | -0.546 | 5.85E-01 | 7.99E-01 | -6.056 |
| ZNF395 |  | 0.029 | 9.893 | 0.544 | 5.86E-01 | 8.00E-01 | -6.056 |
| NLGN4X |  | -0.05 | 6.273 | -0.545 | 5.86E-01 | 8.00E-01 | -6.057 |
| NKX2-2 |  | 0.029 | 4.661 | 0.544 | 5.86E-01 | 8.00E-01 | -6.057 |
| IDE |  | -0.019 | 7.98 | -0.544 | 5.86E-01 | 8.00E-01 | -6.057 |
| TOLLIP |  | 0.022 | 8.124 | 0.543 | 5.87E-01 | 8.00E-01 | -6.057 |
| USH2A |  | -0.025 | 4.763 | -0.543 | 5.87E-01 | 8.01E-01 | -6.058 |
| BCORL1 |  | 0.027 | 7.877 | 0.542 | 5.88E-01 | 8.01E-01 | -6.058 |
| NDUFAF1 |  | 0.022 | 9.008 | 0.541 | 5.88E-01 | 8.01E-01 | -6.058 |
| ARHGDIA |  | 0.032 | 9.35 | 0.54 | 5.89E-01 | 8.02E-01 | -6.059 |
| PNLIP |  | 0.029 | 4.539 | 0.539 | 5.90E-01 | 8.03E-01 | -6.059 |
| CYP2R1 |  | -0.03 | 7.51 | -0.539 | 5.90E-01 | 8.03E-01 | -6.059 |
| CD248 |  | -0.045 | 10.095 | -0.539 | 5.90E-01 | 8.03E-01 | -6.059 |
| CNOT8 |  | 0.025 | 9.171 | 0.538 | 5.90E-01 | 8.03E-01 | -6.06 |
| TBX2 |  | -0.041 | 8.637 | -0.539 | 5.90E-01 | 8.03E-01 | -6.06 |
| SYAP1 |  | 0.026 | 9.371 | 0.538 | 5.90E-01 | 8.03E-01 | -6.06 |
| PTPRH |  | 0.035 | 5.968 | 0.538 | 5.91E-01 | 8.03E-01 | -6.06 |
| GNAO1 |  | 0.026 | 5.208 | 0.537 | 5.91E-01 | 8.03E-01 | -6.06 |
| RNF166 |  | 0.025 | 6.814 | 0.537 | 5.91E-01 | 8.04E-01 | -6.06 |
| SLC24A1 |  | -0.025 | 6.81 | -0.538 | 5.91E-01 | 8.04E-01 | -6.06 |
| SPG7 |  | 0.021 | 7.607 | 0.537 | 5.91E-01 | 8.04E-01 | -6.06 |
| P2RY14 |  | -0.049 | 8.188 | -0.538 | 5.91E-01 | 8.04E-01 | -6.06 |
| TAOK1 |  | 0.028 | 8.462 | 0.536 | 5.91E-01 | 8.04E-01 | -6.061 |
| PPP2CA |  | -0.025 | 11.318 | -0.537 | 5.91E-01 | 8.04E-01 | -6.061 |
| PJA1 |  | 0.022 | 9.224 | 0.536 | 5.91E-01 | 8.04E-01 | -6.061 |
| RHOQ |  | 0.02 | 9.432 | 0.535 | 5.92E-01 | 8.04E-01 | -6.061 |
| INPP5A |  | 0.03 | 7.495 | 0.535 | 5.92E-01 | 8.04E-01 | -6.061 |
| HIST1H2BK |  | -0.027 | 11.365 | -0.536 | 5.92E-01 | 8.04E-01 | -6.061 |
| TIMM17B |  | 0.02 | 9.297 | 0.535 | 5.92E-01 | 8.04E-01 | -6.061 |
| SLC22A11 |  | -0.027 | 8.821 | -0.536 | 5.93E-01 | 8.04E-01 | -6.061 |
| KIFC2 |  | 0.031 | 7.663 | 0.534 | 5.93E-01 | 8.04E-01 | -6.062 |
| NR2E1 |  | -0.029 | 5.205 | -0.534 | 5.93E-01 | 8.05E-01 | -6.062 |
| FBLN1 |  | -0.045 | 10.344 | -0.534 | 5.93E-01 | 8.05E-01 | -6.062 |
| HOXA6 |  | 0.037 | 5.91 | 0.533 | 5.94E-01 | 8.05E-01 | -6.062 |
| CCL16 |  | -0.032 | 5.375 | -0.534 | 5.94E-01 | 8.05E-01 | -6.062 |
| YWHAZ |  | -0.019 | 11.234 | -0.533 | 5.94E-01 | 8.05E-01 | -6.063 |
| F8 |  | 0.03 | 6.254 | 0.532 | 5.94E-01 | 8.05E-01 | -6.063 |
| TIMM10 |  | 0.028 | 9.741 | 0.532 | 5.94E-01 | 8.05E-01 | -6.063 |
| MCF2L |  | 0.024 | 5.717 | 0.532 | 5.94E-01 | 8.05E-01 | -6.063 |
| CREM |  | 0.021 | 7.494 | 0.532 | 5.94E-01 | 8.05E-01 | -6.063 |
| PROL1 |  | 0.032 | 5.079 | 0.531 | 5.95E-01 | 8.06E-01 | -6.063 |
| SLC16A11 |  | -0.029 | 7.464 | -0.532 | 5.95E-01 | 8.06E-01 | -6.063 |
| FN3K |  | 0.029 | 5.635 | 0.531 | 5.95E-01 | 8.06E-01 | -6.063 |
| TGFB1I1 |  | -0.047 | 8.164 | -0.532 | 5.95E-01 | 8.06E-01 | -6.063 |
| SEZ6L |  | 0.031 | 5.064 | 0.531 | 5.95E-01 | 8.06E-01 | -6.063 |
| ARNTL2 |  | -0.031 | 6.375 | -0.531 | 5.96E-01 | 8.06E-01 | -6.064 |
| DUSP19 |  | 0.025 | 5.901 | 0.53 | 5.96E-01 | 8.06E-01 | -6.064 |
| ETV3 |  | -0.024 | 6.906 | -0.53 | 5.96E-01 | 8.06E-01 | -6.064 |
| CACNA2D1 |  | 0.026 | 5.053 | 0.529 | 5.96E-01 | 8.07E-01 | -6.064 |
| MAP1LC3A |  | 0.03 | 8.019 | 0.529 | 5.97E-01 | 8.07E-01 | -6.064 |
| DHRS3 |  | 0.033 | 10.356 | 0.528 | 5.97E-01 | 8.07E-01 | -6.065 |
| SCUBE3 |  | 0.031 | 5.361 | 0.528 | 5.97E-01 | 8.07E-01 | -6.065 |
| GYPA |  | 0.049 | 5.958 | 0.528 | 5.97E-01 | 8.07E-01 | -6.065 |
| KCTD15 |  | 0.024 | 7.048 | 0.528 | 5.97E-01 | 8.07E-01 | -6.065 |
| OAS2 |  | -0.039 | 8.02 | -0.528 | 5.98E-01 | 8.07E-01 | -6.065 |
| LTBP4 |  | -0.027 | 7.967 | -0.528 | 5.98E-01 | 8.07E-01 | -6.065 |
| RHBG |  | -0.029 | 5.335 | -0.528 | 5.98E-01 | 8.07E-01 | -6.066 |
| PPAT |  | -0.024 | 8.396 | -0.526 | 5.99E-01 | 8.09E-01 | -6.066 |
| NMD3 |  | 0.032 | 8.47 | 0.525 | 5.99E-01 | 8.09E-01 | -6.066 |
| INPP5F |  | 0.018 | 7.722 | 0.524 | 6.00E-01 | 8.09E-01 | -6.067 |
| BICC1 |  | -0.031 | 5.416 | -0.525 | 6.00E-01 | 8.09E-01 | -6.067 |
| RELN |  | 0.029 | 4.863 | 0.524 | 6.00E-01 | 8.09E-01 | -6.067 |
| RAB18 |  | 0.026 | 8.893 | 0.523 | 6.01E-01 | 8.10E-01 | -6.067 |
| DACH2 |  | -0.026 | 4.758 | -0.524 | 6.01E-01 | 8.10E-01 | -6.067 |
| ZNF519 |  | -0.031 | 5.453 | -0.524 | 6.01E-01 | 8.10E-01 | -6.067 |
| FOXP4 |  | -0.035 | 6.719 | -0.523 | 6.01E-01 | 8.10E-01 | -6.068 |
| NFE2 |  | -0.032 | 8.824 | -0.523 | 6.01E-01 | 8.10E-01 | -6.068 |
| DPYSL5 |  | -0.021 | 5.245 | -0.523 | 6.01E-01 | 8.10E-01 | -6.068 |
| HSPB1 |  | 0.026 | 13.455 | 0.522 | 6.01E-01 | 8.10E-01 | -6.068 |
| SNTB1 |  | -0.036 | 7.073 | -0.522 | 6.02E-01 | 8.10E-01 | -6.068 |
| CLUAP1 |  | 0.019 | 8.021 | 0.521 | 6.02E-01 | 8.10E-01 | -6.068 |
| MRE11A |  | -0.019 | 7.226 | -0.522 | 6.02E-01 | 8.10E-01 | -6.068 |
| NUDT16 |  | -0.024 | 7.545 | -0.522 | 6.02E-01 | 8.10E-01 | -6.068 |
| C18orf25 |  | 0.027 | 8.022 | 0.52 | 6.03E-01 | 8.11E-01 | -6.069 |
| PSMB7 |  | -0.018 | 11.994 | -0.521 | 6.03E-01 | 8.11E-01 | -6.069 |
| MBTPS1 |  | 0.02 | 9.862 | 0.519 | 6.03E-01 | 8.11E-01 | -6.069 |
| FCHO1 |  | 0.025 | 6.713 | 0.518 | 6.04E-01 | 8.12E-01 | -6.07 |
| TROAP |  | 0.032 | 7.721 | 0.518 | 6.04E-01 | 8.12E-01 | -6.07 |
| ITSN2 |  | 0.017 | 7.443 | 0.518 | 6.04E-01 | 8.12E-01 | -6.07 |
| PEA15 |  | 0.02 | 10.923 | 0.517 | 6.05E-01 | 8.13E-01 | -6.07 |
| CETN1 |  | -0.035 | 4.722 | -0.518 | 6.05E-01 | 8.13E-01 | -6.07 |
| TMCC3 |  | 0.027 | 8.103 | 0.516 | 6.05E-01 | 8.13E-01 | -6.071 |
| SCAMP4 |  | -0.027 | 7.668 | -0.517 | 6.05E-01 | 8.13E-01 | -6.071 |
| C14orf119 |  | 0.014 | 7.164 | 0.516 | 6.05E-01 | 8.13E-01 | -6.071 |
| IL10 |  | -0.027 | 5.99 | -0.516 | 6.06E-01 | 8.14E-01 | -6.071 |
| ACADVL |  | -0.023 | 12.603 | -0.516 | 6.06E-01 | 8.14E-01 | -6.071 |
| UNC13B |  | -0.028 | 6.678 | -0.516 | 6.06E-01 | 8.14E-01 | -6.071 |
| KIAA0196 |  | 0.02 | 9.737 | 0.515 | 6.06E-01 | 8.14E-01 | -6.071 |
| ZNF532 |  | 0.02 | 7.84 | 0.515 | 6.06E-01 | 8.14E-01 | -6.072 |
| LECT1 |  | 0.026 | 5.558 | 0.514 | 6.07E-01 | 8.14E-01 | -6.072 |
| MPP2 |  | 0.031 | 6.115 | 0.514 | 6.07E-01 | 8.14E-01 | -6.072 |
| GPR18 |  | 0.039 | 5.929 | 0.514 | 6.07E-01 | 8.14E-01 | -6.072 |
| HIF3A |  | -0.03 | 7.042 | -0.515 | 6.07E-01 | 8.14E-01 | -6.072 |
| MRPL15 |  | 0.023 | 10.607 | 0.514 | 6.07E-01 | 8.14E-01 | -6.072 |
| TRAF1 |  | 0.031 | 6.518 | 0.514 | 6.07E-01 | 8.14E-01 | -6.072 |
| OTX2 |  | 0.024 | 4.742 | 0.513 | 6.08E-01 | 8.15E-01 | -6.073 |
| TRIM35 |  | -0.029 | 7.633 | -0.513 | 6.08E-01 | 8.15E-01 | -6.073 |
| EXT2 |  | 0.016 | 9.913 | 0.512 | 6.09E-01 | 8.15E-01 | -6.073 |
| SLC2A1 |  | 0.035 | 11.219 | 0.511 | 6.09E-01 | 8.16E-01 | -6.073 |
| ARHGEF7 |  | 0.018 | 7.666 | 0.511 | 6.09E-01 | 8.16E-01 | -6.074 |
| RAB8A |  | -0.022 | 10.367 | -0.511 | 6.10E-01 | 8.16E-01 | -6.074 |
| RXRA |  | 0.02 | 9.958 | 0.509 | 6.10E-01 | 8.17E-01 | -6.074 |
| EHD2 |  | 0.03 | 8.103 | 0.509 | 6.11E-01 | 8.17E-01 | -6.074 |
| PGM5 |  | -0.03 | 5.808 | -0.509 | 6.11E-01 | 8.17E-01 | -6.075 |
| ZNF397 |  | -0.029 | 7.012 | -0.509 | 6.11E-01 | 8.17E-01 | -6.075 |
| RGS16 |  | 0.038 | 7.639 | 0.508 | 6.11E-01 | 8.17E-01 | -6.075 |
| TMED5 |  | 0.023 | 9.484 | 0.508 | 6.11E-01 | 8.17E-01 | -6.075 |
| ZNF530 |  | -0.029 | 5.747 | -0.508 | 6.12E-01 | 8.18E-01 | -6.075 |
| ADH6 |  | -0.023 | 4.714 | -0.508 | 6.12E-01 | 8.18E-01 | -6.075 |
| CYP4F3 |  | -0.032 | 5.62 | -0.508 | 6.12E-01 | 8.18E-01 | -6.076 |
| SAP30L |  | 0.023 | 9.145 | 0.506 | 6.12E-01 | 8.18E-01 | -6.076 |
| HORMAD1 |  | -0.049 | 5.573 | -0.507 | 6.13E-01 | 8.18E-01 | -6.076 |
| PAPSS2 |  | -0.032 | 9.478 | -0.507 | 6.13E-01 | 8.18E-01 | -6.076 |
| CALD1 |  | 0.03 | 9.736 | 0.505 | 6.13E-01 | 8.18E-01 | -6.076 |
| MOSPD3 |  | 0.026 | 7.076 | 0.505 | 6.13E-01 | 8.18E-01 | -6.076 |
| RRAGB |  | 0.019 | 6.884 | 0.505 | 6.13E-01 | 8.18E-01 | -6.076 |
| RHEBL1 |  | 0.027 | 6.678 | 0.505 | 6.13E-01 | 8.18E-01 | -6.076 |
| PTPN18 |  | 0.022 | 7.951 | 0.505 | 6.13E-01 | 8.18E-01 | -6.076 |
| KLK1 |  | -0.035 | 5.551 | -0.505 | 6.14E-01 | 8.19E-01 | -6.077 |
| MYBL2 |  | -0.033 | 6.481 | -0.504 | 6.14E-01 | 8.20E-01 | -6.077 |
| PDE6B |  | -0.026 | 5.896 | -0.504 | 6.15E-01 | 8.20E-01 | -6.077 |
| NCR1 |  | 0.029 | 5.9 | 0.503 | 6.15E-01 | 8.20E-01 | -6.077 |
| SYT13 |  | -0.032 | 5.189 | -0.503 | 6.15E-01 | 8.20E-01 | -6.078 |
| AAMP |  | -0.025 | 9.619 | -0.503 | 6.15E-01 | 8.20E-01 | -6.078 |
| SLC17A7 |  | -0.033 | 7.25 | -0.503 | 6.15E-01 | 8.20E-01 | -6.078 |
| POU3F2 |  | 0.032 | 4.945 | 0.502 | 6.15E-01 | 8.20E-01 | -6.078 |
| CSE1L |  | 0.018 | 10.332 | 0.501 | 6.16E-01 | 8.20E-01 | -6.078 |
| LMOD1 |  | 0.033 | 7.054 | 0.501 | 6.16E-01 | 8.20E-01 | -6.078 |
| PSMB10 |  | 0.022 | 11.365 | 0.501 | 6.16E-01 | 8.20E-01 | -6.078 |
| ADAR |  | -0.028 | 9.795 | -0.502 | 6.16E-01 | 8.20E-01 | -6.078 |
| SH2D4B |  | -0.029 | 4.897 | -0.502 | 6.16E-01 | 8.20E-01 | -6.078 |
| GALNT14 |  | 0.038 | 6.727 | 0.5 | 6.16E-01 | 8.20E-01 | -6.079 |
| TRAPPC2 |  | -0.018 | 7.691 | -0.501 | 6.17E-01 | 8.20E-01 | -6.079 |
| ACTR3 |  | 0.029 | 10.707 | 0.5 | 6.17E-01 | 8.20E-01 | -6.079 |
| SLC24A2 |  | -0.024 | 4.993 | -0.501 | 6.17E-01 | 8.20E-01 | -6.079 |
| CDAN1 |  | 0.021 | 7.274 | 0.5 | 6.17E-01 | 8.20E-01 | -6.079 |
| TAS2R10 |  | -0.032 | 5.623 | -0.5 | 6.17E-01 | 8.20E-01 | -6.079 |
| MAP3K9 |  | -0.028 | 6.639 | -0.5 | 6.17E-01 | 8.20E-01 | -6.079 |
| SLMAP |  | -0.028 | 8.162 | -0.5 | 6.18E-01 | 8.21E-01 | -6.079 |
| NFIA |  | 0.026 | 7.702 | 0.498 | 6.18E-01 | 8.21E-01 | -6.079 |
| EPHA8 |  | -0.024 | 5.308 | -0.499 | 6.18E-01 | 8.21E-01 | -6.079 |
| NUFIP1 |  | -0.025 | 6.276 | -0.499 | 6.18E-01 | 8.21E-01 | -6.079 |
| PTPN12 |  | 0.025 | 10.805 | 0.497 | 6.18E-01 | 8.21E-01 | -6.08 |
| COX6A1 |  | -0.018 | 11.788 | -0.498 | 6.19E-01 | 8.22E-01 | -6.08 |
| PCNXL2 |  | -0.02 | 6.118 | -0.498 | 6.19E-01 | 8.22E-01 | -6.08 |
| EPS15 |  | 0.022 | 8.46 | 0.496 | 6.19E-01 | 8.22E-01 | -6.081 |
| EXOC8 |  | -0.023 | 8.234 | -0.497 | 6.19E-01 | 8.22E-01 | -6.081 |
| PLRG1 |  | -0.024 | 8.898 | -0.497 | 6.19E-01 | 8.22E-01 | -6.081 |
| UBN1 |  | 0.018 | 9.684 | 0.496 | 6.20E-01 | 8.22E-01 | -6.081 |
| GNB1L |  | -0.027 | 8.23 | -0.497 | 6.20E-01 | 8.22E-01 | -6.081 |
| MSR1 |  | 0.025 | 5.894 | 0.495 | 6.20E-01 | 8.22E-01 | -6.081 |
| BTBD9 |  | 0.018 | 6.261 | 0.494 | 6.21E-01 | 8.23E-01 | -6.082 |
| NCOA1 |  | -0.02 | 8.541 | -0.495 | 6.21E-01 | 8.23E-01 | -6.082 |
| OPLAH |  | 0.03 | 7.272 | 0.494 | 6.21E-01 | 8.23E-01 | -6.082 |
| GABRB2 |  | -0.027 | 5.689 | -0.495 | 6.21E-01 | 8.23E-01 | -6.082 |
| SUPT4H1 |  | 0.016 | 10.484 | 0.493 | 6.21E-01 | 8.23E-01 | -6.082 |
| PRG3 |  | -0.034 | 5.532 | -0.494 | 6.21E-01 | 8.23E-01 | -6.082 |
| PEX11B |  | -0.018 | 9.63 | -0.494 | 6.21E-01 | 8.23E-01 | -6.082 |
| XCR1 |  | -0.031 | 6.863 | -0.494 | 6.21E-01 | 8.23E-01 | -6.082 |
| LIAS |  | -0.019 | 7.513 | -0.494 | 6.22E-01 | 8.23E-01 | -6.082 |
| CHGA |  | -0.035 | 6.531 | -0.493 | 6.22E-01 | 8.24E-01 | -6.083 |
| HIST1H1E |  | 0.039 | 7.997 | 0.492 | 6.23E-01 | 8.24E-01 | -6.083 |
| BAZ2A |  | 0.02 | 8.018 | 0.492 | 6.23E-01 | 8.24E-01 | -6.083 |
| RABGEF1 |  | 0.017 | 8.652 | 0.492 | 6.23E-01 | 8.24E-01 | -6.083 |
| PROSC |  | -0.018 | 9.048 | -0.492 | 6.23E-01 | 8.24E-01 | -6.083 |
| MLANA |  | 0.031 | 5.16 | 0.491 | 6.23E-01 | 8.24E-01 | -6.083 |
| STX18 |  | -0.016 | 8.41 | -0.492 | 6.23E-01 | 8.24E-01 | -6.083 |
| PARK2 |  | -0.021 | 5.608 | -0.491 | 6.24E-01 | 8.24E-01 | -6.083 |
| FGD5 |  | -0.029 | 7.077 | -0.49 | 6.24E-01 | 8.25E-01 | -6.084 |
| DKK4 |  | 0.021 | 4.638 | 0.489 | 6.24E-01 | 8.25E-01 | -6.084 |
| USH1C |  | -0.028 | 5.089 | -0.49 | 6.24E-01 | 8.25E-01 | -6.084 |
| EFNA3 |  | 0.026 | 5.798 | 0.489 | 6.25E-01 | 8.25E-01 | -6.084 |
| HTR2C |  | 0.031 | 4.917 | 0.489 | 6.25E-01 | 8.25E-01 | -6.084 |
| MCOLN2 |  | 0.034 | 6.565 | 0.488 | 6.25E-01 | 8.25E-01 | -6.084 |
| SPRY3 |  | -0.028 | 5.871 | -0.489 | 6.25E-01 | 8.25E-01 | -6.085 |
| WDR13 |  | -0.022 | 8.596 | -0.489 | 6.25E-01 | 8.25E-01 | -6.085 |
| ATP6V1A |  | -0.021 | 10.609 | -0.488 | 6.26E-01 | 8.26E-01 | -6.085 |
| EXOC7 |  | -0.014 | 9.505 | -0.488 | 6.26E-01 | 8.26E-01 | -6.085 |
| PGGT1B |  | -0.03 | 6.664 | -0.488 | 6.26E-01 | 8.26E-01 | -6.085 |
| OAS3 |  | -0.041 | 7.465 | -0.488 | 6.26E-01 | 8.26E-01 | -6.085 |
| PHTF1 |  | 0.027 | 7.528 | 0.486 | 6.26E-01 | 8.26E-01 | -6.085 |
| SYNPO2 |  | -0.026 | 5.572 | -0.487 | 6.27E-01 | 8.26E-01 | -6.085 |
| OSGEPL1 |  | 0.03 | 6.834 | 0.486 | 6.27E-01 | 8.26E-01 | -6.086 |
| RNF17 |  | 0.032 | 4.965 | 0.485 | 6.27E-01 | 8.26E-01 | -6.086 |
| C10orf95 |  | 0.03 | 6.859 | 0.485 | 6.27E-01 | 8.26E-01 | -6.086 |
| SYNPR |  | -0.048 | 5.367 | -0.486 | 6.27E-01 | 8.26E-01 | -6.086 |
| THUMPD1 |  | -0.021 | 8.426 | -0.486 | 6.27E-01 | 8.26E-01 | -6.086 |
| LIX1L |  | 0.024 | 7.826 | 0.484 | 6.28E-01 | 8.27E-01 | -6.086 |
| CDX1 |  | 0.026 | 5.612 | 0.484 | 6.28E-01 | 8.27E-01 | -6.086 |
| LTBR |  | -0.023 | 9.962 | -0.484 | 6.28E-01 | 8.27E-01 | -6.086 |
| OGFR |  | -0.028 | 10.243 | -0.484 | 6.28E-01 | 8.27E-01 | -6.086 |
| C2orf15 |  | 0.034 | 5.622 | 0.483 | 6.29E-01 | 8.27E-01 | -6.087 |
| PSD4 |  | 0.023 | 7.871 | 0.482 | 6.29E-01 | 8.28E-01 | -6.087 |
| ZC3HC1 |  | -0.017 | 9.269 | -0.483 | 6.29E-01 | 8.28E-01 | -6.087 |
| C9orf43 |  | 0.028 | 5.209 | 0.482 | 6.29E-01 | 8.28E-01 | -6.087 |
| IGSF3 |  | -0.026 | 7.078 | -0.483 | 6.30E-01 | 8.28E-01 | -6.087 |
| FAU |  | 0.018 | 13.738 | 0.479 | 6.31E-01 | 8.30E-01 | -6.088 |
| LHX6 |  | 0.024 | 6.606 | 0.479 | 6.32E-01 | 8.30E-01 | -6.089 |
| ANGPT2 |  | 0.056 | 8.382 | 0.479 | 6.32E-01 | 8.30E-01 | -6.089 |
| CAMKK1 |  | -0.023 | 6.023 | -0.48 | 6.32E-01 | 8.30E-01 | -6.089 |
| NEFH |  | 0.035 | 6.626 | 0.478 | 6.32E-01 | 8.30E-01 | -6.089 |
| IQSEC1 |  | -0.021 | 8.954 | -0.479 | 6.32E-01 | 8.30E-01 | -6.089 |
| ARIH2 |  | 0.014 | 9.513 | 0.478 | 6.32E-01 | 8.30E-01 | -6.089 |
| CASR |  | 0.026 | 5.12 | 0.478 | 6.32E-01 | 8.30E-01 | -6.089 |
| CDC37 |  | 0.017 | 11.022 | 0.477 | 6.33E-01 | 8.30E-01 | -6.09 |
| FTH1 |  | -0.024 | 12.392 | -0.478 | 6.33E-01 | 8.30E-01 | -6.09 |
| UPK3B |  | -0.026 | 7.381 | -0.478 | 6.33E-01 | 8.30E-01 | -6.09 |
| TMEM18 |  | -0.019 | 8.155 | -0.478 | 6.33E-01 | 8.30E-01 | -6.09 |
| GNAI3 |  | -0.02 | 8.599 | -0.477 | 6.33E-01 | 8.30E-01 | -6.09 |
| MAGED1 |  | -0.021 | 10.822 | -0.477 | 6.33E-01 | 8.30E-01 | -6.09 |
| ALDH1A3 |  | -0.032 | 7.218 | -0.476 | 6.34E-01 | 8.31E-01 | -6.09 |
| CCDC9 |  | -0.027 | 7.743 | -0.476 | 6.34E-01 | 8.31E-01 | -6.09 |
| PDP2 |  | -0.023 | 6.292 | -0.476 | 6.34E-01 | 8.31E-01 | -6.09 |
| RAF1 |  | 0.014 | 10.4 | 0.475 | 6.35E-01 | 8.31E-01 | -6.091 |
| PDCL |  | 0.016 | 8.327 | 0.475 | 6.35E-01 | 8.31E-01 | -6.091 |
| HMGCL |  | -0.017 | 8.876 | -0.475 | 6.35E-01 | 8.32E-01 | -6.091 |
| WNT9B |  | -0.028 | 5.376 | -0.475 | 6.35E-01 | 8.32E-01 | -6.091 |
| SLC39A12 |  | 0.031 | 5.217 | 0.474 | 6.35E-01 | 8.32E-01 | -6.091 |
| RELB |  | 0.035 | 8.607 | 0.473 | 6.36E-01 | 8.32E-01 | -6.091 |
| CLUL1 |  | 0.029 | 4.736 | 0.473 | 6.36E-01 | 8.32E-01 | -6.091 |
| PON2 |  | 0.023 | 10.054 | 0.472 | 6.36E-01 | 8.32E-01 | -6.092 |
| MAL2 |  | -0.028 | 7.142 | -0.473 | 6.36E-01 | 8.32E-01 | -6.092 |
| TMEM42 |  | 0.023 | 9.072 | 0.472 | 6.36E-01 | 8.32E-01 | -6.092 |
| ITGA2B |  | 0.029 | 6.982 | 0.472 | 6.37E-01 | 8.32E-01 | -6.092 |
| IL15 |  | -0.041 | 7.772 | -0.473 | 6.37E-01 | 8.32E-01 | -6.092 |
| SLC35C2 |  | -0.02 | 8.726 | -0.472 | 6.37E-01 | 8.32E-01 | -6.092 |
| NPHP4 |  | 0.025 | 6.698 | 0.471 | 6.37E-01 | 8.32E-01 | -6.092 |
| KRT20 |  | -0.031 | 5.214 | -0.472 | 6.37E-01 | 8.32E-01 | -6.092 |
| IGFBP1 |  | -0.105 | 7.788 | -0.472 | 6.37E-01 | 8.32E-01 | -6.092 |
| HMGCR |  | 0.029 | 9.466 | 0.471 | 6.37E-01 | 8.32E-01 | -6.092 |
| FCN3 |  | -0.027 | 8.107 | -0.471 | 6.38E-01 | 8.33E-01 | -6.093 |
| ARX |  | 0.026 | 5.627 | 0.47 | 6.38E-01 | 8.33E-01 | -6.093 |
| CTNNB1 |  | 0.015 | 9.62 | 0.47 | 6.38E-01 | 8.33E-01 | -6.093 |
| HTATIP2 |  | 0.022 | 9.038 | 0.469 | 6.38E-01 | 8.33E-01 | -6.093 |
| NEGR1 |  | 0.027 | 5.821 | 0.469 | 6.39E-01 | 8.33E-01 | -6.093 |
| TPD52 |  | 0.018 | 7.455 | 0.469 | 6.39E-01 | 8.33E-01 | -6.093 |
| CDC42SE1 |  | -0.023 | 9.521 | -0.469 | 6.39E-01 | 8.33E-01 | -6.093 |
| BUB1 |  | 0.033 | 7.046 | 0.468 | 6.39E-01 | 8.33E-01 | -6.093 |
| ZBTB12 |  | -0.033 | 5.129 | -0.469 | 6.39E-01 | 8.33E-01 | -6.093 |
| MANEA |  | -0.024 | 6.856 | -0.469 | 6.39E-01 | 8.33E-01 | -6.094 |
| BAZ1B |  | -0.016 | 9.026 | -0.469 | 6.40E-01 | 8.33E-01 | -6.094 |
| TMF1 |  | -0.024 | 8.296 | -0.468 | 6.40E-01 | 8.33E-01 | -6.094 |
| MCPH1 |  | 0.019 | 6.995 | 0.467 | 6.40E-01 | 8.33E-01 | -6.094 |
| NEK8 |  | 0.023 | 6.268 | 0.467 | 6.40E-01 | 8.33E-01 | -6.094 |
| RGMA |  | 0.029 | 6.327 | 0.467 | 6.40E-01 | 8.33E-01 | -6.094 |
| DAPP1 |  | 0.03 | 8.106 | 0.467 | 6.40E-01 | 8.33E-01 | -6.094 |
| CLU |  | -0.034 | 8.809 | -0.468 | 6.40E-01 | 8.34E-01 | -6.094 |
| SLC30A3 |  | 0.029 | 6.241 | 0.466 | 6.40E-01 | 8.34E-01 | -6.094 |
| STAT6 |  | 0.029 | 10.154 | 0.466 | 6.41E-01 | 8.34E-01 | -6.094 |
| NR0B2 |  | -0.03 | 4.977 | -0.467 | 6.41E-01 | 8.34E-01 | -6.095 |
| KCNQ4 |  | 0.017 | 5.485 | 0.465 | 6.41E-01 | 8.34E-01 | -6.095 |
| TLL2 |  | -0.024 | 5.043 | -0.466 | 6.42E-01 | 8.35E-01 | -6.095 |
| ZNF426 |  | 0.019 | 7.864 | 0.464 | 6.42E-01 | 8.35E-01 | -6.095 |
| HIC1 |  | 0.024 | 6.316 | 0.464 | 6.42E-01 | 8.35E-01 | -6.096 |
| COL9A3 |  | 0.038 | 7.2 | 0.463 | 6.43E-01 | 8.35E-01 | -6.096 |
| SLC39A10 |  | -0.028 | 8.471 | -0.464 | 6.43E-01 | 8.36E-01 | -6.096 |
| CD3E |  | 0.022 | 6.222 | 0.462 | 6.44E-01 | 8.36E-01 | -6.096 |
| BIK |  | 0.029 | 5.853 | 0.462 | 6.44E-01 | 8.36E-01 | -6.096 |
| CDH3 |  | -0.035 | 7.215 | -0.462 | 6.44E-01 | 8.37E-01 | -6.097 |
| MAN2A1 |  | -0.023 | 9.568 | -0.461 | 6.45E-01 | 8.37E-01 | -6.097 |
| F11 |  | 0.019 | 4.925 | 0.46 | 6.45E-01 | 8.37E-01 | -6.097 |
| GMIP |  | 0.027 | 7.874 | 0.46 | 6.45E-01 | 8.37E-01 | -6.097 |
| CGN |  | 0.031 | 6.574 | 0.46 | 6.45E-01 | 8.37E-01 | -6.097 |
| RNF20 |  | 0.024 | 8.518 | 0.46 | 6.45E-01 | 8.37E-01 | -6.097 |
| DDHD1 |  | -0.021 | 6.84 | -0.461 | 6.45E-01 | 8.37E-01 | -6.097 |
| SCUBE2 |  | -0.036 | 6.495 | -0.46 | 6.46E-01 | 8.38E-01 | -6.098 |
| ZBTB9 |  | -0.027 | 7.544 | -0.46 | 6.46E-01 | 8.38E-01 | -6.098 |
| TCEA1 |  | -0.017 | 9.294 | -0.46 | 6.46E-01 | 8.38E-01 | -6.098 |
| HYAL1 |  | -0.03 | 5.867 | -0.459 | 6.47E-01 | 8.38E-01 | -6.098 |
| PTPRM |  | -0.021 | 9.126 | -0.459 | 6.47E-01 | 8.38E-01 | -6.098 |
| PF4V1 |  | 0.032 | 6.382 | 0.457 | 6.47E-01 | 8.38E-01 | -6.098 |
| PHOX2B |  | 0.023 | 4.893 | 0.457 | 6.47E-01 | 8.38E-01 | -6.098 |
| TIMP4 |  | -0.036 | 6.218 | -0.458 | 6.47E-01 | 8.38E-01 | -6.098 |
| SCRN3 |  | -0.018 | 7.088 | -0.458 | 6.47E-01 | 8.38E-01 | -6.099 |
| NR2C1 |  | 0.02 | 7.818 | 0.457 | 6.47E-01 | 8.38E-01 | -6.099 |
| NIF3L1 |  | -0.016 | 10.329 | -0.458 | 6.47E-01 | 8.38E-01 | -6.099 |
| CD74 |  | -0.04 | 8.22 | -0.458 | 6.47E-01 | 8.38E-01 | -6.099 |
| SUCLG1 |  | 0.015 | 10.253 | 0.456 | 6.48E-01 | 8.39E-01 | -6.099 |
| LOXL2 |  | -0.027 | 7.768 | -0.457 | 6.48E-01 | 8.39E-01 | -6.099 |
| CORO6 |  | -0.04 | 9.264 | -0.456 | 6.48E-01 | 8.39E-01 | -6.099 |
| MEIS1 |  | -0.023 | 7.543 | -0.456 | 6.49E-01 | 8.40E-01 | -6.1 |
| APOE |  | -0.039 | 10.132 | -0.455 | 6.49E-01 | 8.40E-01 | -6.1 |
| HSPA4L |  | -0.038 | 5.708 | -0.455 | 6.50E-01 | 8.40E-01 | -6.1 |
| CST8 |  | -0.024 | 4.831 | -0.455 | 6.50E-01 | 8.40E-01 | -6.1 |
| SLPI |  | 0.045 | 7.438 | 0.453 | 6.50E-01 | 8.40E-01 | -6.1 |
| FRAS1 |  | -0.02 | 6.242 | -0.454 | 6.50E-01 | 8.40E-01 | -6.1 |
| BOC |  | 0.025 | 5.394 | 0.453 | 6.50E-01 | 8.40E-01 | -6.1 |
| COL4A6 |  | 0.025 | 5.754 | 0.453 | 6.50E-01 | 8.40E-01 | -6.1 |
| OSBPL6 |  | -0.023 | 5.989 | -0.453 | 6.51E-01 | 8.40E-01 | -6.101 |
| CPA5 |  | -0.028 | 5.871 | -0.453 | 6.51E-01 | 8.41E-01 | -6.101 |
| S100A12 |  | -0.044 | 7.76 | -0.453 | 6.51E-01 | 8.41E-01 | -6.101 |
| CAMK2D |  | 0.021 | 6.866 | 0.451 | 6.52E-01 | 8.41E-01 | -6.101 |
| NAB1 |  | 0.023 | 8.477 | 0.45 | 6.52E-01 | 8.41E-01 | -6.101 |
| PYHIN1 |  | -0.024 | 5.788 | -0.451 | 6.52E-01 | 8.41E-01 | -6.101 |
| TRIM29 |  | 0.035 | 6.942 | 0.45 | 6.52E-01 | 8.41E-01 | -6.101 |
| CILP2 |  | 0.033 | 5.683 | 0.45 | 6.52E-01 | 8.41E-01 | -6.102 |
| C7orf13 |  | -0.026 | 6.296 | -0.451 | 6.52E-01 | 8.41E-01 | -6.102 |
| FEN1 |  | -0.019 | 8.76 | -0.451 | 6.52E-01 | 8.41E-01 | -6.102 |
| HOXD9 |  | 0.029 | 5.859 | 0.45 | 6.52E-01 | 8.41E-01 | -6.102 |
| ATOH1 |  | 0.023 | 4.781 | 0.45 | 6.52E-01 | 8.41E-01 | -6.102 |
| BNIP1 |  | -0.023 | 7.804 | -0.45 | 6.53E-01 | 8.41E-01 | -6.102 |
| SPIRE2 |  | -0.034 | 8.086 | -0.45 | 6.53E-01 | 8.41E-01 | -6.102 |
| OSBP |  | -0.026 | 9.616 | -0.45 | 6.53E-01 | 8.41E-01 | -6.102 |
| POLR2L |  | 0.021 | 10.516 | 0.449 | 6.53E-01 | 8.41E-01 | -6.102 |
| PRKRA |  | -0.021 | 9.293 | -0.449 | 6.53E-01 | 8.42E-01 | -6.102 |
| TNFRSF21 |  | 0.023 | 9.387 | 0.448 | 6.54E-01 | 8.42E-01 | -6.102 |
| MTBP |  | -0.027 | 5.876 | -0.449 | 6.54E-01 | 8.42E-01 | -6.102 |
| HOXC11 |  | 0.02 | 4.894 | 0.447 | 6.55E-01 | 8.43E-01 | -6.103 |
| CACNA2D4 |  | 0.022 | 6.244 | 0.446 | 6.55E-01 | 8.43E-01 | -6.103 |
| GLCE |  | 0.029 | 8.384 | 0.446 | 6.55E-01 | 8.43E-01 | -6.103 |
| HIST1H3G |  | -0.028 | 7.8 | -0.447 | 6.55E-01 | 8.43E-01 | -6.103 |
| EVI5L |  | 0.025 | 8.619 | 0.445 | 6.56E-01 | 8.43E-01 | -6.104 |
| RPRM |  | 0.042 | 5.522 | 0.445 | 6.56E-01 | 8.43E-01 | -6.104 |
| ENTPD1 |  | 0.032 | 8.285 | 0.445 | 6.56E-01 | 8.43E-01 | -6.104 |
| ZNF274 |  | -0.017 | 8.328 | -0.445 | 6.57E-01 | 8.44E-01 | -6.104 |
| FAM3C |  | -0.021 | 9.392 | -0.445 | 6.57E-01 | 8.44E-01 | -6.104 |
| RASD1 |  | -0.039 | 6.999 | -0.444 | 6.57E-01 | 8.45E-01 | -6.105 |
| TRPM5 |  | 0.031 | 6.257 | 0.443 | 6.57E-01 | 8.45E-01 | -6.105 |
| TANK |  | 0.021 | 8.934 | 0.443 | 6.57E-01 | 8.45E-01 | -6.105 |
| HYAL4 |  | -0.022 | 6.67 | -0.443 | 6.58E-01 | 8.45E-01 | -6.105 |
| DAZL |  | -0.033 | 5.036 | -0.443 | 6.58E-01 | 8.45E-01 | -6.105 |
| RPS21 |  | 0.021 | 12.354 | 0.442 | 6.58E-01 | 8.45E-01 | -6.105 |
| DDX28 |  | -0.017 | 9.54 | -0.442 | 6.59E-01 | 8.45E-01 | -6.105 |
| MPP3 |  | -0.028 | 6.275 | -0.442 | 6.59E-01 | 8.45E-01 | -6.105 |
| SIGLEC11 |  | -0.03 | 6.051 | -0.442 | 6.59E-01 | 8.46E-01 | -6.106 |
| KCNJ10 |  | -0.022 | 5.462 | -0.441 | 6.59E-01 | 8.46E-01 | -6.106 |
| LPA |  | -0.019 | 5.453 | -0.441 | 6.59E-01 | 8.46E-01 | -6.106 |
| MAP2 |  | -0.025 | 5.328 | -0.441 | 6.59E-01 | 8.46E-01 | -6.106 |
| NLGN4Y |  | -0.04 | 5.521 | -0.44 | 6.60E-01 | 8.46E-01 | -6.106 |
| SEC23IP |  | -0.017 | 8.356 | -0.44 | 6.60E-01 | 8.46E-01 | -6.106 |
| GATA2 |  | 0.029 | 9.296 | 0.439 | 6.60E-01 | 8.46E-01 | -6.106 |
| CNTNAP4 |  | 0.017 | 5.127 | 0.439 | 6.60E-01 | 8.46E-01 | -6.106 |
| TESK2 |  | -0.022 | 7.905 | -0.439 | 6.61E-01 | 8.47E-01 | -6.107 |
| SHCBP1 |  | 0.029 | 7.285 | 0.438 | 6.61E-01 | 8.47E-01 | -6.107 |
| PNPLA1 |  | -0.029 | 5.245 | -0.439 | 6.61E-01 | 8.47E-01 | -6.107 |
| MAN2B1 |  | 0.021 | 9.169 | 0.437 | 6.62E-01 | 8.47E-01 | -6.107 |
| ABCC10 |  | -0.021 | 7.685 | -0.438 | 6.62E-01 | 8.47E-01 | -6.107 |
| GCK |  | 0.025 | 6.192 | 0.437 | 6.62E-01 | 8.47E-01 | -6.107 |
| C6orf10 |  | -0.023 | 4.829 | -0.438 | 6.62E-01 | 8.47E-01 | -6.107 |
| HTR1A |  | -0.025 | 5.078 | -0.437 | 6.62E-01 | 8.47E-01 | -6.107 |
| LYPLA1 |  | -0.018 | 10.067 | -0.437 | 6.62E-01 | 8.47E-01 | -6.107 |
| ITM2A |  | 0.036 | 8.513 | 0.436 | 6.62E-01 | 8.47E-01 | -6.108 |
| TRIM54 |  | 0.022 | 5.237 | 0.436 | 6.63E-01 | 8.47E-01 | -6.108 |
| IGFBP6 |  | -0.046 | 8.124 | -0.436 | 6.63E-01 | 8.47E-01 | -6.108 |
| GLRA2 |  | -0.029 | 5.079 | -0.436 | 6.63E-01 | 8.48E-01 | -6.108 |
| DMRTB1 |  | 0.023 | 4.971 | 0.435 | 6.63E-01 | 8.48E-01 | -6.108 |
| GAS2 |  | 0.025 | 5.225 | 0.435 | 6.63E-01 | 8.48E-01 | -6.108 |
| MET |  | 0.029 | 8.076 | 0.434 | 6.64E-01 | 8.48E-01 | -6.108 |
| SUZ12 |  | -0.02 | 10.15 | -0.434 | 6.65E-01 | 8.49E-01 | -6.109 |
| ZW10 |  | 0.017 | 8.145 | 0.432 | 6.65E-01 | 8.49E-01 | -6.109 |
| ANP32D |  | -0.029 | 6.791 | -0.433 | 6.65E-01 | 8.49E-01 | -6.109 |
| GNA13 |  | -0.023 | 8.523 | -0.433 | 6.65E-01 | 8.49E-01 | -6.109 |
| SLC10A1 |  | -0.026 | 5.28 | -0.433 | 6.65E-01 | 8.49E-01 | -6.109 |
| CDH11 |  | -0.03 | 8.365 | -0.433 | 6.65E-01 | 8.49E-01 | -6.109 |
| SCTR |  | 0.024 | 5.246 | 0.431 | 6.66E-01 | 8.50E-01 | -6.11 |
| GLRA3 |  | 0.023 | 4.822 | 0.43 | 6.67E-01 | 8.51E-01 | -6.11 |
| CPXCR1 |  | -0.022 | 4.884 | -0.431 | 6.67E-01 | 8.51E-01 | -6.11 |
| PPP1R16A |  | 0.018 | 9.021 | 0.43 | 6.67E-01 | 8.51E-01 | -6.11 |
| ZHX2 |  | -0.021 | 7.315 | -0.43 | 6.67E-01 | 8.51E-01 | -6.11 |
| IMPA1 |  | -0.022 | 7.901 | -0.43 | 6.67E-01 | 8.51E-01 | -6.11 |
| MAX |  | -0.017 | 8.36 | -0.43 | 6.67E-01 | 8.51E-01 | -6.11 |
| AOX1 |  | 0.046 | 7.196 | 0.428 | 6.68E-01 | 8.51E-01 | -6.111 |
| CCNT1 |  | -0.021 | 7.51 | -0.429 | 6.68E-01 | 8.51E-01 | -6.111 |
| HELLS |  | 0.025 | 6.628 | 0.428 | 6.68E-01 | 8.51E-01 | -6.111 |
| MSN |  | -0.02 | 9.976 | -0.428 | 6.69E-01 | 8.52E-01 | -6.111 |
| LPO |  | 0.027 | 5.11 | 0.426 | 6.69E-01 | 8.52E-01 | -6.111 |
| ST8SIA5 |  | -0.026 | 5.282 | -0.427 | 6.70E-01 | 8.53E-01 | -6.112 |
| SPAG5 |  | 0.025 | 8.216 | 0.426 | 6.70E-01 | 8.53E-01 | -6.112 |
| EP400 |  | -0.018 | 7.79 | -0.427 | 6.70E-01 | 8.53E-01 | -6.112 |
| ALLC |  | 0.02 | 4.893 | 0.425 | 6.71E-01 | 8.53E-01 | -6.112 |
| DPM2 |  | 0.019 | 8.915 | 0.425 | 6.71E-01 | 8.53E-01 | -6.112 |
| USP49 |  | -0.034 | 7.662 | -0.425 | 6.71E-01 | 8.54E-01 | -6.112 |
| SALL4 |  | 0.032 | 5.249 | 0.424 | 6.71E-01 | 8.54E-01 | -6.113 |
| ZNF569 |  | 0.028 | 6.275 | 0.423 | 6.72E-01 | 8.54E-01 | -6.113 |
| JAM3 |  | -0.027 | 9.543 | -0.423 | 6.72E-01 | 8.55E-01 | -6.113 |
| GEMIN7 |  | 0.016 | 7.095 | 0.422 | 6.72E-01 | 8.55E-01 | -6.113 |
| ADIPOR2 |  | 0.015 | 9.9 | 0.422 | 6.73E-01 | 8.55E-01 | -6.113 |
| MNT |  | 0.016 | 9.491 | 0.422 | 6.73E-01 | 8.55E-01 | -6.113 |
| SLC6A16 |  | 0.025 | 6.373 | 0.422 | 6.73E-01 | 8.55E-01 | -6.113 |
| ZNF653 |  | -0.019 | 7.557 | -0.422 | 6.73E-01 | 8.55E-01 | -6.114 |
| PRKCZ |  | -0.022 | 9.685 | -0.422 | 6.73E-01 | 8.55E-01 | -6.114 |
| WFDC2 |  | 0.024 | 5.981 | 0.421 | 6.73E-01 | 8.55E-01 | -6.114 |
| L3MBTL3 |  | -0.02 | 7.421 | -0.422 | 6.74E-01 | 8.55E-01 | -6.114 |
| NTRK3 |  | -0.021 | 5.477 | -0.421 | 6.74E-01 | 8.55E-01 | -6.114 |
| ADAMTS20 |  | 0.018 | 5.274 | 0.42 | 6.74E-01 | 8.55E-01 | -6.114 |
| PRKCA |  | -0.023 | 8.107 | -0.421 | 6.74E-01 | 8.55E-01 | -6.114 |
| COG7 |  | -0.023 | 7.812 | -0.421 | 6.74E-01 | 8.55E-01 | -6.114 |
| FLOT1 |  | 0.021 | 10.361 | 0.42 | 6.74E-01 | 8.55E-01 | -6.114 |
| CNN3 |  | 0.022 | 10.788 | 0.419 | 6.75E-01 | 8.55E-01 | -6.114 |
| SIX6 |  | -0.024 | 4.864 | -0.42 | 6.75E-01 | 8.56E-01 | -6.115 |
| DIAPH1 |  | 0.029 | 9.903 | 0.419 | 6.75E-01 | 8.56E-01 | -6.115 |
| PHF20 |  | 0.016 | 7.5 | 0.418 | 6.75E-01 | 8.56E-01 | -6.115 |
| AUP1 |  | 0.016 | 9.877 | 0.418 | 6.76E-01 | 8.56E-01 | -6.115 |
| TESK1 |  | 0.017 | 9.285 | 0.418 | 6.76E-01 | 8.56E-01 | -6.115 |
| NT5C2 |  | -0.02 | 9.413 | -0.418 | 6.76E-01 | 8.56E-01 | -6.115 |
| PSAP |  | -0.022 | 12.459 | -0.418 | 6.76E-01 | 8.56E-01 | -6.115 |
| PTPRCAP |  | -0.021 | 7.516 | -0.418 | 6.76E-01 | 8.56E-01 | -6.115 |
| PIM3 |  | 0.022 | 10.713 | 0.417 | 6.76E-01 | 8.56E-01 | -6.115 |
| USP54 |  | 0.018 | 6.648 | 0.417 | 6.76E-01 | 8.56E-01 | -6.115 |
| KNDC1 |  | -0.018 | 5.332 | -0.418 | 6.76E-01 | 8.56E-01 | -6.115 |
| SCN10A |  | -0.022 | 4.856 | -0.417 | 6.77E-01 | 8.56E-01 | -6.116 |
| PDYN |  | -0.019 | 4.951 | -0.417 | 6.77E-01 | 8.56E-01 | -6.116 |
| CLK3 |  | -0.023 | 9.563 | -0.417 | 6.77E-01 | 8.56E-01 | -6.116 |
| BACE2 |  | -0.025 | 9.158 | -0.417 | 6.77E-01 | 8.56E-01 | -6.116 |
| GNL3L |  | 0.019 | 8.467 | 0.415 | 6.77E-01 | 8.57E-01 | -6.116 |
| MYL3 |  | -0.022 | 5.855 | -0.416 | 6.77E-01 | 8.57E-01 | -6.116 |
| CRTAC1 |  | -0.029 | 5.403 | -0.416 | 6.78E-01 | 8.57E-01 | -6.116 |
| CPN2 |  | 0.024 | 5.231 | 0.414 | 6.78E-01 | 8.57E-01 | -6.116 |
| ELP4 |  | 0.022 | 7.802 | 0.414 | 6.78E-01 | 8.57E-01 | -6.117 |
| UROD |  | 0.018 | 11.025 | 0.414 | 6.78E-01 | 8.57E-01 | -6.117 |
| GPR22 |  | -0.024 | 4.543 | -0.415 | 6.79E-01 | 8.57E-01 | -6.117 |
| KCNB2 |  | -0.025 | 4.861 | -0.414 | 6.79E-01 | 8.57E-01 | -6.117 |
| DHX38 |  | 0.018 | 8.381 | 0.412 | 6.80E-01 | 8.58E-01 | -6.117 |
| EDN2 |  | -0.022 | 5.013 | -0.413 | 6.80E-01 | 8.59E-01 | -6.117 |
| BTK |  | 0.023 | 6.984 | 0.411 | 6.81E-01 | 8.59E-01 | -6.118 |
| ACBD3 |  | 0.018 | 9.683 | 0.411 | 6.81E-01 | 8.59E-01 | -6.118 |
| ZNF225 |  | 0.023 | 6.18 | 0.411 | 6.81E-01 | 8.59E-01 | -6.118 |
| LRP12 |  | -0.026 | 6.478 | -0.411 | 6.81E-01 | 8.60E-01 | -6.118 |
| F9 |  | 0.019 | 4.649 | 0.409 | 6.82E-01 | 8.60E-01 | -6.118 |
| HFE2 |  | -0.021 | 4.89 | -0.41 | 6.82E-01 | 8.60E-01 | -6.119 |
| HSF4 |  | 0.02 | 6.726 | 0.409 | 6.82E-01 | 8.60E-01 | -6.119 |
| KLF15 |  | -0.022 | 5.697 | -0.41 | 6.82E-01 | 8.60E-01 | -6.119 |
| IL26 |  | 0.021 | 4.906 | 0.408 | 6.83E-01 | 8.60E-01 | -6.119 |
| TAGLN2 |  | 0.018 | 11.449 | 0.408 | 6.83E-01 | 8.61E-01 | -6.119 |
| CLK4 |  | 0.021 | 8.071 | 0.408 | 6.83E-01 | 8.61E-01 | -6.119 |
| PYGO2 |  | 0.016 | 8.636 | 0.407 | 6.83E-01 | 8.61E-01 | -6.119 |
| NDUFA4 |  | 0.02 | 11.861 | 0.407 | 6.84E-01 | 8.61E-01 | -6.119 |
| SLC27A4 |  | 0.022 | 6.634 | 0.406 | 6.84E-01 | 8.61E-01 | -6.12 |
| OSBPL3 |  | 0.019 | 7.304 | 0.406 | 6.84E-01 | 8.61E-01 | -6.12 |
| HAL |  | -0.024 | 5.648 | -0.407 | 6.84E-01 | 8.61E-01 | -6.12 |
| NPHS2 |  | -0.027 | 5.407 | -0.406 | 6.85E-01 | 8.62E-01 | -6.12 |
| TRPV2 |  | 0.022 | 9.283 | 0.405 | 6.85E-01 | 8.62E-01 | -6.12 |
| ADCY4 |  | 0.019 | 9.14 | 0.405 | 6.85E-01 | 8.62E-01 | -6.12 |
| ZDHHC23 |  | -0.021 | 6.531 | -0.406 | 6.85E-01 | 8.62E-01 | -6.12 |
| ZNF615 |  | 0.018 | 7.528 | 0.404 | 6.86E-01 | 8.62E-01 | -6.121 |
| FYTTD1 |  | -0.02 | 9.254 | -0.405 | 6.86E-01 | 8.62E-01 | -6.121 |
| DIO3 |  | 0.028 | 6.604 | 0.403 | 6.86E-01 | 8.62E-01 | -6.121 |
| KCTD18 |  | 0.022 | 7.1 | 0.403 | 6.86E-01 | 8.62E-01 | -6.121 |
| CCL25 |  | 0.02 | 5.521 | 0.403 | 6.86E-01 | 8.62E-01 | -6.121 |
| WFDC10B |  | 0.025 | 5.685 | 0.403 | 6.86E-01 | 8.62E-01 | -6.121 |
| OR51B4 |  | -0.027 | 4.758 | -0.404 | 6.86E-01 | 8.62E-01 | -6.121 |
| SPARC |  | -0.026 | 12.862 | -0.403 | 6.87E-01 | 8.62E-01 | -6.121 |
| ALPPL2 |  | 0.027 | 6.817 | 0.402 | 6.87E-01 | 8.62E-01 | -6.121 |
| TIGD1 |  | 0.021 | 6.796 | 0.402 | 6.87E-01 | 8.62E-01 | -6.121 |
| CNTFR |  | -0.026 | 5.616 | -0.403 | 6.87E-01 | 8.62E-01 | -6.121 |
| RHAG |  | 0.034 | 5.529 | 0.402 | 6.87E-01 | 8.63E-01 | -6.121 |
| ADIPOQ |  | -0.024 | 4.92 | -0.402 | 6.88E-01 | 8.63E-01 | -6.122 |
| MAP4K4 |  | -0.017 | 8.863 | -0.402 | 6.88E-01 | 8.63E-01 | -6.122 |
| ABCG5 |  | -0.028 | 4.92 | -0.402 | 6.88E-01 | 8.63E-01 | -6.122 |
| TEX13A |  | -0.023 | 4.883 | -0.401 | 6.89E-01 | 8.64E-01 | -6.122 |
| PDZRN4 |  | -0.021 | 6.135 | -0.4 | 6.90E-01 | 8.65E-01 | -6.123 |
| TDRD3 |  | 0.018 | 6.598 | 0.398 | 6.90E-01 | 8.66E-01 | -6.123 |
| OAZ1 |  | -0.009 | 14.715 | -0.398 | 6.91E-01 | 8.66E-01 | -6.123 |
| POLE4 |  | 0.018 | 10.344 | 0.397 | 6.91E-01 | 8.66E-01 | -6.123 |
| SLC25A20 |  | 0.017 | 8.151 | 0.397 | 6.91E-01 | 8.66E-01 | -6.123 |
| PLA2G12B |  | -0.025 | 5.295 | -0.397 | 6.91E-01 | 8.66E-01 | -6.123 |
| ARHGAP25 |  | 0.021 | 7.803 | 0.396 | 6.91E-01 | 8.66E-01 | -6.123 |
| SEMA6A |  | -0.024 | 7.798 | -0.397 | 6.91E-01 | 8.66E-01 | -6.123 |
| MAB21L2 |  | -0.037 | 6.008 | -0.396 | 6.92E-01 | 8.67E-01 | -6.124 |
| INSR |  | -0.034 | 7.783 | -0.395 | 6.93E-01 | 8.68E-01 | -6.124 |
| PPP3CC |  | 0.017 | 9.062 | 0.394 | 6.93E-01 | 8.68E-01 | -6.125 |
| PARP16 |  | 0.017 | 7.589 | 0.393 | 6.93E-01 | 8.68E-01 | -6.125 |
| WT1 |  | -0.038 | 5.742 | -0.394 | 6.93E-01 | 8.68E-01 | -6.125 |
| OTOR |  | -0.021 | 4.793 | -0.394 | 6.93E-01 | 8.68E-01 | -6.125 |
| CRYGB |  | -0.02 | 4.751 | -0.394 | 6.94E-01 | 8.68E-01 | -6.125 |
| MARK4 |  | 0.017 | 6.264 | 0.393 | 6.94E-01 | 8.68E-01 | -6.125 |
| GRB2 |  | -0.014 | 10.069 | -0.393 | 6.95E-01 | 8.68E-01 | -6.125 |
| PKHD1L1 |  | -0.023 | 4.856 | -0.393 | 6.95E-01 | 8.68E-01 | -6.125 |
| SCP2 |  | -0.021 | 8.232 | -0.393 | 6.95E-01 | 8.68E-01 | -6.125 |
| SGSH |  | -0.018 | 9.984 | -0.393 | 6.95E-01 | 8.68E-01 | -6.125 |
| LDOC1 |  | 0.027 | 7.669 | 0.391 | 6.95E-01 | 8.68E-01 | -6.125 |
| MPHOSPH6 |  | 0.02 | 7.847 | 0.391 | 6.95E-01 | 8.68E-01 | -6.125 |
| CALR3 |  | 0.027 | 5.162 | 0.391 | 6.95E-01 | 8.68E-01 | -6.125 |
| SUSD1 |  | -0.018 | 9.249 | -0.392 | 6.95E-01 | 8.68E-01 | -6.125 |
| C1orf27 |  | -0.018 | 7.836 | -0.391 | 6.96E-01 | 8.69E-01 | -6.126 |
| SSTR3 |  | 0.028 | 6.962 | 0.39 | 6.96E-01 | 8.69E-01 | -6.126 |
| MEP1B |  | 0.021 | 4.782 | 0.39 | 6.96E-01 | 8.69E-01 | -6.126 |
| CECR5 |  | 0.015 | 8.932 | 0.39 | 6.96E-01 | 8.69E-01 | -6.126 |
| IL22RA1 |  | -0.021 | 5.03 | -0.391 | 6.96E-01 | 8.69E-01 | -6.126 |
| CASP3 |  | 0.021 | 9.347 | 0.389 | 6.97E-01 | 8.69E-01 | -6.126 |
| MFAP1 |  | 0.013 | 9.806 | 0.389 | 6.97E-01 | 8.69E-01 | -6.126 |
| SLC26A7 |  | -0.032 | 5.945 | -0.389 | 6.97E-01 | 8.69E-01 | -6.126 |
| ATP6V1G1 |  | 0.024 | 11.566 | 0.388 | 6.97E-01 | 8.69E-01 | -6.127 |
| GPR171 |  | 0.035 | 6.77 | 0.388 | 6.97E-01 | 8.69E-01 | -6.127 |
| PTPN13 |  | -0.027 | 7.332 | -0.389 | 6.98E-01 | 8.70E-01 | -6.127 |
| LGI1 |  | 0.023 | 4.748 | 0.388 | 6.98E-01 | 8.70E-01 | -6.127 |
| MRPS12 |  | -0.013 | 9.256 | -0.388 | 6.98E-01 | 8.70E-01 | -6.127 |
| AP1G1 |  | 0.013 | 8.939 | 0.387 | 6.98E-01 | 8.70E-01 | -6.127 |
| EIF5A |  | -0.022 | 10.613 | -0.388 | 6.98E-01 | 8.70E-01 | -6.127 |
| SERPINF2 |  | -0.026 | 5.665 | -0.388 | 6.98E-01 | 8.70E-01 | -6.127 |
| TIMM23 |  | -0.015 | 9.81 | -0.387 | 6.99E-01 | 8.70E-01 | -6.127 |
| MEF2A |  | 0.017 | 8.02 | 0.386 | 6.99E-01 | 8.70E-01 | -6.127 |
| FUNDC2 |  | 0.015 | 7.899 | 0.386 | 6.99E-01 | 8.70E-01 | -6.127 |
| HLA-G |  | -0.031 | 10.459 | -0.387 | 6.99E-01 | 8.70E-01 | -6.127 |
| CAP2 |  | -0.033 | 8.433 | -0.386 | 6.99E-01 | 8.70E-01 | -6.128 |
| TMSB4Y |  | -0.026 | 5.271 | -0.386 | 7.00E-01 | 8.70E-01 | -6.128 |
| SNRPA |  | 0.02 | 9.748 | 0.385 | 7.00E-01 | 8.70E-01 | -6.128 |
| C14orf132 |  | 0.026 | 6.941 | 0.385 | 7.00E-01 | 8.70E-01 | -6.128 |
| ODF3L1 |  | 0.019 | 4.916 | 0.384 | 7.00E-01 | 8.70E-01 | -6.128 |
| RASEF |  | -0.026 | 6.059 | -0.385 | 7.00E-01 | 8.70E-01 | -6.128 |
| ZNF239 |  | -0.025 | 6.722 | -0.385 | 7.00E-01 | 8.70E-01 | -6.128 |
| METAP2 |  | -0.018 | 10.343 | -0.385 | 7.00E-01 | 8.70E-01 | -6.128 |
| MAN2A2 |  | 0.012 | 7.935 | 0.384 | 7.00E-01 | 8.70E-01 | -6.128 |
| GLTSCR1 |  | -0.023 | 7.775 | -0.385 | 7.01E-01 | 8.70E-01 | -6.128 |
| KLK5 |  | -0.023 | 5.332 | -0.385 | 7.01E-01 | 8.70E-01 | -6.128 |
| IRX4 |  | 0.029 | 5.537 | 0.383 | 7.01E-01 | 8.70E-01 | -6.128 |
| SNX6 |  | -0.014 | 10.084 | -0.384 | 7.01E-01 | 8.70E-01 | -6.128 |
| CLDN3 |  | 0.026 | 7.582 | 0.383 | 7.01E-01 | 8.70E-01 | -6.128 |
| MEFV |  | 0.021 | 6.275 | 0.383 | 7.01E-01 | 8.70E-01 | -6.128 |
| RPH3AL |  | -0.023 | 7.313 | -0.384 | 7.01E-01 | 8.70E-01 | -6.128 |
| ACYP1 |  | 0.016 | 9.145 | 0.383 | 7.01E-01 | 8.70E-01 | -6.128 |
| LAMP2 |  | 0.014 | 10.45 | 0.382 | 7.02E-01 | 8.71E-01 | -6.129 |
| ZNF536 |  | 0.022 | 4.85 | 0.382 | 7.02E-01 | 8.71E-01 | -6.129 |
| SPCS2 |  | 0.012 | 10.887 | 0.381 | 7.02E-01 | 8.71E-01 | -6.129 |
| ATPAF2 |  | -0.017 | 8.068 | -0.382 | 7.03E-01 | 8.71E-01 | -6.129 |
| TRIM31 |  | 0.02 | 4.738 | 0.381 | 7.03E-01 | 8.71E-01 | -6.129 |
| PHF5A |  | -0.022 | 8.437 | -0.382 | 7.03E-01 | 8.71E-01 | -6.129 |
| SLC35F3 |  | -0.026 | 5.562 | -0.382 | 7.03E-01 | 8.71E-01 | -6.129 |
| LHX1 |  | -0.027 | 6.088 | -0.381 | 7.03E-01 | 8.71E-01 | -6.129 |
| USP1 |  | -0.019 | 8.919 | -0.381 | 7.03E-01 | 8.71E-01 | -6.129 |
| GPR15 |  | -0.027 | 5.995 | -0.381 | 7.03E-01 | 8.71E-01 | -6.129 |
| P2RY2 |  | 0.022 | 7.707 | 0.38 | 7.03E-01 | 8.71E-01 | -6.13 |
| TBL1Y |  | -0.028 | 7.135 | -0.38 | 7.04E-01 | 8.71E-01 | -6.13 |
| ASNA1 |  | -0.02 | 8.4 | -0.38 | 7.04E-01 | 8.71E-01 | -6.13 |
| ZNF135 |  | 0.016 | 6.81 | 0.379 | 7.04E-01 | 8.71E-01 | -6.13 |
| TMEM40 |  | -0.024 | 8.953 | -0.38 | 7.04E-01 | 8.71E-01 | -6.13 |
| SYT5 |  | -0.022 | 5.359 | -0.379 | 7.05E-01 | 8.71E-01 | -6.13 |
| TEX11 |  | -0.023 | 4.932 | -0.379 | 7.05E-01 | 8.71E-01 | -6.13 |
| UBASH3A |  | 0.022 | 5.669 | 0.378 | 7.05E-01 | 8.71E-01 | -6.13 |
| FAM9A |  | 0.021 | 4.745 | 0.378 | 7.05E-01 | 8.71E-01 | -6.13 |
| PYCRL |  | -0.021 | 6.373 | -0.379 | 7.05E-01 | 8.71E-01 | -6.13 |
| MPHOSPH9 |  | 0.015 | 7.215 | 0.378 | 7.05E-01 | 8.71E-01 | -6.13 |
| PPP2R5B |  | 0.017 | 8 | 0.377 | 7.05E-01 | 8.72E-01 | -6.131 |
| PXMP2 |  | 0.014 | 7.751 | 0.377 | 7.06E-01 | 8.72E-01 | -6.131 |
| HIF1AN |  | 0.021 | 7.923 | 0.377 | 7.06E-01 | 8.72E-01 | -6.131 |
| PRPSAP1 |  | -0.013 | 10.355 | -0.377 | 7.06E-01 | 8.72E-01 | -6.131 |
| PPP4R2 |  | -0.024 | 7.882 | -0.377 | 7.06E-01 | 8.72E-01 | -6.131 |
| NUP88 |  | 0.013 | 9.718 | 0.376 | 7.06E-01 | 8.72E-01 | -6.131 |
| C6orf201 |  | 0.019 | 4.812 | 0.376 | 7.06E-01 | 8.72E-01 | -6.131 |
| FSD1 |  | -0.021 | 6.298 | -0.377 | 7.06E-01 | 8.72E-01 | -6.131 |
| LTA |  | -0.023 | 6.125 | -0.377 | 7.06E-01 | 8.72E-01 | -6.131 |
| VPS54 |  | -0.015 | 7.768 | -0.376 | 7.07E-01 | 8.72E-01 | -6.131 |
| LIM2 |  | 0.025 | 5.35 | 0.375 | 7.07E-01 | 8.72E-01 | -6.131 |
| GCG |  | 0.019 | 4.88 | 0.375 | 7.07E-01 | 8.72E-01 | -6.131 |
| WFDC9 |  | -0.024 | 5.346 | -0.376 | 7.07E-01 | 8.72E-01 | -6.131 |
| TCEA2 |  | -0.015 | 7.53 | -0.376 | 7.07E-01 | 8.72E-01 | -6.132 |
| WBP2 |  | -0.018 | 10.932 | -0.375 | 7.08E-01 | 8.72E-01 | -6.132 |
| RAB5C |  | -0.019 | 9.864 | -0.375 | 7.08E-01 | 8.72E-01 | -6.132 |
| KCNQ5 |  | 0.017 | 5.454 | 0.374 | 7.08E-01 | 8.72E-01 | -6.132 |
| TNFRSF17 |  | -0.027 | 5.584 | -0.375 | 7.08E-01 | 8.72E-01 | -6.132 |
| DHH |  | 0.024 | 6.468 | 0.374 | 7.08E-01 | 8.72E-01 | -6.132 |
| TEAD3 |  | 0.025 | 9.15 | 0.374 | 7.08E-01 | 8.72E-01 | -6.132 |
| GOLT1A |  | 0.022 | 5.982 | 0.373 | 7.08E-01 | 8.72E-01 | -6.132 |
| PALMD |  | 0.024 | 7.22 | 0.373 | 7.09E-01 | 8.72E-01 | -6.132 |
| DOK2 |  | -0.021 | 6.722 | -0.374 | 7.09E-01 | 8.72E-01 | -6.132 |
| TLK1 |  | 0.021 | 7.687 | 0.373 | 7.09E-01 | 8.73E-01 | -6.132 |
| RPGRIP1 |  | 0.02 | 6.555 | 0.371 | 7.10E-01 | 8.73E-01 | -6.133 |
| EMX2 |  | -0.034 | 5.381 | -0.372 | 7.10E-01 | 8.74E-01 | -6.133 |
| CTNNA2 |  | 0.017 | 4.858 | 0.37 | 7.11E-01 | 8.74E-01 | -6.133 |
| ANKRD7 |  | 0.023 | 4.922 | 0.369 | 7.11E-01 | 8.75E-01 | -6.133 |
| KIF7 |  | 0.02 | 5.957 | 0.369 | 7.11E-01 | 8.75E-01 | -6.133 |
| DUSP12 |  | 0.014 | 10.122 | 0.369 | 7.12E-01 | 8.75E-01 | -6.134 |
| EIF2B5 |  | -0.013 | 8.9 | -0.37 | 7.12E-01 | 8.75E-01 | -6.134 |
| CABP4 |  | -0.015 | 6.507 | -0.37 | 7.12E-01 | 8.75E-01 | -6.134 |
| SERPINI2 |  | 0.022 | 5.249 | 0.368 | 7.12E-01 | 8.75E-01 | -6.134 |
| C10orf62 |  | 0.024 | 5.037 | 0.368 | 7.13E-01 | 8.75E-01 | -6.134 |
| IL2RB |  | 0.04 | 9.213 | 0.368 | 7.13E-01 | 8.75E-01 | -6.134 |
| MRPL3 |  | -0.013 | 9.867 | -0.368 | 7.13E-01 | 8.75E-01 | -6.134 |
| TXNL4B |  | 0.014 | 6.801 | 0.367 | 7.13E-01 | 8.75E-01 | -6.134 |
| KCNK12 |  | -0.043 | 7.101 | -0.368 | 7.13E-01 | 8.75E-01 | -6.134 |
| KLRB1 |  | 0.028 | 6.806 | 0.367 | 7.13E-01 | 8.75E-01 | -6.134 |
| HGS |  | -0.021 | 10.307 | -0.368 | 7.13E-01 | 8.75E-01 | -6.134 |
| B3GALT2 |  | 0.019 | 5.131 | 0.366 | 7.14E-01 | 8.76E-01 | -6.135 |
| CA3 |  | -0.024 | 4.849 | -0.367 | 7.14E-01 | 8.76E-01 | -6.135 |
| PSKH2 |  | -0.02 | 5.358 | -0.367 | 7.14E-01 | 8.76E-01 | -6.135 |
| VAMP3 |  | 0.015 | 10.256 | 0.365 | 7.14E-01 | 8.76E-01 | -6.135 |
| CDH20 |  | 0.022 | 4.782 | 0.364 | 7.16E-01 | 8.77E-01 | -6.135 |
| OASL |  | 0.034 | 7.648 | 0.364 | 7.16E-01 | 8.77E-01 | -6.135 |
| CSMD2 |  | 0.017 | 5.23 | 0.363 | 7.16E-01 | 8.78E-01 | -6.136 |
| SLC6A5 |  | -0.022 | 5.681 | -0.364 | 7.16E-01 | 8.78E-01 | -6.136 |
| HNMT |  | -0.017 | 7.418 | -0.363 | 7.17E-01 | 8.78E-01 | -6.136 |
| FOXM1 |  | -0.023 | 7.82 | -0.363 | 7.17E-01 | 8.78E-01 | -6.136 |
| KLC2 |  | -0.024 | 6.9 | -0.362 | 7.17E-01 | 8.79E-01 | -6.136 |
| IGSF9 |  | -0.025 | 6.374 | -0.362 | 7.17E-01 | 8.79E-01 | -6.136 |
| SATB1 |  | 0.019 | 7.332 | 0.361 | 7.17E-01 | 8.79E-01 | -6.136 |
| KRTAP1-5 |  | -0.03 | 5.674 | -0.362 | 7.18E-01 | 8.79E-01 | -6.136 |
| CNNM1 |  | 0.02 | 5.273 | 0.361 | 7.18E-01 | 8.79E-01 | -6.136 |
| FAM9B |  | 0.023 | 4.779 | 0.36 | 7.18E-01 | 8.79E-01 | -6.137 |
| ZZZ3 |  | -0.016 | 8.405 | -0.361 | 7.18E-01 | 8.79E-01 | -6.137 |
| UCP3 |  | 0.026 | 7.068 | 0.359 | 7.19E-01 | 8.79E-01 | -6.137 |
| B4GALT6 |  | 0.015 | 5.429 | 0.359 | 7.19E-01 | 8.80E-01 | -6.137 |
| NDUFA11 |  | -0.015 | 11.572 | -0.359 | 7.19E-01 | 8.80E-01 | -6.137 |
| PHF3 |  | 0.016 | 9.182 | 0.358 | 7.20E-01 | 8.80E-01 | -6.137 |
| HYOU1 |  | -0.024 | 8.585 | -0.359 | 7.20E-01 | 8.80E-01 | -6.137 |
| CLSTN2 |  | 0.029 | 5.746 | 0.358 | 7.20E-01 | 8.80E-01 | -6.137 |
| FOXP3 |  | 0.02 | 5.437 | 0.357 | 7.20E-01 | 8.80E-01 | -6.138 |
| GOLGA1 |  | -0.017 | 7.279 | -0.358 | 7.20E-01 | 8.80E-01 | -6.138 |
| FDX1 |  | -0.023 | 9.099 | -0.358 | 7.20E-01 | 8.80E-01 | -6.138 |
| KIRREL |  | 0.019 | 6.907 | 0.357 | 7.21E-01 | 8.80E-01 | -6.138 |
| LRPPRC |  | -0.014 | 8.645 | -0.357 | 7.21E-01 | 8.80E-01 | -6.138 |
| PZP |  | -0.024 | 5.27 | -0.357 | 7.21E-01 | 8.80E-01 | -6.138 |
| FKBP7 |  | 0.021 | 7.264 | 0.356 | 7.21E-01 | 8.80E-01 | -6.138 |
| TNFAIP6 |  | -0.03 | 6.641 | -0.357 | 7.21E-01 | 8.80E-01 | -6.138 |
| MSMB |  | 0.018 | 4.97 | 0.356 | 7.21E-01 | 8.80E-01 | -6.138 |
| HLA-F |  | 0.021 | 10.59 | 0.356 | 7.21E-01 | 8.80E-01 | -6.138 |
| TAGAP |  | 0.02 | 6.845 | 0.356 | 7.21E-01 | 8.80E-01 | -6.138 |
| ZNF121 |  | -0.023 | 5.683 | -0.357 | 7.21E-01 | 8.80E-01 | -6.138 |
| CALCR |  | -0.024 | 5.444 | -0.357 | 7.21E-01 | 8.80E-01 | -6.138 |
| EPHB1 |  | -0.019 | 5.394 | -0.357 | 7.22E-01 | 8.80E-01 | -6.138 |
| ANKRD30A |  | 0.02 | 4.81 | 0.354 | 7.22E-01 | 8.81E-01 | -6.139 |
| PHC2 |  | -0.018 | 8.194 | -0.355 | 7.23E-01 | 8.81E-01 | -6.139 |
| DNALI1 |  | 0.023 | 6.515 | 0.354 | 7.23E-01 | 8.81E-01 | -6.139 |
| MBD4 |  | 0.016 | 9.458 | 0.353 | 7.23E-01 | 8.81E-01 | -6.139 |
| SAGE1 |  | 0.021 | 4.799 | 0.353 | 7.24E-01 | 8.81E-01 | -6.139 |
| FXYD6 |  | 0.019 | 8.121 | 0.353 | 7.24E-01 | 8.81E-01 | -6.139 |
| ADAMTS4 |  | -0.024 | 6.628 | -0.354 | 7.24E-01 | 8.81E-01 | -6.139 |
| PTPN6 |  | -0.014 | 8.139 | -0.353 | 7.24E-01 | 8.82E-01 | -6.139 |
| MAP4 |  | 0.013 | 7.452 | 0.352 | 7.24E-01 | 8.82E-01 | -6.14 |
| CMIP |  | -0.021 | 8.443 | -0.353 | 7.25E-01 | 8.82E-01 | -6.14 |
| TNFRSF1A |  | 0.025 | 9.971 | 0.351 | 7.25E-01 | 8.82E-01 | -6.14 |
| ITPKC |  | -0.017 | 8.826 | -0.352 | 7.25E-01 | 8.82E-01 | -6.14 |
| GATA5 |  | 0.02 | 5.288 | 0.35 | 7.25E-01 | 8.83E-01 | -6.14 |
| COX6B1 |  | 0.011 | 12.475 | 0.35 | 7.26E-01 | 8.83E-01 | -6.14 |
| CUL7 |  | 0.016 | 7.701 | 0.35 | 7.26E-01 | 8.83E-01 | -6.14 |
| TTC17 |  | -0.018 | 7.874 | -0.351 | 7.26E-01 | 8.83E-01 | -6.14 |
| GTPBP2 |  | -0.016 | 8.055 | -0.351 | 7.26E-01 | 8.83E-01 | -6.14 |
| VEPH1 |  | -0.026 | 5.752 | -0.35 | 7.27E-01 | 8.83E-01 | -6.14 |
| TCF19 |  | 0.018 | 6.117 | 0.349 | 7.27E-01 | 8.83E-01 | -6.141 |
| DMGDH |  | 0.017 | 4.885 | 0.348 | 7.27E-01 | 8.84E-01 | -6.141 |
| PNPO |  | 0.018 | 8.847 | 0.348 | 7.27E-01 | 8.84E-01 | -6.141 |
| AFMID |  | -0.024 | 6.232 | -0.348 | 7.28E-01 | 8.85E-01 | -6.141 |
| PRSS35 |  | -0.026 | 5.903 | -0.347 | 7.29E-01 | 8.85E-01 | -6.141 |
| PEX13 |  | 0.011 | 7.801 | 0.346 | 7.29E-01 | 8.85E-01 | -6.141 |
| KLHDC3 |  | -0.015 | 9.565 | -0.347 | 7.29E-01 | 8.85E-01 | -6.142 |
| SCG3 |  | 0.019 | 4.835 | 0.345 | 7.29E-01 | 8.85E-01 | -6.142 |
| KRTAP3-2 |  | -0.023 | 4.903 | -0.346 | 7.29E-01 | 8.85E-01 | -6.142 |
| CDKN2B |  | 0.02 | 6.233 | 0.345 | 7.30E-01 | 8.85E-01 | -6.142 |
| IER5 |  | 0.017 | 11.161 | 0.344 | 7.30E-01 | 8.86E-01 | -6.142 |
| CLCN4 |  | -0.02 | 5.716 | -0.344 | 7.31E-01 | 8.87E-01 | -6.143 |
| ATP6V0A1 |  | 0.018 | 10.375 | 0.342 | 7.32E-01 | 8.87E-01 | -6.143 |
| SPAG6 |  | 0.02 | 4.867 | 0.342 | 7.32E-01 | 8.88E-01 | -6.143 |
| PROK2 |  | 0.03 | 7.156 | 0.341 | 7.32E-01 | 8.88E-01 | -6.143 |
| POLR2I |  | -0.014 | 9.935 | -0.342 | 7.32E-01 | 8.88E-01 | -6.143 |
| CALCRL |  | 0.023 | 6.101 | 0.341 | 7.32E-01 | 8.88E-01 | -6.143 |
| P2RX5 |  | 0.02 | 5.992 | 0.341 | 7.32E-01 | 8.88E-01 | -6.143 |
| HAMP |  | -0.021 | 7.101 | -0.342 | 7.33E-01 | 8.88E-01 | -6.143 |
| PPP1CB |  | 0.017 | 10.355 | 0.34 | 7.33E-01 | 8.88E-01 | -6.143 |
| SLC35A2 |  | 0.011 | 8.379 | 0.34 | 7.33E-01 | 8.88E-01 | -6.143 |
| HAO1 |  | -0.019 | 5.133 | -0.341 | 7.34E-01 | 8.88E-01 | -6.144 |
| PRKCSH |  | -0.019 | 9.482 | -0.34 | 7.34E-01 | 8.88E-01 | -6.144 |
| MAGEH1 |  | 0.017 | 9.013 | 0.339 | 7.34E-01 | 8.88E-01 | -6.144 |
| LBR |  | 0.016 | 10.278 | 0.339 | 7.34E-01 | 8.88E-01 | -6.144 |
| GALNT8 |  | 0.02 | 4.623 | 0.338 | 7.35E-01 | 8.89E-01 | -6.144 |
| CALN1 |  | -0.013 | 5.602 | -0.339 | 7.35E-01 | 8.89E-01 | -6.144 |
| PDZK1 |  | 0.025 | 5.767 | 0.338 | 7.35E-01 | 8.89E-01 | -6.144 |
| GPRC5B |  | -0.025 | 7.405 | -0.339 | 7.35E-01 | 8.89E-01 | -6.144 |
| ATOH8 |  | -0.024 | 7.102 | -0.339 | 7.35E-01 | 8.89E-01 | -6.144 |
| SMTN |  | 0.022 | 7.234 | 0.337 | 7.35E-01 | 8.89E-01 | -6.144 |
| DSG2 |  | 0.026 | 6.774 | 0.337 | 7.35E-01 | 8.89E-01 | -6.144 |
| HBQ1 |  | 0.027 | 8.603 | 0.337 | 7.36E-01 | 8.89E-01 | -6.144 |
| PLA2G4C |  | 0.024 | 7.455 | 0.336 | 7.36E-01 | 8.89E-01 | -6.145 |
| RAB3D |  | -0.017 | 6.988 | -0.337 | 7.36E-01 | 8.89E-01 | -6.145 |
| CYP27A1 |  | 0.024 | 7.84 | 0.336 | 7.36E-01 | 8.89E-01 | -6.145 |
| NDUFB7 |  | 0.014 | 11.083 | 0.336 | 7.36E-01 | 8.89E-01 | -6.145 |
| IFITM1 |  | 0.02 | 12.24 | 0.335 | 7.37E-01 | 8.90E-01 | -6.145 |
| KIAA1468 |  | -0.015 | 8.246 | -0.336 | 7.37E-01 | 8.90E-01 | -6.145 |
| SPOCK2 |  | -0.022 | 7.569 | -0.336 | 7.37E-01 | 8.90E-01 | -6.145 |
| ST8SIA3 |  | 0.016 | 5.417 | 0.334 | 7.38E-01 | 8.90E-01 | -6.145 |
| ZNF367 |  | -0.018 | 5.651 | -0.335 | 7.38E-01 | 8.91E-01 | -6.146 |
| THUMPD3 |  | -0.012 | 8.035 | -0.334 | 7.38E-01 | 8.91E-01 | -6.146 |
| GSTM5 |  | -0.02 | 6.235 | -0.334 | 7.39E-01 | 8.91E-01 | -6.146 |
| CASP10 |  | 0.014 | 6.794 | 0.333 | 7.39E-01 | 8.91E-01 | -6.146 |
| IFNAR2 |  | 0.015 | 7.607 | 0.332 | 7.39E-01 | 8.91E-01 | -6.146 |
| ICMT |  | 0.017 | 8.031 | 0.332 | 7.39E-01 | 8.91E-01 | -6.146 |
| PGK2 |  | 0.014 | 4.813 | 0.332 | 7.39E-01 | 8.91E-01 | -6.146 |
| UBE2H |  | -0.018 | 9.345 | -0.333 | 7.39E-01 | 8.91E-01 | -6.146 |
| KIAA1143 |  | 0.014 | 7.958 | 0.332 | 7.39E-01 | 8.91E-01 | -6.146 |
| HIST1H3A |  | 0.019 | 8.835 | 0.332 | 7.40E-01 | 8.91E-01 | -6.146 |
| KBTBD2 |  | -0.013 | 9.565 | -0.333 | 7.40E-01 | 8.91E-01 | -6.146 |
| POLL |  | 0.014 | 7.557 | 0.332 | 7.40E-01 | 8.91E-01 | -6.146 |
| WBSCR28 |  | -0.021 | 4.878 | -0.332 | 7.40E-01 | 8.91E-01 | -6.146 |
| DSC1 |  | -0.026 | 5.425 | -0.332 | 7.40E-01 | 8.91E-01 | -6.146 |
| MAPKAPK3 |  | 0.015 | 9.613 | 0.331 | 7.40E-01 | 8.91E-01 | -6.146 |
| PGAP1 |  | -0.018 | 5.863 | -0.332 | 7.40E-01 | 8.91E-01 | -6.146 |
| RGS5 |  | -0.023 | 6.246 | -0.331 | 7.41E-01 | 8.91E-01 | -6.147 |
| CRISP2 |  | 0.021 | 5.456 | 0.33 | 7.41E-01 | 8.91E-01 | -6.147 |
| CLEC4E |  | 0.02 | 6.394 | 0.33 | 7.41E-01 | 8.91E-01 | -6.147 |
| PPP1R3B |  | 0.018 | 8.296 | 0.329 | 7.41E-01 | 8.91E-01 | -6.147 |
| CPNE1 |  | -0.021 | 9.895 | -0.33 | 7.41E-01 | 8.91E-01 | -6.147 |
| SLIT3 |  | -0.019 | 7.905 | -0.33 | 7.42E-01 | 8.92E-01 | -6.147 |
| TOPBP1 |  | 0.014 | 8.863 | 0.329 | 7.42E-01 | 8.92E-01 | -6.147 |
| CDKL5 |  | 0.019 | 7.324 | 0.328 | 7.43E-01 | 8.92E-01 | -6.147 |
| OLIG3 |  | -0.018 | 4.787 | -0.328 | 7.43E-01 | 8.92E-01 | -6.147 |
| TBL1XR1 |  | -0.017 | 9.062 | -0.327 | 7.43E-01 | 8.93E-01 | -6.148 |
| ST6GALNAC2 |  | -0.018 | 8.045 | -0.327 | 7.44E-01 | 8.93E-01 | -6.148 |
| OGN |  | 0.029 | 6.172 | 0.325 | 7.44E-01 | 8.94E-01 | -6.148 |
| POLR1D |  | -0.015 | 9.82 | -0.326 | 7.44E-01 | 8.94E-01 | -6.148 |
| UNG |  | -0.014 | 8.622 | -0.326 | 7.45E-01 | 8.94E-01 | -6.148 |
| EHMT1 |  | 0.016 | 7.358 | 0.325 | 7.45E-01 | 8.94E-01 | -6.148 |
| SULF2 |  | 0.02 | 9.579 | 0.324 | 7.45E-01 | 8.94E-01 | -6.148 |
| NOV |  | -0.025 | 6.647 | -0.324 | 7.46E-01 | 8.95E-01 | -6.149 |
| CTAGE1 |  | -0.012 | 5.853 | -0.323 | 7.47E-01 | 8.95E-01 | -6.149 |
| MNDA |  | -0.021 | 8.527 | -0.323 | 7.47E-01 | 8.95E-01 | -6.149 |
| GHRHR |  | -0.018 | 5.814 | -0.323 | 7.47E-01 | 8.95E-01 | -6.149 |
| ZNF436 |  | -0.014 | 7.173 | -0.323 | 7.47E-01 | 8.95E-01 | -6.149 |
| ZNF221 |  | -0.016 | 5.243 | -0.323 | 7.47E-01 | 8.95E-01 | -6.149 |
| CLEC10A |  | -0.021 | 5.986 | -0.323 | 7.47E-01 | 8.96E-01 | -6.149 |
| HPCAL4 |  | -0.019 | 5.337 | -0.321 | 7.48E-01 | 8.97E-01 | -6.15 |
| RBMX2 |  | -0.014 | 7.822 | -0.321 | 7.48E-01 | 8.97E-01 | -6.15 |
| IL13RA2 |  | -0.039 | 5.748 | -0.32 | 7.49E-01 | 8.97E-01 | -6.15 |
| COG8 |  | 0.01 | 7.91 | 0.319 | 7.49E-01 | 8.97E-01 | -6.15 |
| ZNF35 |  | 0.014 | 7.272 | 0.319 | 7.49E-01 | 8.98E-01 | -6.15 |
| ATXN7 |  | -0.021 | 6.571 | -0.319 | 7.50E-01 | 8.98E-01 | -6.15 |
| SAMD8 |  | -0.014 | 7.313 | -0.319 | 7.50E-01 | 8.98E-01 | -6.15 |
| DBP |  | 0.019 | 7.72 | 0.318 | 7.50E-01 | 8.98E-01 | -6.15 |
[truncated: 78,610 more chars]
