# Supplementary material for: Identifying molecular subgroups of patients with preeclampsia through bioinformatics
Source: Front Cardiovasc Med. 2024 Jun 3;11:1367578. doi: 10.3389/fcvm.2024.1367578 (PMC11180819; doi:10.3389/fcvm.2024.1367578)
Supplement: Supplementary file 3 [file Table3.docx]

**Supplementary Table S3.** Differential expression genes between subgroup Ⅱ and control group

| Gene | meanDiff | AveExpr | t | P.Value | adj.P.Val | B |
| --- | --- | --- | --- | --- | --- | --- |
| ETNK1 | -0.722 | 7.553 | -9.111 | 0.00E+00 | 0.00E+00 | 26.291 |
| CLTC | -0.865 | 9.374 | -8.142 | 0.00E+00 | 0.00E+00 | 20.721 |
| FNDC3A | -1.267 | 9.305 | -8.103 | 0.00E+00 | 0.00E+00 | 20.501 |
| BTAF1 | -0.951 | 8.491 | -8 | 0.00E+00 | 0.00E+00 | 19.921 |
| DHX15 | -0.705 | 10.994 | -7.834 | 0.00E+00 | 0.00E+00 | 18.996 |
| RANBP6 | -0.927 | 7.474 | -7.786 | 0.00E+00 | 0.00E+00 | 18.735 |
| USP38 | -0.983 | 8.593 | -7.752 | 0.00E+00 | 0.00E+00 | 18.548 |
| PPP1R15B | -1.072 | 8.513 | -7.73 | 0.00E+00 | 0.00E+00 | 18.424 |
| PHTF2 | -0.868 | 7.202 | -7.692 | 0.00E+00 | 0.00E+00 | 18.213 |
| PPM1D | -0.758 | 8.857 | -7.635 | 0.00E+00 | 0.00E+00 | 17.905 |
| SEC24D | -0.905 | 9.004 | -7.615 | 0.00E+00 | 0.00E+00 | 17.793 |
| CCNT2 | -0.729 | 7.515 | -7.567 | 0.00E+00 | 0.00E+00 | 17.528 |
| OSBPL11 | -0.588 | 8.328 | -7.416 | 0.00E+00 | 0.00E+00 | 16.71 |
| VPS4B | -0.618 | 10.256 | -7.362 | 0.00E+00 | 0.00E+00 | 16.417 |
| SNAP23 | -1.137 | 8.747 | -7.298 | 0.00E+00 | 0.00E+00 | 16.074 |
| CHSY1 | -0.82 | 11.688 | -7.259 | 0.00E+00 | 0.00E+00 | 15.865 |
| PAIP2 | -0.617 | 10.081 | -7.248 | 0.00E+00 | 0.00E+00 | 15.806 |
| SMAD4 | -0.614 | 9.632 | -7.19 | 0.00E+00 | 0.00E+00 | 15.496 |
| ACTR3 | -0.9 | 10.59 | -7.155 | 0.00E+00 | 0.00E+00 | 15.31 |
| RBM7 | -0.921 | 8.627 | -7.11 | 0.00E+00 | 0.00E+00 | 15.076 |
| RB1CC1 | -0.692 | 9.584 | -7.018 | 0.00E+00 | 0.00E+00 | 14.589 |
| FYTTD1 | -0.695 | 9.183 | -7.013 | 0.00E+00 | 0.00E+00 | 14.561 |
| SDHD | -0.7 | 10.219 | -6.905 | 0.00E+00 | 0.00E+00 | 13.995 |
| SYNJ1 | -0.746 | 7.186 | -6.88 | 0.00E+00 | 0.00E+00 | 13.866 |
| ZNF140 | -0.659 | 6.746 | -6.866 | 0.00E+00 | 0.00E+00 | 13.793 |
| ADNP | -0.48 | 9.332 | -6.854 | 0.00E+00 | 0.00E+00 | 13.735 |
| CHCHD5 | 0.752 | 8.996 | 6.842 | 0.00E+00 | 0.00E+00 | 13.675 |
| APPBP2 | -0.595 | 8.339 | -6.804 | 0.00E+00 | 0.00E+00 | 13.475 |
| GALNT3 | -1.011 | 7.183 | -6.779 | 0.00E+00 | 0.00E+00 | 13.343 |
| RAB21 | -0.535 | 9.116 | -6.743 | 0.00E+00 | 0.00E+00 | 13.16 |
| STX7 | -0.685 | 8.617 | -6.706 | 0.00E+00 | 0.00E+00 | 12.967 |
| MRPL28 | 0.998 | 9.42 | 6.663 | 0.00E+00 | 0.00E+00 | 12.751 |
| RASA1 | -0.784 | 10.196 | -6.636 | 0.00E+00 | 0.00E+00 | 12.609 |
| ATOX1 | 0.562 | 10.987 | 6.623 | 0.00E+00 | 0.00E+00 | 12.549 |
| ZNF600 | -0.646 | 8.009 | -6.617 | 0.00E+00 | 0.00E+00 | 12.511 |
| NT5C2 | -0.633 | 9.349 | -6.593 | 0.00E+00 | 0.00E+00 | 12.393 |
| SLK | -0.667 | 9.292 | -6.581 | 0.00E+00 | 0.00E+00 | 12.328 |
| GOLPH3 | -0.639 | 10.494 | -6.56 | 0.00E+00 | 0.00E+00 | 12.226 |
| NMD3 | -0.872 | 8.355 | -6.56 | 0.00E+00 | 0.00E+00 | 12.225 |
| CAPZA1 | -1.11 | 9 | -6.544 | 0.00E+00 | 0.00E+00 | 12.141 |
| PHF3 | -0.615 | 9.104 | -6.539 | 0.00E+00 | 0.00E+00 | 12.118 |
| PPP4R2 | -0.827 | 7.797 | -6.532 | 0.00E+00 | 0.00E+00 | 12.082 |
| ACBD3 | -0.6 | 9.605 | -6.525 | 0.00E+00 | 0.00E+00 | 12.045 |
| DPYD | -0.809 | 7.327 | -6.515 | 0.00E+00 | 0.00E+00 | 11.998 |
| ELF1 | -1.009 | 9.241 | -6.51 | 0.00E+00 | 0.00E+00 | 11.97 |
| ACVR1 | -0.534 | 9.445 | -6.507 | 0.00E+00 | 0.00E+00 | 11.958 |
| SCYL2 | -0.764 | 8.928 | -6.498 | 0.00E+00 | 0.00E+00 | 11.911 |
| CTSO | -0.901 | 8.569 | -6.473 | 0.00E+00 | 0.00E+00 | 11.784 |
| GMFB | -0.713 | 8.824 | -6.456 | 0.00E+00 | 0.00E+00 | 11.698 |
| MTMR6 | -0.765 | 7.652 | -6.444 | 0.00E+00 | 0.00E+00 | 11.641 |
| TCEB2 | 0.571 | 11.036 | 6.438 | 0.00E+00 | 0.00E+00 | 11.615 |
| TMEM33 | -0.534 | 7.61 | -6.428 | 0.00E+00 | 0.00E+00 | 11.561 |
| ANKRD10 | -0.808 | 8.741 | -6.427 | 0.00E+00 | 0.00E+00 | 11.556 |
| IGSF8 | 0.808 | 7.528 | 6.422 | 0.00E+00 | 0.00E+00 | 11.532 |
| MINPP1 | -0.864 | 8.596 | -6.401 | 0.00E+00 | 0.00E+00 | 11.423 |
| KIF17 | 0.823 | 6.803 | 6.394 | 0.00E+00 | 0.00E+00 | 11.395 |
| SPG20 | -0.707 | 7.907 | -6.391 | 0.00E+00 | 0.00E+00 | 11.373 |
| SLC33A1 | -0.666 | 7.841 | -6.385 | 0.00E+00 | 0.00E+00 | 11.342 |
| C19orf24 | 0.563 | 9.164 | 6.36 | 0.00E+00 | 0.00E+00 | 11.223 |
| PIGN | -0.656 | 7.652 | -6.355 | 0.00E+00 | 0.00E+00 | 11.193 |
| EEF1A1 | -1.294 | 14.006 | -6.338 | 0.00E+00 | 0.00E+00 | 11.111 |
| LAPTM4A | -0.638 | 12.264 | -6.335 | 0.00E+00 | 0.00E+00 | 11.098 |
| ATP2C1 | -0.582 | 8.573 | -6.333 | 0.00E+00 | 0.00E+00 | 11.085 |
| STAG1 | -0.762 | 7.637 | -6.329 | 0.00E+00 | 0.00E+00 | 11.067 |
| SIAH1 | -0.628 | 9.222 | -6.324 | 0.00E+00 | 0.00E+00 | 11.039 |
| MALT1 | -0.763 | 6.616 | -6.318 | 0.00E+00 | 0.00E+00 | 11.013 |
| ZNRF2 | -0.457 | 7.257 | -6.3 | 0.00E+00 | 0.00E+00 | 10.92 |
| KRTCAP2 | 0.593 | 11.835 | 6.284 | 0.00E+00 | 0.00E+00 | 10.845 |
| IQGAP1 | -0.745 | 11.199 | -6.284 | 0.00E+00 | 0.00E+00 | 10.843 |
| KIF5B | -0.672 | 9.318 | -6.284 | 0.00E+00 | 0.00E+00 | 10.841 |
| NCK1 | -0.77 | 8.854 | -6.274 | 0.00E+00 | 0.00E+00 | 10.793 |
| ARID4A | -0.525 | 7.342 | -6.269 | 0.00E+00 | 0.00E+00 | 10.767 |
| ZNF226 | -0.488 | 8.25 | -6.26 | 0.00E+00 | 0.00E+00 | 10.724 |
| C12orf10 | 0.586 | 9.633 | 6.256 | 0.00E+00 | 0.00E+00 | 10.709 |
| C9orf116 | 0.658 | 6.809 | 6.244 | 0.00E+00 | 0.00E+00 | 10.651 |
| SLC35A5 | -0.707 | 9.099 | -6.245 | 0.00E+00 | 0.00E+00 | 10.648 |
| SOAT1 | -0.764 | 7.526 | -6.235 | 0.00E+00 | 0.00E+00 | 10.6 |
| ERAL1 | 0.516 | 9.403 | 6.233 | 0.00E+00 | 0.00E+00 | 10.595 |
| ZNF431 | -0.545 | 6.026 | -6.218 | 0.00E+00 | 0.00E+00 | 10.516 |
| EIF2AK3 | -0.59 | 8.069 | -6.207 | 0.00E+00 | 0.00E+00 | 10.466 |
| PPP1CB | -0.625 | 10.275 | -6.202 | 0.00E+00 | 0.00E+00 | 10.439 |
| RAB18 | -0.643 | 8.807 | -6.195 | 0.00E+00 | 0.00E+00 | 10.403 |
| UBE2V2 | -0.761 | 8.351 | -6.192 | 0.00E+00 | 0.00E+00 | 10.389 |
| JAK2 | -0.583 | 8.239 | -6.189 | 0.00E+00 | 0.00E+00 | 10.373 |
| TLK1 | -0.714 | 7.595 | -6.176 | 0.00E+00 | 0.00E+00 | 10.313 |
| CCS | 0.467 | 9.007 | 6.173 | 0.00E+00 | 0.00E+00 | 10.303 |
| DEK | -0.771 | 10.704 | -6.173 | 0.00E+00 | 0.00E+00 | 10.298 |
| ABI1 | -0.494 | 8.507 | -6.172 | 0.00E+00 | 0.00E+00 | 10.292 |
| DNAJB4 | -0.914 | 7.412 | -6.159 | 0.00E+00 | 0.00E+00 | 10.228 |
| IL1R1 | -0.843 | 8.942 | -6.112 | 0.00E+00 | 0.00E+00 | 10.001 |
| SLC39A10 | -0.715 | 8.401 | -6.099 | 0.00E+00 | 0.00E+00 | 9.937 |
| C1orf27 | -0.556 | 7.779 | -6.087 | 0.00E+00 | 0.00E+00 | 9.877 |
| EEA1 | -0.578 | 7.752 | -6.066 | 0.00E+00 | 0.00E+00 | 9.778 |
| B3GNT5 | -0.781 | 8.411 | -6.061 | 0.00E+00 | 0.00E+00 | 9.751 |
| NARS | -0.718 | 10.838 | -6.048 | 0.00E+00 | 0.00E+00 | 9.689 |
| TCERG1 | -0.473 | 8.475 | -6.021 | 0.00E+00 | 0.00E+00 | 9.559 |
| NFE2L2 | -0.565 | 9.538 | -5.997 | 0.00E+00 | 0.00E+00 | 9.445 |
| DNAJC10 | -0.408 | 8.65 | -5.991 | 0.00E+00 | 0.00E+00 | 9.417 |
| GNAI3 | -0.508 | 8.549 | -5.991 | 0.00E+00 | 0.00E+00 | 9.416 |
| C21orf91 | -0.515 | 7.674 | -5.959 | 0.00E+00 | 0.00E+00 | 9.261 |
| MAT2B | -0.478 | 9.92 | -5.953 | 0.00E+00 | 0.00E+00 | 9.232 |
| GCA | -0.727 | 9.942 | -5.948 | 0.00E+00 | 0.00E+00 | 9.209 |
| GOLGA4 | -0.679 | 9.35 | -5.945 | 0.00E+00 | 0.00E+00 | 9.194 |
| DOCK8 | -0.616 | 8.302 | -5.942 | 0.00E+00 | 0.00E+00 | 9.179 |
| PRKRA | -0.556 | 9.238 | -5.939 | 0.00E+00 | 0.00E+00 | 9.166 |
| PIGH | -0.549 | 9.018 | -5.93 | 0.00E+00 | 0.00E+00 | 9.123 |
| ZNF593 | 0.627 | 9.887 | 5.909 | 0.00E+00 | 0.00E+00 | 9.026 |
| PPP1R12A | -0.76 | 8.584 | -5.906 | 0.00E+00 | 0.00E+00 | 9.008 |
| WRB | -0.651 | 8.696 | -5.901 | 0.00E+00 | 0.00E+00 | 8.986 |
| SEMA3C | -0.999 | 7.076 | -5.883 | 0.00E+00 | 0.00E+00 | 8.901 |
| CNOT8 | -0.587 | 9.092 | -5.88 | 0.00E+00 | 0.00E+00 | 8.888 |
| SCOC | -0.814 | 8.457 | -5.879 | 0.00E+00 | 0.00E+00 | 8.883 |
| TMEM9 | 0.512 | 8.684 | 5.877 | 0.00E+00 | 0.00E+00 | 8.878 |
| ARID4B | -0.403 | 8.088 | -5.868 | 0.00E+00 | 0.00E+00 | 8.83 |
| ZBTB11 | -0.652 | 8.491 | -5.867 | 0.00E+00 | 0.00E+00 | 8.823 |
| CCNG1 | -0.722 | 9.319 | -5.863 | 0.00E+00 | 0.00E+00 | 8.803 |
| PLCD1 | 0.595 | 8.164 | 5.858 | 0.00E+00 | 0.00E+00 | 8.786 |
| SP3 | -0.635 | 8.835 | -5.852 | 0.00E+00 | 0.00E+00 | 8.755 |
| BAG5 | -0.391 | 8.283 | -5.849 | 0.00E+00 | 0.00E+00 | 8.739 |
| APAF1 | -0.597 | 7.525 | -5.841 | 0.00E+00 | 0.00E+00 | 8.7 |
| DLD | -0.604 | 9.041 | -5.841 | 0.00E+00 | 0.00E+00 | 8.699 |
| IDI1 | -0.636 | 9.125 | -5.836 | 0.00E+00 | 0.00E+00 | 8.677 |
| RAP2C | -0.666 | 8.695 | -5.818 | 0.00E+00 | 0.00E+00 | 8.593 |
| ERCC1 | 0.368 | 9.269 | 5.812 | 0.00E+00 | 0.00E+00 | 8.57 |
| SNX14 | -0.654 | 9.244 | -5.807 | 0.00E+00 | 0.00E+00 | 8.543 |
| KLHL2 | -0.645 | 8.324 | -5.805 | 0.00E+00 | 0.00E+00 | 8.533 |
| SMPDL3A | -0.713 | 9.118 | -5.798 | 0.00E+00 | 0.00E+00 | 8.497 |
| C6orf62 | -0.523 | 10.266 | -5.792 | 0.00E+00 | 0.00E+00 | 8.471 |
| PCMT1 | -0.466 | 10.362 | -5.77 | 0.00E+00 | 0.00E+00 | 8.37 |
| COMMD2 | -0.514 | 8.354 | -5.758 | 0.00E+00 | 0.00E+00 | 8.313 |
| ARL6IP6 | -0.542 | 9.544 | -5.746 | 0.00E+00 | 0.00E+00 | 8.256 |
| SLC35B3 | -0.555 | 8.624 | -5.733 | 0.00E+00 | 0.00E+00 | 8.195 |
| COPS2 | -0.633 | 8.91 | -5.729 | 0.00E+00 | 0.00E+00 | 8.175 |
| MRPL2 | 0.68 | 7.679 | 5.724 | 0.00E+00 | 0.00E+00 | 8.158 |
| UBE4A | -0.521 | 9.969 | -5.724 | 0.00E+00 | 0.00E+00 | 8.154 |
| TAF10 | 0.476 | 10.21 | 5.717 | 0.00E+00 | 0.00E+00 | 8.124 |
| SKIL | -0.591 | 7.122 | -5.712 | 0.00E+00 | 0.00E+00 | 8.094 |
| MRPS34 | 0.408 | 9.789 | 5.71 | 0.00E+00 | 0.00E+00 | 8.093 |
| SLC35A3 | -0.538 | 7.958 | -5.711 | 0.00E+00 | 0.00E+00 | 8.092 |
| DR1 | -0.514 | 9.044 | -5.699 | 0.00E+00 | 0.00E+00 | 8.036 |
| GCLM | -0.624 | 9.544 | -5.699 | 0.00E+00 | 0.00E+00 | 8.034 |
| PTPN12 | -0.636 | 10.72 | -5.688 | 0.00E+00 | 0.00E+00 | 7.984 |
| NDUFB7 | 0.516 | 11.136 | 5.675 | 0.00E+00 | 0.00E+00 | 7.931 |
| PDE8A | -0.49 | 7.63 | -5.667 | 0.00E+00 | 0.00E+00 | 7.889 |
| RDH10 | -0.634 | 7.801 | -5.635 | 0.00E+00 | 0.00E+00 | 7.741 |
| C9orf16 | 0.464 | 7.98 | 5.623 | 0.00E+00 | 0.00E+00 | 7.689 |
| XPO1 | -0.58 | 10.16 | -5.622 | 0.00E+00 | 0.00E+00 | 7.681 |
| STRN3 | -0.57 | 8.394 | -5.621 | 0.00E+00 | 0.00E+00 | 7.675 |
| CNOT7 | -0.371 | 9.318 | -5.62 | 0.00E+00 | 0.00E+00 | 7.67 |
| ZNF529 | -0.515 | 7.476 | -5.61 | 0.00E+00 | 0.00E+00 | 7.624 |
| SNX13 | -0.625 | 8.401 | -5.609 | 0.00E+00 | 0.00E+00 | 7.621 |
| ACD | 0.513 | 8.55 | 5.606 | 0.00E+00 | 0.00E+00 | 7.612 |
| SLC36A4 | -0.497 | 7.86 | -5.584 | 0.00E+00 | 0.00E+00 | 7.506 |
| PLSCR1 | -0.9 | 9.462 | -5.58 | 0.00E+00 | 0.00E+00 | 7.486 |
| SDCBP | -0.615 | 11.106 | -5.569 | 0.00E+00 | 0.00E+00 | 7.439 |
| KIAA0907 | -0.467 | 9.111 | -5.568 | 0.00E+00 | 0.00E+00 | 7.434 |
| NDUFB11 | 0.403 | 10.876 | 5.565 | 0.00E+00 | 0.00E+00 | 7.422 |
| FDX1 | -0.642 | 9.035 | -5.563 | 0.00E+00 | 0.00E+00 | 7.409 |
| PSMD12 | -0.471 | 8.861 | -5.563 | 0.00E+00 | 0.00E+00 | 7.408 |
| STARD3NL | -0.596 | 9.624 | -5.555 | 0.00E+00 | 0.00E+00 | 7.373 |
| RAB28 | -0.555 | 7.623 | -5.552 | 0.00E+00 | 0.00E+00 | 7.359 |
| PRR3 | 0.434 | 7.233 | 5.545 | 0.00E+00 | 0.00E+00 | 7.333 |
| TRIP12 | -0.707 | 9.512 | -5.544 | 0.00E+00 | 0.00E+00 | 7.322 |
| AGL | -0.617 | 8.446 | -5.541 | 0.00E+00 | 0.00E+00 | 7.31 |
| EIF1AX | -0.648 | 9.96 | -5.54 | 0.00E+00 | 0.00E+00 | 7.304 |
| SLMAP | -0.64 | 8.101 | -5.531 | 0.00E+00 | 0.00E+00 | 7.265 |
| ATP6V1A | -0.476 | 10.564 | -5.531 | 0.00E+00 | 0.00E+00 | 7.264 |
| DCTN4 | -0.611 | 9.653 | -5.531 | 0.00E+00 | 0.00E+00 | 7.263 |
| KRT15 | 0.667 | 7.278 | 5.528 | 0.00E+00 | 0.00E+00 | 7.256 |
| ETF1 | -0.459 | 10.208 | -5.529 | 0.00E+00 | 0.00E+00 | 7.255 |
| ACTR6 | -0.587 | 9.079 | -5.516 | 0.00E+00 | 0.00E+00 | 7.193 |
| SOCS5 | -0.579 | 7.589 | -5.515 | 0.00E+00 | 0.00E+00 | 7.193 |
| MNDA | -0.739 | 8.451 | -5.507 | 0.00E+00 | 0.00E+00 | 7.153 |
| YRDC | -0.725 | 9.805 | -5.507 | 0.00E+00 | 0.00E+00 | 7.153 |
| LLGL1 | 0.594 | 6.987 | 5.498 | 0.00E+00 | 0.00E+00 | 7.116 |
| RPGR | -0.591 | 7.25 | -5.495 | 0.00E+00 | 0.00E+00 | 7.1 |
| GNA13 | -0.626 | 8.461 | -5.493 | 0.00E+00 | 0.00E+00 | 7.089 |
| AHR | -0.724 | 10.701 | -5.481 | 0.00E+00 | 0.00E+00 | 7.034 |
| GPD2 | -0.463 | 7.022 | -5.477 | 0.00E+00 | 0.00E+00 | 7.017 |
| C1GALT1 | -0.648 | 8.582 | -5.475 | 0.00E+00 | 0.00E+00 | 7.007 |
| ZNF75A | -0.602 | 7.248 | -5.472 | 0.00E+00 | 0.00E+00 | 6.998 |
| SIRT6 | 0.419 | 7.969 | 5.468 | 0.00E+00 | 0.00E+00 | 6.981 |
| CSNK1A1 | -0.383 | 9.483 | -5.468 | 0.00E+00 | 0.00E+00 | 6.976 |
| PSIP1 | -0.566 | 8.123 | -5.467 | 0.00E+00 | 0.00E+00 | 6.971 |
| PRDX3 | -0.445 | 10.456 | -5.467 | 0.00E+00 | 0.00E+00 | 6.971 |
| UHRF2 | -0.673 | 7.572 | -5.458 | 0.00E+00 | 0.00E+00 | 6.934 |
| RABGGTB | -0.619 | 9.142 | -5.452 | 0.00E+00 | 0.00E+00 | 6.903 |
| RAB14 | -0.356 | 7.98 | -5.443 | 0.00E+00 | 0.00E+00 | 6.866 |
| TOPBP1 | -0.483 | 8.801 | -5.438 | 0.00E+00 | 0.00E+00 | 6.84 |
| PNRC2 | -0.454 | 9.641 | -5.435 | 0.00E+00 | 0.00E+00 | 6.829 |
| MRPS26 | 0.399 | 10.346 | 5.421 | 0.00E+00 | 0.00E+00 | 6.769 |
| MATR3 | -0.556 | 9.819 | -5.421 | 0.00E+00 | 0.00E+00 | 6.765 |
| EVI5 | -0.736 | 8.306 | -5.417 | 0.00E+00 | 0.00E+00 | 6.75 |
| MAP3K8 | -0.631 | 8.776 | -5.417 | 0.00E+00 | 0.00E+00 | 6.749 |
| CAST | -0.413 | 10.063 | -5.414 | 0.00E+00 | 0.00E+00 | 6.735 |
| CHML | -0.64 | 7.26 | -5.409 | 0.00E+00 | 0.00E+00 | 6.71 |
| ASAH1 | -0.596 | 9.396 | -5.406 | 0.00E+00 | 0.00E+00 | 6.699 |
| ETFA | -0.398 | 10.437 | -5.404 | 0.00E+00 | 0.00E+00 | 6.689 |
| TARBP2 | 0.411 | 9.014 | 5.4 | 0.00E+00 | 0.00E+00 | 6.677 |
| PPL | 0.822 | 7.633 | 5.4 | 0.00E+00 | 0.00E+00 | 6.676 |
| SPCS2 | -0.366 | 10.839 | -5.4 | 0.00E+00 | 0.00E+00 | 6.673 |
| CGGBP1 | -0.462 | 9.433 | -5.394 | 0.00E+00 | 0.00E+00 | 6.646 |
| WAC | -0.401 | 10.613 | -5.391 | 0.00E+00 | 0.00E+00 | 6.629 |
| GDI2 | -0.535 | 10.667 | -5.39 | 0.00E+00 | 0.00E+00 | 6.628 |
| RAB33B | -0.692 | 7.215 | -5.389 | 0.00E+00 | 0.00E+00 | 6.621 |
| CRELD1 | 0.398 | 8.262 | 5.385 | 0.00E+00 | 0.00E+00 | 6.608 |
| USP14 | -0.722 | 8.028 | -5.367 | 0.00E+00 | 0.00E+00 | 6.524 |
| CACNA1H | 0.654 | 6.853 | 5.362 | 0.00E+00 | 0.00E+00 | 6.507 |
| PSENEN | 0.43 | 9.127 | 5.361 | 0.00E+00 | 0.00E+00 | 6.502 |
| SLC40A1 | -0.915 | 9.851 | -5.355 | 0.00E+00 | 0.00E+00 | 6.472 |
| ETHE1 | 0.465 | 8.855 | 5.351 | 0.00E+00 | 0.00E+00 | 6.456 |
| CDH15 | 0.773 | 6.74 | 5.341 | 0.00E+00 | 0.00E+00 | 6.412 |
| SSR4 | 0.725 | 12.351 | 5.337 | 0.00E+00 | 0.00E+00 | 6.396 |
| ECHDC1 | -0.458 | 8.247 | -5.333 | 0.00E+00 | 0.00E+00 | 6.371 |
| MAP4K3 | -0.579 | 8.134 | -5.331 | 0.00E+00 | 0.00E+00 | 6.365 |
| USP1 | -0.533 | 8.865 | -5.328 | 0.00E+00 | 0.00E+00 | 6.351 |
| PLEKHF2 | -0.509 | 9.264 | -5.326 | 0.00E+00 | 0.00E+00 | 6.343 |
| SNCG | 0.579 | 6.75 | 5.324 | 0.00E+00 | 0.00E+00 | 6.336 |
| GNAI1 | -0.688 | 8.337 | -5.324 | 0.00E+00 | 0.00E+00 | 6.333 |
| ADSS | -0.619 | 8.744 | -5.321 | 0.00E+00 | 0.00E+00 | 6.321 |
| PSMD14 | -0.513 | 10.504 | -5.301 | 0.00E+00 | 0.00E+00 | 6.232 |
| SRP9 | -0.518 | 9.985 | -5.3 | 0.00E+00 | 0.00E+00 | 6.226 |
| C6orf203 | -0.474 | 8.044 | -5.293 | 0.00E+00 | 0.00E+00 | 6.193 |
| MAPK6 | -0.646 | 8.773 | -5.291 | 0.00E+00 | 0.00E+00 | 6.185 |
| IL18 | -0.828 | 8.63 | -5.29 | 0.00E+00 | 0.00E+00 | 6.184 |
| C6orf106 | 0.459 | 8.883 | 5.285 | 0.00E+00 | 0.00E+00 | 6.166 |
| VDAC1 | -0.44 | 10.867 | -5.282 | 0.00E+00 | 0.00E+00 | 6.145 |
| OCRL | 0.478 | 8.936 | 5.279 | 0.00E+00 | 0.00E+00 | 6.14 |
| JRKL | -0.417 | 7.175 | -5.27 | 0.00E+00 | 0.00E+00 | 6.095 |
| MPG | 0.487 | 8.64 | 5.261 | 0.00E+00 | 0.00E+00 | 6.057 |
| TXN2 | 0.358 | 9.329 | 5.257 | 0.00E+00 | 0.00E+00 | 6.039 |
| PAN3 | -0.471 | 8.337 | -5.254 | 0.00E+00 | 0.00E+00 | 6.025 |
| NCOA2 | -0.592 | 6.681 | -5.242 | 0.00E+00 | 0.00E+00 | 5.968 |
| IGFBP5 | 0.732 | 8.797 | 5.24 | 0.00E+00 | 0.00E+00 | 5.968 |
| S100A16 | 0.573 | 9.652 | 5.237 | 0.00E+00 | 0.00E+00 | 5.952 |
| GLIS1 | 0.718 | 6.658 | 5.233 | 0.00E+00 | 0.00E+00 | 5.935 |
| KIAA0196 | -0.471 | 9.674 | -5.229 | 0.00E+00 | 0.00E+00 | 5.915 |
| TCF4 | 0.529 | 9.811 | 5.227 | 0.00E+00 | 0.00E+00 | 5.908 |
| OCIAD1 | -0.393 | 10.354 | -5.224 | 0.00E+00 | 0.00E+00 | 5.891 |
| CTBP2 | -0.312 | 9.132 | -5.218 | 0.00E+00 | 0.00E+00 | 5.867 |
| RCHY1 | -0.465 | 7.882 | -5.218 | 0.00E+00 | 0.00E+00 | 5.866 |
| SCFD1 | -0.508 | 9.183 | -5.212 | 0.00E+00 | 0.00E+00 | 5.84 |
| DNM1L | -0.416 | 8.273 | -5.203 | 0.00E+00 | 0.00E+00 | 5.798 |
| TFG | -0.364 | 10.723 | -5.201 | 0.00E+00 | 0.00E+00 | 5.789 |
| TUBGCP3 | -0.454 | 7.418 | -5.197 | 0.00E+00 | 0.00E+00 | 5.772 |
| PGM2 | -0.544 | 9.694 | -5.194 | 0.00E+00 | 0.00E+00 | 5.759 |
| MBTD1 | -0.56 | 6.95 | -5.181 | 0.00E+00 | 0.00E+00 | 5.704 |
| LCMT1 | 0.389 | 9.857 | 5.172 | 0.00E+00 | 0.00E+00 | 5.67 |
| IMPA1 | -0.572 | 7.845 | -5.172 | 0.00E+00 | 0.00E+00 | 5.665 |
| ZFR | -0.437 | 9.657 | -5.172 | 0.00E+00 | 0.00E+00 | 5.664 |
| ARL6IP5 | -0.471 | 11.581 | -5.171 | 0.00E+00 | 0.00E+00 | 5.66 |
| IL1RAP | -0.649 | 8.347 | -5.16 | 0.00E+00 | 0.00E+00 | 5.614 |
| MRPL23 | 0.454 | 10.1 | 5.158 | 0.00E+00 | 0.00E+00 | 5.608 |
| FZD6 | -0.88 | 7.34 | -5.157 | 0.00E+00 | 0.00E+00 | 5.601 |
| PTGER2 | -0.591 | 7.691 | -5.144 | 0.00E+00 | 0.00E+00 | 5.544 |
| ANXA3 | -0.77 | 10.058 | -5.14 | 0.00E+00 | 0.00E+00 | 5.527 |
| FAM49B | -0.501 | 8.902 | -5.14 | 0.00E+00 | 0.00E+00 | 5.526 |
| YES1 | -0.678 | 9.193 | -5.137 | 0.00E+00 | 0.00E+00 | 5.511 |
| A1BG | 0.656 | 7.024 | 5.128 | 0.00E+00 | 0.00E+00 | 5.477 |
| ATP6V1C1 | -0.558 | 8.76 | -5.113 | 0.00E+00 | 0.00E+00 | 5.41 |
| TMED5 | -0.475 | 9.419 | -5.111 | 0.00E+00 | 0.00E+00 | 5.402 |
| TRIM56 | -0.458 | 9.347 | -5.098 | 0.00E+00 | 0.00E+00 | 5.344 |
| SAMD1 | 0.532 | 7.248 | 5.095 | 0.00E+00 | 0.00E+00 | 5.334 |
| PUM1 | -0.341 | 11.161 | -5.094 | 0.00E+00 | 0.00E+00 | 5.328 |
| NKIRAS2 | 0.42 | 8.817 | 5.092 | 0.00E+00 | 0.00E+00 | 5.325 |
| TPD52 | -0.383 | 7.403 | -5.093 | 0.00E+00 | 0.00E+00 | 5.321 |
| RAB10 | -0.361 | 10.881 | -5.088 | 0.00E+00 | 0.00E+00 | 5.299 |
| SNX3 | -0.429 | 10.882 | -5.083 | 0.00E+00 | 0.00E+00 | 5.279 |
| KIAA0232 | -0.569 | 8.391 | -5.083 | 0.00E+00 | 0.00E+00 | 5.279 |
| MBD4 | -0.494 | 9.394 | -5.08 | 0.00E+00 | 0.00E+00 | 5.266 |
| WDR24 | 0.452 | 7.945 | 5.076 | 0.00E+00 | 0.00E+00 | 5.254 |
| UBE3C | -0.386 | 8.222 | -5.076 | 0.00E+00 | 0.00E+00 | 5.249 |
| CDC42SE2 | -0.524 | 7.861 | -5.074 | 0.00E+00 | 0.00E+00 | 5.24 |
| SLC38A6 | -0.461 | 7.789 | -5.072 | 0.00E+00 | 0.00E+00 | 5.231 |
| RERE | 0.48 | 7.848 | 5.067 | 0.00E+00 | 0.00E+00 | 5.217 |
| DLG5 | -0.715 | 8.889 | -5.065 | 0.00E+00 | 0.00E+00 | 5.202 |
| NAP1L1 | -0.565 | 9.3 | -5.063 | 0.00E+00 | 0.00E+00 | 5.196 |
| SERPINF1 | 0.526 | 10.451 | 5.057 | 0.00E+00 | 0.00E+00 | 5.173 |
| RAP2A | -0.698 | 8.297 | -5.057 | 0.00E+00 | 0.00E+00 | 5.171 |
| NUP43 | -0.428 | 8.293 | -5.052 | 0.00E+00 | 0.00E+00 | 5.146 |
| HERC4 | -0.398 | 8.153 | -5.044 | 0.00E+00 | 0.00E+00 | 5.111 |
| CABP4 | 0.39 | 6.558 | 5.042 | 0.00E+00 | 0.00E+00 | 5.107 |
| POLRMT | 0.444 | 8.882 | 5.038 | 0.00E+00 | 0.00E+00 | 5.09 |
| LDHD | 0.693 | 6.811 | 5.037 | 0.00E+00 | 0.00E+00 | 5.088 |
| POLR2I | 0.421 | 9.989 | 5.035 | 0.00E+00 | 0.00E+00 | 5.079 |
| SH3GLB1 | -0.377 | 10.384 | -5.036 | 0.00E+00 | 0.00E+00 | 5.079 |
| BNIP2 | -0.517 | 9.822 | -5.031 | 0.00E+00 | 0.00E+00 | 5.056 |
| GPR3 | 0.515 | 6.013 | 5.028 | 0.00E+00 | 0.00E+00 | 5.05 |
| NDUFA11 | 0.426 | 11.627 | 5.023 | 0.00E+00 | 0.00E+00 | 5.027 |
| PHLDB2 | -0.593 | 8.65 | -5.023 | 0.00E+00 | 0.00E+00 | 5.025 |
| CPD | -0.508 | 9.488 | -5.017 | 0.00E+00 | 0.00E+00 | 4.996 |
| SNAPC2 | 0.406 | 9.821 | 5.012 | 0.00E+00 | 0.00E+00 | 4.981 |
| TRUB1 | -0.575 | 7.264 | -5.008 | 0.00E+00 | 0.00E+00 | 4.961 |
| NTHL1 | 0.478 | 8.089 | 5.006 | 0.00E+00 | 0.00E+00 | 4.956 |
| TRIT1 | -0.584 | 8.961 | -5.004 | 0.00E+00 | 0.00E+00 | 4.944 |
| IFI16 | -0.616 | 10.131 | -5.001 | 0.00E+00 | 0.00E+00 | 4.93 |
| EIF4G2 | -0.565 | 12.45 | -5 | 0.00E+00 | 0.00E+00 | 4.927 |
| OSBPL2 | -0.381 | 9.062 | -4.996 | 0.00E+00 | 0.00E+00 | 4.91 |
| CPEB2 | -0.634 | 6.133 | -4.989 | 0.00E+00 | 0.00E+00 | 4.877 |
| LRP1 | 0.604 | 7.74 | 4.987 | 0.00E+00 | 0.00E+00 | 4.876 |
| PAK6 | 0.588 | 7.43 | 4.987 | 0.00E+00 | 0.00E+00 | 4.875 |
| PSMC6 | -0.482 | 10.594 | -4.984 | 0.00E+00 | 0.00E+00 | 4.859 |
| PTEN | -0.468 | 10.014 | -4.976 | 0.00E+00 | 0.00E+00 | 4.826 |
| NCF2 | -0.583 | 8.708 | -4.972 | 0.00E+00 | 0.00E+00 | 4.806 |
| PLA2G2A | 0.839 | 8.86 | 4.97 | 0.00E+00 | 0.00E+00 | 4.805 |
| MYL6 | 0.364 | 14 | 4.969 | 0.00E+00 | 0.00E+00 | 4.798 |
| SPRY2 | -0.503 | 8.312 | -4.97 | 0.00E+00 | 0.00E+00 | 4.797 |
| COX6A1 | 0.346 | 11.836 | 4.968 | 0.00E+00 | 0.00E+00 | 4.792 |
| FOXJ3 | -0.522 | 10.633 | -4.956 | 0.00E+00 | 0.00E+00 | 4.74 |
| MBD2 | -0.366 | 8.543 | -4.954 | 0.00E+00 | 0.00E+00 | 4.732 |
| TRIM52 | -0.431 | 7.776 | -4.949 | 0.00E+00 | 0.00E+00 | 4.711 |
| CPT1C | 0.684 | 7.077 | 4.946 | 0.00E+00 | 0.00E+00 | 4.703 |
| OXER1 | 0.621 | 7.433 | 4.945 | 0.00E+00 | 0.00E+00 | 4.698 |
| GALNT7 | -0.531 | 7.737 | -4.944 | 0.00E+00 | 0.00E+00 | 4.689 |
| PNN | -0.514 | 9.617 | -4.944 | 0.00E+00 | 0.00E+00 | 4.688 |
| CCNL1 | -0.529 | 9.239 | -4.944 | 0.00E+00 | 0.00E+00 | 4.687 |
| CHD1 | -0.685 | 8.7 | -4.941 | 0.00E+00 | 0.00E+00 | 4.674 |
| GOLGA5 | -0.45 | 9.787 | -4.938 | 0.00E+00 | 0.00E+00 | 4.665 |
| PON2 | -0.499 | 9.986 | -4.936 | 0.00E+00 | 0.00E+00 | 4.655 |
| APEX2 | 0.475 | 8.815 | 4.935 | 0.00E+00 | 0.00E+00 | 4.654 |
| RILP | 0.529 | 7.899 | 4.923 | 0.00E+00 | 0.00E+00 | 4.605 |
| UBE2A | -0.367 | 10.526 | -4.923 | 0.00E+00 | 0.00E+00 | 4.603 |
| HDHD2 | -0.374 | 9.61 | -4.923 | 0.00E+00 | 0.00E+00 | 4.603 |
| DENR | -0.399 | 9.304 | -4.92 | 0.00E+00 | 0.00E+00 | 4.588 |
| COMMD10 | -0.495 | 9.09 | -4.916 | 0.00E+00 | 0.00E+00 | 4.573 |
| SENP7 | -0.551 | 7.313 | -4.914 | 0.00E+00 | 0.00E+00 | 4.563 |
| CHCHD6 | 0.472 | 7.649 | 4.907 | 0.00E+00 | 0.00E+00 | 4.54 |
| EYA3 | 0.352 | 6.248 | 4.905 | 0.00E+00 | 0.00E+00 | 4.531 |
| HSF2 | -0.516 | 8.144 | -4.904 | 0.00E+00 | 0.00E+00 | 4.522 |
| CHPF | 0.743 | 9.262 | 4.903 | 0.00E+00 | 0.00E+00 | 4.521 |
| E4F1 | 0.378 | 9.191 | 4.902 | 0.00E+00 | 0.00E+00 | 4.518 |
| POLR3F | -0.424 | 7.608 | -4.899 | 0.00E+00 | 0.00E+00 | 4.501 |
| PSTPIP2 | -0.575 | 7.464 | -4.899 | 0.00E+00 | 0.00E+00 | 4.5 |
| OAT | -0.476 | 9.969 | -4.898 | 0.00E+00 | 0.00E+00 | 4.494 |
| PPP2CA | -0.472 | 11.275 | -4.897 | 0.00E+00 | 0.00E+00 | 4.493 |
| AMD1 | -0.657 | 10.752 | -4.896 | 0.00E+00 | 0.00E+00 | 4.489 |
| CLK4 | -0.53 | 8 | -4.896 | 0.00E+00 | 0.00E+00 | 4.486 |
| HSPH1 | -0.573 | 9.583 | -4.895 | 0.00E+00 | 0.00E+00 | 4.485 |
| SMARCC2 | 0.474 | 9.641 | 4.885 | 0.00E+00 | 0.00E+00 | 4.446 |
| KIAA1468 | -0.431 | 8.203 | -4.884 | 0.00E+00 | 0.00E+00 | 4.436 |
| GTF3C3 | -0.332 | 8.463 | -4.883 | 0.00E+00 | 0.00E+00 | 4.432 |
| GSTM2 | 0.557 | 6.783 | 4.879 | 0.00E+00 | 0.00E+00 | 4.422 |
| BTNL2 | 0.601 | 6.589 | 4.877 | 0.00E+00 | 0.00E+00 | 4.412 |
| ZNF555 | -0.562 | 7.07 | -4.872 | 0.00E+00 | 0.00E+00 | 4.389 |
| PERP | -0.711 | 9.728 | -4.872 | 0.00E+00 | 0.00E+00 | 4.388 |
| DNAJC4 | 0.434 | 7.829 | 4.869 | 0.00E+00 | 0.00E+00 | 4.379 |
| PPIH | 0.52 | 8.814 | 4.862 | 0.00E+00 | 0.00E+00 | 4.351 |
| PIGA | -0.482 | 7.932 | -4.863 | 0.00E+00 | 0.00E+00 | 4.35 |
| C8A | 0.463 | 5.114 | 4.861 | 0.00E+00 | 0.00E+00 | 4.348 |
| ZNF71 | 0.442 | 7 | 4.86 | 0.00E+00 | 0.00E+00 | 4.343 |
| STAP2 | 0.794 | 7.265 | 4.858 | 0.00E+00 | 0.00E+00 | 4.333 |
| RAB8B | -0.508 | 8.965 | -4.858 | 0.00E+00 | 0.00E+00 | 4.33 |
| MAPKAP1 | -0.425 | 8.309 | -4.857 | 0.00E+00 | 0.00E+00 | 4.326 |
| PDCD4 | -0.626 | 9.398 | -4.853 | 0.00E+00 | 0.00E+00 | 4.31 |
| SUPT5H | 0.44 | 9.22 | 4.845 | 0.00E+00 | 0.00E+00 | 4.279 |
| FGFR1OP2 | -0.427 | 8.101 | -4.843 | 0.00E+00 | 0.00E+00 | 4.268 |
| DMXL1 | -0.554 | 6.513 | -4.841 | 0.00E+00 | 0.00E+00 | 4.259 |
| PTBP2 | -0.524 | 7.971 | -4.838 | 0.00E+00 | 0.00E+00 | 4.245 |
| GAB1 | -0.564 | 6.439 | -4.835 | 0.00E+00 | 0.00E+00 | 4.232 |
| PTP4A2 | -0.413 | 9.221 | -4.832 | 0.00E+00 | 0.00E+00 | 4.222 |
| RTKN | 0.471 | 6.992 | 4.828 | 0.00E+00 | 0.00E+00 | 4.209 |
| MRPL37 | 0.417 | 10.225 | 4.821 | 0.00E+00 | 0.00E+00 | 4.18 |
| SSSCA1 | 0.39 | 9.313 | 4.817 | 0.00E+00 | 0.00E+00 | 4.163 |
| ASF1A | -0.538 | 7.307 | -4.813 | 0.00E+00 | 0.00E+00 | 4.144 |
| SAP30L | -0.447 | 9.083 | -4.808 | 0.00E+00 | 0.00E+00 | 4.123 |
| LACTB2 | -0.441 | 8.057 | -4.807 | 0.00E+00 | 0.00E+00 | 4.118 |
| BTK | -0.53 | 6.913 | -4.803 | 0.00E+00 | 0.00E+00 | 4.104 |
| RHOA | -0.419 | 11.91 | -4.802 | 0.00E+00 | 0.00E+00 | 4.097 |
| HIST1H2AJ | 0.457 | 8.659 | 4.796 | 0.00E+00 | 0.00E+00 | 4.076 |
| THAP1 | -0.354 | 8.322 | -4.792 | 0.00E+00 | 0.00E+00 | 4.055 |
| MTRF1L | -0.302 | 7.589 | -4.789 | 0.00E+00 | 0.00E+00 | 4.042 |
| AZI2 | -0.389 | 8.428 | -4.781 | 0.00E+00 | 0.00E+00 | 4.013 |
| RANBP2 | -0.398 | 8.2 | -4.78 | 0.00E+00 | 0.00E+00 | 4.008 |
| ZNF639 | -0.318 | 7.511 | -4.779 | 0.00E+00 | 0.00E+00 | 4.003 |
| PDGFC | -0.703 | 7.686 | -4.779 | 0.00E+00 | 0.00E+00 | 4.002 |
| ARL2 | 0.387 | 10.482 | 4.773 | 0.00E+00 | 0.00E+00 | 3.983 |
| LMOD1 | 0.616 | 7.11 | 4.766 | 0.00E+00 | 0.00E+00 | 3.956 |
| C1orf43 | -0.367 | 10.08 | -4.767 | 0.00E+00 | 0.00E+00 | 3.952 |
| TRIM26 | 0.397 | 8.809 | 4.765 | 0.00E+00 | 0.00E+00 | 3.952 |
| ABCD3 | -0.519 | 7.502 | -4.766 | 0.00E+00 | 0.00E+00 | 3.95 |
| LYPLA1 | -0.4 | 10.029 | -4.766 | 0.00E+00 | 0.00E+00 | 3.95 |
| PDCD10 | -0.521 | 9.669 | -4.763 | 0.00E+00 | 0.00E+00 | 3.939 |
| PKN2 | -0.427 | 8.484 | -4.76 | 0.00E+00 | 0.00E+00 | 3.924 |
| NDUFA5 | -0.405 | 8.084 | -4.758 | 0.00E+00 | 0.00E+00 | 3.919 |
| ZBED4 | -0.403 | 7.455 | -4.75 | 0.00E+00 | 0.00E+00 | 3.886 |
| CD58 | -0.405 | 8.498 | -4.745 | 0.00E+00 | 0.00E+00 | 3.865 |
| CHUK | -0.523 | 8.568 | -4.735 | 0.00E+00 | 0.00E+00 | 3.824 |
| VPS54 | -0.37 | 7.732 | -4.735 | 0.00E+00 | 0.00E+00 | 3.821 |
| RNF13 | -0.468 | 9.724 | -4.734 | 0.00E+00 | 0.00E+00 | 3.817 |
| NFIL3 | -0.509 | 10.604 | -4.726 | 0.00E+00 | 0.00E+00 | 3.786 |
| TAOK2 | 0.45 | 8.373 | 4.72 | 0.00E+00 | 0.00E+00 | 3.768 |
| TLR1 | -0.537 | 7.544 | -4.717 | 0.00E+00 | 0.00E+00 | 3.75 |
| SLC27A1 | 0.352 | 8.939 | 4.716 | 0.00E+00 | 0.00E+00 | 3.75 |
| ZNF644 | -0.596 | 7.707 | -4.716 | 0.00E+00 | 0.00E+00 | 3.747 |
| GDPD1 | -0.642 | 6.486 | -4.715 | 0.00E+00 | 0.00E+00 | 3.744 |
| CHFR | -0.417 | 8.445 | -4.713 | 0.00E+00 | 0.00E+00 | 3.735 |
| MST1R | 0.555 | 5.934 | 4.712 | 0.00E+00 | 0.00E+00 | 3.734 |
| CREBL2 | -0.363 | 8.148 | -4.709 | 0.00E+00 | 0.00E+00 | 3.719 |
| KLHL17 | 0.48 | 7.5 | 4.707 | 0.00E+00 | 0.00E+00 | 3.713 |
| IL27RA | 0.532 | 8.023 | 4.7 | 0.00E+00 | 0.00E+00 | 3.684 |
| CDH3 | 0.647 | 7.305 | 4.7 | 0.00E+00 | 0.00E+00 | 3.684 |
| UCKL1 | 0.537 | 9.281 | 4.697 | 0.00E+00 | 0.00E+00 | 3.671 |
| LIFR | -0.735 | 7.755 | -4.69 | 0.00E+00 | 0.00E+00 | 3.64 |
| DUSP12 | -0.355 | 10.074 | -4.687 | 0.00E+00 | 0.00E+00 | 3.627 |
| BARX2 | 0.5 | 5.56 | 4.683 | 0.00E+00 | 0.00E+00 | 3.616 |
| TXNL1 | -0.572 | 9.142 | -4.683 | 0.00E+00 | 0.00E+00 | 3.613 |
| DPP7 | 0.441 | 9.068 | 4.682 | 0.00E+00 | 0.00E+00 | 3.611 |
| BCAS4 | 0.447 | 7.556 | 4.682 | 0.00E+00 | 0.00E+00 | 3.611 |
| CREM | -0.342 | 7.445 | -4.682 | 0.00E+00 | 0.00E+00 | 3.607 |
| ADM | -0.641 | 13.619 | -4.68 | 0.00E+00 | 0.00E+00 | 3.599 |
| TFPT | 0.359 | 9.335 | 4.676 | 0.00E+00 | 0.00E+00 | 3.587 |
| PHF14 | -0.406 | 7.383 | -4.673 | 0.00E+00 | 0.00E+00 | 3.571 |
| PHLDA3 | 0.514 | 8.153 | 4.669 | 0.00E+00 | 0.00E+00 | 3.558 |
| TSNAX | -0.401 | 8.887 | -4.669 | 0.00E+00 | 0.00E+00 | 3.555 |
| TNFRSF4 | 0.572 | 7.016 | 4.664 | 0.00E+00 | 0.00E+00 | 3.539 |
| EHHADH | -0.637 | 5.635 | -4.664 | 0.00E+00 | 0.00E+00 | 3.536 |
| PRKRIR | -0.386 | 9.791 | -4.658 | 0.00E+00 | 0.00E+00 | 3.513 |
| ACTR1A | 0.401 | 10.232 | 4.654 | 0.00E+00 | 0.00E+00 | 3.497 |
| NUP160 | -0.427 | 8.942 | -4.653 | 0.00E+00 | 0.00E+00 | 3.493 |
| NDUFB5 | -0.307 | 10.41 | -4.652 | 0.00E+00 | 0.00E+00 | 3.488 |
| SLC25A5 | -0.319 | 12.856 | -4.65 | 0.00E+00 | 0.00E+00 | 3.479 |
| SMARCA5 | -0.373 | 8.713 | -4.647 | 0.00E+00 | 0.00E+00 | 3.468 |
| RBBP4 | -0.409 | 8.292 | -4.647 | 0.00E+00 | 0.00E+00 | 3.465 |
| NBEA | -0.583 | 6.022 | -4.643 | 0.00E+00 | 0.00E+00 | 3.451 |
| RAB33A | 0.533 | 6.863 | 4.642 | 0.00E+00 | 0.00E+00 | 3.45 |
| SLC41A3 | 0.34 | 8.785 | 4.642 | 0.00E+00 | 0.00E+00 | 3.449 |
| SH3YL1 | -0.638 | 7.557 | -4.642 | 0.00E+00 | 0.00E+00 | 3.445 |
| ANKRD12 | -0.485 | 8.427 | -4.641 | 0.00E+00 | 0.00E+00 | 3.443 |
| ZNF354B | -0.559 | 7.196 | -4.64 | 0.00E+00 | 0.00E+00 | 3.439 |
| MRPS27 | 0.35 | 8.515 | 4.637 | 0.00E+00 | 0.00E+00 | 3.43 |
| KBTBD7 | -0.476 | 7.947 | -4.638 | 0.00E+00 | 0.00E+00 | 3.429 |
| TCEB1 | -0.309 | 10.349 | -4.635 | 0.00E+00 | 0.00E+00 | 3.42 |
| PDRG1 | 0.326 | 9.256 | 4.631 | 0.00E+00 | 0.00E+00 | 3.406 |
| SLC24A1 | -0.431 | 6.772 | -4.631 | 0.00E+00 | 0.00E+00 | 3.403 |
| NXPH4 | 0.653 | 6.851 | 4.63 | 0.00E+00 | 0.00E+00 | 3.402 |
| FAM3C | -0.457 | 9.349 | -4.631 | 0.00E+00 | 0.00E+00 | 3.402 |
| ADD3 | -0.708 | 8.721 | -4.631 | 0.00E+00 | 0.00E+00 | 3.401 |
| SLC19A2 | -0.615 | 8.915 | -4.629 | 0.00E+00 | 0.00E+00 | 3.393 |
| MIF | 0.381 | 13.531 | 4.627 | 0.00E+00 | 0.00E+00 | 3.391 |
| RP2 | -0.418 | 9.204 | -4.625 | 0.00E+00 | 0.00E+00 | 3.379 |
| USP49 | -0.761 | 7.59 | -4.624 | 0.00E+00 | 0.00E+00 | 3.375 |
| ANP32A | -0.36 | 8.709 | -4.623 | 0.00E+00 | 0.00E+00 | 3.371 |
| MANEA | -0.433 | 6.816 | -4.617 | 0.00E+00 | 0.00E+00 | 3.347 |
| CTBS | -0.391 | 9.219 | -4.612 | 0.00E+00 | 0.00E+00 | 3.326 |
| VBP1 | -0.537 | 9.258 | -4.612 | 0.00E+00 | 0.00E+00 | 3.325 |
| CLDN3 | 0.675 | 7.648 | 4.609 | 0.00E+00 | 0.00E+00 | 3.32 |
| STAG2 | -0.528 | 9.031 | -4.61 | 0.00E+00 | 0.00E+00 | 3.319 |
| GSTK1 | 0.329 | 11.031 | 4.607 | 0.00E+00 | 0.00E+00 | 3.31 |
| GKAP1 | -0.443 | 6.916 | -4.603 | 0.00E+00 | 0.00E+00 | 3.29 |
| MAPRE1 | -0.381 | 10.813 | -4.602 | 0.00E+00 | 0.00E+00 | 3.287 |
| MSH2 | -0.428 | 8.138 | -4.602 | 0.00E+00 | 0.00E+00 | 3.285 |
| YEATS4 | -0.529 | 8.126 | -4.601 | 0.00E+00 | 0.00E+00 | 3.284 |
| ODF2 | 0.341 | 7.71 | 4.599 | 0.00E+00 | 0.00E+00 | 3.279 |
| KPNA3 | -0.436 | 8.783 | -4.595 | 0.00E+00 | 0.00E+00 | 3.258 |
| WDR44 | -0.42 | 7.595 | -4.592 | 0.00E+00 | 0.00E+00 | 3.246 |
| NIPSNAP3B | -0.528 | 6.337 | -4.592 | 0.00E+00 | 0.00E+00 | 3.246 |
| DDX20 | -0.314 | 7.952 | -4.583 | 0.00E+00 | 0.00E+00 | 3.211 |
| WSB1 | -0.537 | 10.522 | -4.582 | 0.00E+00 | 0.00E+00 | 3.207 |
| C9orf72 | -0.572 | 6.974 | -4.58 | 0.00E+00 | 0.00E+00 | 3.199 |
| TSN | -0.495 | 8.029 | -4.576 | 0.00E+00 | 0.00E+00 | 3.184 |
| CAPN7 | -0.387 | 8.064 | -4.57 | 0.00E+00 | 0.00E+00 | 3.16 |
| GRIK5 | 0.495 | 6.315 | 4.568 | 0.00E+00 | 0.00E+00 | 3.157 |
| FGD6 | -0.502 | 7.119 | -4.567 | 0.00E+00 | 0.00E+00 | 3.148 |
| VPS4A | 0.395 | 9.6 | 4.565 | 0.00E+00 | 0.00E+00 | 3.143 |
| ALG5 | -0.481 | 10.33 | -4.566 | 0.00E+00 | 0.00E+00 | 3.142 |
| RSU1 | -0.358 | 8.243 | -4.564 | 0.00E+00 | 0.00E+00 | 3.136 |
| BOK | 0.627 | 8.05 | 4.56 | 0.00E+00 | 0.00E+00 | 3.123 |
| CA11 | 0.549 | 6.566 | 4.557 | 0.00E+00 | 0.00E+00 | 3.111 |
| C1orf50 | 0.329 | 9.23 | 4.553 | 0.00E+00 | 0.00E+00 | 3.096 |
| MRPS18A | 0.38 | 9.256 | 4.547 | 0.00E+00 | 0.00E+00 | 3.071 |
| R3HDML | 0.604 | 5.622 | 4.541 | 0.00E+00 | 0.00E+00 | 3.048 |
| STAT5B | -0.513 | 9.425 | -4.542 | 0.00E+00 | 0.00E+00 | 3.048 |
| WBP4 | -0.597 | 8.473 | -4.541 | 0.00E+00 | 0.00E+00 | 3.045 |
| MYNN | -0.383 | 7.302 | -4.54 | 0.00E+00 | 0.00E+00 | 3.041 |
| NIPA1 | -0.545 | 6.834 | -4.534 | 0.00E+00 | 0.00E+00 | 3.019 |
| SLC43A1 | 0.395 | 7.603 | 4.529 | 0.00E+00 | 0.00E+00 | 3.001 |
| BMP2 | 1.009 | 7.461 | 4.529 | 0.00E+00 | 0.00E+00 | 2.999 |
| GGN | 0.433 | 6.575 | 4.521 | 0.00E+00 | 0.00E+00 | 2.971 |
| ELL2 | -0.511 | 8.776 | -4.522 | 0.00E+00 | 0.00E+00 | 2.971 |
| MYLIP | -0.408 | 11.088 | -4.521 | 0.00E+00 | 0.00E+00 | 2.967 |
| TRNT1 | -0.325 | 7.572 | -4.515 | 0.00E+00 | 0.00E+00 | 2.942 |
| NLGN2 | 0.449 | 7.581 | 4.513 | 0.00E+00 | 0.00E+00 | 2.938 |
| ARID3A | 0.511 | 10.178 | 4.507 | 0.00E+00 | 0.00E+00 | 2.916 |
| XPA | -0.33 | 7.863 | -4.505 | 0.00E+00 | 0.00E+00 | 2.902 |
| PAFAH1B3 | 0.501 | 8.394 | 4.501 | 0.00E+00 | 0.00E+00 | 2.89 |
| RRAS | 0.416 | 11.282 | 4.49 | 0.00E+00 | 0.00E+00 | 2.848 |
| HOMER1 | -0.475 | 7.318 | -4.489 | 0.00E+00 | 0.00E+00 | 2.838 |
| SLC7A13 | 0.517 | 4.914 | 4.483 | 0.00E+00 | 0.00E+00 | 2.821 |
| SLBP | -0.353 | 10.323 | -4.483 | 0.00E+00 | 0.00E+00 | 2.816 |
| MTRF1 | -0.399 | 7.205 | -4.473 | 0.00E+00 | 0.00E+00 | 2.777 |
| RNF20 | -0.512 | 8.448 | -4.468 | 0.00E+00 | 0.00E+00 | 2.759 |
| E2F3 | -0.583 | 8.304 | -4.467 | 0.00E+00 | 0.00E+00 | 2.754 |
| C18orf25 | -0.466 | 7.956 | -4.465 | 0.00E+00 | 0.00E+00 | 2.746 |
| DDX51 | 0.46 | 7.839 | 4.463 | 0.00E+00 | 0.00E+00 | 2.741 |
| OFD1 | -0.426 | 8.602 | -4.463 | 0.00E+00 | 0.00E+00 | 2.74 |
| RNF121 | 0.344 | 7.91 | 4.461 | 0.00E+00 | 0.00E+00 | 2.733 |
| PABPN1 | 0.291 | 10.709 | 4.456 | 0.00E+00 | 0.00E+00 | 2.715 |
| IDH3G | 0.295 | 9.12 | 4.443 | 0.00E+00 | 0.00E+00 | 2.664 |
| DDX24 | 0.412 | 9.201 | 4.442 | 0.00E+00 | 0.00E+00 | 2.662 |
| CD2AP | -0.536 | 7.634 | -4.443 | 0.00E+00 | 0.00E+00 | 2.661 |
| GSPT1 | -0.499 | 9.768 | -4.443 | 0.00E+00 | 0.00E+00 | 2.659 |
| CASP3 | -0.514 | 9.278 | -4.433 | 0.00E+00 | 0.00E+00 | 2.622 |
| THUMPD1 | -0.392 | 8.391 | -4.43 | 0.00E+00 | 0.00E+00 | 2.609 |
| CRB3 | 0.572 | 7.187 | 4.42 | 0.00E+00 | 0.00E+00 | 2.575 |
| ATAD1 | -0.565 | 9.607 | -4.42 | 0.00E+00 | 0.00E+00 | 2.572 |
| CPB1 | 0.673 | 6.346 | 4.413 | 0.00E+00 | 0.00E+00 | 2.55 |
| APH1A | 0.425 | 9.035 | 4.411 | 0.00E+00 | 0.00E+00 | 2.54 |
| G6PC3 | 0.658 | 8.628 | 4.409 | 0.00E+00 | 0.00E+00 | 2.534 |
| MESP1 | 0.572 | 6.491 | 4.405 | 0.00E+00 | 0.00E+00 | 2.516 |
| KIF1A | 0.466 | 5.468 | 4.404 | 0.00E+00 | 0.00E+00 | 2.514 |
| MDM4 | -0.466 | 7.16 | -4.405 | 0.00E+00 | 0.00E+00 | 2.513 |
| TCOF1 | 0.343 | 7.981 | 4.402 | 0.00E+00 | 0.00E+00 | 2.505 |
| RDX | -0.441 | 9.652 | -4.402 | 0.00E+00 | 0.00E+00 | 2.5 |
| MBNL1 | -0.412 | 9 | -4.399 | 0.00E+00 | 0.00E+00 | 2.492 |
| SUV39H2 | -0.465 | 6.413 | -4.397 | 0.00E+00 | 0.00E+00 | 2.485 |
| CES2 | 0.327 | 8.549 | 4.394 | 0.00E+00 | 0.00E+00 | 2.475 |
| RPUSD3 | 0.417 | 8.51 | 4.393 | 0.00E+00 | 0.00E+00 | 2.47 |
| ACY3 | 0.48 | 6.503 | 4.389 | 0.00E+00 | 0.00E+00 | 2.457 |
| OAZ1 | -0.194 | 14.697 | -4.386 | 0.00E+00 | 0.00E+00 | 2.439 |
| GNB4 | -0.671 | 7.984 | -4.384 | 0.00E+00 | 0.00E+00 | 2.431 |
| POLE3 | -0.46 | 10.035 | -4.383 | 0.00E+00 | 0.00E+00 | 2.43 |
| NOVA2 | 0.764 | 7.902 | 4.377 | 0.00E+00 | 0.00E+00 | 2.411 |
| ALS2CL | 0.414 | 6.884 | 4.372 | 0.00E+00 | 0.00E+00 | 2.39 |
| LIMK2 | -0.412 | 9.567 | -4.366 | 0.00E+00 | 0.00E+00 | 2.363 |
| EIF2S1 | -0.324 | 8.701 | -4.363 | 0.00E+00 | 0.00E+00 | 2.354 |
| SFPQ | -0.469 | 9.237 | -4.363 | 0.00E+00 | 0.00E+00 | 2.351 |
| HIVEP2 | -0.501 | 8.457 | -4.36 | 0.00E+00 | 0.00E+00 | 2.34 |
| PKIA | -0.532 | 7.262 | -4.357 | 0.00E+00 | 0.00E+00 | 2.33 |
| DAPP1 | -0.58 | 8.026 | -4.357 | 0.00E+00 | 0.00E+00 | 2.329 |
| PRPF18 | -0.368 | 8.727 | -4.351 | 0.00E+00 | 0.00E+00 | 2.307 |
| ZCCHC9 | -0.355 | 9.45 | -4.349 | 0.00E+00 | 0.00E+00 | 2.297 |
| HCFC2 | -0.38 | 7.748 | -4.346 | 0.00E+00 | 0.00E+00 | 2.286 |
| ATP1B3 | -0.482 | 10.823 | -4.342 | 0.00E+00 | 0.00E+00 | 2.27 |
| CLPP | 0.357 | 10.208 | 4.339 | 0.00E+00 | 0.00E+00 | 2.266 |
| MSI2 | 0.42 | 8.045 | 4.339 | 0.00E+00 | 0.00E+00 | 2.265 |
| PUSL1 | 0.342 | 8.931 | 4.339 | 0.00E+00 | 0.00E+00 | 2.263 |
| SUZ12 | -0.394 | 10.113 | -4.338 | 0.00E+00 | 0.00E+00 | 2.257 |
| KHSRP | 0.406 | 9.809 | 4.334 | 0.00E+00 | 0.00E+00 | 2.244 |
| DCK | -0.5 | 7.789 | -4.333 | 0.00E+00 | 0.00E+00 | 2.237 |
| IL18R1 | -0.727 | 6.591 | -4.332 | 0.00E+00 | 0.00E+00 | 2.235 |
| ATPAF2 | 0.438 | 8.126 | 4.33 | 0.00E+00 | 0.00E+00 | 2.23 |
| IFRD2 | 0.385 | 8.692 | 4.329 | 0.00E+00 | 0.00E+00 | 2.225 |
| HMG20B | 0.43 | 9.586 | 4.328 | 0.00E+00 | 0.00E+00 | 2.223 |
| ZNF569 | -0.58 | 6.195 | -4.327 | 0.00E+00 | 0.00E+00 | 2.216 |
| NCOA4 | -0.496 | 11.814 | -4.324 | 0.00E+00 | 0.00E+00 | 2.204 |
| THAP9 | -0.405 | 5.889 | -4.319 | 0.00E+00 | 0.00E+00 | 2.185 |
| TRPS1 | -0.439 | 6.626 | -4.315 | 0.00E+00 | 0.00E+00 | 2.169 |
| UVRAG | -0.392 | 7.835 | -4.312 | 0.00E+00 | 0.00E+00 | 2.158 |
| RNF38 | -0.449 | 8.437 | -4.312 | 0.00E+00 | 0.00E+00 | 2.156 |
| ILVBL | 0.444 | 10.586 | 4.31 | 0.00E+00 | 0.00E+00 | 2.153 |
| QPRT | 0.421 | 9.42 | 4.307 | 0.00E+00 | 0.00E+00 | 2.143 |
| NOL7 | -0.347 | 11.619 | -4.308 | 0.00E+00 | 0.00E+00 | 2.143 |
| MRPL50 | -0.406 | 9.366 | -4.308 | 0.00E+00 | 0.00E+00 | 2.143 |
| TRAPPC1 | 0.405 | 10.225 | 4.307 | 0.00E+00 | 0.00E+00 | 2.142 |
| ADSSL1 | 0.395 | 7.549 | 4.306 | 0.00E+00 | 0.00E+00 | 2.137 |
| LIN7B | 0.463 | 7.497 | 4.304 | 0.00E+00 | 0.00E+00 | 2.133 |
| ARF6 | -0.482 | 7.997 | -4.304 | 0.00E+00 | 0.00E+00 | 2.128 |
| ASMTL | 0.33 | 8.879 | 4.299 | 0.00E+00 | 0.00E+00 | 2.112 |
| ZNF267 | -0.584 | 7.333 | -4.297 | 0.00E+00 | 0.00E+00 | 2.099 |
| PPT1 | -0.49 | 10.793 | -4.295 | 0.00E+00 | 0.00E+00 | 2.093 |
| RASL12 | 0.513 | 7.263 | 4.293 | 0.00E+00 | 0.00E+00 | 2.09 |
| KCMF1 | -0.336 | 8.431 | -4.293 | 0.00E+00 | 0.00E+00 | 2.084 |
| DDX49 | 0.291 | 7.932 | 4.29 | 0.00E+00 | 0.00E+00 | 2.08 |
| PA2G4 | 0.422 | 9.984 | 4.289 | 0.00E+00 | 0.00E+00 | 2.073 |
| LHFPL5 | 0.52 | 6.12 | 4.288 | 0.00E+00 | 0.00E+00 | 2.072 |
| BUB3 | -0.297 | 9.659 | -4.287 | 0.00E+00 | 0.00E+00 | 2.063 |
| RPS6KA5 | -0.399 | 8.222 | -4.285 | 0.00E+00 | 0.00E+00 | 2.055 |
| C14orf142 | -0.484 | 7.509 | -4.283 | 0.00E+00 | 0.00E+00 | 2.048 |
| MAN1B1 | 0.326 | 9.369 | 4.28 | 0.00E+00 | 0.00E+00 | 2.042 |
| RAF1 | -0.288 | 10.36 | -4.28 | 0.00E+00 | 0.00E+00 | 2.038 |
| COX7A1 | 0.619 | 9.31 | 4.279 | 0.00E+00 | 0.00E+00 | 2.035 |
| TCP10L | 0.423 | 5.76 | 4.278 | 0.00E+00 | 0.00E+00 | 2.032 |
| ZNF227 | -0.39 | 7.121 | -4.278 | 0.00E+00 | 0.00E+00 | 2.028 |
| CDC16 | -0.322 | 10.658 | -4.276 | 0.00E+00 | 0.00E+00 | 2.021 |
| PRKD3 | -0.345 | 7.621 | -4.274 | 0.00E+00 | 0.00E+00 | 2.013 |
| SLC27A5 | 0.474 | 7.394 | 4.27 | 0.00E+00 | 0.00E+00 | 2.002 |
| AUH | -0.36 | 9.268 | -4.27 | 0.00E+00 | 0.00E+00 | 2 |
| WWOX | 0.24 | 6.547 | 4.268 | 0.00E+00 | 0.00E+00 | 1.996 |
| DONSON | -0.3 | 7.877 | -4.263 | 0.00E+00 | 0.00E+00 | 1.972 |
| COL6A1 | 0.57 | 10.706 | 4.259 | 0.00E+00 | 0.00E+00 | 1.962 |
| HMBS | 0.476 | 9.917 | 4.257 | 0.00E+00 | 0.00E+00 | 1.955 |
| BEX1 | -0.81 | 7.927 | -4.258 | 0.00E+00 | 0.00E+00 | 1.953 |
| CLK1 | -0.6 | 9.218 | -4.254 | 0.00E+00 | 0.00E+00 | 1.938 |
| OGG1 | 0.31 | 7.151 | 4.252 | 0.00E+00 | 0.00E+00 | 1.935 |
| RDH11 | -0.381 | 9.879 | -4.244 | 0.00E+00 | 0.00E+00 | 1.902 |
| DBR1 | -0.365 | 7.325 | -4.243 | 0.00E+00 | 0.00E+00 | 1.897 |
| MPI | 0.369 | 8.188 | 4.241 | 0.00E+00 | 0.00E+00 | 1.895 |
| GTPBP4 | -0.469 | 9.969 | -4.239 | 0.00E+00 | 0.00E+00 | 1.881 |
| ASB13 | 0.351 | 7.433 | 4.232 | 0.00E+00 | 0.00E+00 | 1.86 |
| UFD1L | 0.312 | 9.8 | 4.231 | 0.00E+00 | 0.00E+00 | 1.858 |
| FRAS1 | 0.337 | 6.29 | 4.23 | 0.00E+00 | 0.00E+00 | 1.854 |
| EVI2A | -0.442 | 7.512 | -4.23 | 0.00E+00 | 0.00E+00 | 1.85 |
| DYRK1B | 0.5 | 8.231 | 4.228 | 0.00E+00 | 0.00E+00 | 1.845 |
| SGTB | -0.379 | 6.707 | -4.227 | 0.00E+00 | 0.00E+00 | 1.839 |
| APOA4 | 0.472 | 5.037 | 4.226 | 0.00E+00 | 0.00E+00 | 1.837 |
| PPIF | -0.405 | 8.713 | -4.226 | 0.00E+00 | 0.00E+00 | 1.835 |
| PDCD6 | -0.295 | 11.14 | -4.225 | 0.00E+00 | 0.00E+00 | 1.829 |
| UBE2N | -0.425 | 8.998 | -4.223 | 0.00E+00 | 0.00E+00 | 1.821 |
| ALX3 | 0.506 | 5.636 | 4.221 | 0.00E+00 | 0.00E+00 | 1.82 |
| KIRREL | 0.492 | 6.955 | 4.219 | 0.00E+00 | 0.00E+00 | 1.814 |
| HINT3 | -0.401 | 8.061 | -4.219 | 0.00E+00 | 0.00E+00 | 1.807 |
| GGH | -0.639 | 9.638 | -4.218 | 0.00E+00 | 0.00E+00 | 1.805 |
| FBXO17 | 0.458 | 7.385 | 4.213 | 0.00E+00 | 0.00E+00 | 1.791 |
| MC5R | 0.593 | 5.83 | 4.213 | 0.00E+00 | 0.00E+00 | 1.788 |
| MRPL19 | -0.426 | 9.085 | -4.205 | 0.00E+00 | 0.00E+00 | 1.755 |
| SELT | -0.415 | 9.708 | -4.204 | 0.00E+00 | 0.00E+00 | 1.754 |
| PRPF39 | -0.397 | 7.791 | -4.203 | 0.00E+00 | 0.00E+00 | 1.749 |
| PDE4DIP | -0.332 | 7.591 | -4.197 | 0.00E+00 | 0.00E+00 | 1.726 |
| SESTD1 | -0.401 | 7.466 | -4.197 | 0.00E+00 | 0.00E+00 | 1.726 |
| FGF9 | 0.496 | 5.694 | 4.192 | 0.00E+00 | 0.00E+00 | 1.711 |
| GMNN | -0.475 | 9.097 | -4.193 | 0.00E+00 | 0.00E+00 | 1.71 |
| COL4A5 | 0.535 | 7.916 | 4.191 | 0.00E+00 | 0.00E+00 | 1.707 |
| C14orf132 | 0.558 | 6.993 | 4.191 | 0.00E+00 | 0.00E+00 | 1.707 |
| MRPS12 | 0.269 | 9.292 | 4.188 | 0.00E+00 | 0.00E+00 | 1.695 |
| WDTC1 | 0.566 | 7.189 | 4.187 | 0.00E+00 | 0.00E+00 | 1.691 |
| MRPS30 | -0.33 | 8.472 | -4.184 | 0.00E+00 | 0.00E+00 | 1.676 |
| ATF4 | -0.327 | 12.356 | -4.183 | 0.00E+00 | 0.00E+00 | 1.673 |
| ZBTB37 | 0.371 | 5.907 | 4.182 | 0.00E+00 | 0.00E+00 | 1.673 |
| JMJD1C | -0.493 | 8.284 | -4.18 | 0.00E+00 | 0.00E+00 | 1.664 |
| SOX15 | 0.5 | 5.574 | 4.177 | 0.00E+00 | 0.00E+00 | 1.656 |
| SNAPC3 | -0.31 | 7.438 | -4.171 | 0.00E+00 | 0.00E+00 | 1.63 |
| OPN3 | -0.462 | 8.814 | -4.17 | 0.00E+00 | 0.00E+00 | 1.626 |
| EEF1D | 0.299 | 11.458 | 4.168 | 0.00E+00 | 0.00E+00 | 1.622 |
| SH2D3A | 0.472 | 7.592 | 4.165 | 0.00E+00 | 0.00E+00 | 1.61 |
| TAF6 | 0.383 | 9.395 | 4.164 | 0.00E+00 | 0.00E+00 | 1.606 |
| CD34 | 0.406 | 7.696 | 4.163 | 0.00E+00 | 0.00E+00 | 1.605 |
| C18orf8 | -0.366 | 9.261 | -4.164 | 0.00E+00 | 0.00E+00 | 1.604 |
| MTMR9 | -0.506 | 7.836 | -4.162 | 0.00E+00 | 0.00E+00 | 1.597 |
| SIGIRR | 0.338 | 8.329 | 4.153 | 0.00E+00 | 0.00E+00 | 1.568 |
| PDCD6IP | -0.278 | 9.139 | -4.154 | 0.00E+00 | 0.00E+00 | 1.568 |
| ATP11C | -0.369 | 6.827 | -4.149 | 0.00E+00 | 0.00E+00 | 1.55 |
| MLANA | 0.561 | 5.21 | 4.148 | 0.00E+00 | 0.00E+00 | 1.549 |
| NARFL | 0.465 | 8.048 | 4.147 | 0.00E+00 | 0.00E+00 | 1.546 |
| H1FX | 0.449 | 9.535 | 4.145 | 0.00E+00 | 0.00E+00 | 1.539 |
| MRPL22 | 0.36 | 10.344 | 4.143 | 0.00E+00 | 0.00E+00 | 1.531 |
| WDR5B | -0.391 | 6.351 | -4.144 | 0.00E+00 | 0.00E+00 | 1.529 |
| PCNA | -0.378 | 9.749 | -4.139 | 0.00E+00 | 0.00E+00 | 1.51 |
| ZNF213 | 0.427 | 7.775 | 4.137 | 0.00E+00 | 0.00E+00 | 1.508 |
| ZNF324 | 0.517 | 8.051 | 4.134 | 0.00E+00 | 0.00E+00 | 1.496 |
| ICAM5 | 0.499 | 5.994 | 4.133 | 0.00E+00 | 0.00E+00 | 1.495 |
| CACNA1C | 0.327 | 6.654 | 4.13 | 0.00E+00 | 0.00E+00 | 1.484 |
| DPM2 | 0.4 | 8.953 | 4.13 | 0.00E+00 | 0.00E+00 | 1.482 |
| S100Z | 0.463 | 6.441 | 4.129 | 0.00E+00 | 0.00E+00 | 1.479 |
| ISYNA1 | 0.429 | 10.085 | 4.128 | 0.00E+00 | 0.00E+00 | 1.474 |
| SNAPC1 | -0.507 | 7.029 | -4.126 | 0.00E+00 | 0.00E+00 | 1.466 |
| BCL10 | -0.408 | 6.785 | -4.125 | 0.00E+00 | 0.00E+00 | 1.463 |
| RAB11A | -0.47 | 9.853 | -4.125 | 0.00E+00 | 0.00E+00 | 1.459 |
| ZNF574 | 0.496 | 7.781 | 4.123 | 0.00E+00 | 0.00E+00 | 1.457 |
| TBXA2R | 0.344 | 7.469 | 4.122 | 0.00E+00 | 0.00E+00 | 1.454 |
| GFM2 | -0.515 | 8.002 | -4.122 | 0.00E+00 | 0.00E+00 | 1.45 |
| COL5A1 | 0.494 | 9.204 | 4.12 | 0.00E+00 | 0.00E+00 | 1.446 |
| SH3BGRL | -0.489 | 9.799 | -4.121 | 0.00E+00 | 0.00E+00 | 1.444 |
| CUEDC2 | 0.297 | 9.776 | 4.118 | 0.00E+00 | 0.00E+00 | 1.439 |
| ZMAT2 | 0.422 | 10.196 | 4.118 | 0.00E+00 | 0.00E+00 | 1.439 |
| ZNF214 | -0.488 | 5.038 | -4.119 | 0.00E+00 | 0.00E+00 | 1.438 |
| RUFY2 | -0.476 | 6.468 | -4.117 | 0.00E+00 | 0.00E+00 | 1.431 |
| CEBPZ | -0.442 | 9.648 | -4.117 | 0.00E+00 | 0.00E+00 | 1.431 |
| KCTD18 | -0.484 | 7.034 | -4.115 | 0.00E+00 | 0.00E+00 | 1.425 |
| KLHL3 | -0.448 | 7.611 | -4.114 | 0.00E+00 | 0.00E+00 | 1.422 |
| TRIM37 | -0.396 | 7.945 | -4.114 | 0.00E+00 | 0.00E+00 | 1.42 |
| TAF1A | -0.38 | 6.998 | -4.109 | 0.00E+00 | 0.00E+00 | 1.404 |
| SNRK | -0.46 | 8.045 | -4.107 | 0.00E+00 | 0.00E+00 | 1.394 |
| XRN1 | -0.392 | 8.24 | -4.106 | 0.00E+00 | 0.00E+00 | 1.393 |
| RNF14 | -0.354 | 8.588 | -4.104 | 0.00E+00 | 0.00E+00 | 1.384 |
| CLEC2D | -0.385 | 7.472 | -4.102 | 0.00E+00 | 0.00E+00 | 1.377 |
| FZD7 | -0.394 | 7.195 | -4.1 | 0.00E+00 | 0.00E+00 | 1.37 |
| AP2B1 | 0.49 | 9.053 | 4.098 | 0.00E+00 | 0.00E+00 | 1.365 |
| PPFIA1 | -0.365 | 8.558 | -4.096 | 0.00E+00 | 0.00E+00 | 1.354 |
| ARHGAP5 | -0.335 | 7.582 | -4.096 | 0.00E+00 | 0.00E+00 | 1.354 |
| SLC38A1 | -0.464 | 11.135 | -4.095 | 0.00E+00 | 0.00E+00 | 1.351 |
| RUVBL1 | 0.364 | 8.036 | 4.093 | 0.00E+00 | 0.00E+00 | 1.349 |
| ZNF217 | -0.44 | 9.914 | -4.093 | 0.00E+00 | 0.00E+00 | 1.345 |
| LASP1 | -0.453 | 11.417 | -4.092 | 0.00E+00 | 0.00E+00 | 1.341 |
| INPP5F | -0.297 | 7.679 | -4.092 | 0.00E+00 | 0.00E+00 | 1.339 |
| B3GAT3 | 0.353 | 8.513 | 4.089 | 0.00E+00 | 0.00E+00 | 1.334 |
| CHRNB1 | 0.489 | 6.928 | 4.089 | 0.00E+00 | 0.00E+00 | 1.333 |
| EME1 | 0.391 | 6.575 | 4.088 | 0.00E+00 | 0.00E+00 | 1.331 |
| ZNF248 | -0.44 | 6.596 | -4.089 | 0.00E+00 | 0.00E+00 | 1.329 |
| RORC | 0.422 | 5.505 | 4.08 | 0.00E+00 | 0.00E+00 | 1.3 |
| EMID1 | 0.538 | 6.383 | 4.079 | 0.00E+00 | 0.00E+00 | 1.297 |
| ZNF274 | -0.311 | 8.299 | -4.076 | 0.00E+00 | 0.00E+00 | 1.284 |
| CASP7 | -0.364 | 8.489 | -4.075 | 0.00E+00 | 0.00E+00 | 1.278 |
| NIPBL | -0.436 | 8.383 | -4.071 | 0.00E+00 | 0.00E+00 | 1.265 |
| RNGTT | -0.515 | 8.126 | -4.068 | 0.00E+00 | 0.00E+00 | 1.255 |
| EPHB3 | 0.589 | 6.787 | 4.067 | 0.00E+00 | 0.00E+00 | 1.255 |
| RABGAP1L | -0.356 | 7.357 | -4.065 | 0.00E+00 | 0.00E+00 | 1.244 |
| SH3TC1 | 0.396 | 7.476 | 4.064 | 0.00E+00 | 0.00E+00 | 1.242 |
| RPL7 | -0.622 | 12.077 | -4.063 | 0.00E+00 | 0.00E+00 | 1.235 |
| ITGB1BP1 | -0.363 | 9.137 | -4.059 | 0.00E+00 | 0.00E+00 | 1.22 |
| CCDC12 | 0.277 | 10.63 | 4.057 | 0.00E+00 | 0.00E+00 | 1.217 |
| ZNF430 | -0.332 | 7.555 | -4.057 | 0.00E+00 | 0.00E+00 | 1.212 |
| COL4A2 | 0.409 | 11.546 | 4.051 | 0.00E+00 | 0.00E+00 | 1.197 |
| VPS29 | -0.327 | 10.475 | -4.052 | 0.00E+00 | 0.00E+00 | 1.196 |
| ENPP4 | -0.481 | 7.514 | -4.05 | 0.00E+00 | 0.00E+00 | 1.188 |
| NCR2 | 0.507 | 5.912 | 4.049 | 0.00E+00 | 0.00E+00 | 1.187 |
| UPF3A | -0.253 | 8.748 | -4.049 | 0.00E+00 | 0.00E+00 | 1.185 |
| CPSF6 | -0.399 | 7.793 | -4.048 | 0.00E+00 | 0.00E+00 | 1.182 |
| TRIM47 | 0.434 | 7.74 | 4.047 | 0.00E+00 | 0.00E+00 | 1.181 |
| RNF139 | -0.35 | 8.697 | -4.046 | 0.00E+00 | 0.00E+00 | 1.173 |
| PDHA1 | -0.269 | 10.494 | -4.042 | 0.00E+00 | 0.00E+00 | 1.158 |
| RNF128 | -0.477 | 5.964 | -4.037 | 0.00E+00 | 0.00E+00 | 1.141 |
| BACH1 | -0.365 | 6.884 | -4.034 | 0.00E+00 | 0.00E+00 | 1.132 |
| NTRK2 | 0.426 | 5.268 | 4.033 | 0.00E+00 | 0.00E+00 | 1.13 |
| FHOD1 | 0.394 | 8.858 | 4.031 | 0.00E+00 | 0.00E+00 | 1.124 |
| GCNT1 | -0.497 | 7.228 | -4.031 | 0.00E+00 | 0.00E+00 | 1.12 |
| KCTD3 | -0.477 | 9.087 | -4.03 | 0.00E+00 | 0.00E+00 | 1.115 |
| SLC29A4 | 0.454 | 6.28 | 4.025 | 0.00E+00 | 0.00E+00 | 1.101 |
| RBBP9 | -0.286 | 6.322 | -4.025 | 0.00E+00 | 0.00E+00 | 1.097 |
| DEAF1 | 0.284 | 8.395 | 4.02 | 0.00E+00 | 0.00E+00 | 1.085 |
| DNMT1 | -0.383 | 10.614 | -4.016 | 0.00E+00 | 0.00E+00 | 1.065 |
| GNAQ | -0.348 | 8.635 | -4.014 | 0.00E+00 | 0.00E+00 | 1.06 |
| RUVBL2 | 0.331 | 10.045 | 4.011 | 0.00E+00 | 0.00E+00 | 1.053 |
| NKIRAS1 | -0.567 | 7.38 | -4.008 | 0.00E+00 | 0.00E+00 | 1.036 |
| POMT1 | 0.315 | 8.996 | 4.001 | 0.00E+00 | 0.00E+00 | 1.017 |
| MAN1A1 | -0.527 | 8.267 | -4.002 | 0.00E+00 | 0.00E+00 | 1.015 |
| PSMA1 | -0.248 | 10.346 | -3.999 | 0.00E+00 | 0.00E+00 | 1.006 |
| ZNF367 | -0.427 | 5.61 | -3.993 | 0.00E+00 | 0.00E+00 | 0.985 |
| ATP5G1 | 0.339 | 10.208 | 3.991 | 0.00E+00 | 0.00E+00 | 0.98 |
| RPS6KB1 | -0.352 | 8.318 | -3.991 | 0.00E+00 | 0.00E+00 | 0.978 |
| CUL3 | -0.399 | 6.625 | -3.99 | 0.00E+00 | 0.00E+00 | 0.973 |
| VDAC2 | -0.275 | 10.597 | -3.988 | 0.00E+00 | 0.00E+00 | 0.965 |
| RSBN1 | -0.41 | 8.08 | -3.986 | 0.00E+00 | 0.00E+00 | 0.959 |
| CGRRF1 | -0.409 | 7.918 | -3.986 | 0.00E+00 | 0.00E+00 | 0.957 |
| ZNF395 | 0.444 | 9.932 | 3.984 | 0.00E+00 | 0.00E+00 | 0.957 |
| SLC39A7 | -0.541 | 8.828 | -3.985 | 0.00E+00 | 0.00E+00 | 0.955 |
| ROBO4 | 0.367 | 7.807 | 3.982 | 0.00E+00 | 0.00E+00 | 0.949 |
| ATP5D | 0.316 | 10.223 | 3.98 | 0.00E+00 | 0.00E+00 | 0.941 |
| VIT | 0.615 | 5.832 | 3.979 | 0.00E+00 | 0.00E+00 | 0.936 |
| MTHFD2 | -0.494 | 8.8 | -3.976 | 0.00E+00 | 1.00E-03 | 0.924 |
| POLR2L | 0.377 | 10.551 | 3.973 | 0.00E+00 | 1.00E-03 | 0.915 |
| NRG4 | 0.457 | 5.204 | 3.971 | 0.00E+00 | 1.00E-03 | 0.909 |
| SRD5A2 | 0.452 | 4.886 | 3.963 | 0.00E+00 | 1.00E-03 | 0.881 |
| TERF1 | -0.273 | 7.741 | -3.964 | 0.00E+00 | 1.00E-03 | 0.88 |
| ZNF43 | -0.444 | 6.76 | -3.961 | 0.00E+00 | 1.00E-03 | 0.871 |
| CLEC11A | 0.48 | 8.78 | 3.96 | 0.00E+00 | 1.00E-03 | 0.87 |
| TRIM23 | -0.459 | 6.86 | -3.961 | 0.00E+00 | 1.00E-03 | 0.869 |
| NDUFA7 | 0.32 | 10.806 | 3.959 | 0.00E+00 | 1.00E-03 | 0.865 |
| TOMM20 | -0.355 | 10.489 | -3.958 | 0.00E+00 | 1.00E-03 | 0.861 |
| GBP3 | -0.547 | 8.603 | -3.957 | 0.00E+00 | 1.00E-03 | 0.857 |
| RASA2 | -0.429 | 7.032 | -3.956 | 0.00E+00 | 1.00E-03 | 0.851 |
| NCOA6 | -0.308 | 9.697 | -3.952 | 0.00E+00 | 1.00E-03 | 0.838 |
| IRF3 | 0.328 | 8.865 | 3.95 | 0.00E+00 | 1.00E-03 | 0.836 |
| SPRED1 | -0.444 | 8.201 | -3.95 | 0.00E+00 | 1.00E-03 | 0.833 |
| CMIP | 0.463 | 8.506 | 3.949 | 0.00E+00 | 1.00E-03 | 0.83 |
| B3GALT4 | 0.464 | 8.745 | 3.948 | 0.00E+00 | 1.00E-03 | 0.826 |
| CHCHD7 | -0.298 | 8.768 | -3.946 | 0.00E+00 | 1.00E-03 | 0.817 |
| MCTS1 | -0.458 | 8.908 | -3.946 | 0.00E+00 | 1.00E-03 | 0.816 |
| TBC1D22A | 0.275 | 8.243 | 3.942 | 0.00E+00 | 1.00E-03 | 0.805 |
| TRAF5 | -0.347 | 7.874 | -3.942 | 0.00E+00 | 1.00E-03 | 0.804 |
| BZW1 | -0.29 | 9.206 | -3.941 | 0.00E+00 | 1.00E-03 | 0.799 |
| KLF3 | -0.39 | 8.731 | -3.94 | 0.00E+00 | 1.00E-03 | 0.797 |
| DTX3 | 0.366 | 7.552 | 3.936 | 0.00E+00 | 1.00E-03 | 0.786 |
| CD1D | -0.433 | 5.988 | -3.937 | 0.00E+00 | 1.00E-03 | 0.784 |
| PTTG1 | 0.55 | 9.562 | 3.935 | 0.00E+00 | 1.00E-03 | 0.783 |
| CD59 | -0.41 | 10.276 | -3.936 | 0.00E+00 | 1.00E-03 | 0.78 |
| LDHB | -0.623 | 11.808 | -3.935 | 0.00E+00 | 1.00E-03 | 0.779 |
| STYK1 | -0.502 | 6.821 | -3.934 | 0.00E+00 | 1.00E-03 | 0.775 |
| RBM15B | 0.316 | 7.748 | 3.93 | 0.00E+00 | 1.00E-03 | 0.765 |
| FZR1 | 0.345 | 7.62 | 3.928 | 0.00E+00 | 1.00E-03 | 0.758 |
| SRPK2 | -0.378 | 7.898 | -3.928 | 0.00E+00 | 1.00E-03 | 0.754 |
| RFC5 | -0.397 | 7.898 | -3.927 | 0.00E+00 | 1.00E-03 | 0.75 |
| TMF1 | -0.416 | 8.259 | -3.925 | 0.00E+00 | 1.00E-03 | 0.742 |
| NR1D2 | -0.478 | 7.33 | -3.921 | 0.00E+00 | 1.00E-03 | 0.73 |
| TIAM1 | 0.436 | 7.649 | 3.92 | 0.00E+00 | 1.00E-03 | 0.729 |
| DPT | 0.612 | 7.78 | 3.919 | 0.00E+00 | 1.00E-03 | 0.726 |
| FREM1 | 0.46 | 5.475 | 3.918 | 0.00E+00 | 1.00E-03 | 0.723 |
| CREB5 | -0.415 | 7.982 | -3.919 | 0.00E+00 | 1.00E-03 | 0.721 |
| MOV10 | 0.453 | 8.466 | 3.915 | 0.00E+00 | 1.00E-03 | 0.713 |
| SLC29A3 | 0.388 | 7.714 | 3.912 | 0.00E+00 | 1.00E-03 | 0.702 |
| HPSE | -0.684 | 7.461 | -3.911 | 0.00E+00 | 1.00E-03 | 0.694 |
| CIRH1A | 0.339 | 9.391 | 3.908 | 0.00E+00 | 1.00E-03 | 0.688 |
| LDOC1 | 0.604 | 7.726 | 3.905 | 0.00E+00 | 1.00E-03 | 0.677 |
| HBE1 | 0.656 | 6.084 | 3.899 | 0.00E+00 | 1.00E-03 | 0.654 |
| STARD13 | -0.377 | 7.043 | -3.899 | 0.00E+00 | 1.00E-03 | 0.653 |
| GZMM | 0.404 | 7.518 | 3.898 | 0.00E+00 | 1.00E-03 | 0.652 |
| VCP | -0.296 | 11.032 | -3.893 | 0.00E+00 | 1.00E-03 | 0.632 |
| CDAN1 | 0.313 | 7.3 | 3.892 | 0.00E+00 | 1.00E-03 | 0.631 |
| IPO4 | 0.371 | 8.14 | 3.891 | 0.00E+00 | 1.00E-03 | 0.628 |
| LGALS4 | 0.46 | 6.028 | 3.887 | 0.00E+00 | 1.00E-03 | 0.614 |
| SYAP1 | -0.42 | 9.311 | -3.886 | 0.00E+00 | 1.00E-03 | 0.605 |
| ZNF230 | -0.399 | 6.351 | -3.885 | 0.00E+00 | 1.00E-03 | 0.602 |
| AP1M2 | 0.504 | 7.886 | 3.882 | 0.00E+00 | 1.00E-03 | 0.597 |
| BRF1 | 0.296 | 8.063 | 3.882 | 0.00E+00 | 1.00E-03 | 0.595 |
| NDUFB2 | 0.368 | 12.279 | 3.881 | 0.00E+00 | 1.00E-03 | 0.592 |
| IPP | -0.362 | 7.162 | -3.881 | 0.00E+00 | 1.00E-03 | 0.59 |
| SPRY3 | 0.424 | 5.933 | 3.879 | 0.00E+00 | 1.00E-03 | 0.587 |
| COPE | 0.271 | 9.959 | 3.877 | 0.00E+00 | 1.00E-03 | 0.579 |
| MTX1 | 0.298 | 9.547 | 3.875 | 0.00E+00 | 1.00E-03 | 0.571 |
| BAALC | 0.362 | 7.251 | 3.873 | 0.00E+00 | 1.00E-03 | 0.566 |
| WNT9B | 0.458 | 5.441 | 3.873 | 0.00E+00 | 1.00E-03 | 0.564 |
| SLC27A2 | -0.569 | 8.191 | -3.873 | 0.00E+00 | 1.00E-03 | 0.561 |
| FCHO2 | -0.523 | 7.687 | -3.872 | 0.00E+00 | 1.00E-03 | 0.557 |
| FGD1 | 0.418 | 7.44 | 3.869 | 0.00E+00 | 1.00E-03 | 0.55 |
| CRYZL1 | -0.25 | 7.785 | -3.868 | 0.00E+00 | 1.00E-03 | 0.543 |
| THAP6 | -0.42 | 7.381 | -3.866 | 0.00E+00 | 1.00E-03 | 0.538 |
| MAPT | 0.475 | 6.376 | 3.865 | 0.00E+00 | 1.00E-03 | 0.538 |
| OSGEPL1 | -0.51 | 6.761 | -3.863 | 0.00E+00 | 1.00E-03 | 0.525 |
| CUL2 | -0.429 | 8.806 | -3.86 | 0.00E+00 | 1.00E-03 | 0.516 |
| PCTP | -0.312 | 9.836 | -3.855 | 0.00E+00 | 1.00E-03 | 0.498 |
| BCAT2 | 0.428 | 8.517 | 3.854 | 0.00E+00 | 1.00E-03 | 0.497 |
| NEK6 | 0.38 | 8.467 | 3.854 | 0.00E+00 | 1.00E-03 | 0.497 |
| THUMPD3 | -0.25 | 8.011 | -3.853 | 0.00E+00 | 1.00E-03 | 0.492 |
| RPL36 | 0.33 | 12.04 | 3.852 | 0.00E+00 | 1.00E-03 | 0.492 |
| NGLY1 | -0.307 | 9.464 | -3.852 | 0.00E+00 | 1.00E-03 | 0.49 |
| P2RY10 | -0.485 | 5.818 | -3.851 | 0.00E+00 | 1.00E-03 | 0.486 |
| POLR2C | -0.286 | 9.414 | -3.851 | 0.00E+00 | 1.00E-03 | 0.486 |
| ARID1B | 0.291 | 7.106 | 3.85 | 0.00E+00 | 1.00E-03 | 0.485 |
| PAPOLA | -0.426 | 9.701 | -3.849 | 0.00E+00 | 1.00E-03 | 0.477 |
| CASP1 | -0.465 | 9.272 | -3.847 | 0.00E+00 | 1.00E-03 | 0.471 |
| RAPGEF4 | -0.438 | 5.329 | -3.846 | 0.00E+00 | 1.00E-03 | 0.468 |
| SLC22A15 | -0.393 | 6.616 | -3.845 | 0.00E+00 | 1.00E-03 | 0.465 |
| JAK3 | 0.377 | 7.141 | 3.844 | 0.00E+00 | 1.00E-03 | 0.465 |
| MAP1LC3A | 0.452 | 8.058 | 3.844 | 0.00E+00 | 1.00E-03 | 0.464 |
| GYG2 | 0.558 | 6.001 | 3.844 | 0.00E+00 | 1.00E-03 | 0.464 |
| PPA2 | -0.433 | 8.927 | -3.845 | 0.00E+00 | 1.00E-03 | 0.463 |
| SAP30 | -0.41 | 8.862 | -3.843 | 0.00E+00 | 1.00E-03 | 0.456 |
| ARR3 | 0.418 | 4.918 | 3.84 | 0.00E+00 | 1.00E-03 | 0.452 |
| TMEM39B | 0.361 | 8.952 | 3.84 | 0.00E+00 | 1.00E-03 | 0.45 |
| ZNF382 | -0.46 | 5.538 | -3.841 | 0.00E+00 | 1.00E-03 | 0.449 |
| MTO1 | -0.252 | 8.065 | -3.841 | 0.00E+00 | 1.00E-03 | 0.449 |
| NKAP | -0.432 | 8.292 | -3.84 | 0.00E+00 | 1.00E-03 | 0.446 |
| TACR1 | 0.478 | 5.791 | 3.836 | 0.00E+00 | 1.00E-03 | 0.436 |
| IMMT | -0.312 | 9.962 | -3.835 | 0.00E+00 | 1.00E-03 | 0.431 |
| ZNF623 | -0.308 | 6.499 | -3.832 | 0.00E+00 | 1.00E-03 | 0.418 |
| ZNF468 | -0.508 | 7.778 | -3.831 | 0.00E+00 | 1.00E-03 | 0.415 |
| CNOT6 | -0.354 | 8.619 | -3.83 | 0.00E+00 | 1.00E-03 | 0.413 |
| HBP1 | -0.429 | 9.549 | -3.829 | 0.00E+00 | 1.00E-03 | 0.408 |
| PFKM | 0.338 | 8.838 | 3.827 | 0.00E+00 | 1.00E-03 | 0.407 |
| ITGAV | -0.578 | 9.787 | -3.828 | 0.00E+00 | 1.00E-03 | 0.407 |
| PCF11 | -0.463 | 7.027 | -3.817 | 0.00E+00 | 1.00E-03 | 0.369 |
| LAS1L | 0.367 | 9.49 | 3.816 | 0.00E+00 | 1.00E-03 | 0.368 |
| AP3B1 | -0.296 | 9.856 | -3.816 | 0.00E+00 | 1.00E-03 | 0.365 |
| SNAPC5 | -0.308 | 7.663 | -3.816 | 0.00E+00 | 1.00E-03 | 0.364 |
| FOLR1 | 0.416 | 8.564 | 3.814 | 0.00E+00 | 1.00E-03 | 0.361 |
| SAMD8 | -0.314 | 7.283 | -3.814 | 0.00E+00 | 1.00E-03 | 0.356 |
| MCFD2 | -0.384 | 9.333 | -3.809 | 0.00E+00 | 1.00E-03 | 0.341 |
| PGBD2 | -0.515 | 7.816 | -3.804 | 0.00E+00 | 1.00E-03 | 0.324 |
| MOSPD3 | 0.385 | 7.109 | 3.803 | 0.00E+00 | 1.00E-03 | 0.322 |
| CLTB | 0.376 | 8.514 | 3.801 | 0.00E+00 | 1.00E-03 | 0.317 |
| CORO1C | -0.482 | 9.665 | -3.798 | 0.00E+00 | 1.00E-03 | 0.304 |
| NME4 | 0.427 | 9.692 | 3.795 | 0.00E+00 | 1.00E-03 | 0.294 |
| DZIP1 | 0.432 | 6.414 | 3.791 | 0.00E+00 | 1.00E-03 | 0.281 |
| EFNA1 | 0.421 | 9.506 | 3.791 | 0.00E+00 | 1.00E-03 | 0.281 |
| PAX4 | 0.439 | 6.736 | 3.789 | 0.00E+00 | 1.00E-03 | 0.276 |
| CIB1 | 0.34 | 10.748 | 3.789 | 0.00E+00 | 1.00E-03 | 0.275 |
| RASGRF2 | 0.529 | 6.167 | 3.788 | 0.00E+00 | 1.00E-03 | 0.27 |
| ANXA1 | -0.476 | 13.377 | -3.788 | 0.00E+00 | 1.00E-03 | 0.269 |
| ADAMTS3 | -0.645 | 6.074 | -3.787 | 0.00E+00 | 1.00E-03 | 0.264 |
| PLXDC1 | 0.41 | 6.475 | 3.785 | 0.00E+00 | 1.00E-03 | 0.261 |
| PEX13 | -0.248 | 7.768 | -3.783 | 0.00E+00 | 1.00E-03 | 0.25 |
| CYGB | 0.464 | 7.415 | 3.781 | 0.00E+00 | 1.00E-03 | 0.248 |
| SLC30A9 | -0.401 | 8.71 | -3.781 | 0.00E+00 | 1.00E-03 | 0.244 |
| SRP72 | -0.387 | 9.948 | -3.779 | 0.00E+00 | 1.00E-03 | 0.237 |
| PHYH | -0.544 | 8.496 | -3.778 | 0.00E+00 | 1.00E-03 | 0.235 |
| POSTN | -0.547 | 8.06 | -3.778 | 0.00E+00 | 1.00E-03 | 0.233 |
| TSSC1 | 0.393 | 8.685 | 3.776 | 0.00E+00 | 1.00E-03 | 0.231 |
| SYNGR1 | 0.378 | 7.601 | 3.776 | 0.00E+00 | 1.00E-03 | 0.229 |
| PXMP4 | 0.321 | 7.288 | 3.775 | 0.00E+00 | 1.00E-03 | 0.227 |
| PIGK | -0.39 | 7.898 | -3.774 | 0.00E+00 | 1.00E-03 | 0.221 |
| PDE1A | 0.38 | 5.184 | 3.773 | 0.00E+00 | 1.00E-03 | 0.22 |
| GPRC6A | 0.624 | 4.862 | 3.772 | 0.00E+00 | 1.00E-03 | 0.218 |
| ATE1 | -0.395 | 6.442 | -3.77 | 0.00E+00 | 1.00E-03 | 0.206 |
| ARMCX5 | -0.387 | 7.802 | -3.767 | 0.00E+00 | 1.00E-03 | 0.196 |
| GSTO2 | 0.503 | 5.963 | 3.766 | 0.00E+00 | 1.00E-03 | 0.196 |
| EXOSC8 | -0.29 | 9.986 | -3.766 | 0.00E+00 | 1.00E-03 | 0.193 |
| PIGQ | 0.39 | 8.185 | 3.764 | 0.00E+00 | 1.00E-03 | 0.19 |
| GPR65 | -0.461 | 6.934 | -3.764 | 0.00E+00 | 1.00E-03 | 0.188 |
| C14orf93 | 0.308 | 8.177 | 3.763 | 0.00E+00 | 1.00E-03 | 0.188 |
| MRPL20 | 0.274 | 10.933 | 3.763 | 0.00E+00 | 1.00E-03 | 0.187 |
| SDC4 | 0.415 | 8.814 | 3.761 | 0.00E+00 | 1.00E-03 | 0.181 |
| PTCRA | 0.366 | 7.513 | 3.761 | 0.00E+00 | 1.00E-03 | 0.178 |
| KPTN | 0.366 | 7.165 | 3.761 | 0.00E+00 | 1.00E-03 | 0.178 |
| KIF21A | -0.509 | 7.759 | -3.761 | 0.00E+00 | 1.00E-03 | 0.177 |
| TIMM17A | -0.3 | 9.16 | -3.76 | 0.00E+00 | 1.00E-03 | 0.173 |
| KYNU | -0.605 | 8.637 | -3.759 | 0.00E+00 | 1.00E-03 | 0.17 |
| GUCA1A | 0.442 | 5.367 | 3.756 | 0.00E+00 | 1.00E-03 | 0.164 |
| C3orf14 | -0.403 | 7.168 | -3.754 | 0.00E+00 | 1.00E-03 | 0.153 |
| CUL4A | -0.319 | 8.978 | -3.754 | 0.00E+00 | 1.00E-03 | 0.152 |
| HACE1 | -0.388 | 8.209 | -3.754 | 0.00E+00 | 1.00E-03 | 0.151 |
| ZZZ3 | -0.337 | 8.373 | -3.751 | 0.00E+00 | 1.00E-03 | 0.142 |
| SLC27A4 | -0.425 | 6.575 | -3.75 | 0.00E+00 | 1.00E-03 | 0.138 |
| CRLF2 | 0.439 | 5.361 | 3.747 | 0.00E+00 | 1.00E-03 | 0.132 |
| ZNF595 | -0.397 | 7.476 | -3.745 | 0.00E+00 | 1.00E-03 | 0.121 |
| SSNA1 | 0.366 | 9.656 | 3.743 | 0.00E+00 | 1.00E-03 | 0.12 |
| MRPS11 | 0.307 | 9.139 | 3.743 | 0.00E+00 | 1.00E-03 | 0.12 |
| TFPI2 | -0.737 | 11.408 | -3.744 | 0.00E+00 | 1.00E-03 | 0.118 |
| ATP6V1G1 | -0.409 | 11.508 | -3.743 | 0.00E+00 | 1.00E-03 | 0.117 |
| OSTM1 | -0.318 | 8.455 | -3.741 | 0.00E+00 | 1.00E-03 | 0.109 |
| CDCA7 | -0.407 | 7.63 | -3.741 | 0.00E+00 | 1.00E-03 | 0.109 |
| MICA | 0.44 | 8.178 | 3.738 | 0.00E+00 | 2.00E-03 | 0.101 |
| RPAP1 | 0.37 | 8.432 | 3.737 | 0.00E+00 | 2.00E-03 | 0.1 |
| COL4A1 | 0.527 | 11.271 | 3.737 | 0.00E+00 | 2.00E-03 | 0.098 |
| INPP5A | -0.418 | 7.433 | -3.737 | 0.00E+00 | 2.00E-03 | 0.097 |
| SS18L1 | -0.326 | 7.693 | -3.735 | 0.00E+00 | 2.00E-03 | 0.09 |
| TIMM17B | 0.294 | 9.322 | 3.734 | 0.00E+00 | 2.00E-03 | 0.089 |
| STARD5 | 0.395 | 7.101 | 3.733 | 0.00E+00 | 2.00E-03 | 0.085 |
| SP4 | -0.396 | 6.433 | -3.734 | 0.00E+00 | 2.00E-03 | 0.085 |
| FKBP8 | 0.47 | 9.109 | 3.733 | 0.00E+00 | 2.00E-03 | 0.084 |
| SLC22A18 | 0.423 | 8.179 | 3.731 | 0.00E+00 | 2.00E-03 | 0.08 |
| ZDHHC23 | -0.364 | 6.498 | -3.731 | 0.00E+00 | 2.00E-03 | 0.076 |
| H2AFY2 | 0.545 | 6.992 | 3.729 | 0.00E+00 | 2.00E-03 | 0.072 |
| ACIN1 | 0.366 | 9.341 | 3.728 | 0.00E+00 | 2.00E-03 | 0.067 |
| TYRO3 | 0.409 | 7.958 | 3.725 | 0.00E+00 | 2.00E-03 | 0.06 |
| NID2 | -0.593 | 7.704 | -3.726 | 0.00E+00 | 2.00E-03 | 0.057 |
| PRPH | 0.378 | 5.896 | 3.724 | 0.00E+00 | 2.00E-03 | 0.053 |
| RNF130 | -0.322 | 10.049 | -3.722 | 0.00E+00 | 2.00E-03 | 0.044 |
| TUFM | 0.347 | 10.669 | 3.719 | 0.00E+00 | 2.00E-03 | 0.039 |
| GJA4 | 0.571 | 8.078 | 3.716 | 0.00E+00 | 2.00E-03 | 0.028 |
| TMPRSS6 | 0.428 | 5.767 | 3.716 | 0.00E+00 | 2.00E-03 | 0.026 |
| EVI5L | 0.453 | 8.66 | 3.714 | 0.00E+00 | 2.00E-03 | 0.021 |
| MBD1 | -0.226 | 7.895 | -3.715 | 0.00E+00 | 2.00E-03 | 0.02 |
| NOXA1 | 0.462 | 7.177 | 3.713 | 0.00E+00 | 2.00E-03 | 0.019 |
| NUDT16L1 | 0.345 | 8.938 | 3.712 | 0.00E+00 | 2.00E-03 | 0.014 |
| GDAP1 | -0.427 | 5.537 | -3.713 | 0.00E+00 | 2.00E-03 | 0.013 |
| TRAM1 | -0.395 | 11.419 | -3.711 | 0.00E+00 | 2.00E-03 | 0.008 |
| PNKP | 0.396 | 8.914 | 3.707 | 0.00E+00 | 2.00E-03 | -0.001 |
| NSUN2 | -0.331 | 10.043 | -3.706 | 0.00E+00 | 2.00E-03 | -0.008 |
| PSG2 | 0.78 | 11.817 | 3.705 | 0.00E+00 | 2.00E-03 | -0.009 |
| MAP1LC3B | -0.329 | 10.99 | -3.706 | 0.00E+00 | 2.00E-03 | -0.011 |
| TGFB1I1 | 0.657 | 8.26 | 3.704 | 0.00E+00 | 2.00E-03 | -0.013 |
| VAC14 | 0.366 | 7.442 | 3.702 | 0.00E+00 | 2.00E-03 | -0.018 |
| NAGPA | 0.334 | 8.81 | 3.699 | 0.00E+00 | 2.00E-03 | -0.028 |
| ACTR2 | -0.395 | 10.537 | -3.698 | 0.00E+00 | 2.00E-03 | -0.037 |
| INSIG2 | -0.406 | 8.325 | -3.695 | 0.00E+00 | 2.00E-03 | -0.046 |
| ZNF185 | -0.419 | 9.337 | -3.691 | 0.00E+00 | 2.00E-03 | -0.059 |
| SRC | 0.399 | 7.053 | 3.69 | 0.00E+00 | 2.00E-03 | -0.06 |
| PLA2G5 | 0.446 | 8.059 | 3.69 | 0.00E+00 | 2.00E-03 | -0.061 |
| CYB561D2 | 0.313 | 8.977 | 3.685 | 0.00E+00 | 2.00E-03 | -0.076 |
| CIZ1 | 0.298 | 8.457 | 3.684 | 0.00E+00 | 2.00E-03 | -0.081 |
| PACSIN2 | -0.274 | 10.594 | -3.684 | 0.00E+00 | 2.00E-03 | -0.084 |
| CACNB4 | -0.467 | 5.139 | -3.683 | 0.00E+00 | 2.00E-03 | -0.087 |
| KRT16 | 0.427 | 6.372 | 3.68 | 0.00E+00 | 2.00E-03 | -0.092 |
| BNIP1 | 0.371 | 7.857 | 3.68 | 0.00E+00 | 2.00E-03 | -0.092 |
| GLI4 | 0.383 | 7.231 | 3.677 | 0.00E+00 | 2.00E-03 | -0.102 |
| PLEK | -0.38 | 8.673 | -3.676 | 0.00E+00 | 2.00E-03 | -0.109 |
| PCDH12 | 0.496 | 7.149 | 3.674 | 0.00E+00 | 2.00E-03 | -0.113 |
| LAMC3 | 0.559 | 9.296 | 3.671 | 0.00E+00 | 2.00E-03 | -0.122 |
| DEGS1 | -0.324 | 11.01 | -3.672 | 0.00E+00 | 2.00E-03 | -0.123 |
| LBR | -0.392 | 10.225 | -3.671 | 0.00E+00 | 2.00E-03 | -0.127 |
| PTGS2 | -0.761 | 6.502 | -3.667 | 0.00E+00 | 2.00E-03 | -0.14 |
| CHST10 | 0.454 | 6.945 | 3.659 | 0.00E+00 | 2.00E-03 | -0.162 |
| OXCT2 | 0.422 | 5.966 | 3.659 | 0.00E+00 | 2.00E-03 | -0.164 |
| ARNTL2 | -0.388 | 6.344 | -3.658 | 0.00E+00 | 2.00E-03 | -0.168 |
| ISLR | 0.685 | 8.037 | 3.657 | 0.00E+00 | 2.00E-03 | -0.169 |
| FBXO3 | -0.431 | 7.36 | -3.656 | 0.00E+00 | 2.00E-03 | -0.175 |
| RBM25 | -0.391 | 9.951 | -3.655 | 0.00E+00 | 2.00E-03 | -0.179 |
| HCFC1 | -0.395 | 8.48 | -3.653 | 0.00E+00 | 2.00E-03 | -0.185 |
| GRTP1 | -0.435 | 6.2 | -3.653 | 0.00E+00 | 2.00E-03 | -0.186 |
| INHBE | 0.584 | 6.091 | 3.651 | 0.00E+00 | 2.00E-03 | -0.188 |
| ZNF343 | 0.35 | 6.382 | 3.651 | 0.00E+00 | 2.00E-03 | -0.19 |
| C1orf35 | 0.278 | 9.578 | 3.648 | 0.00E+00 | 2.00E-03 | -0.2 |
| SF3B1 | -0.295 | 9.523 | -3.649 | 0.00E+00 | 2.00E-03 | -0.2 |
| KHDRBS1 | -0.278 | 10.86 | -3.646 | 0.00E+00 | 2.00E-03 | -0.208 |
| EPS8L2 | 0.329 | 8.098 | 3.644 | 0.00E+00 | 2.00E-03 | -0.213 |
| GLRX | -0.447 | 12.274 | -3.644 | 0.00E+00 | 2.00E-03 | -0.214 |
| BRD3 | 0.328 | 8.838 | 3.643 | 0.00E+00 | 2.00E-03 | -0.215 |
| AP3M2 | 0.313 | 8.355 | 3.642 | 0.00E+00 | 2.00E-03 | -0.22 |
| PLSCR4 | -0.544 | 8.549 | -3.642 | 0.00E+00 | 2.00E-03 | -0.223 |
| OLFML2A | 0.526 | 6.495 | 3.64 | 0.00E+00 | 2.00E-03 | -0.226 |
| RBBP8 | -0.39 | 7.926 | -3.641 | 0.00E+00 | 2.00E-03 | -0.226 |
| PPIB | 0.334 | 11.407 | 3.637 | 0.00E+00 | 2.00E-03 | -0.234 |
| OPN4 | 0.442 | 6.125 | 3.636 | 0.00E+00 | 2.00E-03 | -0.239 |
| TDRD3 | -0.311 | 6.553 | -3.637 | 0.00E+00 | 2.00E-03 | -0.24 |
| TNNC1 | 0.541 | 6.468 | 3.635 | 0.00E+00 | 2.00E-03 | -0.241 |
| MRPL15 | -0.324 | 10.559 | -3.636 | 0.00E+00 | 2.00E-03 | -0.241 |
| SNX16 | -0.461 | 6.677 | -3.634 | 0.00E+00 | 2.00E-03 | -0.249 |
| NPAS1 | 0.452 | 5.794 | 3.63 | 0.00E+00 | 2.00E-03 | -0.258 |
| PLCB1 | -0.345 | 7.535 | -3.629 | 0.00E+00 | 2.00E-03 | -0.265 |
| PKD2 | -0.394 | 8.588 | -3.628 | 0.00E+00 | 2.00E-03 | -0.269 |
| SEC24B | -0.323 | 8.543 | -3.627 | 0.00E+00 | 2.00E-03 | -0.27 |
| ADK | -0.324 | 8.944 | -3.627 | 0.00E+00 | 2.00E-03 | -0.273 |
| ABCC10 | 0.366 | 7.737 | 3.625 | 0.00E+00 | 2.00E-03 | -0.274 |
| VCL | -0.322 | 10.232 | -3.623 | 0.00E+00 | 2.00E-03 | -0.283 |
| STARD4 | -0.356 | 7.278 | -3.623 | 0.00E+00 | 2.00E-03 | -0.285 |
| SBNO1 | -0.335 | 8.148 | -3.623 | 0.00E+00 | 2.00E-03 | -0.286 |
| GPR62 | 0.448 | 5.759 | 3.62 | 0.00E+00 | 2.00E-03 | -0.291 |
| RBM11 | -0.411 | 5.209 | -3.619 | 0.00E+00 | 2.00E-03 | -0.297 |
| EIF2B5 | 0.277 | 8.938 | 3.618 | 0.00E+00 | 2.00E-03 | -0.297 |
| LYZL6 | 0.48 | 5.12 | 3.617 | 0.00E+00 | 2.00E-03 | -0.302 |
| RPL27A | -0.535 | 9.636 | -3.616 | 0.00E+00 | 2.00E-03 | -0.306 |
| RNF26 | 0.426 | 9.166 | 3.613 | 0.00E+00 | 2.00E-03 | -0.313 |
| NUDCD2 | -0.371 | 9.388 | -3.614 | 0.00E+00 | 2.00E-03 | -0.314 |
| GHITM | -0.271 | 10.682 | -3.614 | 0.00E+00 | 2.00E-03 | -0.315 |
| FGFRL1 | 0.448 | 6.999 | 3.612 | 0.00E+00 | 2.00E-03 | -0.317 |
| KCNN1 | 0.482 | 5.56 | 3.61 | 0.00E+00 | 2.00E-03 | -0.324 |
| GCH1 | -0.462 | 7.223 | -3.609 | 0.00E+00 | 2.00E-03 | -0.332 |
| HIRA | -0.392 | 8.401 | -3.607 | 0.00E+00 | 2.00E-03 | -0.338 |
| SCN4B | -0.758 | 6.25 | -3.602 | 0.00E+00 | 2.00E-03 | -0.353 |
| F7 | 0.433 | 5.882 | 3.6 | 0.00E+00 | 2.00E-03 | -0.357 |
| FXYD6 | 0.456 | 8.165 | 3.598 | 0.00E+00 | 2.00E-03 | -0.363 |
| PDK1 | -0.403 | 6.915 | -3.598 | 0.00E+00 | 2.00E-03 | -0.366 |
| LAMA4 | 0.398 | 8.079 | 3.597 | 0.00E+00 | 2.00E-03 | -0.367 |
| AMPD3 | -0.321 | 7.571 | -3.597 | 0.00E+00 | 2.00E-03 | -0.37 |
| VGLL1 | 0.361 | 8.515 | 3.595 | 0.00E+00 | 2.00E-03 | -0.372 |
| SLC4A1AP | -0.264 | 9.018 | -3.592 | 0.00E+00 | 3.00E-03 | -0.385 |
| SLC13A4 | 0.441 | 8.535 | 3.591 | 0.00E+00 | 3.00E-03 | -0.388 |
| FUT8 | -0.333 | 8.762 | -3.591 | 0.00E+00 | 3.00E-03 | -0.39 |
| WWP1 | -0.332 | 9.324 | -3.59 | 0.00E+00 | 3.00E-03 | -0.392 |
| GPR160 | -0.461 | 7.395 | -3.587 | 0.00E+00 | 3.00E-03 | -0.402 |
| TROAP | 0.441 | 7.757 | 3.585 | 0.00E+00 | 3.00E-03 | -0.405 |
| ALOXE3 | 0.556 | 6.466 | 3.585 | 0.00E+00 | 3.00E-03 | -0.407 |
| LEP | 1.355 | 8.071 | 3.583 | 0.00E+00 | 3.00E-03 | -0.411 |
| DPYS | -0.35 | 5.001 | -3.584 | 0.00E+00 | 3.00E-03 | -0.412 |
| TARDBP | -0.24 | 8.896 | -3.583 | 0.00E+00 | 3.00E-03 | -0.415 |
| SH2D2A | 0.429 | 6.863 | 3.582 | 0.00E+00 | 3.00E-03 | -0.416 |
| ACRC | -0.418 | 6.961 | -3.581 | 0.00E+00 | 3.00E-03 | -0.423 |
| LEPROTL1 | -0.43 | 10.409 | -3.58 | 0.00E+00 | 3.00E-03 | -0.424 |
| FARS2 | 0.278 | 8.495 | 3.579 | 0.00E+00 | 3.00E-03 | -0.427 |
| CDH11 | -0.512 | 8.32 | -3.577 | 0.00E+00 | 3.00E-03 | -0.436 |
| TM6SF2 | 0.427 | 6.341 | 3.574 | 0.00E+00 | 3.00E-03 | -0.44 |
| HAND1 | 0.543 | 5.928 | 3.572 | 0.00E+00 | 3.00E-03 | -0.447 |
| C11orf24 | 0.283 | 9.31 | 3.572 | 0.00E+00 | 3.00E-03 | -0.45 |
| ANGPTL1 | -0.368 | 6.716 | -3.572 | 0.00E+00 | 3.00E-03 | -0.451 |
| GPR50 | 0.408 | 5.501 | 3.569 | 0.00E+00 | 3.00E-03 | -0.458 |
| NR2C1 | -0.324 | 7.771 | -3.569 | 0.00E+00 | 3.00E-03 | -0.462 |
| FBXO11 | -0.314 | 7.802 | -3.568 | 0.00E+00 | 3.00E-03 | -0.465 |
| NT5C | 0.309 | 9.975 | 3.566 | 0.00E+00 | 3.00E-03 | -0.466 |
| TIMM50 | 0.255 | 8.447 | 3.565 | 0.00E+00 | 3.00E-03 | -0.47 |
| HECTD1 | -0.353 | 10.202 | -3.565 | 0.00E+00 | 3.00E-03 | -0.474 |
| ATP5F1 | -0.328 | 11.001 | -3.564 | 0.00E+00 | 3.00E-03 | -0.476 |
| VRK2 | -0.264 | 7.872 | -3.563 | 0.00E+00 | 3.00E-03 | -0.479 |
| HTATSF1 | -0.267 | 9.552 | -3.562 | 0.00E+00 | 3.00E-03 | -0.483 |
| P2RX2 | 0.466 | 7.476 | 3.56 | 0.00E+00 | 3.00E-03 | -0.487 |
| PPP5C | -0.394 | 6.833 | -3.561 | 0.00E+00 | 3.00E-03 | -0.487 |
| DLL4 | 0.418 | 6.012 | 3.559 | 0.00E+00 | 3.00E-03 | -0.489 |
| ABCE1 | -0.331 | 8.198 | -3.559 | 0.00E+00 | 3.00E-03 | -0.493 |
| HAMP | 0.432 | 7.16 | 3.556 | 0.00E+00 | 3.00E-03 | -0.499 |
| RABEP1 | -0.378 | 8.415 | -3.557 | 0.00E+00 | 3.00E-03 | -0.499 |
| WDR47 | -0.394 | 7.316 | -3.557 | 0.00E+00 | 3.00E-03 | -0.5 |
| RFXANK | 0.255 | 9.782 | 3.555 | 0.00E+00 | 3.00E-03 | -0.505 |
| MINK1 | 0.508 | 6.965 | 3.554 | 0.00E+00 | 3.00E-03 | -0.506 |
| NUP214 | 0.391 | 8.652 | 3.554 | 0.00E+00 | 3.00E-03 | -0.507 |
| MMP15 | 0.352 | 8.028 | 3.55 | 0.00E+00 | 3.00E-03 | -0.519 |
| ZNF429 | -0.44 | 8.588 | -3.551 | 0.00E+00 | 3.00E-03 | -0.521 |
| GPR12 | 0.476 | 6.285 | 3.549 | 0.00E+00 | 3.00E-03 | -0.524 |
| ALG3 | 0.356 | 9.442 | 3.549 | 0.00E+00 | 3.00E-03 | -0.524 |
| MTRR | -0.365 | 7.486 | -3.547 | 0.00E+00 | 3.00E-03 | -0.533 |
| TANK | -0.338 | 8.886 | -3.546 | 0.00E+00 | 3.00E-03 | -0.535 |
| ZNF222 | -0.389 | 6.673 | -3.544 | 0.00E+00 | 3.00E-03 | -0.542 |
| NET1 | -0.376 | 9.802 | -3.542 | 0.00E+00 | 3.00E-03 | -0.548 |
| C11orf30 | -0.246 | 7.808 | -3.542 | 0.00E+00 | 3.00E-03 | -0.549 |
| RBM22 | -0.352 | 10.293 | -3.541 | 0.00E+00 | 3.00E-03 | -0.551 |
| PHC3 | -0.414 | 7.456 | -3.541 | 0.00E+00 | 3.00E-03 | -0.552 |
| USP16 | -0.4 | 9.065 | -3.541 | 0.00E+00 | 3.00E-03 | -0.553 |
| MCM5 | 0.333 | 9.539 | 3.538 | 0.00E+00 | 3.00E-03 | -0.559 |
| HTR7 | -0.359 | 6.627 | -3.537 | 0.00E+00 | 3.00E-03 | -0.563 |
| GADD45G | -0.748 | 9.29 | -3.537 | 0.00E+00 | 3.00E-03 | -0.564 |
| DECR1 | -0.43 | 9.547 | -3.536 | 0.00E+00 | 3.00E-03 | -0.569 |
| NPR2 | 0.317 | 7.126 | 3.533 | 0.00E+00 | 3.00E-03 | -0.574 |
| SEC23B | -0.256 | 9.308 | -3.533 | 0.00E+00 | 3.00E-03 | -0.576 |
| ZNF350 | -0.306 | 8.322 | -3.533 | 0.00E+00 | 3.00E-03 | -0.578 |
| PPP1R13L | 0.456 | 9.006 | 3.529 | 0.00E+00 | 3.00E-03 | -0.588 |
| CARS | -0.321 | 8.473 | -3.528 | 0.00E+00 | 3.00E-03 | -0.592 |
| ST5 | 0.417 | 8.119 | 3.526 | 0.00E+00 | 3.00E-03 | -0.596 |
| SPTLC1 | -0.491 | 8.705 | -3.527 | 0.00E+00 | 3.00E-03 | -0.596 |
| CARD6 | -0.322 | 8.446 | -3.527 | 0.00E+00 | 3.00E-03 | -0.597 |
| TPM2 | 0.392 | 10.221 | 3.525 | 0.00E+00 | 3.00E-03 | -0.599 |
| SEC24C | -0.397 | 9.403 | -3.526 | 0.00E+00 | 3.00E-03 | -0.599 |
| L3MBTL3 | -0.337 | 7.391 | -3.526 | 0.00E+00 | 3.00E-03 | -0.6 |
| AGPAT4 | -0.319 | 6.901 | -3.525 | 0.00E+00 | 3.00E-03 | -0.604 |
| FMNL2 | -0.367 | 8.384 | -3.524 | 0.00E+00 | 3.00E-03 | -0.606 |
| ZNF165 | -0.381 | 7.727 | -3.52 | 0.00E+00 | 3.00E-03 | -0.62 |
| EIF5B | -0.345 | 8.376 | -3.518 | 0.00E+00 | 3.00E-03 | -0.626 |
| SNX8 | 0.417 | 8.47 | 3.516 | 0.00E+00 | 3.00E-03 | -0.629 |
| TTF1 | -0.323 | 7.523 | -3.517 | 0.00E+00 | 3.00E-03 | -0.63 |
| SOCS4 | -0.522 | 7.221 | -3.513 | 0.00E+00 | 3.00E-03 | -0.641 |
| STK16 | 0.282 | 7.391 | 3.511 | 0.00E+00 | 3.00E-03 | -0.644 |
| FAM49A | -0.306 | 9.131 | -3.511 | 0.00E+00 | 3.00E-03 | -0.649 |
| ITPKC | 0.326 | 8.871 | 3.508 | 0.00E+00 | 3.00E-03 | -0.655 |
| ANAPC11 | 0.227 | 10.611 | 3.507 | 0.00E+00 | 3.00E-03 | -0.658 |
| MRPL43 | 0.281 | 8.344 | 3.507 | 0.00E+00 | 3.00E-03 | -0.659 |
| SOX9 | 0.537 | 5.853 | 3.506 | 0.00E+00 | 3.00E-03 | -0.66 |
| TTC19 | -0.247 | 9.262 | -3.507 | 0.00E+00 | 3.00E-03 | -0.661 |
| HAPLN4 | 0.412 | 5.443 | 3.506 | 0.00E+00 | 3.00E-03 | -0.661 |
| MOGAT3 | 0.397 | 5.615 | 3.505 | 0.00E+00 | 3.00E-03 | -0.666 |
| RHOBTB3 | -0.643 | 9.884 | -3.504 | 0.00E+00 | 3.00E-03 | -0.671 |
| KRT1 | 0.665 | 7.949 | 3.502 | 0.00E+00 | 3.00E-03 | -0.673 |
| PSMA2 | -0.296 | 10.256 | -3.501 | 0.00E+00 | 3.00E-03 | -0.681 |
| FAM50A | 0.261 | 10.48 | 3.498 | 0.00E+00 | 3.00E-03 | -0.685 |
| SLC23A1 | -0.372 | 5.027 | -3.499 | 0.00E+00 | 3.00E-03 | -0.686 |
| CXCL2 | -0.789 | 6.898 | -3.498 | 0.00E+00 | 3.00E-03 | -0.69 |
| PRKAA1 | -0.24 | 7.813 | -3.497 | 0.00E+00 | 3.00E-03 | -0.693 |
| MPV17 | 0.274 | 9.53 | 3.495 | 0.00E+00 | 3.00E-03 | -0.696 |
| PCNXL2 | 0.277 | 6.159 | 3.495 | 0.00E+00 | 3.00E-03 | -0.697 |
| POLE4 | 0.329 | 10.373 | 3.494 | 0.00E+00 | 3.00E-03 | -0.701 |
| MRPL34 | 0.277 | 10.233 | 3.492 | 0.00E+00 | 3.00E-03 | -0.707 |
| PSAP | -0.341 | 12.429 | -3.492 | 0.00E+00 | 3.00E-03 | -0.708 |
| WBSCR22 | 0.35 | 8.989 | 3.49 | 0.00E+00 | 3.00E-03 | -0.711 |
| PPP1R10 | 0.258 | 9.238 | 3.49 | 0.00E+00 | 3.00E-03 | -0.713 |
| HGFAC | 0.382 | 6.948 | 3.488 | 0.00E+00 | 3.00E-03 | -0.717 |
| NR5A2 | 0.347 | 5.401 | 3.484 | 0.00E+00 | 4.00E-03 | -0.731 |
| RAB9A | -0.371 | 10.439 | -3.483 | 0.00E+00 | 4.00E-03 | -0.739 |
| NDEL1 | -0.317 | 9.946 | -3.482 | 0.00E+00 | 4.00E-03 | -0.741 |
| BRSK2 | 0.483 | 5.995 | 3.479 | 0.00E+00 | 4.00E-03 | -0.746 |
| PRKCDBP | 0.488 | 10.783 | 3.478 | 0.00E+00 | 4.00E-03 | -0.752 |
| WFDC9 | 0.434 | 5.407 | 3.476 | 0.00E+00 | 4.00E-03 | -0.757 |
| SLIT3 | 0.371 | 7.956 | 3.474 | 0.00E+00 | 4.00E-03 | -0.762 |
| C1D | -0.326 | 8.325 | -3.474 | 0.00E+00 | 4.00E-03 | -0.765 |
| SEZ6L2 | 0.364 | 7.078 | 3.471 | 0.00E+00 | 4.00E-03 | -0.771 |
| F11R | -0.525 | 8.963 | -3.47 | 0.00E+00 | 4.00E-03 | -0.78 |
| BET1L | 0.33 | 8.346 | 3.466 | 0.00E+00 | 4.00E-03 | -0.789 |
| ADAMTS19 | 0.391 | 6.283 | 3.466 | 0.00E+00 | 4.00E-03 | -0.789 |
| MAPKAPK3 | 0.314 | 9.643 | 3.463 | 0.00E+00 | 4.00E-03 | -0.798 |
| CHRM1 | 0.458 | 6.098 | 3.462 | 0.00E+00 | 4.00E-03 | -0.801 |
| SHMT2 | 0.324 | 9.872 | 3.461 | 0.00E+00 | 4.00E-03 | -0.804 |
| ZFP36L1 | -0.515 | 11.15 | -3.462 | 0.00E+00 | 4.00E-03 | -0.804 |
| NDUFS7 | 0.297 | 10.186 | 3.458 | 0.00E+00 | 4.00E-03 | -0.812 |
| MCOLN1 | 0.394 | 9.161 | 3.456 | 0.00E+00 | 4.00E-03 | -0.819 |
| DCPS | 0.3 | 8.93 | 3.453 | 0.00E+00 | 4.00E-03 | -0.829 |
| LLGL2 | 0.349 | 7.374 | 3.453 | 0.00E+00 | 4.00E-03 | -0.829 |
| ATP5I | 0.316 | 11.3 | 3.451 | 0.00E+00 | 4.00E-03 | -0.835 |
| SLC4A3 | 0.395 | 6.783 | 3.451 | 0.00E+00 | 4.00E-03 | -0.837 |
| PPP2R2C | 0.402 | 5.416 | 3.45 | 0.00E+00 | 4.00E-03 | -0.838 |
| ARMC1 | -0.298 | 8.98 | -3.451 | 0.00E+00 | 4.00E-03 | -0.838 |
| CTSC | -0.384 | 9.378 | -3.451 | 0.00E+00 | 4.00E-03 | -0.84 |
| CRYBB2 | 0.529 | 7.25 | 3.45 | 0.00E+00 | 4.00E-03 | -0.84 |
| TBC1D13 | 0.29 | 9.714 | 3.448 | 0.00E+00 | 4.00E-03 | -0.845 |
| RAD18 | -0.312 | 6.602 | -3.446 | 0.00E+00 | 4.00E-03 | -0.853 |
| HLCS | 0.318 | 7.412 | 3.444 | 0.00E+00 | 4.00E-03 | -0.857 |
| TMC5 | 0.429 | 7.072 | 3.443 | 0.00E+00 | 4.00E-03 | -0.86 |
| MGC50722 | 0.447 | 5.037 | 3.44 | 0.00E+00 | 4.00E-03 | -0.869 |
| ACADVL | 0.311 | 12.649 | 3.44 | 0.00E+00 | 4.00E-03 | -0.871 |
| IMPDH2 | 0.32 | 10.698 | 3.438 | 0.00E+00 | 4.00E-03 | -0.877 |
| RNASEH1 | -0.242 | 8.715 | -3.438 | 0.00E+00 | 4.00E-03 | -0.879 |
| FMO3 | 0.373 | 5.016 | 3.437 | 0.00E+00 | 4.00E-03 | -0.879 |
| PYY | 0.368 | 5.755 | 3.437 | 0.00E+00 | 4.00E-03 | -0.88 |
| WDR37 | -0.286 | 8.47 | -3.437 | 0.00E+00 | 4.00E-03 | -0.884 |
| CRKL | -0.394 | 8.52 | -3.432 | 0.00E+00 | 4.00E-03 | -0.898 |
| HDAC6 | 0.266 | 7.943 | 3.43 | 0.00E+00 | 4.00E-03 | -0.903 |
| IL17F | 0.484 | 6.038 | 3.429 | 0.00E+00 | 4.00E-03 | -0.905 |
| MTBP | -0.391 | 5.842 | -3.43 | 0.00E+00 | 4.00E-03 | -0.906 |
| GALK1 | 0.343 | 8.719 | 3.425 | 0.00E+00 | 4.00E-03 | -0.918 |
| ZNRD1 | 0.304 | 9.715 | 3.425 | 0.00E+00 | 4.00E-03 | -0.919 |
| PIK3CB | -0.448 | 7.657 | -3.425 | 0.00E+00 | 4.00E-03 | -0.92 |
| CAP1 | -0.269 | 11.938 | -3.424 | 0.00E+00 | 4.00E-03 | -0.925 |
| OAZ3 | 0.425 | 5.997 | 3.42 | 0.00E+00 | 4.00E-03 | -0.933 |
| HS2ST1 | -0.342 | 7.562 | -3.421 | 0.00E+00 | 4.00E-03 | -0.934 |
| OPA3 | 0.318 | 7.528 | 3.419 | 0.00E+00 | 4.00E-03 | -0.936 |
| ZNF133 | 0.239 | 8.815 | 3.418 | 0.00E+00 | 4.00E-03 | -0.94 |
| PDK2 | 0.508 | 7.902 | 3.414 | 0.00E+00 | 4.00E-03 | -0.952 |
| SFN | 0.587 | 6.972 | 3.414 | 0.00E+00 | 4.00E-03 | -0.953 |
| DDX3X | -0.383 | 9.309 | -3.414 | 0.00E+00 | 4.00E-03 | -0.953 |
| MGAT2 | -0.297 | 8.86 | -3.414 | 0.00E+00 | 4.00E-03 | -0.955 |
| ARMC7 | 0.322 | 8.889 | 3.412 | 0.00E+00 | 4.00E-03 | -0.957 |
| HOXC8 | 0.431 | 6.332 | 3.412 | 0.00E+00 | 4.00E-03 | -0.958 |
| RRH | 0.427 | 6.126 | 3.412 | 0.00E+00 | 4.00E-03 | -0.959 |
| MPST | 0.258 | 9.505 | 3.412 | 0.00E+00 | 4.00E-03 | -0.959 |
| POP5 | 0.265 | 8.824 | 3.409 | 0.00E+00 | 4.00E-03 | -0.969 |
| DNAJB9 | -0.379 | 10.921 | -3.409 | 0.00E+00 | 4.00E-03 | -0.97 |
| FXYD7 | 0.385 | 6.1 | 3.406 | 0.00E+00 | 5.00E-03 | -0.977 |
| RHEBL1 | 0.371 | 6.709 | 3.405 | 0.00E+00 | 5.00E-03 | -0.979 |
| MGAM | -0.37 | 5.937 | -3.405 | 0.00E+00 | 5.00E-03 | -0.982 |
| IQGAP3 | 0.45 | 7.574 | 3.401 | 0.00E+00 | 5.00E-03 | -0.992 |
| C9orf41 | -0.365 | 5.642 | -3.401 | 0.00E+00 | 5.00E-03 | -0.996 |
| NUDT2 | 0.326 | 8.857 | 3.399 | 0.00E+00 | 5.00E-03 | -1 |
| BIRC3 | -0.653 | 7.454 | -3.396 | 0.00E+00 | 5.00E-03 | -1.013 |
| SSBP4 | 0.276 | 8.936 | 3.391 | 0.00E+00 | 5.00E-03 | -1.025 |
| CTHRC1 | -0.566 | 8.882 | -3.389 | 0.00E+00 | 5.00E-03 | -1.032 |
| UBL5 | 0.289 | 11.866 | 3.388 | 0.00E+00 | 5.00E-03 | -1.034 |
| PYCRL | 0.384 | 6.427 | 3.386 | 0.00E+00 | 5.00E-03 | -1.039 |
| HSD17B8 | 0.304 | 8.239 | 3.383 | 0.00E+00 | 5.00E-03 | -1.048 |
| BCL2L2 | -0.258 | 9.53 | -3.383 | 0.00E+00 | 5.00E-03 | -1.053 |
| HSF1 | 0.202 | 9.751 | 3.38 | 0.00E+00 | 5.00E-03 | -1.056 |
| FOXA2 | 0.382 | 5.468 | 3.379 | 0.00E+00 | 5.00E-03 | -1.06 |
| FBXO4 | -0.329 | 6.731 | -3.378 | 0.00E+00 | 5.00E-03 | -1.069 |
| ZNF510 | -0.26 | 5.652 | -3.378 | 0.00E+00 | 5.00E-03 | -1.069 |
| MAF1 | 0.343 | 9.411 | 3.376 | 0.00E+00 | 5.00E-03 | -1.069 |
| MRPL17 | 0.281 | 9.73 | 3.373 | 0.00E+00 | 5.00E-03 | -1.08 |
| IDS | -0.255 | 8.879 | -3.374 | 0.00E+00 | 5.00E-03 | -1.08 |
| DNM3 | -0.381 | 5.877 | -3.372 | 0.00E+00 | 5.00E-03 | -1.087 |
| IFIH1 | -0.434 | 9.335 | -3.371 | 0.00E+00 | 5.00E-03 | -1.09 |
| TJP3 | 0.387 | 6.997 | 3.369 | 0.00E+00 | 5.00E-03 | -1.092 |
| ZMYND15 | 0.371 | 7.2 | 3.368 | 0.00E+00 | 5.00E-03 | -1.095 |
| CACNA2D1 | 0.319 | 5.078 | 3.367 | 0.00E+00 | 5.00E-03 | -1.097 |
| ZNF608 | -0.457 | 6.013 | -3.367 | 0.00E+00 | 5.00E-03 | -1.101 |
| GSN | 0.306 | 9.733 | 3.365 | 0.00E+00 | 5.00E-03 | -1.103 |
| MS4A1 | 0.348 | 6.061 | 3.364 | 0.00E+00 | 5.00E-03 | -1.108 |
| SULT2A1 | 0.358 | 4.884 | 3.363 | 0.00E+00 | 5.00E-03 | -1.112 |
| SLC9A3R1 | 0.324 | 10.421 | 3.362 | 0.00E+00 | 5.00E-03 | -1.114 |
| ITGB2 | -0.376 | 9.354 | -3.362 | 0.00E+00 | 5.00E-03 | -1.116 |
| ZNF10 | -0.299 | 7.013 | -3.362 | 0.00E+00 | 5.00E-03 | -1.116 |
| ENTPD5 | 0.428 | 6.013 | 3.359 | 0.00E+00 | 5.00E-03 | -1.121 |
| MID1IP1 | -0.375 | 8.619 | -3.36 | 0.00E+00 | 5.00E-03 | -1.124 |
| TLN2 | 0.388 | 6.852 | 3.357 | 0.00E+00 | 5.00E-03 | -1.13 |
| ASPH | -0.342 | 8.135 | -3.357 | 0.00E+00 | 5.00E-03 | -1.133 |
| HMGCL | 0.245 | 8.912 | 3.353 | 0.00E+00 | 5.00E-03 | -1.14 |
| SCT | 0.299 | 6.591 | 3.351 | 0.00E+00 | 5.00E-03 | -1.147 |
| EVL | 0.332 | 9.957 | 3.35 | 1.00E-03 | 5.00E-03 | -1.149 |
| TTC1 | 0.29 | 9.593 | 3.348 | 1.00E-03 | 5.00E-03 | -1.156 |
| LNX2 | -0.314 | 8.011 | -3.349 | 1.00E-03 | 5.00E-03 | -1.156 |
| HEBP2 | -0.434 | 11.181 | -3.347 | 1.00E-03 | 5.00E-03 | -1.164 |
| NR4A1 | 0.475 | 6.32 | 3.345 | 1.00E-03 | 5.00E-03 | -1.167 |
| CCNG2 | -0.458 | 7.718 | -3.345 | 1.00E-03 | 5.00E-03 | -1.17 |
| NRM | 0.271 | 8.401 | 3.344 | 1.00E-03 | 5.00E-03 | -1.17 |
| CNGB1 | 0.321 | 5.676 | 3.343 | 1.00E-03 | 5.00E-03 | -1.172 |
| AMACR | -0.483 | 6.572 | -3.342 | 1.00E-03 | 6.00E-03 | -1.18 |
| ANP32C | -0.625 | 6.394 | -3.341 | 1.00E-03 | 6.00E-03 | -1.181 |
| SULT1C2 | 0.4 | 5.326 | 3.34 | 1.00E-03 | 6.00E-03 | -1.182 |
| GPR25 | 0.483 | 6.933 | 3.339 | 1.00E-03 | 6.00E-03 | -1.186 |
| C6orf15 | 0.338 | 6.214 | 3.338 | 1.00E-03 | 6.00E-03 | -1.186 |
| APOA1BP | 0.265 | 9.99 | 3.336 | 1.00E-03 | 6.00E-03 | -1.193 |
| TRIM17 | -0.494 | 5.514 | -3.336 | 1.00E-03 | 6.00E-03 | -1.198 |
| HES5 | 0.388 | 6.873 | 3.333 | 1.00E-03 | 6.00E-03 | -1.202 |
| TRAF2 | 0.377 | 7.951 | 3.333 | 1.00E-03 | 6.00E-03 | -1.203 |
| ZNF304 | -0.334 | 6.765 | -3.333 | 1.00E-03 | 6.00E-03 | -1.206 |
| MRPL24 | 0.307 | 10.206 | 3.326 | 1.00E-03 | 6.00E-03 | -1.223 |
| GMEB2 | 0.402 | 8.412 | 3.326 | 1.00E-03 | 6.00E-03 | -1.223 |
| ZCCHC12 | -0.375 | 5.105 | -3.327 | 1.00E-03 | 6.00E-03 | -1.224 |
| BAZ2B | -0.393 | 8.532 | -3.327 | 1.00E-03 | 6.00E-03 | -1.225 |
| HMGN3 | -0.288 | 9.302 | -3.326 | 1.00E-03 | 6.00E-03 | -1.227 |
| G6PC2 | 0.355 | 5.561 | 3.319 | 1.00E-03 | 6.00E-03 | -1.247 |
| ADAMTS2 | 0.469 | 6.299 | 3.314 | 1.00E-03 | 6.00E-03 | -1.259 |
| KPNA6 | 0.349 | 9.771 | 3.313 | 1.00E-03 | 6.00E-03 | -1.262 |
| PPP2CB | -0.302 | 9.873 | -3.312 | 1.00E-03 | 6.00E-03 | -1.271 |
| PRDX5 | 0.242 | 12.312 | 3.311 | 1.00E-03 | 6.00E-03 | -1.271 |
| HCP5 | 0.473 | 7.921 | 3.31 | 1.00E-03 | 6.00E-03 | -1.271 |
| CCNJ | -0.289 | 7.335 | -3.311 | 1.00E-03 | 6.00E-03 | -1.274 |
| POLDIP2 | 0.201 | 9.469 | 3.308 | 1.00E-03 | 6.00E-03 | -1.278 |
| SOX21 | 0.349 | 5.55 | 3.306 | 1.00E-03 | 6.00E-03 | -1.284 |
| IL10RB | -0.336 | 10.091 | -3.307 | 1.00E-03 | 6.00E-03 | -1.284 |
| PAFAH1B2 | -0.284 | 8.186 | -3.306 | 1.00E-03 | 6.00E-03 | -1.287 |
| SCO2 | 0.318 | 9.348 | 3.303 | 1.00E-03 | 6.00E-03 | -1.293 |
| HLF | -0.563 | 6.214 | -3.303 | 1.00E-03 | 6.00E-03 | -1.296 |
| ULK1 | -0.311 | 9.918 | -3.303 | 1.00E-03 | 6.00E-03 | -1.296 |
| CKMT2 | 0.43 | 5.07 | 3.3 | 1.00E-03 | 6.00E-03 | -1.304 |
| STK11 | 0.316 | 8.47 | 3.298 | 1.00E-03 | 6.00E-03 | -1.308 |
| PCDHB4 | 0.449 | 5.809 | 3.298 | 1.00E-03 | 6.00E-03 | -1.31 |
| APOE | 0.574 | 10.215 | 3.297 | 1.00E-03 | 6.00E-03 | -1.312 |
| SEPHS1 | -0.228 | 8.824 | -3.298 | 1.00E-03 | 6.00E-03 | -1.313 |
| PEX11G | 0.432 | 6.344 | 3.296 | 1.00E-03 | 6.00E-03 | -1.314 |
| VAT1 | 0.298 | 9.488 | 3.296 | 1.00E-03 | 6.00E-03 | -1.315 |
| OXR1 | -0.374 | 7.793 | -3.297 | 1.00E-03 | 6.00E-03 | -1.316 |
| ST8SIA2 | 0.353 | 5.592 | 3.295 | 1.00E-03 | 6.00E-03 | -1.319 |
| POLDIP3 | 0.235 | 9.416 | 3.295 | 1.00E-03 | 6.00E-03 | -1.319 |
| HSPB2 | 0.367 | 7.32 | 3.294 | 1.00E-03 | 6.00E-03 | -1.321 |
| ITCH | -0.237 | 8.135 | -3.294 | 1.00E-03 | 6.00E-03 | -1.324 |
| EPHB4 | -0.429 | 8.279 | -3.293 | 1.00E-03 | 6.00E-03 | -1.328 |
| TCF12 | -0.261 | 8.343 | -3.293 | 1.00E-03 | 6.00E-03 | -1.328 |
| TGDS | -0.288 | 8.543 | -3.293 | 1.00E-03 | 6.00E-03 | -1.329 |
| DSC3 | 0.308 | 6.557 | 3.291 | 1.00E-03 | 6.00E-03 | -1.33 |
| SLC10A1 | 0.368 | 5.334 | 3.291 | 1.00E-03 | 6.00E-03 | -1.33 |
| MRPL44 | -0.337 | 8.659 | -3.292 | 1.00E-03 | 6.00E-03 | -1.331 |
| ARFGAP3 | -0.335 | 10.003 | -3.292 | 1.00E-03 | 6.00E-03 | -1.331 |
| UBE2H | -0.369 | 9.31 | -3.291 | 1.00E-03 | 6.00E-03 | -1.333 |
| UNG | -0.277 | 8.596 | -3.291 | 1.00E-03 | 6.00E-03 | -1.335 |
| TARSL2 | -0.382 | 6.371 | -3.29 | 1.00E-03 | 6.00E-03 | -1.335 |
| REL | -0.27 | 7.402 | -3.29 | 1.00E-03 | 6.00E-03 | -1.336 |
| XPNPEP2 | 0.398 | 6.006 | 3.289 | 1.00E-03 | 6.00E-03 | -1.337 |
| PSMA3 | -0.282 | 11.241 | -3.29 | 1.00E-03 | 6.00E-03 | -1.338 |
| CAD | 0.311 | 8.257 | 3.288 | 1.00E-03 | 6.00E-03 | -1.339 |
| INCENP | -0.473 | 6.273 | -3.286 | 1.00E-03 | 6.00E-03 | -1.348 |
| HIST1H4A | 0.424 | 5.843 | 3.282 | 1.00E-03 | 7.00E-03 | -1.359 |
| PCSK1N | 0.732 | 8.42 | 3.279 | 1.00E-03 | 7.00E-03 | -1.368 |
| SPAG9 | -0.468 | 7.886 | -3.278 | 1.00E-03 | 7.00E-03 | -1.372 |
| PCSK4 | 0.356 | 6.371 | 3.276 | 1.00E-03 | 7.00E-03 | -1.377 |
| NOX3 | 0.335 | 4.598 | 3.275 | 1.00E-03 | 7.00E-03 | -1.379 |
| PES1 | 0.283 | 7.613 | 3.275 | 1.00E-03 | 7.00E-03 | -1.38 |
| RANBP10 | 0.352 | 8.698 | 3.273 | 1.00E-03 | 7.00E-03 | -1.384 |
| UTRN | -0.334 | 7.948 | -3.273 | 1.00E-03 | 7.00E-03 | -1.388 |
| ZNF511 | 0.304 | 8.729 | 3.269 | 1.00E-03 | 7.00E-03 | -1.396 |
| B4GALT7 | 0.379 | 8.567 | 3.269 | 1.00E-03 | 7.00E-03 | -1.396 |
| PPOX | 0.276 | 8.89 | 3.269 | 1.00E-03 | 7.00E-03 | -1.397 |
| AMMECR1 | -0.418 | 7.414 | -3.269 | 1.00E-03 | 7.00E-03 | -1.401 |
| SNCB | 0.474 | 6.889 | 3.268 | 1.00E-03 | 7.00E-03 | -1.401 |
| CRIP1 | 0.399 | 10.597 | 3.266 | 1.00E-03 | 7.00E-03 | -1.405 |
| GTF2A1 | -0.392 | 6.666 | -3.267 | 1.00E-03 | 7.00E-03 | -1.407 |
| PAPPA | -0.69 | 10.622 | -3.266 | 1.00E-03 | 7.00E-03 | -1.409 |
| PTOV1 | 0.296 | 10.514 | 3.259 | 1.00E-03 | 7.00E-03 | -1.426 |
| TRIM41 | 0.248 | 8.476 | 3.259 | 1.00E-03 | 7.00E-03 | -1.428 |
| MPDZ | 0.279 | 8.182 | 3.257 | 1.00E-03 | 7.00E-03 | -1.433 |
| YWHAB | -0.22 | 12.13 | -3.258 | 1.00E-03 | 7.00E-03 | -1.434 |
| SCP2 | -0.341 | 8.202 | -3.252 | 1.00E-03 | 7.00E-03 | -1.45 |
| GIMAP2 | -0.338 | 8.333 | -3.252 | 1.00E-03 | 7.00E-03 | -1.452 |
| MCM3AP | 0.233 | 9.429 | 3.25 | 1.00E-03 | 7.00E-03 | -1.453 |
| KRAS | -0.232 | 7.549 | -3.25 | 1.00E-03 | 7.00E-03 | -1.456 |
| TWSG1 | -0.448 | 7.812 | -3.25 | 1.00E-03 | 7.00E-03 | -1.458 |
| ITM2C | 0.37 | 10.046 | 3.248 | 1.00E-03 | 7.00E-03 | -1.46 |
| CTCF | -0.209 | 9.288 | -3.249 | 1.00E-03 | 7.00E-03 | -1.461 |
| ADAM9 | -0.339 | 7.757 | -3.247 | 1.00E-03 | 7.00E-03 | -1.466 |
| KRT23 | -0.362 | 10.36 | -3.246 | 1.00E-03 | 7.00E-03 | -1.468 |
| BFSP1 | 0.404 | 6.681 | 3.245 | 1.00E-03 | 7.00E-03 | -1.469 |
| OR8B8 | 0.392 | 5.734 | 3.244 | 1.00E-03 | 7.00E-03 | -1.472 |
| AICDA | -0.514 | 6.507 | -3.244 | 1.00E-03 | 7.00E-03 | -1.475 |
| ADRB3 | 0.46 | 6.799 | 3.24 | 1.00E-03 | 7.00E-03 | -1.483 |
| FAM19A2 | -0.419 | 5.64 | -3.241 | 1.00E-03 | 7.00E-03 | -1.484 |
| UAP1L1 | 0.354 | 7.477 | 3.239 | 1.00E-03 | 7.00E-03 | -1.486 |
| B4GALT6 | -0.312 | 5.386 | -3.24 | 1.00E-03 | 7.00E-03 | -1.488 |
| UQCRC2 | -0.39 | 10.342 | -3.238 | 1.00E-03 | 7.00E-03 | -1.492 |
| RPL8 | 0.252 | 13.059 | 3.236 | 1.00E-03 | 7.00E-03 | -1.495 |
| PSMC5 | -0.318 | 10.835 | -3.237 | 1.00E-03 | 7.00E-03 | -1.497 |
| LMNB1 | -0.402 | 8.183 | -3.236 | 1.00E-03 | 7.00E-03 | -1.498 |
| SLC23A2 | -0.388 | 7.619 | -3.236 | 1.00E-03 | 7.00E-03 | -1.499 |
| RNF168 | -0.358 | 6.226 | -3.235 | 1.00E-03 | 7.00E-03 | -1.502 |
| EXOSC5 | 0.298 | 7.635 | 3.233 | 1.00E-03 | 7.00E-03 | -1.505 |
| FIGNL1 | -0.405 | 6.913 | -3.233 | 1.00E-03 | 7.00E-03 | -1.508 |
| P2RY14 | -0.558 | 8.145 | -3.23 | 1.00E-03 | 8.00E-03 | -1.517 |
| NDUFA9 | 0.239 | 9.931 | 3.229 | 1.00E-03 | 8.00E-03 | -1.518 |
| TAF5 | -0.375 | 7.33 | -3.23 | 1.00E-03 | 8.00E-03 | -1.518 |
| LSM1 | -0.331 | 10.457 | -3.23 | 1.00E-03 | 8.00E-03 | -1.518 |
| CA4 | 0.554 | 7.562 | 3.227 | 1.00E-03 | 8.00E-03 | -1.521 |
| HOXA10 | -0.392 | 7.434 | -3.227 | 1.00E-03 | 8.00E-03 | -1.525 |
| GTPBP1 | 0.248 | 7.82 | 3.224 | 1.00E-03 | 8.00E-03 | -1.531 |
| F13A1 | -0.463 | 10.551 | -3.224 | 1.00E-03 | 8.00E-03 | -1.536 |
| NUDCD3 | 0.252 | 8.061 | 3.22 | 1.00E-03 | 8.00E-03 | -1.542 |
| RAB40B | -0.344 | 7.497 | -3.221 | 1.00E-03 | 8.00E-03 | -1.543 |
| CD248 | 0.525 | 10.176 | 3.219 | 1.00E-03 | 8.00E-03 | -1.545 |
| BCCIP | -0.272 | 8.04 | -3.22 | 1.00E-03 | 8.00E-03 | -1.548 |
| PPP1R12B | -0.29 | 7.509 | -3.218 | 1.00E-03 | 8.00E-03 | -1.552 |
| LILRA2 | -0.385 | 6.554 | -3.218 | 1.00E-03 | 8.00E-03 | -1.553 |
| NCOA3 | -0.291 | 9.347 | -3.217 | 1.00E-03 | 8.00E-03 | -1.556 |
| TOP1MT | 0.271 | 8.443 | 3.214 | 1.00E-03 | 8.00E-03 | -1.56 |
| FKBP14 | -0.31 | 7.942 | -3.215 | 1.00E-03 | 8.00E-03 | -1.561 |
| BCKDK | 0.256 | 10.186 | 3.213 | 1.00E-03 | 8.00E-03 | -1.563 |
| HK1 | 0.285 | 8.75 | 3.213 | 1.00E-03 | 8.00E-03 | -1.563 |
| NCOA1 | -0.256 | 8.521 | -3.214 | 1.00E-03 | 8.00E-03 | -1.564 |
| BRD4 | 0.304 | 7.51 | 3.212 | 1.00E-03 | 8.00E-03 | -1.566 |
| NPC2 | -0.312 | 12.756 | -3.212 | 1.00E-03 | 8.00E-03 | -1.57 |
| PCDHB1 | 0.409 | 5.223 | 3.211 | 1.00E-03 | 8.00E-03 | -1.571 |
| XRCC4 | -0.307 | 7.285 | -3.211 | 1.00E-03 | 8.00E-03 | -1.573 |
| QTRTD1 | -0.271 | 8.243 | -3.211 | 1.00E-03 | 8.00E-03 | -1.574 |
| DCTN1 | 0.301 | 8.259 | 3.207 | 1.00E-03 | 8.00E-03 | -1.583 |
| CNIH2 | 0.345 | 6.342 | 3.205 | 1.00E-03 | 8.00E-03 | -1.587 |
| CHRNA3 | 0.234 | 4.929 | 3.205 | 1.00E-03 | 8.00E-03 | -1.587 |
| UNC5B | 0.578 | 7.038 | 3.205 | 1.00E-03 | 8.00E-03 | -1.588 |
| ARRDC3 | -0.499 | 8.626 | -3.205 | 1.00E-03 | 8.00E-03 | -1.59 |
| CGN | 0.404 | 6.607 | 3.202 | 1.00E-03 | 8.00E-03 | -1.596 |
| CSNK1G3 | -0.338 | 7.739 | -3.203 | 1.00E-03 | 8.00E-03 | -1.596 |
| CYP2S1 | 0.421 | 6.362 | 3.202 | 1.00E-03 | 8.00E-03 | -1.596 |
| MAPK12 | 0.351 | 6.896 | 3.202 | 1.00E-03 | 8.00E-03 | -1.597 |
| RPS10 | 0.278 | 13.667 | 3.197 | 1.00E-03 | 8.00E-03 | -1.612 |
| EPB41L4A | 0.288 | 6.225 | 3.196 | 1.00E-03 | 8.00E-03 | -1.614 |
| SCAMP4 | 0.342 | 7.72 | 3.189 | 1.00E-03 | 8.00E-03 | -1.636 |
| BRAF | 0.315 | 7.339 | 3.188 | 1.00E-03 | 8.00E-03 | -1.638 |
| H2AFV | -0.224 | 8.953 | -3.188 | 1.00E-03 | 8.00E-03 | -1.641 |
| NINJ2 | 0.482 | 7.373 | 3.184 | 1.00E-03 | 9.00E-03 | -1.65 |
| OAS2 | 0.47 | 8.092 | 3.184 | 1.00E-03 | 9.00E-03 | -1.651 |
| PPHLN1 | -0.212 | 8.068 | -3.185 | 1.00E-03 | 9.00E-03 | -1.651 |
| FOSB | 0.589 | 8.154 | 3.183 | 1.00E-03 | 9.00E-03 | -1.654 |
| ITGB5 | 0.365 | 9.431 | 3.181 | 1.00E-03 | 9.00E-03 | -1.658 |
| GALNS | -0.392 | 7.473 | -3.181 | 1.00E-03 | 9.00E-03 | -1.66 |
| MPP1 | 0.292 | 10.862 | 3.172 | 1.00E-03 | 9.00E-03 | -1.685 |
| HAX1 | 0.204 | 10.002 | 3.171 | 1.00E-03 | 9.00E-03 | -1.687 |
| ZDHHC19 | 0.364 | 5.531 | 3.171 | 1.00E-03 | 9.00E-03 | -1.688 |
| CENPJ | -0.288 | 7.609 | -3.171 | 1.00E-03 | 9.00E-03 | -1.692 |
| GLA | 0.255 | 10.594 | 3.168 | 1.00E-03 | 9.00E-03 | -1.696 |
| F9 | 0.321 | 4.678 | 3.168 | 1.00E-03 | 9.00E-03 | -1.696 |
| CKB | 0.431 | 8.292 | 3.168 | 1.00E-03 | 9.00E-03 | -1.697 |
| PROK1 | 0.615 | 6.321 | 3.167 | 1.00E-03 | 9.00E-03 | -1.7 |
| JUN | 0.603 | 9.301 | 3.167 | 1.00E-03 | 9.00E-03 | -1.7 |
| EMD | 0.448 | 9.312 | 3.164 | 1.00E-03 | 9.00E-03 | -1.709 |
| RAB1A | -0.32 | 10.474 | -3.164 | 1.00E-03 | 9.00E-03 | -1.711 |
| COMMD1 | 0.302 | 10.143 | 3.162 | 1.00E-03 | 9.00E-03 | -1.713 |
| TRIP10 | 0.388 | 8.921 | 3.162 | 1.00E-03 | 9.00E-03 | -1.715 |
| POLR2H | 0.217 | 10.533 | 3.162 | 1.00E-03 | 9.00E-03 | -1.715 |
| P4HA1 | -0.346 | 8.496 | -3.162 | 1.00E-03 | 9.00E-03 | -1.718 |
| AP1S2 | -0.344 | 8.801 | -3.161 | 1.00E-03 | 9.00E-03 | -1.721 |
| GPHN | 0.351 | 6.983 | 3.159 | 1.00E-03 | 9.00E-03 | -1.722 |
| PTP4A3 | 0.311 | 7.586 | 3.159 | 1.00E-03 | 9.00E-03 | -1.722 |
| HDAC4 | -0.281 | 7.828 | -3.159 | 1.00E-03 | 9.00E-03 | -1.727 |
| BSG | 0.384 | 10.548 | 3.157 | 1.00E-03 | 9.00E-03 | -1.73 |
| ADD1 | 0.245 | 9.5 | 3.156 | 1.00E-03 | 9.00E-03 | -1.731 |
| SNRPC | -0.298 | 9.162 | -3.157 | 1.00E-03 | 9.00E-03 | -1.732 |
| RNF11 | -0.396 | 10.632 | -3.156 | 1.00E-03 | 9.00E-03 | -1.734 |
| LRP12 | -0.39 | 6.444 | -3.155 | 1.00E-03 | 9.00E-03 | -1.737 |
| PIK3R4 | -0.318 | 8.251 | -3.155 | 1.00E-03 | 9.00E-03 | -1.739 |
| ZNF205 | 0.552 | 7.98 | 3.152 | 1.00E-03 | 9.00E-03 | -1.744 |
| C10orf62 | 0.435 | 5.076 | 3.15 | 1.00E-03 | 9.00E-03 | -1.75 |
| WDR45 | 0.29 | 9.585 | 3.149 | 1.00E-03 | 9.00E-03 | -1.752 |
| SPESP1 | -0.651 | 7.03 | -3.145 | 1.00E-03 | 1.00E-02 | -1.768 |
| STK38 | -0.379 | 9.07 | -3.144 | 1.00E-03 | 1.00E-02 | -1.769 |
| RHEB | -0.287 | 9.931 | -3.143 | 1.00E-03 | 1.00E-02 | -1.772 |
| NDUFA8 | 0.241 | 11.665 | 3.141 | 1.00E-03 | 1.00E-02 | -1.775 |
| RNF111 | -0.22 | 8.534 | -3.141 | 2.00E-03 | 1.00E-02 | -1.78 |
| GPX4 | 0.246 | 11.861 | 3.138 | 2.00E-03 | 1.00E-02 | -1.785 |
| SRM | 0.294 | 9.085 | 3.138 | 2.00E-03 | 1.00E-02 | -1.786 |
| FANCD2 | -0.41 | 6.156 | -3.138 | 2.00E-03 | 1.00E-02 | -1.786 |
| LCN1 | 0.366 | 5.648 | 3.137 | 2.00E-03 | 1.00E-02 | -1.788 |
| YAP1 | -0.471 | 8.791 | -3.137 | 2.00E-03 | 1.00E-02 | -1.791 |
| THRA | 0.308 | 7.651 | 3.135 | 2.00E-03 | 1.00E-02 | -1.793 |
| POLR3D | 0.249 | 8.444 | 3.131 | 2.00E-03 | 1.00E-02 | -1.806 |
| GTF2F2 | -0.252 | 9.765 | -3.13 | 2.00E-03 | 1.00E-02 | -1.81 |
| RHOB | -0.634 | 9.149 | -3.13 | 2.00E-03 | 1.00E-02 | -1.811 |
| SERP1 | -0.304 | 9.64 | -3.129 | 2.00E-03 | 1.00E-02 | -1.814 |
| ZDHHC17 | -0.252 | 8.208 | -3.128 | 2.00E-03 | 1.00E-02 | -1.815 |
| DPF3 | 0.297 | 5.866 | 3.124 | 2.00E-03 | 1.00E-02 | -1.824 |
| PLXNA3 | 0.417 | 7.939 | 3.124 | 2.00E-03 | 1.00E-02 | -1.826 |
| FKBP10 | 0.429 | 8.153 | 3.122 | 2.00E-03 | 1.00E-02 | -1.831 |
| SNX27 | -0.254 | 9.79 | -3.123 | 2.00E-03 | 1.00E-02 | -1.831 |
| MKLN1 | -0.259 | 9.143 | -3.122 | 2.00E-03 | 1.00E-02 | -1.832 |
| RPUSD2 | 0.216 | 9.337 | 3.118 | 2.00E-03 | 1.00E-02 | -1.843 |
| TBP | -0.202 | 8.817 | -3.118 | 2.00E-03 | 1.00E-02 | -1.845 |
| FOXC1 | 0.386 | 8.735 | 3.117 | 2.00E-03 | 1.00E-02 | -1.846 |
| CLPX | -0.222 | 9.192 | -3.116 | 2.00E-03 | 1.00E-02 | -1.852 |
| GFAP | 0.309 | 5.283 | 3.113 | 2.00E-03 | 1.00E-02 | -1.857 |
| ZNF24 | -0.231 | 9.157 | -3.113 | 2.00E-03 | 1.00E-02 | -1.858 |
| ZC3HC1 | 0.233 | 9.304 | 3.111 | 2.00E-03 | 1.00E-02 | -1.862 |
| CHIC2 | -0.284 | 9.337 | -3.11 | 2.00E-03 | 1.10E-02 | -1.87 |
| BAX | 0.269 | 8.5 | 3.108 | 2.00E-03 | 1.10E-02 | -1.871 |
| MAN2B2 | -0.414 | 8.408 | -3.104 | 2.00E-03 | 1.10E-02 | -1.885 |
| CNOT10 | 0.254 | 8.761 | 3.103 | 2.00E-03 | 1.10E-02 | -1.887 |
| CYP1B1 | -0.629 | 8.293 | -3.101 | 2.00E-03 | 1.10E-02 | -1.893 |
| ZNF576 | 0.241 | 7.385 | 3.099 | 2.00E-03 | 1.10E-02 | -1.897 |
| LZTS2 | 0.283 | 9.083 | 3.096 | 2.00E-03 | 1.10E-02 | -1.905 |
| GALM | 0.317 | 7.574 | 3.094 | 2.00E-03 | 1.10E-02 | -1.912 |
| DAZL | -0.448 | 4.999 | -3.093 | 2.00E-03 | 1.10E-02 | -1.918 |
| KLF16 | 0.353 | 7.807 | 3.091 | 2.00E-03 | 1.10E-02 | -1.921 |
| PAPPA2 | 0.567 | 7.882 | 3.09 | 2.00E-03 | 1.10E-02 | -1.923 |
| LPPR2 | 0.29 | 7.623 | 3.089 | 2.00E-03 | 1.10E-02 | -1.926 |
| LRRN1 | -0.456 | 5.51 | -3.089 | 2.00E-03 | 1.10E-02 | -1.929 |
| KRT20 | 0.414 | 5.275 | 3.087 | 2.00E-03 | 1.10E-02 | -1.932 |
| COMTD1 | 0.346 | 9.248 | 3.086 | 2.00E-03 | 1.10E-02 | -1.934 |
| MAP3K5 | -0.395 | 8.53 | -3.087 | 2.00E-03 | 1.10E-02 | -1.935 |
| COPS6 | 0.179 | 10.786 | 3.085 | 2.00E-03 | 1.10E-02 | -1.937 |
| CACNA1F | 0.339 | 5.765 | 3.085 | 2.00E-03 | 1.10E-02 | -1.937 |
| PDCD2 | -0.217 | 8.992 | -3.085 | 2.00E-03 | 1.10E-02 | -1.94 |
| DDX27 | -0.204 | 9.833 | -3.083 | 2.00E-03 | 1.10E-02 | -1.946 |
| ARV1 | -0.204 | 8.339 | -3.081 | 2.00E-03 | 1.10E-02 | -1.951 |
| STOM | -0.543 | 10.483 | -3.08 | 2.00E-03 | 1.10E-02 | -1.953 |
| MB | 0.401 | 5.021 | 3.079 | 2.00E-03 | 1.10E-02 | -1.955 |
| GRINA | 0.506 | 10.124 | 3.078 | 2.00E-03 | 1.10E-02 | -1.956 |
| NDUFV3 | 0.243 | 9.028 | 3.076 | 2.00E-03 | 1.20E-02 | -1.962 |
| MRPL52 | 0.27 | 9.01 | 3.075 | 2.00E-03 | 1.20E-02 | -1.965 |
| HARS2 | -0.232 | 8.787 | -3.076 | 2.00E-03 | 1.20E-02 | -1.966 |
| GTF2H5 | 0.283 | 9.541 | 3.075 | 2.00E-03 | 1.20E-02 | -1.966 |
| SMYD5 | 0.261 | 7.62 | 3.075 | 2.00E-03 | 1.20E-02 | -1.966 |
| APEH | 0.248 | 9.969 | 3.075 | 2.00E-03 | 1.20E-02 | -1.966 |
| KIDINS220 | -0.269 | 9.326 | -3.076 | 2.00E-03 | 1.20E-02 | -1.967 |
| PKN3 | 0.397 | 6.933 | 3.074 | 2.00E-03 | 1.20E-02 | -1.969 |
| PYGO2 | 0.259 | 8.659 | 3.074 | 2.00E-03 | 1.20E-02 | -1.97 |
| TBL1X | 0.291 | 9.814 | 3.071 | 2.00E-03 | 1.20E-02 | -1.976 |
| SNRPD1 | -0.254 | 8.987 | -3.072 | 2.00E-03 | 1.20E-02 | -1.978 |
| SLC25A27 | -0.345 | 5.583 | -3.072 | 2.00E-03 | 1.20E-02 | -1.978 |
| BTBD3 | -0.351 | 8.757 | -3.069 | 2.00E-03 | 1.20E-02 | -1.987 |
| CAP2 | -0.526 | 8.387 | -3.069 | 2.00E-03 | 1.20E-02 | -1.987 |
| EGF | -0.423 | 5.016 | -3.068 | 2.00E-03 | 1.20E-02 | -1.987 |
| LRCH3 | 0.256 | 7.107 | 3.066 | 2.00E-03 | 1.20E-02 | -1.991 |
| HMGCR | -0.373 | 9.41 | -3.066 | 2.00E-03 | 1.20E-02 | -1.993 |
| WNT10B | 0.482 | 6.119 | 3.065 | 2.00E-03 | 1.20E-02 | -1.995 |
| BHMT2 | -0.453 | 5.195 | -3.065 | 2.00E-03 | 1.20E-02 | -1.998 |
| MAGEH1 | -0.322 | 8.968 | -3.064 | 2.00E-03 | 1.20E-02 | -1.999 |
| BCAN | 0.31 | 5.578 | 3.063 | 2.00E-03 | 1.20E-02 | -2.001 |
| KIF3A | -0.28 | 7.453 | -3.062 | 2.00E-03 | 1.20E-02 | -2.006 |
| WFDC3 | 0.4 | 7.084 | 3.059 | 2.00E-03 | 1.20E-02 | -2.011 |
| NAT2 | -0.38 | 6.207 | -3.058 | 2.00E-03 | 1.20E-02 | -2.017 |
| DNAJB2 | 0.242 | 9.533 | 3.057 | 2.00E-03 | 1.20E-02 | -2.018 |
| EEF1E1 | -0.348 | 8.543 | -3.056 | 2.00E-03 | 1.20E-02 | -2.021 |
| SDSL | 0.462 | 8.572 | 3.054 | 2.00E-03 | 1.20E-02 | -2.025 |
| TSTA3 | 0.321 | 9.387 | 3.054 | 2.00E-03 | 1.20E-02 | -2.025 |
| SLITRK2 | 0.246 | 4.972 | 3.053 | 2.00E-03 | 1.20E-02 | -2.028 |
| TMEM39A | -0.239 | 8.584 | -3.054 | 2.00E-03 | 1.20E-02 | -2.03 |
| SLC35D1 | -0.339 | 6.764 | -3.052 | 2.00E-03 | 1.20E-02 | -2.034 |
| WFDC8 | 0.316 | 5.064 | 3.05 | 2.00E-03 | 1.20E-02 | -2.038 |
| TRIM36 | -0.346 | 5.359 | -3.05 | 2.00E-03 | 1.20E-02 | -2.041 |
| CREB3L1 | 0.4 | 8.17 | 3.048 | 2.00E-03 | 1.20E-02 | -2.041 |
| PAX2 | 0.225 | 5.51 | 3.048 | 2.00E-03 | 1.20E-02 | -2.042 |
| PACSIN3 | 0.466 | 8.453 | 3.044 | 2.00E-03 | 1.20E-02 | -2.053 |
| TTC12 | 0.309 | 6.93 | 3.044 | 2.00E-03 | 1.20E-02 | -2.054 |
| ATP11A | -0.303 | 6.591 | -3.044 | 2.00E-03 | 1.30E-02 | -2.057 |
| BRMS1 | 0.248 | 9.3 | 3.042 | 2.00E-03 | 1.30E-02 | -2.06 |
| CAV1 | 0.34 | 11.103 | 3.041 | 2.00E-03 | 1.30E-02 | -2.061 |
| ATP5J2 | 0.327 | 10.971 | 3.04 | 2.00E-03 | 1.30E-02 | -2.064 |
| NUBP1 | 0.402 | 9.259 | 3.039 | 2.00E-03 | 1.30E-02 | -2.068 |
| AIRE | 0.272 | 6.418 | 3.039 | 2.00E-03 | 1.30E-02 | -2.068 |
| EMILIN1 | 0.556 | 9.344 | 3.038 | 2.00E-03 | 1.30E-02 | -2.071 |
| ENDOG | 0.412 | 8.811 | 3.038 | 2.00E-03 | 1.30E-02 | -2.072 |
| COL9A2 | 0.362 | 6.038 | 3.037 | 2.00E-03 | 1.30E-02 | -2.072 |
| GSS | 0.26 | 9.805 | 3.037 | 2.00E-03 | 1.30E-02 | -2.074 |
| ZNF30 | -0.351 | 6.154 | -3.037 | 2.00E-03 | 1.30E-02 | -2.076 |
| APLN | 0.449 | 8.623 | 3.036 | 2.00E-03 | 1.30E-02 | -2.077 |
| KNDC1 | 0.246 | 5.368 | 3.035 | 2.00E-03 | 1.30E-02 | -2.079 |
| MFAP1 | -0.224 | 9.774 | -3.035 | 2.00E-03 | 1.30E-02 | -2.083 |
| ZNF552 | -0.251 | 7.159 | -3.035 | 2.00E-03 | 1.30E-02 | -2.083 |
| ABCD1 | 0.334 | 7.798 | 3.033 | 2.00E-03 | 1.30E-02 | -2.084 |
| PER2 | -0.337 | 7.023 | -3.034 | 2.00E-03 | 1.30E-02 | -2.085 |
| C4BPB | -0.442 | 6.703 | -3.033 | 2.00E-03 | 1.30E-02 | -2.087 |
| AP2S1 | 0.221 | 11.693 | 3.031 | 2.00E-03 | 1.30E-02 | -2.09 |
| IL17RC | 0.261 | 6.956 | 3.031 | 2.00E-03 | 1.30E-02 | -2.09 |
| GPR37 | 0.347 | 6.74 | 3.031 | 2.00E-03 | 1.30E-02 | -2.09 |
| ASB16 | 0.394 | 6.907 | 3.03 | 2.00E-03 | 1.30E-02 | -2.094 |
| UCK1 | 0.204 | 8.798 | 3.027 | 2.00E-03 | 1.30E-02 | -2.101 |
| SERPINB6 | 0.322 | 9.882 | 3.027 | 2.00E-03 | 1.30E-02 | -2.102 |
| GRIN2C | 0.387 | 6.81 | 3.026 | 2.00E-03 | 1.30E-02 | -2.105 |
| LOXL2 | 0.359 | 7.821 | 3.023 | 2.00E-03 | 1.30E-02 | -2.113 |
| EIF2AK1 | 0.33 | 10.503 | 3.023 | 2.00E-03 | 1.30E-02 | -2.114 |
| CNGA1 | 0.499 | 6.287 | 3.022 | 2.00E-03 | 1.30E-02 | -2.115 |
| FBXO21 | -0.204 | 10.116 | -3.022 | 2.00E-03 | 1.30E-02 | -2.119 |
| KIAA1279 | -0.285 | 8.705 | -3.022 | 2.00E-03 | 1.30E-02 | -2.12 |
| NUBPL | -0.248 | 7.312 | -3.02 | 2.00E-03 | 1.30E-02 | -2.125 |
| CCNF | 0.284 | 7.014 | 3.016 | 2.00E-03 | 1.30E-02 | -2.133 |
| ZNF619 | 0.287 | 5.892 | 3.016 | 2.00E-03 | 1.30E-02 | -2.133 |
| SIPA1L2 | -0.438 | 8.799 | -3.016 | 2.00E-03 | 1.30E-02 | -2.135 |
| ZDHHC13 | -0.284 | 7.734 | -3.016 | 2.00E-03 | 1.30E-02 | -2.135 |
| ACMSD | 0.286 | 4.892 | 3.015 | 2.00E-03 | 1.30E-02 | -2.136 |
| TIMP2 | -0.321 | 12.191 | -3.014 | 2.00E-03 | 1.30E-02 | -2.14 |
| MAT1A | 0.385 | 5.175 | 3.01 | 3.00E-03 | 1.40E-02 | -2.148 |
| SSBP1 | -0.29 | 10.221 | -3.011 | 3.00E-03 | 1.40E-02 | -2.15 |
| MTF1 | -0.274 | 8.34 | -3.01 | 3.00E-03 | 1.40E-02 | -2.152 |
| RGS3 | 0.208 | 6.916 | 3.008 | 3.00E-03 | 1.40E-02 | -2.155 |
| TAF1B | -0.254 | 8.116 | -3.008 | 3.00E-03 | 1.40E-02 | -2.157 |
| SLC29A1 | 0.394 | 9.108 | 3.006 | 3.00E-03 | 1.40E-02 | -2.16 |
| GPSM3 | 0.313 | 9.055 | 3.005 | 3.00E-03 | 1.40E-02 | -2.165 |
| ATIC | 0.248 | 10.662 | 3.004 | 3.00E-03 | 1.40E-02 | -2.168 |
| PPP2R5D | 0.21 | 8.674 | 3.003 | 3.00E-03 | 1.40E-02 | -2.17 |
| RNF44 | -0.262 | 9.559 | -3.003 | 3.00E-03 | 1.40E-02 | -2.172 |
| ZFYVE1 | 0.23 | 8.306 | 3.001 | 3.00E-03 | 1.40E-02 | -2.174 |
| SPIB | 0.301 | 6.139 | 3 | 3.00E-03 | 1.40E-02 | -2.179 |
| MEIS1 | -0.305 | 7.518 | -3 | 3.00E-03 | 1.40E-02 | -2.18 |
| POLK | -0.255 | 7.236 | -2.999 | 3.00E-03 | 1.40E-02 | -2.182 |
| JDP2 | -0.351 | 8.352 | -2.999 | 3.00E-03 | 1.40E-02 | -2.183 |
| RAD52 | 0.252 | 6.597 | 2.998 | 3.00E-03 | 1.40E-02 | -2.183 |
| GSTM5 | 0.363 | 6.286 | 2.998 | 3.00E-03 | 1.40E-02 | -2.184 |
| ICK | -0.399 | 8.044 | -2.997 | 3.00E-03 | 1.40E-02 | -2.189 |
| LMO4 | -0.263 | 10.43 | -2.997 | 3.00E-03 | 1.40E-02 | -2.19 |
| SYNJ2 | -0.286 | 7.173 | -2.996 | 3.00E-03 | 1.40E-02 | -2.19 |
| KLHDC3 | 0.257 | 9.601 | 2.995 | 3.00E-03 | 1.40E-02 | -2.191 |
| SERPINB5 | 0.318 | 5.181 | 2.994 | 3.00E-03 | 1.40E-02 | -2.195 |
| DCP2 | -0.313 | 9.569 | -2.993 | 3.00E-03 | 1.40E-02 | -2.199 |
| CRYZ | -0.458 | 8.254 | -2.992 | 3.00E-03 | 1.40E-02 | -2.203 |
| FMO1 | 0.34 | 6.258 | 2.991 | 3.00E-03 | 1.40E-02 | -2.204 |
| CCT5 | -0.286 | 8.499 | -2.99 | 3.00E-03 | 1.40E-02 | -2.207 |
| RPL37 | 0.222 | 10.576 | 2.988 | 3.00E-03 | 1.40E-02 | -2.21 |
| POP4 | 0.194 | 9.674 | 2.988 | 3.00E-03 | 1.40E-02 | -2.212 |
| SLC26A7 | -0.463 | 5.906 | -2.988 | 3.00E-03 | 1.40E-02 | -2.214 |
| ARPC3 | -0.266 | 12.242 | -2.986 | 3.00E-03 | 1.40E-02 | -2.218 |
| PTPN23 | 0.261 | 9.425 | 2.984 | 3.00E-03 | 1.40E-02 | -2.223 |
| NCF4 | -0.28 | 8.304 | -2.984 | 3.00E-03 | 1.40E-02 | -2.224 |
| MASP2 | 0.263 | 5.49 | 2.983 | 3.00E-03 | 1.40E-02 | -2.224 |
| VPS18 | 0.411 | 9.466 | 2.983 | 3.00E-03 | 1.40E-02 | -2.225 |
| CALD1 | -0.364 | 9.681 | -2.984 | 3.00E-03 | 1.40E-02 | -2.226 |
| THBS3 | -0.354 | 7.748 | -2.983 | 3.00E-03 | 1.50E-02 | -2.229 |
| ABCA1 | -0.429 | 8.678 | -2.983 | 3.00E-03 | 1.50E-02 | -2.229 |
| LCE1B | 0.371 | 4.949 | 2.981 | 3.00E-03 | 1.50E-02 | -2.23 |
| ROR1 | 0.307 | 6.254 | 2.981 | 3.00E-03 | 1.50E-02 | -2.23 |
| APRT | 0.249 | 10.676 | 2.98 | 3.00E-03 | 1.50E-02 | -2.233 |
| FHIT | 0.321 | 7.489 | 2.98 | 3.00E-03 | 1.50E-02 | -2.234 |
| CDH10 | 0.325 | 4.885 | 2.977 | 3.00E-03 | 1.50E-02 | -2.242 |
| TTK | -0.445 | 7.199 | -2.978 | 3.00E-03 | 1.50E-02 | -2.243 |
| DLEU1 | 0.251 | 7.2 | 2.976 | 3.00E-03 | 1.50E-02 | -2.243 |
| SYCP1 | 0.351 | 5.451 | 2.976 | 3.00E-03 | 1.50E-02 | -2.245 |
| ITGB3BP | -0.303 | 8.2 | -2.976 | 3.00E-03 | 1.50E-02 | -2.246 |
| MAN1A2 | -0.392 | 9.273 | -2.976 | 3.00E-03 | 1.50E-02 | -2.247 |
| KPNA1 | -0.264 | 8.285 | -2.975 | 3.00E-03 | 1.50E-02 | -2.251 |
| MUC13 | 0.3 | 4.842 | 2.973 | 3.00E-03 | 1.50E-02 | -2.252 |
| TRIM21 | 0.378 | 8.184 | 2.973 | 3.00E-03 | 1.50E-02 | -2.254 |
| NT5C1A | 0.318 | 5.145 | 2.971 | 3.00E-03 | 1.50E-02 | -2.258 |
| COL5A3 | 0.441 | 7.014 | 2.97 | 3.00E-03 | 1.50E-02 | -2.26 |
| MRPS15 | 0.215 | 10.586 | 2.968 | 3.00E-03 | 1.50E-02 | -2.267 |
| VPS33B | 0.225 | 8.385 | 2.967 | 3.00E-03 | 1.50E-02 | -2.27 |
| BRI3BP | 0.296 | 7.065 | 2.967 | 3.00E-03 | 1.50E-02 | -2.27 |
| PCDHB6 | 0.362 | 4.877 | 2.965 | 3.00E-03 | 1.50E-02 | -2.274 |
| CD7 | 0.376 | 6.737 | 2.965 | 3.00E-03 | 1.50E-02 | -2.275 |
| ABHD6 | 0.255 | 7.579 | 2.964 | 3.00E-03 | 1.50E-02 | -2.277 |
| CYP2B6 | 0.284 | 6.315 | 2.964 | 3.00E-03 | 1.50E-02 | -2.279 |
| NUDT5 | -0.279 | 10.637 | -2.964 | 3.00E-03 | 1.50E-02 | -2.279 |
| SLC2A1 | 0.384 | 11.248 | 2.963 | 3.00E-03 | 1.50E-02 | -2.279 |
| KLF10 | -0.278 | 9.237 | -2.964 | 3.00E-03 | 1.50E-02 | -2.281 |
| ATXN7 | -0.361 | 6.539 | -2.961 | 3.00E-03 | 1.50E-02 | -2.289 |
| HIST1H2BJ | 0.372 | 7.493 | 2.96 | 3.00E-03 | 1.50E-02 | -2.289 |
| INSR | -0.495 | 7.741 | -2.961 | 3.00E-03 | 1.50E-02 | -2.289 |
| NDUFV1 | 0.216 | 10.957 | 2.958 | 3.00E-03 | 1.50E-02 | -2.293 |
| DLG3 | 0.296 | 7.481 | 2.958 | 3.00E-03 | 1.50E-02 | -2.294 |
| SPATS2 | 0.309 | 7.241 | 2.958 | 3.00E-03 | 1.50E-02 | -2.294 |
| PTPRJ | -0.323 | 7.321 | -2.953 | 3.00E-03 | 1.60E-02 | -2.309 |
| ZNF559 | -0.419 | 7.242 | -2.953 | 3.00E-03 | 1.60E-02 | -2.309 |
| BYSL | 0.25 | 9.019 | 2.952 | 3.00E-03 | 1.60E-02 | -2.31 |
| UMPS | -0.241 | 8.78 | -2.95 | 3.00E-03 | 1.60E-02 | -2.318 |
| ANKRD27 | -0.261 | 7.907 | -2.949 | 3.00E-03 | 1.60E-02 | -2.32 |
| SLITRK3 | 0.353 | 4.781 | 2.948 | 3.00E-03 | 1.60E-02 | -2.321 |
| PGGT1B | -0.373 | 6.634 | -2.949 | 3.00E-03 | 1.60E-02 | -2.321 |
| GEMIN6 | 0.31 | 8.965 | 2.945 | 3.00E-03 | 1.60E-02 | -2.33 |
| NUPL2 | -0.257 | 8.244 | -2.946 | 3.00E-03 | 1.60E-02 | -2.331 |
| CLYBL | 0.294 | 5.849 | 2.944 | 3.00E-03 | 1.60E-02 | -2.333 |
| IFI27 | 0.528 | 11.727 | 2.943 | 3.00E-03 | 1.60E-02 | -2.335 |
| NEURL2 | 0.309 | 6.838 | 2.941 | 3.00E-03 | 1.60E-02 | -2.341 |
| CD6 | 0.382 | 6.708 | 2.94 | 3.00E-03 | 1.60E-02 | -2.344 |
| NDUFB1 | 0.273 | 9.956 | 2.939 | 3.00E-03 | 1.60E-02 | -2.345 |
| MFAP3 | -0.426 | 7.781 | -2.939 | 3.00E-03 | 1.60E-02 | -2.35 |
| FGD5 | 0.368 | 7.133 | 2.937 | 3.00E-03 | 1.60E-02 | -2.352 |
| KLF7 | -0.33 | 6.752 | -2.931 | 3.00E-03 | 1.70E-02 | -2.372 |
| EFHD1 | 0.432 | 9.99 | 2.928 | 3.00E-03 | 1.70E-02 | -2.377 |
| NETO2 | -0.374 | 7.062 | -2.928 | 3.00E-03 | 1.70E-02 | -2.379 |
| GABRB2 | 0.295 | 5.735 | 2.927 | 3.00E-03 | 1.70E-02 | -2.38 |
| MYBPC2 | 0.451 | 5.592 | 2.925 | 3.00E-03 | 1.70E-02 | -2.384 |
| NUBP2 | 0.251 | 8.538 | 2.924 | 3.00E-03 | 1.70E-02 | -2.387 |
| BCL6B | 0.283 | 6.732 | 2.924 | 3.00E-03 | 1.70E-02 | -2.388 |
| TUSC2 | 0.169 | 9.293 | 2.923 | 3.00E-03 | 1.70E-02 | -2.39 |
| FUT9 | 0.217 | 4.884 | 2.923 | 3.00E-03 | 1.70E-02 | -2.391 |
| CDC25A | 0.36 | 7.141 | 2.922 | 3.00E-03 | 1.70E-02 | -2.391 |
| DHDDS | 0.268 | 7.458 | 2.922 | 3.00E-03 | 1.70E-02 | -2.394 |
| TNC | 0.648 | 6.838 | 2.919 | 4.00E-03 | 1.70E-02 | -2.402 |
| SCARB1 | 0.319 | 11.054 | 2.918 | 4.00E-03 | 1.70E-02 | -2.403 |
| DSCR8 | 0.322 | 7.805 | 2.918 | 4.00E-03 | 1.70E-02 | -2.403 |
| DZIP3 | -0.238 | 6.708 | -2.919 | 4.00E-03 | 1.70E-02 | -2.404 |
| SGCG | 0.3 | 4.706 | 2.918 | 4.00E-03 | 1.70E-02 | -2.405 |
| CALU | 0.294 | 10.748 | 2.917 | 4.00E-03 | 1.70E-02 | -2.407 |
| EGLN3 | -0.517 | 6.81 | -2.916 | 4.00E-03 | 1.70E-02 | -2.411 |
| SLC6A7 | 0.271 | 5.17 | 2.914 | 4.00E-03 | 1.70E-02 | -2.413 |
| KATNB1 | 0.226 | 7.847 | 2.911 | 4.00E-03 | 1.70E-02 | -2.424 |
| ACAD9 | 0.221 | 9.57 | 2.91 | 4.00E-03 | 1.70E-02 | -2.424 |
| ARL1 | -0.284 | 9.54 | -2.911 | 4.00E-03 | 1.70E-02 | -2.426 |
| C14orf119 | -0.174 | 7.137 | -2.91 | 4.00E-03 | 1.70E-02 | -2.428 |
| ZNF398 | -0.269 | 7.91 | -2.91 | 4.00E-03 | 1.70E-02 | -2.428 |
| POU3F4 | 0.379 | 5.696 | 2.908 | 4.00E-03 | 1.70E-02 | -2.43 |
| RHOF | 0.325 | 6.087 | 2.908 | 4.00E-03 | 1.70E-02 | -2.432 |
| CLDN1 | -0.66 | 7.866 | -2.906 | 4.00E-03 | 1.80E-02 | -2.439 |
| DCBLD1 | 0.283 | 6.895 | 2.904 | 4.00E-03 | 1.80E-02 | -2.44 |
| PSMA5 | -0.233 | 11.26 | -2.904 | 4.00E-03 | 1.80E-02 | -2.445 |
| USF2 | 0.254 | 8.681 | 2.902 | 4.00E-03 | 1.80E-02 | -2.447 |
| OR12D2 | 0.333 | 4.998 | 2.901 | 4.00E-03 | 1.80E-02 | -2.449 |
| UBR1 | -0.252 | 8.23 | -2.902 | 4.00E-03 | 1.80E-02 | -2.449 |
| CANX | -0.228 | 9.785 | -2.901 | 4.00E-03 | 1.80E-02 | -2.451 |
| ZNF491 | -0.313 | 6.057 | -2.9 | 4.00E-03 | 1.80E-02 | -2.455 |
| ITSN2 | -0.191 | 7.413 | -2.898 | 4.00E-03 | 1.80E-02 | -2.461 |
| MTMR1 | -0.235 | 7.934 | -2.897 | 4.00E-03 | 1.80E-02 | -2.463 |
| DRAP1 | 0.277 | 11.405 | 2.896 | 4.00E-03 | 1.80E-02 | -2.464 |
| THRB | 0.343 | 5.432 | 2.894 | 4.00E-03 | 1.80E-02 | -2.468 |
| PAM | 0.379 | 10.785 | 2.892 | 4.00E-03 | 1.80E-02 | -2.473 |
| SIX5 | 0.283 | 9.116 | 2.891 | 4.00E-03 | 1.80E-02 | -2.476 |
| H6PD | 0.365 | 6.962 | 2.89 | 4.00E-03 | 1.80E-02 | -2.478 |
| TRAPPC2 | -0.193 | 7.676 | -2.891 | 4.00E-03 | 1.80E-02 | -2.48 |
| PLXNB1 | 0.325 | 7.55 | 2.887 | 4.00E-03 | 1.80E-02 | -2.486 |
| PCBP4 | 0.32 | 7.808 | 2.886 | 4.00E-03 | 1.80E-02 | -2.49 |
| KLC2 | 0.376 | 6.954 | 2.882 | 4.00E-03 | 1.90E-02 | -2.502 |
| ZNF136 | -0.168 | 7.37 | -2.882 | 4.00E-03 | 1.90E-02 | -2.503 |
| PPP1R15A | 0.308 | 10.332 | 2.879 | 4.00E-03 | 1.90E-02 | -2.508 |
| PTPN6 | 0.222 | 8.171 | 2.877 | 4.00E-03 | 1.90E-02 | -2.515 |
| ELMO3 | 0.266 | 7.884 | 2.876 | 4.00E-03 | 1.90E-02 | -2.517 |
| ZCCHC5 | 0.347 | 4.938 | 2.875 | 4.00E-03 | 1.90E-02 | -2.519 |
| ZNF541 | 0.449 | 5.937 | 2.875 | 4.00E-03 | 1.90E-02 | -2.52 |
| MARCKS | -0.3 | 12.264 | -2.874 | 4.00E-03 | 1.90E-02 | -2.526 |
| DHTKD1 | 0.249 | 8.025 | 2.872 | 4.00E-03 | 1.90E-02 | -2.527 |
| CEBPE | -0.343 | 7.115 | -2.872 | 4.00E-03 | 1.90E-02 | -2.53 |
| RHBG | 0.313 | 5.384 | 2.869 | 4.00E-03 | 1.90E-02 | -2.536 |
| CACNA1B | 0.284 | 5.692 | 2.868 | 4.00E-03 | 1.90E-02 | -2.539 |
| PRM2 | 0.358 | 5.275 | 2.867 | 4.00E-03 | 1.90E-02 | -2.541 |
| CLEC4E | -0.335 | 6.346 | -2.868 | 4.00E-03 | 1.90E-02 | -2.542 |
| CSMD1 | 0.392 | 5.83 | 2.865 | 4.00E-03 | 1.90E-02 | -2.546 |
| SUPT4H1 | 0.185 | 10.498 | 2.865 | 4.00E-03 | 1.90E-02 | -2.546 |
| KCNJ1 | 0.258 | 4.968 | 2.864 | 4.00E-03 | 2.00E-02 | -2.55 |
| SLC25A24 | -0.346 | 8.32 | -2.864 | 4.00E-03 | 2.00E-02 | -2.551 |
| GPR15 | 0.374 | 6.05 | 2.862 | 4.00E-03 | 2.00E-02 | -2.554 |
| PPIL3 | -0.262 | 8.942 | -2.862 | 4.00E-03 | 2.00E-02 | -2.557 |
| ZAR1 | 0.317 | 5.118 | 2.861 | 4.00E-03 | 2.00E-02 | -2.558 |
| TMED1 | 0.258 | 9.251 | 2.861 | 4.00E-03 | 2.00E-02 | -2.559 |
| LRBA | -0.236 | 7.853 | -2.861 | 4.00E-03 | 2.00E-02 | -2.56 |
| ZNF26 | -0.302 | 7.728 | -2.861 | 4.00E-03 | 2.00E-02 | -2.561 |
| TFF1 | 0.325 | 6.547 | 2.857 | 4.00E-03 | 2.00E-02 | -2.568 |
| RLBP1 | 0.336 | 5.962 | 2.855 | 4.00E-03 | 2.00E-02 | -2.574 |
| INHA | 0.517 | 7.564 | 2.853 | 4.00E-03 | 2.00E-02 | -2.578 |
| METAP1 | -0.362 | 8.813 | -2.854 | 4.00E-03 | 2.00E-02 | -2.579 |
| CDK9 | 0.225 | 9.109 | 2.853 | 4.00E-03 | 2.00E-02 | -2.58 |
| PRCC | 0.228 | 8.045 | 2.852 | 4.00E-03 | 2.00E-02 | -2.582 |
| TAS1R1 | 0.357 | 6.377 | 2.85 | 4.00E-03 | 2.00E-02 | -2.588 |
| SYN1 | 0.306 | 6.695 | 2.849 | 4.00E-03 | 2.00E-02 | -2.59 |
| NR1H2 | 0.312 | 8.969 | 2.848 | 4.00E-03 | 2.00E-02 | -2.593 |
| ZMYND10 | 0.272 | 6.291 | 2.846 | 4.00E-03 | 2.00E-02 | -2.598 |
| PRKAR2B | -0.315 | 8.343 | -2.845 | 5.00E-03 | 2.10E-02 | -2.601 |
| SKI | 0.286 | 7.484 | 2.844 | 5.00E-03 | 2.10E-02 | -2.602 |
| SOX7 | 0.362 | 7.877 | 2.842 | 5.00E-03 | 2.10E-02 | -2.608 |
| IRF1 | -0.362 | 9.436 | -2.843 | 5.00E-03 | 2.10E-02 | -2.608 |
| ASCC2 | 0.457 | 9.156 | 2.839 | 5.00E-03 | 2.10E-02 | -2.616 |
| FOLH1 | 0.331 | 5.352 | 2.839 | 5.00E-03 | 2.10E-02 | -2.617 |
| GPR27 | -0.289 | 6.619 | -2.838 | 5.00E-03 | 2.10E-02 | -2.62 |
| DHRS3 | 0.36 | 10.382 | 2.836 | 5.00E-03 | 2.10E-02 | -2.623 |
| SF3A2 | 0.356 | 10.05 | 2.836 | 5.00E-03 | 2.10E-02 | -2.623 |
| MPP6 | -0.336 | 6.434 | -2.837 | 5.00E-03 | 2.10E-02 | -2.624 |
| SMURF2 | -0.413 | 6.891 | -2.833 | 5.00E-03 | 2.10E-02 | -2.636 |
| MYCN | 0.425 | 7.432 | 2.831 | 5.00E-03 | 2.10E-02 | -2.638 |
| LIAS | -0.214 | 7.497 | -2.831 | 5.00E-03 | 2.10E-02 | -2.64 |
| ELP4 | 0.302 | 7.826 | 2.83 | 5.00E-03 | 2.10E-02 | -2.641 |
| GNB1L | 0.291 | 8.276 | 2.828 | 5.00E-03 | 2.10E-02 | -2.646 |
| UPK3B | 0.324 | 7.43 | 2.827 | 5.00E-03 | 2.10E-02 | -2.648 |
| GPC1 | 0.343 | 9.348 | 2.825 | 5.00E-03 | 2.20E-02 | -2.653 |
| ADAMTSL2 | 0.318 | 6.214 | 2.825 | 5.00E-03 | 2.20E-02 | -2.654 |
| PRKY | -0.347 | 6.783 | -2.823 | 5.00E-03 | 2.20E-02 | -2.661 |
| TBC1D22B | 0.319 | 7.974 | 2.82 | 5.00E-03 | 2.20E-02 | -2.666 |
| LPIN1 | -0.407 | 8.145 | -2.82 | 5.00E-03 | 2.20E-02 | -2.67 |
| ETFDH | -0.275 | 9.399 | -2.819 | 5.00E-03 | 2.20E-02 | -2.673 |
| TRIM10 | 0.291 | 6.558 | 2.817 | 5.00E-03 | 2.20E-02 | -2.674 |
| LSM2 | 0.198 | 10.44 | 2.816 | 5.00E-03 | 2.20E-02 | -2.676 |
| PLEKHF1 | 0.251 | 9.315 | 2.816 | 5.00E-03 | 2.20E-02 | -2.677 |
| BLCAP | 0.321 | 8.998 | 2.815 | 5.00E-03 | 2.20E-02 | -2.679 |
| EPRS | -0.247 | 10.616 | -2.815 | 5.00E-03 | 2.20E-02 | -2.681 |
| SHANK1 | 0.323 | 6.617 | 2.813 | 5.00E-03 | 2.20E-02 | -2.683 |
| PSEN1 | -0.253 | 8.417 | -2.813 | 5.00E-03 | 2.20E-02 | -2.687 |
| CNFN | 0.254 | 8.038 | 2.812 | 5.00E-03 | 2.20E-02 | -2.689 |
| MRPL4 | 0.199 | 8.917 | 2.811 | 5.00E-03 | 2.20E-02 | -2.69 |
| ARHGEF17 | 0.295 | 8.483 | 2.81 | 5.00E-03 | 2.20E-02 | -2.693 |
| TCN2 | 0.363 | 7.089 | 2.809 | 5.00E-03 | 2.20E-02 | -2.695 |
| HERPUD1 | -0.235 | 11.552 | -2.807 | 5.00E-03 | 2.30E-02 | -2.702 |
| NDUFS2 | -0.236 | 9.259 | -2.807 | 5.00E-03 | 2.30E-02 | -2.703 |
| PBOV1 | 0.303 | 5.482 | 2.806 | 5.00E-03 | 2.30E-02 | -2.704 |
| SMARCA2 | -0.223 | 8.502 | -2.806 | 5.00E-03 | 2.30E-02 | -2.705 |
| DHX36 | -0.235 | 9.616 | -2.804 | 5.00E-03 | 2.30E-02 | -2.71 |
| HPS1 | 0.304 | 7.774 | 2.803 | 5.00E-03 | 2.30E-02 | -2.712 |
| ICA1 | -0.276 | 7.681 | -2.804 | 5.00E-03 | 2.30E-02 | -2.712 |
| FGF2 | -0.375 | 6.622 | -2.803 | 5.00E-03 | 2.30E-02 | -2.712 |
| LY75 | -0.341 | 6.478 | -2.803 | 5.00E-03 | 2.30E-02 | -2.713 |
| ADAM19 | -0.385 | 8.611 | -2.803 | 5.00E-03 | 2.30E-02 | -2.714 |
| RSBN1L | -0.246 | 7.555 | -2.802 | 5.00E-03 | 2.30E-02 | -2.716 |
| FGFR1 | 0.243 | 7.067 | 2.801 | 5.00E-03 | 2.30E-02 | -2.716 |
| NAV1 | 0.346 | 7.302 | 2.8 | 5.00E-03 | 2.30E-02 | -2.719 |
| TCEA1 | -0.21 | 9.278 | -2.801 | 5.00E-03 | 2.30E-02 | -2.72 |
| TIMM23 | 0.208 | 9.841 | 2.799 | 5.00E-03 | 2.30E-02 | -2.721 |
| KCNK7 | 0.355 | 7.322 | 2.798 | 5.00E-03 | 2.30E-02 | -2.725 |
| FTH1 | -0.285 | 12.369 | -2.797 | 5.00E-03 | 2.30E-02 | -2.73 |
| MYOG | 0.386 | 6.97 | 2.796 | 5.00E-03 | 2.30E-02 | -2.73 |
| SLC35A2 | 0.181 | 8.395 | 2.795 | 5.00E-03 | 2.30E-02 | -2.732 |
| ZBTB33 | -0.305 | 7.796 | -2.796 | 5.00E-03 | 2.30E-02 | -2.733 |
| CASP6 | -0.225 | 8.099 | -2.794 | 5.00E-03 | 2.30E-02 | -2.736 |
| BRWD3 | -0.281 | 7.052 | -2.793 | 5.00E-03 | 2.30E-02 | -2.74 |
| DGCR14 | 0.341 | 7.31 | 2.791 | 5.00E-03 | 2.30E-02 | -2.742 |
| DNASE1L1 | 0.173 | 8.732 | 2.788 | 5.00E-03 | 2.40E-02 | -2.749 |
| IFI44 | -0.491 | 8.753 | -2.788 | 5.00E-03 | 2.40E-02 | -2.752 |
| MYOZ3 | 0.31 | 6.433 | 2.787 | 5.00E-03 | 2.40E-02 | -2.753 |
| PVR | 0.338 | 6.942 | 2.787 | 5.00E-03 | 2.40E-02 | -2.754 |
| DYRK3 | 0.385 | 6.578 | 2.783 | 6.00E-03 | 2.40E-02 | -2.763 |
| PHOSPHO1 | 0.364 | 7.439 | 2.783 | 6.00E-03 | 2.40E-02 | -2.764 |
| LRRC28 | 0.242 | 7.152 | 2.782 | 6.00E-03 | 2.40E-02 | -2.765 |
| CPVL | -0.36 | 9.227 | -2.781 | 6.00E-03 | 2.40E-02 | -2.77 |
| SEPP1 | -0.452 | 9.904 | -2.781 | 6.00E-03 | 2.40E-02 | -2.77 |
| ACPP | -0.349 | 7.064 | -2.781 | 6.00E-03 | 2.40E-02 | -2.772 |
| MOSPD1 | -0.367 | 7.977 | -2.781 | 6.00E-03 | 2.40E-02 | -2.772 |
| SENP2 | -0.359 | 8.444 | -2.78 | 6.00E-03 | 2.40E-02 | -2.774 |
| NAB1 | -0.319 | 8.43 | -2.779 | 6.00E-03 | 2.40E-02 | -2.776 |
| UQCRB | -0.349 | 8.543 | -2.779 | 6.00E-03 | 2.40E-02 | -2.777 |
| IL13RA1 | -0.276 | 9.709 | -2.779 | 6.00E-03 | 2.40E-02 | -2.777 |
| CD36 | -0.311 | 9.357 | -2.779 | 6.00E-03 | 2.40E-02 | -2.777 |
| CENPH | -0.294 | 7.266 | -2.778 | 6.00E-03 | 2.40E-02 | -2.779 |
| ZMYND11 | -0.293 | 7.683 | -2.777 | 6.00E-03 | 2.40E-02 | -2.781 |
| SURF6 | -0.289 | 7.508 | -2.775 | 6.00E-03 | 2.40E-02 | -2.788 |
| GLRX2 | -0.228 | 8.185 | -2.772 | 6.00E-03 | 2.50E-02 | -2.795 |
| CORO7 | 0.222 | 8.8 | 2.77 | 6.00E-03 | 2.50E-02 | -2.797 |
| MMP11 | 0.41 | 10.391 | 2.769 | 6.00E-03 | 2.50E-02 | -2.799 |
| RAB3IL1 | 0.398 | 7.956 | 2.768 | 6.00E-03 | 2.50E-02 | -2.802 |
| PPP1R12C | 0.245 | 9.248 | 2.768 | 6.00E-03 | 2.50E-02 | -2.804 |
| RIOK3 | -0.323 | 9.723 | -2.768 | 6.00E-03 | 2.50E-02 | -2.806 |
| KCTD11 | 0.301 | 7.538 | 2.766 | 6.00E-03 | 2.50E-02 | -2.807 |
| EIF4E2 | 0.205 | 10.581 | 2.763 | 6.00E-03 | 2.50E-02 | -2.814 |
| BTBD7 | -0.191 | 7.848 | -2.762 | 6.00E-03 | 2.50E-02 | -2.82 |
| AMOTL2 | 0.27 | 8.476 | 2.761 | 6.00E-03 | 2.50E-02 | -2.821 |
| TP53I3 | 0.327 | 8.37 | 2.761 | 6.00E-03 | 2.50E-02 | -2.821 |
| ZNF124 | -0.305 | 6.672 | -2.761 | 6.00E-03 | 2.50E-02 | -2.823 |
| FCGR2B | 0.297 | 9.287 | 2.758 | 6.00E-03 | 2.50E-02 | -2.828 |
| CLDN12 | -0.262 | 6.412 | -2.759 | 6.00E-03 | 2.50E-02 | -2.829 |
| CASP4 | -0.188 | 11.023 | -2.757 | 6.00E-03 | 2.50E-02 | -2.834 |
| LSM10 | 0.214 | 9.765 | 2.754 | 6.00E-03 | 2.60E-02 | -2.838 |
| HEXA | 0.219 | 8.557 | 2.754 | 6.00E-03 | 2.60E-02 | -2.838 |
| AOC3 | -0.443 | 8.162 | -2.755 | 6.00E-03 | 2.60E-02 | -2.839 |
| RPS6KC1 | -0.261 | 8.078 | -2.749 | 6.00E-03 | 2.60E-02 | -2.853 |
| ADIPOR1 | 0.26 | 11.097 | 2.748 | 6.00E-03 | 2.60E-02 | -2.854 |
| FUBP1 | -0.244 | 8.297 | -2.748 | 6.00E-03 | 2.60E-02 | -2.856 |
| APOBEC3G | 0.386 | 7.67 | 2.746 | 6.00E-03 | 2.60E-02 | -2.859 |
| APOL4 | -0.321 | 6.102 | -2.746 | 6.00E-03 | 2.60E-02 | -2.863 |
| C21orf59 | 0.174 | 9.206 | 2.743 | 6.00E-03 | 2.60E-02 | -2.866 |
| UBE2J2 | -0.262 | 7.876 | -2.744 | 6.00E-03 | 2.60E-02 | -2.867 |
| PHACTR2 | -0.26 | 9.471 | -2.743 | 6.00E-03 | 2.60E-02 | -2.869 |
| CNR2 | 0.303 | 6.288 | 2.742 | 6.00E-03 | 2.60E-02 | -2.87 |
| SPRR3 | 0.463 | 6.33 | 2.742 | 6.00E-03 | 2.60E-02 | -2.871 |
| HSD17B7 | -0.293 | 7.811 | -2.742 | 6.00E-03 | 2.60E-02 | -2.871 |
| COG6 | -0.208 | 7.722 | -2.742 | 6.00E-03 | 2.60E-02 | -2.872 |
| MAN2B1 | 0.272 | 9.19 | 2.741 | 6.00E-03 | 2.60E-02 | -2.872 |
| CASP8 | -0.277 | 6.943 | -2.741 | 6.00E-03 | 2.60E-02 | -2.876 |
| ART5 | 0.435 | 5.574 | 2.739 | 6.00E-03 | 2.60E-02 | -2.877 |
| ATM | -0.275 | 6.115 | -2.739 | 6.00E-03 | 2.70E-02 | -2.88 |
| CECR5 | 0.216 | 8.95 | 2.734 | 6.00E-03 | 2.70E-02 | -2.891 |
| PPP1R16A | 0.257 | 9.042 | 2.732 | 6.00E-03 | 2.70E-02 | -2.895 |
| TSHB | 0.285 | 5.871 | 2.732 | 6.00E-03 | 2.70E-02 | -2.896 |
| ANLN | -0.519 | 7.342 | -2.732 | 6.00E-03 | 2.70E-02 | -2.897 |
| VAMP3 | -0.246 | 10.221 | -2.732 | 7.00E-03 | 2.70E-02 | -2.898 |
| COL16A1 | 0.357 | 7.854 | 2.731 | 7.00E-03 | 2.70E-02 | -2.899 |
| DCTN3 | 0.2 | 10.123 | 2.728 | 7.00E-03 | 2.70E-02 | -2.905 |
| MAX | 0.209 | 8.392 | 2.728 | 7.00E-03 | 2.70E-02 | -2.906 |
| WFDC1 | 0.437 | 8.657 | 2.728 | 7.00E-03 | 2.70E-02 | -2.906 |
| CATSPER1 | 0.388 | 6.557 | 2.727 | 7.00E-03 | 2.70E-02 | -2.908 |
| XAB2 | 0.388 | 7.409 | 2.727 | 7.00E-03 | 2.70E-02 | -2.908 |
| TRIB3 | 0.291 | 9.135 | 2.727 | 7.00E-03 | 2.70E-02 | -2.909 |
| CCT6B | -0.337 | 6.201 | -2.727 | 7.00E-03 | 2.70E-02 | -2.91 |
| MPHOSPH9 | -0.233 | 7.181 | -2.727 | 7.00E-03 | 2.70E-02 | -2.91 |
| RNF40 | 0.235 | 8.02 | 2.726 | 7.00E-03 | 2.70E-02 | -2.911 |
| MDM1 | -0.253 | 6.568 | -2.725 | 7.00E-03 | 2.70E-02 | -2.916 |
| HIF1AN | 0.315 | 7.95 | 2.724 | 7.00E-03 | 2.70E-02 | -2.917 |
| KRTAP11-1 | 0.412 | 5.694 | 2.723 | 7.00E-03 | 2.70E-02 | -2.918 |
| MAFB | -0.348 | 9.459 | -2.724 | 7.00E-03 | 2.70E-02 | -2.919 |
| TRIM4 | -0.245 | 7.91 | -2.723 | 7.00E-03 | 2.70E-02 | -2.92 |
| SLC38A5 | 0.341 | 7.07 | 2.721 | 7.00E-03 | 2.80E-02 | -2.923 |
| ARX | 0.302 | 5.65 | 2.721 | 7.00E-03 | 2.80E-02 | -2.923 |
| DHODH | 0.282 | 6.783 | 2.719 | 7.00E-03 | 2.80E-02 | -2.928 |
| ABCF1 | 0.209 | 10.53 | 2.719 | 7.00E-03 | 2.80E-02 | -2.928 |
| PNRC1 | -0.247 | 9.895 | -2.72 | 7.00E-03 | 2.80E-02 | -2.929 |
| KDELR1 | 0.287 | 9.994 | 2.719 | 7.00E-03 | 2.80E-02 | -2.929 |
| FBXO28 | -0.217 | 8.744 | -2.719 | 7.00E-03 | 2.80E-02 | -2.93 |
| ETV7 | -0.49 | 6.661 | -2.719 | 7.00E-03 | 2.80E-02 | -2.931 |
| SCGB1D2 | 0.433 | 4.879 | 2.718 | 7.00E-03 | 2.80E-02 | -2.932 |
| TRIM44 | -0.248 | 9.17 | -2.719 | 7.00E-03 | 2.80E-02 | -2.932 |
| SPOCK2 | 0.343 | 7.618 | 2.717 | 7.00E-03 | 2.80E-02 | -2.933 |
| MAGEE1 | 0.237 | 5.935 | 2.717 | 7.00E-03 | 2.80E-02 | -2.934 |
| PREP | 0.262 | 8.92 | 2.716 | 7.00E-03 | 2.80E-02 | -2.935 |
| MAN2C1 | 0.262 | 8.642 | 2.716 | 7.00E-03 | 2.80E-02 | -2.936 |
| DLC1 | 0.242 | 7.488 | 2.715 | 7.00E-03 | 2.80E-02 | -2.938 |
| POLL | 0.247 | 7.579 | 2.715 | 7.00E-03 | 2.80E-02 | -2.939 |
| ZCCHC2 | -0.256 | 7.702 | -2.715 | 7.00E-03 | 2.80E-02 | -2.941 |
| SSBP3 | 0.364 | 7.449 | 2.713 | 7.00E-03 | 2.80E-02 | -2.945 |
| MLH3 | -0.23 | 7.55 | -2.714 | 7.00E-03 | 2.80E-02 | -2.945 |
| LY6G5C | 0.268 | 6.216 | 2.711 | 7.00E-03 | 2.80E-02 | -2.948 |
| FUT5 | 0.363 | 5.696 | 2.711 | 7.00E-03 | 2.80E-02 | -2.949 |
| HELLS | -0.315 | 6.581 | -2.71 | 7.00E-03 | 2.80E-02 | -2.953 |
| SART3 | -0.204 | 7.636 | -2.709 | 7.00E-03 | 2.80E-02 | -2.956 |
| RRBP1 | 0.416 | 9.596 | 2.708 | 7.00E-03 | 2.80E-02 | -2.957 |
| TMPRSS4 | 0.358 | 5.316 | 2.707 | 7.00E-03 | 2.80E-02 | -2.958 |
| PDE7A | -0.313 | 6.519 | -2.708 | 7.00E-03 | 2.80E-02 | -2.959 |
| NARF | 0.198 | 9.741 | 2.706 | 7.00E-03 | 2.80E-02 | -2.961 |
| COX6B1 | 0.204 | 12.494 | 2.706 | 7.00E-03 | 2.80E-02 | -2.961 |
| LMNA | 0.395 | 10.467 | 2.705 | 7.00E-03 | 2.80E-02 | -2.964 |
| XRCC3 | 0.293 | 7.524 | 2.705 | 7.00E-03 | 2.80E-02 | -2.965 |
| ZNF317 | 0.229 | 8.2 | 2.703 | 7.00E-03 | 2.90E-02 | -2.969 |
| CETN1 | -0.362 | 4.695 | -2.704 | 7.00E-03 | 2.90E-02 | -2.97 |
| STK31 | -0.343 | 5.456 | -2.703 | 7.00E-03 | 2.90E-02 | -2.971 |
| CYP7A1 | 0.24 | 5.251 | 2.702 | 7.00E-03 | 2.90E-02 | -2.973 |
| ZNF653 | 0.242 | 7.594 | 2.699 | 7.00E-03 | 2.90E-02 | -2.98 |
| EPAS1 | -0.315 | 10.902 | -2.698 | 7.00E-03 | 2.90E-02 | -2.984 |
| UGT8 | -0.277 | 5.271 | -2.696 | 7.00E-03 | 2.90E-02 | -2.991 |
| PUS3 | 0.245 | 8.543 | 2.694 | 7.00E-03 | 2.90E-02 | -2.992 |
| SERPINB1 | -0.257 | 10.232 | -2.695 | 7.00E-03 | 2.90E-02 | -2.992 |
| PHF10 | -0.22 | 9.398 | -2.695 | 7.00E-03 | 2.90E-02 | -2.992 |
| ZFYVE21 | -0.216 | 10.61 | -2.694 | 7.00E-03 | 2.90E-02 | -2.995 |
| PKP1 | 0.368 | 5.701 | 2.693 | 7.00E-03 | 2.90E-02 | -2.995 |
| HRAS | 0.231 | 8.53 | 2.691 | 7.00E-03 | 2.90E-02 | -3.001 |
| TOB1 | 0.309 | 8.227 | 2.69 | 7.00E-03 | 2.90E-02 | -3.002 |
| SLC28A3 | -0.318 | 4.967 | -2.69 | 7.00E-03 | 2.90E-02 | -3.004 |
| PPRC1 | 0.255 | 9.527 | 2.689 | 7.00E-03 | 2.90E-02 | -3.005 |
| ST6GALNAC4 | 0.356 | 7.796 | 2.689 | 7.00E-03 | 3.00E-02 | -3.006 |
| GABPA | -0.345 | 7.959 | -2.688 | 7.00E-03 | 3.00E-02 | -3.011 |
| ANK2 | 0.271 | 5.443 | 2.686 | 7.00E-03 | 3.00E-02 | -3.014 |
| F11 | 0.216 | 4.941 | 2.684 | 8.00E-03 | 3.00E-02 | -3.019 |
| GYS1 | 0.352 | 8.317 | 2.683 | 8.00E-03 | 3.00E-02 | -3.02 |
| CCDC7 | 0.337 | 5.551 | 2.682 | 8.00E-03 | 3.00E-02 | -3.022 |
| CBR4 | -0.255 | 7.972 | -2.683 | 8.00E-03 | 3.00E-02 | -3.023 |
| GLTSCR1 | 0.323 | 7.823 | 2.681 | 8.00E-03 | 3.00E-02 | -3.025 |
| MMP19 | 0.313 | 7.337 | 2.681 | 8.00E-03 | 3.00E-02 | -3.026 |
| PSMA8 | 0.332 | 4.961 | 2.68 | 8.00E-03 | 3.00E-02 | -3.027 |
| TNNT1 | 0.517 | 6.919 | 2.68 | 8.00E-03 | 3.00E-02 | -3.028 |
| KRT9 | 0.328 | 6.075 | 2.679 | 8.00E-03 | 3.00E-02 | -3.03 |
| FUNDC1 | -0.205 | 8.76 | -2.679 | 8.00E-03 | 3.00E-02 | -3.032 |
| NIF3L1 | -0.159 | 10.317 | -2.679 | 8.00E-03 | 3.00E-02 | -3.033 |
| ICMT | 0.289 | 8.056 | 2.677 | 8.00E-03 | 3.00E-02 | -3.034 |
| IPO8 | -0.338 | 7.662 | -2.677 | 8.00E-03 | 3.00E-02 | -3.036 |
| DPAGT1 | 0.349 | 8.485 | 2.675 | 8.00E-03 | 3.00E-02 | -3.039 |
| WTAP | -0.242 | 8.465 | -2.676 | 8.00E-03 | 3.00E-02 | -3.039 |
| IL15 | -0.486 | 7.734 | -2.676 | 8.00E-03 | 3.00E-02 | -3.041 |
| MRPL47 | -0.257 | 9.155 | -2.674 | 8.00E-03 | 3.10E-02 | -3.046 |
| WNK2 | 0.322 | 5.463 | 2.672 | 8.00E-03 | 3.10E-02 | -3.047 |
| GAS2L1 | 0.25 | 7.658 | 2.672 | 8.00E-03 | 3.10E-02 | -3.048 |
| VPS35 | -0.193 | 10.499 | -2.673 | 8.00E-03 | 3.10E-02 | -3.049 |
| COX7A2 | 0.24 | 12.449 | 2.671 | 8.00E-03 | 3.10E-02 | -3.051 |
| GML | 0.336 | 5.929 | 2.67 | 8.00E-03 | 3.10E-02 | -3.052 |
| CCL21 | 0.413 | 6.19 | 2.668 | 8.00E-03 | 3.10E-02 | -3.058 |
| SLC22A11 | 0.26 | 8.862 | 2.666 | 8.00E-03 | 3.10E-02 | -3.062 |
| RABIF | -0.224 | 7.035 | -2.667 | 8.00E-03 | 3.10E-02 | -3.063 |
| STMN2 | 0.462 | 5.766 | 2.664 | 8.00E-03 | 3.10E-02 | -3.068 |
| RAD51 | -0.339 | 7.929 | -2.664 | 8.00E-03 | 3.10E-02 | -3.069 |
| SLC2A4RG | 0.296 | 9.142 | 2.663 | 8.00E-03 | 3.10E-02 | -3.071 |
| FCAR | -0.246 | 5.946 | -2.664 | 8.00E-03 | 3.10E-02 | -3.071 |
| COX11 | -0.187 | 7.577 | -2.664 | 8.00E-03 | 3.10E-02 | -3.071 |
| REG3A | 0.339 | 5.138 | 2.661 | 8.00E-03 | 3.10E-02 | -3.075 |
| KDELR2 | -0.201 | 11.228 | -2.662 | 8.00E-03 | 3.10E-02 | -3.076 |
| TAF1 | 0.229 | 7.239 | 2.66 | 8.00E-03 | 3.10E-02 | -3.078 |
| FZD1 | -0.349 | 8.111 | -2.661 | 8.00E-03 | 3.10E-02 | -3.078 |
| ME3 | 0.252 | 6.723 | 2.66 | 8.00E-03 | 3.10E-02 | -3.079 |
| SNX2 | -0.263 | 9.988 | -2.66 | 8.00E-03 | 3.10E-02 | -3.079 |
| UNC13D | 0.376 | 7.709 | 2.659 | 8.00E-03 | 3.10E-02 | -3.08 |
| AKAP9 | -0.251 | 7.427 | -2.66 | 8.00E-03 | 3.10E-02 | -3.08 |
| MXI1 | 0.324 | 8.101 | 2.658 | 8.00E-03 | 3.20E-02 | -3.082 |
| MEST | -0.342 | 9.762 | -2.657 | 8.00E-03 | 3.20E-02 | -3.089 |
| BMP15 | 0.312 | 4.869 | 2.655 | 8.00E-03 | 3.20E-02 | -3.09 |
| DLST | 0.293 | 8.678 | 2.653 | 8.00E-03 | 3.20E-02 | -3.094 |
| ZCCHC6 | -0.237 | 8.383 | -2.654 | 8.00E-03 | 3.20E-02 | -3.094 |
| TRHDE | 0.209 | 4.583 | 2.652 | 8.00E-03 | 3.20E-02 | -3.097 |
| DNMT3A | -0.302 | 7.664 | -2.652 | 8.00E-03 | 3.20E-02 | -3.101 |
| ZFP28 | -0.293 | 5.445 | -2.651 | 8.00E-03 | 3.20E-02 | -3.103 |
| MMP2 | 0.338 | 7.197 | 2.65 | 8.00E-03 | 3.20E-02 | -3.104 |
| TJP2 | -0.263 | 8.645 | -2.648 | 8.00E-03 | 3.20E-02 | -3.111 |
| SON | -0.203 | 8.908 | -2.647 | 8.00E-03 | 3.20E-02 | -3.112 |
| MAFF | 0.466 | 9.474 | 2.644 | 8.00E-03 | 3.30E-02 | -3.117 |
| PCGF3 | -0.201 | 7.685 | -2.644 | 9.00E-03 | 3.30E-02 | -3.12 |
| ONECUT1 | 0.321 | 4.983 | 2.643 | 9.00E-03 | 3.30E-02 | -3.12 |
| VRK1 | -0.272 | 8.082 | -2.643 | 9.00E-03 | 3.30E-02 | -3.122 |
| FBXO33 | -0.226 | 8.981 | -2.643 | 9.00E-03 | 3.30E-02 | -3.122 |
| SGPL1 | -0.221 | 7.669 | -2.643 | 9.00E-03 | 3.30E-02 | -3.124 |
| INA | 0.296 | 6.515 | 2.639 | 9.00E-03 | 3.30E-02 | -3.129 |
| SLC9A5 | 0.312 | 6.573 | 2.639 | 9.00E-03 | 3.30E-02 | -3.131 |
| BAP1 | 0.265 | 8.699 | 2.639 | 9.00E-03 | 3.30E-02 | -3.131 |
| KRT8 | 0.318 | 10.023 | 2.636 | 9.00E-03 | 3.30E-02 | -3.138 |
| TRAF4 | 0.213 | 7.895 | 2.635 | 9.00E-03 | 3.30E-02 | -3.139 |
| MBL2 | 0.238 | 4.626 | 2.635 | 9.00E-03 | 3.30E-02 | -3.139 |
| TBCA | -0.213 | 12.678 | -2.636 | 9.00E-03 | 3.30E-02 | -3.14 |
| HN1 | -0.418 | 10.476 | -2.635 | 9.00E-03 | 3.30E-02 | -3.142 |
| CR2 | 0.325 | 5.92 | 2.634 | 9.00E-03 | 3.30E-02 | -3.143 |
| AUP1 | -0.215 | 9.845 | -2.634 | 9.00E-03 | 3.30E-02 | -3.144 |
| UGCG | -0.302 | 9.167 | -2.633 | 9.00E-03 | 3.30E-02 | -3.147 |
| RNF126 | 0.206 | 9.173 | 2.632 | 9.00E-03 | 3.30E-02 | -3.147 |
| ZNF232 | 0.244 | 7.6 | 2.63 | 9.00E-03 | 3.40E-02 | -3.152 |
| SCARF2 | 0.328 | 8.398 | 2.63 | 9.00E-03 | 3.40E-02 | -3.152 |
| LETM1 | 0.223 | 7.41 | 2.629 | 9.00E-03 | 3.40E-02 | -3.156 |
| IRF8 | -0.306 | 7.212 | -2.63 | 9.00E-03 | 3.40E-02 | -3.156 |
| H2AFY | -0.187 | 10.711 | -2.629 | 9.00E-03 | 3.40E-02 | -3.157 |
| SEC23A | -0.271 | 8.671 | -2.628 | 9.00E-03 | 3.40E-02 | -3.159 |
| SLC13A3 | 0.327 | 7.194 | 2.627 | 9.00E-03 | 3.40E-02 | -3.16 |
| CCL1 | 0.256 | 5.592 | 2.626 | 9.00E-03 | 3.40E-02 | -3.162 |
| SLA | -0.296 | 8.418 | -2.627 | 9.00E-03 | 3.40E-02 | -3.163 |
| ANKRA2 | -0.22 | 8.709 | -2.626 | 9.00E-03 | 3.40E-02 | -3.164 |
| FCN3 | 0.294 | 8.153 | 2.625 | 9.00E-03 | 3.40E-02 | -3.166 |
| ELN | 0.341 | 6.445 | 2.625 | 9.00E-03 | 3.40E-02 | -3.166 |
| FDFT1 | -0.237 | 10.89 | -2.625 | 9.00E-03 | 3.40E-02 | -3.168 |
| IMPACT | -0.213 | 7.602 | -2.624 | 9.00E-03 | 3.40E-02 | -3.169 |
| METTL1 | 0.209 | 7.931 | 2.623 | 9.00E-03 | 3.40E-02 | -3.169 |
| PPM1A | -0.234 | 8.122 | -2.624 | 9.00E-03 | 3.40E-02 | -3.169 |
| DISP1 | -0.261 | 7.08 | -2.624 | 9.00E-03 | 3.40E-02 | -3.171 |
| TEX13A | 0.299 | 4.928 | 2.622 | 9.00E-03 | 3.40E-02 | -3.173 |
| FHL3 | 0.384 | 8.02 | 2.621 | 9.00E-03 | 3.40E-02 | -3.176 |
| CXCL5 | -0.531 | 6.682 | -2.621 | 9.00E-03 | 3.40E-02 | -3.178 |
| ATAD2 | -0.219 | 7.802 | -2.62 | 9.00E-03 | 3.40E-02 | -3.179 |
| CDC25B | 0.254 | 9.345 | 2.619 | 9.00E-03 | 3.40E-02 | -3.18 |
| RTN4R | 0.335 | 7.515 | 2.618 | 9.00E-03 | 3.40E-02 | -3.182 |
| PRKAR1A | -0.428 | 10.214 | -2.619 | 9.00E-03 | 3.40E-02 | -3.183 |
| E2F1 | 0.271 | 6.446 | 2.617 | 9.00E-03 | 3.40E-02 | -3.185 |
| LTB4R | -0.223 | 6.93 | -2.617 | 9.00E-03 | 3.50E-02 | -3.187 |
| LPPR4 | -0.372 | 5.665 | -2.617 | 9.00E-03 | 3.50E-02 | -3.187 |
| PRM3 | 0.286 | 6.283 | 2.615 | 9.00E-03 | 3.50E-02 | -3.189 |
| DEF6 | -0.252 | 7.803 | -2.615 | 9.00E-03 | 3.50E-02 | -3.193 |
| CPN1 | 0.347 | 5.836 | 2.613 | 9.00E-03 | 3.50E-02 | -3.195 |
| PTPRT | 0.32 | 5.828 | 2.61 | 9.00E-03 | 3.50E-02 | -3.202 |
| RGAG1 | 0.301 | 5.446 | 2.61 | 9.00E-03 | 3.50E-02 | -3.202 |
| TCTA | 0.251 | 8.625 | 2.608 | 9.00E-03 | 3.50E-02 | -3.208 |
| CALCRL | -0.348 | 6.051 | -2.609 | 9.00E-03 | 3.50E-02 | -3.208 |
| RING1 | 0.178 | 10.201 | 2.606 | 1.00E-02 | 3.50E-02 | -3.213 |
| KIF13B | 0.236 | 8.218 | 2.605 | 1.00E-02 | 3.60E-02 | -3.215 |
| CCBE1 | 0.235 | 5.189 | 2.604 | 1.00E-02 | 3.60E-02 | -3.216 |
| OXA1L | 0.218 | 9.923 | 2.604 | 1.00E-02 | 3.60E-02 | -3.217 |
| TRPV5 | 0.323 | 5.693 | 2.604 | 1.00E-02 | 3.60E-02 | -3.217 |
| SOAT2 | 0.43 | 5.899 | 2.603 | 1.00E-02 | 3.60E-02 | -3.219 |
| DNAL4 | 0.315 | 9.033 | 2.603 | 1.00E-02 | 3.60E-02 | -3.22 |
| SIGLEC5 | 0.268 | 6.247 | 2.601 | 1.00E-02 | 3.60E-02 | -3.223 |
| SMO | 0.317 | 7.368 | 2.6 | 1.00E-02 | 3.60E-02 | -3.227 |
| SMTN | 0.35 | 7.265 | 2.596 | 1.00E-02 | 3.60E-02 | -3.237 |
| RHOT1 | -0.24 | 7.406 | -2.596 | 1.00E-02 | 3.60E-02 | -3.239 |
| GPR6 | 0.262 | 5.831 | 2.594 | 1.00E-02 | 3.60E-02 | -3.241 |
| GIT1 | 0.236 | 7.488 | 2.594 | 1.00E-02 | 3.60E-02 | -3.242 |
| HTR5A | 0.224 | 4.694 | 2.592 | 1.00E-02 | 3.70E-02 | -3.246 |
| RETNLB | 0.317 | 5.745 | 2.592 | 1.00E-02 | 3.70E-02 | -3.246 |
| PHYHD1 | 0.246 | 6.846 | 2.592 | 1.00E-02 | 3.70E-02 | -3.246 |
| PRKAR2A | 0.277 | 7.106 | 2.59 | 1.00E-02 | 3.70E-02 | -3.25 |
| DEPDC1B | -0.452 | 9.185 | -2.591 | 1.00E-02 | 3.70E-02 | -3.251 |
| TRIM35 | 0.271 | 7.677 | 2.589 | 1.00E-02 | 3.70E-02 | -3.253 |
| ADCYAP1 | 0.264 | 5.343 | 2.588 | 1.00E-02 | 3.70E-02 | -3.255 |
| RGS6 | 0.273 | 5.58 | 2.588 | 1.00E-02 | 3.70E-02 | -3.256 |
| PRKCH | -0.223 | 9.349 | -2.589 | 1.00E-02 | 3.70E-02 | -3.257 |
| GPR20 | 0.355 | 6.842 | 2.586 | 1.00E-02 | 3.70E-02 | -3.26 |
| TIMM8A | -0.208 | 7.461 | -2.586 | 1.00E-02 | 3.70E-02 | -3.263 |
| SLC6A19 | -0.293 | 6.012 | -2.586 | 1.00E-02 | 3.70E-02 | -3.263 |
| ADAM12 | -0.391 | 9.933 | -2.585 | 1.00E-02 | 3.70E-02 | -3.266 |
| FAM20C | 0.313 | 8.197 | 2.583 | 1.00E-02 | 3.70E-02 | -3.268 |
| VSIG1 | 0.275 | 5.55 | 2.582 | 1.00E-02 | 3.70E-02 | -3.27 |
| ZNF184 | -0.232 | 7.302 | -2.583 | 1.00E-02 | 3.70E-02 | -3.27 |
| WISP1 | 0.279 | 4.918 | 2.582 | 1.00E-02 | 3.70E-02 | -3.27 |
| SHFM1 | -0.216 | 11.099 | -2.582 | 1.00E-02 | 3.70E-02 | -3.272 |
| TRIM59 | -0.307 | 6.095 | -2.582 | 1.00E-02 | 3.70E-02 | -3.273 |
| PAICS | -0.227 | 9.747 | -2.579 | 1.00E-02 | 3.80E-02 | -3.279 |
| PLS3 | -0.275 | 8.981 | -2.575 | 1.00E-02 | 3.80E-02 | -3.289 |
| PIK3AP1 | -0.483 | 7.116 | -2.575 | 1.00E-02 | 3.80E-02 | -3.29 |
| PINX1 | 0.203 | 7.487 | 2.574 | 1.00E-02 | 3.80E-02 | -3.291 |
| CHAF1A | 0.206 | 8.203 | 2.572 | 1.00E-02 | 3.80E-02 | -3.294 |
| DIRAS3 | -0.291 | 5.088 | -2.573 | 1.00E-02 | 3.80E-02 | -3.295 |
| ATOH8 | 0.385 | 7.157 | 2.572 | 1.00E-02 | 3.80E-02 | -3.295 |
| HYOU1 | 0.373 | 8.639 | 2.567 | 1.10E-02 | 3.90E-02 | -3.306 |
| RGS16 | 0.354 | 7.662 | 2.566 | 1.10E-02 | 3.90E-02 | -3.308 |
| GNE | -0.243 | 10.87 | -2.567 | 1.10E-02 | 3.90E-02 | -3.31 |
| MED6 | -0.172 | 9.033 | -2.566 | 1.10E-02 | 3.90E-02 | -3.311 |
| CA5A | 0.277 | 5.757 | 2.565 | 1.10E-02 | 3.90E-02 | -3.312 |
| SLC5A11 | -0.354 | 5.652 | -2.566 | 1.10E-02 | 3.90E-02 | -3.313 |
| SV2B | 0.449 | 6.252 | 2.564 | 1.10E-02 | 3.90E-02 | -3.314 |
| PIAS4 | 0.29 | 8.063 | 2.564 | 1.10E-02 | 3.90E-02 | -3.315 |
| CTSH | 0.333 | 9.866 | 2.563 | 1.10E-02 | 3.90E-02 | -3.317 |
| VPS13A | -0.206 | 7.472 | -2.563 | 1.10E-02 | 3.90E-02 | -3.318 |
| AHCY | -0.246 | 10.955 | -2.563 | 1.10E-02 | 3.90E-02 | -3.318 |
| LSM3 | 0.23 | 10.499 | 2.562 | 1.10E-02 | 3.90E-02 | -3.319 |
| SPTBN4 | 0.232 | 5.469 | 2.561 | 1.10E-02 | 3.90E-02 | -3.321 |
| OR2H2 | 0.406 | 6.751 | 2.561 | 1.10E-02 | 3.90E-02 | -3.321 |
| ZNF614 | -0.27 | 7.307 | -2.562 | 1.10E-02 | 3.90E-02 | -3.322 |
| CD22 | -0.317 | 5.732 | -2.561 | 1.10E-02 | 3.90E-02 | -3.323 |
| ENTPD2 | 0.318 | 6.557 | 2.559 | 1.10E-02 | 3.90E-02 | -3.326 |
| C3orf18 | 0.287 | 7.643 | 2.559 | 1.10E-02 | 3.90E-02 | -3.327 |
| TGM7 | 0.239 | 5.805 | 2.558 | 1.10E-02 | 3.90E-02 | -3.328 |
| FETUB | 0.325 | 5.368 | 2.557 | 1.10E-02 | 3.90E-02 | -3.33 |
| PODXL2 | 0.276 | 5.808 | 2.557 | 1.10E-02 | 3.90E-02 | -3.331 |
| GRIN2D | 0.309 | 8.387 | 2.557 | 1.10E-02 | 3.90E-02 | -3.332 |
| MLX | -0.166 | 8.342 | -2.558 | 1.10E-02 | 3.90E-02 | -3.332 |
| TFEB | 0.285 | 8.457 | 2.556 | 1.10E-02 | 3.90E-02 | -3.333 |
| MAPK3 | 0.289 | 9.427 | 2.556 | 1.10E-02 | 3.90E-02 | -3.335 |
| TIE1 | 0.332 | 7.716 | 2.555 | 1.10E-02 | 4.00E-02 | -3.336 |
| MAP6 | 0.279 | 5.833 | 2.554 | 1.10E-02 | 4.00E-02 | -3.338 |
| ACAD8 | -0.251 | 7.327 | -2.554 | 1.10E-02 | 4.00E-02 | -3.34 |
| FAM3A | 0.257 | 8.795 | 2.553 | 1.10E-02 | 4.00E-02 | -3.341 |
| P2RY12 | -0.364 | 5.644 | -2.553 | 1.10E-02 | 4.00E-02 | -3.343 |
| EBAG9 | -0.186 | 8.339 | -2.553 | 1.10E-02 | 4.00E-02 | -3.343 |
| STC1 | 0.533 | 7.684 | 2.549 | 1.10E-02 | 4.00E-02 | -3.351 |
| PDCL2 | 0.235 | 4.644 | 2.547 | 1.10E-02 | 4.00E-02 | -3.354 |
| CLDN14 | -0.294 | 6.52 | -2.548 | 1.10E-02 | 4.00E-02 | -3.354 |
| KRTAP4-5 | 0.302 | 5.161 | 2.543 | 1.10E-02 | 4.10E-02 | -3.366 |
| PCDHA9 | 0.267 | 4.881 | 2.542 | 1.10E-02 | 4.10E-02 | -3.366 |
| OR2H1 | 0.242 | 5.887 | 2.541 | 1.10E-02 | 4.10E-02 | -3.371 |
| MPP2 | 0.302 | 6.136 | 2.539 | 1.20E-02 | 4.10E-02 | -3.373 |
| LTBP4 | 0.254 | 8.008 | 2.538 | 1.20E-02 | 4.10E-02 | -3.377 |
| LIPT1 | -0.22 | 7.398 | -2.538 | 1.20E-02 | 4.10E-02 | -3.378 |
| TM7SF2 | 0.297 | 8.012 | 2.536 | 1.20E-02 | 4.10E-02 | -3.381 |
| ZNF197 | -0.241 | 6.919 | -2.537 | 1.20E-02 | 4.10E-02 | -3.381 |
| KLK5 | 0.322 | 5.379 | 2.531 | 1.20E-02 | 4.20E-02 | -3.393 |
| BCL2L13 | 0.168 | 8.723 | 2.531 | 1.20E-02 | 4.20E-02 | -3.395 |
| PAK7 | 0.243 | 4.831 | 2.53 | 1.20E-02 | 4.20E-02 | -3.395 |
| ZNF407 | -0.199 | 6.762 | -2.531 | 1.20E-02 | 4.20E-02 | -3.395 |
| NSUN4 | 0.167 | 8.187 | 2.53 | 1.20E-02 | 4.20E-02 | -3.396 |
| CDADC1 | -0.236 | 6.508 | -2.53 | 1.20E-02 | 4.20E-02 | -3.398 |
| AQP8 | 0.31 | 5.881 | 2.528 | 1.20E-02 | 4.20E-02 | -3.4 |
| SLC25A19 | 0.24 | 7.785 | 2.527 | 1.20E-02 | 4.20E-02 | -3.404 |
| ANGPTL6 | 0.371 | 6.87 | 2.527 | 1.20E-02 | 4.20E-02 | -3.404 |
| DUSP19 | -0.255 | 5.86 | -2.527 | 1.20E-02 | 4.20E-02 | -3.404 |
| SLC4A8 | 0.262 | 5.457 | 2.526 | 1.20E-02 | 4.20E-02 | -3.405 |
| GRPEL1 | -0.189 | 8.998 | -2.525 | 1.20E-02 | 4.20E-02 | -3.409 |
| MS4A3 | -0.387 | 5.603 | -2.525 | 1.20E-02 | 4.20E-02 | -3.409 |
| KIAA1715 | -0.297 | 6.655 | -2.525 | 1.20E-02 | 4.20E-02 | -3.41 |
| MDH1 | -0.148 | 10.822 | -2.524 | 1.20E-02 | 4.20E-02 | -3.412 |
| NUP155 | -0.171 | 8.17 | -2.524 | 1.20E-02 | 4.20E-02 | -3.412 |
| PARP16 | 0.243 | 7.609 | 2.523 | 1.20E-02 | 4.20E-02 | -3.412 |
| EXOSC6 | 0.263 | 7.978 | 2.523 | 1.20E-02 | 4.30E-02 | -3.413 |
| CRYBB1 | 0.334 | 6.072 | 2.522 | 1.20E-02 | 4.30E-02 | -3.416 |
| COMMD7 | 0.315 | 10.411 | 2.521 | 1.20E-02 | 4.30E-02 | -3.417 |
| RBM14 | 0.202 | 9.33 | 2.521 | 1.20E-02 | 4.30E-02 | -3.418 |
| PTPRH | 0.334 | 5.991 | 2.52 | 1.20E-02 | 4.30E-02 | -3.42 |
| ZBTB26 | -0.29 | 6.294 | -2.519 | 1.20E-02 | 4.30E-02 | -3.425 |
| PEX11B | 0.189 | 9.659 | 2.518 | 1.20E-02 | 4.30E-02 | -3.425 |
| KRTAP3-1 | 0.339 | 6.233 | 2.517 | 1.20E-02 | 4.30E-02 | -3.426 |
| CACNG4 | 0.233 | 5.874 | 2.516 | 1.20E-02 | 4.30E-02 | -3.429 |
| CST6 | 0.539 | 7.8 | 2.515 | 1.20E-02 | 4.30E-02 | -3.432 |
| SLAMF7 | -0.449 | 6.668 | -2.516 | 1.20E-02 | 4.30E-02 | -3.433 |
| KCTD13 | 0.275 | 6.571 | 2.513 | 1.20E-02 | 4.30E-02 | -3.436 |
| COPS8 | -0.19 | 8.752 | -2.514 | 1.20E-02 | 4.30E-02 | -3.437 |
| AMN | 0.373 | 7.199 | 2.513 | 1.20E-02 | 4.30E-02 | -3.437 |
| SPACA3 | 0.322 | 6.482 | 2.512 | 1.20E-02 | 4.30E-02 | -3.438 |
| TMCC1 | -0.263 | 7.867 | -2.513 | 1.20E-02 | 4.30E-02 | -3.438 |
| IL7 | -0.374 | 5.346 | -2.512 | 1.30E-02 | 4.40E-02 | -3.442 |
| ESRRG | -0.279 | 7.172 | -2.51 | 1.30E-02 | 4.40E-02 | -3.446 |
| PSMA7 | 0.185 | 10.201 | 2.508 | 1.30E-02 | 4.40E-02 | -3.449 |
| HNF4A | 0.224 | 4.84 | 2.507 | 1.30E-02 | 4.40E-02 | -3.451 |
| BRPF3 | -0.258 | 7.615 | -2.507 | 1.30E-02 | 4.40E-02 | -3.454 |
| ARL6 | -0.257 | 5.556 | -2.506 | 1.30E-02 | 4.40E-02 | -3.455 |
| TSPYL5 | -0.34 | 7.021 | -2.506 | 1.30E-02 | 4.40E-02 | -3.455 |
| ENTPD4 | -0.27 | 8.003 | -2.506 | 1.30E-02 | 4.40E-02 | -3.455 |
| EPHA2 | 0.277 | 7.692 | 2.504 | 1.30E-02 | 4.40E-02 | -3.457 |
| MRPL11 | 0.204 | 8.745 | 2.504 | 1.30E-02 | 4.40E-02 | -3.458 |
| TEX11 | -0.247 | 4.913 | -2.505 | 1.30E-02 | 4.40E-02 | -3.459 |
| UGT2B15 | 0.299 | 5.651 | 2.502 | 1.30E-02 | 4.40E-02 | -3.463 |
| RPS14 | 0.2 | 13.004 | 2.501 | 1.30E-02 | 4.40E-02 | -3.464 |
| OR4D2 | 0.292 | 6.051 | 2.5 | 1.30E-02 | 4.50E-02 | -3.468 |
| ZNF587 | -0.219 | 7.961 | -2.501 | 1.30E-02 | 4.50E-02 | -3.468 |
| ASXL1 | 0.229 | 8.945 | 2.499 | 1.30E-02 | 4.50E-02 | -3.469 |
| MBP | -0.242 | 6.123 | -2.498 | 1.30E-02 | 4.50E-02 | -3.475 |
| CENPB | 0.263 | 9.525 | 2.496 | 1.30E-02 | 4.50E-02 | -3.476 |
| COPZ2 | 0.317 | 8.142 | 2.494 | 1.30E-02 | 4.50E-02 | -3.48 |
| CDKN2B | -0.289 | 6.19 | -2.495 | 1.30E-02 | 4.50E-02 | -3.481 |
| HMGN2 | -0.185 | 11.05 | -2.495 | 1.30E-02 | 4.50E-02 | -3.481 |
| SLC13A5 | 0.316 | 4.883 | 2.493 | 1.30E-02 | 4.50E-02 | -3.483 |
| STXBP5 | -0.247 | 7.557 | -2.494 | 1.30E-02 | 4.50E-02 | -3.484 |
| MANBAL | 0.191 | 9.402 | 2.493 | 1.30E-02 | 4.50E-02 | -3.485 |
| CHST11 | -0.221 | 8.306 | -2.493 | 1.30E-02 | 4.50E-02 | -3.486 |
| SLC2A13 | 0.198 | 5.702 | 2.49 | 1.30E-02 | 4.60E-02 | -3.49 |
| AKAP11 | -0.2 | 7.957 | -2.491 | 1.30E-02 | 4.60E-02 | -3.49 |
| FGF22 | 0.276 | 5.15 | 2.49 | 1.30E-02 | 4.60E-02 | -3.491 |
| DDX3Y | -0.866 | 6.689 | -2.488 | 1.30E-02 | 4.60E-02 | -3.497 |
| KCNJ8 | 0.399 | 7.317 | 2.486 | 1.30E-02 | 4.60E-02 | -3.499 |
| CD79A | 0.317 | 8.021 | 2.484 | 1.30E-02 | 4.60E-02 | -3.504 |
| TAF7 | -0.239 | 10.817 | -2.485 | 1.30E-02 | 4.60E-02 | -3.505 |
| NRD1 | 0.189 | 11.039 | 2.484 | 1.30E-02 | 4.60E-02 | -3.505 |
| SMPDL3B | 0.332 | 5.84 | 2.484 | 1.30E-02 | 4.60E-02 | -3.506 |
| TNFRSF21 | 0.268 | 9.407 | 2.483 | 1.40E-02 | 4.60E-02 | -3.506 |
| ATP5B | -0.149 | 12.626 | -2.483 | 1.40E-02 | 4.60E-02 | -3.51 |
| CD53 | -0.32 | 8.46 | -2.483 | 1.40E-02 | 4.60E-02 | -3.511 |
| CSTF3 | -0.16 | 8.534 | -2.483 | 1.40E-02 | 4.60E-02 | -3.511 |
| TUBGCP5 | -0.238 | 6.835 | -2.481 | 1.40E-02 | 4.70E-02 | -3.514 |
| CHDH | 0.241 | 5.79 | 2.48 | 1.40E-02 | 4.70E-02 | -3.514 |
| MT1F | 0.503 | 8.056 | 2.479 | 1.40E-02 | 4.70E-02 | -3.516 |
| PTDSS1 | 0.201 | 10.549 | 2.479 | 1.40E-02 | 4.70E-02 | -3.517 |
| SUMF2 | -0.353 | 9.125 | -2.479 | 1.40E-02 | 4.70E-02 | -3.519 |
| FGL1 | -0.249 | 6.643 | -2.477 | 1.40E-02 | 4.70E-02 | -3.523 |
| LEF1 | 0.267 | 7.55 | 2.476 | 1.40E-02 | 4.70E-02 | -3.523 |
| CPSF1 | 0.292 | 8.392 | 2.476 | 1.40E-02 | 4.70E-02 | -3.523 |
| FNBP1 | -0.219 | 9.548 | -2.476 | 1.40E-02 | 4.70E-02 | -3.526 |
| CD79B | 0.306 | 6.694 | 2.475 | 1.40E-02 | 4.70E-02 | -3.527 |
| SURF2 | 0.349 | 8.072 | 2.475 | 1.40E-02 | 4.70E-02 | -3.527 |
| KCNQ1 | -0.183 | 7.348 | -2.475 | 1.40E-02 | 4.70E-02 | -3.529 |
| ADSL | 0.187 | 10.122 | 2.474 | 1.40E-02 | 4.70E-02 | -3.529 |
| TMEM35 | 0.354 | 5.573 | 2.473 | 1.40E-02 | 4.70E-02 | -3.53 |
| LRRC14 | 0.306 | 7.935 | 2.473 | 1.40E-02 | 4.70E-02 | -3.531 |
| NUDT16 | 0.232 | 7.583 | 2.472 | 1.40E-02 | 4.70E-02 | -3.533 |
| RGS18 | -0.336 | 7.234 | -2.473 | 1.40E-02 | 4.70E-02 | -3.533 |
| MC2R | 0.326 | 5.42 | 2.472 | 1.40E-02 | 4.70E-02 | -3.534 |
| FEZ1 | 0.232 | 7.372 | 2.471 | 1.40E-02 | 4.70E-02 | -3.536 |
| NAPG | 0.204 | 8.687 | 2.47 | 1.40E-02 | 4.70E-02 | -3.537 |
| MSN | 0.225 | 10.011 | 2.466 | 1.40E-02 | 4.80E-02 | -3.547 |
| CAPZB | 0.167 | 9.953 | 2.465 | 1.40E-02 | 4.80E-02 | -3.55 |
| PSMB7 | 0.163 | 12.021 | 2.462 | 1.40E-02 | 4.80E-02 | -3.557 |
| PSMD8 | 0.217 | 10.665 | 2.462 | 1.40E-02 | 4.80E-02 | -3.557 |
| GNPDA1 | -0.245 | 9.286 | -2.463 | 1.40E-02 | 4.80E-02 | -3.557 |
| ZW10 | -0.194 | 8.115 | -2.462 | 1.40E-02 | 4.80E-02 | -3.558 |
| ELF5 | 0.269 | 5.563 | 2.461 | 1.40E-02 | 4.80E-02 | -3.559 |
| TLR4 | -0.315 | 7.927 | -2.459 | 1.50E-02 | 4.90E-02 | -3.566 |
| IL1RL2 | 0.284 | 5.424 | 2.458 | 1.50E-02 | 4.90E-02 | -3.567 |
| TPP2 | -0.183 | 8.198 | -2.457 | 1.50E-02 | 4.90E-02 | -3.571 |
| LAMC2 | 0.342 | 6.561 | 2.454 | 1.50E-02 | 4.90E-02 | -3.574 |
| GABRA1 | 0.24 | 4.94 | 2.453 | 1.50E-02 | 4.90E-02 | -3.578 |
| INPPL1 | -0.228 | 9.616 | -2.453 | 1.50E-02 | 4.90E-02 | -3.58 |
| GCM2 | 0.257 | 4.681 | 2.451 | 1.50E-02 | 5.00E-02 | -3.581 |
| HYAL2 | 0.344 | 9.529 | 2.451 | 1.50E-02 | 5.00E-02 | -3.582 |
| VRK3 | 0.159 | 8.312 | 2.451 | 1.50E-02 | 5.00E-02 | -3.583 |
| RAB38 | -0.276 | 7.987 | -2.451 | 1.50E-02 | 5.00E-02 | -3.585 |
| IRX4 | 0.374 | 5.567 | 2.449 | 1.50E-02 | 5.00E-02 | -3.587 |
| MDFI | 0.305 | 7.659 | 2.449 | 1.50E-02 | 5.00E-02 | -3.587 |
| EXTL2 | -0.267 | 8.229 | -2.449 | 1.50E-02 | 5.00E-02 | -3.588 |
| HMGCS2 | -0.451 | 6.113 | -2.448 | 1.50E-02 | 5.00E-02 | -3.59 |
| RENBP | 0.32 | 7.057 | 2.447 | 1.50E-02 | 5.00E-02 | -3.592 |
| CCL28 | 0.283 | 6.678 | 2.446 | 1.50E-02 | 5.00E-02 | -3.594 |
| SH3GL3 | 0.272 | 6.35 | 2.445 | 1.50E-02 | 5.00E-02 | -3.596 |
| NTRK3 | 0.217 | 5.511 | 2.445 | 1.50E-02 | 5.00E-02 | -3.596 |
| RFX2 | 0.258 | 6.942 | 2.443 | 1.50E-02 | 5.00E-02 | -3.6 |
| VASP | 0.292 | 9.63 | 2.442 | 1.50E-02 | 5.10E-02 | -3.604 |
| PCOLCE | 0.352 | 9.168 | 2.441 | 1.50E-02 | 5.10E-02 | -3.606 |
| QPCTL | 0.303 | 5.704 | 2.44 | 1.50E-02 | 5.10E-02 | -3.607 |
| TNFRSF12A | -0.304 | 10.551 | -2.441 | 1.50E-02 | 5.10E-02 | -3.609 |
| ZNF415 | -0.314 | 6.96 | -2.44 | 1.50E-02 | 5.10E-02 | -3.61 |
| GCNT3 | 0.278 | 5.165 | 2.439 | 1.50E-02 | 5.10E-02 | -3.611 |
| WDR4 | 0.275 | 7.305 | 2.438 | 1.50E-02 | 5.10E-02 | -3.612 |
| AP4B1 | 0.199 | 7.891 | 2.438 | 1.50E-02 | 5.10E-02 | -3.613 |
| CDK5 | 0.193 | 8.632 | 2.438 | 1.50E-02 | 5.10E-02 | -3.613 |
| MECP2 | 0.159 | 8.169 | 2.437 | 1.50E-02 | 5.10E-02 | -3.615 |
| ACTN1 | -0.267 | 11.383 | -2.437 | 1.50E-02 | 5.10E-02 | -3.617 |
| DAB2IP | 0.245 | 7.158 | 2.434 | 1.50E-02 | 5.10E-02 | -3.62 |
| RPLP2 | 0.17 | 14.386 | 2.434 | 1.60E-02 | 5.10E-02 | -3.622 |
| HSF4 | 0.259 | 6.747 | 2.433 | 1.60E-02 | 5.10E-02 | -3.624 |
| PTX3 | -0.6 | 7.3 | -2.434 | 1.60E-02 | 5.10E-02 | -3.624 |
| GEMIN4 | 0.221 | 8.728 | 2.433 | 1.60E-02 | 5.10E-02 | -3.625 |
| FXR1 | -0.239 | 8.724 | -2.432 | 1.60E-02 | 5.20E-02 | -3.629 |
| PFKFB3 | 0.274 | 8.171 | 2.426 | 1.60E-02 | 5.20E-02 | -3.64 |
| CPNE8 | -0.27 | 7.417 | -2.427 | 1.60E-02 | 5.20E-02 | -3.64 |
| CDSN | 0.444 | 6.213 | 2.424 | 1.60E-02 | 5.20E-02 | -3.644 |
| CACNA1S | 0.286 | 5.625 | 2.421 | 1.60E-02 | 5.30E-02 | -3.651 |
| MYH10 | 0.237 | 9.275 | 2.421 | 1.60E-02 | 5.30E-02 | -3.652 |
| SFRP2 | 0.355 | 5.672 | 2.42 | 1.60E-02 | 5.30E-02 | -3.653 |
| STK17B | -0.252 | 7.705 | -2.421 | 1.60E-02 | 5.30E-02 | -3.654 |
| DNAJC7 | -0.194 | 9.75 | -2.421 | 1.60E-02 | 5.30E-02 | -3.654 |
| ATXN1 | -0.228 | 7.665 | -2.42 | 1.60E-02 | 5.30E-02 | -3.656 |
| NUDT15 | -0.292 | 7.676 | -2.42 | 1.60E-02 | 5.30E-02 | -3.657 |
| SLC28A2 | 0.429 | 5.615 | 2.419 | 1.60E-02 | 5.30E-02 | -3.657 |
| ARHGAP21 | -0.21 | 8.857 | -2.419 | 1.60E-02 | 5.30E-02 | -3.658 |
| ZNF207 | -0.193 | 9.933 | -2.418 | 1.60E-02 | 5.30E-02 | -3.66 |
| BARD1 | -0.213 | 7.911 | -2.418 | 1.60E-02 | 5.30E-02 | -3.661 |
| RNF2 | -0.276 | 7.454 | -2.417 | 1.60E-02 | 5.30E-02 | -3.662 |
| VGLL4 | 0.178 | 8.842 | 2.416 | 1.60E-02 | 5.30E-02 | -3.663 |
| CDKL5 | 0.297 | 7.35 | 2.415 | 1.60E-02 | 5.30E-02 | -3.665 |
| WNT4 | 0.394 | 5.896 | 2.414 | 1.60E-02 | 5.30E-02 | -3.666 |
| CHIA | 0.346 | 5.807 | 2.414 | 1.60E-02 | 5.30E-02 | -3.667 |
| SLC41A1 | 0.243 | 7.114 | 2.414 | 1.60E-02 | 5.40E-02 | -3.668 |
| HBZ | 0.566 | 7.367 | 2.412 | 1.60E-02 | 5.40E-02 | -3.671 |
| CDC42BPG | 0.259 | 6.89 | 2.412 | 1.60E-02 | 5.40E-02 | -3.672 |
| PDK4 | -0.536 | 8.349 | -2.412 | 1.60E-02 | 5.40E-02 | -3.674 |
| UCP2 | 0.394 | 8.776 | 2.411 | 1.70E-02 | 5.40E-02 | -3.674 |
| UPB1 | 0.248 | 5.844 | 2.41 | 1.70E-02 | 5.40E-02 | -3.676 |
| NIT1 | -0.143 | 8.53 | -2.41 | 1.70E-02 | 5.40E-02 | -3.679 |
| ADCK4 | 0.305 | 6.563 | 2.409 | 1.70E-02 | 5.40E-02 | -3.68 |
| BCKDHB | -0.293 | 6.949 | -2.408 | 1.70E-02 | 5.40E-02 | -3.683 |
| LTC4S | 0.261 | 6.92 | 2.405 | 1.70E-02 | 5.50E-02 | -3.688 |
| GTPBP2 | 0.225 | 8.088 | 2.404 | 1.70E-02 | 5.50E-02 | -3.691 |
| SH2D4A | 0.369 | 7.002 | 2.402 | 1.70E-02 | 5.50E-02 | -3.694 |
| MAP4K4 | -0.199 | 8.847 | -2.403 | 1.70E-02 | 5.50E-02 | -3.695 |
| VSX1 | 0.221 | 5.542 | 2.401 | 1.70E-02 | 5.50E-02 | -3.696 |
| FGF21 | 0.287 | 5.017 | 2.401 | 1.70E-02 | 5.50E-02 | -3.697 |
| RANBP3 | -0.224 | 7.529 | -2.402 | 1.70E-02 | 5.50E-02 | -3.697 |
| IFNA4 | 0.296 | 5.647 | 2.401 | 1.70E-02 | 5.50E-02 | -3.697 |
| NGEF | 0.304 | 5.526 | 2.396 | 1.70E-02 | 5.60E-02 | -3.708 |
| TCEA3 | 0.274 | 8.02 | 2.395 | 1.70E-02 | 5.60E-02 | -3.71 |
| NRAP | 0.207 | 4.887 | 2.395 | 1.70E-02 | 5.60E-02 | -3.711 |
| GCN1L1 | -0.264 | 10.242 | -2.396 | 1.70E-02 | 5.60E-02 | -3.712 |
| C9orf43 | -0.291 | 5.162 | -2.394 | 1.70E-02 | 5.60E-02 | -3.716 |
| DMAP1 | 0.244 | 9.015 | 2.392 | 1.70E-02 | 5.60E-02 | -3.718 |
| PRKACB | -0.217 | 6.466 | -2.393 | 1.70E-02 | 5.60E-02 | -3.719 |
| SULF2 | -0.312 | 9.534 | -2.392 | 1.70E-02 | 5.60E-02 | -3.719 |
| LRIG3 | -0.27 | 7.152 | -2.392 | 1.70E-02 | 5.60E-02 | -3.72 |
| ZNF3 | 0.135 | 7.659 | 2.391 | 1.70E-02 | 5.60E-02 | -3.721 |
| POLR1D | 0.206 | 9.85 | 2.389 | 1.80E-02 | 5.60E-02 | -3.724 |
| MGLL | 0.245 | 9.881 | 2.389 | 1.80E-02 | 5.60E-02 | -3.725 |
| HORMAD1 | -0.458 | 5.542 | -2.39 | 1.80E-02 | 5.60E-02 | -3.725 |
| RER1 | -0.153 | 9.829 | -2.389 | 1.80E-02 | 5.70E-02 | -3.726 |
| ZNF624 | -0.307 | 6.255 | -2.389 | 1.80E-02 | 5.70E-02 | -3.727 |
| UBE4B | -0.212 | 9.02 | -2.388 | 1.80E-02 | 5.70E-02 | -3.729 |
| OR5L2 | 0.331 | 5.979 | 2.387 | 1.80E-02 | 5.70E-02 | -3.729 |
| GLTSCR2 | 0.212 | 12.041 | 2.384 | 1.80E-02 | 5.70E-02 | -3.735 |
| RPS17 | 0.229 | 12.79 | 2.383 | 1.80E-02 | 5.70E-02 | -3.738 |
| YTHDF3 | -0.329 | 8.436 | -2.384 | 1.80E-02 | 5.70E-02 | -3.738 |
| TRIM15 | 0.195 | 5.751 | 2.382 | 1.80E-02 | 5.70E-02 | -3.74 |
| NAPA | 0.276 | 8.603 | 2.382 | 1.80E-02 | 5.70E-02 | -3.74 |
| CACYBP | -0.181 | 8.143 | -2.382 | 1.80E-02 | 5.70E-02 | -3.742 |
| COPG2 | 0.266 | 7.55 | 2.38 | 1.80E-02 | 5.70E-02 | -3.744 |
| SCN10A | 0.242 | 4.894 | 2.378 | 1.80E-02 | 5.80E-02 | -3.75 |
| GALNT12 | -0.261 | 6.175 | -2.378 | 1.80E-02 | 5.80E-02 | -3.751 |
| TAX1BP1 | -0.257 | 11.554 | -2.376 | 1.80E-02 | 5.80E-02 | -3.757 |
| TRIM28 | 0.348 | 10.031 | 2.374 | 1.80E-02 | 5.80E-02 | -3.757 |
| CALML5 | -0.33 | 5.271 | -2.374 | 1.80E-02 | 5.80E-02 | -3.761 |
| SSBP2 | -0.278 | 8.539 | -2.373 | 1.80E-02 | 5.90E-02 | -3.763 |
| ERO1LB | -0.262 | 6.392 | -2.371 | 1.80E-02 | 5.90E-02 | -3.768 |
| PKLR | 0.289 | 6.233 | 2.369 | 1.80E-02 | 5.90E-02 | -3.769 |
| BUB1 | -0.344 | 6.991 | -2.369 | 1.90E-02 | 5.90E-02 | -3.772 |
| PRKCA | -0.257 | 8.088 | -2.369 | 1.90E-02 | 5.90E-02 | -3.773 |
| BANF1 | 0.203 | 8.846 | 2.368 | 1.90E-02 | 5.90E-02 | -3.773 |
| CYHR1 | 0.194 | 7.884 | 2.368 | 1.90E-02 | 5.90E-02 | -3.773 |
| FRZB | -0.401 | 8.865 | -2.368 | 1.90E-02 | 5.90E-02 | -3.774 |
| HOXD1 | 0.284 | 5.442 | 2.367 | 1.90E-02 | 5.90E-02 | -3.775 |
| DYSF | -0.315 | 10.01 | -2.367 | 1.90E-02 | 5.90E-02 | -3.777 |
| NR5A1 | 0.319 | 5.631 | 2.365 | 1.90E-02 | 5.90E-02 | -3.778 |
| PVRL2 | 0.377 | 9.367 | 2.364 | 1.90E-02 | 6.00E-02 | -3.781 |
| SLC1A7 | 0.209 | 6.491 | 2.363 | 1.90E-02 | 6.00E-02 | -3.782 |
| DSCR3 | -0.198 | 8.519 | -2.363 | 1.90E-02 | 6.00E-02 | -3.785 |
| LTK | 0.266 | 7.449 | 2.362 | 1.90E-02 | 6.00E-02 | -3.785 |
| DNAH9 | 0.313 | 5.176 | 2.36 | 1.90E-02 | 6.00E-02 | -3.789 |
| LSAMP | 0.362 | 5.485 | 2.36 | 1.90E-02 | 6.00E-02 | -3.79 |
| ANKRD1 | -0.273 | 5.522 | -2.36 | 1.90E-02 | 6.00E-02 | -3.792 |
| LAMB3 | 0.387 | 7.81 | 2.359 | 1.90E-02 | 6.00E-02 | -3.792 |
| MLF2 | 0.317 | 10.433 | 2.358 | 1.90E-02 | 6.00E-02 | -3.793 |
| PPP1R3F | 0.216 | 6.856 | 2.357 | 1.90E-02 | 6.00E-02 | -3.797 |
| PFDN4 | -0.349 | 8.139 | -2.356 | 1.90E-02 | 6.10E-02 | -3.801 |
| ZDHHC5 | 0.274 | 10.211 | 2.355 | 1.90E-02 | 6.10E-02 | -3.802 |
| ARSF | 0.337 | 5.378 | 2.355 | 1.90E-02 | 6.10E-02 | -3.802 |
| ALLC | 0.215 | 4.908 | 2.353 | 1.90E-02 | 6.10E-02 | -3.806 |
| SP7 | 0.247 | 4.988 | 2.352 | 1.90E-02 | 6.10E-02 | -3.808 |
| SF3A1 | -0.193 | 8.83 | -2.353 | 1.90E-02 | 6.10E-02 | -3.808 |
| POLD1 | 0.211 | 8.654 | 2.351 | 1.90E-02 | 6.10E-02 | -3.809 |
| STX6 | 0.246 | 8.234 | 2.351 | 1.90E-02 | 6.10E-02 | -3.81 |
| ADD2 | 0.219 | 5.32 | 2.351 | 1.90E-02 | 6.10E-02 | -3.81 |
| MYO18B | 0.279 | 4.94 | 2.351 | 1.90E-02 | 6.10E-02 | -3.81 |
| MKNK2 | -0.187 | 10.488 | -2.352 | 1.90E-02 | 6.10E-02 | -3.81 |
| FSCN1 | -0.245 | 10.647 | -2.349 | 2.00E-02 | 6.20E-02 | -3.818 |
| SLC25A13 | -0.202 | 8.481 | -2.349 | 2.00E-02 | 6.20E-02 | -3.818 |
| SLC25A23 | 0.219 | 8.308 | 2.347 | 2.00E-02 | 6.20E-02 | -3.819 |
| CLEC4A | -0.257 | 7.609 | -2.348 | 2.00E-02 | 6.20E-02 | -3.819 |
| FGF23 | -0.258 | 4.832 | -2.347 | 2.00E-02 | 6.20E-02 | -3.82 |
| RECQL5 | -0.219 | 6.111 | -2.347 | 2.00E-02 | 6.20E-02 | -3.822 |
| FGF20 | -0.234 | 5.229 | -2.347 | 2.00E-02 | 6.20E-02 | -3.822 |
| GABRA2 | 0.24 | 4.759 | 2.345 | 2.00E-02 | 6.20E-02 | -3.824 |
| IFIT1 | 0.461 | 9.32 | 2.343 | 2.00E-02 | 6.20E-02 | -3.827 |
| ESPN | 0.289 | 5.486 | 2.343 | 2.00E-02 | 6.20E-02 | -3.827 |
| LSM4 | 0.201 | 10.011 | 2.343 | 2.00E-02 | 6.20E-02 | -3.828 |
| OR51B5 | 0.249 | 5.288 | 2.342 | 2.00E-02 | 6.20E-02 | -3.831 |
| CRMP1 | 0.277 | 6.43 | 2.341 | 2.00E-02 | 6.20E-02 | -3.832 |
| RYR2 | 0.269 | 5.387 | 2.34 | 2.00E-02 | 6.20E-02 | -3.834 |
| MED8 | -0.157 | 8.979 | -2.34 | 2.00E-02 | 6.30E-02 | -3.837 |
| LGI3 | 0.271 | 6.244 | 2.339 | 2.00E-02 | 6.30E-02 | -3.837 |
| EFHC2 | -0.265 | 5.428 | -2.339 | 2.00E-02 | 6.30E-02 | -3.839 |
| C8orf4 | 0.409 | 8.373 | 2.337 | 2.00E-02 | 6.30E-02 | -3.841 |
| AK2 | 0.137 | 9.038 | 2.337 | 2.00E-02 | 6.30E-02 | -3.842 |
| SERPIND1 | 0.324 | 5.668 | 2.336 | 2.00E-02 | 6.30E-02 | -3.844 |
| HCRT | 0.258 | 8.131 | 2.335 | 2.00E-02 | 6.30E-02 | -3.847 |
| NOSTRIN | 0.263 | 7.665 | 2.334 | 2.00E-02 | 6.30E-02 | -3.847 |
| UTF1 | 0.295 | 7.707 | 2.334 | 2.00E-02 | 6.30E-02 | -3.848 |
| MTHFD1L | 0.247 | 7.882 | 2.333 | 2.00E-02 | 6.30E-02 | -3.851 |
| SCRN2 | 0.304 | 7.601 | 2.332 | 2.00E-02 | 6.30E-02 | -3.851 |
| FAU | 0.19 | 13.752 | 2.332 | 2.00E-02 | 6.30E-02 | -3.852 |
| TYSND1 | 0.17 | 7.672 | 2.332 | 2.00E-02 | 6.30E-02 | -3.852 |
| SLC17A7 | 0.32 | 7.302 | 2.332 | 2.00E-02 | 6.30E-02 | -3.852 |
| PDPK1 | -0.167 | 8.478 | -2.332 | 2.00E-02 | 6.40E-02 | -3.855 |
| SNN | -0.207 | 7.772 | -2.331 | 2.10E-02 | 6.40E-02 | -3.857 |
| HPS5 | -0.144 | 8.291 | -2.33 | 2.10E-02 | 6.40E-02 | -3.859 |
| DHRS7 | -0.227 | 9.862 | -2.329 | 2.10E-02 | 6.40E-02 | -3.86 |
| HYDIN | 0.206 | 4.853 | 2.328 | 2.10E-02 | 6.40E-02 | -3.861 |
| RFXAP | -0.284 | 7.1 | -2.329 | 2.10E-02 | 6.40E-02 | -3.862 |
| CYP2C8 | 0.211 | 5.192 | 2.327 | 2.10E-02 | 6.40E-02 | -3.863 |
| TEP1 | -0.213 | 7.321 | -2.327 | 2.10E-02 | 6.40E-02 | -3.865 |
| HCK | -0.257 | 8.301 | -2.326 | 2.10E-02 | 6.40E-02 | -3.867 |
| TMEM14B | -0.242 | 10.973 | -2.326 | 2.10E-02 | 6.40E-02 | -3.867 |
| SPTBN2 | 0.349 | 6.076 | 2.323 | 2.10E-02 | 6.50E-02 | -3.873 |
| T | 0.261 | 5.653 | 2.32 | 2.10E-02 | 6.50E-02 | -3.88 |
| ZNF92 | -0.23 | 7.487 | -2.32 | 2.10E-02 | 6.50E-02 | -3.881 |
| MMAA | -0.168 | 6.799 | -2.32 | 2.10E-02 | 6.50E-02 | -3.881 |
| GAL | 0.434 | 6.395 | 2.319 | 2.10E-02 | 6.50E-02 | -3.882 |
| SLC25A10 | 0.243 | 6.686 | 2.318 | 2.10E-02 | 6.50E-02 | -3.883 |
| GPRC5C | 0.27 | 6.791 | 2.317 | 2.10E-02 | 6.50E-02 | -3.885 |
| ST14 | 0.199 | 8.147 | 2.317 | 2.10E-02 | 6.50E-02 | -3.885 |
| BCL11B | -0.215 | 5.681 | -2.317 | 2.10E-02 | 6.50E-02 | -3.887 |
| SLC39A5 | 0.274 | 5.872 | 2.316 | 2.10E-02 | 6.50E-02 | -3.887 |
| RNF8 | -0.187 | 6.142 | -2.316 | 2.10E-02 | 6.60E-02 | -3.889 |
| PHGDH | 0.332 | 9.586 | 2.315 | 2.10E-02 | 6.60E-02 | -3.89 |
| COX7B | -0.26 | 10.859 | -2.315 | 2.10E-02 | 6.60E-02 | -3.893 |
| TIMD4 | 0.399 | 6.339 | 2.314 | 2.10E-02 | 6.60E-02 | -3.893 |
| TST | 0.233 | 10.882 | 2.313 | 2.10E-02 | 6.60E-02 | -3.893 |
| VTI1B | -0.264 | 10.358 | -2.314 | 2.10E-02 | 6.60E-02 | -3.894 |
| ANAPC5 | 0.151 | 10.783 | 2.313 | 2.10E-02 | 6.60E-02 | -3.894 |
| LSM7 | 0.234 | 11.09 | 2.312 | 2.20E-02 | 6.60E-02 | -3.896 |
| C18orf21 | -0.158 | 9.3 | -2.312 | 2.20E-02 | 6.60E-02 | -3.898 |
| PPM1E | 0.253 | 4.826 | 2.311 | 2.20E-02 | 6.60E-02 | -3.899 |
| FANCA | -0.229 | 7.29 | -2.31 | 2.20E-02 | 6.60E-02 | -3.902 |
| LPL | -0.537 | 7.947 | -2.31 | 2.20E-02 | 6.60E-02 | -3.902 |
| CSTA | 0.293 | 7.666 | 2.309 | 2.20E-02 | 6.60E-02 | -3.903 |
| GSTA4 | -0.23 | 9.215 | -2.308 | 2.20E-02 | 6.70E-02 | -3.907 |
| PIM2 | 0.365 | 8.188 | 2.307 | 2.20E-02 | 6.70E-02 | -3.907 |
| BCL6 | -0.382 | 9.539 | -2.308 | 2.20E-02 | 6.70E-02 | -3.908 |
| TDG | -0.185 | 9.701 | -2.306 | 2.20E-02 | 6.70E-02 | -3.911 |
| FBL | 0.159 | 11.715 | 2.305 | 2.20E-02 | 6.70E-02 | -3.912 |
| COG7 | 0.261 | 7.853 | 2.304 | 2.20E-02 | 6.70E-02 | -3.915 |
| CYR61 | 0.293 | 10.184 | 2.302 | 2.20E-02 | 6.70E-02 | -3.919 |
| MRPS18B | 0.151 | 9.405 | 2.302 | 2.20E-02 | 6.70E-02 | -3.919 |
| KIAA0125 | 0.281 | 5.799 | 2.3 | 2.20E-02 | 6.70E-02 | -3.922 |
| FBXO40 | 0.201 | 4.916 | 2.299 | 2.20E-02 | 6.80E-02 | -3.924 |
| PIGF | -0.184 | 8.723 | -2.3 | 2.20E-02 | 6.80E-02 | -3.925 |
| WHSC1 | -0.177 | 6.501 | -2.3 | 2.20E-02 | 6.80E-02 | -3.925 |
| ARHGAP20 | 0.288 | 5.598 | 2.298 | 2.20E-02 | 6.80E-02 | -3.927 |
| DUSP23 | 0.265 | 10.009 | 2.297 | 2.20E-02 | 6.80E-02 | -3.93 |
| SLC6A15 | 0.224 | 5.347 | 2.297 | 2.20E-02 | 6.80E-02 | -3.93 |
| PLOD3 | 0.257 | 10.268 | 2.295 | 2.30E-02 | 6.80E-02 | -3.934 |
| HTR1D | 0.237 | 5.798 | 2.295 | 2.30E-02 | 6.80E-02 | -3.935 |
| LAMA3 | 0.359 | 7.226 | 2.294 | 2.30E-02 | 6.80E-02 | -3.935 |
| EXOSC10 | 0.156 | 9.993 | 2.294 | 2.30E-02 | 6.80E-02 | -3.937 |
| PIGO | 0.221 | 8.544 | 2.293 | 2.30E-02 | 6.80E-02 | -3.937 |
| RCOR3 | -0.182 | 8.733 | -2.294 | 2.30E-02 | 6.80E-02 | -3.938 |
| B3GNT4 | 0.32 | 5.338 | 2.292 | 2.30E-02 | 6.90E-02 | -3.939 |
| ADAM7 | 0.29 | 5.152 | 2.292 | 2.30E-02 | 6.90E-02 | -3.94 |
| SAMHD1 | -0.251 | 7.482 | -2.293 | 2.30E-02 | 6.90E-02 | -3.94 |
| FXYD3 | 0.272 | 7.551 | 2.292 | 2.30E-02 | 6.90E-02 | -3.941 |
| EDA | 0.201 | 5.98 | 2.291 | 2.30E-02 | 6.90E-02 | -3.942 |
| SLC25A28 | 0.168 | 9.929 | 2.291 | 2.30E-02 | 6.90E-02 | -3.943 |
| GP9 | 0.243 | 7.653 | 2.29 | 2.30E-02 | 6.90E-02 | -3.944 |
| CD84 | 0.246 | 6.675 | 2.29 | 2.30E-02 | 6.90E-02 | -3.944 |
| COG4 | 0.194 | 8.404 | 2.29 | 2.30E-02 | 6.90E-02 | -3.944 |
| PIWIL2 | 0.257 | 5.418 | 2.288 | 2.30E-02 | 6.90E-02 | -3.948 |
| ST3GAL2 | 0.249 | 7.84 | 2.287 | 2.30E-02 | 6.90E-02 | -3.95 |
| PRDX2 | 0.262 | 9.554 | 2.287 | 2.30E-02 | 6.90E-02 | -3.95 |
| PRRG4 | -0.303 | 7.651 | -2.288 | 2.30E-02 | 6.90E-02 | -3.952 |
| SIDT1 | 0.281 | 6.156 | 2.286 | 2.30E-02 | 6.90E-02 | -3.952 |
| CLDN5 | 0.348 | 8.829 | 2.286 | 2.30E-02 | 6.90E-02 | -3.953 |
| BICC1 | 0.269 | 5.461 | 2.285 | 2.30E-02 | 6.90E-02 | -3.955 |
| IGFBP6 | 0.475 | 8.199 | 2.283 | 2.30E-02 | 7.00E-02 | -3.96 |
| TAS2R3 | 0.256 | 4.91 | 2.283 | 2.30E-02 | 7.00E-02 | -3.96 |
| PMM1 | 0.19 | 9.038 | 2.28 | 2.30E-02 | 7.00E-02 | -3.966 |
| S100A9 | -0.309 | 10.315 | -2.281 | 2.30E-02 | 7.00E-02 | -3.966 |
| MAK | -0.257 | 5.64 | -2.281 | 2.30E-02 | 7.00E-02 | -3.967 |
| THUMPD2 | -0.174 | 7.489 | -2.28 | 2.30E-02 | 7.00E-02 | -3.969 |
| TOMM40 | -0.216 | 8.887 | -2.279 | 2.40E-02 | 7.00E-02 | -3.972 |
| PHYHIP | 0.282 | 6.098 | 2.277 | 2.40E-02 | 7.10E-02 | -3.973 |
| CDC37 | -0.182 | 10.993 | -2.278 | 2.40E-02 | 7.10E-02 | -3.973 |
| KIAA1407 | 0.279 | 6.578 | 2.276 | 2.40E-02 | 7.10E-02 | -3.975 |
| RTN2 | 0.284 | 8.284 | 2.275 | 2.40E-02 | 7.10E-02 | -3.977 |
| ABCB11 | 0.222 | 4.795 | 2.274 | 2.40E-02 | 7.10E-02 | -3.978 |
| LHX4 | 0.328 | 4.977 | 2.273 | 2.40E-02 | 7.10E-02 | -3.981 |
| TGM2 | 0.57 | 9.943 | 2.272 | 2.40E-02 | 7.10E-02 | -3.983 |
| ARF5 | 0.195 | 10.741 | 2.272 | 2.40E-02 | 7.10E-02 | -3.984 |
| GPS2 | 0.165 | 9.899 | 2.271 | 2.40E-02 | 7.10E-02 | -3.985 |
| PRKAB1 | 0.333 | 8.235 | 2.27 | 2.40E-02 | 7.20E-02 | -3.988 |
| B2M | -0.233 | 13.945 | -2.27 | 2.40E-02 | 7.20E-02 | -3.99 |
| TMOD2 | -0.278 | 5.672 | -2.269 | 2.40E-02 | 7.20E-02 | -3.992 |
| GFRA1 | 0.213 | 6.115 | 2.267 | 2.40E-02 | 7.20E-02 | -3.995 |
| TAZ | 0.182 | 8.017 | 2.266 | 2.40E-02 | 7.20E-02 | -3.997 |
| APC | -0.169 | 6.483 | -2.266 | 2.40E-02 | 7.20E-02 | -3.998 |
| HSBP1 | 0.157 | 11.158 | 2.265 | 2.40E-02 | 7.20E-02 | -3.998 |
| CDC42BPB | 0.199 | 9.699 | 2.265 | 2.40E-02 | 7.20E-02 | -3.999 |
| MYF6 | -0.228 | 4.853 | -2.265 | 2.40E-02 | 7.20E-02 | -4 |
| OASL | 0.43 | 7.682 | 2.264 | 2.40E-02 | 7.20E-02 | -4 |
| TBC1D17 | 0.257 | 8.265 | 2.263 | 2.40E-02 | 7.20E-02 | -4.002 |
| NCR1 | 0.246 | 5.915 | 2.262 | 2.40E-02 | 7.30E-02 | -4.004 |
| MRPS9 | 0.198 | 9.083 | 2.262 | 2.50E-02 | 7.30E-02 | -4.006 |
| IL22RA2 | 0.329 | 5.833 | 2.257 | 2.50E-02 | 7.40E-02 | -4.016 |
| TMEM40 | 0.265 | 8.994 | 2.256 | 2.50E-02 | 7.40E-02 | -4.018 |
| ATP8A1 | -0.301 | 6.067 | -2.256 | 2.50E-02 | 7.40E-02 | -4.021 |
| APBB2 | -0.209 | 7.239 | -2.254 | 2.50E-02 | 7.40E-02 | -4.024 |
| IDH2 | 0.246 | 10.516 | 2.253 | 2.50E-02 | 7.40E-02 | -4.024 |
| RIBC2 | 0.344 | 5.814 | 2.252 | 2.50E-02 | 7.40E-02 | -4.026 |
| OTOS | 0.364 | 6.686 | 2.251 | 2.50E-02 | 7.50E-02 | -4.029 |
| TRIM11 | 0.175 | 8.615 | 2.249 | 2.50E-02 | 7.50E-02 | -4.032 |
| STAC3 | 0.232 | 6.147 | 2.249 | 2.50E-02 | 7.50E-02 | -4.034 |
| FNBP4 | -0.225 | 9.733 | -2.249 | 2.50E-02 | 7.50E-02 | -4.036 |
| OSBPL6 | 0.225 | 6.025 | 2.248 | 2.50E-02 | 7.50E-02 | -4.036 |
| RYR3 | 0.208 | 4.987 | 2.247 | 2.50E-02 | 7.50E-02 | -4.038 |
| GPX5 | 0.294 | 5.981 | 2.246 | 2.60E-02 | 7.50E-02 | -4.039 |
| CLSTN2 | 0.394 | 5.779 | 2.245 | 2.60E-02 | 7.50E-02 | -4.041 |
| POLR3A | 0.157 | 7.858 | 2.245 | 2.60E-02 | 7.50E-02 | -4.041 |
| EDEM1 | -0.194 | 9.062 | -2.244 | 2.60E-02 | 7.60E-02 | -4.045 |
| APEX1 | 0.182 | 10.062 | 2.243 | 2.60E-02 | 7.60E-02 | -4.046 |
| PELI1 | -0.266 | 8.869 | -2.244 | 2.60E-02 | 7.60E-02 | -4.047 |
| KCNJ14 | 0.219 | 6.595 | 2.243 | 2.60E-02 | 7.60E-02 | -4.047 |
| OR10H1 | 0.259 | 5.988 | 2.24 | 2.60E-02 | 7.60E-02 | -4.051 |
| CDT1 | 0.318 | 8.096 | 2.238 | 2.60E-02 | 7.60E-02 | -4.056 |
| NR2E3 | 0.245 | 4.954 | 2.238 | 2.60E-02 | 7.70E-02 | -4.057 |
| MYO10 | -0.226 | 8.238 | -2.237 | 2.60E-02 | 7.70E-02 | -4.06 |
| KIAA1586 | -0.269 | 6.039 | -2.237 | 2.60E-02 | 7.70E-02 | -4.061 |
| CPNE6 | 0.262 | 5.471 | 2.236 | 2.60E-02 | 7.70E-02 | -4.061 |
| ST3GAL3 | 0.244 | 6.678 | 2.236 | 2.60E-02 | 7.70E-02 | -4.062 |
| ARRB1 | 0.23 | 8.358 | 2.235 | 2.60E-02 | 7.70E-02 | -4.063 |
| SLC16A9 | 0.337 | 6.037 | 2.234 | 2.60E-02 | 7.70E-02 | -4.065 |
| GALNT6 | 0.336 | 7.66 | 2.234 | 2.60E-02 | 7.70E-02 | -4.066 |
| ZNF568 | -0.204 | 5.678 | -2.234 | 2.60E-02 | 7.70E-02 | -4.066 |
| CDC7 | -0.235 | 8.143 | -2.234 | 2.60E-02 | 7.70E-02 | -4.067 |
| PHF21B | -0.204 | 4.987 | -2.234 | 2.60E-02 | 7.70E-02 | -4.067 |
| POLR3GL | 0.174 | 9.935 | 2.233 | 2.60E-02 | 7.70E-02 | -4.067 |
| CPE | -0.369 | 6.507 | -2.234 | 2.60E-02 | 7.70E-02 | -4.068 |
| RBKS | 0.255 | 6.965 | 2.23 | 2.70E-02 | 7.80E-02 | -4.073 |
| MCEE | -0.202 | 8.839 | -2.231 | 2.70E-02 | 7.80E-02 | -4.073 |
| CNOT2 | 0.154 | 9.502 | 2.229 | 2.70E-02 | 7.80E-02 | -4.076 |
| SCG3 | 0.233 | 4.854 | 2.228 | 2.70E-02 | 7.80E-02 | -4.077 |
| SMUG1 | -0.189 | 8.82 | -2.229 | 2.70E-02 | 7.80E-02 | -4.079 |
| CYB5R2 | 0.29 | 7.934 | 2.228 | 2.70E-02 | 7.80E-02 | -4.079 |
| MEN1 | 0.266 | 7.281 | 2.227 | 2.70E-02 | 7.80E-02 | -4.079 |
| CA14 | 0.266 | 6.225 | 2.227 | 2.70E-02 | 7.80E-02 | -4.08 |
| ZNF573 | 0.206 | 8.393 | 2.227 | 2.70E-02 | 7.80E-02 | -4.08 |
| MFHAS1 | 0.219 | 7.175 | 2.226 | 2.70E-02 | 7.80E-02 | -4.082 |
| RPL14 | -0.27 | 12.126 | -2.227 | 2.70E-02 | 7.80E-02 | -4.082 |
| MET | -0.328 | 8.025 | -2.225 | 2.70E-02 | 7.80E-02 | -4.086 |
| FHL5 | 0.329 | 5.732 | 2.224 | 2.70E-02 | 7.80E-02 | -4.087 |
| EPHA7 | 0.169 | 4.774 | 2.222 | 2.70E-02 | 7.90E-02 | -4.09 |
| ANXA7 | -0.135 | 9.615 | -2.223 | 2.70E-02 | 7.90E-02 | -4.091 |
| TGFBR3 | -0.238 | 10.022 | -2.223 | 2.70E-02 | 7.90E-02 | -4.091 |
| STRN | -0.213 | 6.674 | -2.223 | 2.70E-02 | 7.90E-02 | -4.091 |
| PSMD4 | 0.166 | 10.648 | 2.221 | 2.70E-02 | 7.90E-02 | -4.092 |
| ST13 | -0.194 | 10.062 | -2.222 | 2.70E-02 | 7.90E-02 | -4.092 |
| FAM53C | 0.185 | 9.205 | 2.22 | 2.70E-02 | 7.90E-02 | -4.094 |
| FBXO31 | 0.245 | 7.749 | 2.219 | 2.70E-02 | 7.90E-02 | -4.096 |
| NPTXR | 0.294 | 5.767 | 2.219 | 2.70E-02 | 7.90E-02 | -4.097 |
| COL8A1 | 0.376 | 7.096 | 2.218 | 2.70E-02 | 7.90E-02 | -4.099 |
| LAIR2 | 0.56 | 7.187 | 2.217 | 2.70E-02 | 7.90E-02 | -4.1 |
| HRK | 0.28 | 6.151 | 2.217 | 2.70E-02 | 7.90E-02 | -4.101 |
| KMO | -0.3 | 7.995 | -2.218 | 2.70E-02 | 7.90E-02 | -4.101 |
| VEPH1 | -0.299 | 5.729 | -2.218 | 2.70E-02 | 7.90E-02 | -4.102 |
| ZNF80 | 0.229 | 5.138 | 2.216 | 2.80E-02 | 8.00E-02 | -4.103 |
| FBXW2 | -0.169 | 7.032 | -2.217 | 2.80E-02 | 8.00E-02 | -4.104 |
| EMP3 | 0.239 | 10.158 | 2.215 | 2.80E-02 | 8.00E-02 | -4.105 |
| CHI3L1 | -0.392 | 7.289 | -2.216 | 2.80E-02 | 8.00E-02 | -4.106 |
| CHAD | 0.285 | 5.854 | 2.214 | 2.80E-02 | 8.00E-02 | -4.107 |
| NKX2-2 | -0.237 | 4.62 | -2.215 | 2.80E-02 | 8.00E-02 | -4.107 |
| DBN1 | 0.277 | 8.611 | 2.212 | 2.80E-02 | 8.00E-02 | -4.111 |
| MKNK1 | -0.192 | 8.951 | -2.213 | 2.80E-02 | 8.00E-02 | -4.112 |
| TIGD7 | -0.289 | 6.53 | -2.212 | 2.80E-02 | 8.00E-02 | -4.115 |
| MCOLN3 | 0.3 | 6.064 | 2.209 | 2.80E-02 | 8.10E-02 | -4.118 |
| MRPL3 | -0.16 | 9.855 | -2.208 | 2.80E-02 | 8.10E-02 | -4.122 |
| ENPP1 | -0.294 | 8.207 | -2.207 | 2.80E-02 | 8.10E-02 | -4.124 |
| KIFC2 | 0.259 | 7.679 | 2.206 | 2.80E-02 | 8.10E-02 | -4.125 |
| SLC30A6 | -0.27 | 6.268 | -2.206 | 2.80E-02 | 8.10E-02 | -4.126 |
| GPR18 | -0.313 | 5.876 | -2.206 | 2.80E-02 | 8.10E-02 | -4.127 |
| PTPRD | 0.314 | 6.772 | 2.203 | 2.80E-02 | 8.20E-02 | -4.131 |
| STK11IP | 0.27 | 8.344 | 2.202 | 2.90E-02 | 8.20E-02 | -4.132 |
| RAPGEF6 | 0.178 | 7.868 | 2.202 | 2.90E-02 | 8.20E-02 | -4.134 |
| VILL | 0.199 | 7.16 | 2.2 | 2.90E-02 | 8.20E-02 | -4.137 |
| PTPRCAP | 0.225 | 7.551 | 2.2 | 2.90E-02 | 8.20E-02 | -4.138 |
| RAPGEF3 | 0.213 | 7.544 | 2.2 | 2.90E-02 | 8.20E-02 | -4.138 |
| ADH1C | 0.302 | 5.892 | 2.2 | 2.90E-02 | 8.20E-02 | -4.138 |
| LDHAL6B | 0.222 | 4.709 | 2.197 | 2.90E-02 | 8.30E-02 | -4.144 |
| GEMIN5 | 0.2 | 7.593 | 2.196 | 2.90E-02 | 8.30E-02 | -4.145 |
| TMEM17 | -0.293 | 6.184 | -2.197 | 2.90E-02 | 8.30E-02 | -4.145 |
| SYT11 | 0.243 | 7.564 | 2.196 | 2.90E-02 | 8.30E-02 | -4.146 |
| IL23A | -0.313 | 6.397 | -2.197 | 2.90E-02 | 8.30E-02 | -4.146 |
| RPUSD4 | -0.176 | 8.469 | -2.196 | 2.90E-02 | 8.30E-02 | -4.148 |
| AIM2 | 0.302 | 6.877 | 2.193 | 2.90E-02 | 8.30E-02 | -4.152 |
| IL2RB | -0.435 | 9.145 | -2.193 | 2.90E-02 | 8.30E-02 | -4.153 |
| GPC4 | 0.316 | 8.321 | 2.192 | 2.90E-02 | 8.30E-02 | -4.154 |
| PSKH1 | 0.208 | 8.007 | 2.192 | 2.90E-02 | 8.30E-02 | -4.155 |
| ZAK | -0.192 | 8.403 | -2.193 | 2.90E-02 | 8.30E-02 | -4.155 |
| ISOC1 | -0.197 | 9.516 | -2.192 | 2.90E-02 | 8.30E-02 | -4.156 |
| GHRHR | 0.242 | 5.85 | 2.19 | 2.90E-02 | 8.40E-02 | -4.157 |
| ACOX1 | -0.177 | 8.369 | -2.191 | 2.90E-02 | 8.40E-02 | -4.158 |
| MAD1L1 | 0.193 | 8.138 | 2.19 | 2.90E-02 | 8.40E-02 | -4.159 |
| TEX14 | -0.199 | 5.195 | -2.19 | 2.90E-02 | 8.40E-02 | -4.16 |
| ALDH7A1 | 0.203 | 8.723 | 2.188 | 3.00E-02 | 8.40E-02 | -4.163 |
| ADAR | -0.251 | 9.778 | -2.188 | 3.00E-02 | 8.40E-02 | -4.165 |
| HERC5 | 0.308 | 8.908 | 2.186 | 3.00E-02 | 8.40E-02 | -4.166 |
| SEC61B | -0.172 | 11.917 | -2.187 | 3.00E-02 | 8.40E-02 | -4.166 |
| DIO3 | -0.291 | 6.558 | -2.186 | 3.00E-02 | 8.40E-02 | -4.167 |
| AKR1C4 | 0.204 | 4.953 | 2.185 | 3.00E-02 | 8.40E-02 | -4.168 |
| HAGHL | 0.25 | 6.765 | 2.184 | 3.00E-02 | 8.50E-02 | -4.171 |
| WDR17 | 0.222 | 4.854 | 2.183 | 3.00E-02 | 8.50E-02 | -4.172 |
| DOT1L | 0.168 | 6.625 | 2.183 | 3.00E-02 | 8.50E-02 | -4.172 |
| IVL | -0.284 | 4.84 | -2.184 | 3.00E-02 | 8.50E-02 | -4.172 |
| SERPINF2 | 0.282 | 5.709 | 2.183 | 3.00E-02 | 8.50E-02 | -4.173 |
| PCP4 | 0.315 | 6.801 | 2.181 | 3.00E-02 | 8.50E-02 | -4.176 |
| MARVELD2 | -0.216 | 6.887 | -2.182 | 3.00E-02 | 8.50E-02 | -4.177 |
| PLA2G4C | 0.324 | 7.481 | 2.181 | 3.00E-02 | 8.50E-02 | -4.177 |
| ALPP | 0.414 | 11.878 | 2.18 | 3.00E-02 | 8.50E-02 | -4.179 |
| BICD2 | -0.21 | 8.286 | -2.18 | 3.00E-02 | 8.50E-02 | -4.18 |
| TRAF6 | -0.152 | 7.637 | -2.18 | 3.00E-02 | 8.50E-02 | -4.182 |
| NFE2 | 0.256 | 8.868 | 2.178 | 3.00E-02 | 8.50E-02 | -4.183 |
| UMOD | 0.269 | 7.21 | 2.178 | 3.00E-02 | 8.50E-02 | -4.183 |
| PIAS1 | 0.176 | 9.163 | 2.177 | 3.00E-02 | 8.60E-02 | -4.185 |
| ASH1L | -0.28 | 7.026 | -2.176 | 3.10E-02 | 8.60E-02 | -4.189 |
| MAP2K5 | 0.169 | 7.235 | 2.173 | 3.10E-02 | 8.60E-02 | -4.193 |
| GLCE | -0.294 | 8.337 | -2.174 | 3.10E-02 | 8.60E-02 | -4.194 |
| IER3 | 0.358 | 10.834 | 2.172 | 3.10E-02 | 8.70E-02 | -4.196 |
| DNAJA1 | -0.222 | 11.697 | -2.172 | 3.10E-02 | 8.70E-02 | -4.197 |
| GFM1 | -0.202 | 8.243 | -2.172 | 3.10E-02 | 8.70E-02 | -4.197 |
| LMX1A | 0.255 | 5.239 | 2.17 | 3.10E-02 | 8.70E-02 | -4.199 |
| WNT1 | 0.283 | 5.39 | 2.17 | 3.10E-02 | 8.70E-02 | -4.2 |
| GPRASP1 | -0.231 | 7.091 | -2.17 | 3.10E-02 | 8.70E-02 | -4.201 |
| USP12 | -0.236 | 7.114 | -2.17 | 3.10E-02 | 8.70E-02 | -4.201 |
| EIF4ENIF1 | -0.154 | 8.595 | -2.169 | 3.10E-02 | 8.70E-02 | -4.203 |
| AMOT | 0.233 | 7.776 | 2.168 | 3.10E-02 | 8.70E-02 | -4.204 |
| ANP32E | -0.185 | 7.488 | -2.169 | 3.10E-02 | 8.70E-02 | -4.204 |
| RPL15 | 0.152 | 12.132 | 2.167 | 3.10E-02 | 8.70E-02 | -4.205 |
| LOH12CR1 | -0.242 | 7.582 | -2.168 | 3.10E-02 | 8.70E-02 | -4.206 |
| RALBP1 | -0.181 | 9.865 | -2.167 | 3.10E-02 | 8.70E-02 | -4.208 |
| CKAP4 | -0.235 | 10.601 | -2.165 | 3.10E-02 | 8.80E-02 | -4.213 |
| TINF2 | 0.149 | 10.828 | 2.162 | 3.20E-02 | 8.80E-02 | -4.216 |
| SPIN3 | -0.201 | 6.056 | -2.163 | 3.20E-02 | 8.80E-02 | -4.216 |
| HTATIP2 | -0.224 | 9.002 | -2.162 | 3.20E-02 | 8.80E-02 | -4.218 |
| GCDH | 0.193 | 7.918 | 2.161 | 3.20E-02 | 8.80E-02 | -4.218 |
| DDX46 | -0.202 | 8.13 | -2.162 | 3.20E-02 | 8.80E-02 | -4.219 |
| ZNF281 | -0.19 | 9.748 | -2.161 | 3.20E-02 | 8.80E-02 | -4.22 |
| HOXD11 | -0.275 | 5.573 | -2.161 | 3.20E-02 | 8.80E-02 | -4.221 |
| ABCF3 | 0.221 | 8.69 | 2.16 | 3.20E-02 | 8.80E-02 | -4.221 |
| RRAGB | -0.194 | 6.853 | -2.161 | 3.20E-02 | 8.80E-02 | -4.221 |
| PDILT | 0.277 | 4.754 | 2.159 | 3.20E-02 | 8.80E-02 | -4.221 |
| FAF1 | 0.151 | 9.513 | 2.158 | 3.20E-02 | 8.90E-02 | -4.224 |
| PARD6A | 0.24 | 6.6 | 2.158 | 3.20E-02 | 8.90E-02 | -4.224 |
| C2 | 0.257 | 8.094 | 2.158 | 3.20E-02 | 8.90E-02 | -4.225 |
| KRTAP5-9 | 0.263 | 6.905 | 2.157 | 3.20E-02 | 8.90E-02 | -4.226 |
| TLL1 | -0.248 | 5.477 | -2.158 | 3.20E-02 | 8.90E-02 | -4.227 |
| GLB1L | 0.254 | 7.325 | 2.156 | 3.20E-02 | 8.90E-02 | -4.229 |
| FLCN | -0.172 | 7.279 | -2.155 | 3.20E-02 | 8.90E-02 | -4.232 |
| CALML4 | 0.213 | 7.578 | 2.154 | 3.20E-02 | 8.90E-02 | -4.233 |
| IGBP1 | 0.155 | 9.921 | 2.153 | 3.20E-02 | 9.00E-02 | -4.235 |
| MAPKBP1 | 0.179 | 7.588 | 2.151 | 3.20E-02 | 9.00E-02 | -4.239 |
| KLF14 | 0.25 | 6.816 | 2.15 | 3.20E-02 | 9.00E-02 | -4.241 |
| CLEC2B | -0.329 | 7.901 | -2.151 | 3.20E-02 | 9.00E-02 | -4.241 |
| RAB27A | -0.185 | 7.918 | -2.151 | 3.30E-02 | 9.00E-02 | -4.241 |
| GNL2 | -0.171 | 9.83 | -2.151 | 3.30E-02 | 9.00E-02 | -4.241 |
| SSTR2 | 0.213 | 5.681 | 2.148 | 3.30E-02 | 9.00E-02 | -4.244 |
| SLC2A6 | 0.27 | 7.982 | 2.148 | 3.30E-02 | 9.00E-02 | -4.245 |
| KRTAP8-1 | -0.277 | 5.342 | -2.148 | 3.30E-02 | 9.00E-02 | -4.246 |
| EGR1 | 0.471 | 9.524 | 2.146 | 3.30E-02 | 9.10E-02 | -4.248 |
| NR2F6 | 0.311 | 9.66 | 2.145 | 3.30E-02 | 9.10E-02 | -4.25 |
| NDUFS1 | -0.148 | 9.209 | -2.146 | 3.30E-02 | 9.10E-02 | -4.251 |
| VN1R1 | -0.323 | 5.178 | -2.146 | 3.30E-02 | 9.10E-02 | -4.252 |
| SCARB2 | 0.259 | 10.387 | 2.144 | 3.30E-02 | 9.10E-02 | -4.252 |
| LAMP1 | -0.281 | 12.253 | -2.145 | 3.30E-02 | 9.10E-02 | -4.253 |
| GGA1 | -0.19 | 8.113 | -2.144 | 3.30E-02 | 9.10E-02 | -4.254 |
| RALB | -0.204 | 10.361 | -2.144 | 3.30E-02 | 9.10E-02 | -4.255 |
| BHLHB9 | -0.244 | 6.277 | -2.144 | 3.30E-02 | 9.10E-02 | -4.255 |
| WFDC11 | 0.202 | 4.623 | 2.143 | 3.30E-02 | 9.10E-02 | -4.255 |
| KCTD17 | 0.276 | 8.194 | 2.143 | 3.30E-02 | 9.10E-02 | -4.255 |
| KIF4A | 0.295 | 6.741 | 2.142 | 3.30E-02 | 9.10E-02 | -4.257 |
| PAPOLG | -0.218 | 6.961 | -2.143 | 3.30E-02 | 9.10E-02 | -4.258 |
| TAF12 | 0.207 | 9.761 | 2.142 | 3.30E-02 | 9.10E-02 | -4.258 |
| BAMBI | -0.31 | 10.109 | -2.141 | 3.30E-02 | 9.20E-02 | -4.261 |
| MYL7 | 0.264 | 5.868 | 2.14 | 3.30E-02 | 9.20E-02 | -4.262 |
| PLEKHB1 | 0.238 | 5.803 | 2.14 | 3.30E-02 | 9.20E-02 | -4.262 |
| MMP26 | 0.26 | 4.775 | 2.139 | 3.30E-02 | 9.20E-02 | -4.264 |
| CIC | 0.187 | 9.176 | 2.138 | 3.30E-02 | 9.20E-02 | -4.265 |
| MRPL42 | -0.191 | 8.31 | -2.139 | 3.30E-02 | 9.20E-02 | -4.266 |
| NSDHL | 0.171 | 8.905 | 2.138 | 3.40E-02 | 9.20E-02 | -4.266 |
| PDE10A | -0.16 | 5.553 | -2.138 | 3.40E-02 | 9.20E-02 | -4.268 |
| EPHA8 | 0.199 | 5.341 | 2.137 | 3.40E-02 | 9.20E-02 | -4.268 |
| TUSC1 | -0.271 | 8.155 | -2.138 | 3.40E-02 | 9.20E-02 | -4.268 |
| IGSF1 | 0.259 | 5.444 | 2.137 | 3.40E-02 | 9.20E-02 | -4.268 |
| GCK | 0.237 | 6.208 | 2.136 | 3.40E-02 | 9.20E-02 | -4.27 |
| ATP6V0A1 | -0.242 | 10.339 | -2.136 | 3.40E-02 | 9.20E-02 | -4.271 |
| LAT | 0.187 | 7.927 | 2.134 | 3.40E-02 | 9.30E-02 | -4.274 |
| AKAP6 | 0.219 | 5.381 | 2.133 | 3.40E-02 | 9.30E-02 | -4.275 |
| GDNF | 0.194 | 5.659 | 2.133 | 3.40E-02 | 9.30E-02 | -4.276 |
| PEX6 | 0.346 | 8.245 | 2.132 | 3.40E-02 | 9.30E-02 | -4.277 |
| SFTPB | 0.193 | 5.286 | 2.132 | 3.40E-02 | 9.30E-02 | -4.278 |
| PTPRM | 0.207 | 9.16 | 2.131 | 3.40E-02 | 9.30E-02 | -4.28 |
| KDELC1 | -0.211 | 7.617 | -2.132 | 3.40E-02 | 9.30E-02 | -4.28 |
| CPLX2 | 0.224 | 5.904 | 2.13 | 3.40E-02 | 9.30E-02 | -4.281 |
| MADD | 0.163 | 8.717 | 2.13 | 3.40E-02 | 9.30E-02 | -4.281 |
| MPHOSPH10 | 0.205 | 9.255 | 2.13 | 3.40E-02 | 9.30E-02 | -4.282 |
| SMAD5 | -0.292 | 7.326 | -2.131 | 3.40E-02 | 9.30E-02 | -4.282 |
| SCRN3 | -0.164 | 7.077 | -2.128 | 3.40E-02 | 9.40E-02 | -4.287 |
| ENO1 | 0.181 | 12.091 | 2.127 | 3.40E-02 | 9.40E-02 | -4.287 |
| JAM2 | 0.234 | 9.048 | 2.126 | 3.40E-02 | 9.40E-02 | -4.289 |
| OR1D2 | 0.247 | 5.839 | 2.126 | 3.40E-02 | 9.40E-02 | -4.289 |
| RPE | -0.174 | 7.628 | -2.127 | 3.40E-02 | 9.40E-02 | -4.289 |
| GPR19 | 0.283 | 5.94 | 2.126 | 3.40E-02 | 9.40E-02 | -4.29 |
| CAPNS1 | 0.208 | 10.938 | 2.125 | 3.50E-02 | 9.40E-02 | -4.291 |
| KCNE3 | -0.25 | 7.184 | -2.126 | 3.50E-02 | 9.40E-02 | -4.291 |
| UPK3A | 0.233 | 5.509 | 2.124 | 3.50E-02 | 9.40E-02 | -4.294 |
| SMAD9 | -0.222 | 6.691 | -2.124 | 3.50E-02 | 9.40E-02 | -4.295 |
| CYP2W1 | 0.31 | 8.445 | 2.123 | 3.50E-02 | 9.40E-02 | -4.296 |
| PEX12 | -0.206 | 7.208 | -2.123 | 3.50E-02 | 9.40E-02 | -4.298 |
| GSTO1 | 0.245 | 11.956 | 2.122 | 3.50E-02 | 9.40E-02 | -4.299 |
| JTB | -0.127 | 11.689 | -2.122 | 3.50E-02 | 9.50E-02 | -4.3 |
| PDGFB | 0.333 | 7.469 | 2.12 | 3.50E-02 | 9.50E-02 | -4.302 |
| NDUFC1 | 0.171 | 11.177 | 2.12 | 3.50E-02 | 9.50E-02 | -4.302 |
| IFNA2 | 0.258 | 4.818 | 2.12 | 3.50E-02 | 9.50E-02 | -4.303 |
| PSKH2 | 0.227 | 5.393 | 2.118 | 3.50E-02 | 9.50E-02 | -4.305 |
| NODAL | 0.259 | 5.16 | 2.118 | 3.50E-02 | 9.50E-02 | -4.306 |
| KRTAP1-5 | 0.361 | 5.729 | 2.118 | 3.50E-02 | 9.50E-02 | -4.306 |
| RECK | -0.224 | 7.766 | -2.119 | 3.50E-02 | 9.50E-02 | -4.307 |
| PARVA | 0.189 | 8.423 | 2.116 | 3.50E-02 | 9.50E-02 | -4.31 |
| ASTN2 | 0.238 | 6.819 | 2.115 | 3.50E-02 | 9.50E-02 | -4.311 |
| MRPL1 | -0.207 | 8.4 | -2.116 | 3.50E-02 | 9.60E-02 | -4.313 |
| DPP8 | -0.153 | 9.146 | -2.116 | 3.50E-02 | 9.60E-02 | -4.313 |
| PARN | -0.142 | 8.501 | -2.116 | 3.50E-02 | 9.60E-02 | -4.313 |
| PTPRZ1 | 0.209 | 4.952 | 2.114 | 3.50E-02 | 9.60E-02 | -4.314 |
| BOLL | 0.174 | 5.156 | 2.114 | 3.60E-02 | 9.60E-02 | -4.314 |
| HTR3A | 0.272 | 5.806 | 2.113 | 3.60E-02 | 9.60E-02 | -4.316 |
| MRPL46 | 0.167 | 9.856 | 2.113 | 3.60E-02 | 9.60E-02 | -4.316 |
| LAMP2 | -0.181 | 10.422 | -2.114 | 3.60E-02 | 9.60E-02 | -4.317 |
| GNL1 | 0.261 | 8.06 | 2.112 | 3.60E-02 | 9.60E-02 | -4.317 |
| CUEDC1 | 0.234 | 7.801 | 2.112 | 3.60E-02 | 9.60E-02 | -4.318 |
| CHEK2 | -0.192 | 6.964 | -2.112 | 3.60E-02 | 9.60E-02 | -4.319 |
| GATA4 | 0.196 | 5.787 | 2.111 | 3.60E-02 | 9.60E-02 | -4.32 |
| LAP3 | -0.298 | 10.569 | -2.112 | 3.60E-02 | 9.60E-02 | -4.32 |
| GPR1 | -0.364 | 6.146 | -2.111 | 3.60E-02 | 9.60E-02 | -4.322 |
| HSPB1 | -0.241 | 13.415 | -2.11 | 3.60E-02 | 9.60E-02 | -4.323 |
| MED31 | -0.235 | 6.356 | -2.11 | 3.60E-02 | 9.60E-02 | -4.325 |
| KLRD1 | -0.257 | 6.635 | -2.11 | 3.60E-02 | 9.60E-02 | -4.325 |
| GPR135 | 0.188 | 6.58 | 2.109 | 3.60E-02 | 9.60E-02 | -4.325 |
| UXT | 0.169 | 10.441 | 2.108 | 3.60E-02 | 9.60E-02 | -4.326 |
| CNNM3 | 0.154 | 8.505 | 2.108 | 3.60E-02 | 9.70E-02 | -4.327 |
| CNKSR1 | 0.218 | 6.999 | 2.107 | 3.60E-02 | 9.70E-02 | -4.327 |
| RASEF | 0.291 | 6.105 | 2.107 | 3.60E-02 | 9.70E-02 | -4.328 |
| SSRP1 | 0.178 | 11.213 | 2.106 | 3.60E-02 | 9.70E-02 | -4.33 |
| LPAL2 | 0.186 | 4.862 | 2.106 | 3.60E-02 | 9.70E-02 | -4.331 |
| CITED2 | -0.29 | 9.816 | -2.106 | 3.60E-02 | 9.70E-02 | -4.332 |
| DUOX1 | 0.247 | 6.377 | 2.105 | 3.60E-02 | 9.70E-02 | -4.333 |
| RPS18 | -0.217 | 14.21 | -2.105 | 3.60E-02 | 9.70E-02 | -4.333 |
| PASD1 | 0.211 | 4.706 | 2.104 | 3.60E-02 | 9.70E-02 | -4.334 |
| HIST1H2AM | 0.234 | 7.922 | 2.104 | 3.60E-02 | 9.70E-02 | -4.335 |
| TIMM10 | 0.232 | 9.755 | 2.103 | 3.60E-02 | 9.70E-02 | -4.336 |
| STIM2 | -0.182 | 7.006 | -2.102 | 3.70E-02 | 9.80E-02 | -4.34 |
| ETV5 | 0.27 | 8.3 | 2.1 | 3.70E-02 | 9.80E-02 | -4.342 |
| NR0B1 | 0.185 | 4.864 | 2.1 | 3.70E-02 | 9.80E-02 | -4.343 |
| RBPMS | 0.171 | 7.536 | 2.099 | 3.70E-02 | 9.80E-02 | -4.343 |
| NEBL | -0.381 | 6.407 | -2.099 | 3.70E-02 | 9.80E-02 | -4.347 |
| RRAD | 0.363 | 7.743 | 2.098 | 3.70E-02 | 9.80E-02 | -4.347 |
| SCAMP3 | 0.155 | 10.213 | 2.097 | 3.70E-02 | 9.80E-02 | -4.347 |
| TTC17 | 0.23 | 7.909 | 2.097 | 3.70E-02 | 9.80E-02 | -4.348 |
| POLR2D | 0.17 | 8.222 | 2.097 | 3.70E-02 | 9.80E-02 | -4.348 |
| PIK3C2A | -0.352 | 7.748 | -2.097 | 3.70E-02 | 9.80E-02 | -4.35 |
| OR51E1 | 0.262 | 5.848 | 2.095 | 3.70E-02 | 9.90E-02 | -4.353 |
| PNMA3 | -0.247 | 5.617 | -2.096 | 3.70E-02 | 9.90E-02 | -4.353 |
| PTPRG | -0.184 | 6.629 | -2.096 | 3.70E-02 | 9.90E-02 | -4.353 |
| BBS7 | -0.234 | 5.961 | -2.094 | 3.70E-02 | 9.90E-02 | -4.356 |
| GRPEL2 | -0.214 | 8.898 | -2.094 | 3.70E-02 | 9.90E-02 | -4.356 |
| OR1Q1 | 0.244 | 4.735 | 2.093 | 3.70E-02 | 9.90E-02 | -4.357 |
| KCNN4 | 0.257 | 9.736 | 2.093 | 3.70E-02 | 9.90E-02 | -4.357 |
| CBR3 | 0.294 | 7.683 | 2.092 | 3.70E-02 | 9.90E-02 | -4.358 |
| PRSS16 | 0.227 | 6.784 | 2.091 | 3.80E-02 | 9.90E-02 | -4.361 |
| POU2F2 | 0.198 | 7.39 | 2.091 | 3.80E-02 | 9.90E-02 | -4.361 |
| CAPN13 | -0.273 | 5.192 | -2.091 | 3.80E-02 | 9.90E-02 | -4.362 |
| MEIS3 | 0.26 | 5.937 | 2.089 | 3.80E-02 | 1.00E-01 | -4.364 |
| EMP2 | -0.226 | 8.394 | -2.089 | 3.80E-02 | 1.00E-01 | -4.367 |
| CCPG1 | -0.205 | 7.913 | -2.089 | 3.80E-02 | 1.00E-01 | -4.367 |
| OGFR | 0.239 | 10.283 | 2.088 | 3.80E-02 | 1.00E-01 | -4.367 |
| MEF2A | -0.188 | 7.991 | -2.089 | 3.80E-02 | 1.00E-01 | -4.367 |
| FOXJ2 | 0.176 | 9.031 | 2.087 | 3.80E-02 | 1.00E-01 | -4.368 |
| SDF2 | 0.176 | 10.005 | 2.085 | 3.80E-02 | 1.01E-01 | -4.373 |
| PDCD1 | 0.228 | 7.491 | 2.084 | 3.80E-02 | 1.01E-01 | -4.374 |
| RRAGA | -0.137 | 11.89 | -2.085 | 3.80E-02 | 1.01E-01 | -4.375 |
| NR3C2 | -0.208 | 6.828 | -2.084 | 3.80E-02 | 1.01E-01 | -4.375 |
| PLOD1 | 0.233 | 9.664 | 2.083 | 3.80E-02 | 1.01E-01 | -4.377 |
| MAP4K2 | 0.196 | 9.383 | 2.082 | 3.80E-02 | 1.01E-01 | -4.378 |
| B3GALT2 | 0.227 | 5.148 | 2.08 | 3.90E-02 | 1.01E-01 | -4.382 |
| ZZEF1 | -0.166 | 8.77 | -2.08 | 3.90E-02 | 1.02E-01 | -4.384 |
| HIRIP3 | 0.174 | 7.81 | 2.079 | 3.90E-02 | 1.02E-01 | -4.385 |
| USP32 | -0.179 | 7.35 | -2.079 | 3.90E-02 | 1.02E-01 | -4.387 |
| TBX5 | -0.216 | 5.684 | -2.077 | 3.90E-02 | 1.02E-01 | -4.39 |
| VAMP1 | -0.273 | 5.843 | -2.077 | 3.90E-02 | 1.02E-01 | -4.39 |
| RPL3 | -0.21 | 13.887 | -2.077 | 3.90E-02 | 1.02E-01 | -4.391 |
| PODN | 0.257 | 5.706 | 2.076 | 3.90E-02 | 1.02E-01 | -4.391 |
| PLAG1 | -0.206 | 7.765 | -2.076 | 3.90E-02 | 1.02E-01 | -4.393 |
| ITGAM | -0.239 | 7.83 | -2.075 | 3.90E-02 | 1.03E-01 | -4.394 |
| TIMP3 | -0.376 | 9.832 | -2.073 | 3.90E-02 | 1.03E-01 | -4.398 |
| HES4 | 0.33 | 10.538 | 2.072 | 3.90E-02 | 1.03E-01 | -4.399 |
| MAGEA11 | 0.239 | 6.085 | 2.07 | 4.00E-02 | 1.03E-01 | -4.403 |
| SMAD2 | -0.132 | 7.794 | -2.07 | 4.00E-02 | 1.04E-01 | -4.404 |
| PGLYRP2 | 0.21 | 4.95 | 2.067 | 4.00E-02 | 1.04E-01 | -4.409 |
| PRDM2 | -0.114 | 7.693 | -2.067 | 4.00E-02 | 1.04E-01 | -4.411 |
| DDO | 0.244 | 5.694 | 2.065 | 4.00E-02 | 1.04E-01 | -4.412 |
| OSTF1 | -0.157 | 10.837 | -2.066 | 4.00E-02 | 1.04E-01 | -4.412 |
| DTX3L | -0.259 | 7.926 | -2.065 | 4.00E-02 | 1.05E-01 | -4.413 |
| IQCF1 | 0.187 | 4.855 | 2.064 | 4.00E-02 | 1.05E-01 | -4.413 |
| ATXN7L2 | 0.225 | 8.46 | 2.063 | 4.00E-02 | 1.05E-01 | -4.417 |
| UROD | 0.179 | 11.038 | 2.062 | 4.00E-02 | 1.05E-01 | -4.417 |
| MVP | 0.227 | 11.147 | 2.061 | 4.00E-02 | 1.05E-01 | -4.42 |
| HIST1H3G | 0.253 | 7.841 | 2.061 | 4.00E-02 | 1.05E-01 | -4.42 |
| OTX1 | 0.23 | 6.216 | 2.06 | 4.00E-02 | 1.06E-01 | -4.423 |
| ABCA3 | 0.257 | 7.522 | 2.059 | 4.10E-02 | 1.06E-01 | -4.424 |
| ZBTB24 | -0.185 | 7.912 | -2.06 | 4.10E-02 | 1.06E-01 | -4.424 |
| ADIPOR2 | -0.157 | 9.875 | -2.06 | 4.10E-02 | 1.06E-01 | -4.424 |
| CCNB3 | -0.241 | 5.209 | -2.06 | 4.10E-02 | 1.06E-01 | -4.425 |
| CAMTA1 | -0.206 | 6.571 | -2.059 | 4.10E-02 | 1.06E-01 | -4.427 |
| ATP1B2 | 0.287 | 7.404 | 2.057 | 4.10E-02 | 1.06E-01 | -4.427 |
| NRIP1 | -0.326 | 9.573 | -2.057 | 4.10E-02 | 1.06E-01 | -4.43 |
| RNASE1 | 0.221 | 10.289 | 2.054 | 4.10E-02 | 1.07E-01 | -4.434 |
| CDK8 | -0.254 | 7.291 | -2.054 | 4.10E-02 | 1.07E-01 | -4.436 |
| HMGA1 | 0.207 | 9.232 | 2.051 | 4.10E-02 | 1.07E-01 | -4.439 |
| ALPPL2 | 0.278 | 6.837 | 2.051 | 4.10E-02 | 1.07E-01 | -4.439 |
| HEPH | 0.257 | 6.24 | 2.051 | 4.10E-02 | 1.07E-01 | -4.439 |
| NES | 0.255 | 8.251 | 2.051 | 4.10E-02 | 1.07E-01 | -4.44 |
| SLC17A5 | -0.204 | 8.561 | -2.051 | 4.10E-02 | 1.07E-01 | -4.441 |
| FOXR1 | 0.185 | 5.012 | 2.049 | 4.20E-02 | 1.08E-01 | -4.444 |
| CCR10 | 0.341 | 7.2 | 2.048 | 4.20E-02 | 1.08E-01 | -4.445 |
| IQSEC2 | 0.161 | 7.935 | 2.048 | 4.20E-02 | 1.08E-01 | -4.446 |
| BRCA1 | -0.195 | 7.218 | -2.048 | 4.20E-02 | 1.08E-01 | -4.448 |
| ANAPC2 | 0.189 | 6.926 | 2.045 | 4.20E-02 | 1.08E-01 | -4.451 |
| DHX57 | -0.175 | 6.438 | -2.046 | 4.20E-02 | 1.08E-01 | -4.451 |
| PPP1R3B | -0.233 | 8.261 | -2.046 | 4.20E-02 | 1.08E-01 | -4.451 |
| FMOD | 0.275 | 8.267 | 2.042 | 4.20E-02 | 1.09E-01 | -4.457 |
| PTPRF | -0.264 | 9.579 | -2.043 | 4.20E-02 | 1.09E-01 | -4.458 |
| STAT2 | 0.197 | 9.78 | 2.041 | 4.20E-02 | 1.09E-01 | -4.459 |
| KIAA0141 | 0.126 | 8.878 | 2.04 | 4.20E-02 | 1.10E-01 | -4.461 |
| PYCR1 | 0.262 | 7.451 | 2.04 | 4.20E-02 | 1.10E-01 | -4.462 |
| MAP2K1 | -0.193 | 10.154 | -2.039 | 4.30E-02 | 1.10E-01 | -4.464 |
| HOXB13 | 0.222 | 6.079 | 2.037 | 4.30E-02 | 1.10E-01 | -4.467 |
| SPATA8 | 0.219 | 5.544 | 2.037 | 4.30E-02 | 1.10E-01 | -4.468 |
| AFM | -0.232 | 4.642 | -2.037 | 4.30E-02 | 1.10E-01 | -4.469 |
| ZSCAN1 | 0.265 | 6.447 | 2.036 | 4.30E-02 | 1.10E-01 | -4.47 |
| ZNF189 | -0.18 | 7.959 | -2.036 | 4.30E-02 | 1.11E-01 | -4.472 |
| DDX4 | 0.228 | 4.619 | 2.035 | 4.30E-02 | 1.11E-01 | -4.472 |
| APOC1 | 0.317 | 7.732 | 2.032 | 4.30E-02 | 1.11E-01 | -4.476 |
| CLDN17 | 0.223 | 5.137 | 2.032 | 4.30E-02 | 1.11E-01 | -4.476 |
| IFITM3 | 0.225 | 13.756 | 2.032 | 4.30E-02 | 1.11E-01 | -4.478 |
| FAM19A5 | -0.342 | 5.083 | -2.032 | 4.30E-02 | 1.11E-01 | -4.478 |
| POFUT2 | 0.165 | 7.513 | 2.03 | 4.30E-02 | 1.12E-01 | -4.48 |
| ALDOC | 0.332 | 7.407 | 2.03 | 4.30E-02 | 1.12E-01 | -4.481 |
| LIX1 | 0.286 | 5.294 | 2.027 | 4.40E-02 | 1.12E-01 | -4.486 |
| COMMD9 | 0.169 | 8.762 | 2.027 | 4.40E-02 | 1.12E-01 | -4.487 |
| SRGAP1 | 0.238 | 6.802 | 2.027 | 4.40E-02 | 1.12E-01 | -4.487 |
| SLCO3A1 | 0.194 | 6.628 | 2.026 | 4.40E-02 | 1.12E-01 | -4.489 |
| PLA2R1 | 0.234 | 5.649 | 2.025 | 4.40E-02 | 1.13E-01 | -4.49 |
| C1orf52 | -0.138 | 9.155 | -2.025 | 4.40E-02 | 1.13E-01 | -4.493 |
| CACNB3 | 0.231 | 7.584 | 2.023 | 4.40E-02 | 1.13E-01 | -4.495 |
| COIL | -0.144 | 8.728 | -2.023 | 4.40E-02 | 1.13E-01 | -4.496 |
| APLP2 | 0.187 | 10.298 | 2.02 | 4.50E-02 | 1.14E-01 | -4.501 |
| MANSC1 | -0.223 | 8.161 | -2.02 | 4.50E-02 | 1.14E-01 | -4.502 |
| ATP8B1 | -0.237 | 6.491 | -2.02 | 4.50E-02 | 1.14E-01 | -4.502 |
| ODF3L1 | 0.21 | 4.932 | 2.019 | 4.50E-02 | 1.14E-01 | -4.502 |
| KCNB2 | 0.246 | 4.9 | 2.019 | 4.50E-02 | 1.14E-01 | -4.503 |
| TTBK1 | 0.228 | 6.961 | 2.019 | 4.50E-02 | 1.14E-01 | -4.503 |
| GPAM | -0.194 | 6.398 | -2.019 | 4.50E-02 | 1.14E-01 | -4.503 |
| GK | -0.206 | 6.452 | -2.018 | 4.50E-02 | 1.14E-01 | -4.506 |
| OXCT1 | -0.266 | 7.388 | -2.018 | 4.50E-02 | 1.14E-01 | -4.506 |
| RPP14 | -0.173 | 7.935 | -2.016 | 4.50E-02 | 1.15E-01 | -4.509 |
| C4BPA | -0.428 | 6.18 | -2.015 | 4.50E-02 | 1.15E-01 | -4.512 |
| GPR171 | -0.346 | 6.715 | -2.015 | 4.50E-02 | 1.15E-01 | -4.513 |
| DNM1 | 0.262 | 6.792 | 2.013 | 4.50E-02 | 1.15E-01 | -4.514 |
| CST1 | 0.301 | 5.44 | 2.013 | 4.50E-02 | 1.15E-01 | -4.514 |
| DIRC2 | -0.207 | 10.394 | -2.013 | 4.50E-02 | 1.15E-01 | -4.515 |
| STRA6 | 0.224 | 9.006 | 2.012 | 4.50E-02 | 1.15E-01 | -4.516 |
| ITGB1 | -0.165 | 10.327 | -2.012 | 4.50E-02 | 1.16E-01 | -4.518 |
| ZNF471 | -0.185 | 6.206 | -2.011 | 4.60E-02 | 1.16E-01 | -4.519 |
| SSH3 | 0.199 | 8.014 | 2.009 | 4.60E-02 | 1.16E-01 | -4.522 |
| TNP1 | -0.201 | 4.905 | -2.01 | 4.60E-02 | 1.16E-01 | -4.522 |
| LIPG | -0.246 | 8.331 | -2.009 | 4.60E-02 | 1.16E-01 | -4.523 |
| EPB41L3 | -0.192 | 9.274 | -2.009 | 4.60E-02 | 1.16E-01 | -4.523 |
| TFB1M | -0.191 | 7.971 | -2.009 | 4.60E-02 | 1.16E-01 | -4.523 |
| SCNN1B | 0.378 | 6.975 | 2.007 | 4.60E-02 | 1.16E-01 | -4.524 |
| IDH1 | -0.198 | 10.231 | -2.008 | 4.60E-02 | 1.16E-01 | -4.524 |
| TFPI | -0.267 | 10.504 | -2.008 | 4.60E-02 | 1.16E-01 | -4.524 |
| NFX1 | -0.122 | 7.928 | -2.008 | 4.60E-02 | 1.16E-01 | -4.525 |
| RPP30 | -0.138 | 6.808 | -2.008 | 4.60E-02 | 1.16E-01 | -4.525 |
| UCHL5 | -0.148 | 8.13 | -2.007 | 4.60E-02 | 1.16E-01 | -4.527 |
| TNNI1 | 0.23 | 5.557 | 2.005 | 4.60E-02 | 1.17E-01 | -4.53 |
| SUPV3L1 | -0.158 | 8.35 | -2.005 | 4.60E-02 | 1.17E-01 | -4.531 |
| SYT14 | 0.219 | 4.789 | 2.004 | 4.60E-02 | 1.17E-01 | -4.532 |
| SLC6A17 | 0.299 | 5.96 | 2.003 | 4.60E-02 | 1.17E-01 | -4.533 |
| UPP2 | 0.234 | 4.961 | 2.002 | 4.60E-02 | 1.17E-01 | -4.535 |
| DHX35 | 0.164 | 7.991 | 2.001 | 4.60E-02 | 1.17E-01 | -4.536 |
| HIBCH | -0.2 | 8.847 | -2.002 | 4.60E-02 | 1.17E-01 | -4.536 |
| CRYGC | -0.19 | 4.668 | -2.002 | 4.70E-02 | 1.17E-01 | -4.537 |
| LZTR1 | 0.169 | 9.365 | 1.999 | 4.70E-02 | 1.18E-01 | -4.54 |
| B3GALT6 | 0.171 | 9.188 | 1.998 | 4.70E-02 | 1.18E-01 | -4.542 |
| RFT1 | -0.142 | 6.751 | -1.998 | 4.70E-02 | 1.18E-01 | -4.545 |
| TGM4 | 0.212 | 4.887 | 1.996 | 4.70E-02 | 1.19E-01 | -4.546 |
| AMHR2 | 0.231 | 4.781 | 1.996 | 4.70E-02 | 1.19E-01 | -4.547 |
| PRRG3 | 0.232 | 5.633 | 1.995 | 4.70E-02 | 1.19E-01 | -4.548 |
| ASPM | -0.284 | 7.729 | -1.995 | 4.70E-02 | 1.19E-01 | -4.549 |
| GREM2 | 0.3 | 6.974 | 1.994 | 4.70E-02 | 1.19E-01 | -4.55 |
| ARHGEF18 | -0.155 | 9.814 | -1.994 | 4.70E-02 | 1.19E-01 | -4.552 |
| GTF3C5 | 0.153 | 9.02 | 1.993 | 4.70E-02 | 1.19E-01 | -4.553 |
| CLIC6 | 0.284 | 5.225 | 1.993 | 4.70E-02 | 1.19E-01 | -4.553 |
| ISOC2 | 0.202 | 8.326 | 1.992 | 4.70E-02 | 1.19E-01 | -4.553 |
| LRP5 | 0.25 | 7.416 | 1.992 | 4.70E-02 | 1.19E-01 | -4.553 |
| CRABP1 | 0.323 | 6.754 | 1.991 | 4.80E-02 | 1.19E-01 | -4.555 |
| ZCCHC3 | 0.191 | 7.884 | 1.991 | 4.80E-02 | 1.20E-01 | -4.556 |
| PEMT | 0.216 | 8.121 | 1.991 | 4.80E-02 | 1.20E-01 | -4.557 |
| PER3 | -0.212 | 6.321 | -1.992 | 4.80E-02 | 1.20E-01 | -4.557 |
| TMCC3 | 0.205 | 8.114 | 1.989 | 4.80E-02 | 1.20E-01 | -4.559 |
| YPEL5 | -0.155 | 10.53 | -1.99 | 4.80E-02 | 1.20E-01 | -4.56 |
| CHRNA2 | 0.21 | 5.304 | 1.987 | 4.80E-02 | 1.20E-01 | -4.563 |
| CXCL16 | 0.21 | 7.796 | 1.987 | 4.80E-02 | 1.20E-01 | -4.563 |
| ARF1 | -0.177 | 10.599 | -1.988 | 4.80E-02 | 1.20E-01 | -4.563 |
| ECE1 | 0.265 | 7.972 | 1.987 | 4.80E-02 | 1.20E-01 | -4.564 |
| ECHS1 | 0.17 | 11.084 | 1.986 | 4.80E-02 | 1.20E-01 | -4.565 |
| RAB5C | 0.2 | 9.895 | 1.985 | 4.80E-02 | 1.21E-01 | -4.567 |
| PLA2G3 | 0.183 | 4.933 | 1.983 | 4.80E-02 | 1.21E-01 | -4.571 |
| KLK14 | 0.216 | 5.914 | 1.983 | 4.80E-02 | 1.21E-01 | -4.571 |
| SLIT1 | 0.138 | 5.22 | 1.982 | 4.90E-02 | 1.21E-01 | -4.572 |
| CALM1 | -0.193 | 11.177 | -1.983 | 4.90E-02 | 1.21E-01 | -4.572 |
| DOLPP1 | 0.237 | 8.342 | 1.982 | 4.90E-02 | 1.21E-01 | -4.573 |
| GAS2 | -0.221 | 5.188 | -1.983 | 4.90E-02 | 1.21E-01 | -4.573 |
| RPS15A | 0.172 | 13.395 | 1.98 | 4.90E-02 | 1.22E-01 | -4.576 |
| CRIM1 | -0.228 | 8.673 | -1.98 | 4.90E-02 | 1.22E-01 | -4.578 |
| LENG1 | 0.212 | 6.954 | 1.978 | 4.90E-02 | 1.22E-01 | -4.58 |
| C9orf3 | -0.171 | 7.452 | -1.979 | 4.90E-02 | 1.22E-01 | -4.582 |
| GRIN1 | 0.209 | 6.846 | 1.977 | 4.90E-02 | 1.23E-01 | -4.583 |
| DOCK7 | -0.186 | 7.637 | -1.977 | 4.90E-02 | 1.23E-01 | -4.584 |
| PRX | 0.186 | 6.348 | 1.976 | 4.90E-02 | 1.23E-01 | -4.584 |
| AP4S1 | -0.296 | 6.649 | -1.977 | 4.90E-02 | 1.23E-01 | -4.584 |
| CDH13 | -0.257 | 6.122 | -1.977 | 4.90E-02 | 1.23E-01 | -4.585 |
| CD200 | -0.307 | 8.367 | -1.976 | 4.90E-02 | 1.23E-01 | -4.586 |
| SSFA2 | -0.33 | 8.393 | -1.975 | 5.00E-02 | 1.23E-01 | -4.589 |
| LTB4R2 | 0.179 | 6.451 | 1.974 | 5.00E-02 | 1.23E-01 | -4.589 |
| BTBD8 | 0.195 | 4.742 | 1.971 | 5.00E-02 | 1.24E-01 | -4.593 |
| MARVELD3 | 0.208 | 6.023 | 1.971 | 5.00E-02 | 1.24E-01 | -4.593 |
| NMNAT2 | -0.246 | 6.004 | -1.972 | 5.00E-02 | 1.24E-01 | -4.595 |
| FTSJ1 | 0.171 | 8.353 | 1.97 | 5.00E-02 | 1.24E-01 | -4.596 |
| CRYL1 | 0.179 | 8.547 | 1.969 | 5.00E-02 | 1.24E-01 | -4.597 |
| ANKRD7 | 0.247 | 4.94 | 1.968 | 5.00E-02 | 1.25E-01 | -4.6 |
| ATPIF1 | -0.177 | 11.111 | -1.967 | 5.00E-02 | 1.25E-01 | -4.603 |
| PXMP2 | 0.16 | 7.763 | 1.966 | 5.00E-02 | 1.25E-01 | -4.604 |
| PSMA4 | -0.178 | 10.859 | -1.964 | 5.10E-02 | 1.26E-01 | -4.609 |
| PRKAB2 | -0.236 | 7.516 | -1.964 | 5.10E-02 | 1.26E-01 | -4.609 |
| RPL23A | -0.172 | 11.643 | -1.964 | 5.10E-02 | 1.26E-01 | -4.61 |
| SSTR3 | 0.276 | 6.981 | 1.963 | 5.10E-02 | 1.26E-01 | -4.61 |
| FGF10 | 0.257 | 5.351 | 1.961 | 5.10E-02 | 1.26E-01 | -4.614 |
| SOX3 | 0.248 | 5.893 | 1.959 | 5.10E-02 | 1.27E-01 | -4.616 |
| SLU7 | -0.15 | 9.422 | -1.959 | 5.10E-02 | 1.27E-01 | -4.618 |
| ATP5O | -0.16 | 12.514 | -1.959 | 5.10E-02 | 1.27E-01 | -4.619 |
| ADORA3 | 0.276 | 6.38 | 1.957 | 5.10E-02 | 1.27E-01 | -4.62 |
| KIF7 | 0.229 | 5.974 | 1.957 | 5.20E-02 | 1.27E-01 | -4.621 |
| FOS | -0.442 | 10.614 | -1.956 | 5.20E-02 | 1.28E-01 | -4.624 |
| TOLLIP | 0.179 | 8.134 | 1.955 | 5.20E-02 | 1.28E-01 | -4.624 |
| SRPX2 | 0.239 | 8.23 | 1.955 | 5.20E-02 | 1.28E-01 | -4.624 |
| MX1 | 0.393 | 10.944 | 1.955 | 5.20E-02 | 1.28E-01 | -4.625 |
| EIF1AY | -0.731 | 6.991 | -1.955 | 5.20E-02 | 1.28E-01 | -4.625 |
| EPS8L1 | 0.223 | 8.951 | 1.953 | 5.20E-02 | 1.28E-01 | -4.628 |
| SHC1 | 0.151 | 9.625 | 1.951 | 5.20E-02 | 1.29E-01 | -4.631 |
| PPIL4 | -0.15 | 7.48 | -1.952 | 5.20E-02 | 1.29E-01 | -4.632 |
| FOXA3 | 0.218 | 5.531 | 1.95 | 5.20E-02 | 1.29E-01 | -4.633 |
| TBL1XR1 | -0.205 | 9.045 | -1.951 | 5.20E-02 | 1.29E-01 | -4.634 |
| MED12L | -0.265 | 5.583 | -1.95 | 5.20E-02 | 1.29E-01 | -4.635 |
| TUBGCP2 | 0.166 | 8.838 | 1.949 | 5.20E-02 | 1.29E-01 | -4.636 |
| SIPA1L1 | 0.212 | 9.369 | 1.948 | 5.30E-02 | 1.29E-01 | -4.636 |
| PDE1C | 0.211 | 5.55 | 1.947 | 5.30E-02 | 1.30E-01 | -4.64 |
| HORMAD2 | 0.248 | 5.178 | 1.946 | 5.30E-02 | 1.30E-01 | -4.64 |
| PDZRN3 | -0.186 | 6.548 | -1.946 | 5.30E-02 | 1.30E-01 | -4.642 |
| CST7 | 0.322 | 6.663 | 1.945 | 5.30E-02 | 1.30E-01 | -4.642 |
| RBX1 | -0.203 | 11.362 | -1.946 | 5.30E-02 | 1.30E-01 | -4.643 |
| TFAP2A | -0.253 | 7.724 | -1.945 | 5.30E-02 | 1.30E-01 | -4.644 |
| PCK2 | 0.193 | 7.777 | 1.944 | 5.30E-02 | 1.30E-01 | -4.645 |
| CSTF2 | 0.149 | 8.95 | 1.944 | 5.30E-02 | 1.30E-01 | -4.646 |
| IL9 | 0.185 | 4.825 | 1.943 | 5.30E-02 | 1.30E-01 | -4.646 |
| KCND3 | 0.163 | 5.82 | 1.943 | 5.30E-02 | 1.30E-01 | -4.647 |
| RAPGEFL1 | -0.232 | 6.078 | -1.944 | 5.30E-02 | 1.30E-01 | -4.647 |
| RTN4 | -0.133 | 11.071 | -1.943 | 5.30E-02 | 1.30E-01 | -4.648 |
| LOXL4 | -0.274 | 6.805 | -1.942 | 5.30E-02 | 1.31E-01 | -4.65 |
| NMT2 | -0.219 | 7.815 | -1.941 | 5.40E-02 | 1.31E-01 | -4.652 |
| FTHL17 | -0.287 | 8.207 | -1.941 | 5.40E-02 | 1.31E-01 | -4.653 |
| BIRC6 | -0.169 | 8.315 | -1.94 | 5.40E-02 | 1.31E-01 | -4.654 |
| ABCG1 | -0.223 | 8.185 | -1.939 | 5.40E-02 | 1.31E-01 | -4.656 |
| MIPEP | 0.192 | 7.747 | 1.938 | 5.40E-02 | 1.31E-01 | -4.656 |
| CASP5 | -0.16 | 8.722 | -1.938 | 5.40E-02 | 1.32E-01 | -4.659 |
| HECTD2 | -0.227 | 6.09 | -1.937 | 5.40E-02 | 1.32E-01 | -4.659 |
| MARK4 | 0.164 | 6.275 | 1.935 | 5.40E-02 | 1.32E-01 | -4.661 |
| ADRA1B | 0.276 | 5.556 | 1.935 | 5.40E-02 | 1.32E-01 | -4.661 |
| IFI44L | 0.46 | 8.28 | 1.935 | 5.40E-02 | 1.32E-01 | -4.661 |
| FXR2 | -0.241 | 7.707 | -1.935 | 5.40E-02 | 1.32E-01 | -4.663 |
| PIK3C2G | 0.157 | 4.671 | 1.933 | 5.40E-02 | 1.32E-01 | -4.665 |
| SEMA6D | 0.191 | 6.174 | 1.932 | 5.50E-02 | 1.33E-01 | -4.667 |
| MRPL30 | -0.133 | 8.434 | -1.933 | 5.50E-02 | 1.33E-01 | -4.668 |
| SNRPE | -0.182 | 8.931 | -1.932 | 5.50E-02 | 1.33E-01 | -4.67 |
| HIPK4 | 0.233 | 5.302 | 1.929 | 5.50E-02 | 1.33E-01 | -4.672 |
| LTA | 0.218 | 6.16 | 1.929 | 5.50E-02 | 1.33E-01 | -4.673 |
| HSD17B1 | 0.318 | 10.728 | 1.928 | 5.50E-02 | 1.34E-01 | -4.675 |
| EPSTI1 | -0.318 | 8.551 | -1.928 | 5.50E-02 | 1.34E-01 | -4.676 |
| OMD | 0.406 | 5.287 | 1.927 | 5.50E-02 | 1.34E-01 | -4.677 |
| SLC9A6 | -0.202 | 7.404 | -1.925 | 5.60E-02 | 1.35E-01 | -4.682 |
| PPP1R13B | 0.196 | 7.425 | 1.924 | 5.60E-02 | 1.35E-01 | -4.683 |
| SPP2 | 0.226 | 4.954 | 1.923 | 5.60E-02 | 1.35E-01 | -4.684 |
| IL4I1 | 0.316 | 7.302 | 1.921 | 5.60E-02 | 1.35E-01 | -4.687 |
| SPTAN1 | 0.214 | 10.261 | 1.921 | 5.60E-02 | 1.35E-01 | -4.687 |
| VIPR1 | 0.275 | 7.026 | 1.92 | 5.60E-02 | 1.36E-01 | -4.689 |
| AKT2 | 0.197 | 7.142 | 1.92 | 5.60E-02 | 1.36E-01 | -4.69 |
| ASNS | 0.238 | 9.11 | 1.919 | 5.60E-02 | 1.36E-01 | -4.691 |
| GYPE | 0.262 | 6.408 | 1.918 | 5.60E-02 | 1.36E-01 | -4.692 |
| MGAT5B | 0.165 | 5.635 | 1.916 | 5.70E-02 | 1.37E-01 | -4.697 |
| TGFBR2 | -0.189 | 8.67 | -1.917 | 5.70E-02 | 1.37E-01 | -4.697 |
| ELAVL3 | 0.196 | 5.32 | 1.916 | 5.70E-02 | 1.37E-01 | -4.697 |
| NOV | -0.267 | 6.628 | -1.915 | 5.70E-02 | 1.37E-01 | -4.701 |
| OR2C3 | -0.18 | 4.84 | -1.914 | 5.70E-02 | 1.38E-01 | -4.702 |
| PRKAA2 | 0.194 | 5.125 | 1.913 | 5.70E-02 | 1.38E-01 | -4.702 |
| CADPS | 0.192 | 4.894 | 1.913 | 5.70E-02 | 1.38E-01 | -4.702 |
| HLA-DOA | 0.224 | 6.466 | 1.911 | 5.70E-02 | 1.38E-01 | -4.705 |
| CIRBP | -0.143 | 10.933 | -1.912 | 5.70E-02 | 1.38E-01 | -4.705 |
| CLASP2 | -0.23 | 7.186 | -1.912 | 5.70E-02 | 1.38E-01 | -4.705 |
| PDE6G | 0.24 | 6.823 | 1.911 | 5.70E-02 | 1.38E-01 | -4.707 |
| TRPM3 | 0.172 | 6.219 | 1.91 | 5.70E-02 | 1.38E-01 | -4.707 |
| FOXQ1 | 0.28 | 7.879 | 1.91 | 5.70E-02 | 1.38E-01 | -4.707 |
| PLA2G7 | -0.398 | 7.884 | -1.911 | 5.70E-02 | 1.38E-01 | -4.707 |
| CASP14 | 0.206 | 5.136 | 1.909 | 5.70E-02 | 1.38E-01 | -4.71 |
| IGF1R | -0.162 | 7.965 | -1.909 | 5.80E-02 | 1.38E-01 | -4.711 |
| USP10 | 0.143 | 9.234 | 1.908 | 5.80E-02 | 1.38E-01 | -4.711 |
| POU1F1 | 0.191 | 4.684 | 1.908 | 5.80E-02 | 1.38E-01 | -4.711 |
| USP11 | 0.182 | 9.123 | 1.907 | 5.80E-02 | 1.39E-01 | -4.714 |
| ASCL2 | 0.362 | 8.072 | 1.906 | 5.80E-02 | 1.39E-01 | -4.715 |
| KAAG1 | 0.253 | 5.333 | 1.906 | 5.80E-02 | 1.39E-01 | -4.715 |
| RAI1 | 0.176 | 7.185 | 1.906 | 5.80E-02 | 1.39E-01 | -4.715 |
| TRIP6 | 0.177 | 10.537 | 1.905 | 5.80E-02 | 1.39E-01 | -4.716 |
| BLOC1S2 | -0.15 | 8.734 | -1.906 | 5.80E-02 | 1.39E-01 | -4.717 |
| SLC34A1 | 0.244 | 5.903 | 1.904 | 5.80E-02 | 1.39E-01 | -4.718 |
| NEFH | 0.261 | 6.641 | 1.9 | 5.90E-02 | 1.41E-01 | -4.726 |
| CTSG | 0.426 | 7.311 | 1.9 | 5.90E-02 | 1.41E-01 | -4.726 |
| AK3 | 0.169 | 9.308 | 1.9 | 5.90E-02 | 1.41E-01 | -4.726 |
| AKAP8L | 0.152 | 9.293 | 1.899 | 5.90E-02 | 1.41E-01 | -4.727 |
| PPP1R9B | 0.201 | 7.6 | 1.899 | 5.90E-02 | 1.41E-01 | -4.728 |
| CHST6 | 0.258 | 7.892 | 1.897 | 5.90E-02 | 1.41E-01 | -4.731 |
| RP1 | 0.201 | 4.786 | 1.897 | 5.90E-02 | 1.41E-01 | -4.731 |
| RBM10 | 0.187 | 9.567 | 1.896 | 5.90E-02 | 1.42E-01 | -4.733 |
| SPINK5 | -0.225 | 6.003 | -1.896 | 5.90E-02 | 1.42E-01 | -4.735 |
| SLC12A3 | 0.231 | 5.501 | 1.894 | 5.90E-02 | 1.42E-01 | -4.736 |
| STAB1 | 0.237 | 8.838 | 1.894 | 5.90E-02 | 1.42E-01 | -4.737 |
| ILK | 0.13 | 10.362 | 1.894 | 5.90E-02 | 1.42E-01 | -4.737 |
| FSCN2 | 0.29 | 5.539 | 1.894 | 5.90E-02 | 1.42E-01 | -4.737 |
| LAMP3 | 0.293 | 6.789 | 1.893 | 6.00E-02 | 1.42E-01 | -4.739 |
| HRC | 0.291 | 6.527 | 1.892 | 6.00E-02 | 1.43E-01 | -4.741 |
| TLR10 | -0.204 | 5.311 | -1.892 | 6.00E-02 | 1.43E-01 | -4.742 |
| SEZ6 | 0.234 | 5.556 | 1.891 | 6.00E-02 | 1.43E-01 | -4.742 |
| SIGLEC11 | -0.256 | 6.035 | -1.892 | 6.00E-02 | 1.43E-01 | -4.742 |
| TRIM5 | -0.188 | 7.342 | -1.89 | 6.00E-02 | 1.43E-01 | -4.746 |
| NEK8 | 0.196 | 6.28 | 1.889 | 6.00E-02 | 1.43E-01 | -4.746 |
| TSG101 | 0.165 | 10.973 | 1.889 | 6.00E-02 | 1.43E-01 | -4.746 |
| CLU | -0.262 | 8.794 | -1.89 | 6.00E-02 | 1.43E-01 | -4.746 |
| ITPKB | 0.175 | 8.036 | 1.889 | 6.00E-02 | 1.43E-01 | -4.746 |
| LRRTM4 | -0.199 | 4.8 | -1.889 | 6.00E-02 | 1.43E-01 | -4.748 |
| MYO1F | 0.165 | 7.867 | 1.888 | 6.00E-02 | 1.43E-01 | -4.749 |
| MAP4K5 | -0.208 | 8.352 | -1.888 | 6.00E-02 | 1.43E-01 | -4.749 |
| LRRC20 | 0.242 | 6.874 | 1.887 | 6.00E-02 | 1.44E-01 | -4.75 |
| ADAM15 | 0.207 | 9.107 | 1.887 | 6.00E-02 | 1.44E-01 | -4.751 |
| KIAA1328 | 0.199 | 6.294 | 1.886 | 6.10E-02 | 1.44E-01 | -4.752 |
| MAN2A1 | 0.194 | 9.601 | 1.885 | 6.10E-02 | 1.44E-01 | -4.753 |
| TIGD5 | 0.153 | 8.364 | 1.885 | 6.10E-02 | 1.44E-01 | -4.754 |
| SDC2 | -0.239 | 7.258 | -1.885 | 6.10E-02 | 1.44E-01 | -4.754 |
| TXK | -0.318 | 7.886 | -1.885 | 6.10E-02 | 1.44E-01 | -4.755 |
| GSK3A | 0.168 | 9.127 | 1.884 | 6.10E-02 | 1.44E-01 | -4.756 |
| POLR3B | 0.142 | 8.893 | 1.883 | 6.10E-02 | 1.44E-01 | -4.756 |
| OR1F1 | -0.207 | 5.275 | -1.882 | 6.10E-02 | 1.45E-01 | -4.76 |
| SULT1E1 | -0.233 | 5.22 | -1.881 | 6.10E-02 | 1.45E-01 | -4.762 |
| TMPRSS5 | 0.23 | 5.956 | 1.879 | 6.10E-02 | 1.45E-01 | -4.763 |
| IL3 | 0.238 | 5.06 | 1.879 | 6.20E-02 | 1.46E-01 | -4.765 |
| CRYGN | 0.279 | 5.61 | 1.877 | 6.20E-02 | 1.46E-01 | -4.768 |
| ALPK3 | -0.227 | 6.116 | -1.878 | 6.20E-02 | 1.46E-01 | -4.768 |
| PMF1 | 0.153 | 9.093 | 1.875 | 6.20E-02 | 1.47E-01 | -4.771 |
| IL12A | -0.261 | 5.209 | -1.876 | 6.20E-02 | 1.47E-01 | -4.772 |
| NDST1 | 0.181 | 8.504 | 1.874 | 6.20E-02 | 1.47E-01 | -4.772 |
| PTTG2 | 0.228 | 7.675 | 1.874 | 6.20E-02 | 1.47E-01 | -4.772 |
| UMODL1 | 0.24 | 4.907 | 1.874 | 6.20E-02 | 1.47E-01 | -4.773 |
| DNAI2 | 0.221 | 5.418 | 1.873 | 6.20E-02 | 1.47E-01 | -4.775 |
| GAS2L3 | 0.233 | 6.488 | 1.873 | 6.20E-02 | 1.47E-01 | -4.775 |
| KCNQ5 | 0.181 | 5.467 | 1.873 | 6.20E-02 | 1.47E-01 | -4.776 |
| CAMK4 | -0.229 | 5.55 | -1.873 | 6.20E-02 | 1.47E-01 | -4.777 |
| PSCA | 0.386 | 6.736 | 1.871 | 6.30E-02 | 1.47E-01 | -4.778 |
| SHOX2 | -0.2 | 6.247 | -1.872 | 6.30E-02 | 1.47E-01 | -4.779 |
| SNX5 | -0.137 | 9.855 | -1.871 | 6.30E-02 | 1.48E-01 | -4.781 |
| NXPH3 | 0.188 | 6.138 | 1.869 | 6.30E-02 | 1.48E-01 | -4.783 |
| ALPI | 0.247 | 5.598 | 1.869 | 6.30E-02 | 1.48E-01 | -4.783 |
| DLGAP4 | 0.175 | 8.378 | 1.865 | 6.30E-02 | 1.49E-01 | -4.79 |
| ERO1L | -0.248 | 8.333 | -1.865 | 6.40E-02 | 1.49E-01 | -4.79 |
| AMPH | 0.246 | 5.184 | 1.864 | 6.40E-02 | 1.49E-01 | -4.791 |
| MYO1A | 0.265 | 6.081 | 1.862 | 6.40E-02 | 1.50E-01 | -4.796 |
| NINJ1 | 0.18 | 11.78 | 1.861 | 6.40E-02 | 1.50E-01 | -4.797 |
| ANGPTL7 | 0.24 | 5.079 | 1.861 | 6.40E-02 | 1.50E-01 | -4.797 |
| NDUFA4 | -0.205 | 11.828 | -1.861 | 6.40E-02 | 1.50E-01 | -4.798 |
| EXT1 | 0.19 | 8.854 | 1.86 | 6.40E-02 | 1.50E-01 | -4.798 |
| MMP20 | 0.204 | 4.745 | 1.858 | 6.40E-02 | 1.51E-01 | -4.803 |
| KCNA7 | 0.212 | 4.741 | 1.858 | 6.40E-02 | 1.51E-01 | -4.803 |
| RBM4 | -0.119 | 8.668 | -1.858 | 6.50E-02 | 1.51E-01 | -4.804 |
| SLC27A6 | -0.314 | 6.5 | -1.858 | 6.50E-02 | 1.51E-01 | -4.804 |
| SEMA4G | 0.204 | 6.159 | 1.856 | 6.50E-02 | 1.51E-01 | -4.805 |
| TLL2 | 0.187 | 5.075 | 1.856 | 6.50E-02 | 1.51E-01 | -4.805 |
| SFXN2 | 0.191 | 6.74 | 1.856 | 6.50E-02 | 1.51E-01 | -4.805 |
| GABRP | 0.297 | 7.057 | 1.855 | 6.50E-02 | 1.52E-01 | -4.807 |
| HPX | 0.226 | 5.623 | 1.852 | 6.50E-02 | 1.53E-01 | -4.812 |
| HOXB9 | 0.253 | 6.058 | 1.851 | 6.50E-02 | 1.53E-01 | -4.815 |
| CSNK1G1 | 0.122 | 6.737 | 1.85 | 6.60E-02 | 1.53E-01 | -4.817 |
| EHD1 | 0.191 | 11.12 | 1.85 | 6.60E-02 | 1.53E-01 | -4.817 |
| DTYMK | 0.147 | 8.884 | 1.849 | 6.60E-02 | 1.53E-01 | -4.818 |
| PTER | -0.245 | 6.975 | -1.85 | 6.60E-02 | 1.53E-01 | -4.818 |
| CABP5 | 0.265 | 6.959 | 1.849 | 6.60E-02 | 1.53E-01 | -4.818 |
| FMN2 | 0.223 | 5.379 | 1.849 | 6.60E-02 | 1.53E-01 | -4.819 |
| CD160 | 0.234 | 5.656 | 1.848 | 6.60E-02 | 1.53E-01 | -4.819 |
| TRIM14 | 0.164 | 7.143 | 1.848 | 6.60E-02 | 1.53E-01 | -4.819 |
| CST9L | 0.159 | 4.716 | 1.848 | 6.60E-02 | 1.53E-01 | -4.819 |
| TOM1L1 | -0.187 | 6.173 | -1.849 | 6.60E-02 | 1.53E-01 | -4.82 |
| ZNF346 | 0.221 | 6.702 | 1.847 | 6.60E-02 | 1.54E-01 | -4.821 |
| UCP3 | 0.263 | 7.086 | 1.847 | 6.60E-02 | 1.54E-01 | -4.822 |
| RDH13 | 0.326 | 8.35 | 1.846 | 6.60E-02 | 1.54E-01 | -4.824 |
| DPP3 | 0.206 | 7.858 | 1.845 | 6.60E-02 | 1.54E-01 | -4.825 |
| GRK6 | 0.169 | 8.288 | 1.845 | 6.60E-02 | 1.54E-01 | -4.826 |
| ARHGEF9 | -0.157 | 7.158 | -1.843 | 6.70E-02 | 1.55E-01 | -4.83 |
| RS1 | -0.242 | 4.872 | -1.843 | 6.70E-02 | 1.55E-01 | -4.83 |
| PFKP | 0.284 | 8.895 | 1.841 | 6.70E-02 | 1.55E-01 | -4.831 |
| M6PR | -0.144 | 9.732 | -1.842 | 6.70E-02 | 1.55E-01 | -4.833 |
| SIGLEC7 | 0.204 | 6.416 | 1.839 | 6.70E-02 | 1.56E-01 | -4.836 |
| BCL7C | 0.168 | 8.107 | 1.839 | 6.70E-02 | 1.56E-01 | -4.836 |
| WEE1 | -0.237 | 8.413 | -1.84 | 6.70E-02 | 1.56E-01 | -4.836 |
| APCDD1 | 0.342 | 8.445 | 1.838 | 6.70E-02 | 1.56E-01 | -4.838 |
| GABARAPL2 | -0.136 | 11.457 | -1.839 | 6.70E-02 | 1.56E-01 | -4.838 |
| GEM | 0.293 | 6.495 | 1.838 | 6.70E-02 | 1.56E-01 | -4.838 |
| SPAG16 | -0.159 | 6.254 | -1.838 | 6.70E-02 | 1.56E-01 | -4.839 |
| MAPK8IP1 | 0.321 | 6.364 | 1.837 | 6.80E-02 | 1.56E-01 | -4.84 |
| ZNF35 | 0.181 | 7.286 | 1.836 | 6.80E-02 | 1.57E-01 | -4.841 |
| DMRTB1 | 0.187 | 4.982 | 1.836 | 6.80E-02 | 1.57E-01 | -4.841 |
| RNF41 | 0.18 | 7.006 | 1.836 | 6.80E-02 | 1.57E-01 | -4.842 |
| ADAMTS6 | -0.289 | 6.74 | -1.836 | 6.80E-02 | 1.57E-01 | -4.843 |
| MUM1 | 0.161 | 8.258 | 1.835 | 6.80E-02 | 1.57E-01 | -4.844 |
| SEMA3F | 0.263 | 7.339 | 1.834 | 6.80E-02 | 1.57E-01 | -4.844 |
| ARHGAP9 | -0.239 | 8.242 | -1.835 | 6.80E-02 | 1.57E-01 | -4.845 |
| RECQL4 | 0.21 | 7.326 | 1.832 | 6.80E-02 | 1.58E-01 | -4.847 |
| PROCR | 0.301 | 10.149 | 1.831 | 6.80E-02 | 1.58E-01 | -4.849 |
| SLC4A1 | 0.234 | 7.079 | 1.831 | 6.80E-02 | 1.58E-01 | -4.85 |
| RAB2B | 0.206 | 8.822 | 1.83 | 6.80E-02 | 1.58E-01 | -4.851 |
| SETMAR | 0.179 | 7.156 | 1.83 | 6.80E-02 | 1.58E-01 | -4.851 |
| LCN6 | 0.162 | 5.168 | 1.828 | 6.90E-02 | 1.59E-01 | -4.855 |
| LY86 | 0.186 | 8.184 | 1.827 | 6.90E-02 | 1.59E-01 | -4.856 |
| AIG1 | -0.19 | 8.118 | -1.828 | 6.90E-02 | 1.59E-01 | -4.857 |
| CDC14A | -0.201 | 6.064 | -1.828 | 6.90E-02 | 1.59E-01 | -4.857 |
| ITGBL1 | -0.221 | 6.853 | -1.827 | 6.90E-02 | 1.59E-01 | -4.858 |
| PPP2R2D | -0.132 | 8.213 | -1.827 | 6.90E-02 | 1.59E-01 | -4.859 |
| CRTAC1 | 0.251 | 5.445 | 1.826 | 6.90E-02 | 1.59E-01 | -4.859 |
| GTF2A2 | -0.134 | 10.604 | -1.826 | 6.90E-02 | 1.60E-01 | -4.861 |
| TLR9 | 0.227 | 5.981 | 1.824 | 6.90E-02 | 1.60E-01 | -4.861 |
| SPPL2B | 0.175 | 7.845 | 1.824 | 6.90E-02 | 1.60E-01 | -4.862 |
| CPT1A | 0.154 | 7.576 | 1.823 | 7.00E-02 | 1.60E-01 | -4.864 |
| ADAMTS12 | 0.173 | 5.225 | 1.822 | 7.00E-02 | 1.60E-01 | -4.866 |
| GPR87 | -0.239 | 6.379 | -1.823 | 7.00E-02 | 1.60E-01 | -4.866 |
| OIP5 | -0.273 | 7.42 | -1.822 | 7.00E-02 | 1.61E-01 | -4.868 |
| LMAN2L | 0.146 | 9.229 | 1.82 | 7.00E-02 | 1.61E-01 | -4.869 |
| BIN1 | 0.184 | 9.486 | 1.819 | 7.00E-02 | 1.61E-01 | -4.87 |
| TYK2 | 0.196 | 8.774 | 1.819 | 7.00E-02 | 1.61E-01 | -4.87 |
| ERMAP | -0.21 | 7.037 | -1.82 | 7.00E-02 | 1.61E-01 | -4.871 |
| EFTUD1 | 0.198 | 7.091 | 1.819 | 7.00E-02 | 1.61E-01 | -4.871 |
| CLOCK | -0.165 | 7.914 | -1.82 | 7.00E-02 | 1.61E-01 | -4.871 |
| GTF2H4 | 0.156 | 7.757 | 1.818 | 7.00E-02 | 1.61E-01 | -4.873 |
| BRS3 | 0.194 | 4.985 | 1.818 | 7.00E-02 | 1.61E-01 | -4.873 |
| EDIL3 | 0.237 | 6.073 | 1.817 | 7.10E-02 | 1.62E-01 | -4.875 |
| AP1S1 | 0.25 | 7.829 | 1.816 | 7.10E-02 | 1.62E-01 | -4.876 |
| UBE2J1 | -0.15 | 9.004 | -1.816 | 7.10E-02 | 1.62E-01 | -4.879 |
| PALM2 | 0.185 | 5.151 | 1.814 | 7.10E-02 | 1.62E-01 | -4.879 |
| DHCR7 | 0.233 | 8.648 | 1.813 | 7.10E-02 | 1.62E-01 | -4.881 |
| FAHD1 | -0.18 | 7.903 | -1.814 | 7.10E-02 | 1.63E-01 | -4.881 |
| CLUAP1 | 0.137 | 8.028 | 1.812 | 7.10E-02 | 1.63E-01 | -4.883 |
| TAF4 | -0.173 | 7.875 | -1.813 | 7.10E-02 | 1.63E-01 | -4.883 |
| ATP13A1 | 0.187 | 8.469 | 1.811 | 7.10E-02 | 1.63E-01 | -4.885 |
| RNF7 | -0.12 | 9.977 | -1.811 | 7.20E-02 | 1.63E-01 | -4.887 |
| ABCA7 | 0.211 | 7.105 | 1.809 | 7.20E-02 | 1.64E-01 | -4.888 |
| DCTD | -0.148 | 9.19 | -1.81 | 7.20E-02 | 1.64E-01 | -4.888 |
| CAMK1D | 0.16 | 7.341 | 1.807 | 7.20E-02 | 1.64E-01 | -4.891 |
| XRN2 | -0.249 | 8.769 | -1.807 | 7.20E-02 | 1.64E-01 | -4.893 |
| NRCAM | -0.195 | 8.228 | -1.807 | 7.20E-02 | 1.64E-01 | -4.893 |
| PVRL1 | 0.231 | 6.919 | 1.806 | 7.20E-02 | 1.64E-01 | -4.893 |
| TRIB2 | 0.188 | 7.936 | 1.806 | 7.20E-02 | 1.65E-01 | -4.895 |
| FGF3 | 0.244 | 7.213 | 1.805 | 7.20E-02 | 1.65E-01 | -4.895 |
| PLXNA1 | 0.187 | 8.36 | 1.805 | 7.20E-02 | 1.65E-01 | -4.895 |
| TNFSF10 | -0.305 | 9.7 | -1.806 | 7.20E-02 | 1.65E-01 | -4.896 |
| VHL | -0.161 | 9.762 | -1.806 | 7.20E-02 | 1.65E-01 | -4.896 |
| CTNNBL1 | 0.119 | 9.989 | 1.804 | 7.20E-02 | 1.65E-01 | -4.897 |
| USMG5 | -0.233 | 9.373 | -1.805 | 7.30E-02 | 1.65E-01 | -4.897 |
| DMP1 | 0.213 | 4.718 | 1.804 | 7.30E-02 | 1.65E-01 | -4.898 |
| USP34 | -0.191 | 8 | -1.805 | 7.30E-02 | 1.65E-01 | -4.898 |
| PURB | -0.219 | 8.943 | -1.804 | 7.30E-02 | 1.65E-01 | -4.898 |
| SLC12A7 | -0.19 | 7.871 | -1.803 | 7.30E-02 | 1.65E-01 | -4.9 |
| GATA2 | -0.253 | 9.253 | -1.802 | 7.30E-02 | 1.65E-01 | -4.902 |
| HTR6 | 0.248 | 5.745 | 1.801 | 7.30E-02 | 1.66E-01 | -4.902 |
| LHX5 | 0.201 | 4.943 | 1.8 | 7.30E-02 | 1.66E-01 | -4.904 |
| FABP3 | -0.24 | 5.559 | -1.801 | 7.30E-02 | 1.66E-01 | -4.905 |
| BCKDHA | 0.202 | 9.082 | 1.799 | 7.30E-02 | 1.66E-01 | -4.905 |
| ZNF287 | -0.223 | 5.576 | -1.8 | 7.30E-02 | 1.66E-01 | -4.906 |
| RGS17 | -0.236 | 5.014 | -1.799 | 7.30E-02 | 1.66E-01 | -4.907 |
| MYLK2 | 0.207 | 5.631 | 1.798 | 7.30E-02 | 1.66E-01 | -4.908 |
| EFNA2 | 0.217 | 6.024 | 1.796 | 7.40E-02 | 1.67E-01 | -4.91 |
| MAP2K7 | 0.174 | 7.635 | 1.795 | 7.40E-02 | 1.67E-01 | -4.912 |
| ENO3 | 0.189 | 7.481 | 1.795 | 7.40E-02 | 1.67E-01 | -4.912 |
| TOB2 | 0.18 | 7.884 | 1.794 | 7.40E-02 | 1.67E-01 | -4.914 |
| GBGT1 | 0.202 | 7.554 | 1.794 | 7.40E-02 | 1.67E-01 | -4.914 |
| RPS29 | 0.159 | 13.372 | 1.792 | 7.40E-02 | 1.68E-01 | -4.918 |
| DDI1 | -0.205 | 5.049 | -1.792 | 7.50E-02 | 1.69E-01 | -4.92 |
| LAMC1 | -0.222 | 11.045 | -1.789 | 7.50E-02 | 1.69E-01 | -4.924 |
| RABEP2 | 0.157 | 8.021 | 1.788 | 7.50E-02 | 1.69E-01 | -4.924 |
| SYN3 | 0.238 | 5.711 | 1.786 | 7.50E-02 | 1.70E-01 | -4.929 |
| PPP1R8 | 0.119 | 8.319 | 1.785 | 7.50E-02 | 1.70E-01 | -4.929 |
| MYOCD | 0.214 | 5.499 | 1.785 | 7.50E-02 | 1.70E-01 | -4.929 |
| RNPS1 | 0.12 | 9.908 | 1.785 | 7.60E-02 | 1.70E-01 | -4.931 |
| FMO5 | -0.186 | 5.817 | -1.785 | 7.60E-02 | 1.71E-01 | -4.931 |
| MAP3K3 | 0.173 | 9.067 | 1.784 | 7.60E-02 | 1.71E-01 | -4.931 |
| APCS | 0.276 | 5.91 | 1.784 | 7.60E-02 | 1.71E-01 | -4.932 |
| LIPC | -0.21 | 5.435 | -1.785 | 7.60E-02 | 1.71E-01 | -4.932 |
| ETV4 | 0.285 | 6.612 | 1.783 | 7.60E-02 | 1.71E-01 | -4.933 |
| PEX5 | -0.162 | 8.286 | -1.781 | 7.60E-02 | 1.72E-01 | -4.938 |
| IKBKG | 0.198 | 8.933 | 1.779 | 7.70E-02 | 1.72E-01 | -4.941 |
| COL2A1 | 0.17 | 5.778 | 1.778 | 7.70E-02 | 1.72E-01 | -4.942 |
| RPS6 | -0.145 | 13.476 | -1.779 | 7.70E-02 | 1.73E-01 | -4.943 |
| DDHD1 | -0.153 | 6.832 | -1.777 | 7.70E-02 | 1.73E-01 | -4.945 |
| SSH1 | 0.16 | 7.122 | 1.776 | 7.70E-02 | 1.73E-01 | -4.946 |
| OR51B2 | 0.189 | 4.838 | 1.776 | 7.70E-02 | 1.73E-01 | -4.946 |
| PTGES | -0.292 | 7.849 | -1.776 | 7.70E-02 | 1.73E-01 | -4.947 |
| SNTB2 | 0.131 | 8.458 | 1.775 | 7.70E-02 | 1.73E-01 | -4.947 |
| IL17D | -0.279 | 5.832 | -1.775 | 7.70E-02 | 1.74E-01 | -4.95 |
| E2F6 | -0.155 | 8.321 | -1.774 | 7.70E-02 | 1.74E-01 | -4.95 |
| RPL31 | -0.187 | 12.608 | -1.774 | 7.80E-02 | 1.74E-01 | -4.95 |
| RPL12 | 0.112 | 13.928 | 1.773 | 7.80E-02 | 1.74E-01 | -4.951 |
| CYBB | -0.193 | 8.703 | -1.774 | 7.80E-02 | 1.74E-01 | -4.951 |
| PF4 | 0.228 | 8.003 | 1.773 | 7.80E-02 | 1.74E-01 | -4.951 |
| TEC | -0.174 | 5.555 | -1.774 | 7.80E-02 | 1.74E-01 | -4.951 |
| OPLAH | 0.229 | 7.285 | 1.772 | 7.80E-02 | 1.74E-01 | -4.952 |
| MAPK8IP3 | 0.24 | 7.591 | 1.771 | 7.80E-02 | 1.74E-01 | -4.954 |
| GADD45A | -0.283 | 9.247 | -1.772 | 7.80E-02 | 1.74E-01 | -4.954 |
| ZNF660 | -0.273 | 5.46 | -1.772 | 7.80E-02 | 1.74E-01 | -4.954 |
| CPNE5 | 0.225 | 7.213 | 1.771 | 7.80E-02 | 1.74E-01 | -4.954 |
| AKR1A1 | 0.131 | 9.761 | 1.769 | 7.80E-02 | 1.75E-01 | -4.957 |
| GPR52 | 0.208 | 5.054 | 1.768 | 7.80E-02 | 1.75E-01 | -4.959 |
| RND2 | 0.227 | 5.597 | 1.768 | 7.80E-02 | 1.75E-01 | -4.96 |
| NME5 | -0.237 | 5.143 | -1.767 | 7.90E-02 | 1.76E-01 | -4.962 |
| DAPK1 | -0.211 | 9.054 | -1.767 | 7.90E-02 | 1.76E-01 | -4.962 |
| P2RY4 | 0.209 | 6.097 | 1.766 | 7.90E-02 | 1.76E-01 | -4.963 |
| FAM47B | 0.231 | 4.674 | 1.765 | 7.90E-02 | 1.76E-01 | -4.965 |
| PLA2G1B | 0.217 | 5.545 | 1.764 | 7.90E-02 | 1.76E-01 | -4.966 |
| PHF13 | 0.125 | 9.976 | 1.763 | 7.90E-02 | 1.76E-01 | -4.967 |
| OR12D3 | 0.217 | 4.928 | 1.763 | 7.90E-02 | 1.77E-01 | -4.968 |
| SLAMF9 | 0.197 | 6.641 | 1.763 | 7.90E-02 | 1.77E-01 | -4.968 |
| LGI1 | -0.206 | 4.714 | -1.764 | 7.90E-02 | 1.77E-01 | -4.968 |
| SMURF1 | -0.266 | 6.618 | -1.763 | 7.90E-02 | 1.77E-01 | -4.969 |
| KCNJ9 | 0.157 | 5.45 | 1.762 | 7.90E-02 | 1.77E-01 | -4.969 |
| HIST1H1T | -0.271 | 5.477 | -1.761 | 8.00E-02 | 1.77E-01 | -4.972 |
| DNALI1 | 0.232 | 6.532 | 1.759 | 8.00E-02 | 1.77E-01 | -4.974 |
| ICAM3 | 0.184 | 9.294 | 1.759 | 8.00E-02 | 1.78E-01 | -4.975 |
| CAMTA2 | -0.198 | 6.206 | -1.759 | 8.00E-02 | 1.78E-01 | -4.976 |
| SOX4 | -0.23 | 7.689 | -1.759 | 8.00E-02 | 1.78E-01 | -4.976 |
| NUP153 | -0.161 | 10.043 | -1.759 | 8.00E-02 | 1.78E-01 | -4.976 |
| SLC30A7 | -0.185 | 8.698 | -1.758 | 8.00E-02 | 1.78E-01 | -4.978 |
| KLF15 | 0.19 | 5.729 | 1.757 | 8.00E-02 | 1.78E-01 | -4.979 |
| NDUFB3 | -0.181 | 10.221 | -1.757 | 8.00E-02 | 1.78E-01 | -4.979 |
| GTPBP6 | 0.139 | 9.972 | 1.756 | 8.00E-02 | 1.78E-01 | -4.979 |
| FOXN1 | 0.207 | 6.214 | 1.755 | 8.00E-02 | 1.78E-01 | -4.98 |
| BTN3A2 | 0.31 | 8.808 | 1.755 | 8.10E-02 | 1.79E-01 | -4.981 |
| USH2A | -0.147 | 4.757 | -1.755 | 8.10E-02 | 1.79E-01 | -4.982 |
| MBD6 | -0.21 | 10.06 | -1.755 | 8.10E-02 | 1.79E-01 | -4.983 |
| FLOT1 | 0.202 | 10.375 | 1.754 | 8.10E-02 | 1.79E-01 | -4.983 |
| ACVR1B | 0.182 | 6.541 | 1.752 | 8.10E-02 | 1.79E-01 | -4.986 |
| CATSPER3 | 0.193 | 5.715 | 1.751 | 8.10E-02 | 1.80E-01 | -4.987 |
| EIF4G3 | 0.172 | 7.851 | 1.751 | 8.10E-02 | 1.80E-01 | -4.988 |
| TRIM2 | -0.214 | 7.053 | -1.752 | 8.10E-02 | 1.80E-01 | -4.989 |
| POLA2 | 0.16 | 7.974 | 1.75 | 8.10E-02 | 1.80E-01 | -4.99 |
| CDH8 | 0.141 | 4.79 | 1.75 | 8.10E-02 | 1.80E-01 | -4.99 |
| PKD1L1 | -0.206 | 4.865 | -1.75 | 8.20E-02 | 1.80E-01 | -4.992 |
| CAMKK2 | 0.162 | 8.734 | 1.748 | 8.20E-02 | 1.80E-01 | -4.993 |
| NAGS | 0.198 | 7.104 | 1.748 | 8.20E-02 | 1.80E-01 | -4.993 |
| USP3 | -0.159 | 8.833 | -1.749 | 8.20E-02 | 1.80E-01 | -4.993 |
| NICN1 | 0.205 | 8.469 | 1.747 | 8.20E-02 | 1.81E-01 | -4.994 |
| THRAP3 | -0.127 | 9.012 | -1.748 | 8.20E-02 | 1.81E-01 | -4.994 |
| CENPE | -0.222 | 7.265 | -1.748 | 8.20E-02 | 1.81E-01 | -4.995 |
| SIRT4 | 0.204 | 6.512 | 1.746 | 8.20E-02 | 1.81E-01 | -4.996 |
| TLN1 | 0.203 | 9.167 | 1.746 | 8.20E-02 | 1.81E-01 | -4.997 |
| ELAVL1 | -0.139 | 7.883 | -1.747 | 8.20E-02 | 1.81E-01 | -4.997 |
| FGF17 | -0.167 | 5.154 | -1.746 | 8.20E-02 | 1.81E-01 | -4.997 |
| HMOX2 | 0.126 | 8.693 | 1.745 | 8.20E-02 | 1.81E-01 | -4.997 |
| CYP3A43 | 0.225 | 5.675 | 1.745 | 8.20E-02 | 1.81E-01 | -4.999 |
| EXOC8 | -0.171 | 8.224 | -1.744 | 8.30E-02 | 1.82E-01 | -5.001 |
| OR5I1 | 0.171 | 4.741 | 1.742 | 8.30E-02 | 1.82E-01 | -5.004 |
| SELL | 0.262 | 6.897 | 1.741 | 8.30E-02 | 1.83E-01 | -5.005 |
| SNPH | 0.23 | 6.518 | 1.74 | 8.30E-02 | 1.83E-01 | -5.007 |
| TERF2 | 0.115 | 7.996 | 1.739 | 8.30E-02 | 1.83E-01 | -5.007 |
| CIDEC | 0.125 | 7.195 | 1.738 | 8.30E-02 | 1.83E-01 | -5.009 |
| PNPLA2 | 0.281 | 8.752 | 1.737 | 8.40E-02 | 1.84E-01 | -5.011 |
| SPHK2 | 0.204 | 8.88 | 1.737 | 8.40E-02 | 1.84E-01 | -5.012 |
| DPEP3 | 0.22 | 7.862 | 1.736 | 8.40E-02 | 1.84E-01 | -5.013 |
| PEX14 | 0.162 | 9.452 | 1.736 | 8.40E-02 | 1.84E-01 | -5.013 |
| ERN1 | 0.136 | 6.941 | 1.736 | 8.40E-02 | 1.84E-01 | -5.014 |
| UCP1 | -0.19 | 4.743 | -1.737 | 8.40E-02 | 1.84E-01 | -5.014 |
| SELENBP1 | 0.22 | 9.294 | 1.735 | 8.40E-02 | 1.84E-01 | -5.014 |
| CXXC1 | 0.17 | 8.858 | 1.735 | 8.40E-02 | 1.84E-01 | -5.015 |
| CD163 | -0.215 | 9.62 | -1.736 | 8.40E-02 | 1.84E-01 | -5.016 |
| LILRA1 | -0.197 | 6.631 | -1.733 | 8.50E-02 | 1.85E-01 | -5.02 |
| IMPA2 | -0.209 | 11.064 | -1.732 | 8.50E-02 | 1.85E-01 | -5.021 |
| RFC2 | -0.129 | 8.23 | -1.732 | 8.50E-02 | 1.85E-01 | -5.021 |
| AKR1B10 | -0.213 | 5.96 | -1.732 | 8.50E-02 | 1.85E-01 | -5.021 |
| TIMELESS | 0.144 | 8.352 | 1.731 | 8.50E-02 | 1.85E-01 | -5.022 |
| PRSS21 | 0.204 | 5.821 | 1.731 | 8.50E-02 | 1.86E-01 | -5.022 |
| HIST1H2AB | 0.181 | 7.331 | 1.73 | 8.50E-02 | 1.86E-01 | -5.023 |
| LRP8 | -0.269 | 8.052 | -1.729 | 8.50E-02 | 1.86E-01 | -5.027 |
| MCCC2 | -0.156 | 7.682 | -1.728 | 8.50E-02 | 1.87E-01 | -5.028 |
| KIAA1524 | -0.213 | 5.495 | -1.728 | 8.50E-02 | 1.87E-01 | -5.028 |
| COL19A1 | -0.19 | 5.184 | -1.728 | 8.60E-02 | 1.87E-01 | -5.029 |
| RAB30 | -0.186 | 6.251 | -1.727 | 8.60E-02 | 1.87E-01 | -5.029 |
| PGLYRP3 | 0.175 | 5.132 | 1.725 | 8.60E-02 | 1.87E-01 | -5.032 |
| SERPINA3 | -0.542 | 6.244 | -1.726 | 8.60E-02 | 1.87E-01 | -5.032 |
| FMR1 | -0.136 | 8.565 | -1.725 | 8.60E-02 | 1.88E-01 | -5.034 |
| BFSP2 | 0.186 | 5.364 | 1.722 | 8.60E-02 | 1.88E-01 | -5.036 |
| STAC | -0.228 | 5.322 | -1.723 | 8.60E-02 | 1.88E-01 | -5.037 |
| CCKAR | 0.163 | 4.991 | 1.721 | 8.70E-02 | 1.89E-01 | -5.038 |
| CDC25C | 0.236 | 6.691 | 1.721 | 8.70E-02 | 1.89E-01 | -5.039 |
| BMF | 0.161 | 7.339 | 1.72 | 8.70E-02 | 1.89E-01 | -5.04 |
| TIFA | -0.219 | 6.745 | -1.72 | 8.70E-02 | 1.89E-01 | -5.041 |
| MAN2A2 | 0.112 | 7.942 | 1.719 | 8.70E-02 | 1.89E-01 | -5.041 |
| PAOX | 0.168 | 7.206 | 1.719 | 8.70E-02 | 1.89E-01 | -5.042 |
| CYP27A1 | 0.264 | 7.86 | 1.718 | 8.70E-02 | 1.89E-01 | -5.043 |
| ALDH1A3 | 0.232 | 7.259 | 1.718 | 8.70E-02 | 1.89E-01 | -5.044 |
| ASF1B | 0.189 | 8.426 | 1.717 | 8.70E-02 | 1.89E-01 | -5.044 |
| ITGAE | -0.17 | 8.856 | -1.718 | 8.70E-02 | 1.90E-01 | -5.044 |
| ACYP1 | -0.142 | 9.121 | -1.717 | 8.80E-02 | 1.90E-01 | -5.047 |
| COL10A1 | 0.155 | 5.653 | 1.715 | 8.80E-02 | 1.90E-01 | -5.048 |
| EPS8L3 | 0.252 | 6.123 | 1.714 | 8.80E-02 | 1.91E-01 | -5.05 |
| KCTD1 | -0.162 | 7.948 | -1.714 | 8.80E-02 | 1.91E-01 | -5.051 |
| EPS15 | -0.164 | 8.431 | -1.713 | 8.80E-02 | 1.91E-01 | -5.053 |
| FBN2 | 0.205 | 10.505 | 1.711 | 8.80E-02 | 1.92E-01 | -5.055 |
| BRF2 | 0.134 | 8.447 | 1.71 | 8.90E-02 | 1.92E-01 | -5.056 |
| ARHGEF11 | -0.213 | 6.774 | -1.711 | 8.90E-02 | 1.92E-01 | -5.056 |
| HIST1H4D | 0.247 | 8.217 | 1.709 | 8.90E-02 | 1.92E-01 | -5.058 |
| PTGFRN | 0.255 | 7.385 | 1.709 | 8.90E-02 | 1.92E-01 | -5.058 |
| SPATA2 | 0.168 | 7.046 | 1.709 | 8.90E-02 | 1.92E-01 | -5.058 |
| HNMT | -0.168 | 7.406 | -1.71 | 8.90E-02 | 1.92E-01 | -5.059 |
| PNPO | -0.188 | 8.817 | -1.709 | 8.90E-02 | 1.92E-01 | -5.059 |
| MT1E | 0.199 | 9.218 | 1.708 | 8.90E-02 | 1.92E-01 | -5.06 |
| SNRPA | 0.197 | 9.762 | 1.708 | 8.90E-02 | 1.92E-01 | -5.06 |
| WNK4 | 0.178 | 5.762 | 1.707 | 8.90E-02 | 1.93E-01 | -5.062 |
| HIST1H1B | 0.298 | 5.855 | 1.706 | 8.90E-02 | 1.93E-01 | -5.062 |
| PTPRA | 0.122 | 9.485 | 1.706 | 8.90E-02 | 1.93E-01 | -5.064 |
| TIMM8B | -0.198 | 9.893 | -1.704 | 9.00E-02 | 1.94E-01 | -5.068 |
| CYP2C18 | 0.168 | 4.911 | 1.703 | 9.00E-02 | 1.94E-01 | -5.069 |
| SPTA1 | -0.275 | 6.551 | -1.703 | 9.00E-02 | 1.94E-01 | -5.069 |
| APOM | 0.193 | 6.718 | 1.702 | 9.00E-02 | 1.94E-01 | -5.07 |
| SUFU | 0.173 | 6.597 | 1.702 | 9.00E-02 | 1.94E-01 | -5.07 |
| CD4 | 0.231 | 8.144 | 1.701 | 9.00E-02 | 1.94E-01 | -5.071 |
| LGMN | -0.197 | 10.481 | -1.702 | 9.00E-02 | 1.94E-01 | -5.071 |
| FANCL | 0.141 | 8.694 | 1.7 | 9.00E-02 | 1.95E-01 | -5.072 |
| SESN2 | 0.129 | 8.078 | 1.7 | 9.00E-02 | 1.95E-01 | -5.072 |
| LZTFL1 | -0.185 | 7.499 | -1.701 | 9.10E-02 | 1.95E-01 | -5.073 |
| VPS52 | 0.15 | 8.332 | 1.699 | 9.10E-02 | 1.95E-01 | -5.074 |
| CSN1S1 | 0.22 | 5.368 | 1.699 | 9.10E-02 | 1.95E-01 | -5.075 |
| LSS | 0.254 | 8.336 | 1.698 | 9.10E-02 | 1.95E-01 | -5.076 |
| ARHGAP22 | 0.179 | 7.347 | 1.697 | 9.10E-02 | 1.96E-01 | -5.077 |
| CCNB1 | -0.197 | 8.477 | -1.698 | 9.10E-02 | 1.96E-01 | -5.078 |
| ADAMTS13 | 0.145 | 6.31 | 1.695 | 9.10E-02 | 1.96E-01 | -5.081 |
| CACNB2 | 0.194 | 4.983 | 1.694 | 9.20E-02 | 1.97E-01 | -5.083 |
| SLC25A25 | -0.184 | 8.283 | -1.694 | 9.20E-02 | 1.97E-01 | -5.084 |
| ACO1 | -0.127 | 10.093 | -1.694 | 9.20E-02 | 1.97E-01 | -5.084 |
| CDC23 | -0.129 | 9.096 | -1.694 | 9.20E-02 | 1.97E-01 | -5.084 |
| ITK | -0.218 | 6.004 | -1.693 | 9.20E-02 | 1.97E-01 | -5.086 |
| BATF | 0.209 | 7.473 | 1.691 | 9.20E-02 | 1.98E-01 | -5.088 |
| KCNA3 | 0.283 | 5.631 | 1.691 | 9.20E-02 | 1.98E-01 | -5.088 |
| ALOX12 | 0.239 | 6.157 | 1.691 | 9.20E-02 | 1.98E-01 | -5.088 |
| CD86 | 0.363 | 7.255 | 1.69 | 9.20E-02 | 1.98E-01 | -5.089 |
| WNT9A | 0.182 | 5.166 | 1.689 | 9.30E-02 | 1.98E-01 | -5.091 |
| TXNIP | -0.185 | 12.886 | -1.689 | 9.30E-02 | 1.98E-01 | -5.092 |
| ITGA10 | 0.197 | 6.542 | 1.688 | 9.30E-02 | 1.98E-01 | -5.092 |
| KCNC3 | 0.166 | 6.449 | 1.688 | 9.30E-02 | 1.98E-01 | -5.092 |
| ODC1 | 0.202 | 9.768 | 1.688 | 9.30E-02 | 1.99E-01 | -5.093 |
| OR10H2 | 0.25 | 7.024 | 1.687 | 9.30E-02 | 1.99E-01 | -5.093 |
| LHFPL3 | -0.19 | 4.965 | -1.688 | 9.30E-02 | 1.99E-01 | -5.094 |
| SBF2 | -0.127 | 7.657 | -1.686 | 9.30E-02 | 1.99E-01 | -5.097 |
| CNN1 | 0.349 | 8.301 | 1.685 | 9.30E-02 | 2.00E-01 | -5.098 |
| DNAJC3 | -0.195 | 7.968 | -1.685 | 9.30E-02 | 2.00E-01 | -5.099 |
| PIGC | -0.188 | 8.789 | -1.685 | 9.40E-02 | 2.00E-01 | -5.099 |
| PACS2 | 0.17 | 7.287 | 1.684 | 9.40E-02 | 2.00E-01 | -5.099 |
| SPON1 | 0.246 | 7.595 | 1.683 | 9.40E-02 | 2.00E-01 | -5.101 |
| NKD2 | 0.197 | 8.304 | 1.682 | 9.40E-02 | 2.00E-01 | -5.102 |
| HAL | -0.193 | 5.637 | -1.683 | 9.40E-02 | 2.00E-01 | -5.103 |
| BAG4 | -0.166 | 7.126 | -1.682 | 9.40E-02 | 2.01E-01 | -5.104 |
| TNNI2 | 0.271 | 7.775 | 1.68 | 9.40E-02 | 2.01E-01 | -5.106 |
| LRRC25 | -0.237 | 7.096 | -1.681 | 9.40E-02 | 2.01E-01 | -5.106 |
| TIRAP | 0.129 | 6.517 | 1.679 | 9.40E-02 | 2.01E-01 | -5.107 |
| STAC2 | 0.207 | 5.781 | 1.679 | 9.50E-02 | 2.01E-01 | -5.108 |
| ENSA | -0.157 | 8.369 | -1.679 | 9.50E-02 | 2.02E-01 | -5.109 |
| ZNF34 | 0.132 | 6.789 | 1.678 | 9.50E-02 | 2.02E-01 | -5.109 |
| ZAP70 | 0.231 | 6.369 | 1.677 | 9.50E-02 | 2.02E-01 | -5.11 |
| ERBB2IP | -0.191 | 8.814 | -1.678 | 9.50E-02 | 2.02E-01 | -5.111 |
| MS4A6A | -0.203 | 8.794 | -1.678 | 9.50E-02 | 2.02E-01 | -5.111 |
| CRYGD | 0.187 | 5.21 | 1.676 | 9.50E-02 | 2.02E-01 | -5.112 |
| TIMP1 | 0.208 | 12.263 | 1.675 | 9.50E-02 | 2.02E-01 | -5.113 |
| SASH1 | 0.199 | 9.133 | 1.675 | 9.50E-02 | 2.02E-01 | -5.114 |
| CRLF1 | 0.371 | 7.428 | 1.675 | 9.50E-02 | 2.02E-01 | -5.114 |
| RPH3AL | 0.197 | 7.346 | 1.675 | 9.50E-02 | 2.02E-01 | -5.114 |
| GABRA4 | -0.181 | 5.271 | -1.676 | 9.50E-02 | 2.02E-01 | -5.114 |
| ARMC6 | 0.186 | 8.187 | 1.674 | 9.50E-02 | 2.03E-01 | -5.115 |
| REPIN1 | -0.132 | 9.128 | -1.674 | 9.60E-02 | 2.03E-01 | -5.116 |
| GPR173 | 0.171 | 5.983 | 1.673 | 9.60E-02 | 2.03E-01 | -5.117 |
| LIM2 | 0.22 | 5.364 | 1.673 | 9.60E-02 | 2.03E-01 | -5.118 |
| MYOM3 | 0.158 | 5.141 | 1.673 | 9.60E-02 | 2.03E-01 | -5.118 |
| GRIN2A | 0.168 | 5.103 | 1.671 | 9.60E-02 | 2.03E-01 | -5.12 |
| MMP16 | 0.146 | 5.159 | 1.671 | 9.60E-02 | 2.03E-01 | -5.12 |
| ARAF | 0.155 | 8.735 | 1.671 | 9.60E-02 | 2.03E-01 | -5.12 |
| FARP2 | 0.142 | 6.659 | 1.671 | 9.60E-02 | 2.03E-01 | -5.12 |
| PYCARD | 0.192 | 9.858 | 1.668 | 9.70E-02 | 2.04E-01 | -5.125 |
| SLC6A18 | 0.232 | 6.242 | 1.668 | 9.70E-02 | 2.04E-01 | -5.125 |
| COL23A1 | -0.208 | 6.41 | -1.668 | 9.70E-02 | 2.05E-01 | -5.127 |
| ARIH2 | -0.103 | 9.495 | -1.667 | 9.70E-02 | 2.05E-01 | -5.128 |
| SGSH | 0.157 | 10.01 | 1.666 | 9.70E-02 | 2.05E-01 | -5.128 |
| PGK2 | 0.151 | 4.824 | 1.666 | 9.70E-02 | 2.05E-01 | -5.128 |
| NCR3 | 0.189 | 6.232 | 1.665 | 9.70E-02 | 2.05E-01 | -5.13 |
| LHX6 | 0.163 | 6.614 | 1.664 | 9.70E-02 | 2.06E-01 | -5.131 |
| UCN2 | 0.249 | 8.219 | 1.664 | 9.70E-02 | 2.06E-01 | -5.131 |
| ATP2B2 | 0.155 | 4.872 | 1.664 | 9.70E-02 | 2.06E-01 | -5.131 |
| ARMC2 | -0.194 | 5.654 | -1.665 | 9.70E-02 | 2.06E-01 | -5.131 |
| ZNF253 | -0.166 | 7.39 | -1.664 | 9.80E-02 | 2.06E-01 | -5.132 |
| S100A12 | -0.32 | 7.743 | -1.664 | 9.80E-02 | 2.06E-01 | -5.133 |
| DPYSL4 | 0.285 | 6.386 | 1.663 | 9.80E-02 | 2.06E-01 | -5.133 |
| C6orf201 | 0.174 | 4.823 | 1.662 | 9.80E-02 | 2.06E-01 | -5.135 |
| CXXC4 | 0.222 | 6.027 | 1.661 | 9.80E-02 | 2.06E-01 | -5.135 |
| AP1S3 | -0.219 | 5.884 | -1.662 | 9.80E-02 | 2.06E-01 | -5.136 |
| ADAM17 | 0.121 | 8.906 | 1.661 | 9.80E-02 | 2.06E-01 | -5.136 |
| PTAFR | -0.177 | 7.832 | -1.662 | 9.80E-02 | 2.06E-01 | -5.136 |
| ABCF2 | 0.111 | 8.064 | 1.661 | 9.80E-02 | 2.06E-01 | -5.137 |
| UBE2D2 | 0.109 | 9.166 | 1.66 | 9.80E-02 | 2.06E-01 | -5.137 |
| P2RX5 | -0.199 | 5.96 | -1.661 | 9.80E-02 | 2.06E-01 | -5.137 |
| VTI1A | 0.136 | 7.269 | 1.66 | 9.80E-02 | 2.07E-01 | -5.138 |
| BMP3 | 0.145 | 4.764 | 1.657 | 9.90E-02 | 2.08E-01 | -5.142 |
| DOCK10 | -0.196 | 7.4 | -1.657 | 9.90E-02 | 2.08E-01 | -5.144 |
| PIGV | -0.129 | 8.772 | -1.657 | 9.90E-02 | 2.08E-01 | -5.144 |
| NMU | 0.277 | 6.827 | 1.656 | 9.90E-02 | 2.08E-01 | -5.144 |
| IL11RA | 0.131 | 8.376 | 1.654 | 1.00E-01 | 2.09E-01 | -5.148 |
| CYP8B1 | 0.184 | 5.796 | 1.653 | 1.00E-01 | 2.09E-01 | -5.149 |
| FASTK | 0.142 | 9.9 | 1.653 | 1.00E-01 | 2.09E-01 | -5.149 |
| RSAD1 | 0.115 | 8.932 | 1.652 | 1.00E-01 | 2.09E-01 | -5.15 |
| TRIM42 | 0.196 | 5.121 | 1.652 | 1.00E-01 | 2.09E-01 | -5.15 |
| PLA2G2E | 0.206 | 4.958 | 1.652 | 1.00E-01 | 2.09E-01 | -5.15 |
| DISC1 | 0.127 | 6.577 | 1.652 | 1.00E-01 | 2.09E-01 | -5.151 |
| HOXB4 | 0.152 | 7.219 | 1.652 | 1.00E-01 | 2.09E-01 | -5.151 |
| TBC1D20 | 0.19 | 7.913 | 1.649 | 1.00E-01 | 2.10E-01 | -5.155 |
| EGR2 | -0.32 | 6.189 | -1.649 | 1.01E-01 | 2.10E-01 | -5.156 |
| SIDT2 | -0.145 | 10.04 | -1.649 | 1.01E-01 | 2.10E-01 | -5.157 |
| POLR2G | 0.128 | 10.95 | 1.648 | 1.01E-01 | 2.10E-01 | -5.157 |
| NFATC2IP | 0.136 | 7.678 | 1.647 | 1.01E-01 | 2.11E-01 | -5.158 |
| HOXC13 | 0.21 | 5.27 | 1.647 | 1.01E-01 | 2.11E-01 | -5.158 |
| CACNG7 | 0.17 | 5.274 | 1.647 | 1.01E-01 | 2.11E-01 | -5.159 |
| NYX | 0.203 | 7.732 | 1.647 | 1.01E-01 | 2.11E-01 | -5.159 |
| CYP4F2 | -0.196 | 5.72 | -1.646 | 1.01E-01 | 2.11E-01 | -5.162 |
| MED12 | 0.191 | 8.25 | 1.645 | 1.01E-01 | 2.11E-01 | -5.162 |
| MPP5 | -0.212 | 8.756 | -1.646 | 1.01E-01 | 2.12E-01 | -5.162 |
| DLL3 | 0.171 | 5.371 | 1.645 | 1.01E-01 | 2.12E-01 | -5.163 |
| RAB17 | 0.226 | 6.577 | 1.644 | 1.01E-01 | 2.12E-01 | -5.163 |
| KCNH4 | 0.198 | 5.855 | 1.644 | 1.02E-01 | 2.12E-01 | -5.163 |
| SERTAD4 | 0.303 | 6.833 | 1.643 | 1.02E-01 | 2.12E-01 | -5.166 |
| LEPR | -0.196 | 6.292 | -1.643 | 1.02E-01 | 2.12E-01 | -5.166 |
| MARCKSL1 | 0.195 | 9.933 | 1.64 | 1.02E-01 | 2.13E-01 | -5.17 |
| ANKRD17 | 0.118 | 8.319 | 1.64 | 1.02E-01 | 2.13E-01 | -5.17 |
| LHX2 | 0.231 | 6.506 | 1.64 | 1.02E-01 | 2.13E-01 | -5.17 |
| SDK1 | 0.195 | 5.335 | 1.639 | 1.03E-01 | 2.14E-01 | -5.172 |
| MLN | 0.182 | 5.214 | 1.639 | 1.03E-01 | 2.14E-01 | -5.172 |
| ANP32D | -0.211 | 6.78 | -1.639 | 1.03E-01 | 2.14E-01 | -5.172 |
| MDC1 | -0.14 | 8.925 | -1.639 | 1.03E-01 | 2.14E-01 | -5.173 |
| MRPS14 | 0.125 | 8.855 | 1.637 | 1.03E-01 | 2.14E-01 | -5.174 |
| MYOC | -0.218 | 4.998 | -1.637 | 1.03E-01 | 2.14E-01 | -5.176 |
| UNC13B | 0.196 | 6.713 | 1.636 | 1.03E-01 | 2.14E-01 | -5.176 |
| DNAH7 | 0.18 | 5.105 | 1.635 | 1.03E-01 | 2.15E-01 | -5.178 |
| FGF8 | -0.171 | 5.903 | -1.635 | 1.04E-01 | 2.15E-01 | -5.179 |
| RNASEL | -0.17 | 7.982 | -1.635 | 1.04E-01 | 2.15E-01 | -5.179 |
| SNX9 | -0.17 | 7.774 | -1.635 | 1.04E-01 | 2.15E-01 | -5.179 |
| PPIG | -0.213 | 8.427 | -1.635 | 1.04E-01 | 2.15E-01 | -5.18 |
| PDE8B | 0.212 | 8.141 | 1.632 | 1.04E-01 | 2.16E-01 | -5.182 |
| MNS1 | -0.208 | 6.179 | -1.633 | 1.04E-01 | 2.16E-01 | -5.183 |
| MAGEB3 | -0.21 | 5.012 | -1.632 | 1.04E-01 | 2.16E-01 | -5.184 |
| GDAP2 | -0.111 | 7.392 | -1.632 | 1.04E-01 | 2.16E-01 | -5.184 |
| AP3B2 | 0.261 | 5.717 | 1.631 | 1.04E-01 | 2.16E-01 | -5.184 |
| ZRANB1 | 0.198 | 8.115 | 1.63 | 1.04E-01 | 2.16E-01 | -5.185 |
| TSGA10 | -0.181 | 6.08 | -1.631 | 1.04E-01 | 2.16E-01 | -5.185 |
| ANGPT4 | 0.239 | 5.255 | 1.63 | 1.04E-01 | 2.16E-01 | -5.186 |
| KRT6B | 0.149 | 5.305 | 1.63 | 1.05E-01 | 2.16E-01 | -5.186 |
| DMGDH | -0.169 | 4.858 | -1.63 | 1.05E-01 | 2.16E-01 | -5.187 |
| IL27 | 0.306 | 7.539 | 1.629 | 1.05E-01 | 2.16E-01 | -5.187 |
| NEK7 | -0.292 | 7.788 | -1.629 | 1.05E-01 | 2.17E-01 | -5.188 |
| LY6K | 0.249 | 7.117 | 1.626 | 1.05E-01 | 2.18E-01 | -5.192 |
| AMIGO2 | -0.202 | 7.636 | -1.626 | 1.05E-01 | 2.18E-01 | -5.193 |
| ADAMTS5 | -0.276 | 8.392 | -1.626 | 1.05E-01 | 2.18E-01 | -5.193 |
| TUBB | -0.197 | 10.993 | -1.626 | 1.05E-01 | 2.18E-01 | -5.193 |
| PCBP1 | -0.168 | 12.541 | -1.626 | 1.06E-01 | 2.18E-01 | -5.194 |
| KCNK1 | -0.248 | 7.032 | -1.625 | 1.06E-01 | 2.18E-01 | -5.195 |
| SLCO1C1 | -0.152 | 4.658 | -1.624 | 1.06E-01 | 2.18E-01 | -5.196 |
| PGRMC2 | -0.147 | 11.872 | -1.623 | 1.06E-01 | 2.19E-01 | -5.198 |
| AHSA1 | -0.123 | 10.429 | -1.623 | 1.06E-01 | 2.19E-01 | -5.198 |
| SLC31A1 | -0.145 | 8.46 | -1.622 | 1.06E-01 | 2.19E-01 | -5.2 |
| SCUBE3 | -0.191 | 5.325 | -1.621 | 1.07E-01 | 2.20E-01 | -5.201 |
| SCYL1 | 0.189 | 10.054 | 1.619 | 1.07E-01 | 2.20E-01 | -5.203 |
| NMNAT3 | 0.211 | 5.868 | 1.619 | 1.07E-01 | 2.20E-01 | -5.203 |
| MRPS5 | 0.109 | 9.401 | 1.618 | 1.07E-01 | 2.20E-01 | -5.205 |
| ITGA2B | 0.201 | 6.992 | 1.617 | 1.07E-01 | 2.20E-01 | -5.205 |
| GFER | 0.138 | 7.076 | 1.617 | 1.07E-01 | 2.20E-01 | -5.205 |
| TACR3 | 0.205 | 4.885 | 1.617 | 1.07E-01 | 2.21E-01 | -5.206 |
| PRKCE | -0.177 | 7.343 | -1.617 | 1.08E-01 | 2.21E-01 | -5.208 |
| OR7C2 | 0.186 | 5.654 | 1.615 | 1.08E-01 | 2.21E-01 | -5.208 |
| XPO4 | -0.143 | 7.653 | -1.616 | 1.08E-01 | 2.21E-01 | -5.209 |
| HSPA2 | -0.214 | 9.459 | -1.616 | 1.08E-01 | 2.21E-01 | -5.209 |
| ATP2B4 | 0.162 | 8.118 | 1.614 | 1.08E-01 | 2.21E-01 | -5.21 |
| ZNF585B | -0.143 | 6.846 | -1.615 | 1.08E-01 | 2.21E-01 | -5.21 |
| CYLD | -0.163 | 7.554 | -1.615 | 1.08E-01 | 2.21E-01 | -5.211 |
| STC2 | 0.264 | 7.141 | 1.614 | 1.08E-01 | 2.21E-01 | -5.211 |
| SOST | 0.233 | 5.346 | 1.613 | 1.08E-01 | 2.22E-01 | -5.212 |
| HERC6 | 0.221 | 7.085 | 1.613 | 1.08E-01 | 2.22E-01 | -5.212 |
| WNT5A | -0.227 | 7.892 | -1.614 | 1.08E-01 | 2.22E-01 | -5.213 |
| ACOXL | -0.335 | 5.829 | -1.613 | 1.08E-01 | 2.22E-01 | -5.213 |
| MARK2 | 0.132 | 7.911 | 1.612 | 1.08E-01 | 2.22E-01 | -5.213 |
| PAPLN | -0.215 | 6.539 | -1.612 | 1.08E-01 | 2.22E-01 | -5.215 |
| DIAPH1 | -0.228 | 9.864 | -1.612 | 1.09E-01 | 2.22E-01 | -5.216 |
| SLC25A22 | 0.168 | 8.373 | 1.611 | 1.09E-01 | 2.22E-01 | -5.216 |
| FGF18 | -0.195 | 5.536 | -1.611 | 1.09E-01 | 2.22E-01 | -5.217 |
| OSBPL5 | 0.175 | 8.153 | 1.61 | 1.09E-01 | 2.22E-01 | -5.217 |
| TRPV2 | 0.168 | 9.292 | 1.61 | 1.09E-01 | 2.22E-01 | -5.217 |
| SDS | 0.183 | 6.199 | 1.61 | 1.09E-01 | 2.22E-01 | -5.217 |
| CHRND | 0.196 | 5.407 | 1.608 | 1.09E-01 | 2.23E-01 | -5.219 |
| GGA3 | -0.092 | 8.936 | -1.609 | 1.09E-01 | 2.23E-01 | -5.221 |
| ATP6V1D | 0.139 | 10.114 | 1.607 | 1.09E-01 | 2.23E-01 | -5.222 |
| DFNB31 | 0.171 | 5.648 | 1.607 | 1.09E-01 | 2.23E-01 | -5.222 |
| RCOR2 | 0.193 | 7.713 | 1.606 | 1.10E-01 | 2.24E-01 | -5.224 |
| TK1 | 0.254 | 10.176 | 1.605 | 1.10E-01 | 2.24E-01 | -5.225 |
| OCLN | -0.204 | 6.435 | -1.606 | 1.10E-01 | 2.24E-01 | -5.225 |
| PTDSS2 | 0.203 | 9.429 | 1.604 | 1.10E-01 | 2.24E-01 | -5.226 |
| EPC1 | 0.158 | 8.137 | 1.604 | 1.10E-01 | 2.24E-01 | -5.226 |
| AKAP13 | 0.17 | 8.459 | 1.604 | 1.10E-01 | 2.24E-01 | -5.227 |
| CTNND2 | 0.202 | 5.757 | 1.604 | 1.10E-01 | 2.24E-01 | -5.227 |
| RELB | 0.239 | 8.619 | 1.603 | 1.10E-01 | 2.25E-01 | -5.227 |
| APLP1 | 0.18 | 5.857 | 1.602 | 1.10E-01 | 2.25E-01 | -5.229 |
| TYR | 0.131 | 4.622 | 1.602 | 1.10E-01 | 2.25E-01 | -5.229 |
| CRP | 0.207 | 5.329 | 1.602 | 1.10E-01 | 2.25E-01 | -5.229 |
| PTGIS | 0.228 | 5.783 | 1.601 | 1.11E-01 | 2.25E-01 | -5.23 |
| CPB2 | 0.165 | 4.793 | 1.601 | 1.11E-01 | 2.25E-01 | -5.231 |
| PLB1 | 0.184 | 6.223 | 1.6 | 1.11E-01 | 2.26E-01 | -5.233 |
| REST | 0.157 | 6.925 | 1.598 | 1.11E-01 | 2.26E-01 | -5.235 |
| FOXE1 | 0.174 | 5.478 | 1.598 | 1.11E-01 | 2.26E-01 | -5.236 |
| LYZL4 | 0.175 | 4.851 | 1.597 | 1.12E-01 | 2.27E-01 | -5.237 |
| ALOX12B | 0.198 | 6.154 | 1.597 | 1.12E-01 | 2.27E-01 | -5.238 |
| NPHP3 | 0.153 | 8.137 | 1.596 | 1.12E-01 | 2.27E-01 | -5.238 |
| ASB4 | 0.185 | 5.536 | 1.596 | 1.12E-01 | 2.27E-01 | -5.239 |
| GPR101 | 0.169 | 4.798 | 1.596 | 1.12E-01 | 2.27E-01 | -5.239 |
| WFDC2 | 0.181 | 5.991 | 1.596 | 1.12E-01 | 2.27E-01 | -5.239 |
| SH3GL2 | 0.197 | 4.801 | 1.594 | 1.12E-01 | 2.28E-01 | -5.242 |
| ZNF449 | -0.161 | 6.221 | -1.595 | 1.12E-01 | 2.28E-01 | -5.242 |
| AOC2 | 0.139 | 6.073 | 1.593 | 1.12E-01 | 2.28E-01 | -5.243 |
| DACH2 | 0.167 | 4.789 | 1.593 | 1.13E-01 | 2.28E-01 | -5.243 |
| FKBPL | 0.167 | 7.069 | 1.592 | 1.13E-01 | 2.28E-01 | -5.245 |
| GLIS2 | 0.206 | 7.556 | 1.592 | 1.13E-01 | 2.28E-01 | -5.245 |
| RARG | 0.212 | 7.308 | 1.591 | 1.13E-01 | 2.28E-01 | -5.246 |
| IRX5 | 0.251 | 5.22 | 1.591 | 1.13E-01 | 2.29E-01 | -5.247 |
| CHRNG | 0.143 | 4.823 | 1.59 | 1.13E-01 | 2.29E-01 | -5.247 |
| FBXO32 | -0.208 | 8.358 | -1.591 | 1.13E-01 | 2.29E-01 | -5.248 |
| BRMS1L | -0.174 | 7.198 | -1.589 | 1.14E-01 | 2.30E-01 | -5.25 |
| CARHSP1 | 0.188 | 11.032 | 1.588 | 1.14E-01 | 2.30E-01 | -5.251 |
| HOXA5 | 0.225 | 6.817 | 1.588 | 1.14E-01 | 2.30E-01 | -5.251 |
| MAP2 | -0.162 | 5.32 | -1.588 | 1.14E-01 | 2.30E-01 | -5.252 |
| PLAU | -0.227 | 10.674 | -1.588 | 1.14E-01 | 2.30E-01 | -5.253 |
| PHPT1 | 0.129 | 11.005 | 1.586 | 1.14E-01 | 2.30E-01 | -5.253 |
| FSHB | -0.171 | 4.787 | -1.587 | 1.14E-01 | 2.30E-01 | -5.254 |
| CMAS | -0.151 | 9.512 | -1.586 | 1.14E-01 | 2.31E-01 | -5.256 |
| KCNG4 | 0.17 | 5.453 | 1.585 | 1.14E-01 | 2.31E-01 | -5.256 |
| SERPINB9 | 0.215 | 8.029 | 1.584 | 1.14E-01 | 2.31E-01 | -5.256 |
| PLAGL2 | 0.121 | 8.392 | 1.583 | 1.15E-01 | 2.31E-01 | -5.258 |
| BAIAP2L2 | 0.189 | 6.61 | 1.583 | 1.15E-01 | 2.31E-01 | -5.258 |
| NCOR2 | -0.175 | 10.51 | -1.584 | 1.15E-01 | 2.31E-01 | -5.259 |
| RPRM | 0.302 | 5.538 | 1.582 | 1.15E-01 | 2.32E-01 | -5.26 |
| CDCA2 | 0.235 | 6.693 | 1.582 | 1.15E-01 | 2.32E-01 | -5.26 |
| DOK2 | -0.171 | 6.711 | -1.583 | 1.15E-01 | 2.32E-01 | -5.261 |
| ZNF547 | -0.15 | 5.593 | -1.582 | 1.15E-01 | 2.32E-01 | -5.262 |
| REM1 | 0.192 | 5.597 | 1.58 | 1.15E-01 | 2.32E-01 | -5.263 |
| C9orf40 | 0.206 | 7.804 | 1.58 | 1.15E-01 | 2.32E-01 | -5.263 |
| SPARCL1 | 0.254 | 9.19 | 1.579 | 1.16E-01 | 2.32E-01 | -5.264 |
| MIP | 0.186 | 5.03 | 1.579 | 1.16E-01 | 2.32E-01 | -5.264 |
| KCNK15 | 0.198 | 7.283 | 1.579 | 1.16E-01 | 2.32E-01 | -5.265 |
| HRH4 | -0.221 | 4.729 | -1.58 | 1.16E-01 | 2.32E-01 | -5.265 |
| SEMA4F | 0.178 | 6.428 | 1.578 | 1.16E-01 | 2.33E-01 | -5.266 |
| ZNF419 | -0.143 | 7.479 | -1.579 | 1.16E-01 | 2.33E-01 | -5.266 |
| SEC61A2 | -0.157 | 6.68 | -1.579 | 1.16E-01 | 2.33E-01 | -5.267 |
| NOS1 | 0.139 | 5.438 | 1.577 | 1.16E-01 | 2.33E-01 | -5.268 |
| JAG1 | -0.192 | 8.906 | -1.578 | 1.16E-01 | 2.33E-01 | -5.268 |
| SETDB1 | 0.115 | 8.984 | 1.576 | 1.16E-01 | 2.33E-01 | -5.269 |
| KIT | -0.22 | 7.222 | -1.576 | 1.16E-01 | 2.34E-01 | -5.27 |
| MOG | 0.154 | 5.361 | 1.575 | 1.17E-01 | 2.34E-01 | -5.27 |
| MAGEA10 | 0.204 | 7.465 | 1.575 | 1.17E-01 | 2.34E-01 | -5.271 |
| SUCLG2 | -0.128 | 8.498 | -1.575 | 1.17E-01 | 2.34E-01 | -5.273 |
| SF3B2 | 0.113 | 11.539 | 1.573 | 1.17E-01 | 2.34E-01 | -5.273 |
| ZNF645 | 0.187 | 4.885 | 1.573 | 1.17E-01 | 2.34E-01 | -5.274 |
| COG8 | 0.104 | 7.918 | 1.573 | 1.17E-01 | 2.34E-01 | -5.274 |
| PF4V1 | 0.224 | 6.394 | 1.572 | 1.17E-01 | 2.35E-01 | -5.275 |
| BAHD1 | -0.101 | 8.941 | -1.573 | 1.17E-01 | 2.35E-01 | -5.275 |
| MYO5C | -0.222 | 6.156 | -1.573 | 1.17E-01 | 2.35E-01 | -5.276 |
| WNT16 | 0.222 | 4.991 | 1.57 | 1.18E-01 | 2.35E-01 | -5.278 |
| TNFRSF13C | 0.141 | 6.375 | 1.57 | 1.18E-01 | 2.35E-01 | -5.278 |
| KCNK2 | 0.179 | 4.69 | 1.57 | 1.18E-01 | 2.35E-01 | -5.278 |
| TM4SF4 | 0.201 | 4.903 | 1.57 | 1.18E-01 | 2.35E-01 | -5.278 |
| STAT6 | -0.216 | 10.116 | -1.571 | 1.18E-01 | 2.35E-01 | -5.279 |
| NKX2-3 | 0.19 | 6.054 | 1.569 | 1.18E-01 | 2.35E-01 | -5.279 |
| DEFB1 | 0.307 | 6.849 | 1.569 | 1.18E-01 | 2.35E-01 | -5.279 |
| SDAD1 | 0.185 | 8.483 | 1.569 | 1.18E-01 | 2.35E-01 | -5.28 |
| TIGD3 | -0.169 | 5.975 | -1.57 | 1.18E-01 | 2.35E-01 | -5.28 |
| CAMKK1 | -0.151 | 6.016 | -1.569 | 1.18E-01 | 2.36E-01 | -5.281 |
| ZNF79 | 0.155 | 7.214 | 1.568 | 1.18E-01 | 2.36E-01 | -5.282 |
| CLTA | 0.117 | 11.123 | 1.568 | 1.18E-01 | 2.36E-01 | -5.282 |
| CPNE7 | 0.204 | 6.666 | 1.567 | 1.18E-01 | 2.36E-01 | -5.282 |
| PRPF4 | 0.114 | 9.054 | 1.567 | 1.18E-01 | 2.36E-01 | -5.283 |
| SLITRK5 | 0.135 | 4.857 | 1.567 | 1.18E-01 | 2.36E-01 | -5.283 |
| BIRC5 | 0.234 | 8.411 | 1.566 | 1.19E-01 | 2.36E-01 | -5.285 |
| ALOX5AP | -0.22 | 9.459 | -1.566 | 1.19E-01 | 2.37E-01 | -5.286 |
| SLC16A11 | 0.179 | 7.497 | 1.565 | 1.19E-01 | 2.37E-01 | -5.287 |
| PSG11 | -0.279 | 9.915 | -1.565 | 1.19E-01 | 2.37E-01 | -5.287 |
| PRC1 | 0.187 | 9.283 | 1.564 | 1.19E-01 | 2.37E-01 | -5.287 |
| PLTP | 0.239 | 9.721 | 1.564 | 1.19E-01 | 2.37E-01 | -5.287 |
| NAP1L4 | -0.135 | 9.828 | -1.564 | 1.19E-01 | 2.37E-01 | -5.288 |
| SYT5 | 0.182 | 5.39 | 1.563 | 1.19E-01 | 2.37E-01 | -5.289 |
| BSND | -0.191 | 4.933 | -1.564 | 1.19E-01 | 2.37E-01 | -5.289 |
| CASQ2 | 0.207 | 4.917 | 1.562 | 1.20E-01 | 2.37E-01 | -5.29 |
| RTN1 | -0.186 | 6.442 | -1.563 | 1.20E-01 | 2.37E-01 | -5.291 |
| ITGAL | 0.175 | 6.994 | 1.558 | 1.21E-01 | 2.39E-01 | -5.296 |
| PREB | 0.126 | 9.562 | 1.558 | 1.21E-01 | 2.39E-01 | -5.296 |
| TIPARP | -0.151 | 8.444 | -1.558 | 1.21E-01 | 2.39E-01 | -5.297 |
| CA2 | -0.306 | 7.786 | -1.558 | 1.21E-01 | 2.40E-01 | -5.298 |
| CSF1R | 0.173 | 11.284 | 1.556 | 1.21E-01 | 2.40E-01 | -5.299 |
| CLPS | 0.202 | 6.093 | 1.554 | 1.22E-01 | 2.41E-01 | -5.303 |
| F5 | 0.174 | 9.291 | 1.554 | 1.22E-01 | 2.41E-01 | -5.303 |
| IGSF9 | 0.207 | 6.409 | 1.553 | 1.22E-01 | 2.41E-01 | -5.304 |
| PAIP1 | -0.094 | 8.774 | -1.553 | 1.22E-01 | 2.42E-01 | -5.306 |
| ASNA1 | 0.164 | 8.428 | 1.552 | 1.22E-01 | 2.42E-01 | -5.306 |
| KLF13 | 0.131 | 8.981 | 1.551 | 1.22E-01 | 2.42E-01 | -5.307 |
| CCR9 | -0.246 | 5.166 | -1.552 | 1.22E-01 | 2.42E-01 | -5.307 |
| GPR55 | 0.173 | 5.105 | 1.551 | 1.22E-01 | 2.42E-01 | -5.307 |
| DHX38 | 0.139 | 8.389 | 1.549 | 1.23E-01 | 2.43E-01 | -5.31 |
| CLEC4D | -0.213 | 6.43 | -1.55 | 1.23E-01 | 2.43E-01 | -5.311 |
| ATP5L | 0.137 | 12.047 | 1.548 | 1.23E-01 | 2.43E-01 | -5.311 |
| OGN | 0.285 | 6.192 | 1.547 | 1.23E-01 | 2.43E-01 | -5.313 |
| ZNF319 | -0.17 | 7.449 | -1.548 | 1.23E-01 | 2.43E-01 | -5.313 |
| HCLS1 | 0.146 | 10.615 | 1.547 | 1.23E-01 | 2.43E-01 | -5.314 |
| LRRC31 | -0.144 | 5.415 | -1.547 | 1.23E-01 | 2.43E-01 | -5.314 |
| BLVRA | -0.233 | 10.222 | -1.547 | 1.23E-01 | 2.43E-01 | -5.314 |
| ZNF397 | -0.179 | 7.004 | -1.547 | 1.23E-01 | 2.43E-01 | -5.315 |
| KCNAB1 | -0.172 | 5.212 | -1.546 | 1.24E-01 | 2.44E-01 | -5.315 |
| RGS5 | -0.212 | 6.232 | -1.546 | 1.24E-01 | 2.44E-01 | -5.316 |
| MCCC1 | -0.107 | 8.922 | -1.546 | 1.24E-01 | 2.44E-01 | -5.316 |
| NPL | -0.137 | 8.729 | -1.546 | 1.24E-01 | 2.44E-01 | -5.316 |
| ABHD8 | -0.184 | 8.782 | -1.546 | 1.24E-01 | 2.44E-01 | -5.317 |
| STAB2 | 0.305 | 6.084 | 1.544 | 1.24E-01 | 2.44E-01 | -5.318 |
| DGKE | -0.144 | 5.246 | -1.544 | 1.24E-01 | 2.44E-01 | -5.319 |
| SEC23IP | -0.136 | 8.348 | -1.544 | 1.24E-01 | 2.44E-01 | -5.32 |
| TRPC1 | 0.211 | 7.158 | 1.54 | 1.25E-01 | 2.45E-01 | -5.323 |
| CFDP1 | -0.122 | 9.88 | -1.541 | 1.25E-01 | 2.45E-01 | -5.323 |
| ALG1 | 0.198 | 7.56 | 1.54 | 1.25E-01 | 2.45E-01 | -5.323 |
| TRIM31 | -0.154 | 4.712 | -1.541 | 1.25E-01 | 2.45E-01 | -5.323 |
| GPR84 | 0.188 | 6.792 | 1.54 | 1.25E-01 | 2.45E-01 | -5.324 |
| UNC93B1 | 0.179 | 7.706 | 1.54 | 1.25E-01 | 2.46E-01 | -5.324 |
| SMOC2 | -0.245 | 7.137 | -1.54 | 1.25E-01 | 2.46E-01 | -5.325 |
| MFNG | 0.127 | 9.512 | 1.539 | 1.25E-01 | 2.46E-01 | -5.325 |
| SELE | -0.234 | 5.562 | -1.54 | 1.25E-01 | 2.46E-01 | -5.325 |
| CX3CL1 | 0.241 | 6.764 | 1.539 | 1.25E-01 | 2.46E-01 | -5.326 |
| ELOVL3 | 0.217 | 5.757 | 1.538 | 1.25E-01 | 2.46E-01 | -5.326 |
| ITGA6 | -0.163 | 9.345 | -1.539 | 1.25E-01 | 2.46E-01 | -5.326 |
| PEX19 | 0.099 | 9.367 | 1.538 | 1.25E-01 | 2.46E-01 | -5.326 |
| HS3ST2 | 0.256 | 5.349 | 1.538 | 1.25E-01 | 2.46E-01 | -5.327 |
| TBR1 | 0.125 | 4.881 | 1.537 | 1.26E-01 | 2.46E-01 | -5.328 |
| GP1BA | 0.231 | 6.625 | 1.537 | 1.26E-01 | 2.46E-01 | -5.328 |
| GIMAP7 | 0.161 | 9.251 | 1.537 | 1.26E-01 | 2.46E-01 | -5.328 |
| C9orf24 | 0.161 | 5.784 | 1.536 | 1.26E-01 | 2.46E-01 | -5.33 |
| IRX3 | 0.206 | 7.157 | 1.536 | 1.26E-01 | 2.47E-01 | -5.33 |
| GIF | 0.166 | 4.834 | 1.535 | 1.26E-01 | 2.47E-01 | -5.331 |
| NOVA1 | 0.156 | 5.318 | 1.534 | 1.26E-01 | 2.47E-01 | -5.332 |
| TP73 | 0.167 | 5.506 | 1.534 | 1.26E-01 | 2.47E-01 | -5.333 |
| PLEKHM1 | -0.125 | 8.349 | -1.534 | 1.27E-01 | 2.47E-01 | -5.333 |
| IK | -0.107 | 10.808 | -1.532 | 1.27E-01 | 2.48E-01 | -5.337 |
| OXSR1 | 0.109 | 9.944 | 1.53 | 1.27E-01 | 2.49E-01 | -5.338 |
| USH1G | 0.188 | 5.533 | 1.529 | 1.28E-01 | 2.49E-01 | -5.34 |
| HSD17B3 | -0.185 | 5.009 | -1.53 | 1.28E-01 | 2.49E-01 | -5.34 |
| PANK4 | 0.136 | 8.112 | 1.529 | 1.28E-01 | 2.49E-01 | -5.34 |
| TRAF1 | 0.191 | 6.526 | 1.529 | 1.28E-01 | 2.49E-01 | -5.34 |
| C20orf144 | 0.179 | 6.042 | 1.528 | 1.28E-01 | 2.50E-01 | -5.342 |
| SLC35B4 | -0.17 | 7.555 | -1.528 | 1.28E-01 | 2.50E-01 | -5.342 |
| SH2D1A | -0.169 | 5.889 | -1.528 | 1.28E-01 | 2.50E-01 | -5.342 |
| CXCL10 | -0.522 | 6.758 | -1.528 | 1.28E-01 | 2.50E-01 | -5.343 |
| CD2BP2 | 0.148 | 9.008 | 1.527 | 1.28E-01 | 2.50E-01 | -5.344 |
| FAM47A | 0.163 | 4.82 | 1.526 | 1.28E-01 | 2.50E-01 | -5.344 |
| NLGN4Y | -0.272 | 5.507 | -1.527 | 1.28E-01 | 2.50E-01 | -5.345 |
| TFF2 | 0.152 | 5.562 | 1.525 | 1.29E-01 | 2.50E-01 | -5.346 |
| LHX9 | 0.144 | 4.659 | 1.525 | 1.29E-01 | 2.50E-01 | -5.346 |
| DVL2 | 0.158 | 8.743 | 1.524 | 1.29E-01 | 2.51E-01 | -5.347 |
| STXBP6 | -0.213 | 7.896 | -1.525 | 1.29E-01 | 2.51E-01 | -5.347 |
| PRSS23 | -0.213 | 8.726 | -1.525 | 1.29E-01 | 2.51E-01 | -5.348 |
| COPA | -0.148 | 10.17 | -1.523 | 1.29E-01 | 2.52E-01 | -5.351 |
| ROBO1 | -0.179 | 5.885 | -1.523 | 1.29E-01 | 2.52E-01 | -5.351 |
| PLVAP | 0.189 | 8.375 | 1.521 | 1.29E-01 | 2.52E-01 | -5.351 |
| ZDHHC16 | 0.111 | 9.124 | 1.521 | 1.30E-01 | 2.52E-01 | -5.351 |
| ZNF384 | 0.109 | 9.209 | 1.52 | 1.30E-01 | 2.52E-01 | -5.353 |
| CA5B | -0.139 | 6.289 | -1.521 | 1.30E-01 | 2.52E-01 | -5.353 |
| GPC2 | 0.153 | 6.58 | 1.52 | 1.30E-01 | 2.52E-01 | -5.353 |
| GP5 | -0.138 | 5.382 | -1.521 | 1.30E-01 | 2.52E-01 | -5.353 |
| HIST1H3I | -0.218 | 7.213 | -1.52 | 1.30E-01 | 2.52E-01 | -5.354 |
| SIX3 | 0.196 | 5.239 | 1.519 | 1.30E-01 | 2.52E-01 | -5.355 |
| PRRX2 | 0.162 | 7.236 | 1.519 | 1.30E-01 | 2.52E-01 | -5.355 |
| CEACAM4 | -0.254 | 7.492 | -1.52 | 1.30E-01 | 2.52E-01 | -5.355 |
| DHX32 | -0.141 | 9.285 | -1.519 | 1.30E-01 | 2.53E-01 | -5.356 |
| ACAT2 | 0.152 | 8.849 | 1.518 | 1.30E-01 | 2.53E-01 | -5.356 |
| GPD1L | 0.142 | 9.157 | 1.518 | 1.30E-01 | 2.53E-01 | -5.357 |
| PTGIR | 0.153 | 7.542 | 1.517 | 1.31E-01 | 2.53E-01 | -5.358 |
| EIF5 | -0.152 | 8.778 | -1.516 | 1.31E-01 | 2.54E-01 | -5.36 |
| NRTN | 0.211 | 5.793 | 1.515 | 1.31E-01 | 2.54E-01 | -5.361 |
| ADAMTS20 | 0.13 | 5.281 | 1.514 | 1.31E-01 | 2.54E-01 | -5.362 |
| RNPEPL1 | 0.135 | 9.161 | 1.514 | 1.31E-01 | 2.54E-01 | -5.363 |
| MOCS1 | 0.16 | 7.3 | 1.513 | 1.31E-01 | 2.54E-01 | -5.363 |
| KCND1 | -0.182 | 5.452 | -1.514 | 1.32E-01 | 2.54E-01 | -5.363 |
| DBP | 0.193 | 7.734 | 1.513 | 1.32E-01 | 2.54E-01 | -5.363 |
| GMPPA | 0.143 | 8.064 | 1.513 | 1.32E-01 | 2.54E-01 | -5.364 |
| SMOX | 0.202 | 8.426 | 1.513 | 1.32E-01 | 2.54E-01 | -5.364 |
| SSB | -0.143 | 10.129 | -1.513 | 1.32E-01 | 2.54E-01 | -5.364 |
| MLLT4 | -0.155 | 6.528 | -1.512 | 1.32E-01 | 2.55E-01 | -5.366 |
| PNLDC1 | -0.2 | 5.061 | -1.512 | 1.32E-01 | 2.55E-01 | -5.366 |
| ELOVL6 | 0.162 | 6.753 | 1.511 | 1.32E-01 | 2.55E-01 | -5.367 |
| DISP2 | 0.218 | 6.627 | 1.51 | 1.32E-01 | 2.55E-01 | -5.368 |
| NR4A2 | -0.214 | 5.876 | -1.51 | 1.33E-01 | 2.56E-01 | -5.369 |
| RIC3 | -0.182 | 5.291 | -1.51 | 1.33E-01 | 2.56E-01 | -5.37 |
| RNF135 | -0.104 | 8.78 | -1.509 | 1.33E-01 | 2.56E-01 | -5.371 |
| UBE2R2 | -0.112 | 8.584 | -1.509 | 1.33E-01 | 2.56E-01 | -5.371 |
| PNPLA5 | 0.191 | 6.209 | 1.508 | 1.33E-01 | 2.56E-01 | -5.371 |
| CYP2A7 | 0.207 | 5.19 | 1.508 | 1.33E-01 | 2.56E-01 | -5.371 |
| OPHN1 | -0.168 | 6.902 | -1.508 | 1.33E-01 | 2.56E-01 | -5.372 |
| MOCS2 | -0.139 | 7.904 | -1.507 | 1.33E-01 | 2.57E-01 | -5.374 |
| SH3BP4 | -0.182 | 8.567 | -1.507 | 1.33E-01 | 2.57E-01 | -5.374 |
| IREB2 | -0.133 | 8.381 | -1.507 | 1.33E-01 | 2.57E-01 | -5.374 |
| FCER1G | 0.195 | 9.676 | 1.505 | 1.34E-01 | 2.57E-01 | -5.376 |
| C7orf33 | 0.181 | 4.877 | 1.504 | 1.34E-01 | 2.57E-01 | -5.377 |
| IGFL2 | 0.19 | 5.993 | 1.503 | 1.34E-01 | 2.58E-01 | -5.378 |
| FRMPD1 | 0.225 | 5.243 | 1.503 | 1.34E-01 | 2.58E-01 | -5.378 |
| SERPINB12 | 0.193 | 4.828 | 1.502 | 1.34E-01 | 2.58E-01 | -5.379 |
| CHI3L2 | 0.32 | 6.623 | 1.502 | 1.34E-01 | 2.58E-01 | -5.38 |
| SIM2 | 0.13 | 5.241 | 1.502 | 1.34E-01 | 2.58E-01 | -5.38 |
| CDH6 | 0.249 | 5.583 | 1.502 | 1.35E-01 | 2.58E-01 | -5.38 |
| NPPA | 0.273 | 5.413 | 1.502 | 1.35E-01 | 2.58E-01 | -5.38 |
| RANGAP1 | -0.154 | 9.941 | -1.502 | 1.35E-01 | 2.58E-01 | -5.381 |
| NAGA | 0.134 | 8.519 | 1.501 | 1.35E-01 | 2.58E-01 | -5.382 |
| CABYR | -0.172 | 5.714 | -1.501 | 1.35E-01 | 2.59E-01 | -5.382 |
| ARFGEF2 | -0.137 | 7.71 | -1.5 | 1.35E-01 | 2.59E-01 | -5.384 |
| SEMA6B | 0.181 | 6.564 | 1.499 | 1.35E-01 | 2.59E-01 | -5.384 |
| ZNF16 | 0.134 | 6.756 | 1.499 | 1.35E-01 | 2.59E-01 | -5.384 |
| BAIAP3 | 0.193 | 6.171 | 1.499 | 1.35E-01 | 2.59E-01 | -5.384 |
| MAPK1 | -0.1 | 8.742 | -1.499 | 1.35E-01 | 2.59E-01 | -5.386 |
| UBE2L6 | 0.16 | 9.655 | 1.498 | 1.35E-01 | 2.59E-01 | -5.386 |
| COL9A1 | 0.174 | 5.342 | 1.497 | 1.36E-01 | 2.59E-01 | -5.386 |
| DNASE1L3 | 0.199 | 7.271 | 1.497 | 1.36E-01 | 2.60E-01 | -5.387 |
| NEU1 | 0.118 | 10.094 | 1.496 | 1.36E-01 | 2.60E-01 | -5.388 |
| RPL38 | 0.105 | 14.542 | 1.495 | 1.36E-01 | 2.60E-01 | -5.389 |
| MYH14 | 0.165 | 7.319 | 1.495 | 1.36E-01 | 2.60E-01 | -5.39 |
| RAG2 | 0.149 | 4.741 | 1.495 | 1.36E-01 | 2.60E-01 | -5.39 |
| RGS10 | 0.156 | 10.348 | 1.493 | 1.37E-01 | 2.61E-01 | -5.393 |
| FASN | 0.241 | 8.837 | 1.492 | 1.37E-01 | 2.62E-01 | -5.394 |
| PADI2 | -0.154 | 5.933 | -1.492 | 1.37E-01 | 2.62E-01 | -5.396 |
| GYPA | 0.276 | 5.968 | 1.49 | 1.37E-01 | 2.62E-01 | -5.397 |
| TRAM2 | -0.15 | 10.066 | -1.491 | 1.38E-01 | 2.63E-01 | -5.398 |
| TOR2A | 0.154 | 7.776 | 1.489 | 1.38E-01 | 2.63E-01 | -5.399 |
| NFKBIL1 | 0.188 | 7.864 | 1.489 | 1.38E-01 | 2.63E-01 | -5.399 |
| RAE1 | -0.088 | 9.15 | -1.489 | 1.38E-01 | 2.63E-01 | -5.4 |
| PSMB1 | 0.095 | 13.244 | 1.488 | 1.38E-01 | 2.63E-01 | -5.4 |
| PPAT | -0.136 | 8.391 | -1.488 | 1.38E-01 | 2.63E-01 | -5.401 |
| PSMC3 | 0.128 | 10.965 | 1.487 | 1.38E-01 | 2.63E-01 | -5.401 |
| FSTL3 | 0.324 | 9.833 | 1.487 | 1.38E-01 | 2.63E-01 | -5.402 |
| UROC1 | -0.135 | 5.291 | -1.488 | 1.38E-01 | 2.63E-01 | -5.402 |
| TPH2 | -0.123 | 4.617 | -1.487 | 1.39E-01 | 2.64E-01 | -5.402 |
| NSUN5 | 0.155 | 8.96 | 1.486 | 1.39E-01 | 2.64E-01 | -5.403 |
| CARD14 | -0.146 | 6.2 | -1.487 | 1.39E-01 | 2.64E-01 | -5.403 |
| TRIM9 | -0.133 | 5.108 | -1.486 | 1.39E-01 | 2.64E-01 | -5.404 |
| IL1F10 | 0.205 | 5.62 | 1.484 | 1.39E-01 | 2.65E-01 | -5.406 |
| PSMD5 | 0.124 | 7.805 | 1.483 | 1.39E-01 | 2.65E-01 | -5.407 |
| S100A5 | 0.259 | 5.949 | 1.483 | 1.39E-01 | 2.65E-01 | -5.407 |
| ZNF445 | -0.138 | 6.452 | -1.484 | 1.39E-01 | 2.65E-01 | -5.407 |
| SPP1 | -0.332 | 10.304 | -1.483 | 1.40E-01 | 2.65E-01 | -5.408 |
| TRMT1 | 0.166 | 8.976 | 1.481 | 1.40E-01 | 2.65E-01 | -5.409 |
| GAS1 | -0.304 | 8.567 | -1.482 | 1.40E-01 | 2.66E-01 | -5.41 |
| PDAP1 | 0.125 | 7.927 | 1.48 | 1.40E-01 | 2.66E-01 | -5.412 |
| KRT12 | 0.153 | 4.763 | 1.48 | 1.40E-01 | 2.66E-01 | -5.412 |
| MAP3K10 | 0.147 | 7.127 | 1.479 | 1.40E-01 | 2.66E-01 | -5.413 |
| GPX3 | 0.24 | 9.26 | 1.479 | 1.41E-01 | 2.67E-01 | -5.413 |
| KIAA2018 | -0.158 | 6.59 | -1.479 | 1.41E-01 | 2.67E-01 | -5.414 |
| SAGE1 | 0.18 | 4.811 | 1.478 | 1.41E-01 | 2.67E-01 | -5.414 |
| OSBP | -0.179 | 9.607 | -1.478 | 1.41E-01 | 2.67E-01 | -5.415 |
| IL13RA2 | -0.348 | 5.725 | -1.478 | 1.41E-01 | 2.67E-01 | -5.415 |
| TAF5L | -0.143 | 6.481 | -1.478 | 1.41E-01 | 2.67E-01 | -5.415 |
| TPST1 | 0.214 | 9.4 | 1.477 | 1.41E-01 | 2.67E-01 | -5.416 |
| LAD1 | 0.232 | 7.275 | 1.476 | 1.41E-01 | 2.67E-01 | -5.417 |
| HOXC4 | 0.125 | 6.149 | 1.476 | 1.41E-01 | 2.67E-01 | -5.417 |
| MATN1 | 0.136 | 5.491 | 1.476 | 1.41E-01 | 2.67E-01 | -5.417 |
| PRRG2 | 0.177 | 6.137 | 1.475 | 1.42E-01 | 2.68E-01 | -5.419 |
| GMEB1 | 0.135 | 8.304 | 1.474 | 1.42E-01 | 2.68E-01 | -5.42 |
| MUSK | 0.161 | 6.26 | 1.473 | 1.42E-01 | 2.68E-01 | -5.421 |
| ZNF212 | 0.104 | 9.093 | 1.473 | 1.42E-01 | 2.68E-01 | -5.421 |
| SLC6A20 | 0.175 | 4.988 | 1.472 | 1.42E-01 | 2.69E-01 | -5.423 |
| MAGEC2 | 0.172 | 4.963 | 1.472 | 1.42E-01 | 2.69E-01 | -5.423 |
| RHOH | -0.184 | 6.546 | -1.472 | 1.43E-01 | 2.69E-01 | -5.424 |
| RUNX3 | 0.192 | 6.765 | 1.471 | 1.43E-01 | 2.69E-01 | -5.425 |
| DCBLD2 | 0.156 | 7.342 | 1.471 | 1.43E-01 | 2.69E-01 | -5.425 |
| ANG | 0.198 | 8.154 | 1.471 | 1.43E-01 | 2.69E-01 | -5.425 |
| CLK3 | 0.161 | 9.592 | 1.47 | 1.43E-01 | 2.69E-01 | -5.425 |
| FZD2 | 0.195 | 8.49 | 1.468 | 1.43E-01 | 2.70E-01 | -5.428 |
| RXRG | -0.16 | 4.98 | -1.469 | 1.43E-01 | 2.70E-01 | -5.429 |
| C9orf84 | -0.19 | 4.901 | -1.469 | 1.44E-01 | 2.70E-01 | -5.429 |
| EWSR1 | -0.109 | 9.614 | -1.468 | 1.44E-01 | 2.70E-01 | -5.43 |
| CPNE1 | 0.18 | 9.924 | 1.467 | 1.44E-01 | 2.71E-01 | -5.43 |
| SPAG5 | 0.173 | 8.225 | 1.466 | 1.44E-01 | 2.71E-01 | -5.431 |
| FGF19 | 0.132 | 5.605 | 1.466 | 1.44E-01 | 2.71E-01 | -5.432 |
| STAT1 | -0.198 | 9.807 | -1.467 | 1.44E-01 | 2.71E-01 | -5.432 |
| MXD1 | -0.203 | 8.749 | -1.467 | 1.44E-01 | 2.71E-01 | -5.432 |
| NPB | 0.25 | 7.25 | 1.465 | 1.44E-01 | 2.71E-01 | -5.433 |
| PPP3CC | 0.135 | 9.07 | 1.464 | 1.44E-01 | 2.71E-01 | -5.434 |
| N4BP1 | -0.11 | 8.54 | -1.465 | 1.45E-01 | 2.72E-01 | -5.435 |
| PITX3 | 0.162 | 6.987 | 1.463 | 1.45E-01 | 2.72E-01 | -5.435 |
| GIP | 0.175 | 5.071 | 1.463 | 1.45E-01 | 2.72E-01 | -5.435 |
| FUT6 | 0.124 | 7.396 | 1.463 | 1.45E-01 | 2.72E-01 | -5.436 |
| FKBP4 | 0.125 | 9.247 | 1.462 | 1.45E-01 | 2.72E-01 | -5.437 |
| GNL3 | -0.137 | 9.393 | -1.463 | 1.45E-01 | 2.72E-01 | -5.437 |
| CDH20 | -0.198 | 4.749 | -1.463 | 1.45E-01 | 2.72E-01 | -5.437 |
| KLK2 | 0.156 | 5.497 | 1.462 | 1.45E-01 | 2.72E-01 | -5.437 |
| SACS | -0.157 | 6.705 | -1.462 | 1.45E-01 | 2.72E-01 | -5.438 |
| CTPS2 | 0.125 | 6.942 | 1.461 | 1.45E-01 | 2.72E-01 | -5.439 |
| PADI4 | 0.169 | 5.841 | 1.46 | 1.45E-01 | 2.73E-01 | -5.439 |
| LIMD1 | -0.176 | 6.482 | -1.461 | 1.46E-01 | 2.73E-01 | -5.44 |
| TM4SF5 | 0.241 | 6.952 | 1.459 | 1.46E-01 | 2.73E-01 | -5.441 |
| DEPDC5 | 0.11 | 7.236 | 1.459 | 1.46E-01 | 2.73E-01 | -5.441 |
| ABCG8 | 0.138 | 4.888 | 1.459 | 1.46E-01 | 2.73E-01 | -5.442 |
| CPEB1 | 0.187 | 5.402 | 1.459 | 1.46E-01 | 2.73E-01 | -5.442 |
| GTF2E2 | -0.112 | 9.713 | -1.459 | 1.46E-01 | 2.73E-01 | -5.442 |
| TNK2 | 0.127 | 8.165 | 1.458 | 1.46E-01 | 2.73E-01 | -5.442 |
| GABRA3 | 0.179 | 5.843 | 1.458 | 1.46E-01 | 2.73E-01 | -5.442 |
| PLEKHB2 | -0.126 | 9.386 | -1.459 | 1.46E-01 | 2.73E-01 | -5.443 |
| UTY | -0.296 | 5.548 | -1.459 | 1.46E-01 | 2.73E-01 | -5.443 |
| CCNDBP1 | -0.134 | 10.525 | -1.458 | 1.46E-01 | 2.74E-01 | -5.444 |
| DHX40 | -0.143 | 6.771 | -1.458 | 1.47E-01 | 2.74E-01 | -5.445 |
| CSTL1 | 0.153 | 5.116 | 1.457 | 1.47E-01 | 2.74E-01 | -5.445 |
| GARS | 0.144 | 9.582 | 1.457 | 1.47E-01 | 2.74E-01 | -5.445 |
| GLIPR1 | -0.242 | 8.693 | -1.457 | 1.47E-01 | 2.74E-01 | -5.445 |
| NETO1 | 0.103 | 5.104 | 1.456 | 1.47E-01 | 2.74E-01 | -5.445 |
| HDLBP | 0.129 | 8.869 | 1.456 | 1.47E-01 | 2.74E-01 | -5.446 |
| GPX2 | 0.225 | 6.266 | 1.456 | 1.47E-01 | 2.74E-01 | -5.446 |
| SPRR1B | 0.212 | 5.683 | 1.455 | 1.47E-01 | 2.74E-01 | -5.447 |
| NUP133 | 0.102 | 9.09 | 1.455 | 1.47E-01 | 2.74E-01 | -5.447 |
| NTSR2 | 0.165 | 4.98 | 1.455 | 1.47E-01 | 2.74E-01 | -5.447 |
| NAV2 | -0.137 | 6.483 | -1.456 | 1.47E-01 | 2.74E-01 | -5.448 |
| PTPN22 | -0.175 | 5.631 | -1.455 | 1.47E-01 | 2.74E-01 | -5.449 |
| FCGBP | 0.247 | 8.413 | 1.454 | 1.47E-01 | 2.74E-01 | -5.449 |
| SLC30A4 | -0.161 | 6.125 | -1.455 | 1.47E-01 | 2.74E-01 | -5.449 |
| PHTF1 | -0.155 | 7.498 | -1.454 | 1.48E-01 | 2.75E-01 | -5.45 |
| CECR6 | -0.2 | 6.722 | -1.452 | 1.48E-01 | 2.75E-01 | -5.452 |
| TMEM41A | 0.111 | 7.587 | 1.45 | 1.48E-01 | 2.76E-01 | -5.454 |
| SLC43A2 | 0.182 | 10.345 | 1.45 | 1.48E-01 | 2.76E-01 | -5.454 |
| SLC25A21 | -0.178 | 4.786 | -1.451 | 1.48E-01 | 2.76E-01 | -5.455 |
| TLK2 | 0.127 | 8.138 | 1.449 | 1.49E-01 | 2.76E-01 | -5.456 |
| STARD6 | 0.175 | 4.611 | 1.448 | 1.49E-01 | 2.77E-01 | -5.457 |
| TNFRSF19 | 0.141 | 5.343 | 1.447 | 1.49E-01 | 2.77E-01 | -5.458 |
| PTGDS | 0.202 | 9.899 | 1.446 | 1.50E-01 | 2.78E-01 | -5.46 |
| SP2 | -0.135 | 7.418 | -1.446 | 1.50E-01 | 2.78E-01 | -5.46 |
| GAD1 | 0.171 | 5.465 | 1.445 | 1.50E-01 | 2.78E-01 | -5.461 |
| EPHX1 | 0.193 | 7.511 | 1.445 | 1.50E-01 | 2.78E-01 | -5.461 |
| DHH | 0.196 | 6.48 | 1.445 | 1.50E-01 | 2.78E-01 | -5.461 |
| ATRNL1 | -0.145 | 5.236 | -1.445 | 1.50E-01 | 2.78E-01 | -5.463 |
| TPPP | -0.167 | 5.637 | -1.444 | 1.50E-01 | 2.79E-01 | -5.463 |
| TRIM38 | 0.133 | 8.258 | 1.443 | 1.50E-01 | 2.79E-01 | -5.464 |
| ALG2 | -0.099 | 8.478 | -1.444 | 1.50E-01 | 2.79E-01 | -5.464 |
| SPO11 | 0.175 | 4.813 | 1.441 | 1.51E-01 | 2.79E-01 | -5.466 |
| TAGAP | -0.169 | 6.817 | -1.442 | 1.51E-01 | 2.79E-01 | -5.467 |
| WNT8A | -0.128 | 4.614 | -1.442 | 1.51E-01 | 2.79E-01 | -5.467 |
| PGR | 0.185 | 5.805 | 1.44 | 1.51E-01 | 2.80E-01 | -5.468 |
| MRPS18C | -0.135 | 9.351 | -1.44 | 1.51E-01 | 2.80E-01 | -5.47 |
| SOD3 | 0.245 | 7.865 | 1.438 | 1.52E-01 | 2.81E-01 | -5.471 |
| ALCAM | -0.176 | 7.98 | -1.439 | 1.52E-01 | 2.81E-01 | -5.471 |
| POLG | 0.095 | 9.601 | 1.438 | 1.52E-01 | 2.81E-01 | -5.471 |
| RASSF3 | -0.145 | 6.862 | -1.438 | 1.52E-01 | 2.81E-01 | -5.472 |
| CD80 | 0.167 | 5.471 | 1.437 | 1.52E-01 | 2.81E-01 | -5.472 |
| CETP | 0.228 | 7.347 | 1.437 | 1.52E-01 | 2.81E-01 | -5.472 |
| FAIM2 | 0.174 | 5.766 | 1.437 | 1.52E-01 | 2.81E-01 | -5.472 |
| SCARA3 | 0.159 | 8.477 | 1.437 | 1.52E-01 | 2.81E-01 | -5.472 |
| SLC39A14 | 0.166 | 7.411 | 1.435 | 1.53E-01 | 2.82E-01 | -5.475 |
| CD3E | 0.143 | 6.228 | 1.435 | 1.53E-01 | 2.82E-01 | -5.476 |
| PTK2 | -0.11 | 9.33 | -1.436 | 1.53E-01 | 2.82E-01 | -5.476 |
| ATP2A2 | -0.128 | 8.62 | -1.435 | 1.53E-01 | 2.82E-01 | -5.476 |
| TCEAL8 | -0.127 | 9.95 | -1.435 | 1.53E-01 | 2.82E-01 | -5.477 |
| CDC42EP5 | 0.166 | 10.764 | 1.434 | 1.53E-01 | 2.82E-01 | -5.477 |
| LMNB2 | 0.155 | 9.072 | 1.432 | 1.53E-01 | 2.83E-01 | -5.479 |
| TARS | -0.205 | 10.274 | -1.433 | 1.53E-01 | 2.83E-01 | -5.479 |
| VSNL1 | 0.205 | 6.184 | 1.432 | 1.53E-01 | 2.83E-01 | -5.479 |
| DAO | 0.167 | 5.073 | 1.432 | 1.54E-01 | 2.83E-01 | -5.48 |
| TPMT | -0.141 | 7.35 | -1.433 | 1.54E-01 | 2.83E-01 | -5.48 |
| CKAP2 | -0.125 | 7.556 | -1.433 | 1.54E-01 | 2.83E-01 | -5.48 |
| PTPRN2 | 0.129 | 6.467 | 1.431 | 1.54E-01 | 2.83E-01 | -5.48 |
| LARGE | 0.206 | 6.932 | 1.431 | 1.54E-01 | 2.83E-01 | -5.481 |
| RHOJ | 0.14 | 6.235 | 1.431 | 1.54E-01 | 2.83E-01 | -5.481 |
| GRB10 | -0.17 | 8.633 | -1.43 | 1.54E-01 | 2.84E-01 | -5.484 |
| CLC | 0.269 | 6.918 | 1.429 | 1.54E-01 | 2.84E-01 | -5.484 |
| PRPF8 | 0.135 | 10.738 | 1.428 | 1.54E-01 | 2.84E-01 | -5.484 |
| NPHP4 | -0.159 | 6.669 | -1.428 | 1.55E-01 | 2.85E-01 | -5.487 |
| FBLN5 | -0.169 | 8.72 | -1.427 | 1.55E-01 | 2.85E-01 | -5.488 |
| ILDR1 | -0.183 | 8.208 | -1.426 | 1.55E-01 | 2.85E-01 | -5.489 |
| KCNJ3 | 0.121 | 4.716 | 1.425 | 1.55E-01 | 2.86E-01 | -5.489 |
| EFNA4 | 0.141 | 7.745 | 1.425 | 1.56E-01 | 2.86E-01 | -5.49 |
| TRPM8 | -0.11 | 4.633 | -1.425 | 1.56E-01 | 2.86E-01 | -5.49 |
| KLF8 | -0.145 | 7.062 | -1.425 | 1.56E-01 | 2.86E-01 | -5.491 |
| RORA | -0.196 | 7.642 | -1.424 | 1.56E-01 | 2.86E-01 | -5.491 |
| GPR35 | -0.113 | 6.557 | -1.424 | 1.56E-01 | 2.86E-01 | -5.492 |
| APBB1 | 0.159 | 7.207 | 1.423 | 1.56E-01 | 2.86E-01 | -5.492 |
| BTNL8 | 0.276 | 5.931 | 1.423 | 1.56E-01 | 2.86E-01 | -5.492 |
| KRT3 | 0.215 | 7.518 | 1.422 | 1.56E-01 | 2.86E-01 | -5.493 |
| RPS6KA6 | -0.195 | 5.453 | -1.423 | 1.56E-01 | 2.86E-01 | -5.493 |
| ADORA1 | 0.195 | 6.338 | 1.421 | 1.56E-01 | 2.86E-01 | -5.494 |
| RAB3D | 0.143 | 7.012 | 1.421 | 1.56E-01 | 2.86E-01 | -5.494 |
| HIST3H3 | -0.117 | 9.388 | -1.422 | 1.57E-01 | 2.86E-01 | -5.494 |
| FLNB | -0.179 | 9.145 | -1.422 | 1.57E-01 | 2.86E-01 | -5.494 |
| PPIL6 | -0.145 | 5.2 | -1.422 | 1.57E-01 | 2.86E-01 | -5.494 |
| GRIK1 | 0.159 | 4.864 | 1.421 | 1.57E-01 | 2.86E-01 | -5.495 |
| GPR158 | -0.152 | 5.882 | -1.422 | 1.57E-01 | 2.86E-01 | -5.495 |
| COMMD5 | 0.092 | 8.229 | 1.421 | 1.57E-01 | 2.86E-01 | -5.495 |
| SALL1 | -0.164 | 5.544 | -1.421 | 1.57E-01 | 2.86E-01 | -5.495 |
| CSN3 | 0.147 | 4.833 | 1.42 | 1.57E-01 | 2.86E-01 | -5.495 |
| CD8A | -0.184 | 6.781 | -1.42 | 1.57E-01 | 2.87E-01 | -5.497 |
| EMX2 | -0.268 | 5.365 | -1.42 | 1.57E-01 | 2.87E-01 | -5.497 |
| PHLDA2 | 0.208 | 10.217 | 1.418 | 1.58E-01 | 2.88E-01 | -5.499 |
| RPP40 | -0.127 | 8.414 | -1.418 | 1.58E-01 | 2.88E-01 | -5.5 |
| HOXC6 | 0.139 | 6.907 | 1.416 | 1.58E-01 | 2.88E-01 | -5.501 |
| EPS15L1 | 0.117 | 7.631 | 1.416 | 1.58E-01 | 2.88E-01 | -5.501 |
| DEFA6 | -0.147 | 4.795 | -1.417 | 1.58E-01 | 2.88E-01 | -5.502 |
| PLEKHH1 | -0.199 | 6.855 | -1.416 | 1.58E-01 | 2.89E-01 | -5.503 |
| SLC12A9 | 0.128 | 9.737 | 1.415 | 1.58E-01 | 2.89E-01 | -5.503 |
| HIST1H4I | 0.174 | 6.918 | 1.415 | 1.58E-01 | 2.89E-01 | -5.503 |
| SEMA4D | -0.116 | 7.601 | -1.416 | 1.58E-01 | 2.89E-01 | -5.503 |
| PDZRN4 | 0.143 | 6.161 | 1.414 | 1.59E-01 | 2.89E-01 | -5.504 |
| COX4I2 | 0.182 | 8.592 | 1.414 | 1.59E-01 | 2.89E-01 | -5.504 |
| ALK | 0.113 | 4.817 | 1.412 | 1.59E-01 | 2.90E-01 | -5.507 |
| NOL8 | -0.137 | 9.161 | -1.413 | 1.59E-01 | 2.90E-01 | -5.507 |
| ARFGAP1 | -0.12 | 9.046 | -1.413 | 1.59E-01 | 2.90E-01 | -5.507 |
| CCR1 | -0.176 | 7.73 | -1.411 | 1.60E-01 | 2.91E-01 | -5.509 |
| ING5 | -0.112 | 6.838 | -1.409 | 1.60E-01 | 2.91E-01 | -5.512 |
| ATAD3B | 0.127 | 7.798 | 1.408 | 1.60E-01 | 2.92E-01 | -5.513 |
| SLC18A2 | -0.174 | 4.911 | -1.409 | 1.61E-01 | 2.92E-01 | -5.513 |
| CLNS1A | 0.103 | 10.553 | 1.407 | 1.61E-01 | 2.92E-01 | -5.514 |
| BTBD6 | -0.105 | 10.446 | -1.408 | 1.61E-01 | 2.92E-01 | -5.514 |
| NUDT6 | 0.133 | 7.052 | 1.406 | 1.61E-01 | 2.92E-01 | -5.515 |
| CBLN4 | 0.12 | 4.811 | 1.406 | 1.61E-01 | 2.92E-01 | -5.515 |
| ADAMTS17 | 0.156 | 5.277 | 1.406 | 1.61E-01 | 2.92E-01 | -5.515 |
| SUCLG1 | 0.097 | 10.257 | 1.405 | 1.61E-01 | 2.93E-01 | -5.516 |
| SORBS1 | 0.148 | 7.53 | 1.405 | 1.61E-01 | 2.93E-01 | -5.517 |
| NR0B2 | 0.165 | 5.009 | 1.404 | 1.62E-01 | 2.93E-01 | -5.518 |
| TPI1 | 0.113 | 12.111 | 1.404 | 1.62E-01 | 2.93E-01 | -5.518 |
| MORF4L2 | -0.123 | 11.114 | -1.404 | 1.62E-01 | 2.93E-01 | -5.519 |
| PLA2G12B | 0.175 | 5.326 | 1.402 | 1.62E-01 | 2.94E-01 | -5.521 |
| DGKG | 0.167 | 6.829 | 1.401 | 1.62E-01 | 2.94E-01 | -5.522 |
| COL6A2 | 0.192 | 8.938 | 1.401 | 1.63E-01 | 2.94E-01 | -5.522 |
| NUDT13 | -0.171 | 6.322 | -1.401 | 1.63E-01 | 2.95E-01 | -5.523 |
| ZNF596 | -0.143 | 5.917 | -1.401 | 1.63E-01 | 2.95E-01 | -5.523 |
| SLC9A1 | -0.139 | 8.844 | -1.401 | 1.63E-01 | 2.95E-01 | -5.523 |
| KIN | -0.13 | 7.73 | -1.4 | 1.63E-01 | 2.95E-01 | -5.524 |
| C1GALT1C1 | -0.137 | 8.564 | -1.4 | 1.63E-01 | 2.95E-01 | -5.525 |
| NDUFS6 | 0.125 | 10.158 | 1.398 | 1.63E-01 | 2.95E-01 | -5.526 |
| ZNF425 | -0.138 | 6.632 | -1.398 | 1.64E-01 | 2.96E-01 | -5.527 |
| CETN2 | 0.118 | 10.036 | 1.397 | 1.64E-01 | 2.96E-01 | -5.528 |
| PDCD7 | -0.116 | 9.532 | -1.397 | 1.64E-01 | 2.96E-01 | -5.529 |
| PQLC1 | 0.115 | 9.473 | 1.394 | 1.65E-01 | 2.97E-01 | -5.531 |
| STAT4 | -0.215 | 7.449 | -1.395 | 1.65E-01 | 2.97E-01 | -5.532 |
| LIN7A | -0.199 | 6.979 | -1.394 | 1.65E-01 | 2.97E-01 | -5.532 |
| ATP10D | -0.129 | 8.47 | -1.393 | 1.65E-01 | 2.98E-01 | -5.534 |
| WBSCR17 | 0.231 | 6.478 | 1.392 | 1.65E-01 | 2.98E-01 | -5.535 |
| CSNK2A1 | 0.109 | 8.297 | 1.391 | 1.65E-01 | 2.98E-01 | -5.535 |
| ACADS | 0.139 | 7.617 | 1.39 | 1.66E-01 | 2.99E-01 | -5.537 |
| PLXNB3 | 0.165 | 5.283 | 1.389 | 1.66E-01 | 2.99E-01 | -5.538 |
| TMLHE | 0.11 | 7.056 | 1.388 | 1.67E-01 | 3.00E-01 | -5.54 |
| ARIH1 | -0.1 | 8.28 | -1.388 | 1.67E-01 | 3.00E-01 | -5.54 |
| DGAT1 | 0.118 | 8.27 | 1.387 | 1.67E-01 | 3.00E-01 | -5.541 |
| ILKAP | -0.096 | 8.203 | -1.387 | 1.67E-01 | 3.01E-01 | -5.542 |
| SPI1 | 0.132 | 8.328 | 1.386 | 1.67E-01 | 3.01E-01 | -5.542 |
| CIAPIN1 | -0.091 | 9.021 | -1.387 | 1.67E-01 | 3.01E-01 | -5.543 |
| SRY | -0.147 | 4.839 | -1.387 | 1.67E-01 | 3.01E-01 | -5.543 |
| WFDC5 | 0.157 | 5.776 | 1.385 | 1.67E-01 | 3.01E-01 | -5.543 |
| ETV3 | 0.12 | 6.931 | 1.384 | 1.67E-01 | 3.01E-01 | -5.544 |
| KIAA0556 | 0.137 | 7.243 | 1.384 | 1.68E-01 | 3.01E-01 | -5.544 |
| CALB2 | 0.17 | 5.053 | 1.383 | 1.68E-01 | 3.02E-01 | -5.546 |
| SLC30A10 | -0.179 | 4.831 | -1.382 | 1.68E-01 | 3.03E-01 | -5.549 |
| TAGLN2 | -0.131 | 11.426 | -1.382 | 1.69E-01 | 3.03E-01 | -5.549 |
| PRCP | -0.125 | 11.684 | -1.382 | 1.69E-01 | 3.03E-01 | -5.549 |
| IER2 | -0.134 | 11.375 | -1.382 | 1.69E-01 | 3.03E-01 | -5.549 |
| C20orf96 | 0.187 | 5.504 | 1.38 | 1.69E-01 | 3.03E-01 | -5.55 |
| OSBPL3 | 0.129 | 7.311 | 1.38 | 1.69E-01 | 3.03E-01 | -5.551 |
| QKI | -0.161 | 7.429 | -1.38 | 1.69E-01 | 3.04E-01 | -5.552 |
| OR7A5 | 0.155 | 4.722 | 1.378 | 1.69E-01 | 3.04E-01 | -5.553 |
| MOGAT1 | -0.175 | 4.865 | -1.379 | 1.69E-01 | 3.04E-01 | -5.553 |
| MYO1D | 0.137 | 6.461 | 1.378 | 1.70E-01 | 3.04E-01 | -5.553 |
| NPM3 | 0.15 | 8.297 | 1.378 | 1.70E-01 | 3.04E-01 | -5.553 |
| TGM3 | 0.21 | 5.98 | 1.378 | 1.70E-01 | 3.04E-01 | -5.553 |
| PLEKHA8 | -0.141 | 6.162 | -1.379 | 1.70E-01 | 3.04E-01 | -5.553 |
| CD5 | 0.229 | 6.164 | 1.377 | 1.70E-01 | 3.04E-01 | -5.554 |
| ACE | 0.118 | 6.562 | 1.377 | 1.70E-01 | 3.04E-01 | -5.554 |
| FUT3 | 0.215 | 5.371 | 1.377 | 1.70E-01 | 3.04E-01 | -5.554 |
| USH1C | 0.156 | 5.12 | 1.376 | 1.70E-01 | 3.04E-01 | -5.555 |
| PSD | -0.154 | 6.149 | -1.377 | 1.70E-01 | 3.04E-01 | -5.555 |
| FBXW8 | 0.109 | 6.936 | 1.376 | 1.70E-01 | 3.04E-01 | -5.555 |
| SLC5A2 | 0.161 | 6.914 | 1.376 | 1.70E-01 | 3.04E-01 | -5.555 |
| IFNAR1 | -0.12 | 8.278 | -1.376 | 1.70E-01 | 3.05E-01 | -5.557 |
| SOD1 | -0.118 | 13.426 | -1.376 | 1.70E-01 | 3.05E-01 | -5.557 |
| FUT7 | 0.193 | 6.156 | 1.374 | 1.71E-01 | 3.05E-01 | -5.558 |
| DNPEP | 0.11 | 8.797 | 1.374 | 1.71E-01 | 3.05E-01 | -5.558 |
| MFI2 | 0.159 | 5.698 | 1.374 | 1.71E-01 | 3.05E-01 | -5.558 |
| SERPINB2 | -0.251 | 10.222 | -1.375 | 1.71E-01 | 3.05E-01 | -5.558 |
| RAB35 | 0.168 | 9.756 | 1.374 | 1.71E-01 | 3.05E-01 | -5.558 |
| COMMD6 | 0.152 | 8.487 | 1.374 | 1.71E-01 | 3.05E-01 | -5.558 |
| NVL | 0.164 | 8.626 | 1.374 | 1.71E-01 | 3.05E-01 | -5.559 |
| OR3A3 | 0.174 | 5.829 | 1.373 | 1.71E-01 | 3.05E-01 | -5.559 |
| LEPROT | -0.136 | 10.673 | -1.373 | 1.71E-01 | 3.06E-01 | -5.561 |
| COL7A1 | -0.178 | 8.187 | -1.372 | 1.72E-01 | 3.06E-01 | -5.562 |
| ATCAY | 0.175 | 5.848 | 1.37 | 1.72E-01 | 3.06E-01 | -5.563 |
| GANAB | 0.112 | 10.163 | 1.37 | 1.72E-01 | 3.06E-01 | -5.563 |
| CDK5R2 | 0.227 | 7.174 | 1.37 | 1.72E-01 | 3.06E-01 | -5.563 |
| HSPB3 | 0.274 | 5.306 | 1.37 | 1.72E-01 | 3.06E-01 | -5.564 |
| TEAD4 | 0.094 | 7.59 | 1.37 | 1.72E-01 | 3.06E-01 | -5.564 |
| ANXA6 | -0.185 | 8.998 | -1.371 | 1.72E-01 | 3.06E-01 | -5.564 |
| AP4E1 | -0.116 | 7.205 | -1.37 | 1.72E-01 | 3.06E-01 | -5.564 |
| HSD11B1 | -0.3 | 8.24 | -1.37 | 1.72E-01 | 3.07E-01 | -5.565 |
| ZMAT1 | -0.142 | 6.498 | -1.368 | 1.73E-01 | 3.07E-01 | -5.567 |
| SOS1 | 0.159 | 6.675 | 1.366 | 1.73E-01 | 3.08E-01 | -5.569 |
| MSX1 | 0.177 | 7.78 | 1.366 | 1.73E-01 | 3.08E-01 | -5.569 |
| USP28 | -0.158 | 5.951 | -1.367 | 1.73E-01 | 3.08E-01 | -5.569 |
| FBXO30 | -0.107 | 8.881 | -1.367 | 1.73E-01 | 3.08E-01 | -5.569 |
| SQRDL | -0.154 | 9.971 | -1.367 | 1.73E-01 | 3.08E-01 | -5.57 |
| VNN3 | -0.27 | 6.308 | -1.366 | 1.73E-01 | 3.08E-01 | -5.57 |
| MCPH1 | -0.12 | 6.972 | -1.366 | 1.73E-01 | 3.08E-01 | -5.57 |
| SLC6A3 | 0.139 | 5.579 | 1.365 | 1.73E-01 | 3.08E-01 | -5.57 |
| RDH8 | 0.164 | 5.453 | 1.364 | 1.74E-01 | 3.09E-01 | -5.572 |
| TWIST2 | 0.186 | 7.35 | 1.363 | 1.74E-01 | 3.09E-01 | -5.573 |
| GUCY2F | -0.169 | 5.002 | -1.364 | 1.74E-01 | 3.09E-01 | -5.573 |
| WDR36 | 0.112 | 8.149 | 1.362 | 1.74E-01 | 3.09E-01 | -5.574 |
| EPHB2 | 0.169 | 7.174 | 1.362 | 1.74E-01 | 3.09E-01 | -5.574 |
| DHFRL1 | 0.15 | 6.855 | 1.362 | 1.74E-01 | 3.09E-01 | -5.574 |
| CTAGE1 | -0.1 | 5.847 | -1.363 | 1.75E-01 | 3.09E-01 | -5.575 |
| USP9X | -0.118 | 10.489 | -1.362 | 1.75E-01 | 3.09E-01 | -5.575 |
| FANCB | -0.13 | 6.164 | -1.362 | 1.75E-01 | 3.09E-01 | -5.575 |
| F13B | 0.148 | 4.769 | 1.361 | 1.75E-01 | 3.09E-01 | -5.576 |
| DRD5 | 0.175 | 5.164 | 1.361 | 1.75E-01 | 3.09E-01 | -5.576 |
| DNTTIP1 | -0.085 | 10.28 | -1.361 | 1.75E-01 | 3.10E-01 | -5.577 |
| SNRPF | 0.145 | 10.824 | 1.36 | 1.75E-01 | 3.10E-01 | -5.577 |
| ADCY3 | -0.14 | 11.067 | -1.36 | 1.75E-01 | 3.10E-01 | -5.578 |
| CHRNA5 | 0.167 | 5.79 | 1.359 | 1.75E-01 | 3.10E-01 | -5.578 |
| EEF2 | 0.117 | 13.164 | 1.359 | 1.76E-01 | 3.10E-01 | -5.579 |
| GATA3 | 0.125 | 9.412 | 1.357 | 1.76E-01 | 3.11E-01 | -5.581 |
| WNT8B | 0.161 | 4.788 | 1.357 | 1.76E-01 | 3.11E-01 | -5.581 |
| DOK3 | -0.129 | 7.378 | -1.358 | 1.76E-01 | 3.11E-01 | -5.581 |
| FOXP3 | 0.151 | 5.445 | 1.356 | 1.76E-01 | 3.11E-01 | -5.582 |
| ANGPTL2 | 0.185 | 7.767 | 1.356 | 1.76E-01 | 3.12E-01 | -5.583 |
| ARHGEF2 | 0.123 | 9.274 | 1.355 | 1.77E-01 | 3.12E-01 | -5.583 |
| MMP12 | -0.31 | 5.753 | -1.356 | 1.77E-01 | 3.12E-01 | -5.584 |
| TPO | -0.173 | 5.222 | -1.356 | 1.77E-01 | 3.12E-01 | -5.584 |
| CD72 | 0.175 | 6.634 | 1.355 | 1.77E-01 | 3.12E-01 | -5.584 |
| SUMO1 | -0.106 | 8.652 | -1.356 | 1.77E-01 | 3.12E-01 | -5.584 |
| PCDH9 | -0.169 | 5.191 | -1.355 | 1.77E-01 | 3.12E-01 | -5.584 |
| ATP1B1 | -0.163 | 9.552 | -1.355 | 1.77E-01 | 3.12E-01 | -5.585 |
| NOTCH1 | -0.143 | 7.973 | -1.355 | 1.77E-01 | 3.12E-01 | -5.585 |
| MUC20 | 0.26 | 7.563 | 1.352 | 1.78E-01 | 3.13E-01 | -5.587 |
| ANGPT2 | -0.32 | 8.32 | -1.353 | 1.78E-01 | 3.13E-01 | -5.588 |
| RHOV | 0.186 | 7.171 | 1.35 | 1.78E-01 | 3.14E-01 | -5.59 |
| PRKCSH | 0.153 | 9.508 | 1.35 | 1.78E-01 | 3.14E-01 | -5.59 |
| GFI1 | -0.176 | 5.976 | -1.351 | 1.78E-01 | 3.14E-01 | -5.59 |
| CHRM2 | 0.129 | 5.024 | 1.35 | 1.78E-01 | 3.14E-01 | -5.59 |
| MYH13 | 0.176 | 5.464 | 1.35 | 1.78E-01 | 3.14E-01 | -5.59 |
| HOOK2 | 0.12 | 8.787 | 1.349 | 1.78E-01 | 3.14E-01 | -5.591 |
| BCL9L | 0.141 | 7.646 | 1.348 | 1.79E-01 | 3.14E-01 | -5.592 |
| TNFSF18 | 0.149 | 4.819 | 1.348 | 1.79E-01 | 3.14E-01 | -5.592 |
| PSMB8 | -0.135 | 9.641 | -1.349 | 1.79E-01 | 3.14E-01 | -5.593 |
| HRH3 | 0.104 | 6.029 | 1.348 | 1.79E-01 | 3.14E-01 | -5.593 |
| NIPSNAP1 | 0.121 | 8.761 | 1.347 | 1.79E-01 | 3.15E-01 | -5.594 |
| BAIAP2L1 | 0.114 | 7.11 | 1.347 | 1.79E-01 | 3.15E-01 | -5.594 |
| CHST4 | 0.129 | 6.017 | 1.346 | 1.79E-01 | 3.15E-01 | -5.595 |
| ASB6 | 0.101 | 7.605 | 1.346 | 1.79E-01 | 3.15E-01 | -5.595 |
| CNTN4 | 0.137 | 5.364 | 1.346 | 1.80E-01 | 3.15E-01 | -5.596 |
| ARMC8 | -0.102 | 7.892 | -1.347 | 1.80E-01 | 3.15E-01 | -5.596 |
| MNT | 0.106 | 9.496 | 1.346 | 1.80E-01 | 3.15E-01 | -5.596 |
| SHCBP1 | -0.192 | 7.25 | -1.347 | 1.80E-01 | 3.15E-01 | -5.596 |
| ADAMTS15 | 0.144 | 5.463 | 1.346 | 1.80E-01 | 3.15E-01 | -5.596 |
| CEBPG | 0.141 | 9.484 | 1.345 | 1.80E-01 | 3.15E-01 | -5.597 |
| CPNE4 | -0.147 | 4.76 | -1.346 | 1.80E-01 | 3.15E-01 | -5.597 |
| GRASP | 0.234 | 8.285 | 1.345 | 1.80E-01 | 3.15E-01 | -5.597 |
| MMP24 | 0.181 | 5.858 | 1.344 | 1.80E-01 | 3.16E-01 | -5.598 |
| LGR5 | 0.167 | 5.831 | 1.344 | 1.80E-01 | 3.16E-01 | -5.598 |
| RPS19 | 0.091 | 13.977 | 1.344 | 1.80E-01 | 3.16E-01 | -5.598 |
| GNA15 | 0.146 | 7.168 | 1.344 | 1.80E-01 | 3.16E-01 | -5.599 |
| CFTR | -0.18 | 5.377 | -1.343 | 1.81E-01 | 3.16E-01 | -5.601 |
| TCF20 | -0.104 | 7.964 | -1.342 | 1.81E-01 | 3.17E-01 | -5.601 |
| GNMT | 0.171 | 5.484 | 1.341 | 1.81E-01 | 3.17E-01 | -5.602 |
| ISG20 | 0.211 | 10.192 | 1.34 | 1.81E-01 | 3.17E-01 | -5.602 |
| WDR1 | -0.117 | 10.573 | -1.341 | 1.81E-01 | 3.17E-01 | -5.603 |
| CTNNBIP1 | 0.139 | 8.954 | 1.339 | 1.82E-01 | 3.17E-01 | -5.604 |
| NCDN | 0.122 | 6.935 | 1.339 | 1.82E-01 | 3.17E-01 | -5.604 |
| HIC1 | 0.149 | 6.323 | 1.338 | 1.82E-01 | 3.18E-01 | -5.606 |
| C7orf13 | -0.157 | 6.289 | -1.339 | 1.82E-01 | 3.18E-01 | -5.606 |
| ENG | 0.211 | 9.979 | 1.337 | 1.83E-01 | 3.19E-01 | -5.607 |
| SLC5A8 | -0.193 | 5.066 | -1.337 | 1.83E-01 | 3.19E-01 | -5.608 |
| ANAPC10 | -0.118 | 8.543 | -1.337 | 1.83E-01 | 3.19E-01 | -5.608 |
| GPR88 | 0.143 | 5.107 | 1.336 | 1.83E-01 | 3.19E-01 | -5.609 |
| GCC1 | -0.096 | 9.094 | -1.337 | 1.83E-01 | 3.19E-01 | -5.609 |
| KBTBD2 | -0.122 | 9.557 | -1.336 | 1.83E-01 | 3.19E-01 | -5.61 |
| CROT | -0.217 | 7.466 | -1.335 | 1.83E-01 | 3.20E-01 | -5.611 |
| RPS24 | -0.157 | 12.284 | -1.335 | 1.83E-01 | 3.20E-01 | -5.611 |
| DACT1 | -0.167 | 7.361 | -1.334 | 1.84E-01 | 3.20E-01 | -5.612 |
| DNASE1 | 0.124 | 6.1 | 1.333 | 1.84E-01 | 3.20E-01 | -5.613 |
| PNLIPRP2 | 0.149 | 5.003 | 1.331 | 1.84E-01 | 3.21E-01 | -5.615 |
| HIPK3 | -0.172 | 6.98 | -1.332 | 1.84E-01 | 3.21E-01 | -5.615 |
| DRD2 | 0.131 | 5.934 | 1.331 | 1.84E-01 | 3.21E-01 | -5.615 |
| DST | 0.124 | 6.637 | 1.33 | 1.85E-01 | 3.21E-01 | -5.616 |
| POU4F1 | 0.17 | 4.912 | 1.33 | 1.85E-01 | 3.21E-01 | -5.616 |
| MARS2 | 0.138 | 5.818 | 1.33 | 1.85E-01 | 3.21E-01 | -5.616 |
| MYL1 | -0.122 | 4.748 | -1.329 | 1.85E-01 | 3.22E-01 | -5.618 |
| UBE2D4 | 0.105 | 8.409 | 1.328 | 1.85E-01 | 3.22E-01 | -5.618 |
| MRPL21 | 0.103 | 10.044 | 1.328 | 1.85E-01 | 3.22E-01 | -5.619 |
| ASXL2 | -0.11 | 9.022 | -1.329 | 1.85E-01 | 3.22E-01 | -5.619 |
| ZFP37 | -0.191 | 5.696 | -1.328 | 1.86E-01 | 3.22E-01 | -5.62 |
| MRE11A | -0.102 | 7.223 | -1.328 | 1.86E-01 | 3.22E-01 | -5.62 |
| PEPD | 0.125 | 10.238 | 1.327 | 1.86E-01 | 3.23E-01 | -5.621 |
| PKD2L2 | -0.131 | 4.941 | -1.327 | 1.86E-01 | 3.23E-01 | -5.621 |
| KCNK13 | 0.148 | 5.707 | 1.326 | 1.86E-01 | 3.23E-01 | -5.622 |
| POLE2 | -0.155 | 6.799 | -1.327 | 1.86E-01 | 3.23E-01 | -5.622 |
| CYP2E1 | 0.133 | 5.2 | 1.325 | 1.86E-01 | 3.23E-01 | -5.622 |
| C22orf15 | 0.172 | 6.536 | 1.324 | 1.87E-01 | 3.23E-01 | -5.623 |
| TBRG4 | 0.099 | 8.596 | 1.323 | 1.87E-01 | 3.24E-01 | -5.625 |
| STAR | -0.26 | 5.448 | -1.323 | 1.87E-01 | 3.24E-01 | -5.626 |
| ABAT | -0.132 | 7.08 | -1.323 | 1.87E-01 | 3.25E-01 | -5.627 |
| ARHGEF12 | 0.154 | 7.495 | 1.321 | 1.88E-01 | 3.25E-01 | -5.627 |
| RARRES2 | 0.231 | 8.599 | 1.32 | 1.88E-01 | 3.25E-01 | -5.629 |
| ZSWIM3 | -0.117 | 7.08 | -1.321 | 1.88E-01 | 3.26E-01 | -5.629 |
| HBQ1 | 0.221 | 8.616 | 1.32 | 1.88E-01 | 3.26E-01 | -5.63 |
| RGS20 | -0.208 | 6.35 | -1.32 | 1.88E-01 | 3.26E-01 | -5.63 |
| USP44 | -0.182 | 5.409 | -1.32 | 1.88E-01 | 3.26E-01 | -5.63 |
| LETMD1 | 0.093 | 8.946 | 1.319 | 1.88E-01 | 3.26E-01 | -5.63 |
| RALA | 0.146 | 9.785 | 1.319 | 1.88E-01 | 3.26E-01 | -5.63 |
| PCK1 | 0.169 | 5.006 | 1.319 | 1.88E-01 | 3.26E-01 | -5.631 |
| COPB2 | -0.09 | 11.462 | -1.319 | 1.89E-01 | 3.26E-01 | -5.631 |
| GLRA2 | 0.175 | 5.112 | 1.318 | 1.89E-01 | 3.26E-01 | -5.632 |
| SYT12 | 0.142 | 5.963 | 1.318 | 1.89E-01 | 3.26E-01 | -5.632 |
| COL3A1 | -0.197 | 10.381 | -1.319 | 1.89E-01 | 3.26E-01 | -5.632 |
| PRSS3 | 0.19 | 6.877 | 1.318 | 1.89E-01 | 3.26E-01 | -5.632 |
| ARL11 | -0.123 | 5.954 | -1.319 | 1.89E-01 | 3.26E-01 | -5.632 |
| AP2A1 | 0.185 | 8.055 | 1.317 | 1.89E-01 | 3.26E-01 | -5.632 |
| RP1L1 | 0.163 | 5.053 | 1.317 | 1.89E-01 | 3.26E-01 | -5.632 |
| TYROBP | 0.159 | 10.579 | 1.317 | 1.89E-01 | 3.26E-01 | -5.633 |
| MYOZ1 | 0.184 | 5.873 | 1.316 | 1.89E-01 | 3.26E-01 | -5.634 |
| RPL26L1 | 0.143 | 10.453 | 1.316 | 1.89E-01 | 3.26E-01 | -5.634 |
| GUCA1B | -0.133 | 5.776 | -1.316 | 1.90E-01 | 3.27E-01 | -5.636 |
| C1QTNF2 | 0.165 | 5.789 | 1.313 | 1.90E-01 | 3.28E-01 | -5.638 |
| PITPNB | -0.149 | 10.065 | -1.314 | 1.91E-01 | 3.28E-01 | -5.639 |
| INADL | -0.142 | 5.927 | -1.313 | 1.91E-01 | 3.28E-01 | -5.639 |
| SLC35C2 | 0.123 | 8.749 | 1.311 | 1.91E-01 | 3.29E-01 | -5.641 |
| SLC7A1 | -0.186 | 8.173 | -1.312 | 1.91E-01 | 3.29E-01 | -5.641 |
| SORCS2 | 0.171 | 5.552 | 1.31 | 1.91E-01 | 3.29E-01 | -5.641 |
| ZNF132 | 0.144 | 6.438 | 1.31 | 1.91E-01 | 3.29E-01 | -5.642 |
| GGA2 | 0.135 | 8.107 | 1.31 | 1.91E-01 | 3.29E-01 | -5.642 |
| CTGF | -0.264 | 10.227 | -1.31 | 1.92E-01 | 3.30E-01 | -5.643 |
| ROPN1L | 0.143 | 6.702 | 1.308 | 1.92E-01 | 3.30E-01 | -5.644 |
| FBXO9 | 0.102 | 9.312 | 1.304 | 1.93E-01 | 3.32E-01 | -5.649 |
| DSCR9 | -0.16 | 4.931 | -1.305 | 1.93E-01 | 3.32E-01 | -5.649 |
| FBXW7 | -0.097 | 8.338 | -1.305 | 1.93E-01 | 3.32E-01 | -5.65 |
| ZNF366 | -0.161 | 6.766 | -1.304 | 1.94E-01 | 3.33E-01 | -5.651 |
| PQLC2 | 0.111 | 7.871 | 1.302 | 1.94E-01 | 3.33E-01 | -5.652 |
| SERPINA4 | 0.164 | 4.772 | 1.301 | 1.95E-01 | 3.34E-01 | -5.654 |
| GAGE1 | 0.097 | 4.958 | 1.3 | 1.95E-01 | 3.34E-01 | -5.654 |
| SLC6A5 | 0.159 | 5.709 | 1.3 | 1.95E-01 | 3.34E-01 | -5.655 |
| NDN | 0.189 | 8.363 | 1.3 | 1.95E-01 | 3.34E-01 | -5.655 |
| PARP14 | -0.176 | 8.459 | -1.3 | 1.95E-01 | 3.34E-01 | -5.656 |
| AGTRAP | 0.144 | 8.261 | 1.299 | 1.95E-01 | 3.34E-01 | -5.656 |
| CDCA8 | 0.184 | 8.419 | 1.299 | 1.95E-01 | 3.34E-01 | -5.656 |
| NSD1 | -0.082 | 7.368 | -1.299 | 1.96E-01 | 3.35E-01 | -5.657 |
| FILIP1 | 0.171 | 6.247 | 1.298 | 1.96E-01 | 3.35E-01 | -5.657 |
| RAB11FIP3 | 0.122 | 8.449 | 1.297 | 1.96E-01 | 3.35E-01 | -5.658 |
| UNC93A | 0.138 | 5.151 | 1.297 | 1.96E-01 | 3.35E-01 | -5.658 |
| ZNF513 | 0.118 | 7.873 | 1.296 | 1.96E-01 | 3.35E-01 | -5.659 |
| PPP3CB | 0.117 | 9.358 | 1.296 | 1.96E-01 | 3.35E-01 | -5.659 |
| GSTA3 | 0.193 | 7.916 | 1.296 | 1.96E-01 | 3.35E-01 | -5.659 |
| RPL3L | 0.165 | 6.451 | 1.295 | 1.96E-01 | 3.36E-01 | -5.66 |
| PXK | -0.146 | 8.152 | -1.296 | 1.96E-01 | 3.36E-01 | -5.66 |
| EIF4B | 0.099 | 10.523 | 1.295 | 1.96E-01 | 3.36E-01 | -5.661 |
| DUSP16 | 0.135 | 7.688 | 1.295 | 1.96E-01 | 3.36E-01 | -5.661 |
| XYLT1 | 0.116 | 6.621 | 1.295 | 1.96E-01 | 3.36E-01 | -5.661 |
| S100A3 | 0.216 | 6.801 | 1.293 | 1.97E-01 | 3.37E-01 | -5.663 |
| DOK6 | 0.142 | 6.461 | 1.293 | 1.97E-01 | 3.37E-01 | -5.664 |
| TBX3 | -0.159 | 8.169 | -1.293 | 1.98E-01 | 3.37E-01 | -5.665 |
| C9 | 0.202 | 5.672 | 1.292 | 1.98E-01 | 3.37E-01 | -5.665 |
| SHPRH | 0.137 | 6.747 | 1.292 | 1.98E-01 | 3.37E-01 | -5.665 |
| APOD | 0.225 | 8.228 | 1.291 | 1.98E-01 | 3.37E-01 | -5.665 |
| GMIP | 0.161 | 7.881 | 1.291 | 1.98E-01 | 3.37E-01 | -5.666 |
| ATRX | -0.102 | 7.574 | -1.291 | 1.98E-01 | 3.38E-01 | -5.666 |
| ACLY | -0.113 | 10.311 | -1.291 | 1.98E-01 | 3.38E-01 | -5.667 |
| SPINT1 | 0.154 | 8.802 | 1.289 | 1.99E-01 | 3.38E-01 | -5.668 |
| GMPR2 | 0.098 | 9.3 | 1.289 | 1.99E-01 | 3.39E-01 | -5.669 |
| DPF2 | 0.119 | 10.46 | 1.288 | 1.99E-01 | 3.39E-01 | -5.669 |
| RB1 | -0.165 | 9.278 | -1.288 | 1.99E-01 | 3.39E-01 | -5.671 |
| IRX1 | 0.167 | 5.878 | 1.287 | 1.99E-01 | 3.39E-01 | -5.671 |
| BOC | 0.142 | 5.398 | 1.287 | 1.99E-01 | 3.39E-01 | -5.671 |
| DCD | -0.206 | 4.939 | -1.288 | 1.99E-01 | 3.39E-01 | -5.671 |
| SECTM1 | -0.134 | 7.805 | -1.287 | 2.00E-01 | 3.40E-01 | -5.672 |
| ASB2 | -0.263 | 7.344 | -1.287 | 2.00E-01 | 3.40E-01 | -5.672 |
| CDC26 | -0.09 | 10.003 | -1.286 | 2.00E-01 | 3.40E-01 | -5.673 |
| ROM1 | 0.22 | 6.88 | 1.285 | 2.00E-01 | 3.40E-01 | -5.674 |
| VNN1 | -0.217 | 6.926 | -1.286 | 2.00E-01 | 3.40E-01 | -5.674 |
| C11orf16 | -0.138 | 5.167 | -1.285 | 2.00E-01 | 3.41E-01 | -5.675 |
| RIN1 | 0.124 | 8.114 | 1.283 | 2.01E-01 | 3.41E-01 | -5.676 |
| PRDM11 | 0.162 | 6.177 | 1.283 | 2.01E-01 | 3.41E-01 | -5.676 |
| SIM1 | 0.146 | 5.397 | 1.282 | 2.01E-01 | 3.41E-01 | -5.677 |
| PTGER1 | 0.153 | 5.312 | 1.281 | 2.01E-01 | 3.42E-01 | -5.678 |
| HYAL1 | 0.174 | 5.9 | 1.281 | 2.01E-01 | 3.42E-01 | -5.679 |
| SEZ6L | 0.147 | 5.067 | 1.279 | 2.02E-01 | 3.43E-01 | -5.681 |
| PVRL3 | -0.196 | 9.759 | -1.28 | 2.02E-01 | 3.43E-01 | -5.681 |
| GUCA2B | 0.167 | 5.168 | 1.278 | 2.02E-01 | 3.43E-01 | -5.682 |
| FRMD3 | -0.199 | 6.616 | -1.279 | 2.02E-01 | 3.43E-01 | -5.682 |
| MPZL1 | 0.114 | 8.983 | 1.278 | 2.03E-01 | 3.43E-01 | -5.683 |
| YY1AP1 | 0.108 | 9.921 | 1.275 | 2.04E-01 | 3.45E-01 | -5.686 |
| N4BP2 | 0.141 | 8.135 | 1.275 | 2.04E-01 | 3.45E-01 | -5.686 |
| TPM4 | -0.114 | 10.656 | -1.276 | 2.04E-01 | 3.45E-01 | -5.686 |
| TAF1C | -0.113 | 8.67 | -1.275 | 2.04E-01 | 3.45E-01 | -5.687 |
| LYNX1 | 0.149 | 7.169 | 1.271 | 2.05E-01 | 3.47E-01 | -5.69 |
| ABCB1 | -0.227 | 7.631 | -1.272 | 2.05E-01 | 3.47E-01 | -5.691 |
| RNASET2 | 0.148 | 10.111 | 1.271 | 2.05E-01 | 3.47E-01 | -5.691 |
| TMEM18 | 0.097 | 8.175 | 1.269 | 2.05E-01 | 3.48E-01 | -5.693 |
| TYRP1 | 0.124 | 4.68 | 1.269 | 2.05E-01 | 3.48E-01 | -5.693 |
| DKK4 | -0.111 | 4.616 | -1.27 | 2.06E-01 | 3.48E-01 | -5.694 |
| SST | -0.2 | 5.023 | -1.269 | 2.06E-01 | 3.48E-01 | -5.694 |
| COL21A1 | -0.148 | 7.095 | -1.269 | 2.06E-01 | 3.48E-01 | -5.694 |
| SMAD6 | 0.183 | 8.29 | 1.268 | 2.06E-01 | 3.48E-01 | -5.695 |
| DLX4 | 0.148 | 7.51 | 1.268 | 2.06E-01 | 3.48E-01 | -5.695 |
| DHX29 | -0.116 | 9.281 | -1.267 | 2.07E-01 | 3.49E-01 | -5.697 |
| NFKBIB | 0.103 | 7.853 | 1.266 | 2.07E-01 | 3.49E-01 | -5.697 |
| LRG1 | -0.169 | 7.417 | -1.266 | 2.07E-01 | 3.50E-01 | -5.698 |
| C9orf156 | -0.098 | 8.189 | -1.265 | 2.08E-01 | 3.50E-01 | -5.7 |
| PPP2R5B | 0.122 | 8.006 | 1.263 | 2.08E-01 | 3.50E-01 | -5.7 |
| POLR1C | 0.095 | 8.811 | 1.263 | 2.08E-01 | 3.50E-01 | -5.7 |
| SPATA5L1 | -0.096 | 7.946 | -1.264 | 2.08E-01 | 3.50E-01 | -5.7 |
| GALNT9 | 0.141 | 5.465 | 1.262 | 2.08E-01 | 3.51E-01 | -5.701 |
| PTPRE | 0.113 | 7.676 | 1.262 | 2.08E-01 | 3.51E-01 | -5.702 |
| AKR7A2 | 0.086 | 10.583 | 1.262 | 2.08E-01 | 3.51E-01 | -5.702 |
| XYLB | -0.135 | 5.169 | -1.262 | 2.08E-01 | 3.51E-01 | -5.703 |
| PNPLA4 | -0.123 | 6.724 | -1.262 | 2.09E-01 | 3.51E-01 | -5.703 |
| STK39 | 0.147 | 7.662 | 1.26 | 2.09E-01 | 3.52E-01 | -5.704 |
| CHST7 | 0.186 | 8.457 | 1.26 | 2.09E-01 | 3.52E-01 | -5.705 |
| ELAC2 | 0.104 | 9.048 | 1.26 | 2.09E-01 | 3.52E-01 | -5.705 |
| ARVCF | -0.144 | 6.29 | -1.26 | 2.09E-01 | 3.52E-01 | -5.705 |
| MID2 | 0.104 | 6.796 | 1.258 | 2.09E-01 | 3.52E-01 | -5.707 |
| TP53INP2 | 0.151 | 7.234 | 1.257 | 2.10E-01 | 3.53E-01 | -5.708 |
| ITPR2 | -0.117 | 7.632 | -1.258 | 2.10E-01 | 3.53E-01 | -5.708 |
| UNC119 | 0.116 | 8.096 | 1.257 | 2.10E-01 | 3.53E-01 | -5.708 |
| SUSD1 | 0.111 | 9.27 | 1.257 | 2.10E-01 | 3.53E-01 | -5.708 |
| CECR1 | 0.148 | 7.619 | 1.256 | 2.10E-01 | 3.53E-01 | -5.71 |
| CRYAA | -0.144 | 5.37 | -1.256 | 2.11E-01 | 3.54E-01 | -5.71 |
| ARSE | 0.188 | 5.051 | 1.255 | 2.11E-01 | 3.54E-01 | -5.711 |
| HOXA13 | 0.185 | 6.939 | 1.254 | 2.11E-01 | 3.54E-01 | -5.712 |
| ARG2 | 0.197 | 7.309 | 1.253 | 2.11E-01 | 3.55E-01 | -5.712 |
| NNT | -0.116 | 6.71 | -1.253 | 2.12E-01 | 3.55E-01 | -5.714 |
| LAIR1 | -0.132 | 8.081 | -1.252 | 2.12E-01 | 3.56E-01 | -5.716 |
| PSEN2 | 0.106 | 8.537 | 1.251 | 2.12E-01 | 3.56E-01 | -5.716 |
| PI3 | 0.204 | 6.699 | 1.25 | 2.12E-01 | 3.56E-01 | -5.716 |
| SLC22A8 | 0.17 | 5.56 | 1.248 | 2.13E-01 | 3.57E-01 | -5.718 |
| MYT1 | 0.124 | 4.921 | 1.248 | 2.13E-01 | 3.57E-01 | -5.719 |
| PRAP1 | 0.152 | 6.366 | 1.247 | 2.14E-01 | 3.58E-01 | -5.72 |
| RPS11 | 0.085 | 14.372 | 1.247 | 2.14E-01 | 3.58E-01 | -5.72 |
| MYD88 | 0.18 | 8.752 | 1.247 | 2.14E-01 | 3.58E-01 | -5.721 |
| CDH12 | 0.173 | 5.156 | 1.246 | 2.14E-01 | 3.58E-01 | -5.721 |
| HAS1 | 0.153 | 5.243 | 1.246 | 2.14E-01 | 3.58E-01 | -5.721 |
| ZDHHC8 | -0.163 | 9.58 | -1.247 | 2.14E-01 | 3.58E-01 | -5.722 |
| ARRDC2 | 0.132 | 7.974 | 1.245 | 2.14E-01 | 3.59E-01 | -5.723 |
| NXPH1 | -0.159 | 4.699 | -1.245 | 2.15E-01 | 3.59E-01 | -5.724 |
| CNOT4 | 0.075 | 7.227 | 1.244 | 2.15E-01 | 3.59E-01 | -5.724 |
| PABPC5 | 0.15 | 5.257 | 1.243 | 2.15E-01 | 3.59E-01 | -5.725 |
| SYK | 0.149 | 7.832 | 1.243 | 2.15E-01 | 3.59E-01 | -5.725 |
| HMP19 | 0.164 | 5.648 | 1.242 | 2.15E-01 | 3.60E-01 | -5.726 |
| C21orf33 | 0.094 | 10.101 | 1.242 | 2.15E-01 | 3.60E-01 | -5.726 |
| PRF1 | 0.16 | 6.773 | 1.241 | 2.16E-01 | 3.60E-01 | -5.727 |
| PACS1 | -0.163 | 7.991 | -1.241 | 2.16E-01 | 3.61E-01 | -5.729 |
| THPO | 0.13 | 5.197 | 1.24 | 2.16E-01 | 3.61E-01 | -5.729 |
| EYA2 | 0.127 | 6.246 | 1.24 | 2.16E-01 | 3.61E-01 | -5.729 |
| IFNGR1 | -0.182 | 11.368 | -1.241 | 2.16E-01 | 3.61E-01 | -5.729 |
| CD200R1 | -0.16 | 5.836 | -1.24 | 2.16E-01 | 3.61E-01 | -5.73 |
| OR3A2 | 0.162 | 5.043 | 1.239 | 2.16E-01 | 3.61E-01 | -5.73 |
| TRAM1L1 | -0.15 | 5.225 | -1.24 | 2.17E-01 | 3.61E-01 | -5.73 |
| SLC41A2 | -0.164 | 7.311 | -1.239 | 2.17E-01 | 3.61E-01 | -5.731 |
| THADA | 0.079 | 7.28 | 1.238 | 2.17E-01 | 3.61E-01 | -5.731 |
| AACS | 0.136 | 8.422 | 1.238 | 2.17E-01 | 3.61E-01 | -5.731 |
| E2F2 | 0.167 | 8.392 | 1.238 | 2.17E-01 | 3.61E-01 | -5.731 |
| RFC1 | -0.114 | 7.965 | -1.238 | 2.17E-01 | 3.62E-01 | -5.732 |
| INHBA | -0.256 | 9.175 | -1.238 | 2.17E-01 | 3.62E-01 | -5.732 |
| MTNR1B | 0.138 | 4.782 | 1.237 | 2.17E-01 | 3.62E-01 | -5.732 |
| GNPDA2 | -0.161 | 7.007 | -1.238 | 2.17E-01 | 3.62E-01 | -5.733 |
| SAMD11 | 0.141 | 6.432 | 1.237 | 2.17E-01 | 3.62E-01 | -5.733 |
| ARSD | 0.125 | 7.472 | 1.237 | 2.17E-01 | 3.62E-01 | -5.733 |
| CA8 | -0.144 | 6.653 | -1.237 | 2.18E-01 | 3.62E-01 | -5.734 |
| BAIAP2 | 0.101 | 7.304 | 1.235 | 2.18E-01 | 3.62E-01 | -5.734 |
| RCE1 | 0.112 | 7.784 | 1.235 | 2.18E-01 | 3.62E-01 | -5.735 |
| POU4F2 | 0.19 | 5.753 | 1.235 | 2.18E-01 | 3.63E-01 | -5.735 |
| TLR8 | -0.131 | 6.931 | -1.235 | 2.18E-01 | 3.63E-01 | -5.735 |
| TMEM45A | 0.244 | 8.624 | 1.234 | 2.18E-01 | 3.63E-01 | -5.736 |
| USP54 | 0.109 | 6.653 | 1.233 | 2.19E-01 | 3.63E-01 | -5.737 |
| RASSF1 | 0.089 | 8.627 | 1.233 | 2.19E-01 | 3.63E-01 | -5.737 |
| CYP2J2 | -0.168 | 8.545 | -1.233 | 2.19E-01 | 3.63E-01 | -5.738 |
| TRIM22 | -0.205 | 8.652 | -1.233 | 2.19E-01 | 3.63E-01 | -5.738 |
| FCHSD1 | 0.14 | 6.839 | 1.232 | 2.19E-01 | 3.63E-01 | -5.738 |
| UPP1 | -0.138 | 8.55 | -1.233 | 2.19E-01 | 3.64E-01 | -5.738 |
| PTPRS | 0.111 | 5.987 | 1.231 | 2.19E-01 | 3.64E-01 | -5.739 |
| CASP8AP2 | -0.126 | 7.674 | -1.232 | 2.19E-01 | 3.64E-01 | -5.739 |
| C15orf26 | 0.167 | 5.526 | 1.231 | 2.19E-01 | 3.64E-01 | -5.74 |
| CNTNAP4 | -0.097 | 5.108 | -1.232 | 2.20E-01 | 3.64E-01 | -5.74 |
| GPR146 | 0.173 | 7.195 | 1.23 | 2.20E-01 | 3.64E-01 | -5.741 |
| ZNF256 | -0.103 | 8.003 | -1.231 | 2.20E-01 | 3.64E-01 | -5.741 |
| SLCO1A2 | 0.119 | 4.946 | 1.229 | 2.20E-01 | 3.65E-01 | -5.742 |
| GPT | 0.16 | 6.357 | 1.229 | 2.20E-01 | 3.65E-01 | -5.742 |
| GFRA4 | 0.151 | 7.089 | 1.228 | 2.20E-01 | 3.65E-01 | -5.743 |
| HRH1 | 0.124 | 6.555 | 1.227 | 2.21E-01 | 3.65E-01 | -5.744 |
| GABRD | 0.186 | 5.136 | 1.227 | 2.21E-01 | 3.66E-01 | -5.745 |
| NEUROD1 | -0.117 | 4.719 | -1.227 | 2.21E-01 | 3.66E-01 | -5.746 |
| FLNA | -0.15 | 8.779 | -1.226 | 2.22E-01 | 3.67E-01 | -5.747 |
| FGD3 | -0.102 | 7.793 | -1.226 | 2.22E-01 | 3.67E-01 | -5.747 |
| DPCR1 | -0.135 | 5.084 | -1.226 | 2.22E-01 | 3.67E-01 | -5.747 |
| TAF2 | -0.15 | 8.455 | -1.225 | 2.22E-01 | 3.67E-01 | -5.747 |
| ZNF175 | -0.145 | 7.111 | -1.225 | 2.22E-01 | 3.67E-01 | -5.748 |
| PIR | -0.191 | 8.749 | -1.224 | 2.22E-01 | 3.67E-01 | -5.749 |
| CDK5RAP1 | 0.079 | 8.428 | 1.223 | 2.22E-01 | 3.67E-01 | -5.749 |
| C5orf15 | -0.121 | 10.658 | -1.224 | 2.23E-01 | 3.68E-01 | -5.75 |
| NRAS | -0.107 | 8.365 | -1.223 | 2.23E-01 | 3.68E-01 | -5.751 |
| HDAC5 | 0.103 | 8.943 | 1.221 | 2.23E-01 | 3.68E-01 | -5.751 |
| C14orf1 | 0.122 | 6.933 | 1.221 | 2.23E-01 | 3.68E-01 | -5.752 |
| WFDC6 | 0.108 | 4.872 | 1.221 | 2.23E-01 | 3.68E-01 | -5.752 |
| ABO | -0.122 | 5.724 | -1.221 | 2.24E-01 | 3.69E-01 | -5.753 |
| PRKAG3 | 0.172 | 5.74 | 1.22 | 2.24E-01 | 3.69E-01 | -5.753 |
| TBX19 | -0.129 | 6.353 | -1.219 | 2.24E-01 | 3.69E-01 | -5.754 |
| REPS1 | 0.081 | 9.079 | 1.218 | 2.24E-01 | 3.70E-01 | -5.755 |
| GULP1 | -0.169 | 8.745 | -1.219 | 2.24E-01 | 3.70E-01 | -5.755 |
| AGTR2 | -0.13 | 4.742 | -1.218 | 2.25E-01 | 3.70E-01 | -5.756 |
| TMEM14A | 0.125 | 8.685 | 1.216 | 2.25E-01 | 3.71E-01 | -5.757 |
| WDR31 | 0.134 | 5.081 | 1.216 | 2.25E-01 | 3.71E-01 | -5.758 |
| CLSPN | 0.16 | 5.664 | 1.216 | 2.25E-01 | 3.71E-01 | -5.758 |
| CNNM4 | 0.139 | 8.187 | 1.216 | 2.25E-01 | 3.71E-01 | -5.758 |
| ANTXR1 | -0.168 | 7.847 | -1.216 | 2.25E-01 | 3.71E-01 | -5.758 |
| CTNNB1 | -0.085 | 9.603 | -1.216 | 2.26E-01 | 3.71E-01 | -5.759 |
| KCNB1 | 0.151 | 5.176 | 1.214 | 2.26E-01 | 3.71E-01 | -5.76 |
| EZH1 | -0.133 | 7.616 | -1.215 | 2.26E-01 | 3.72E-01 | -5.76 |
| JARID2 | -0.109 | 8.935 | -1.214 | 2.26E-01 | 3.72E-01 | -5.76 |
| MAP2K6 | -0.105 | 6.196 | -1.214 | 2.26E-01 | 3.72E-01 | -5.761 |
| VKORC1 | 0.104 | 9.583 | 1.212 | 2.27E-01 | 3.72E-01 | -5.762 |
| MBD3 | 0.133 | 9.215 | 1.21 | 2.27E-01 | 3.73E-01 | -5.764 |
| CLTCL1 | -0.131 | 6.501 | -1.211 | 2.27E-01 | 3.73E-01 | -5.765 |
| GLI3 | 0.117 | 8.608 | 1.209 | 2.28E-01 | 3.74E-01 | -5.765 |
| PRSS33 | 0.118 | 6.152 | 1.209 | 2.28E-01 | 3.74E-01 | -5.765 |
| SOSTDC1 | 0.175 | 4.962 | 1.209 | 2.28E-01 | 3.74E-01 | -5.766 |
| LIX1L | 0.126 | 7.83 | 1.208 | 2.28E-01 | 3.74E-01 | -5.766 |
| CD1A | -0.119 | 5.274 | -1.209 | 2.28E-01 | 3.74E-01 | -5.766 |
| SCNN1A | 0.206 | 5.794 | 1.208 | 2.28E-01 | 3.74E-01 | -5.766 |
| KLF2 | -0.144 | 11.306 | -1.209 | 2.28E-01 | 3.74E-01 | -5.767 |
| SYNPR | -0.221 | 5.363 | -1.209 | 2.28E-01 | 3.74E-01 | -5.767 |
| ADAM28 | -0.12 | 6.089 | -1.209 | 2.28E-01 | 3.74E-01 | -5.767 |
| DAP | -0.115 | 10.454 | -1.209 | 2.28E-01 | 3.74E-01 | -5.767 |
| IMPG1 | -0.128 | 4.638 | -1.208 | 2.29E-01 | 3.74E-01 | -5.768 |
| PHLDA1 | -0.14 | 7.317 | -1.207 | 2.29E-01 | 3.75E-01 | -5.769 |
| TAF11 | -0.111 | 7.957 | -1.207 | 2.29E-01 | 3.75E-01 | -5.769 |
| MYO9A | -0.14 | 9.369 | -1.207 | 2.29E-01 | 3.75E-01 | -5.77 |
| ATP1A2 | 0.145 | 5.829 | 1.205 | 2.29E-01 | 3.75E-01 | -5.77 |
| CALN1 | 0.095 | 5.619 | 1.205 | 2.29E-01 | 3.75E-01 | -5.771 |
| LACE1 | 0.121 | 6.486 | 1.202 | 2.30E-01 | 3.77E-01 | -5.774 |
| B3GAT1 | 0.176 | 5.949 | 1.201 | 2.31E-01 | 3.77E-01 | -5.775 |
| APOL2 | -0.117 | 9.301 | -1.202 | 2.31E-01 | 3.77E-01 | -5.775 |
| CNDP1 | 0.128 | 4.952 | 1.2 | 2.31E-01 | 3.78E-01 | -5.776 |
| ZNF512 | -0.111 | 7.916 | -1.201 | 2.31E-01 | 3.78E-01 | -5.777 |
| ELAVL4 | 0.131 | 5.042 | 1.2 | 2.31E-01 | 3.78E-01 | -5.777 |
| TTR | 0.263 | 5.643 | 1.199 | 2.31E-01 | 3.78E-01 | -5.777 |
| ZNF32 | 0.11 | 7.57 | 1.199 | 2.31E-01 | 3.78E-01 | -5.777 |
| SF3A3 | 0.099 | 9.971 | 1.198 | 2.32E-01 | 3.79E-01 | -5.779 |
| YARS | -0.103 | 9.352 | -1.198 | 2.33E-01 | 3.80E-01 | -5.78 |
| CLCN4 | 0.137 | 5.741 | 1.196 | 2.33E-01 | 3.80E-01 | -5.78 |
| CCNT1 | 0.117 | 7.533 | 1.196 | 2.33E-01 | 3.80E-01 | -5.781 |
| APOL3 | 0.122 | 7.853 | 1.196 | 2.33E-01 | 3.80E-01 | -5.781 |
| PRKD1 | -0.131 | 6.838 | -1.196 | 2.33E-01 | 3.80E-01 | -5.782 |
| GLRB | 0.131 | 5.71 | 1.194 | 2.33E-01 | 3.81E-01 | -5.783 |
| RARA | 0.111 | 8.501 | 1.194 | 2.34E-01 | 3.81E-01 | -5.784 |
| CACNB1 | 0.107 | 5.695 | 1.193 | 2.34E-01 | 3.81E-01 | -5.784 |
| GDF15 | -0.182 | 12.239 | -1.194 | 2.34E-01 | 3.81E-01 | -5.784 |
| CHRNB2 | 0.129 | 5.597 | 1.193 | 2.34E-01 | 3.81E-01 | -5.784 |
| GLDC | -0.124 | 8.913 | -1.193 | 2.34E-01 | 3.81E-01 | -5.785 |
| GABRA5 | 0.111 | 4.703 | 1.192 | 2.34E-01 | 3.81E-01 | -5.786 |
| SCN11A | -0.182 | 5.222 | -1.193 | 2.34E-01 | 3.81E-01 | -5.786 |
| CXCL9 | -0.307 | 6.12 | -1.192 | 2.35E-01 | 3.82E-01 | -5.787 |
| KIF20A | 0.2 | 7.717 | 1.191 | 2.35E-01 | 3.82E-01 | -5.787 |
| NTRK1 | 0.157 | 5.292 | 1.191 | 2.35E-01 | 3.82E-01 | -5.787 |
| ZNF135 | -0.102 | 6.791 | -1.192 | 2.35E-01 | 3.82E-01 | -5.787 |
| PTCD2 | -0.108 | 6.333 | -1.192 | 2.35E-01 | 3.82E-01 | -5.787 |
| C10orf32 | -0.116 | 9.342 | -1.191 | 2.35E-01 | 3.82E-01 | -5.787 |
| CYYR1 | -0.164 | 7.401 | -1.191 | 2.35E-01 | 3.82E-01 | -5.788 |
| ZNF625 | -0.122 | 6.53 | -1.191 | 2.35E-01 | 3.82E-01 | -5.788 |
| DRG1 | 0.107 | 10.357 | 1.19 | 2.35E-01 | 3.82E-01 | -5.788 |
| ZGPAT | 0.111 | 8.806 | 1.19 | 2.35E-01 | 3.82E-01 | -5.788 |
| SYT13 | 0.142 | 5.22 | 1.19 | 2.35E-01 | 3.82E-01 | -5.788 |
| HBS1L | -0.139 | 6.141 | -1.187 | 2.37E-01 | 3.84E-01 | -5.792 |
| KCTD5 | -0.097 | 8.736 | -1.186 | 2.37E-01 | 3.85E-01 | -5.794 |
| TEKT1 | 0.138 | 4.875 | 1.185 | 2.37E-01 | 3.85E-01 | -5.794 |
| DUSP3 | -0.112 | 9.904 | -1.184 | 2.38E-01 | 3.86E-01 | -5.796 |
| SLC39A12 | 0.158 | 5.221 | 1.181 | 2.38E-01 | 3.87E-01 | -5.798 |
| SPARC | 0.132 | 12.889 | 1.181 | 2.39E-01 | 3.87E-01 | -5.798 |
| COL8A2 | -0.153 | 7.388 | -1.182 | 2.39E-01 | 3.87E-01 | -5.798 |
| UPK1A | 0.13 | 5.187 | 1.179 | 2.40E-01 | 3.88E-01 | -5.801 |
| FOXL2 | 0.173 | 6.379 | 1.178 | 2.40E-01 | 3.88E-01 | -5.802 |
| NUP37 | 0.093 | 9.453 | 1.177 | 2.40E-01 | 3.89E-01 | -5.803 |
| CSTB | 0.118 | 10.966 | 1.176 | 2.40E-01 | 3.89E-01 | -5.804 |
| ZDHHC7 | -0.084 | 10.453 | -1.177 | 2.41E-01 | 3.90E-01 | -5.804 |
| AURKB | -0.152 | 7.627 | -1.176 | 2.41E-01 | 3.90E-01 | -5.805 |
| OSBP2 | 0.164 | 6.965 | 1.174 | 2.41E-01 | 3.90E-01 | -5.806 |
| TEAD3 | -0.173 | 9.118 | -1.175 | 2.42E-01 | 3.91E-01 | -5.807 |
| CXorf38 | -0.115 | 7.777 | -1.174 | 2.42E-01 | 3.91E-01 | -5.807 |
| LRP10 | 0.12 | 10.435 | 1.173 | 2.42E-01 | 3.91E-01 | -5.807 |
| DNAJB8 | -0.141 | 5.189 | -1.174 | 2.42E-01 | 3.91E-01 | -5.808 |
| NEXN | -0.168 | 7.915 | -1.174 | 2.42E-01 | 3.91E-01 | -5.808 |
| DDR2 | 0.136 | 6.689 | 1.173 | 2.42E-01 | 3.91E-01 | -5.808 |
| STS | -0.153 | 8.233 | -1.173 | 2.42E-01 | 3.91E-01 | -5.808 |
| FBXO18 | 0.085 | 8.562 | 1.172 | 2.42E-01 | 3.91E-01 | -5.809 |
| CLDN11 | 0.131 | 8.007 | 1.17 | 2.43E-01 | 3.92E-01 | -5.81 |
| PPIC | -0.112 | 9.323 | -1.171 | 2.43E-01 | 3.92E-01 | -5.811 |
| CDH4 | -0.143 | 5.043 | -1.171 | 2.43E-01 | 3.93E-01 | -5.811 |
| TMSB4Y | -0.172 | 5.263 | -1.17 | 2.43E-01 | 3.93E-01 | -5.812 |
| TAP1 | 0.185 | 10.914 | 1.167 | 2.44E-01 | 3.94E-01 | -5.815 |
| SCN2B | -0.143 | 4.98 | -1.167 | 2.45E-01 | 3.94E-01 | -5.815 |
| WDR6 | 0.087 | 10.347 | 1.166 | 2.45E-01 | 3.94E-01 | -5.815 |
| SERPINA6 | 0.174 | 6.225 | 1.166 | 2.45E-01 | 3.94E-01 | -5.815 |
| ASIP | 0.121 | 6.211 | 1.166 | 2.45E-01 | 3.94E-01 | -5.816 |
| OCA2 | 0.122 | 5.917 | 1.164 | 2.45E-01 | 3.95E-01 | -5.818 |
| VAMP2 | 0.134 | 8.229 | 1.164 | 2.45E-01 | 3.95E-01 | -5.818 |
| GLRA1 | -0.127 | 4.803 | -1.165 | 2.46E-01 | 3.95E-01 | -5.818 |
| CDH1 | -0.129 | 8.456 | -1.164 | 2.46E-01 | 3.96E-01 | -5.819 |
| HMGCS1 | -0.146 | 8.146 | -1.164 | 2.46E-01 | 3.96E-01 | -5.819 |
| SCGB3A2 | 0.148 | 6.566 | 1.163 | 2.46E-01 | 3.96E-01 | -5.819 |
| ADHFE1 | -0.159 | 8.946 | -1.164 | 2.46E-01 | 3.96E-01 | -5.819 |
| CHPT1 | 0.146 | 9.267 | 1.162 | 2.46E-01 | 3.96E-01 | -5.82 |
| LITAF | -0.131 | 11.14 | -1.163 | 2.46E-01 | 3.96E-01 | -5.82 |
| ZFX | -0.128 | 7.007 | -1.162 | 2.47E-01 | 3.97E-01 | -5.822 |
| KRTAP13-1 | 0.155 | 5.141 | 1.16 | 2.47E-01 | 3.97E-01 | -5.822 |
| NSUN6 | -0.142 | 6.824 | -1.161 | 2.47E-01 | 3.97E-01 | -5.822 |
| BANK1 | -0.142 | 6.168 | -1.16 | 2.47E-01 | 3.98E-01 | -5.823 |
| C9orf66 | -0.125 | 6.366 | -1.16 | 2.48E-01 | 3.98E-01 | -5.824 |
| FERD3L | -0.139 | 5.148 | -1.16 | 2.48E-01 | 3.98E-01 | -5.824 |
| LAMA1 | 0.118 | 5.671 | 1.158 | 2.48E-01 | 3.98E-01 | -5.825 |
| FKBP2 | 0.099 | 11.138 | 1.158 | 2.48E-01 | 3.98E-01 | -5.825 |
| DCX | 0.161 | 5.851 | 1.157 | 2.48E-01 | 3.99E-01 | -5.826 |
| KLHL11 | -0.13 | 6.271 | -1.157 | 2.49E-01 | 3.99E-01 | -5.827 |
| NPM2 | 0.15 | 5.903 | 1.155 | 2.49E-01 | 3.99E-01 | -5.828 |
| DNM2 | 0.087 | 9.218 | 1.155 | 2.49E-01 | 4.00E-01 | -5.828 |
| PALMD | 0.144 | 7.226 | 1.154 | 2.49E-01 | 4.00E-01 | -5.829 |
| KLRB1 | 0.186 | 6.814 | 1.154 | 2.49E-01 | 4.00E-01 | -5.829 |
| PSMD3 | 0.097 | 9.822 | 1.153 | 2.50E-01 | 4.00E-01 | -5.83 |
| ZNF239 | -0.147 | 6.716 | -1.154 | 2.50E-01 | 4.01E-01 | -5.831 |
| ANGPTL4 | -0.215 | 9.12 | -1.152 | 2.51E-01 | 4.01E-01 | -5.832 |
| PGLYRP1 | -0.167 | 6.593 | -1.152 | 2.51E-01 | 4.01E-01 | -5.832 |
| MRPL16 | 0.082 | 10.149 | 1.15 | 2.51E-01 | 4.02E-01 | -5.833 |
| TSGA10IP | 0.159 | 5.484 | 1.15 | 2.51E-01 | 4.02E-01 | -5.834 |
| MGAT5 | -0.123 | 6.421 | -1.15 | 2.51E-01 | 4.02E-01 | -5.834 |
| NUP62 | -0.083 | 10.583 | -1.149 | 2.52E-01 | 4.03E-01 | -5.836 |
| CHD5 | 0.119 | 4.916 | 1.146 | 2.53E-01 | 4.04E-01 | -5.838 |
| CLCA1 | 0.13 | 4.831 | 1.145 | 2.53E-01 | 4.05E-01 | -5.839 |
| SGPP2 | -0.114 | 4.939 | -1.146 | 2.53E-01 | 4.05E-01 | -5.839 |
| PHKB | -0.087 | 9.392 | -1.146 | 2.53E-01 | 4.05E-01 | -5.839 |
| TGIF2 | 0.141 | 6.866 | 1.145 | 2.53E-01 | 4.05E-01 | -5.839 |
| IL10 | 0.128 | 6.017 | 1.144 | 2.54E-01 | 4.05E-01 | -5.84 |
| APC2 | 0.146 | 5.971 | 1.143 | 2.54E-01 | 4.06E-01 | -5.841 |
| P2RY8 | 0.148 | 6.456 | 1.143 | 2.54E-01 | 4.06E-01 | -5.842 |
| ANAPC4 | -0.116 | 9.752 | -1.142 | 2.55E-01 | 4.07E-01 | -5.843 |
| RASSF6 | -0.12 | 6.22 | -1.141 | 2.55E-01 | 4.07E-01 | -5.844 |
| WNT7A | 0.189 | 6.952 | 1.14 | 2.55E-01 | 4.07E-01 | -5.845 |
| PSMF1 | 0.091 | 9.247 | 1.14 | 2.55E-01 | 4.07E-01 | -5.845 |
| NUDC | 0.117 | 10.354 | 1.14 | 2.55E-01 | 4.07E-01 | -5.845 |
| CDX4 | 0.124 | 4.931 | 1.14 | 2.55E-01 | 4.07E-01 | -5.845 |
| PCDHB2 | 0.165 | 5.565 | 1.139 | 2.55E-01 | 4.07E-01 | -5.845 |
| PYGM | 0.147 | 6.65 | 1.139 | 2.56E-01 | 4.07E-01 | -5.845 |
| DNTT | 0.124 | 5.055 | 1.139 | 2.56E-01 | 4.07E-01 | -5.846 |
| DOCK2 | -0.129 | 7.766 | -1.14 | 2.56E-01 | 4.07E-01 | -5.846 |
| C11orf1 | -0.103 | 5.957 | -1.14 | 2.56E-01 | 4.08E-01 | -5.846 |
| NANOG | -0.146 | 5.584 | -1.139 | 2.56E-01 | 4.08E-01 | -5.847 |
| RHAG | 0.193 | 5.536 | 1.138 | 2.56E-01 | 4.08E-01 | -5.847 |
| LHCGR | -0.109 | 4.691 | -1.139 | 2.56E-01 | 4.08E-01 | -5.847 |
| PIK3R5 | -0.123 | 6.37 | -1.138 | 2.57E-01 | 4.08E-01 | -5.848 |
| UBE2L3 | 0.07 | 9.565 | 1.136 | 2.57E-01 | 4.09E-01 | -5.849 |
| UBE2G2 | 0.09 | 8.537 | 1.135 | 2.57E-01 | 4.09E-01 | -5.85 |
| NRGN | 0.147 | 8.337 | 1.134 | 2.58E-01 | 4.10E-01 | -5.852 |
| ACTL6A | -0.074 | 10.112 | -1.134 | 2.58E-01 | 4.10E-01 | -5.852 |
| BBS2 | -0.096 | 8.987 | -1.134 | 2.58E-01 | 4.10E-01 | -5.853 |
| RBL1 | -0.097 | 6.026 | -1.134 | 2.58E-01 | 4.10E-01 | -5.853 |
| ABTB1 | -0.123 | 8.541 | -1.133 | 2.58E-01 | 4.11E-01 | -5.853 |
| SLC30A1 | -0.133 | 8.322 | -1.132 | 2.59E-01 | 4.11E-01 | -5.854 |
| EPB41 | 0.125 | 6.389 | 1.13 | 2.59E-01 | 4.12E-01 | -5.855 |
| CBX1 | -0.099 | 9.624 | -1.131 | 2.59E-01 | 4.12E-01 | -5.856 |
| GYPC | 0.123 | 9.885 | 1.129 | 2.60E-01 | 4.13E-01 | -5.857 |
| HAVCR2 | 0.118 | 7.567 | 1.128 | 2.60E-01 | 4.13E-01 | -5.857 |
| SERPINE1 | 0.246 | 10.666 | 1.128 | 2.60E-01 | 4.13E-01 | -5.858 |
| NPHS2 | 0.154 | 5.437 | 1.127 | 2.61E-01 | 4.14E-01 | -5.859 |
| LTBP1 | -0.156 | 8.252 | -1.128 | 2.61E-01 | 4.14E-01 | -5.859 |
| SCD | 0.159 | 8.45 | 1.127 | 2.61E-01 | 4.14E-01 | -5.86 |
| VWF | 0.115 | 9.214 | 1.127 | 2.61E-01 | 4.14E-01 | -5.86 |
| MOS | 0.142 | 6.706 | 1.126 | 2.61E-01 | 4.14E-01 | -5.861 |
| UHRF1 | 0.126 | 7.924 | 1.125 | 2.61E-01 | 4.14E-01 | -5.861 |
| SF1 | -0.081 | 9.205 | -1.126 | 2.62E-01 | 4.14E-01 | -5.861 |
| NNAT | 0.215 | 8.603 | 1.124 | 2.62E-01 | 4.15E-01 | -5.862 |
| ADCY8 | 0.133 | 4.586 | 1.124 | 2.62E-01 | 4.15E-01 | -5.863 |
| CCR2 | -0.115 | 6.15 | -1.125 | 2.62E-01 | 4.15E-01 | -5.863 |
| FBXO27 | 0.147 | 5.567 | 1.123 | 2.62E-01 | 4.15E-01 | -5.864 |
| ABHD1 | 0.115 | 5.716 | 1.123 | 2.63E-01 | 4.15E-01 | -5.864 |
| ZDHHC4 | -0.071 | 8.755 | -1.123 | 2.63E-01 | 4.15E-01 | -5.864 |
| B3GNT7 | 0.117 | 6.08 | 1.122 | 2.63E-01 | 4.16E-01 | -5.865 |
| ARHGDIA | 0.14 | 9.352 | 1.122 | 2.63E-01 | 4.16E-01 | -5.865 |
| PGM5 | 0.119 | 5.835 | 1.12 | 2.64E-01 | 4.17E-01 | -5.866 |
| RNF166 | -0.109 | 6.79 | -1.121 | 2.64E-01 | 4.17E-01 | -5.866 |
| FER | 0.115 | 6.967 | 1.12 | 2.64E-01 | 4.17E-01 | -5.867 |
| CDKN2C | 0.111 | 7.962 | 1.12 | 2.64E-01 | 4.17E-01 | -5.867 |
| TEX13B | 0.138 | 5.491 | 1.119 | 2.64E-01 | 4.17E-01 | -5.868 |
| CDH7 | 0.124 | 5.137 | 1.119 | 2.64E-01 | 4.17E-01 | -5.868 |
| TP53RK | -0.086 | 7.741 | -1.12 | 2.64E-01 | 4.17E-01 | -5.868 |
| PLA1A | 0.138 | 6.48 | 1.118 | 2.64E-01 | 4.17E-01 | -5.868 |
| KRT4 | 0.139 | 5.905 | 1.118 | 2.65E-01 | 4.18E-01 | -5.869 |
| KCNH1 | -0.179 | 5.174 | -1.118 | 2.65E-01 | 4.18E-01 | -5.87 |
| TPTE | 0.172 | 5.683 | 1.117 | 2.65E-01 | 4.18E-01 | -5.87 |
| MAPK13 | 0.132 | 8.488 | 1.116 | 2.65E-01 | 4.19E-01 | -5.871 |
| EPHB1 | 0.119 | 5.416 | 1.115 | 2.66E-01 | 4.19E-01 | -5.872 |
| SLC1A1 | 0.139 | 6.904 | 1.115 | 2.66E-01 | 4.19E-01 | -5.872 |
| TBC1D4 | 0.113 | 8.287 | 1.115 | 2.66E-01 | 4.19E-01 | -5.872 |
| PNLIP | -0.111 | 4.513 | -1.115 | 2.66E-01 | 4.19E-01 | -5.873 |
| GALE | 0.132 | 7.854 | 1.114 | 2.66E-01 | 4.19E-01 | -5.873 |
| PCCA | 0.127 | 9.335 | 1.113 | 2.66E-01 | 4.20E-01 | -5.874 |
| CDH18 | 0.157 | 4.92 | 1.112 | 2.67E-01 | 4.21E-01 | -5.876 |
| HIST1H1E | 0.16 | 7.998 | 1.111 | 2.67E-01 | 4.21E-01 | -5.876 |
| EFNA3 | 0.127 | 5.801 | 1.111 | 2.67E-01 | 4.21E-01 | -5.876 |
| FEN1 | 0.102 | 8.78 | 1.111 | 2.68E-01 | 4.21E-01 | -5.877 |
| SH3GL1 | 0.099 | 10.076 | 1.111 | 2.68E-01 | 4.21E-01 | -5.877 |
| RXRB | 0.089 | 9.397 | 1.11 | 2.68E-01 | 4.21E-01 | -5.877 |
| GPR78 | -0.162 | 7.223 | -1.111 | 2.68E-01 | 4.22E-01 | -5.878 |
| INSL5 | -0.132 | 5.015 | -1.11 | 2.68E-01 | 4.22E-01 | -5.879 |
| GPR155 | -0.091 | 7.322 | -1.11 | 2.68E-01 | 4.22E-01 | -5.879 |
| TCEAL1 | -0.107 | 8.267 | -1.11 | 2.69E-01 | 4.22E-01 | -5.879 |
| ARHGEF4 | -0.177 | 5.65 | -1.109 | 2.69E-01 | 4.22E-01 | -5.88 |
| TSGA13 | -0.101 | 5.378 | -1.109 | 2.69E-01 | 4.22E-01 | -5.88 |
| BAZ2A | -0.09 | 7.999 | -1.106 | 2.70E-01 | 4.24E-01 | -5.883 |
| HM13 | -0.08 | 8.646 | -1.106 | 2.70E-01 | 4.24E-01 | -5.883 |
| CCDC13 | 0.13 | 5.738 | 1.105 | 2.70E-01 | 4.24E-01 | -5.883 |
| ZNF426 | -0.103 | 7.843 | -1.106 | 2.70E-01 | 4.24E-01 | -5.884 |
| LY6E | 0.148 | 10.948 | 1.104 | 2.70E-01 | 4.24E-01 | -5.884 |
| ETS1 | -0.107 | 8.374 | -1.105 | 2.71E-01 | 4.25E-01 | -5.884 |
| CXorf21 | -0.139 | 6.399 | -1.105 | 2.71E-01 | 4.25E-01 | -5.885 |
| PLP1 | 0.168 | 4.889 | 1.103 | 2.71E-01 | 4.25E-01 | -5.885 |
| PRKG1 | 0.097 | 5.788 | 1.103 | 2.71E-01 | 4.25E-01 | -5.885 |
| OAS3 | -0.167 | 7.464 | -1.103 | 2.71E-01 | 4.25E-01 | -5.886 |
| RASGRP4 | 0.107 | 6.711 | 1.102 | 2.71E-01 | 4.25E-01 | -5.886 |
| BMP6 | -0.146 | 8.626 | -1.102 | 2.72E-01 | 4.26E-01 | -5.887 |
| HIST1H2AC | -0.153 | 8.654 | -1.102 | 2.72E-01 | 4.26E-01 | -5.888 |
| NUMB | -0.085 | 9.519 | -1.101 | 2.72E-01 | 4.26E-01 | -5.888 |
| MKKS | 0.08 | 8.362 | 1.1 | 2.72E-01 | 4.26E-01 | -5.888 |
| FJX1 | 0.178 | 8.068 | 1.099 | 2.73E-01 | 4.27E-01 | -5.889 |
| STK17A | -0.115 | 7.479 | -1.1 | 2.73E-01 | 4.27E-01 | -5.89 |
| BRCA2 | -0.105 | 5.915 | -1.098 | 2.74E-01 | 4.28E-01 | -5.892 |
| ATP6V1E1 | 0.089 | 11.076 | 1.096 | 2.74E-01 | 4.28E-01 | -5.892 |
| PPP1R14C | -0.174 | 7.004 | -1.096 | 2.74E-01 | 4.29E-01 | -5.894 |
| C1QTNF1 | -0.161 | 7.801 | -1.095 | 2.75E-01 | 4.30E-01 | -5.895 |
| POLD3 | 0.083 | 8.225 | 1.094 | 2.75E-01 | 4.30E-01 | -5.895 |
| DKK2 | 0.166 | 5.328 | 1.093 | 2.75E-01 | 4.30E-01 | -5.896 |
| SAFB | 0.145 | 9.385 | 1.093 | 2.75E-01 | 4.30E-01 | -5.896 |
| PPBP | -0.165 | 8.157 | -1.094 | 2.75E-01 | 4.30E-01 | -5.896 |
| PPARGC1A | -0.154 | 4.918 | -1.093 | 2.76E-01 | 4.30E-01 | -5.897 |
| EPHA10 | 0.095 | 5.177 | 1.092 | 2.76E-01 | 4.30E-01 | -5.897 |
| SLC39A13 | -0.075 | 9.866 | -1.092 | 2.76E-01 | 4.31E-01 | -5.898 |
| ASMT | -0.13 | 6.028 | -1.091 | 2.76E-01 | 4.31E-01 | -5.899 |
| CDC40 | -0.113 | 8.189 | -1.091 | 2.76E-01 | 4.31E-01 | -5.899 |
| CAMSAP1 | -0.08 | 7.255 | -1.091 | 2.77E-01 | 4.32E-01 | -5.899 |
| SLCO2B1 | -0.098 | 7.711 | -1.091 | 2.77E-01 | 4.32E-01 | -5.899 |
| CLCA2 | -0.114 | 5.072 | -1.09 | 2.77E-01 | 4.32E-01 | -5.9 |
| BMP4 | 0.132 | 7.187 | 1.089 | 2.77E-01 | 4.32E-01 | -5.9 |
| ACSL6 | 0.089 | 5.231 | 1.088 | 2.77E-01 | 4.32E-01 | -5.901 |
| TPK1 | 0.102 | 7.884 | 1.088 | 2.78E-01 | 4.32E-01 | -5.901 |
| MS4A12 | 0.138 | 4.762 | 1.088 | 2.78E-01 | 4.32E-01 | -5.901 |
| POLE | 0.104 | 7.174 | 1.087 | 2.78E-01 | 4.33E-01 | -5.903 |
| ANKRD35 | 0.144 | 7.143 | 1.087 | 2.78E-01 | 4.33E-01 | -5.903 |
| CLEC7A | -0.107 | 6.197 | -1.087 | 2.79E-01 | 4.34E-01 | -5.904 |
| GNG5 | 0.087 | 10.93 | 1.084 | 2.79E-01 | 4.34E-01 | -5.905 |
| ZPBP2 | -0.098 | 4.485 | -1.085 | 2.79E-01 | 4.35E-01 | -5.906 |
| AGA | -0.095 | 8.537 | -1.083 | 2.80E-01 | 4.36E-01 | -5.907 |
| MTA1 | 0.094 | 9.312 | 1.082 | 2.80E-01 | 4.36E-01 | -5.907 |
| CPNE2 | 0.149 | 8.109 | 1.082 | 2.80E-01 | 4.36E-01 | -5.908 |
| ARID3B | -0.087 | 9.706 | -1.082 | 2.80E-01 | 4.36E-01 | -5.908 |
| SERTAD2 | -0.089 | 9.785 | -1.082 | 2.81E-01 | 4.36E-01 | -5.909 |
| ACAA2 | -0.132 | 9.018 | -1.081 | 2.81E-01 | 4.37E-01 | -5.91 |
| LIPF | 0.101 | 4.854 | 1.08 | 2.81E-01 | 4.37E-01 | -5.91 |
| PGAM2 | -0.124 | 5.859 | -1.081 | 2.81E-01 | 4.37E-01 | -5.91 |
| SH3BP5 | -0.116 | 8.705 | -1.079 | 2.82E-01 | 4.37E-01 | -5.911 |
| PCDHGA8 | 0.11 | 6.096 | 1.078 | 2.82E-01 | 4.37E-01 | -5.911 |
| HSD17B2 | 0.17 | 10.051 | 1.078 | 2.82E-01 | 4.38E-01 | -5.912 |
| MRVI1 | 0.154 | 8.116 | 1.077 | 2.82E-01 | 4.38E-01 | -5.913 |
| SDPR | -0.157 | 8.674 | -1.078 | 2.82E-01 | 4.38E-01 | -5.913 |
| GUCY2C | 0.117 | 4.757 | 1.076 | 2.83E-01 | 4.38E-01 | -5.913 |
| NAT1 | -0.151 | 7.055 | -1.077 | 2.83E-01 | 4.38E-01 | -5.913 |
| TDRD7 | 0.097 | 9.093 | 1.075 | 2.83E-01 | 4.39E-01 | -5.915 |
| GIMAP4 | 0.13 | 9.343 | 1.075 | 2.83E-01 | 4.39E-01 | -5.915 |
| MYO9B | 0.104 | 8.187 | 1.075 | 2.83E-01 | 4.39E-01 | -5.915 |
| PHYHIPL | -0.156 | 7.464 | -1.075 | 2.84E-01 | 4.39E-01 | -5.916 |
| HLA-DQB1 | 0.2 | 6.737 | 1.074 | 2.84E-01 | 4.39E-01 | -5.916 |
| TCL1A | 0.147 | 6.99 | 1.074 | 2.84E-01 | 4.40E-01 | -5.916 |
| CDC42SE1 | 0.105 | 9.544 | 1.073 | 2.84E-01 | 4.40E-01 | -5.917 |
| IL1RAPL2 | 0.126 | 5.274 | 1.072 | 2.84E-01 | 4.40E-01 | -5.918 |
| PRSS8 | 0.156 | 9.683 | 1.072 | 2.85E-01 | 4.40E-01 | -5.918 |
| BRD9 | 0.078 | 8.192 | 1.071 | 2.85E-01 | 4.41E-01 | -5.919 |
| ZNF532 | -0.088 | 7.821 | -1.072 | 2.85E-01 | 4.41E-01 | -5.919 |
| RAD9A | 0.091 | 7.725 | 1.071 | 2.85E-01 | 4.41E-01 | -5.92 |
| CDKL1 | -0.12 | 6.477 | -1.071 | 2.85E-01 | 4.41E-01 | -5.92 |
| IL5 | -0.124 | 4.702 | -1.07 | 2.86E-01 | 4.42E-01 | -5.921 |
| GNG13 | 0.173 | 6.921 | 1.069 | 2.86E-01 | 4.42E-01 | -5.921 |
| MAGEC3 | 0.108 | 4.828 | 1.069 | 2.86E-01 | 4.42E-01 | -5.921 |
| FKBP5 | -0.21 | 7.852 | -1.069 | 2.86E-01 | 4.42E-01 | -5.922 |
| DRP2 | -0.129 | 6.065 | -1.069 | 2.86E-01 | 4.42E-01 | -5.922 |
| CTSD | 0.132 | 10.473 | 1.068 | 2.86E-01 | 4.42E-01 | -5.922 |
| SLCO4A1 | -0.243 | 8.081 | -1.069 | 2.87E-01 | 4.42E-01 | -5.923 |
| ALAS2 | 0.171 | 8.959 | 1.067 | 2.87E-01 | 4.43E-01 | -5.923 |
| FBXO36 | -0.093 | 5.848 | -1.067 | 2.87E-01 | 4.43E-01 | -5.924 |
| SCGB2A2 | -0.109 | 4.614 | -1.067 | 2.87E-01 | 4.43E-01 | -5.924 |
| TNNC2 | 0.138 | 6.237 | 1.066 | 2.87E-01 | 4.43E-01 | -5.924 |
| AP1G2 | 0.118 | 8.853 | 1.066 | 2.87E-01 | 4.43E-01 | -5.925 |
| BRDT | 0.084 | 5.054 | 1.066 | 2.87E-01 | 4.43E-01 | -5.925 |
| MAGEB4 | 0.095 | 4.651 | 1.065 | 2.88E-01 | 4.43E-01 | -5.925 |
| CLN6 | 0.087 | 7.824 | 1.065 | 2.88E-01 | 4.44E-01 | -5.926 |
| GTPBP3 | -0.094 | 8.175 | -1.065 | 2.88E-01 | 4.44E-01 | -5.927 |
| WDR18 | -0.121 | 9.96 | -1.064 | 2.88E-01 | 4.44E-01 | -5.927 |
| BACH2 | -0.102 | 6.184 | -1.064 | 2.89E-01 | 4.44E-01 | -5.927 |
| PDZK1 | -0.162 | 5.737 | -1.063 | 2.89E-01 | 4.45E-01 | -5.928 |
| ZNF77 | -0.088 | 7.591 | -1.063 | 2.89E-01 | 4.45E-01 | -5.929 |
| EGFR | 0.108 | 8.508 | 1.061 | 2.89E-01 | 4.45E-01 | -5.929 |
| EFHD2 | -0.105 | 10.905 | -1.062 | 2.89E-01 | 4.45E-01 | -5.929 |
| AGT | 0.166 | 5.664 | 1.061 | 2.89E-01 | 4.45E-01 | -5.929 |
| GRB2 | 0.08 | 10.084 | 1.061 | 2.90E-01 | 4.45E-01 | -5.93 |
| PKHD1L1 | -0.118 | 4.852 | -1.061 | 2.90E-01 | 4.46E-01 | -5.93 |
| HAO2 | 0.1 | 4.73 | 1.059 | 2.91E-01 | 4.47E-01 | -5.932 |
| L3MBTL2 | 0.078 | 7.566 | 1.059 | 2.91E-01 | 4.47E-01 | -5.932 |
| DAG1 | 0.105 | 8.922 | 1.058 | 2.91E-01 | 4.47E-01 | -5.932 |
| PRKCG | 0.118 | 5.648 | 1.058 | 2.91E-01 | 4.47E-01 | -5.933 |
| TIGD4 | -0.127 | 4.86 | -1.058 | 2.91E-01 | 4.47E-01 | -5.934 |
| ADAM2 | -0.15 | 4.797 | -1.058 | 2.91E-01 | 4.48E-01 | -5.934 |
| SPCS1 | -0.074 | 11.645 | -1.057 | 2.92E-01 | 4.48E-01 | -5.935 |
| NDRG2 | 0.135 | 8.512 | 1.056 | 2.92E-01 | 4.48E-01 | -5.935 |
| GIPR | 0.148 | 6.7 | 1.056 | 2.92E-01 | 4.48E-01 | -5.935 |
| SATB1 | 0.116 | 7.337 | 1.055 | 2.92E-01 | 4.48E-01 | -5.935 |
| PLSCR2 | 0.095 | 4.731 | 1.055 | 2.92E-01 | 4.48E-01 | -5.935 |
| FBXO24 | 0.1 | 5.822 | 1.055 | 2.92E-01 | 4.48E-01 | -5.935 |
| PAPSS1 | 0.133 | 8.823 | 1.055 | 2.92E-01 | 4.48E-01 | -5.936 |
| POLB | 0.093 | 7.689 | 1.055 | 2.92E-01 | 4.48E-01 | -5.936 |
| MYO3B | -0.086 | 4.987 | -1.055 | 2.93E-01 | 4.49E-01 | -5.937 |
| NFRKB | 0.101 | 6.946 | 1.054 | 2.93E-01 | 4.49E-01 | -5.937 |
| SPRED2 | 0.118 | 6.755 | 1.054 | 2.93E-01 | 4.49E-01 | -5.937 |
| SLC22A6 | 0.118 | 5.435 | 1.053 | 2.93E-01 | 4.49E-01 | -5.937 |
| PLAC8 | 0.201 | 9.47 | 1.052 | 2.94E-01 | 4.50E-01 | -5.939 |
| OLIG3 | 0.121 | 4.809 | 1.051 | 2.94E-01 | 4.50E-01 | -5.939 |
| CRISPLD1 | -0.168 | 5.709 | -1.052 | 2.94E-01 | 4.50E-01 | -5.94 |
| MTA2 | 0.1 | 8.692 | 1.05 | 2.95E-01 | 4.51E-01 | -5.941 |
| SNTB1 | -0.14 | 7.073 | -1.05 | 2.95E-01 | 4.51E-01 | -5.942 |
| WDR5 | 0.071 | 7.693 | 1.049 | 2.95E-01 | 4.51E-01 | -5.942 |
| C21orf2 | 0.117 | 7.499 | 1.049 | 2.95E-01 | 4.51E-01 | -5.942 |
| PHC2 | -0.111 | 8.189 | -1.05 | 2.95E-01 | 4.51E-01 | -5.942 |
| CYP4F3 | -0.133 | 5.619 | -1.049 | 2.95E-01 | 4.51E-01 | -5.943 |
| CPEB3 | -0.114 | 6.819 | -1.049 | 2.95E-01 | 4.51E-01 | -5.943 |
| ZIC1 | -0.088 | 4.828 | -1.049 | 2.95E-01 | 4.51E-01 | -5.943 |
| NME1 | 0.116 | 10.238 | 1.048 | 2.96E-01 | 4.51E-01 | -5.943 |
| FBXO7 | 0.088 | 10.449 | 1.047 | 2.96E-01 | 4.52E-01 | -5.944 |
| PARVB | 0.131 | 7.308 | 1.046 | 2.96E-01 | 4.52E-01 | -5.945 |
| CRHBP | -0.18 | 6.549 | -1.047 | 2.96E-01 | 4.52E-01 | -5.945 |
| PCDH19 | 0.171 | 5.731 | 1.046 | 2.97E-01 | 4.53E-01 | -5.945 |
| TNR | -0.113 | 5.524 | -1.046 | 2.97E-01 | 4.53E-01 | -5.946 |
| CREB3L3 | 0.12 | 5.708 | 1.045 | 2.97E-01 | 4.53E-01 | -5.946 |
| TAPBP | 0.107 | 8.348 | 1.044 | 2.97E-01 | 4.53E-01 | -5.947 |
| TESK2 | -0.101 | 7.902 | -1.044 | 2.98E-01 | 4.54E-01 | -5.948 |
| KLK10 | -0.114 | 5.035 | -1.044 | 2.98E-01 | 4.54E-01 | -5.948 |
| PGBD1 | -0.125 | 6.07 | -1.043 | 2.98E-01 | 4.54E-01 | -5.949 |
| IL4R | -0.098 | 9.674 | -1.043 | 2.98E-01 | 4.54E-01 | -5.949 |
| GUCY2D | -0.09 | 4.784 | -1.043 | 2.98E-01 | 4.54E-01 | -5.949 |
| PPIE | -0.085 | 8.155 | -1.043 | 2.98E-01 | 4.54E-01 | -5.949 |
| BAG1 | 0.082 | 9.207 | 1.042 | 2.98E-01 | 4.54E-01 | -5.95 |
| COX6B2 | 0.118 | 6.513 | 1.041 | 2.99E-01 | 4.55E-01 | -5.95 |
| NEGR1 | 0.123 | 5.823 | 1.04 | 2.99E-01 | 4.55E-01 | -5.951 |
| CHD3 | 0.104 | 8.14 | 1.04 | 2.99E-01 | 4.55E-01 | -5.951 |
| ABCA9 | -0.115 | 6.537 | -1.04 | 3.00E-01 | 4.56E-01 | -5.952 |
| CTDSPL | 0.122 | 9.074 | 1.039 | 3.00E-01 | 4.56E-01 | -5.952 |
| PPP1R3D | 0.084 | 8.528 | 1.038 | 3.00E-01 | 4.56E-01 | -5.953 |
| ZMYND12 | 0.135 | 5.647 | 1.038 | 3.00E-01 | 4.56E-01 | -5.953 |
| PMCHL1 | -0.112 | 5.309 | -1.038 | 3.00E-01 | 4.57E-01 | -5.954 |
| AREG | 0.147 | 8.163 | 1.037 | 3.01E-01 | 4.57E-01 | -5.955 |
| FXN | 0.095 | 6.913 | 1.037 | 3.01E-01 | 4.57E-01 | -5.955 |
| COL4A6 | 0.119 | 5.757 | 1.036 | 3.01E-01 | 4.57E-01 | -5.955 |
| HYAL4 | -0.096 | 6.669 | -1.037 | 3.01E-01 | 4.57E-01 | -5.955 |
| NME7 | -0.108 | 7.603 | -1.037 | 3.01E-01 | 4.57E-01 | -5.956 |
| KIF2B | 0.202 | 5.208 | 1.035 | 3.01E-01 | 4.58E-01 | -5.956 |
| STRBP | -0.111 | 7.558 | -1.036 | 3.02E-01 | 4.58E-01 | -5.957 |
| CILP2 | 0.149 | 5.686 | 1.034 | 3.02E-01 | 4.58E-01 | -5.957 |
| CD3G | 0.167 | 6.444 | 1.034 | 3.02E-01 | 4.58E-01 | -5.957 |
| SLC8A3 | 0.116 | 5.589 | 1.034 | 3.02E-01 | 4.58E-01 | -5.957 |
| SCPEP1 | -0.119 | 8.705 | -1.034 | 3.02E-01 | 4.58E-01 | -5.958 |
| SHQ1 | -0.102 | 7.423 | -1.034 | 3.03E-01 | 4.59E-01 | -5.958 |
| F2RL1 | -0.211 | 6.692 | -1.033 | 3.03E-01 | 4.59E-01 | -5.959 |
| AIPL1 | 0.088 | 4.844 | 1.03 | 3.04E-01 | 4.60E-01 | -5.961 |
| FLT1 | 0.179 | 8.557 | 1.03 | 3.04E-01 | 4.61E-01 | -5.961 |
| TDRD1 | -0.203 | 5.951 | -1.03 | 3.04E-01 | 4.61E-01 | -5.962 |
| CSPG4 | 0.119 | 7.517 | 1.029 | 3.04E-01 | 4.61E-01 | -5.962 |
| FBXO34 | 0.082 | 8.651 | 1.029 | 3.04E-01 | 4.61E-01 | -5.962 |
| XRCC5 | -0.075 | 10.641 | -1.03 | 3.05E-01 | 4.61E-01 | -5.963 |
| ZNF225 | -0.115 | 6.156 | -1.029 | 3.05E-01 | 4.61E-01 | -5.963 |
| NRXN3 | -0.117 | 5.707 | -1.028 | 3.05E-01 | 4.62E-01 | -5.964 |
| PCDHB9 | 0.107 | 5.604 | 1.027 | 3.05E-01 | 4.62E-01 | -5.964 |
| FGFR3 | 0.118 | 6.972 | 1.027 | 3.05E-01 | 4.62E-01 | -5.965 |
| SUSD2 | 0.124 | 7.181 | 1.026 | 3.06E-01 | 4.62E-01 | -5.965 |
| GRIK2 | 0.095 | 5.178 | 1.026 | 3.06E-01 | 4.62E-01 | -5.965 |
| EFS | 0.132 | 8.483 | 1.026 | 3.06E-01 | 4.62E-01 | -5.965 |
| STK32A | 0.104 | 4.842 | 1.025 | 3.06E-01 | 4.62E-01 | -5.966 |
| TCL1B | 0.153 | 7.323 | 1.025 | 3.06E-01 | 4.62E-01 | -5.966 |
| RTN4RL2 | 0.122 | 6.771 | 1.025 | 3.06E-01 | 4.63E-01 | -5.967 |
| SSTR1 | 0.176 | 6.305 | 1.024 | 3.06E-01 | 4.63E-01 | -5.967 |
| SCGN | 0.222 | 6.284 | 1.024 | 3.06E-01 | 4.63E-01 | -5.967 |
| PRM1 | 0.136 | 5.646 | 1.023 | 3.07E-01 | 4.63E-01 | -5.968 |
| SLC34A2 | 0.138 | 5.61 | 1.023 | 3.07E-01 | 4.63E-01 | -5.968 |
| SYNPO2L | -0.17 | 6.571 | -1.024 | 3.07E-01 | 4.63E-01 | -5.968 |
| SMAD7 | -0.101 | 9.13 | -1.023 | 3.07E-01 | 4.64E-01 | -5.969 |
| SLC9A7 | 0.108 | 6.15 | 1.021 | 3.08E-01 | 4.65E-01 | -5.971 |
| KCNIP2 | 0.113 | 5.381 | 1.02 | 3.08E-01 | 4.65E-01 | -5.971 |
| NXF3 | 0.123 | 6.339 | 1.02 | 3.09E-01 | 4.65E-01 | -5.971 |
| NGFRAP1 | 0.12 | 11.844 | 1.019 | 3.09E-01 | 4.66E-01 | -5.972 |
| DCLRE1A | -0.097 | 7.878 | -1.02 | 3.09E-01 | 4.66E-01 | -5.972 |
| ALG8 | 0.092 | 9.503 | 1.018 | 3.10E-01 | 4.66E-01 | -5.973 |
| TMPRSS3 | -0.156 | 5.268 | -1.018 | 3.10E-01 | 4.67E-01 | -5.975 |
| BICD1 | -0.125 | 6.489 | -1.018 | 3.10E-01 | 4.67E-01 | -5.975 |
| CCND1 | 0.122 | 9.458 | 1.017 | 3.10E-01 | 4.67E-01 | -5.975 |
| PPP4R1L | -0.122 | 5.612 | -1.017 | 3.10E-01 | 4.67E-01 | -5.975 |
| CHRNB3 | 0.12 | 5.522 | 1.016 | 3.11E-01 | 4.67E-01 | -5.975 |
| ARHGEF1 | -0.106 | 7.56 | -1.016 | 3.11E-01 | 4.68E-01 | -5.977 |
| ATP10A | -0.093 | 6.2 | -1.015 | 3.11E-01 | 4.68E-01 | -5.977 |
| PCDH10 | 0.123 | 6.206 | 1.014 | 3.11E-01 | 4.68E-01 | -5.977 |
| TIMM13 | 0.088 | 8.511 | 1.012 | 3.12E-01 | 4.69E-01 | -5.979 |
| ZNF418 | 0.129 | 7.712 | 1.012 | 3.12E-01 | 4.69E-01 | -5.979 |
| DLG2 | 0.097 | 5.341 | 1.012 | 3.12E-01 | 4.69E-01 | -5.979 |
| THBS1 | 0.158 | 8.908 | 1.012 | 3.12E-01 | 4.69E-01 | -5.979 |
| PDP2 | 0.091 | 6.313 | 1.011 | 3.13E-01 | 4.70E-01 | -5.98 |
| WFDC10B | 0.118 | 5.687 | 1.011 | 3.13E-01 | 4.70E-01 | -5.98 |
| FANK1 | -0.113 | 6.076 | -1.012 | 3.13E-01 | 4.70E-01 | -5.98 |
| ANXA13 | 0.147 | 4.932 | 1.01 | 3.13E-01 | 4.71E-01 | -5.981 |
| TM4SF1 | 0.135 | 10.523 | 1.01 | 3.13E-01 | 4.71E-01 | -5.982 |
| KCNK3 | 0.136 | 7.516 | 1.009 | 3.14E-01 | 4.71E-01 | -5.982 |
| DMC1 | 0.114 | 5.309 | 1.007 | 3.14E-01 | 4.72E-01 | -5.984 |
| AGER | 0.11 | 6.907 | 1.007 | 3.15E-01 | 4.72E-01 | -5.984 |
| ADCK1 | 0.119 | 6.729 | 1.007 | 3.15E-01 | 4.72E-01 | -5.984 |
| GPR32 | 0.133 | 5.912 | 1.006 | 3.15E-01 | 4.72E-01 | -5.985 |
| TCF15 | -0.129 | 7.137 | -1.006 | 3.15E-01 | 4.73E-01 | -5.986 |
| INSIG1 | -0.131 | 10.194 | -1.006 | 3.16E-01 | 4.73E-01 | -5.986 |
| DEDD | 0.088 | 8.195 | 1.005 | 3.16E-01 | 4.73E-01 | -5.986 |
| DHFR | -0.112 | 7.784 | -1.005 | 3.16E-01 | 4.73E-01 | -5.987 |
| ZNF408 | 0.126 | 6.681 | 1.004 | 3.16E-01 | 4.74E-01 | -5.987 |
| PAK3 | -0.17 | 6.054 | -1.004 | 3.17E-01 | 4.74E-01 | -5.988 |
| TREH | -0.109 | 4.79 | -1.004 | 3.17E-01 | 4.74E-01 | -5.988 |
| AKAP8 | 0.095 | 9.387 | 1.003 | 3.17E-01 | 4.74E-01 | -5.988 |
| MFN2 | 0.08 | 9.98 | 1.003 | 3.17E-01 | 4.74E-01 | -5.988 |
| NSFL1C | 0.1 | 9.304 | 1.002 | 3.17E-01 | 4.74E-01 | -5.989 |
| KHDRBS2 | -0.143 | 5.213 | -1.003 | 3.17E-01 | 4.74E-01 | -5.989 |
| GPR153 | -0.098 | 8.128 | -1.003 | 3.17E-01 | 4.74E-01 | -5.989 |
| PIP5KL1 | 0.163 | 6.539 | 1.001 | 3.18E-01 | 4.75E-01 | -5.99 |
| NFIA | -0.109 | 7.678 | -1.002 | 3.18E-01 | 4.75E-01 | -5.99 |
| RPS15 | 0.076 | 11.997 | 0.999 | 3.18E-01 | 4.76E-01 | -5.991 |
| NAALAD2 | 0.097 | 6.303 | 0.999 | 3.18E-01 | 4.76E-01 | -5.992 |
| S100G | 0.1 | 4.84 | 0.999 | 3.19E-01 | 4.76E-01 | -5.992 |
| IL18RAP | 0.157 | 6.997 | 0.998 | 3.19E-01 | 4.76E-01 | -5.993 |
| BID | -0.096 | 8.801 | -0.999 | 3.19E-01 | 4.76E-01 | -5.993 |
| IGFBP1 | -0.487 | 7.779 | -0.999 | 3.19E-01 | 4.76E-01 | -5.993 |
| GOLGA1 | 0.097 | 7.298 | 0.997 | 3.19E-01 | 4.77E-01 | -5.994 |
| ATXN3 | -0.079 | 7.394 | -0.998 | 3.20E-01 | 4.77E-01 | -5.994 |
| FOXD2 | 0.117 | 5.948 | 0.996 | 3.20E-01 | 4.78E-01 | -5.995 |
| TTL | 0.09 | 7.675 | 0.995 | 3.20E-01 | 4.78E-01 | -5.996 |
| WWTR1 | -0.077 | 7.269 | -0.995 | 3.21E-01 | 4.78E-01 | -5.996 |
| CXorf36 | 0.093 | 6.142 | 0.994 | 3.21E-01 | 4.79E-01 | -5.997 |
| KCNMB3 | -0.118 | 6.387 | -0.995 | 3.21E-01 | 4.79E-01 | -5.997 |
| PAPSS2 | 0.128 | 9.507 | 0.993 | 3.21E-01 | 4.79E-01 | -5.997 |
| IDE | -0.071 | 7.98 | -0.994 | 3.21E-01 | 4.79E-01 | -5.997 |
| MAP7 | -0.084 | 7.496 | -0.994 | 3.21E-01 | 4.79E-01 | -5.998 |
| PPP1CA | 0.106 | 10.839 | 0.993 | 3.21E-01 | 4.79E-01 | -5.998 |
| GP6 | 0.132 | 6.249 | 0.992 | 3.22E-01 | 4.80E-01 | -5.999 |
| RPL29 | 0.078 | 11.407 | 0.991 | 3.22E-01 | 4.80E-01 | -5.999 |
| OLR1 | 0.175 | 8.902 | 0.99 | 3.23E-01 | 4.80E-01 | -6 |
| IRAK2 | 0.161 | 6.589 | 0.99 | 3.23E-01 | 4.80E-01 | -6.001 |
| CCDC9 | 0.114 | 7.768 | 0.99 | 3.23E-01 | 4.81E-01 | -6.001 |
| SIX4 | -0.139 | 6.366 | -0.99 | 3.23E-01 | 4.81E-01 | -6.001 |
| NAALADL1 | 0.111 | 6.048 | 0.989 | 3.23E-01 | 4.81E-01 | -6.001 |
| HIST1H4G | 0.1 | 4.793 | 0.989 | 3.23E-01 | 4.81E-01 | -6.001 |
| GJB1 | -0.133 | 5.135 | -0.99 | 3.23E-01 | 4.81E-01 | -6.002 |
| GZMH | 0.19 | 6.943 | 0.989 | 3.24E-01 | 4.81E-01 | -6.002 |
| DNAJC13 | -0.096 | 9.482 | -0.989 | 3.24E-01 | 4.81E-01 | -6.002 |
| UBN1 | -0.076 | 9.667 | -0.989 | 3.24E-01 | 4.81E-01 | -6.002 |
| SLC7A6 | 0.094 | 7.629 | 0.988 | 3.24E-01 | 4.81E-01 | -6.003 |
| GPA33 | -0.106 | 5.663 | -0.988 | 3.24E-01 | 4.81E-01 | -6.003 |
| ELF3 | 0.14 | 7.974 | 0.986 | 3.25E-01 | 4.82E-01 | -6.004 |
| ARMCX6 | 0.09 | 9.209 | 0.986 | 3.25E-01 | 4.82E-01 | -6.005 |
| RPS23 | -0.114 | 12.097 | -0.987 | 3.25E-01 | 4.82E-01 | -6.005 |
| RNF133 | 0.075 | 4.531 | 0.984 | 3.26E-01 | 4.83E-01 | -6.006 |
| TNFAIP6 | -0.169 | 6.634 | -0.985 | 3.26E-01 | 4.84E-01 | -6.007 |
| IL18BP | 0.094 | 7.757 | 0.984 | 3.26E-01 | 4.84E-01 | -6.007 |
| COTL1 | -0.117 | 9.631 | -0.983 | 3.27E-01 | 4.84E-01 | -6.008 |
| ST8SIA3 | -0.09 | 5.399 | -0.983 | 3.27E-01 | 4.84E-01 | -6.008 |
| FCER1A | 0.124 | 5.677 | 0.981 | 3.27E-01 | 4.85E-01 | -6.009 |
| MRRF | 0.073 | 7.782 | 0.98 | 3.28E-01 | 4.85E-01 | -6.01 |
| ITGAX | -0.107 | 6.605 | -0.981 | 3.28E-01 | 4.85E-01 | -6.01 |
| CH25H | 0.155 | 6.06 | 0.979 | 3.28E-01 | 4.86E-01 | -6.011 |
| PCYT1A | 0.074 | 7.385 | 0.979 | 3.28E-01 | 4.86E-01 | -6.011 |
| CSF2 | -0.235 | 5.372 | -0.98 | 3.28E-01 | 4.86E-01 | -6.011 |
| SLC39A3 | 0.114 | 7.353 | 0.978 | 3.29E-01 | 4.86E-01 | -6.012 |
| TNFRSF1B | -0.134 | 8.608 | -0.979 | 3.29E-01 | 4.86E-01 | -6.012 |
| PRG3 | 0.119 | 5.561 | 0.978 | 3.29E-01 | 4.86E-01 | -6.012 |
| ETV6 | 0.092 | 7.797 | 0.977 | 3.29E-01 | 4.87E-01 | -6.013 |
| FKBP7 | -0.114 | 7.241 | -0.978 | 3.29E-01 | 4.87E-01 | -6.013 |
| TNFRSF10B | 0.094 | 8.667 | 0.977 | 3.29E-01 | 4.87E-01 | -6.013 |
| S100P | 0.148 | 12.753 | 0.977 | 3.29E-01 | 4.87E-01 | -6.013 |
| ATP8B4 | -0.12 | 7.363 | -0.978 | 3.29E-01 | 4.87E-01 | -6.013 |
| CREB3L4 | 0.065 | 7.921 | 0.976 | 3.30E-01 | 4.87E-01 | -6.014 |
| EP400 | 0.081 | 7.807 | 0.975 | 3.30E-01 | 4.88E-01 | -6.015 |
| SLC13A2 | 0.098 | 5.823 | 0.975 | 3.30E-01 | 4.88E-01 | -6.015 |
| SLC16A7 | -0.105 | 5.55 | -0.975 | 3.31E-01 | 4.88E-01 | -6.016 |
| ADAMTS10 | 0.129 | 6.195 | 0.973 | 3.31E-01 | 4.89E-01 | -6.017 |
| PSORS1C2 | 0.132 | 6.329 | 0.973 | 3.31E-01 | 4.89E-01 | -6.017 |
| SDF2L1 | 0.105 | 11.265 | 0.972 | 3.32E-01 | 4.90E-01 | -6.018 |
| ASB11 | 0.123 | 4.802 | 0.972 | 3.32E-01 | 4.90E-01 | -6.018 |
| CAPN11 | 0.122 | 5.489 | 0.971 | 3.32E-01 | 4.90E-01 | -6.019 |
| ZNF157 | 0.134 | 6.18 | 0.971 | 3.32E-01 | 4.90E-01 | -6.019 |
| SCML4 | -0.097 | 5.807 | -0.972 | 3.32E-01 | 4.90E-01 | -6.019 |
| BTBD9 | -0.077 | 6.244 | -0.972 | 3.32E-01 | 4.90E-01 | -6.019 |
| AKT1S1 | -0.127 | 7.831 | -0.971 | 3.33E-01 | 4.90E-01 | -6.019 |
| NCBP1 | -0.064 | 8.564 | -0.971 | 3.33E-01 | 4.90E-01 | -6.02 |
| DDX42 | 0.08 | 9.46 | 0.97 | 3.33E-01 | 4.90E-01 | -6.02 |
| TBC1D7 | -0.08 | 8.832 | -0.971 | 3.33E-01 | 4.90E-01 | -6.02 |
| ARHGEF7 | -0.076 | 7.648 | -0.97 | 3.33E-01 | 4.91E-01 | -6.021 |
| SLC15A3 | 0.12 | 10.112 | 0.968 | 3.34E-01 | 4.91E-01 | -6.021 |
| FBXO22 | -0.089 | 7.36 | -0.968 | 3.34E-01 | 4.92E-01 | -6.023 |
| PANX1 | -0.106 | 7.79 | -0.968 | 3.34E-01 | 4.92E-01 | -6.023 |
| ZNF345 | 0.098 | 6.608 | 0.966 | 3.35E-01 | 4.92E-01 | -6.023 |
| CALR3 | 0.135 | 5.165 | 0.965 | 3.35E-01 | 4.93E-01 | -6.024 |
| PDE4B | -0.127 | 6.945 | -0.966 | 3.35E-01 | 4.93E-01 | -6.024 |
| ZCCHC14 | 0.084 | 8.224 | 0.965 | 3.35E-01 | 4.93E-01 | -6.025 |
| VSIG4 | 0.178 | 9.288 | 0.965 | 3.35E-01 | 4.93E-01 | -6.025 |
| PMFBP1 | -0.108 | 5.226 | -0.965 | 3.36E-01 | 4.94E-01 | -6.026 |
| ACTL6B | 0.114 | 5.92 | 0.963 | 3.36E-01 | 4.94E-01 | -6.026 |
| PDE6B | 0.101 | 5.919 | 0.962 | 3.36E-01 | 4.94E-01 | -6.027 |
| USHBP1 | 0.095 | 5.923 | 0.962 | 3.37E-01 | 4.95E-01 | -6.028 |
| DDIT4 | -0.195 | 10.039 | -0.963 | 3.37E-01 | 4.95E-01 | -6.028 |
| PHF2 | -0.063 | 8.889 | -0.962 | 3.37E-01 | 4.95E-01 | -6.028 |
| GZMB | 0.2 | 7.588 | 0.961 | 3.37E-01 | 4.95E-01 | -6.028 |
| HECW1 | 0.089 | 4.739 | 0.961 | 3.37E-01 | 4.95E-01 | -6.028 |
| KCTD16 | 0.084 | 4.744 | 0.96 | 3.37E-01 | 4.95E-01 | -6.029 |
| ITLN1 | 0.134 | 6.693 | 0.96 | 3.38E-01 | 4.95E-01 | -6.029 |
| PTK2B | -0.092 | 7.314 | -0.961 | 3.38E-01 | 4.96E-01 | -6.03 |
| F8 | 0.107 | 6.253 | 0.958 | 3.39E-01 | 4.97E-01 | -6.031 |
| TMSB10 | 0.08 | 13.86 | 0.958 | 3.39E-01 | 4.97E-01 | -6.031 |
| MTCH1 | 0.061 | 12.387 | 0.958 | 3.39E-01 | 4.97E-01 | -6.031 |
| RABGEF1 | -0.071 | 8.636 | -0.957 | 3.40E-01 | 4.98E-01 | -6.033 |
| CYSLTR1 | -0.101 | 6.304 | -0.956 | 3.40E-01 | 4.99E-01 | -6.034 |
| CHST8 | 0.146 | 6.281 | 0.953 | 3.41E-01 | 5.00E-01 | -6.036 |
| FCN2 | 0.081 | 5.413 | 0.953 | 3.41E-01 | 5.00E-01 | -6.036 |
| MRPS21 | 0.076 | 10.682 | 0.953 | 3.41E-01 | 5.00E-01 | -6.036 |
| EIF4A2 | -0.108 | 11.386 | -0.954 | 3.41E-01 | 5.00E-01 | -6.036 |
| NEK3 | -0.091 | 7.526 | -0.953 | 3.42E-01 | 5.00E-01 | -6.037 |
| TNPO3 | -0.085 | 8.236 | -0.953 | 3.42E-01 | 5.00E-01 | -6.037 |
| GALNT13 | 0.117 | 5.229 | 0.95 | 3.43E-01 | 5.01E-01 | -6.038 |
| PDLIM2 | 0.093 | 7.552 | 0.95 | 3.43E-01 | 5.01E-01 | -6.039 |
| H2AFJ | 0.092 | 9.163 | 0.95 | 3.43E-01 | 5.01E-01 | -6.039 |
| ENOSF1 | -0.092 | 9.259 | -0.951 | 3.43E-01 | 5.01E-01 | -6.039 |
| STARD3 | -0.074 | 8.671 | -0.95 | 3.43E-01 | 5.01E-01 | -6.039 |
| LGALS14 | -0.169 | 9.084 | -0.95 | 3.44E-01 | 5.02E-01 | -6.04 |
| ATXN2 | 0.102 | 9.278 | 0.948 | 3.44E-01 | 5.02E-01 | -6.04 |
| SFTPD | 0.113 | 6.03 | 0.947 | 3.44E-01 | 5.03E-01 | -6.041 |
| EME2 | 0.09 | 7.187 | 0.947 | 3.44E-01 | 5.03E-01 | -6.042 |
| ABCC3 | 0.146 | 7.742 | 0.947 | 3.44E-01 | 5.03E-01 | -6.042 |
| CCND3 | 0.085 | 10.25 | 0.946 | 3.45E-01 | 5.03E-01 | -6.042 |
| POU3F2 | 0.133 | 4.946 | 0.946 | 3.45E-01 | 5.04E-01 | -6.043 |
| OSBPL1A | -0.092 | 6.734 | -0.946 | 3.45E-01 | 5.04E-01 | -6.043 |
| HS6ST3 | -0.081 | 4.999 | -0.945 | 3.46E-01 | 5.04E-01 | -6.044 |
| ENPEP | -0.105 | 6.385 | -0.945 | 3.46E-01 | 5.04E-01 | -6.044 |
| SLCO4C1 | -0.118 | 5.411 | -0.945 | 3.46E-01 | 5.05E-01 | -6.044 |
| KLHL21 | -0.089 | 8.735 | -0.944 | 3.46E-01 | 5.05E-01 | -6.045 |
| CASR | 0.097 | 5.119 | 0.943 | 3.46E-01 | 5.05E-01 | -6.045 |
| FCGRT | 0.109 | 10.926 | 0.943 | 3.46E-01 | 5.05E-01 | -6.045 |
| INHBC | 0.127 | 6.252 | 0.942 | 3.47E-01 | 5.06E-01 | -6.046 |
| ADRBK2 | -0.107 | 7.198 | -0.942 | 3.47E-01 | 5.06E-01 | -6.047 |
| MTIF3 | -0.079 | 9.701 | -0.942 | 3.47E-01 | 5.06E-01 | -6.047 |
| SHANK2 | -0.078 | 5.374 | -0.942 | 3.47E-01 | 5.06E-01 | -6.047 |
| SCNN1D | 0.11 | 6.552 | 0.94 | 3.48E-01 | 5.07E-01 | -6.048 |
| DOCK6 | 0.112 | 7.733 | 0.939 | 3.48E-01 | 5.07E-01 | -6.049 |
| DDX25 | 0.102 | 4.765 | 0.939 | 3.49E-01 | 5.07E-01 | -6.049 |
| RBM6 | -0.113 | 8.68 | -0.94 | 3.49E-01 | 5.07E-01 | -6.049 |
| POU6F2 | 0.104 | 5.66 | 0.938 | 3.49E-01 | 5.07E-01 | -6.049 |
| LDLR | 0.113 | 10.059 | 0.938 | 3.49E-01 | 5.07E-01 | -6.05 |
| ZNF100 | -0.107 | 6.52 | -0.939 | 3.49E-01 | 5.07E-01 | -6.05 |
| KCNJ10 | 0.093 | 5.482 | 0.937 | 3.49E-01 | 5.08E-01 | -6.05 |
| MBNL3 | 0.099 | 8.887 | 0.937 | 3.49E-01 | 5.08E-01 | -6.05 |
| PRKX | 0.097 | 8.203 | 0.937 | 3.50E-01 | 5.08E-01 | -6.051 |
| TRIO | -0.086 | 7.929 | -0.938 | 3.50E-01 | 5.08E-01 | -6.051 |
| TRIM7 | -0.102 | 5.973 | -0.937 | 3.50E-01 | 5.08E-01 | -6.052 |
| TLE6 | 0.131 | 6.842 | 0.936 | 3.50E-01 | 5.08E-01 | -6.052 |
| IL13 | 0.095 | 4.741 | 0.935 | 3.50E-01 | 5.09E-01 | -6.052 |
| CLK2 | -0.121 | 7.742 | -0.936 | 3.50E-01 | 5.09E-01 | -6.052 |
| DUSP11 | -0.074 | 8.635 | -0.935 | 3.51E-01 | 5.09E-01 | -6.053 |
| FN3K | 0.108 | 5.634 | 0.933 | 3.52E-01 | 5.10E-01 | -6.055 |
| HCN4 | -0.113 | 7.484 | -0.933 | 3.52E-01 | 5.11E-01 | -6.055 |
| DOC2A | -0.126 | 5.477 | -0.932 | 3.52E-01 | 5.11E-01 | -6.056 |
| SFXN5 | 0.108 | 7.056 | 0.931 | 3.52E-01 | 5.11E-01 | -6.056 |
| NOLC1 | -0.08 | 8.627 | -0.932 | 3.52E-01 | 5.11E-01 | -6.056 |
| RTTN | -0.085 | 6.862 | -0.931 | 3.53E-01 | 5.12E-01 | -6.057 |
| HIST1H2AK | -0.097 | 8.216 | -0.931 | 3.53E-01 | 5.12E-01 | -6.057 |
| BNC1 | 0.09 | 6.18 | 0.93 | 3.53E-01 | 5.12E-01 | -6.057 |
| ABCC2 | 0.114 | 6.136 | 0.929 | 3.53E-01 | 5.12E-01 | -6.058 |
| VN1R5 | 0.11 | 4.934 | 0.929 | 3.53E-01 | 5.12E-01 | -6.058 |
| BIVM | -0.095 | 7.453 | -0.93 | 3.54E-01 | 5.12E-01 | -6.058 |
| ZNF234 | -0.097 | 7.88 | -0.93 | 3.54E-01 | 5.12E-01 | -6.058 |
| RND1 | -0.121 | 5.019 | -0.929 | 3.54E-01 | 5.12E-01 | -6.058 |
| FOXP1 | 0.06 | 8.766 | 0.928 | 3.54E-01 | 5.12E-01 | -6.058 |
| SRPK1 | -0.096 | 10.421 | -0.929 | 3.54E-01 | 5.12E-01 | -6.059 |
| LAMB1 | -0.142 | 9.114 | -0.928 | 3.55E-01 | 5.13E-01 | -6.06 |
| MRPS7 | -0.071 | 9.695 | -0.927 | 3.55E-01 | 5.14E-01 | -6.061 |
| PTGER3 | -0.099 | 5.199 | -0.927 | 3.55E-01 | 5.14E-01 | -6.061 |
| DALRD3 | 0.075 | 7.966 | 0.925 | 3.56E-01 | 5.14E-01 | -6.061 |
| WDR13 | -0.09 | 8.596 | -0.926 | 3.56E-01 | 5.14E-01 | -6.062 |
| TALDO1 | -0.067 | 11.421 | -0.926 | 3.56E-01 | 5.14E-01 | -6.062 |
| LRRC15 | -0.216 | 5.807 | -0.926 | 3.56E-01 | 5.14E-01 | -6.062 |
| GALNT5 | 0.11 | 5.119 | 0.924 | 3.56E-01 | 5.14E-01 | -6.062 |
| PIGR | -0.104 | 5.075 | -0.925 | 3.56E-01 | 5.14E-01 | -6.062 |
| NR1H4 | -0.18 | 5.119 | -0.924 | 3.57E-01 | 5.15E-01 | -6.064 |
| CDC42EP3 | -0.105 | 7.962 | -0.923 | 3.57E-01 | 5.15E-01 | -6.064 |
| DHX9 | -0.102 | 8.806 | -0.923 | 3.57E-01 | 5.15E-01 | -6.064 |
| QPCT | -0.156 | 8.653 | -0.923 | 3.57E-01 | 5.15E-01 | -6.064 |
| HERC1 | -0.074 | 9.15 | -0.923 | 3.57E-01 | 5.15E-01 | -6.065 |
| PDYN | 0.082 | 4.969 | 0.921 | 3.58E-01 | 5.15E-01 | -6.065 |
| RBP7 | 0.129 | 8.261 | 0.921 | 3.58E-01 | 5.15E-01 | -6.065 |
| YWHAZ | -0.067 | 11.235 | -0.922 | 3.58E-01 | 5.15E-01 | -6.065 |
| SLC6A2 | 0.121 | 7.648 | 0.921 | 3.58E-01 | 5.15E-01 | -6.065 |
| LPIN2 | -0.097 | 8.87 | -0.922 | 3.58E-01 | 5.15E-01 | -6.065 |
| MGRN1 | -0.091 | 8.918 | -0.921 | 3.58E-01 | 5.16E-01 | -6.066 |
| PSMB10 | 0.086 | 11.364 | 0.92 | 3.58E-01 | 5.16E-01 | -6.066 |
| KLRC3 | 0.127 | 5.654 | 0.918 | 3.59E-01 | 5.17E-01 | -6.068 |
| COL13A1 | 0.134 | 6.417 | 0.917 | 3.60E-01 | 5.18E-01 | -6.069 |
| GRN | -0.112 | 12.984 | -0.918 | 3.60E-01 | 5.18E-01 | -6.069 |
| AFMID | -0.124 | 6.229 | -0.917 | 3.60E-01 | 5.19E-01 | -6.07 |
| BCAS1 | 0.114 | 4.9 | 0.915 | 3.61E-01 | 5.19E-01 | -6.07 |
| NGB | 0.11 | 5.619 | 0.915 | 3.61E-01 | 5.19E-01 | -6.07 |
| SLC4A5 | -0.095 | 6.066 | -0.916 | 3.61E-01 | 5.19E-01 | -6.071 |
| ANKH | 0.088 | 6.92 | 0.914 | 3.61E-01 | 5.19E-01 | -6.071 |
| SOX18 | 0.13 | 8.155 | 0.914 | 3.61E-01 | 5.19E-01 | -6.071 |
| HOXD9 | 0.121 | 5.859 | 0.913 | 3.62E-01 | 5.20E-01 | -6.072 |
| USP20 | 0.091 | 7.908 | 0.913 | 3.62E-01 | 5.20E-01 | -6.072 |
| AZGP1 | -0.103 | 5.52 | -0.914 | 3.62E-01 | 5.20E-01 | -6.072 |
| RASGEF1C | 0.086 | 5.721 | 0.912 | 3.62E-01 | 5.20E-01 | -6.073 |
| SMCR8 | -0.072 | 6.741 | -0.913 | 3.62E-01 | 5.20E-01 | -6.073 |
| CXCL14 | 0.157 | 8.686 | 0.912 | 3.62E-01 | 5.20E-01 | -6.073 |
| SOD2 | -0.173 | 9.198 | -0.913 | 3.62E-01 | 5.20E-01 | -6.073 |
| CREB3 | -0.079 | 8.439 | -0.913 | 3.62E-01 | 5.20E-01 | -6.073 |
| EGR3 | -0.142 | 5.227 | -0.912 | 3.63E-01 | 5.21E-01 | -6.074 |
| LOXL1 | 0.124 | 8.614 | 0.91 | 3.63E-01 | 5.21E-01 | -6.075 |
| CDS2 | 0.072 | 8.632 | 0.91 | 3.63E-01 | 5.21E-01 | -6.075 |
| STAT5A | -0.121 | 8.315 | -0.91 | 3.64E-01 | 5.22E-01 | -6.075 |
| SNX24 | -0.069 | 8.623 | -0.909 | 3.64E-01 | 5.23E-01 | -6.077 |
| STAT3 | 0.082 | 8.819 | 0.907 | 3.65E-01 | 5.23E-01 | -6.077 |
| HSPB8 | -0.135 | 9.109 | -0.907 | 3.65E-01 | 5.24E-01 | -6.078 |
| TES | -0.085 | 8.381 | -0.907 | 3.65E-01 | 5.24E-01 | -6.078 |
| FTL | 0.07 | 14.315 | 0.906 | 3.65E-01 | 5.24E-01 | -6.078 |
| GRM5 | 0.091 | 5.241 | 0.906 | 3.66E-01 | 5.24E-01 | -6.079 |
| DDX28 | -0.068 | 9.54 | -0.906 | 3.66E-01 | 5.24E-01 | -6.079 |
| TRHR | 0.136 | 5.089 | 0.904 | 3.66E-01 | 5.24E-01 | -6.08 |
| CSMD2 | -0.094 | 5.211 | -0.905 | 3.66E-01 | 5.24E-01 | -6.08 |
| B3GALT1 | 0.088 | 4.938 | 0.904 | 3.66E-01 | 5.24E-01 | -6.08 |
| SOCS7 | 0.087 | 6.229 | 0.904 | 3.67E-01 | 5.25E-01 | -6.08 |
| HLA-F | 0.111 | 10.593 | 0.903 | 3.67E-01 | 5.25E-01 | -6.081 |
| CBFA2T3 | 0.097 | 7.523 | 0.902 | 3.68E-01 | 5.26E-01 | -6.082 |
| OSBPL7 | -0.095 | 6.955 | -0.903 | 3.68E-01 | 5.26E-01 | -6.082 |
| MS4A6E | -0.106 | 5.263 | -0.902 | 3.68E-01 | 5.26E-01 | -6.083 |
| ZNF496 | 0.1 | 6.436 | 0.901 | 3.68E-01 | 5.26E-01 | -6.083 |
| SPCS3 | -0.081 | 9.61 | -0.902 | 3.68E-01 | 5.26E-01 | -6.083 |
| CDC42EP2 | -0.102 | 9.014 | -0.901 | 3.69E-01 | 5.27E-01 | -6.084 |
| RPE65 | 0.12 | 4.774 | 0.9 | 3.69E-01 | 5.27E-01 | -6.084 |
| SRPR | 0.082 | 10.091 | 0.898 | 3.70E-01 | 5.28E-01 | -6.086 |
| CDH5 | -0.097 | 9.002 | -0.898 | 3.70E-01 | 5.29E-01 | -6.086 |
| FAAH | 0.106 | 6.613 | 0.897 | 3.70E-01 | 5.29E-01 | -6.087 |
| NFIX | 0.112 | 7.79 | 0.896 | 3.71E-01 | 5.30E-01 | -6.088 |
| BLK | 0.113 | 6.007 | 0.896 | 3.71E-01 | 5.30E-01 | -6.088 |
| CPXCR1 | -0.09 | 4.884 | -0.896 | 3.71E-01 | 5.30E-01 | -6.088 |
| GSG1 | -0.097 | 5.384 | -0.896 | 3.71E-01 | 5.30E-01 | -6.088 |
| ARSA | -0.116 | 8.548 | -0.896 | 3.72E-01 | 5.30E-01 | -6.089 |
| ALG9 | 0.078 | 8.49 | 0.895 | 3.72E-01 | 5.30E-01 | -6.089 |
| GPR161 | -0.089 | 6.463 | -0.895 | 3.72E-01 | 5.30E-01 | -6.089 |
| FBXO39 | 0.101 | 4.788 | 0.893 | 3.72E-01 | 5.31E-01 | -6.09 |
| FOXF2 | 0.096 | 7.39 | 0.893 | 3.72E-01 | 5.31E-01 | -6.09 |
| FBP2 | 0.087 | 4.543 | 0.893 | 3.72E-01 | 5.31E-01 | -6.09 |
| NSF | 0.098 | 7.79 | 0.893 | 3.73E-01 | 5.31E-01 | -6.09 |
| MLNR | -0.095 | 4.704 | -0.894 | 3.73E-01 | 5.31E-01 | -6.09 |
| LAPTM4B | -0.084 | 9.537 | -0.892 | 3.73E-01 | 5.32E-01 | -6.091 |
| BLM | 0.094 | 7.927 | 0.891 | 3.73E-01 | 5.32E-01 | -6.092 |
| CYP4F11 | 0.081 | 4.739 | 0.891 | 3.74E-01 | 5.32E-01 | -6.092 |
| PFDN2 | -0.08 | 9.848 | -0.891 | 3.74E-01 | 5.32E-01 | -6.093 |
| NOG | 0.196 | 7.424 | 0.89 | 3.74E-01 | 5.32E-01 | -6.093 |
| EPX | 0.094 | 4.823 | 0.89 | 3.74E-01 | 5.32E-01 | -6.093 |
| PAX7 | 0.093 | 6.034 | 0.887 | 3.76E-01 | 5.34E-01 | -6.095 |
| PHACTR1 | 0.096 | 5.842 | 0.887 | 3.76E-01 | 5.34E-01 | -6.095 |
| HOXD8 | -0.115 | 5.904 | -0.887 | 3.76E-01 | 5.35E-01 | -6.096 |
| PRRX1 | 0.158 | 6.261 | 0.886 | 3.76E-01 | 5.35E-01 | -6.096 |
| RABGGTA | -0.074 | 7.883 | -0.886 | 3.77E-01 | 5.35E-01 | -6.097 |
| MYH1 | 0.115 | 4.558 | 0.885 | 3.77E-01 | 5.35E-01 | -6.097 |
| UBR2 | -0.073 | 8.497 | -0.886 | 3.77E-01 | 5.35E-01 | -6.097 |
| CASC1 | -0.093 | 4.755 | -0.886 | 3.77E-01 | 5.35E-01 | -6.097 |
| AK5 | 0.101 | 5.561 | 0.884 | 3.77E-01 | 5.36E-01 | -6.098 |
| ZDHHC12 | 0.089 | 8.79 | 0.884 | 3.77E-01 | 5.36E-01 | -6.098 |
| SERPINB8 | 0.097 | 8.129 | 0.883 | 3.78E-01 | 5.36E-01 | -6.099 |
| DIO1 | 0.102 | 5.136 | 0.883 | 3.78E-01 | 5.36E-01 | -6.099 |
| SLC2A12 | 0.113 | 5.479 | 0.883 | 3.78E-01 | 5.36E-01 | -6.099 |
| FAM9B | -0.112 | 4.755 | -0.883 | 3.78E-01 | 5.36E-01 | -6.099 |
| SEMA5A | -0.113 | 7.179 | -0.883 | 3.78E-01 | 5.36E-01 | -6.099 |
| DNMT3B | -0.102 | 6.117 | -0.882 | 3.79E-01 | 5.37E-01 | -6.1 |
| EXOSC7 | 0.061 | 9.296 | 0.881 | 3.79E-01 | 5.37E-01 | -6.1 |
| PTN | -0.115 | 8.722 | -0.882 | 3.79E-01 | 5.37E-01 | -6.1 |
| SNX6 | -0.072 | 10.082 | -0.882 | 3.79E-01 | 5.37E-01 | -6.1 |
| KCNK12 | -0.224 | 7.095 | -0.882 | 3.79E-01 | 5.37E-01 | -6.1 |
| HOXB6 | 0.084 | 7.041 | 0.881 | 3.79E-01 | 5.37E-01 | -6.101 |
| GPR68 | 0.105 | 7.219 | 0.88 | 3.79E-01 | 5.37E-01 | -6.101 |
| CORO1B | 0.093 | 8.897 | 0.88 | 3.80E-01 | 5.37E-01 | -6.102 |
| MEIS2 | 0.078 | 6.912 | 0.88 | 3.80E-01 | 5.37E-01 | -6.102 |
| SNTG2 | -0.102 | 4.78 | -0.88 | 3.80E-01 | 5.38E-01 | -6.102 |
| CPSF3 | -0.076 | 9.32 | -0.88 | 3.80E-01 | 5.38E-01 | -6.102 |
| HLA-DPA1 | -0.154 | 8.917 | -0.88 | 3.80E-01 | 5.38E-01 | -6.102 |
| GCM1 | -0.143 | 8.522 | -0.88 | 3.80E-01 | 5.38E-01 | -6.103 |
| LTBR | -0.09 | 9.962 | -0.878 | 3.81E-01 | 5.39E-01 | -6.104 |
| ARHGEF3 | -0.086 | 8.921 | -0.877 | 3.82E-01 | 5.40E-01 | -6.105 |
| IL32 | 0.156 | 8.609 | 0.875 | 3.82E-01 | 5.40E-01 | -6.105 |
| LYAR | 0.082 | 8.902 | 0.875 | 3.82E-01 | 5.40E-01 | -6.106 |
| SYNPO2 | 0.092 | 5.594 | 0.875 | 3.82E-01 | 5.40E-01 | -6.106 |
| WASL | -0.116 | 9.199 | -0.875 | 3.83E-01 | 5.41E-01 | -6.106 |
| PRDM13 | -0.088 | 5.027 | -0.875 | 3.83E-01 | 5.41E-01 | -6.107 |
| GEMIN7 | 0.067 | 7.096 | 0.873 | 3.83E-01 | 5.41E-01 | -6.107 |
| TLR6 | 0.097 | 6.322 | 0.872 | 3.84E-01 | 5.42E-01 | -6.108 |
| IQCC | 0.094 | 6.276 | 0.872 | 3.84E-01 | 5.42E-01 | -6.108 |
| PARK2 | 0.071 | 5.626 | 0.872 | 3.84E-01 | 5.42E-01 | -6.108 |
| EDN3 | -0.101 | 4.949 | -0.872 | 3.84E-01 | 5.42E-01 | -6.109 |
| ACP1 | -0.065 | 8.873 | -0.872 | 3.84E-01 | 5.42E-01 | -6.109 |
| KIAA0101 | 0.119 | 8.306 | 0.87 | 3.85E-01 | 5.42E-01 | -6.109 |
| LILRB1 | 0.115 | 6.961 | 0.87 | 3.85E-01 | 5.43E-01 | -6.11 |
| SLC22A13 | -0.09 | 5.042 | -0.87 | 3.85E-01 | 5.43E-01 | -6.11 |
| HOXA4 | -0.119 | 5.629 | -0.87 | 3.85E-01 | 5.43E-01 | -6.11 |
| KIAA1143 | -0.078 | 7.943 | -0.87 | 3.85E-01 | 5.43E-01 | -6.11 |
| TFR2 | 0.099 | 6.372 | 0.868 | 3.86E-01 | 5.43E-01 | -6.111 |
| RNF17 | 0.113 | 4.964 | 0.867 | 3.87E-01 | 5.44E-01 | -6.113 |
| MTAP | -0.066 | 7.983 | -0.868 | 3.87E-01 | 5.44E-01 | -6.113 |
| S100A14 | 0.143 | 5.426 | 0.867 | 3.87E-01 | 5.44E-01 | -6.113 |
| MPO | -0.178 | 6.456 | -0.867 | 3.87E-01 | 5.45E-01 | -6.113 |
| AGTR1 | -0.105 | 9.101 | -0.867 | 3.87E-01 | 5.45E-01 | -6.113 |
| ITPK1 | -0.088 | 7.994 | -0.867 | 3.87E-01 | 5.45E-01 | -6.113 |
| C1QBP | -0.062 | 11.615 | -0.866 | 3.87E-01 | 5.45E-01 | -6.114 |
| XBP1 | -0.084 | 11.664 | -0.866 | 3.88E-01 | 5.45E-01 | -6.114 |
| ZBTB9 | 0.104 | 7.568 | 0.864 | 3.88E-01 | 5.46E-01 | -6.115 |
| STATH | 0.093 | 4.755 | 0.864 | 3.88E-01 | 5.46E-01 | -6.115 |
| CAPZA3 | -0.1 | 4.71 | -0.865 | 3.88E-01 | 5.46E-01 | -6.115 |
| NR2F2 | -0.094 | 9.519 | -0.864 | 3.88E-01 | 5.46E-01 | -6.115 |
| SAE1 | -0.054 | 10.814 | -0.864 | 3.89E-01 | 5.46E-01 | -6.116 |
| ADPRH | 0.126 | 7.044 | 0.863 | 3.89E-01 | 5.46E-01 | -6.116 |
| CD226 | -0.121 | 5.343 | -0.864 | 3.89E-01 | 5.46E-01 | -6.116 |
| PROK2 | -0.159 | 7.124 | -0.863 | 3.89E-01 | 5.46E-01 | -6.116 |
| HPS4 | -0.054 | 7.58 | -0.863 | 3.89E-01 | 5.46E-01 | -6.117 |
| HLA-DRA | -0.158 | 9.471 | -0.863 | 3.89E-01 | 5.47E-01 | -6.117 |
| ZNF519 | -0.104 | 5.454 | -0.863 | 3.89E-01 | 5.47E-01 | -6.117 |
| NLGN4X | 0.15 | 6.312 | 0.861 | 3.90E-01 | 5.47E-01 | -6.118 |
| CYP3A5 | 0.12 | 5.62 | 0.86 | 3.90E-01 | 5.47E-01 | -6.118 |
| KREMEN1 | 0.079 | 7.203 | 0.86 | 3.90E-01 | 5.47E-01 | -6.118 |
| CDK5R1 | 0.088 | 6.456 | 0.86 | 3.90E-01 | 5.47E-01 | -6.118 |
| SSR2 | 0.05 | 11.303 | 0.858 | 3.91E-01 | 5.49E-01 | -6.12 |
| ADAM32 | -0.081 | 4.859 | -0.859 | 3.91E-01 | 5.49E-01 | -6.12 |
| TLX3 | -0.101 | 5.254 | -0.859 | 3.92E-01 | 5.49E-01 | -6.12 |
| ESRRB | 0.109 | 5.389 | 0.857 | 3.92E-01 | 5.49E-01 | -6.121 |
| CHD4 | 0.064 | 9.867 | 0.857 | 3.92E-01 | 5.49E-01 | -6.121 |
| NF2 | 0.063 | 7.594 | 0.857 | 3.92E-01 | 5.49E-01 | -6.121 |
| ZBTB10 | -0.109 | 6.182 | -0.858 | 3.92E-01 | 5.49E-01 | -6.121 |
| XAGE3 | -0.095 | 9.104 | -0.857 | 3.93E-01 | 5.50E-01 | -6.122 |
| ANK3 | -0.117 | 7.758 | -0.856 | 3.93E-01 | 5.50E-01 | -6.122 |
| TNFRSF10D | -0.122 | 6.255 | -0.856 | 3.93E-01 | 5.50E-01 | -6.123 |
| DHCR24 | -0.123 | 8.482 | -0.855 | 3.94E-01 | 5.51E-01 | -6.124 |
| RAB15 | -0.098 | 8.434 | -0.854 | 3.94E-01 | 5.51E-01 | -6.124 |
| AGXT2 | 0.102 | 4.935 | 0.853 | 3.94E-01 | 5.51E-01 | -6.124 |
| TAF1L | -0.129 | 6.544 | -0.854 | 3.94E-01 | 5.51E-01 | -6.124 |
| CPAMD8 | -0.087 | 7.119 | -0.853 | 3.94E-01 | 5.51E-01 | -6.125 |
| ALDH5A1 | 0.105 | 6.99 | 0.851 | 3.95E-01 | 5.52E-01 | -6.125 |
| ZNF473 | -0.081 | 6.427 | -0.852 | 3.95E-01 | 5.52E-01 | -6.126 |
| PDC | -0.109 | 4.784 | -0.852 | 3.95E-01 | 5.52E-01 | -6.126 |
| PNPLA1 | 0.102 | 5.27 | 0.85 | 3.96E-01 | 5.53E-01 | -6.126 |
| TCEA2 | 0.069 | 7.545 | 0.85 | 3.96E-01 | 5.53E-01 | -6.126 |
| ARHGDIB | 0.113 | 12.03 | 0.85 | 3.96E-01 | 5.53E-01 | -6.127 |
| GUCA1C | 0.106 | 4.942 | 0.85 | 3.96E-01 | 5.53E-01 | -6.127 |
| SEC14L4 | 0.11 | 6.249 | 0.85 | 3.96E-01 | 5.53E-01 | -6.127 |
| HUNK | 0.089 | 5.17 | 0.849 | 3.96E-01 | 5.53E-01 | -6.127 |
| ZNF557 | -0.081 | 7.526 | -0.85 | 3.96E-01 | 5.53E-01 | -6.127 |
| EIF4E | -0.061 | 7.807 | -0.85 | 3.96E-01 | 5.53E-01 | -6.127 |
| SENP3 | 0.063 | 8.033 | 0.847 | 3.97E-01 | 5.54E-01 | -6.129 |
| ELSPBP1 | 0.101 | 5.426 | 0.846 | 3.98E-01 | 5.55E-01 | -6.13 |
| P2RY1 | 0.129 | 6.807 | 0.845 | 3.98E-01 | 5.55E-01 | -6.131 |
| RIPK4 | 0.08 | 6.863 | 0.845 | 3.99E-01 | 5.56E-01 | -6.131 |
| GALC | -0.113 | 7.78 | -0.845 | 3.99E-01 | 5.56E-01 | -6.132 |
| PLCB4 | 0.099 | 5.988 | 0.843 | 4.00E-01 | 5.57E-01 | -6.132 |
| EBF2 | 0.1 | 4.853 | 0.842 | 4.00E-01 | 5.57E-01 | -6.133 |
| NAB2 | 0.1 | 7.84 | 0.842 | 4.00E-01 | 5.57E-01 | -6.133 |
| EGFL6 | -0.104 | 9.285 | -0.843 | 4.00E-01 | 5.57E-01 | -6.133 |
| LY6H | 0.089 | 4.895 | 0.841 | 4.01E-01 | 5.58E-01 | -6.134 |
| GNB1 | 0.072 | 11.721 | 0.84 | 4.02E-01 | 5.59E-01 | -6.135 |
| ZNF85 | -0.112 | 7.499 | -0.841 | 4.02E-01 | 5.59E-01 | -6.135 |
| PIM3 | -0.09 | 10.693 | -0.84 | 4.02E-01 | 5.59E-01 | -6.135 |
| TAS2R40 | 0.087 | 5.141 | 0.839 | 4.02E-01 | 5.59E-01 | -6.136 |
| HAO1 | 0.089 | 5.152 | 0.838 | 4.02E-01 | 5.60E-01 | -6.136 |
| TSPYL6 | 0.101 | 4.973 | 0.838 | 4.02E-01 | 5.60E-01 | -6.136 |
| ATOH1 | -0.085 | 4.761 | -0.839 | 4.03E-01 | 5.60E-01 | -6.137 |
| SCRG1 | 0.11 | 5.624 | 0.837 | 4.03E-01 | 5.60E-01 | -6.137 |
| EIF2B2 | 0.055 | 10.269 | 0.837 | 4.03E-01 | 5.60E-01 | -6.137 |
| ZNF300 | -0.116 | 5.944 | -0.838 | 4.03E-01 | 5.60E-01 | -6.137 |
| ZNF335 | 0.071 | 8.052 | 0.836 | 4.04E-01 | 5.61E-01 | -6.138 |
| IQCD | 0.11 | 5.743 | 0.835 | 4.04E-01 | 5.61E-01 | -6.139 |
| MDGA1 | -0.107 | 5.908 | -0.836 | 4.04E-01 | 5.61E-01 | -6.139 |
| SEMG2 | 0.089 | 4.685 | 0.835 | 4.04E-01 | 5.61E-01 | -6.139 |
| ZNF584 | 0.083 | 6.648 | 0.835 | 4.04E-01 | 5.61E-01 | -6.139 |
| RABL3 | -0.073 | 7.764 | -0.835 | 4.05E-01 | 5.62E-01 | -6.14 |
| GABRR1 | -0.113 | 5.346 | -0.833 | 4.06E-01 | 5.63E-01 | -6.142 |
| MYB | -0.099 | 6.367 | -0.833 | 4.06E-01 | 5.63E-01 | -6.142 |
| ABCG5 | -0.124 | 4.918 | -0.831 | 4.07E-01 | 5.64E-01 | -6.143 |
| TULP2 | 0.101 | 5.221 | 0.83 | 4.07E-01 | 5.64E-01 | -6.143 |
| ARCN1 | 0.051 | 10.178 | 0.83 | 4.07E-01 | 5.64E-01 | -6.143 |
| HTR1A | -0.101 | 5.078 | -0.831 | 4.07E-01 | 5.64E-01 | -6.143 |
| PMPCB | -0.049 | 9.661 | -0.83 | 4.07E-01 | 5.64E-01 | -6.144 |
| SPRR4 | -0.111 | 5.381 | -0.83 | 4.07E-01 | 5.64E-01 | -6.144 |
| SPAM1 | -0.088 | 4.874 | -0.83 | 4.08E-01 | 5.65E-01 | -6.144 |
| PON1 | 0.092 | 5.016 | 0.828 | 4.08E-01 | 5.65E-01 | -6.144 |
| EDN2 | 0.092 | 5.034 | 0.827 | 4.09E-01 | 5.66E-01 | -6.145 |
| CUTC | -0.07 | 8.49 | -0.828 | 4.09E-01 | 5.66E-01 | -6.146 |
| EXOC7 | 0.047 | 9.516 | 0.826 | 4.09E-01 | 5.67E-01 | -6.146 |
| PLCL2 | -0.1 | 9.389 | -0.826 | 4.10E-01 | 5.67E-01 | -6.147 |
| RPA2 | 0.065 | 9.871 | 0.825 | 4.10E-01 | 5.67E-01 | -6.147 |
| AVPR1A | -0.106 | 4.888 | -0.825 | 4.10E-01 | 5.67E-01 | -6.148 |
| SERPINA1 | 0.1 | 7.813 | 0.824 | 4.10E-01 | 5.67E-01 | -6.148 |
| SCUBE2 | 0.131 | 6.526 | 0.823 | 4.11E-01 | 5.68E-01 | -6.148 |
| FAM9A | -0.102 | 4.723 | -0.824 | 4.11E-01 | 5.68E-01 | -6.148 |
| FOXP4 | 0.116 | 6.748 | 0.823 | 4.11E-01 | 5.68E-01 | -6.149 |
| SLC25A3 | -0.045 | 11.826 | -0.823 | 4.11E-01 | 5.68E-01 | -6.149 |
| TSPYL2 | 0.089 | 7.202 | 0.822 | 4.11E-01 | 5.68E-01 | -6.149 |
| PPARA | 0.063 | 6.179 | 0.821 | 4.12E-01 | 5.69E-01 | -6.15 |
| ZNF485 | -0.092 | 5.814 | -0.822 | 4.12E-01 | 5.69E-01 | -6.15 |
| ZNF615 | -0.075 | 7.512 | -0.822 | 4.12E-01 | 5.69E-01 | -6.15 |
| CDH24 | -0.08 | 7.231 | -0.822 | 4.12E-01 | 5.69E-01 | -6.15 |
| PARP3 | 0.098 | 6.546 | 0.821 | 4.12E-01 | 5.69E-01 | -6.151 |
| MAOA | -0.135 | 10.635 | -0.822 | 4.12E-01 | 5.69E-01 | -6.151 |
| ZNF521 | -0.099 | 7.309 | -0.821 | 4.13E-01 | 5.69E-01 | -6.151 |
| MYL4 | 0.114 | 7.207 | 0.82 | 4.13E-01 | 5.69E-01 | -6.151 |
| CREB3L2 | -0.076 | 11.137 | -0.82 | 4.13E-01 | 5.70E-01 | -6.152 |
| SALL4 | 0.121 | 5.249 | 0.819 | 4.13E-01 | 5.70E-01 | -6.152 |
| EHMT1 | -0.087 | 7.34 | -0.82 | 4.13E-01 | 5.70E-01 | -6.152 |
| FDPS | -0.062 | 10.622 | -0.819 | 4.14E-01 | 5.70E-01 | -6.153 |
| GRK1 | 0.088 | 5.257 | 0.817 | 4.14E-01 | 5.71E-01 | -6.154 |
| CYP46A1 | 0.087 | 4.941 | 0.816 | 4.15E-01 | 5.71E-01 | -6.154 |
| MUC7 | 0.108 | 5.115 | 0.816 | 4.15E-01 | 5.71E-01 | -6.154 |
| HOXC9 | 0.094 | 6.516 | 0.816 | 4.15E-01 | 5.71E-01 | -6.154 |
| LRCH1 | -0.095 | 6.693 | -0.817 | 4.15E-01 | 5.71E-01 | -6.155 |
| FIBCD1 | 0.101 | 7.913 | 0.816 | 4.15E-01 | 5.72E-01 | -6.155 |
| AK7 | -0.097 | 4.931 | -0.816 | 4.15E-01 | 5.72E-01 | -6.155 |
| ZNF101 | 0.057 | 7.593 | 0.815 | 4.15E-01 | 5.72E-01 | -6.155 |
| OVGP1 | 0.098 | 7.666 | 0.815 | 4.16E-01 | 5.72E-01 | -6.156 |
| GFRA3 | -0.109 | 6.49 | -0.815 | 4.16E-01 | 5.72E-01 | -6.156 |
| TCEAL4 | -0.087 | 10.678 | -0.815 | 4.16E-01 | 5.72E-01 | -6.156 |
| NDUFV2 | 0.06 | 11.309 | 0.814 | 4.16E-01 | 5.72E-01 | -6.156 |
| BCL2 | -0.09 | 7.828 | -0.814 | 4.16E-01 | 5.72E-01 | -6.156 |
| CDK10 | -0.061 | 7.792 | -0.814 | 4.17E-01 | 5.72E-01 | -6.157 |
| MEF2C | 0.109 | 8.177 | 0.813 | 4.17E-01 | 5.72E-01 | -6.157 |
| PPIL2 | 0.086 | 7.007 | 0.813 | 4.17E-01 | 5.72E-01 | -6.157 |
| CTNS | -0.089 | 7.222 | -0.813 | 4.17E-01 | 5.73E-01 | -6.158 |
| ACVR2B | 0.076 | 7.883 | 0.81 | 4.18E-01 | 5.74E-01 | -6.159 |
| NHLRC2 | -0.095 | 6.519 | -0.811 | 4.19E-01 | 5.75E-01 | -6.16 |
| NEDD4 | -0.075 | 7.879 | -0.81 | 4.19E-01 | 5.75E-01 | -6.16 |
| SERTAD3 | 0.082 | 8.711 | 0.809 | 4.19E-01 | 5.75E-01 | -6.16 |
| CKM | -0.119 | 5.592 | -0.81 | 4.19E-01 | 5.75E-01 | -6.16 |
| MNAT1 | -0.055 | 8.64 | -0.81 | 4.19E-01 | 5.75E-01 | -6.16 |
| MARCO | 0.127 | 6.963 | 0.808 | 4.19E-01 | 5.75E-01 | -6.16 |
| RPS7 | 0.076 | 12.271 | 0.808 | 4.19E-01 | 5.75E-01 | -6.16 |
| MYL2 | -0.13 | 5.403 | -0.808 | 4.20E-01 | 5.76E-01 | -6.162 |
| OTX2 | -0.082 | 4.721 | -0.808 | 4.20E-01 | 5.76E-01 | -6.162 |
| ITGA4 | -0.102 | 7.093 | -0.808 | 4.20E-01 | 5.76E-01 | -6.162 |
| TPX2 | 0.098 | 8.412 | 0.807 | 4.20E-01 | 5.76E-01 | -6.162 |
| LUC7L | -0.063 | 8.983 | -0.807 | 4.20E-01 | 5.76E-01 | -6.162 |
| PARP1 | 0.064 | 10.393 | 0.806 | 4.21E-01 | 5.76E-01 | -6.163 |
| EPN3 | 0.107 | 6.818 | 0.805 | 4.21E-01 | 5.77E-01 | -6.163 |
| ANKMY1 | 0.083 | 6.114 | 0.805 | 4.21E-01 | 5.77E-01 | -6.163 |
| SCN7A | 0.08 | 5.8 | 0.805 | 4.21E-01 | 5.77E-01 | -6.163 |
| TNFRSF1A | 0.126 | 9.974 | 0.805 | 4.21E-01 | 5.77E-01 | -6.163 |
| LCP2 | -0.086 | 8.311 | -0.806 | 4.21E-01 | 5.77E-01 | -6.163 |
| GSC | 0.087 | 5.013 | 0.804 | 4.22E-01 | 5.77E-01 | -6.164 |
| PWP1 | -0.056 | 10.265 | -0.805 | 4.22E-01 | 5.77E-01 | -6.164 |
| NKX6-1 | 0.091 | 4.862 | 0.803 | 4.23E-01 | 5.78E-01 | -6.165 |
| RPS16 | -0.086 | 14.175 | -0.803 | 4.23E-01 | 5.78E-01 | -6.166 |
| RAB40C | 0.12 | 7.92 | 0.802 | 4.23E-01 | 5.79E-01 | -6.166 |
| CDKN1C | 0.126 | 11.504 | 0.801 | 4.23E-01 | 5.79E-01 | -6.166 |
| VAMP5 | 0.085 | 10.585 | 0.801 | 4.23E-01 | 5.79E-01 | -6.166 |
| UTP14A | 0.092 | 7.637 | 0.801 | 4.23E-01 | 5.79E-01 | -6.166 |
| NDUFAF1 | 0.076 | 9.006 | 0.801 | 4.24E-01 | 5.79E-01 | -6.167 |
| CRY2 | 0.067 | 8.524 | 0.799 | 4.25E-01 | 5.80E-01 | -6.168 |
| TSLP | -0.083 | 4.718 | -0.8 | 4.25E-01 | 5.80E-01 | -6.168 |
| LUZP4 | 0.089 | 4.702 | 0.798 | 4.25E-01 | 5.81E-01 | -6.169 |
| NLN | -0.092 | 6.841 | -0.798 | 4.26E-01 | 5.81E-01 | -6.169 |
| FIP1L1 | -0.109 | 9.078 | -0.798 | 4.26E-01 | 5.81E-01 | -6.169 |
| GIPC3 | 0.09 | 5.484 | 0.796 | 4.26E-01 | 5.82E-01 | -6.17 |
| SLA2 | 0.088 | 5.939 | 0.796 | 4.26E-01 | 5.82E-01 | -6.17 |
| PCSK9 | 0.123 | 5.356 | 0.796 | 4.27E-01 | 5.82E-01 | -6.17 |
| PHF5A | -0.092 | 8.436 | -0.796 | 4.27E-01 | 5.82E-01 | -6.171 |
| KLF9 | -0.125 | 9.151 | -0.796 | 4.27E-01 | 5.82E-01 | -6.171 |
| INPP5D | -0.069 | 8.716 | -0.796 | 4.27E-01 | 5.82E-01 | -6.171 |
| C6 | -0.115 | 5.418 | -0.795 | 4.27E-01 | 5.82E-01 | -6.171 |
| HMGA2 | -0.076 | 5.508 | -0.795 | 4.28E-01 | 5.83E-01 | -6.172 |
| PFKL | 0.114 | 9.091 | 0.794 | 4.28E-01 | 5.83E-01 | -6.172 |
| SCARF1 | 0.085 | 7.373 | 0.794 | 4.28E-01 | 5.83E-01 | -6.172 |
| C6orf1 | 0.062 | 9.325 | 0.793 | 4.28E-01 | 5.83E-01 | -6.172 |
| MAPRE3 | -0.111 | 6.785 | -0.793 | 4.29E-01 | 5.84E-01 | -6.173 |
| PROP1 | 0.113 | 6.945 | 0.792 | 4.29E-01 | 5.84E-01 | -6.173 |
| ZNF334 | 0.089 | 5.7 | 0.792 | 4.29E-01 | 5.84E-01 | -6.173 |
| KATNAL2 | 0.078 | 5.044 | 0.791 | 4.29E-01 | 5.84E-01 | -6.174 |
| C9orf114 | 0.07 | 7.719 | 0.791 | 4.29E-01 | 5.84E-01 | -6.174 |
| ZNF142 | 0.083 | 7.796 | 0.79 | 4.30E-01 | 5.84E-01 | -6.175 |
| TDRD5 | 0.062 | 4.685 | 0.79 | 4.30E-01 | 5.85E-01 | -6.175 |
| SHC3 | 0.101 | 6.287 | 0.788 | 4.31E-01 | 5.86E-01 | -6.176 |
| ANKHD1 | -0.057 | 8.563 | -0.787 | 4.32E-01 | 5.87E-01 | -6.178 |
| ILF3 | 0.077 | 9.177 | 0.786 | 4.32E-01 | 5.87E-01 | -6.178 |
| GALNT2 | -0.083 | 8.122 | -0.787 | 4.32E-01 | 5.87E-01 | -6.178 |
| MMP17 | 0.118 | 7.351 | 0.785 | 4.33E-01 | 5.88E-01 | -6.179 |
| SNAP91 | 0.096 | 5.158 | 0.785 | 4.33E-01 | 5.88E-01 | -6.179 |
| EZH2 | 0.077 | 7.813 | 0.785 | 4.33E-01 | 5.88E-01 | -6.179 |
| TPSD1 | 0.098 | 5.473 | 0.784 | 4.33E-01 | 5.89E-01 | -6.179 |
| QDPR | -0.082 | 8.978 | -0.785 | 4.34E-01 | 5.89E-01 | -6.18 |
| UGT2B4 | 0.082 | 4.52 | 0.783 | 4.34E-01 | 5.89E-01 | -6.18 |
| ADRA1A | 0.077 | 5.307 | 0.783 | 4.34E-01 | 5.89E-01 | -6.18 |
| AAMP | 0.081 | 9.639 | 0.782 | 4.34E-01 | 5.89E-01 | -6.181 |
| POLN | -0.071 | 5.072 | -0.783 | 4.35E-01 | 5.90E-01 | -6.181 |
| EEF1A2 | 0.103 | 5.63 | 0.782 | 4.35E-01 | 5.90E-01 | -6.181 |
| FBXL8 | 0.072 | 6.787 | 0.781 | 4.35E-01 | 5.90E-01 | -6.182 |
| SLC2A5 | 0.099 | 6.725 | 0.78 | 4.36E-01 | 5.91E-01 | -6.182 |
| KCTD2 | 0.071 | 7.78 | 0.779 | 4.36E-01 | 5.91E-01 | -6.183 |
| SYT4 | -0.069 | 4.593 | -0.78 | 4.36E-01 | 5.91E-01 | -6.183 |
| MSH4 | 0.102 | 4.939 | 0.779 | 4.36E-01 | 5.91E-01 | -6.183 |
| GOLGA3 | 0.063 | 9.165 | 0.778 | 4.37E-01 | 5.92E-01 | -6.184 |
| MASP1 | 0.064 | 5.383 | 0.778 | 4.37E-01 | 5.92E-01 | -6.184 |
| ZDHHC14 | 0.077 | 8.072 | 0.777 | 4.37E-01 | 5.92E-01 | -6.185 |
| IL5RA | -0.105 | 5.31 | -0.778 | 4.37E-01 | 5.92E-01 | -6.185 |
| TRIM6 | -0.096 | 7.318 | -0.777 | 4.38E-01 | 5.93E-01 | -6.185 |
| GIT2 | -0.057 | 8.266 | -0.777 | 4.38E-01 | 5.93E-01 | -6.185 |
| MS4A4A | 0.099 | 8.428 | 0.776 | 4.38E-01 | 5.93E-01 | -6.186 |
| APOL6 | 0.102 | 6.719 | 0.775 | 4.38E-01 | 5.93E-01 | -6.186 |
| CD300A | 0.089 | 7.41 | 0.774 | 4.39E-01 | 5.94E-01 | -6.187 |
| NRIP2 | 0.098 | 5.776 | 0.774 | 4.40E-01 | 5.94E-01 | -6.187 |
| COMMD3 | -0.053 | 10.841 | -0.774 | 4.40E-01 | 5.95E-01 | -6.188 |
| DDB2 | 0.058 | 8.392 | 0.773 | 4.40E-01 | 5.95E-01 | -6.188 |
| CNNM1 | 0.089 | 5.274 | 0.772 | 4.40E-01 | 5.95E-01 | -6.188 |
| NUDT9 | -0.065 | 8.879 | -0.771 | 4.42E-01 | 5.97E-01 | -6.19 |
| ZBTB17 | 0.065 | 8.659 | 0.77 | 4.42E-01 | 5.97E-01 | -6.19 |
| CTSK | -0.105 | 9.13 | -0.771 | 4.42E-01 | 5.97E-01 | -6.19 |
| ZNF586 | 0.063 | 7.588 | 0.77 | 4.42E-01 | 5.97E-01 | -6.19 |
| CLEC12A | 0.102 | 6.289 | 0.769 | 4.42E-01 | 5.97E-01 | -6.191 |
| GCGR | 0.089 | 4.907 | 0.769 | 4.42E-01 | 5.97E-01 | -6.191 |
| SLC24A2 | 0.072 | 5.012 | 0.769 | 4.42E-01 | 5.97E-01 | -6.191 |
| HOOK3 | -0.094 | 7.815 | -0.769 | 4.43E-01 | 5.97E-01 | -6.191 |
| GBA2 | 0.062 | 7.841 | 0.768 | 4.43E-01 | 5.97E-01 | -6.192 |
| PELI2 | -0.078 | 7.816 | -0.769 | 4.43E-01 | 5.97E-01 | -6.192 |
| PRODH2 | -0.101 | 5.054 | -0.769 | 4.43E-01 | 5.97E-01 | -6.192 |
| CYP3A4 | 0.103 | 4.898 | 0.767 | 4.44E-01 | 5.98E-01 | -6.193 |
| CHCHD1 | -0.07 | 10.004 | -0.767 | 4.44E-01 | 5.98E-01 | -6.193 |
| THRSP | 0.077 | 4.808 | 0.766 | 4.44E-01 | 5.98E-01 | -6.193 |
| ADH6 | -0.069 | 4.716 | -0.767 | 4.44E-01 | 5.98E-01 | -6.193 |
| G0S2 | -0.178 | 8.052 | -0.767 | 4.44E-01 | 5.98E-01 | -6.193 |
| SALL2 | 0.1 | 7.18 | 0.765 | 4.45E-01 | 5.99E-01 | -6.194 |
| KIAA0513 | 0.083 | 8.11 | 0.764 | 4.45E-01 | 6.00E-01 | -6.195 |
| SLC6A1 | 0.088 | 5.159 | 0.763 | 4.46E-01 | 6.01E-01 | -6.196 |
| SNX12 | -0.061 | 8.063 | -0.763 | 4.46E-01 | 6.01E-01 | -6.196 |
| EXT2 | 0.05 | 9.911 | 0.762 | 4.47E-01 | 6.01E-01 | -6.196 |
| SLC39A1 | 0.061 | 10.565 | 0.761 | 4.47E-01 | 6.02E-01 | -6.197 |
| CACNA2D4 | 0.078 | 6.243 | 0.761 | 4.47E-01 | 6.02E-01 | -6.197 |
| FCHSD2 | 0.053 | 8.515 | 0.76 | 4.47E-01 | 6.02E-01 | -6.197 |
| HS3ST4 | 0.067 | 5.294 | 0.759 | 4.48E-01 | 6.02E-01 | -6.198 |
| P2RX4 | 0.084 | 9.342 | 0.759 | 4.48E-01 | 6.02E-01 | -6.198 |
| COX5B | 0.06 | 12.364 | 0.759 | 4.48E-01 | 6.03E-01 | -6.198 |
| KRTAP3-2 | -0.108 | 4.9 | -0.76 | 4.48E-01 | 6.03E-01 | -6.198 |
| ADCK2 | 0.057 | 8.73 | 0.759 | 4.48E-01 | 6.03E-01 | -6.199 |
| ITGA3 | 0.087 | 7.87 | 0.758 | 4.49E-01 | 6.03E-01 | -6.199 |
| GNLY | 0.172 | 6.878 | 0.758 | 4.49E-01 | 6.03E-01 | -6.199 |
| BCL11A | -0.062 | 6.245 | -0.759 | 4.49E-01 | 6.03E-01 | -6.199 |
| ADPRHL2 | 0.053 | 9.123 | 0.757 | 4.49E-01 | 6.03E-01 | -6.2 |
| ARHGAP25 | 0.09 | 7.804 | 0.757 | 4.49E-01 | 6.03E-01 | -6.2 |
| HIC2 | 0.065 | 8.596 | 0.757 | 4.50E-01 | 6.04E-01 | -6.2 |
| IL1RAPL1 | 0.093 | 4.889 | 0.756 | 4.50E-01 | 6.04E-01 | -6.201 |
| ASRGL1 | 0.092 | 7.421 | 0.755 | 4.50E-01 | 6.04E-01 | -6.201 |
| SPATA13 | -0.105 | 7.414 | -0.756 | 4.51E-01 | 6.05E-01 | -6.202 |
| MBTPS1 | 0.067 | 9.861 | 0.755 | 4.51E-01 | 6.05E-01 | -6.202 |
| CLEC1B | 0.114 | 6.165 | 0.754 | 4.51E-01 | 6.05E-01 | -6.202 |
| API5 | -0.061 | 9.101 | -0.755 | 4.51E-01 | 6.05E-01 | -6.202 |
| SEC61A1 | 0.068 | 10.551 | 0.754 | 4.51E-01 | 6.05E-01 | -6.202 |
| LYPLAL1 | -0.056 | 8.808 | -0.755 | 4.51E-01 | 6.05E-01 | -6.202 |
| SLC2A11 | -0.1 | 7.584 | -0.754 | 4.52E-01 | 6.05E-01 | -6.203 |
| RRM2 | -0.109 | 7.9 | -0.754 | 4.52E-01 | 6.05E-01 | -6.203 |
| SVIL | 0.081 | 7.819 | 0.753 | 4.52E-01 | 6.05E-01 | -6.203 |
| PGM2L1 | -0.088 | 6.844 | -0.753 | 4.52E-01 | 6.05E-01 | -6.203 |
| SPATA9 | -0.08 | 6.037 | -0.753 | 4.52E-01 | 6.05E-01 | -6.203 |
| SOX6 | 0.099 | 5.665 | 0.752 | 4.52E-01 | 6.05E-01 | -6.203 |
| RAB31 | -0.068 | 11.28 | -0.753 | 4.52E-01 | 6.05E-01 | -6.204 |
| HPCAL4 | 0.081 | 5.355 | 0.752 | 4.52E-01 | 6.06E-01 | -6.204 |
| DTX1 | -0.122 | 5.739 | -0.752 | 4.53E-01 | 6.06E-01 | -6.204 |
| MAN1C1 | 0.108 | 9.855 | 0.75 | 4.53E-01 | 6.06E-01 | -6.205 |
| SULF1 | 0.128 | 6.914 | 0.75 | 4.53E-01 | 6.06E-01 | -6.205 |
| SLPI | 0.158 | 7.436 | 0.75 | 4.54E-01 | 6.07E-01 | -6.205 |
| PHF21A | -0.059 | 8.598 | -0.75 | 4.54E-01 | 6.07E-01 | -6.206 |
| KCTD4 | -0.148 | 5.492 | -0.75 | 4.54E-01 | 6.07E-01 | -6.206 |
| C6orf48 | 0.061 | 11.086 | 0.749 | 4.54E-01 | 6.07E-01 | -6.206 |
| SLC22A14 | 0.09 | 6.694 | 0.748 | 4.55E-01 | 6.08E-01 | -6.206 |
| CPA5 | 0.093 | 5.894 | 0.747 | 4.56E-01 | 6.09E-01 | -6.208 |
| EIF4EBP2 | -0.049 | 10.368 | -0.746 | 4.57E-01 | 6.10E-01 | -6.209 |
| KCNA10 | -0.097 | 4.894 | -0.745 | 4.57E-01 | 6.10E-01 | -6.209 |
| SLC16A2 | -0.094 | 7.832 | -0.745 | 4.57E-01 | 6.11E-01 | -6.21 |
| CCL20 | -0.135 | 5.369 | -0.744 | 4.57E-01 | 6.11E-01 | -6.21 |
| MFAP5 | 0.115 | 9.085 | 0.743 | 4.58E-01 | 6.11E-01 | -6.21 |
| TFAP2C | 0.109 | 7.716 | 0.743 | 4.58E-01 | 6.11E-01 | -6.21 |
| TBL1Y | 0.098 | 7.159 | 0.742 | 4.59E-01 | 6.12E-01 | -6.211 |
| ABCA8 | -0.101 | 6.393 | -0.74 | 4.60E-01 | 6.14E-01 | -6.213 |
| SRCAP | 0.079 | 7.114 | 0.739 | 4.60E-01 | 6.14E-01 | -6.213 |
| ABTB2 | -0.109 | 7.703 | -0.74 | 4.60E-01 | 6.14E-01 | -6.213 |
| CST3 | -0.079 | 11.084 | -0.739 | 4.61E-01 | 6.14E-01 | -6.214 |
| CBLB | 0.103 | 7.941 | 0.738 | 4.61E-01 | 6.14E-01 | -6.214 |
| PDCD1LG2 | -0.117 | 5.789 | -0.738 | 4.61E-01 | 6.15E-01 | -6.214 |
| PELP1 | 0.059 | 9.561 | 0.737 | 4.61E-01 | 6.15E-01 | -6.214 |
| PKIG | 0.059 | 9.25 | 0.737 | 4.61E-01 | 6.15E-01 | -6.214 |
| DERL1 | -0.061 | 9.135 | -0.738 | 4.61E-01 | 6.15E-01 | -6.215 |
| E2F5 | -0.09 | 7.163 | -0.738 | 4.62E-01 | 6.15E-01 | -6.215 |
| FUNDC2 | 0.064 | 7.899 | 0.736 | 4.62E-01 | 6.15E-01 | -6.215 |
| IL2RA | 0.103 | 6.265 | 0.736 | 4.62E-01 | 6.15E-01 | -6.215 |
| SYT3 | 0.097 | 6.402 | 0.735 | 4.63E-01 | 6.16E-01 | -6.216 |
| CERK | 0.086 | 8.058 | 0.735 | 4.63E-01 | 6.16E-01 | -6.216 |
| ICOS | -0.087 | 5.966 | -0.736 | 4.63E-01 | 6.16E-01 | -6.216 |
| HMGB2 | -0.106 | 9.488 | -0.736 | 4.63E-01 | 6.16E-01 | -6.216 |
| TNFSF11 | 0.065 | 4.792 | 0.734 | 4.63E-01 | 6.16E-01 | -6.216 |
| ZBED2 | 0.155 | 5.665 | 0.734 | 4.63E-01 | 6.16E-01 | -6.217 |
| CPN2 | 0.094 | 5.23 | 0.732 | 4.64E-01 | 6.17E-01 | -6.218 |
| GRM8 | 0.089 | 5.032 | 0.732 | 4.65E-01 | 6.17E-01 | -6.218 |
| FOXP2 | 0.05 | 4.82 | 0.731 | 4.65E-01 | 6.18E-01 | -6.219 |
| TAS2R50 | -0.098 | 5.094 | -0.732 | 4.65E-01 | 6.18E-01 | -6.219 |
| SOX2 | -0.08 | 5.005 | -0.732 | 4.65E-01 | 6.18E-01 | -6.219 |
| DOCK1 | -0.072 | 8.17 | -0.732 | 4.65E-01 | 6.18E-01 | -6.219 |
| DGKH | 0.083 | 5.581 | 0.73 | 4.65E-01 | 6.18E-01 | -6.219 |
| ITGB7 | 0.086 | 6.953 | 0.73 | 4.66E-01 | 6.18E-01 | -6.22 |
| MMP9 | -0.126 | 8.352 | -0.731 | 4.66E-01 | 6.18E-01 | -6.22 |
| SLC3A1 | -0.084 | 5.069 | -0.73 | 4.66E-01 | 6.19E-01 | -6.22 |
| NOTCH2NL | -0.09 | 8.658 | -0.73 | 4.66E-01 | 6.19E-01 | -6.22 |
| DDAH1 | -0.101 | 7.654 | -0.729 | 4.67E-01 | 6.19E-01 | -6.221 |
| UBE2E2 | 0.081 | 7.922 | 0.728 | 4.67E-01 | 6.19E-01 | -6.221 |
| BMP1 | 0.106 | 7.826 | 0.728 | 4.67E-01 | 6.19E-01 | -6.221 |
| BCR | 0.107 | 7.861 | 0.727 | 4.67E-01 | 6.20E-01 | -6.222 |
| PEX7 | 0.051 | 7.969 | 0.727 | 4.67E-01 | 6.20E-01 | -6.222 |
| REN | 0.128 | 6.308 | 0.726 | 4.68E-01 | 6.21E-01 | -6.223 |
| ATP6V1G3 | 0.089 | 5.02 | 0.726 | 4.68E-01 | 6.21E-01 | -6.223 |
| SIX6 | -0.085 | 4.865 | -0.726 | 4.68E-01 | 6.21E-01 | -6.223 |
| TBX2 | 0.11 | 8.668 | 0.725 | 4.69E-01 | 6.21E-01 | -6.223 |
| EPM2AIP1 | 0.069 | 7.923 | 0.725 | 4.69E-01 | 6.21E-01 | -6.223 |
| TRIM3 | 0.071 | 6.173 | 0.724 | 4.69E-01 | 6.21E-01 | -6.223 |
| CYTL1 | -0.119 | 7.57 | -0.725 | 4.69E-01 | 6.21E-01 | -6.224 |
| CA1 | 0.131 | 7.839 | 0.724 | 4.69E-01 | 6.21E-01 | -6.224 |
| TFAP2E | 0.076 | 5.968 | 0.723 | 4.70E-01 | 6.22E-01 | -6.224 |
| SLC17A3 | 0.082 | 5.107 | 0.723 | 4.70E-01 | 6.22E-01 | -6.225 |
| PITPNM2 | -0.072 | 6.11 | -0.724 | 4.70E-01 | 6.22E-01 | -6.225 |
| CLDN18 | -0.08 | 4.918 | -0.724 | 4.70E-01 | 6.22E-01 | -6.225 |
| C16orf46 | -0.072 | 5.402 | -0.723 | 4.70E-01 | 6.22E-01 | -6.225 |
| CCL13 | 0.089 | 6.557 | 0.722 | 4.70E-01 | 6.22E-01 | -6.225 |
| HMGB3 | 0.089 | 9.787 | 0.722 | 4.70E-01 | 6.22E-01 | -6.225 |
| SPHK1 | -0.09 | 7.832 | -0.723 | 4.71E-01 | 6.22E-01 | -6.225 |
| FAM19A4 | -0.117 | 4.909 | -0.722 | 4.71E-01 | 6.22E-01 | -6.226 |
| WASF1 | 0.075 | 7.348 | 0.721 | 4.71E-01 | 6.22E-01 | -6.226 |
| HTN1 | -0.079 | 4.717 | -0.722 | 4.71E-01 | 6.23E-01 | -6.226 |
| DOK1 | -0.063 | 7.907 | -0.721 | 4.72E-01 | 6.23E-01 | -6.227 |
| SLC25A20 | -0.067 | 8.136 | -0.721 | 4.72E-01 | 6.23E-01 | -6.227 |
| HIST1H1D | 0.11 | 7.381 | 0.719 | 4.72E-01 | 6.23E-01 | -6.227 |
| FHL2 | 0.087 | 10.122 | 0.719 | 4.72E-01 | 6.23E-01 | -6.227 |
| KLK3 | -0.093 | 6.21 | -0.72 | 4.72E-01 | 6.23E-01 | -6.227 |
| TMC7 | 0.078 | 6.333 | 0.719 | 4.73E-01 | 6.23E-01 | -6.228 |
| TAC3 | 0.226 | 8.419 | 0.719 | 4.73E-01 | 6.23E-01 | -6.228 |
| CNN3 | -0.075 | 10.769 | -0.719 | 4.73E-01 | 6.23E-01 | -6.228 |
| IQCE | -0.06 | 7.102 | -0.719 | 4.73E-01 | 6.24E-01 | -6.228 |
| GAS2L2 | -0.075 | 6.073 | -0.719 | 4.73E-01 | 6.24E-01 | -6.228 |
| LETM2 | 0.079 | 6.084 | 0.717 | 4.74E-01 | 6.24E-01 | -6.229 |
| MTHFD1 | -0.061 | 9.152 | -0.718 | 4.74E-01 | 6.24E-01 | -6.229 |
| GOLGA2 | -0.08 | 8.143 | -0.717 | 4.74E-01 | 6.25E-01 | -6.229 |
| CREG1 | 0.079 | 11.146 | 0.716 | 4.74E-01 | 6.25E-01 | -6.23 |
| ZBTB16 | 0.131 | 7.838 | 0.716 | 4.74E-01 | 6.25E-01 | -6.23 |
| CNTN6 | -0.104 | 5.784 | -0.716 | 4.75E-01 | 6.25E-01 | -6.23 |
| ILF2 | 0.073 | 10.433 | 0.715 | 4.75E-01 | 6.25E-01 | -6.23 |
| BACE2 | -0.086 | 9.159 | -0.716 | 4.75E-01 | 6.25E-01 | -6.23 |
| ID2 | -0.09 | 10.137 | -0.715 | 4.76E-01 | 6.26E-01 | -6.231 |
| GNPAT | -0.053 | 9.557 | -0.714 | 4.76E-01 | 6.26E-01 | -6.231 |
| CGREF1 | 0.086 | 5.457 | 0.713 | 4.76E-01 | 6.27E-01 | -6.232 |
| DEFA4 | -0.167 | 6.944 | -0.713 | 4.77E-01 | 6.27E-01 | -6.233 |
| WBP11 | -0.054 | 8.689 | -0.713 | 4.77E-01 | 6.27E-01 | -6.233 |
| MAML2 | -0.08 | 6.824 | -0.712 | 4.77E-01 | 6.27E-01 | -6.233 |
| MPPE1 | 0.053 | 8.289 | 0.711 | 4.77E-01 | 6.27E-01 | -6.233 |
| PLCG2 | -0.075 | 8.458 | -0.712 | 4.77E-01 | 6.27E-01 | -6.233 |
| RBAK | -0.091 | 6.236 | -0.712 | 4.77E-01 | 6.27E-01 | -6.233 |
| C20orf85 | 0.074 | 4.96 | 0.711 | 4.78E-01 | 6.27E-01 | -6.233 |
| HOXC11 | -0.069 | 4.877 | -0.711 | 4.78E-01 | 6.27E-01 | -6.233 |
| PLA2G2F | 0.068 | 5.461 | 0.71 | 4.78E-01 | 6.28E-01 | -6.234 |
| NUDT12 | -0.093 | 6.169 | -0.71 | 4.78E-01 | 6.28E-01 | -6.234 |
| ESM1 | 0.089 | 5.342 | 0.709 | 4.78E-01 | 6.28E-01 | -6.234 |
| FMNL3 | -0.073 | 6.033 | -0.71 | 4.79E-01 | 6.28E-01 | -6.235 |
| MYH3 | -0.086 | 6.254 | -0.71 | 4.79E-01 | 6.28E-01 | -6.235 |
| DMBX1 | 0.091 | 5.082 | 0.709 | 4.79E-01 | 6.28E-01 | -6.235 |
| PHOX2B | 0.076 | 4.891 | 0.708 | 4.79E-01 | 6.28E-01 | -6.235 |
| MCM2 | 0.068 | 9.716 | 0.705 | 4.81E-01 | 6.31E-01 | -6.237 |
| DLL1 | -0.099 | 7.266 | -0.705 | 4.82E-01 | 6.32E-01 | -6.238 |
| YTHDF2 | -0.039 | 10.705 | -0.704 | 4.82E-01 | 6.32E-01 | -6.238 |
| TUBAL3 | -0.098 | 5.819 | -0.704 | 4.82E-01 | 6.32E-01 | -6.238 |
| TREM2 | 0.092 | 6.244 | 0.703 | 4.82E-01 | 6.32E-01 | -6.238 |
| EIF5A | 0.082 | 10.632 | 0.703 | 4.82E-01 | 6.32E-01 | -6.239 |
| DYRK2 | -0.067 | 8.131 | -0.704 | 4.82E-01 | 6.32E-01 | -6.239 |
| FOXM1 | 0.088 | 7.841 | 0.702 | 4.83E-01 | 6.33E-01 | -6.239 |
| C6orf47 | 0.061 | 8.255 | 0.701 | 4.83E-01 | 6.33E-01 | -6.24 |
| KCNIP4 | 0.08 | 5.281 | 0.701 | 4.83E-01 | 6.33E-01 | -6.24 |
| RARRES3 | 0.095 | 9.708 | 0.701 | 4.84E-01 | 6.33E-01 | -6.24 |
| TBX21 | 0.098 | 6.521 | 0.7 | 4.84E-01 | 6.34E-01 | -6.241 |
| JMY | -0.076 | 7.08 | -0.701 | 4.84E-01 | 6.34E-01 | -6.241 |
| SLC24A4 | -0.073 | 6.244 | -0.7 | 4.85E-01 | 6.34E-01 | -6.241 |
| PRPS1 | -0.072 | 8.734 | -0.7 | 4.85E-01 | 6.34E-01 | -6.241 |
| COL24A1 | -0.102 | 5.126 | -0.699 | 4.85E-01 | 6.34E-01 | -6.242 |
| OTOP2 | 0.085 | 5.839 | 0.698 | 4.85E-01 | 6.34E-01 | -6.242 |
| MEP1A | -0.11 | 5.271 | -0.699 | 4.85E-01 | 6.34E-01 | -6.242 |
| SORT1 | 0.069 | 9.437 | 0.698 | 4.85E-01 | 6.34E-01 | -6.242 |
| FOSL1 | -0.081 | 7.018 | -0.699 | 4.85E-01 | 6.34E-01 | -6.242 |
| RASGRP3 | 0.08 | 6.973 | 0.697 | 4.86E-01 | 6.35E-01 | -6.243 |
| MAP3K15 | -0.076 | 4.933 | -0.698 | 4.86E-01 | 6.35E-01 | -6.243 |
| MEGF11 | -0.085 | 5.259 | -0.697 | 4.87E-01 | 6.35E-01 | -6.243 |
| TNIP3 | -0.09 | 4.834 | -0.697 | 4.87E-01 | 6.35E-01 | -6.243 |
| HS3ST1 | -0.103 | 6.32 | -0.697 | 4.87E-01 | 6.36E-01 | -6.244 |
| COPS7B | -0.067 | 7.93 | -0.696 | 4.87E-01 | 6.36E-01 | -6.244 |
| WWP2 | 0.062 | 8.379 | 0.694 | 4.88E-01 | 6.36E-01 | -6.244 |
| ZCCHC13 | 0.085 | 4.766 | 0.694 | 4.88E-01 | 6.36E-01 | -6.245 |
| SYNE2 | -0.077 | 7.377 | -0.695 | 4.88E-01 | 6.36E-01 | -6.245 |
| PTPN9 | -0.067 | 6.974 | -0.695 | 4.88E-01 | 6.37E-01 | -6.245 |
| RASAL1 | 0.097 | 6.303 | 0.692 | 4.89E-01 | 6.38E-01 | -6.246 |
| APOF | -0.058 | 4.681 | -0.693 | 4.89E-01 | 6.38E-01 | -6.246 |
| ST6GALNAC2 | 0.074 | 8.062 | 0.691 | 4.89E-01 | 6.38E-01 | -6.246 |
| C2orf15 | -0.101 | 5.595 | -0.692 | 4.90E-01 | 6.39E-01 | -6.247 |
| COLEC10 | 0.084 | 5.794 | 0.69 | 4.90E-01 | 6.39E-01 | -6.247 |
| C20orf141 | 0.096 | 9.081 | 0.69 | 4.90E-01 | 6.39E-01 | -6.247 |
| ADAM30 | 0.075 | 5.222 | 0.69 | 4.91E-01 | 6.39E-01 | -6.248 |
| SUSD3 | -0.093 | 8.384 | -0.69 | 4.91E-01 | 6.39E-01 | -6.248 |
| PLAGL1 | -0.085 | 9.483 | -0.689 | 4.92E-01 | 6.40E-01 | -6.249 |
| RPS6KL1 | 0.06 | 5.838 | 0.687 | 4.92E-01 | 6.41E-01 | -6.249 |
| PCBP3 | -0.104 | 5.267 | -0.688 | 4.92E-01 | 6.41E-01 | -6.249 |
| SNRPB | -0.046 | 11.233 | -0.686 | 4.93E-01 | 6.42E-01 | -6.251 |
| AQP5 | 0.064 | 6.262 | 0.683 | 4.94E-01 | 6.43E-01 | -6.252 |
| TPR | -0.066 | 8.922 | -0.684 | 4.95E-01 | 6.43E-01 | -6.252 |
| GOLT1A | 0.083 | 5.981 | 0.683 | 4.95E-01 | 6.44E-01 | -6.252 |
| FAM50B | -0.084 | 7.83 | -0.684 | 4.95E-01 | 6.44E-01 | -6.252 |
| CD151 | 0.084 | 10.184 | 0.682 | 4.95E-01 | 6.44E-01 | -6.253 |
| PCSK6 | -0.092 | 6.636 | -0.682 | 4.96E-01 | 6.44E-01 | -6.253 |
| CDCA4 | -0.077 | 7.513 | -0.682 | 4.96E-01 | 6.44E-01 | -6.253 |
| YPEL1 | 0.059 | 6.951 | 0.68 | 4.97E-01 | 6.45E-01 | -6.254 |
| AGRP | -0.093 | 5.639 | -0.681 | 4.97E-01 | 6.46E-01 | -6.254 |
| LIMK1 | 0.067 | 8.07 | 0.679 | 4.97E-01 | 6.46E-01 | -6.255 |
| RHCG | -0.077 | 5.545 | -0.68 | 4.97E-01 | 6.46E-01 | -6.255 |
| GPHA2 | 0.082 | 5.756 | 0.679 | 4.97E-01 | 6.46E-01 | -6.255 |
| ZNF548 | 0.056 | 7.234 | 0.678 | 4.98E-01 | 6.46E-01 | -6.255 |
| PPAP2C | 0.128 | 7.537 | 0.678 | 4.98E-01 | 6.46E-01 | -6.256 |
| PLEKHH2 | -0.1 | 5.826 | -0.678 | 4.99E-01 | 6.47E-01 | -6.256 |
| SI | -0.062 | 4.574 | -0.678 | 4.99E-01 | 6.47E-01 | -6.256 |
| MACF1 | 0.075 | 8.039 | 0.676 | 4.99E-01 | 6.48E-01 | -6.257 |
| CNTFR | 0.082 | 5.636 | 0.675 | 5.00E-01 | 6.48E-01 | -6.257 |
| ARPC4 | 0.055 | 8.711 | 0.675 | 5.00E-01 | 6.48E-01 | -6.258 |
| PTCHD1 | 0.083 | 4.815 | 0.675 | 5.00E-01 | 6.48E-01 | -6.258 |
| SLC22A7 | 0.066 | 5.279 | 0.674 | 5.00E-01 | 6.48E-01 | -6.258 |
| OPRD1 | 0.092 | 5.857 | 0.674 | 5.01E-01 | 6.49E-01 | -6.258 |
| REG4 | 0.058 | 5.14 | 0.674 | 5.01E-01 | 6.49E-01 | -6.258 |
| TBCD | 0.06 | 9.017 | 0.673 | 5.01E-01 | 6.49E-01 | -6.259 |
| GTF2IRD1 | 0.063 | 7.05 | 0.672 | 5.02E-01 | 6.50E-01 | -6.259 |
| TNFSF4 | 0.098 | 6.801 | 0.671 | 5.03E-01 | 6.51E-01 | -6.26 |
| B3GALNT2 | -0.06 | 7.328 | -0.672 | 5.03E-01 | 6.51E-01 | -6.26 |
| CCL16 | 0.082 | 5.399 | 0.67 | 5.03E-01 | 6.51E-01 | -6.26 |
| PJA1 | -0.06 | 9.207 | -0.671 | 5.03E-01 | 6.51E-01 | -6.261 |
| CCT2 | 0.061 | 11.594 | 0.67 | 5.03E-01 | 6.51E-01 | -6.261 |
| RASSF5 | -0.052 | 6.791 | -0.67 | 5.03E-01 | 6.51E-01 | -6.261 |
| PCBP2 | -0.057 | 10.859 | -0.67 | 5.04E-01 | 6.51E-01 | -6.261 |
| RASGRF1 | 0.081 | 5.166 | 0.668 | 5.04E-01 | 6.52E-01 | -6.262 |
| SLC17A4 | 0.072 | 5.291 | 0.668 | 5.04E-01 | 6.52E-01 | -6.262 |
| CORO6 | 0.13 | 9.297 | 0.668 | 5.04E-01 | 6.52E-01 | -6.262 |
| EMCN | -0.089 | 5.522 | -0.668 | 5.05E-01 | 6.52E-01 | -6.262 |
| LRRTM2 | 0.07 | 5.21 | 0.665 | 5.06E-01 | 6.54E-01 | -6.264 |
| CCNE1 | -0.08 | 8.848 | -0.666 | 5.06E-01 | 6.54E-01 | -6.264 |
| FLRT3 | 0.14 | 5.91 | 0.665 | 5.06E-01 | 6.54E-01 | -6.264 |
| OR7D2 | 0.091 | 4.78 | 0.665 | 5.06E-01 | 6.54E-01 | -6.264 |
| TACC2 | -0.064 | 8.536 | -0.663 | 5.08E-01 | 6.56E-01 | -6.266 |
| RPA4 | 0.104 | 7.358 | 0.662 | 5.08E-01 | 6.56E-01 | -6.266 |
| SMPD3 | 0.077 | 6.181 | 0.662 | 5.08E-01 | 6.56E-01 | -6.266 |
| CSF3R | -0.114 | 10.137 | -0.663 | 5.08E-01 | 6.56E-01 | -6.266 |
| CCDC3 | 0.131 | 8.437 | 0.662 | 5.08E-01 | 6.56E-01 | -6.266 |
| NPHP1 | 0.06 | 6.138 | 0.662 | 5.08E-01 | 6.56E-01 | -6.266 |
| HCRTR2 | 0.068 | 5.003 | 0.66 | 5.09E-01 | 6.57E-01 | -6.267 |
| CLEC3A | -0.07 | 4.722 | -0.661 | 5.09E-01 | 6.57E-01 | -6.267 |
| PIAS2 | -0.06 | 6.847 | -0.66 | 5.10E-01 | 6.58E-01 | -6.268 |
| BBS1 | 0.053 | 7.618 | 0.658 | 5.11E-01 | 6.58E-01 | -6.268 |
| GRK7 | -0.062 | 4.598 | -0.659 | 5.11E-01 | 6.58E-01 | -6.269 |
| UBE2C | -0.082 | 8.722 | -0.658 | 5.12E-01 | 6.59E-01 | -6.269 |
| TEAD1 | 0.074 | 8.58 | 0.656 | 5.12E-01 | 6.60E-01 | -6.27 |
| GAB3 | 0.061 | 6.813 | 0.656 | 5.12E-01 | 6.60E-01 | -6.27 |
| CLUL1 | -0.074 | 4.714 | -0.656 | 5.12E-01 | 6.60E-01 | -6.27 |
| GLRA3 | -0.075 | 4.803 | -0.656 | 5.13E-01 | 6.60E-01 | -6.27 |
| RPGRIP1 | -0.07 | 6.537 | -0.656 | 5.13E-01 | 6.60E-01 | -6.271 |
| ZDHHC11 | 0.072 | 6.5 | 0.655 | 5.13E-01 | 6.60E-01 | -6.271 |
| MAGED1 | -0.055 | 10.825 | -0.655 | 5.13E-01 | 6.60E-01 | -6.271 |
| HBD | 0.124 | 9.445 | 0.654 | 5.13E-01 | 6.60E-01 | -6.271 |
| HSD17B4 | -0.06 | 10.058 | -0.655 | 5.13E-01 | 6.60E-01 | -6.271 |
| TECTB | 0.068 | 4.862 | 0.654 | 5.13E-01 | 6.60E-01 | -6.271 |
| RBM5 | -0.05 | 10.906 | -0.655 | 5.13E-01 | 6.60E-01 | -6.271 |
| PTPDC1 | -0.066 | 6.498 | -0.654 | 5.14E-01 | 6.60E-01 | -6.271 |
| GPR22 | -0.079 | 4.544 | -0.652 | 5.15E-01 | 6.62E-01 | -6.273 |
| PSMC2 | -0.048 | 10.959 | -0.652 | 5.15E-01 | 6.62E-01 | -6.273 |
| B3GNT6 | 0.077 | 6.734 | 0.649 | 5.16E-01 | 6.63E-01 | -6.274 |
| LRRFIP2 | 0.044 | 8.731 | 0.649 | 5.16E-01 | 6.63E-01 | -6.274 |
| ESR2 | 0.054 | 5.132 | 0.649 | 5.16E-01 | 6.64E-01 | -6.274 |
| IFNW1 | 0.068 | 4.785 | 0.648 | 5.17E-01 | 6.64E-01 | -6.275 |
| HAPLN1 | 0.088 | 7.884 | 0.648 | 5.17E-01 | 6.64E-01 | -6.275 |
| MAPRE2 | -0.062 | 8.904 | -0.648 | 5.18E-01 | 6.65E-01 | -6.276 |
| GPC5 | -0.073 | 4.967 | -0.645 | 5.20E-01 | 6.67E-01 | -6.278 |
| DNAJC14 | 0.077 | 7.457 | 0.643 | 5.20E-01 | 6.67E-01 | -6.278 |
| FEV | 0.083 | 5.267 | 0.643 | 5.20E-01 | 6.68E-01 | -6.278 |
| FBXO44 | 0.067 | 6.458 | 0.642 | 5.21E-01 | 6.68E-01 | -6.278 |
| NAP1L2 | -0.083 | 5.446 | -0.643 | 5.21E-01 | 6.68E-01 | -6.279 |
| UST | 0.077 | 6.803 | 0.642 | 5.21E-01 | 6.69E-01 | -6.279 |
| SUV420H2 | -0.073 | 6.98 | -0.642 | 5.21E-01 | 6.69E-01 | -6.279 |
| IL12RB1 | 0.066 | 6.224 | 0.64 | 5.22E-01 | 6.70E-01 | -6.28 |
| ENAM | 0.047 | 5.32 | 0.64 | 5.22E-01 | 6.70E-01 | -6.28 |
| PKP4 | 0.068 | 7.034 | 0.639 | 5.23E-01 | 6.70E-01 | -6.28 |
| FYN | 0.059 | 8.875 | 0.639 | 5.23E-01 | 6.70E-01 | -6.281 |
| BMP2K | -0.049 | 7.633 | -0.64 | 5.23E-01 | 6.70E-01 | -6.281 |
| DNAH5 | -0.058 | 4.934 | -0.64 | 5.23E-01 | 6.70E-01 | -6.281 |
| DNAJB6 | -0.047 | 10.41 | -0.639 | 5.23E-01 | 6.70E-01 | -6.281 |
| POGZ | -0.051 | 8.362 | -0.639 | 5.24E-01 | 6.70E-01 | -6.281 |
| CLIC5 | 0.08 | 8.445 | 0.638 | 5.24E-01 | 6.70E-01 | -6.281 |
| TFCP2 | -0.054 | 8.31 | -0.639 | 5.24E-01 | 6.70E-01 | -6.281 |
| LCK | 0.076 | 6.584 | 0.636 | 5.25E-01 | 6.71E-01 | -6.282 |
| BMPER | 0.079 | 5.618 | 0.636 | 5.25E-01 | 6.72E-01 | -6.283 |
| MMP27 | 0.088 | 5.116 | 0.635 | 5.26E-01 | 6.72E-01 | -6.283 |
| CPA6 | -0.06 | 4.579 | -0.634 | 5.27E-01 | 6.74E-01 | -6.285 |
| SPTBN5 | -0.085 | 5.64 | -0.634 | 5.27E-01 | 6.74E-01 | -6.285 |
| LUM | -0.093 | 10.248 | -0.633 | 5.27E-01 | 6.74E-01 | -6.285 |
| RAB3B | 0.126 | 8.033 | 0.632 | 5.28E-01 | 6.75E-01 | -6.285 |
| CBS | -0.1 | 8.118 | -0.632 | 5.28E-01 | 6.75E-01 | -6.285 |
| MCC | -0.09 | 5.82 | -0.631 | 5.28E-01 | 6.75E-01 | -6.286 |
| RGS14 | 0.074 | 6.483 | 0.63 | 5.29E-01 | 6.76E-01 | -6.286 |
| STOML3 | -0.062 | 5.012 | -0.63 | 5.29E-01 | 6.76E-01 | -6.287 |
| SLC25A14 | 0.053 | 8.6 | 0.629 | 5.29E-01 | 6.76E-01 | -6.287 |
| ITM2A | -0.1 | 8.485 | -0.63 | 5.29E-01 | 6.76E-01 | -6.287 |
| PRSS35 | -0.085 | 5.904 | -0.63 | 5.29E-01 | 6.76E-01 | -6.287 |
| GPT2 | 0.079 | 7.642 | 0.629 | 5.29E-01 | 6.76E-01 | -6.287 |
| SAMD3 | -0.075 | 5.751 | -0.629 | 5.30E-01 | 6.76E-01 | -6.287 |
| NFAT5 | 0.064 | 9.467 | 0.628 | 5.30E-01 | 6.76E-01 | -6.287 |
| LGI2 | 0.087 | 5.508 | 0.628 | 5.30E-01 | 6.76E-01 | -6.288 |
| ST3GAL4 | -0.098 | 9.132 | -0.628 | 5.31E-01 | 6.77E-01 | -6.288 |
| CA3 | 0.071 | 4.868 | 0.627 | 5.31E-01 | 6.77E-01 | -6.288 |
| UBASH3A | 0.068 | 5.667 | 0.626 | 5.31E-01 | 6.77E-01 | -6.289 |
| CACNG6 | 0.086 | 6.011 | 0.626 | 5.31E-01 | 6.77E-01 | -6.289 |
| ALDH1L1 | 0.071 | 4.897 | 0.626 | 5.31E-01 | 6.77E-01 | -6.289 |
| POMC | -0.099 | 7.131 | -0.627 | 5.32E-01 | 6.77E-01 | -6.289 |
| CSF3 | 0.091 | 5.278 | 0.625 | 5.32E-01 | 6.78E-01 | -6.289 |
| WBP2 | -0.058 | 10.933 | -0.625 | 5.33E-01 | 6.79E-01 | -6.29 |
| MMP3 | 0.09 | 5.586 | 0.624 | 5.33E-01 | 6.79E-01 | -6.29 |
| ADCY6 | 0.073 | 7.423 | 0.623 | 5.33E-01 | 6.79E-01 | -6.29 |
| DVL1 | -0.055 | 8.338 | -0.624 | 5.33E-01 | 6.79E-01 | -6.29 |
| GBP4 | -0.11 | 8.868 | -0.624 | 5.33E-01 | 6.79E-01 | -6.291 |
| RBM17 | -0.043 | 9.803 | -0.624 | 5.34E-01 | 6.79E-01 | -6.291 |
| CNTNAP2 | -0.075 | 5.594 | -0.624 | 5.34E-01 | 6.79E-01 | -6.291 |
| TBX22 | 0.053 | 4.555 | 0.622 | 5.34E-01 | 6.80E-01 | -6.291 |
| GNL3L | 0.06 | 8.465 | 0.622 | 5.34E-01 | 6.80E-01 | -6.291 |
| SOCS3 | 0.079 | 8.002 | 0.621 | 5.34E-01 | 6.80E-01 | -6.292 |
| TDRKH | 0.066 | 6.418 | 0.621 | 5.35E-01 | 6.80E-01 | -6.292 |
| TFDP2 | 0.059 | 8.369 | 0.621 | 5.35E-01 | 6.80E-01 | -6.292 |
| NXPH2 | -0.058 | 4.815 | -0.621 | 5.35E-01 | 6.81E-01 | -6.292 |
| EHD3 | -0.076 | 8.085 | -0.62 | 5.36E-01 | 6.81E-01 | -6.293 |
| FGFR2 | 0.049 | 6.392 | 0.619 | 5.36E-01 | 6.81E-01 | -6.293 |
| HOMER2 | -0.093 | 5.857 | -0.619 | 5.36E-01 | 6.81E-01 | -6.293 |
| HPSE2 | -0.072 | 5.278 | -0.619 | 5.37E-01 | 6.82E-01 | -6.294 |
| CAMK2D | 0.058 | 6.863 | 0.618 | 5.37E-01 | 6.82E-01 | -6.294 |
| PQBP1 | 0.037 | 8.575 | 0.617 | 5.37E-01 | 6.82E-01 | -6.294 |
| SERPINC1 | -0.078 | 5.432 | -0.618 | 5.37E-01 | 6.82E-01 | -6.294 |
| NEU4 | 0.077 | 5.265 | 0.616 | 5.38E-01 | 6.83E-01 | -6.295 |
| ADRBK1 | 0.062 | 8.432 | 0.616 | 5.38E-01 | 6.83E-01 | -6.295 |
| PTGES2 | 0.063 | 8.281 | 0.615 | 5.38E-01 | 6.83E-01 | -6.295 |
| RHPN2 | 0.078 | 7.438 | 0.615 | 5.39E-01 | 6.83E-01 | -6.295 |
| CCL5 | 0.133 | 8.532 | 0.615 | 5.39E-01 | 6.83E-01 | -6.295 |
| CDH19 | 0.063 | 5.988 | 0.614 | 5.39E-01 | 6.84E-01 | -6.296 |
| NEUROD4 | -0.054 | 4.696 | -0.614 | 5.40E-01 | 6.84E-01 | -6.296 |
| KCNC2 | 0.05 | 4.696 | 0.612 | 5.40E-01 | 6.85E-01 | -6.297 |
| BRAP | 0.057 | 6.932 | 0.612 | 5.41E-01 | 6.85E-01 | -6.297 |
| LZTS1 | 0.072 | 7.012 | 0.612 | 5.41E-01 | 6.85E-01 | -6.297 |
| C1R | 0.072 | 8.735 | 0.612 | 5.41E-01 | 6.85E-01 | -6.297 |
| TEKT3 | -0.065 | 5.001 | -0.612 | 5.41E-01 | 6.85E-01 | -6.298 |
| TFE3 | 0.058 | 9.133 | 0.611 | 5.41E-01 | 6.85E-01 | -6.298 |
| TNFRSF11A | -0.07 | 5.96 | -0.612 | 5.41E-01 | 6.85E-01 | -6.298 |
| CA9 | -0.092 | 5.326 | -0.612 | 5.41E-01 | 6.85E-01 | -6.298 |
| CCT4 | -0.046 | 10.627 | -0.611 | 5.42E-01 | 6.86E-01 | -6.298 |
| RAC2 | -0.062 | 9.505 | -0.611 | 5.42E-01 | 6.86E-01 | -6.299 |
| DSG2 | 0.101 | 6.774 | 0.609 | 5.42E-01 | 6.86E-01 | -6.299 |
| IQCF2 | 0.067 | 5.272 | 0.609 | 5.43E-01 | 6.87E-01 | -6.299 |
| AKAP10 | 0.079 | 7.836 | 0.608 | 5.43E-01 | 6.87E-01 | -6.3 |
| GNB5 | 0.066 | 6.509 | 0.607 | 5.44E-01 | 6.88E-01 | -6.3 |
| THOP1 | -0.081 | 10.386 | -0.607 | 5.44E-01 | 6.88E-01 | -6.301 |
| RRS1 | 0.059 | 9.138 | 0.606 | 5.45E-01 | 6.88E-01 | -6.301 |
| OLFM4 | 0.108 | 6.033 | 0.605 | 5.45E-01 | 6.89E-01 | -6.301 |
| SPATA12 | -0.076 | 5.13 | -0.606 | 5.45E-01 | 6.89E-01 | -6.301 |
| ZNF221 | -0.057 | 5.243 | -0.606 | 5.45E-01 | 6.89E-01 | -6.302 |
| BCOR | -0.053 | 7.159 | -0.606 | 5.45E-01 | 6.89E-01 | -6.302 |
| MPP4 | 0.071 | 5.042 | 0.604 | 5.46E-01 | 6.89E-01 | -6.302 |
| ZRANB3 | -0.056 | 5.612 | -0.605 | 5.46E-01 | 6.89E-01 | -6.302 |
| ADRB1 | 0.057 | 7.689 | 0.603 | 5.46E-01 | 6.90E-01 | -6.302 |
| MAG | 0.079 | 6.214 | 0.602 | 5.47E-01 | 6.90E-01 | -6.303 |
| ALDH4A1 | 0.059 | 8.367 | 0.602 | 5.47E-01 | 6.90E-01 | -6.303 |
| SRRM2 | -0.054 | 9.738 | -0.603 | 5.47E-01 | 6.90E-01 | -6.303 |
| CAPG | -0.082 | 9.026 | -0.603 | 5.47E-01 | 6.90E-01 | -6.303 |
| RIBC1 | 0.047 | 5.13 | 0.602 | 5.47E-01 | 6.91E-01 | -6.303 |
| NFKB1 | -0.059 | 10.355 | -0.602 | 5.48E-01 | 6.91E-01 | -6.303 |
| LYST | -0.054 | 8.067 | -0.602 | 5.48E-01 | 6.91E-01 | -6.304 |
| PRLR | -0.073 | 7.498 | -0.601 | 5.48E-01 | 6.91E-01 | -6.304 |
| AKAP14 | 0.07 | 4.86 | 0.598 | 5.50E-01 | 6.93E-01 | -6.305 |
| GLP1R | 0.053 | 4.777 | 0.597 | 5.50E-01 | 6.94E-01 | -6.306 |
| SLC25A11 | 0.057 | 8.979 | 0.595 | 5.52E-01 | 6.95E-01 | -6.307 |
| RNF32 | -0.073 | 5.716 | -0.596 | 5.52E-01 | 6.95E-01 | -6.307 |
| SCGB1D1 | -0.084 | 4.755 | -0.596 | 5.52E-01 | 6.95E-01 | -6.308 |
| COQ6 | 0.052 | 7.773 | 0.594 | 5.52E-01 | 6.95E-01 | -6.308 |
| SMARCC1 | -0.049 | 9.491 | -0.595 | 5.52E-01 | 6.95E-01 | -6.308 |
| ELK4 | -0.06 | 6.494 | -0.595 | 5.52E-01 | 6.95E-01 | -6.308 |
| USP29 | -0.059 | 4.694 | -0.594 | 5.53E-01 | 6.96E-01 | -6.308 |
| NTF3 | 0.073 | 6.048 | 0.593 | 5.53E-01 | 6.96E-01 | -6.308 |
| HEY2 | 0.066 | 5.937 | 0.593 | 5.53E-01 | 6.96E-01 | -6.309 |
| SNRPD3 | 0.053 | 9.082 | 0.592 | 5.54E-01 | 6.97E-01 | -6.309 |
| ABCA4 | 0.081 | 5.504 | 0.592 | 5.54E-01 | 6.97E-01 | -6.309 |
| YTHDF1 | 0.044 | 10.095 | 0.591 | 5.54E-01 | 6.97E-01 | -6.309 |
| CCR7 | 0.078 | 7.859 | 0.59 | 5.55E-01 | 6.98E-01 | -6.31 |
| PSME3 | 0.045 | 9.199 | 0.59 | 5.55E-01 | 6.98E-01 | -6.31 |
| TRPV3 | 0.089 | 4.983 | 0.59 | 5.55E-01 | 6.98E-01 | -6.31 |
| UNC5D | 0.063 | 4.594 | 0.589 | 5.56E-01 | 6.98E-01 | -6.311 |
| NDRG4 | 0.089 | 7.008 | 0.589 | 5.56E-01 | 6.98E-01 | -6.311 |
| SIRT3 | 0.039 | 7.305 | 0.589 | 5.56E-01 | 6.98E-01 | -6.311 |
| TGFBR1 | -0.066 | 6.629 | -0.589 | 5.56E-01 | 6.99E-01 | -6.311 |
| STMN1 | 0.054 | 8.459 | 0.588 | 5.56E-01 | 6.99E-01 | -6.311 |
| NOXO1 | 0.079 | 6.134 | 0.588 | 5.56E-01 | 6.99E-01 | -6.311 |
| IDI2 | -0.074 | 5.191 | -0.589 | 5.57E-01 | 6.99E-01 | -6.312 |
| CHGB | -0.068 | 5.08 | -0.588 | 5.57E-01 | 6.99E-01 | -6.312 |
| RPS21 | -0.061 | 12.337 | -0.588 | 5.57E-01 | 6.99E-01 | -6.312 |
| KLRC4 | -0.08 | 6.135 | -0.588 | 5.57E-01 | 6.99E-01 | -6.312 |
| AGPAT3 | 0.064 | 7.916 | 0.587 | 5.57E-01 | 6.99E-01 | -6.312 |
| GKN1 | 0.167 | 6.169 | 0.586 | 5.58E-01 | 6.99E-01 | -6.312 |
| BARX1 | 0.063 | 4.681 | 0.586 | 5.58E-01 | 7.00E-01 | -6.313 |
| PKDREJ | 0.06 | 4.691 | 0.586 | 5.58E-01 | 7.00E-01 | -6.313 |
| TTC16 | 0.074 | 6.474 | 0.585 | 5.58E-01 | 7.00E-01 | -6.313 |
| DIRAS2 | 0.098 | 5.017 | 0.585 | 5.59E-01 | 7.00E-01 | -6.313 |
| SLC30A2 | 0.099 | 8.164 | 0.585 | 5.59E-01 | 7.00E-01 | -6.313 |
| SMPD2 | 0.074 | 7.518 | 0.584 | 5.59E-01 | 7.00E-01 | -6.314 |
| STARD8 | -0.059 | 8.571 | -0.584 | 5.60E-01 | 7.01E-01 | -6.314 |
| ATP2A1 | -0.071 | 5.187 | -0.584 | 5.60E-01 | 7.01E-01 | -6.314 |
| TKT | -0.065 | 12.375 | -0.583 | 5.60E-01 | 7.02E-01 | -6.315 |
| MSR1 | 0.06 | 5.89 | 0.582 | 5.60E-01 | 7.02E-01 | -6.315 |
| ZNF530 | -0.068 | 5.752 | -0.583 | 5.61E-01 | 7.02E-01 | -6.315 |
| DLX1 | -0.064 | 5.428 | -0.582 | 5.61E-01 | 7.02E-01 | -6.315 |
| NOL6 | 0.062 | 7.621 | 0.58 | 5.62E-01 | 7.03E-01 | -6.316 |
| ELMO1 | 0.079 | 7.378 | 0.58 | 5.62E-01 | 7.03E-01 | -6.316 |
| ALDH3A2 | 0.043 | 7.838 | 0.58 | 5.62E-01 | 7.03E-01 | -6.316 |
| FAH | -0.069 | 8.013 | -0.58 | 5.62E-01 | 7.03E-01 | -6.316 |
| KRT5 | 0.119 | 5.077 | 0.579 | 5.63E-01 | 7.03E-01 | -6.316 |
| IQSEC1 | -0.053 | 8.958 | -0.58 | 5.63E-01 | 7.03E-01 | -6.317 |
| CBLN2 | 0.069 | 5.053 | 0.579 | 5.63E-01 | 7.03E-01 | -6.317 |
| SH3GLB2 | 0.072 | 9.414 | 0.578 | 5.63E-01 | 7.04E-01 | -6.317 |
| KCNA1 | -0.048 | 4.695 | -0.578 | 5.64E-01 | 7.04E-01 | -6.317 |
| SERPINI1 | -0.105 | 7.052 | -0.578 | 5.64E-01 | 7.04E-01 | -6.317 |
| HHLA2 | 0.048 | 5.144 | 0.577 | 5.64E-01 | 7.04E-01 | -6.318 |
| FLT3 | 0.077 | 5.973 | 0.577 | 5.64E-01 | 7.05E-01 | -6.318 |
| RABAC1 | 0.039 | 12.365 | 0.576 | 5.64E-01 | 7.05E-01 | -6.318 |
| TP53BP2 | -0.055 | 8.668 | -0.577 | 5.65E-01 | 7.05E-01 | -6.318 |
| TM9SF4 | -0.042 | 10.239 | -0.577 | 5.65E-01 | 7.05E-01 | -6.318 |
| SLC11A1 | 0.055 | 6.925 | 0.576 | 5.65E-01 | 7.05E-01 | -6.318 |
| ENPP6 | 0.069 | 4.667 | 0.575 | 5.65E-01 | 7.05E-01 | -6.319 |
| IVD | 0.054 | 7.701 | 0.574 | 5.66E-01 | 7.06E-01 | -6.319 |
| C1orf21 | 0.072 | 7.198 | 0.573 | 5.66E-01 | 7.06E-01 | -6.32 |
| C22orf23 | -0.064 | 5.093 | -0.574 | 5.66E-01 | 7.06E-01 | -6.32 |
| TNRC6A | 0.056 | 7.066 | 0.573 | 5.67E-01 | 7.06E-01 | -6.32 |
| ARC | 0.082 | 5.666 | 0.572 | 5.68E-01 | 7.07E-01 | -6.321 |
| ELF2 | 0.053 | 8.04 | 0.572 | 5.68E-01 | 7.07E-01 | -6.321 |
| ZFHX4 | 0.058 | 4.995 | 0.571 | 5.68E-01 | 7.07E-01 | -6.321 |
| PUS1 | -0.054 | 8.515 | -0.572 | 5.68E-01 | 7.07E-01 | -6.321 |
| HOXB1 | -0.048 | 5.809 | -0.572 | 5.68E-01 | 7.07E-01 | -6.321 |
| EHD2 | -0.076 | 8.08 | -0.572 | 5.68E-01 | 7.07E-01 | -6.321 |
| TPSG1 | 0.077 | 9.195 | 0.57 | 5.69E-01 | 7.08E-01 | -6.322 |
| CLIC1 | -0.037 | 12.195 | -0.57 | 5.69E-01 | 7.08E-01 | -6.322 |
| IL23R | -0.067 | 4.825 | -0.57 | 5.69E-01 | 7.09E-01 | -6.322 |
| AOAH | -0.067 | 7.141 | -0.57 | 5.69E-01 | 7.09E-01 | -6.322 |
| CSE1L | 0.044 | 10.329 | 0.568 | 5.70E-01 | 7.09E-01 | -6.323 |
| OLFM1 | -0.057 | 5.632 | -0.569 | 5.70E-01 | 7.09E-01 | -6.323 |
| DCTN6 | -0.047 | 9.738 | -0.568 | 5.70E-01 | 7.09E-01 | -6.323 |
| GADD45GIP1 | 0.052 | 9.306 | 0.567 | 5.70E-01 | 7.09E-01 | -6.323 |
| GUCY1B3 | -0.081 | 7.528 | -0.568 | 5.70E-01 | 7.09E-01 | -6.323 |
| TMEM42 | 0.056 | 9.068 | 0.567 | 5.71E-01 | 7.09E-01 | -6.323 |
| NEUROD6 | 0.047 | 4.633 | 0.567 | 5.71E-01 | 7.09E-01 | -6.323 |
| CLEC1A | 0.061 | 8.447 | 0.567 | 5.71E-01 | 7.09E-01 | -6.323 |
| ZAN | 0.065 | 5.813 | 0.567 | 5.71E-01 | 7.09E-01 | -6.323 |
| MS4A5 | 0.071 | 5.013 | 0.565 | 5.72E-01 | 7.10E-01 | -6.324 |
| NRG1 | 0.056 | 5.927 | 0.565 | 5.72E-01 | 7.10E-01 | -6.324 |
| ATF5 | 0.078 | 8.16 | 0.564 | 5.72E-01 | 7.11E-01 | -6.325 |
| ACP2 | 0.059 | 8.443 | 0.563 | 5.74E-01 | 7.12E-01 | -6.326 |
| PCDHB12 | 0.071 | 5.281 | 0.562 | 5.74E-01 | 7.13E-01 | -6.326 |
| PNMA1 | -0.052 | 9.565 | -0.563 | 5.74E-01 | 7.13E-01 | -6.326 |
| IPO13 | -0.053 | 7.508 | -0.562 | 5.75E-01 | 7.13E-01 | -6.326 |
| EMILIN3 | 0.076 | 6.231 | 0.561 | 5.75E-01 | 7.13E-01 | -6.327 |
| CHRNA4 | 0.066 | 6.376 | 0.561 | 5.75E-01 | 7.13E-01 | -6.327 |
| ANKRD11 | 0.046 | 8.497 | 0.561 | 5.75E-01 | 7.13E-01 | -6.327 |
| TIMM9 | 0.046 | 9.731 | 0.56 | 5.75E-01 | 7.14E-01 | -6.327 |
| ARPC2 | -0.034 | 11.829 | -0.56 | 5.76E-01 | 7.14E-01 | -6.327 |
| S100A11 | 0.047 | 12.467 | 0.559 | 5.76E-01 | 7.14E-01 | -6.328 |
| SCGB3A1 | -0.103 | 7.11 | -0.56 | 5.76E-01 | 7.14E-01 | -6.328 |
| PRDM4 | 0.038 | 9.149 | 0.559 | 5.76E-01 | 7.14E-01 | -6.328 |
| ZFP91 | 0.059 | 9.31 | 0.559 | 5.76E-01 | 7.14E-01 | -6.328 |
| NTAN1 | 0.041 | 8.818 | 0.559 | 5.76E-01 | 7.14E-01 | -6.328 |
| SH2D3C | -0.068 | 8.154 | -0.56 | 5.76E-01 | 7.14E-01 | -6.328 |
| HCST | 0.058 | 10.197 | 0.559 | 5.76E-01 | 7.14E-01 | -6.328 |
| RPP38 | 0.034 | 8.321 | 0.558 | 5.77E-01 | 7.14E-01 | -6.328 |
| YPEL2 | -0.062 | 8.3 | -0.559 | 5.77E-01 | 7.14E-01 | -6.328 |
| TSKS | -0.107 | 6.834 | -0.558 | 5.78E-01 | 7.15E-01 | -6.329 |
| SRI | -0.046 | 9.036 | -0.558 | 5.78E-01 | 7.15E-01 | -6.329 |
| PDK3 | -0.062 | 7.75 | -0.557 | 5.78E-01 | 7.16E-01 | -6.33 |
| HBB | 0.061 | 12.349 | 0.555 | 5.79E-01 | 7.16E-01 | -6.33 |
| SEMA3D | -0.073 | 5.298 | -0.556 | 5.79E-01 | 7.16E-01 | -6.33 |
| RAB3C | 0.066 | 5.021 | 0.555 | 5.79E-01 | 7.16E-01 | -6.33 |
| GALNT10 | 0.049 | 7.302 | 0.555 | 5.79E-01 | 7.16E-01 | -6.33 |
| KIF1B | 0.056 | 7.951 | 0.554 | 5.79E-01 | 7.16E-01 | -6.33 |
| GOT2 | -0.037 | 10.527 | -0.555 | 5.79E-01 | 7.16E-01 | -6.33 |
| DCLRE1C | 0.038 | 7.843 | 0.554 | 5.79E-01 | 7.16E-01 | -6.33 |
| FOXK2 | -0.042 | 7.437 | -0.554 | 5.80E-01 | 7.17E-01 | -6.331 |
| KLK13 | -0.047 | 5.013 | -0.554 | 5.80E-01 | 7.17E-01 | -6.331 |
| MAPK8IP2 | 0.059 | 6.079 | 0.553 | 5.80E-01 | 7.17E-01 | -6.331 |
| HOXA1 | -0.064 | 5.063 | -0.554 | 5.81E-01 | 7.17E-01 | -6.331 |
| NUFIP1 | 0.056 | 6.294 | 0.553 | 5.81E-01 | 7.17E-01 | -6.331 |
| MRO | 0.051 | 4.98 | 0.552 | 5.81E-01 | 7.17E-01 | -6.331 |
| PTGS1 | -0.074 | 7.021 | -0.553 | 5.81E-01 | 7.17E-01 | -6.332 |
| TNMD | 0.067 | 5.873 | 0.552 | 5.81E-01 | 7.17E-01 | -6.332 |
| EDARADD | 0.092 | 8.486 | 0.551 | 5.81E-01 | 7.17E-01 | -6.332 |
| CDR1 | -0.05 | 4.576 | -0.552 | 5.81E-01 | 7.17E-01 | -6.332 |
| SIRPB1 | -0.059 | 6.242 | -0.552 | 5.82E-01 | 7.17E-01 | -6.332 |
| HEY1 | -0.086 | 8.707 | -0.552 | 5.82E-01 | 7.17E-01 | -6.332 |
| ZNF121 | -0.075 | 5.685 | -0.552 | 5.82E-01 | 7.17E-01 | -6.332 |
| XG | -0.087 | 6.008 | -0.551 | 5.82E-01 | 7.17E-01 | -6.332 |
| MXD3 | -0.059 | 7.875 | -0.55 | 5.83E-01 | 7.18E-01 | -6.333 |
| TMIE | 0.067 | 5.43 | 0.549 | 5.83E-01 | 7.18E-01 | -6.333 |
| DMBT1 | 0.051 | 4.813 | 0.548 | 5.84E-01 | 7.19E-01 | -6.334 |
| FBXO6 | 0.072 | 7.176 | 0.548 | 5.84E-01 | 7.19E-01 | -6.334 |
| EBPL | 0.059 | 9.481 | 0.548 | 5.84E-01 | 7.19E-01 | -6.334 |
| UPF2 | -0.041 | 8.5 | -0.549 | 5.84E-01 | 7.19E-01 | -6.334 |
| OR6A2 | 0.062 | 5.091 | 0.548 | 5.84E-01 | 7.19E-01 | -6.334 |
| PLA2G6 | 0.07 | 6.872 | 0.548 | 5.84E-01 | 7.19E-01 | -6.334 |
| CASP9 | 0.045 | 8.538 | 0.546 | 5.85E-01 | 7.19E-01 | -6.334 |
| ACTL7A | -0.075 | 5.807 | -0.547 | 5.85E-01 | 7.19E-01 | -6.334 |
| KIF11 | -0.08 | 7.735 | -0.547 | 5.85E-01 | 7.19E-01 | -6.335 |
| OAS1 | 0.102 | 8.14 | 0.546 | 5.85E-01 | 7.19E-01 | -6.335 |
| CTDSP1 | -0.043 | 11.033 | -0.547 | 5.85E-01 | 7.19E-01 | -6.335 |
| KIAA0753 | 0.037 | 8.157 | 0.545 | 5.85E-01 | 7.19E-01 | -6.335 |
| NDUFB6 | -0.043 | 10.27 | -0.546 | 5.85E-01 | 7.19E-01 | -6.335 |
| HIST1H2BA | -0.056 | 4.904 | -0.546 | 5.85E-01 | 7.19E-01 | -6.335 |
| CDKL2 | -0.046 | 4.766 | -0.546 | 5.86E-01 | 7.20E-01 | -6.335 |
| RUFY1 | 0.046 | 9.798 | 0.545 | 5.86E-01 | 7.20E-01 | -6.336 |
| FSHR | 0.068 | 5.154 | 0.543 | 5.87E-01 | 7.21E-01 | -6.336 |
| EXOSC9 | -0.057 | 9.328 | -0.544 | 5.87E-01 | 7.21E-01 | -6.336 |
| OGFRL1 | -0.053 | 9.22 | -0.542 | 5.88E-01 | 7.22E-01 | -6.337 |
| SCTR | 0.061 | 5.242 | 0.541 | 5.88E-01 | 7.22E-01 | -6.337 |
| SNAP25 | 0.059 | 5.53 | 0.54 | 5.89E-01 | 7.23E-01 | -6.338 |
| CEBPA | 0.065 | 10.265 | 0.539 | 5.89E-01 | 7.23E-01 | -6.338 |
| IL22RA1 | 0.052 | 5.045 | 0.539 | 5.90E-01 | 7.24E-01 | -6.339 |
| CABP1 | 0.058 | 5.078 | 0.538 | 5.90E-01 | 7.24E-01 | -6.339 |
| SCN9A | -0.07 | 5.987 | -0.539 | 5.91E-01 | 7.24E-01 | -6.339 |
| HIST1H2BO | -0.065 | 8.246 | -0.538 | 5.91E-01 | 7.25E-01 | -6.34 |
| KCNJ16 | -0.115 | 5.952 | -0.538 | 5.91E-01 | 7.25E-01 | -6.34 |
| CD9 | -0.086 | 9.733 | -0.538 | 5.91E-01 | 7.25E-01 | -6.34 |
| SLC6A13 | -0.044 | 6.165 | -0.537 | 5.92E-01 | 7.25E-01 | -6.34 |
| CUL7 | 0.052 | 7.699 | 0.536 | 5.92E-01 | 7.25E-01 | -6.34 |
| AP1G1 | -0.041 | 8.928 | -0.537 | 5.92E-01 | 7.25E-01 | -6.34 |
| INHBB | 0.101 | 7.044 | 0.536 | 5.92E-01 | 7.25E-01 | -6.34 |
| FAT4 | -0.062 | 6.046 | -0.537 | 5.92E-01 | 7.25E-01 | -6.34 |
| INPP5E | -0.054 | 7.741 | -0.537 | 5.92E-01 | 7.25E-01 | -6.34 |
| DCLRE1B | -0.065 | 6.868 | -0.536 | 5.92E-01 | 7.25E-01 | -6.34 |
| RBMS2 | -0.06 | 8.666 | -0.536 | 5.93E-01 | 7.26E-01 | -6.341 |
| NUP98 | -0.048 | 8.535 | -0.535 | 5.93E-01 | 7.26E-01 | -6.341 |
| TRIM40 | -0.096 | 5.949 | -0.534 | 5.94E-01 | 7.26E-01 | -6.341 |
| C1QL1 | 0.069 | 5.759 | 0.533 | 5.94E-01 | 7.26E-01 | -6.341 |
| RTEL1 | 0.047 | 8.453 | 0.533 | 5.94E-01 | 7.27E-01 | -6.342 |
| ZNF76 | 0.061 | 7.391 | 0.532 | 5.94E-01 | 7.27E-01 | -6.342 |
| SPRY1 | 0.06 | 7.859 | 0.531 | 5.95E-01 | 7.28E-01 | -6.343 |
| SMOC1 | 0.067 | 6.507 | 0.531 | 5.95E-01 | 7.28E-01 | -6.343 |
| ZFYVE9 | -0.046 | 5.578 | -0.531 | 5.96E-01 | 7.28E-01 | -6.343 |
| OR51B4 | 0.072 | 4.778 | 0.529 | 5.96E-01 | 7.29E-01 | -6.343 |
| C6orf10 | 0.057 | 4.846 | 0.529 | 5.96E-01 | 7.29E-01 | -6.343 |
| CDKAL1 | 0.053 | 7.263 | 0.529 | 5.97E-01 | 7.29E-01 | -6.344 |
| PTPRR | -0.072 | 5.325 | -0.53 | 5.97E-01 | 7.29E-01 | -6.344 |
| ABR | -0.046 | 9.1 | -0.529 | 5.97E-01 | 7.29E-01 | -6.344 |
| BNIP3L | -0.06 | 11.218 | -0.529 | 5.98E-01 | 7.30E-01 | -6.344 |
| GBX2 | -0.077 | 5.94 | -0.528 | 5.98E-01 | 7.30E-01 | -6.345 |
| CPA3 | 0.091 | 6.634 | 0.527 | 5.98E-01 | 7.31E-01 | -6.345 |
| ERCC4 | -0.052 | 5.97 | -0.527 | 5.99E-01 | 7.31E-01 | -6.345 |
| BECN1 | -0.036 | 9.639 | -0.527 | 5.99E-01 | 7.31E-01 | -6.345 |
| IL1RN | -0.062 | 6.238 | -0.527 | 5.99E-01 | 7.31E-01 | -6.345 |
| CLCN7 | 0.057 | 9.045 | 0.526 | 5.99E-01 | 7.31E-01 | -6.345 |
| AMBP | 0.045 | 4.834 | 0.525 | 6.00E-01 | 7.31E-01 | -6.346 |
| CAT | 0.048 | 9.824 | 0.525 | 6.00E-01 | 7.31E-01 | -6.346 |
| MTSS1 | 0.059 | 8.285 | 0.524 | 6.00E-01 | 7.31E-01 | -6.346 |
| MITF | -0.049 | 6.82 | -0.525 | 6.00E-01 | 7.31E-01 | -6.346 |
| DMRT1 | -0.092 | 5.157 | -0.525 | 6.00E-01 | 7.31E-01 | -6.346 |
| ZHX2 | 0.052 | 7.33 | 0.524 | 6.00E-01 | 7.31E-01 | -6.346 |
| PTPN13 | -0.082 | 7.334 | -0.525 | 6.00E-01 | 7.31E-01 | -6.346 |
| PLCB2 | 0.06 | 7.214 | 0.524 | 6.00E-01 | 7.31E-01 | -6.346 |
| TIMP4 | 0.076 | 6.243 | 0.521 | 6.02E-01 | 7.33E-01 | -6.348 |
| ALDH3A1 | -0.068 | 4.909 | -0.522 | 6.02E-01 | 7.33E-01 | -6.348 |
| PLK1 | 0.064 | 7.085 | 0.521 | 6.02E-01 | 7.33E-01 | -6.348 |
| ITIH4 | 0.052 | 6.665 | 0.521 | 6.02E-01 | 7.33E-01 | -6.348 |
| PPP2R5E | -0.046 | 9.791 | -0.521 | 6.03E-01 | 7.34E-01 | -6.348 |
| TCF19 | -0.06 | 6.102 | -0.52 | 6.04E-01 | 7.35E-01 | -6.349 |
| RTN4RL1 | 0.051 | 6.37 | 0.519 | 6.04E-01 | 7.35E-01 | -6.349 |
| DEFB119 | 0.043 | 4.778 | 0.519 | 6.04E-01 | 7.35E-01 | -6.349 |
| MAGEB1 | 0.066 | 5.339 | 0.518 | 6.04E-01 | 7.35E-01 | -6.349 |
| TOE1 | -0.053 | 7.773 | -0.519 | 6.04E-01 | 7.35E-01 | -6.349 |
| PEA15 | 0.038 | 10.919 | 0.518 | 6.04E-01 | 7.35E-01 | -6.349 |
| DDX31 | -0.048 | 6.201 | -0.517 | 6.06E-01 | 7.37E-01 | -6.351 |
| SUCNR1 | -0.067 | 5.021 | -0.516 | 6.06E-01 | 7.37E-01 | -6.351 |
| CNGA3 | -0.05 | 4.592 | -0.516 | 6.06E-01 | 7.37E-01 | -6.351 |
| HK2 | 0.075 | 7.2 | 0.515 | 6.06E-01 | 7.37E-01 | -6.351 |
| SLC30A3 | -0.062 | 6.22 | -0.516 | 6.06E-01 | 7.37E-01 | -6.351 |
| NUP205 | -0.039 | 9.925 | -0.515 | 6.07E-01 | 7.37E-01 | -6.351 |
| GAS7 | 0.047 | 7.153 | 0.514 | 6.07E-01 | 7.37E-01 | -6.351 |
| MYEF2 | -0.063 | 5.657 | -0.514 | 6.08E-01 | 7.38E-01 | -6.352 |
| PARP9 | 0.06 | 8.866 | 0.513 | 6.08E-01 | 7.38E-01 | -6.352 |
| POLR3H | -0.048 | 7.725 | -0.514 | 6.08E-01 | 7.38E-01 | -6.352 |
| ZNF436 | -0.045 | 7.175 | -0.514 | 6.08E-01 | 7.38E-01 | -6.352 |
| LRCH2 | 0.061 | 6.669 | 0.512 | 6.08E-01 | 7.38E-01 | -6.352 |
| SLC17A6 | 0.045 | 4.642 | 0.512 | 6.08E-01 | 7.38E-01 | -6.352 |
| INE1 | 0.062 | 6.375 | 0.511 | 6.09E-01 | 7.39E-01 | -6.353 |
| BCL7A | -0.054 | 6.797 | -0.512 | 6.09E-01 | 7.39E-01 | -6.353 |
| DDOST | -0.04 | 11.849 | -0.512 | 6.09E-01 | 7.39E-01 | -6.353 |
| EXOSC2 | 0.038 | 7.77 | 0.51 | 6.10E-01 | 7.39E-01 | -6.353 |
| RND3 | -0.097 | 8.786 | -0.511 | 6.10E-01 | 7.39E-01 | -6.353 |
| OSBPL10 | 0.056 | 8.33 | 0.51 | 6.10E-01 | 7.39E-01 | -6.354 |
| GOT1 | -0.041 | 9.461 | -0.511 | 6.10E-01 | 7.39E-01 | -6.354 |
| ETS2 | -0.065 | 8.796 | -0.511 | 6.10E-01 | 7.39E-01 | -6.354 |
| SFXN1 | 0.052 | 7.095 | 0.51 | 6.10E-01 | 7.39E-01 | -6.354 |
| MYH7 | -0.059 | 5.132 | -0.51 | 6.10E-01 | 7.39E-01 | -6.354 |
| TGFBI | 0.063 | 12.329 | 0.509 | 6.11E-01 | 7.39E-01 | -6.354 |
| HLA-G | -0.08 | 10.463 | -0.51 | 6.11E-01 | 7.40E-01 | -6.354 |
| GFOD1 | -0.053 | 8.289 | -0.509 | 6.11E-01 | 7.40E-01 | -6.354 |
| SSX3 | 0.078 | 5.54 | 0.506 | 6.13E-01 | 7.41E-01 | -6.355 |
| DRD3 | 0.061 | 5.538 | 0.505 | 6.13E-01 | 7.42E-01 | -6.356 |
| MAB21L2 | 0.093 | 6.035 | 0.505 | 6.14E-01 | 7.42E-01 | -6.356 |
| CTTNBP2 | 0.055 | 5.69 | 0.504 | 6.14E-01 | 7.42E-01 | -6.356 |
| FRMD1 | 0.071 | 6.426 | 0.504 | 6.14E-01 | 7.42E-01 | -6.356 |
| TRIB1 | 0.057 | 9.943 | 0.504 | 6.14E-01 | 7.43E-01 | -6.356 |
| COL15A1 | -0.058 | 8.833 | -0.505 | 6.14E-01 | 7.43E-01 | -6.357 |
| TCEAL7 | -0.063 | 5.877 | -0.505 | 6.14E-01 | 7.43E-01 | -6.357 |
| PHF20 | 0.044 | 7.498 | 0.503 | 6.15E-01 | 7.43E-01 | -6.357 |
| KCNQ2 | 0.066 | 5.667 | 0.502 | 6.15E-01 | 7.43E-01 | -6.357 |
| SRF | -0.06 | 8.996 | -0.503 | 6.16E-01 | 7.44E-01 | -6.357 |
| DDX17 | -0.07 | 9.641 | -0.503 | 6.16E-01 | 7.44E-01 | -6.358 |
| WDR41 | 0.045 | 8.176 | 0.501 | 6.17E-01 | 7.45E-01 | -6.358 |
| FUT11 | 0.049 | 7.64 | 0.5 | 6.17E-01 | 7.45E-01 | -6.359 |
| OR1C1 | 0.047 | 5.029 | 0.499 | 6.17E-01 | 7.45E-01 | -6.359 |
| GAN | 0.047 | 6.464 | 0.499 | 6.18E-01 | 7.45E-01 | -6.359 |
| FAS | -0.065 | 7.806 | -0.5 | 6.18E-01 | 7.46E-01 | -6.359 |
| CANT1 | -0.052 | 9.431 | -0.499 | 6.18E-01 | 7.46E-01 | -6.359 |
| DTNA | 0.047 | 6.224 | 0.498 | 6.18E-01 | 7.46E-01 | -6.359 |
| PLCZ1 | 0.06 | 4.537 | 0.497 | 6.19E-01 | 7.46E-01 | -6.36 |
| KLF11 | -0.05 | 7.571 | -0.498 | 6.19E-01 | 7.47E-01 | -6.36 |
| BMPR1B | 0.044 | 5.361 | 0.496 | 6.20E-01 | 7.47E-01 | -6.36 |
| DCN | 0.071 | 10.006 | 0.496 | 6.20E-01 | 7.47E-01 | -6.36 |
| TNFRSF17 | 0.071 | 5.604 | 0.495 | 6.20E-01 | 7.48E-01 | -6.361 |
| ST7L | -0.046 | 6.491 | -0.496 | 6.21E-01 | 7.48E-01 | -6.361 |
| FGFBP1 | -0.109 | 5.913 | -0.496 | 6.21E-01 | 7.48E-01 | -6.361 |
| GRB14 | -0.053 | 7.757 | -0.495 | 6.21E-01 | 7.48E-01 | -6.361 |
| ARG1 | -0.085 | 6.107 | -0.494 | 6.22E-01 | 7.49E-01 | -6.362 |
| CLDN15 | 0.048 | 6.863 | 0.493 | 6.22E-01 | 7.49E-01 | -6.362 |
| EDAR | -0.058 | 5.62 | -0.494 | 6.22E-01 | 7.49E-01 | -6.362 |
| MYOD1 | -0.068 | 6.718 | -0.493 | 6.22E-01 | 7.49E-01 | -6.362 |
| INS | 0.058 | 6.022 | 0.492 | 6.23E-01 | 7.49E-01 | -6.362 |
| NR2F1 | 0.068 | 8.736 | 0.492 | 6.23E-01 | 7.49E-01 | -6.362 |
| PDXK | -0.043 | 9.26 | -0.493 | 6.23E-01 | 7.49E-01 | -6.362 |
| LBX1 | 0.066 | 7.491 | 0.491 | 6.23E-01 | 7.50E-01 | -6.363 |
| PFN1 | 0.048 | 12.161 | 0.49 | 6.24E-01 | 7.51E-01 | -6.363 |
| RIN3 | 0.05 | 7.288 | 0.49 | 6.24E-01 | 7.51E-01 | -6.363 |
| ANXA5 | -0.038 | 13.155 | -0.49 | 6.24E-01 | 7.51E-01 | -6.364 |
| ZNF148 | 0.042 | 9.226 | 0.489 | 6.25E-01 | 7.51E-01 | -6.364 |
| MPP3 | -0.061 | 6.281 | -0.49 | 6.25E-01 | 7.51E-01 | -6.364 |
| MAP1B | -0.064 | 7.232 | -0.489 | 6.25E-01 | 7.51E-01 | -6.364 |
| ADH4 | 0.051 | 4.999 | 0.488 | 6.25E-01 | 7.51E-01 | -6.364 |
| WT1 | 0.102 | 5.771 | 0.487 | 6.26E-01 | 7.52E-01 | -6.365 |
| EPO | -0.052 | 5.195 | -0.487 | 6.26E-01 | 7.52E-01 | -6.365 |
| UBE2I | -0.041 | 9.269 | -0.487 | 6.27E-01 | 7.52E-01 | -6.365 |
| PROX1 | -0.047 | 4.907 | -0.487 | 6.27E-01 | 7.53E-01 | -6.365 |
| MAST2 | 0.051 | 7.305 | 0.486 | 6.27E-01 | 7.53E-01 | -6.365 |
| TBC1D21 | -0.057 | 4.844 | -0.487 | 6.27E-01 | 7.53E-01 | -6.365 |
| CRYM | -0.072 | 6.446 | -0.486 | 6.27E-01 | 7.53E-01 | -6.366 |
| KCNJ2 | 0.06 | 8.115 | 0.485 | 6.28E-01 | 7.53E-01 | -6.366 |
| ALDH1B1 | -0.052 | 6.642 | -0.485 | 6.28E-01 | 7.53E-01 | -6.366 |
| PRG2 | -0.139 | 9.034 | -0.485 | 6.28E-01 | 7.54E-01 | -6.366 |
| CEL | 0.052 | 6.248 | 0.483 | 6.29E-01 | 7.54E-01 | -6.366 |
| CRABP2 | 0.07 | 7.601 | 0.483 | 6.29E-01 | 7.54E-01 | -6.367 |
| CSMD3 | 0.061 | 4.761 | 0.482 | 6.29E-01 | 7.55E-01 | -6.367 |
| PGAP1 | -0.052 | 5.865 | -0.483 | 6.30E-01 | 7.55E-01 | -6.367 |
| PAX9 | -0.049 | 5.571 | -0.482 | 6.30E-01 | 7.55E-01 | -6.367 |
| TNFRSF14 | -0.05 | 9.044 | -0.482 | 6.30E-01 | 7.55E-01 | -6.367 |
| KCND2 | -0.099 | 5.161 | -0.482 | 6.30E-01 | 7.55E-01 | -6.367 |
| AQP6 | 0.047 | 4.86 | 0.481 | 6.30E-01 | 7.55E-01 | -6.368 |
| SLC1A6 | 0.076 | 5.716 | 0.481 | 6.31E-01 | 7.55E-01 | -6.368 |
| PKMYT1 | 0.059 | 8.238 | 0.48 | 6.31E-01 | 7.55E-01 | -6.368 |
| OR51E2 | -0.057 | 5.794 | -0.481 | 6.31E-01 | 7.55E-01 | -6.368 |
| SSTR5 | 0.055 | 5.871 | 0.48 | 6.31E-01 | 7.55E-01 | -6.368 |
| CAV3 | -0.065 | 5.326 | -0.48 | 6.31E-01 | 7.56E-01 | -6.368 |
| ZNF570 | 0.047 | 6.06 | 0.479 | 6.31E-01 | 7.56E-01 | -6.368 |
| ITGB8 | -0.063 | 5.98 | -0.48 | 6.32E-01 | 7.56E-01 | -6.369 |
| GSK3B | 0.046 | 9.209 | 0.479 | 6.32E-01 | 7.56E-01 | -6.369 |
| TBX6 | 0.055 | 5.093 | 0.479 | 6.32E-01 | 7.56E-01 | -6.369 |
| DHX37 | -0.044 | 8.667 | -0.479 | 6.32E-01 | 7.56E-01 | -6.369 |
| RPL36AL | 0.042 | 12.918 | 0.478 | 6.33E-01 | 7.56E-01 | -6.369 |
| THBS4 | 0.071 | 5.618 | 0.478 | 6.33E-01 | 7.56E-01 | -6.369 |
| KBTBD4 | 0.036 | 7.893 | 0.478 | 6.33E-01 | 7.56E-01 | -6.369 |
| CD109 | -0.068 | 6.667 | -0.478 | 6.33E-01 | 7.56E-01 | -6.369 |
| GJB4 | 0.053 | 5.888 | 0.477 | 6.33E-01 | 7.56E-01 | -6.369 |
| FOXF1 | 0.061 | 8.35 | 0.477 | 6.33E-01 | 7.56E-01 | -6.369 |
| BMP10 | 0.055 | 4.947 | 0.477 | 6.33E-01 | 7.56E-01 | -6.369 |
| BTNL3 | -0.115 | 5.524 | -0.478 | 6.33E-01 | 7.56E-01 | -6.37 |
| SH3RF2 | 0.059 | 5.58 | 0.475 | 6.34E-01 | 7.58E-01 | -6.37 |
| OTC | 0.059 | 4.657 | 0.475 | 6.35E-01 | 7.58E-01 | -6.37 |
| MSRA | 0.045 | 8.979 | 0.475 | 6.35E-01 | 7.58E-01 | -6.37 |
| PDE9A | 0.046 | 6.973 | 0.474 | 6.35E-01 | 7.58E-01 | -6.371 |
| KRTCAP3 | -0.051 | 6.748 | -0.475 | 6.36E-01 | 7.58E-01 | -6.371 |
| RPS6KA1 | 0.052 | 7.69 | 0.474 | 6.36E-01 | 7.58E-01 | -6.371 |
| CCL8 | 0.113 | 6.537 | 0.473 | 6.36E-01 | 7.58E-01 | -6.371 |
| GPR107 | 0.043 | 8.166 | 0.472 | 6.37E-01 | 7.59E-01 | -6.372 |
| CD83 | 0.065 | 7.668 | 0.471 | 6.38E-01 | 7.60E-01 | -6.372 |
| RAB8A | -0.041 | 10.372 | -0.472 | 6.38E-01 | 7.60E-01 | -6.372 |
| SCGB1A1 | 0.087 | 6.651 | 0.471 | 6.38E-01 | 7.60E-01 | -6.372 |
| CD37 | 0.057 | 7.416 | 0.47 | 6.38E-01 | 7.61E-01 | -6.373 |
| ERBB2 | 0.043 | 8.86 | 0.47 | 6.38E-01 | 7.61E-01 | -6.373 |
| RHOC | 0.037 | 11.428 | 0.469 | 6.39E-01 | 7.61E-01 | -6.373 |
| C1QTNF7 | -0.059 | 5.865 | -0.469 | 6.39E-01 | 7.61E-01 | -6.373 |
| CENPF | 0.053 | 7.771 | 0.468 | 6.40E-01 | 7.62E-01 | -6.374 |
| AQP3 | 0.067 | 7.095 | 0.468 | 6.40E-01 | 7.62E-01 | -6.374 |
| PDPR | -0.056 | 7.46 | -0.469 | 6.40E-01 | 7.62E-01 | -6.374 |
| GNAZ | -0.046 | 6.735 | -0.467 | 6.41E-01 | 7.63E-01 | -6.374 |
| TMEM26 | 0.051 | 5.219 | 0.466 | 6.41E-01 | 7.63E-01 | -6.375 |
| ENTPD1 | 0.066 | 8.278 | 0.465 | 6.42E-01 | 7.63E-01 | -6.375 |
| GLO1 | -0.041 | 9.779 | -0.465 | 6.42E-01 | 7.64E-01 | -6.375 |
| BNIPL | 0.06 | 5.54 | 0.464 | 6.42E-01 | 7.64E-01 | -6.375 |
| SSTR4 | -0.059 | 5.31 | -0.465 | 6.42E-01 | 7.64E-01 | -6.375 |
| PYGB | 0.045 | 8.887 | 0.464 | 6.43E-01 | 7.64E-01 | -6.375 |
| LPO | -0.064 | 5.09 | -0.464 | 6.43E-01 | 7.65E-01 | -6.376 |
| ZDHHC6 | -0.04 | 8.565 | -0.463 | 6.44E-01 | 7.65E-01 | -6.376 |
| ADCY2 | 0.053 | 5.613 | 0.461 | 6.44E-01 | 7.66E-01 | -6.377 |
| ABHD10 | 0.039 | 7.045 | 0.461 | 6.44E-01 | 7.66E-01 | -6.377 |
| CYP11B2 | 0.055 | 5.585 | 0.461 | 6.44E-01 | 7.66E-01 | -6.377 |
| TAS2R1 | 0.043 | 4.876 | 0.459 | 6.46E-01 | 7.67E-01 | -6.378 |
| TXNL4B | 0.038 | 6.799 | 0.459 | 6.46E-01 | 7.67E-01 | -6.378 |
| CTNNA2 | -0.046 | 4.845 | -0.46 | 6.46E-01 | 7.67E-01 | -6.378 |
| CRYBA2 | 0.055 | 6.082 | 0.457 | 6.47E-01 | 7.68E-01 | -6.378 |
| CSRP2 | -0.059 | 9.746 | -0.457 | 6.48E-01 | 7.69E-01 | -6.379 |
| PPP2R3A | -0.041 | 7.538 | -0.457 | 6.48E-01 | 7.69E-01 | -6.379 |
| ADIPOQ | 0.051 | 4.937 | 0.456 | 6.48E-01 | 7.69E-01 | -6.379 |
| ZNF543 | -0.046 | 6.356 | -0.456 | 6.49E-01 | 7.70E-01 | -6.379 |
| NDST2 | 0.027 | 7.922 | 0.454 | 6.50E-01 | 7.70E-01 | -6.38 |
| MTIF2 | -0.041 | 7.509 | -0.455 | 6.50E-01 | 7.70E-01 | -6.38 |
| GNPTG | -0.039 | 10.417 | -0.455 | 6.50E-01 | 7.70E-01 | -6.38 |
| NUP88 | 0.031 | 9.716 | 0.454 | 6.50E-01 | 7.70E-01 | -6.38 |
| TRAP1 | 0.036 | 8.938 | 0.453 | 6.50E-01 | 7.71E-01 | -6.38 |
| BASP1 | 0.047 | 13.111 | 0.453 | 6.50E-01 | 7.71E-01 | -6.38 |
| GDAP1L1 | 0.06 | 4.851 | 0.453 | 6.50E-01 | 7.71E-01 | -6.38 |
| SMARCA4 | -0.031 | 10.823 | -0.453 | 6.51E-01 | 7.71E-01 | -6.381 |
| CDC27 | -0.034 | 7.774 | -0.453 | 6.51E-01 | 7.71E-01 | -6.381 |
| RRM1 | -0.039 | 10.422 | -0.452 | 6.52E-01 | 7.72E-01 | -6.381 |
| RIPK1 | 0.036 | 8.763 | 0.451 | 6.52E-01 | 7.72E-01 | -6.381 |
| RASIP1 | 0.056 | 9.195 | 0.451 | 6.52E-01 | 7.72E-01 | -6.381 |
| TSNAXIP1 | 0.052 | 5.533 | 0.45 | 6.52E-01 | 7.72E-01 | -6.382 |
| PANK3 | -0.041 | 7.373 | -0.451 | 6.52E-01 | 7.72E-01 | -6.382 |
| ZNF174 | 0.033 | 7.061 | 0.45 | 6.53E-01 | 7.72E-01 | -6.382 |
| WDR12 | -0.033 | 8.951 | -0.45 | 6.53E-01 | 7.73E-01 | -6.382 |
| STIP1 | -0.049 | 9.575 | -0.449 | 6.54E-01 | 7.73E-01 | -6.382 |
| KCTD15 | 0.041 | 7.042 | 0.448 | 6.54E-01 | 7.74E-01 | -6.383 |
| NEU2 | 0.05 | 5.008 | 0.447 | 6.55E-01 | 7.74E-01 | -6.383 |
| TMEM14C | -0.035 | 11.609 | -0.448 | 6.55E-01 | 7.74E-01 | -6.383 |
| L3MBTL4 | -0.039 | 4.855 | -0.447 | 6.55E-01 | 7.74E-01 | -6.383 |
| BST1 | -0.046 | 8.087 | -0.447 | 6.55E-01 | 7.75E-01 | -6.383 |
| PPP1R7 | -0.032 | 9.749 | -0.447 | 6.55E-01 | 7.75E-01 | -6.384 |
| CYP11B1 | 0.045 | 5.62 | 0.445 | 6.56E-01 | 7.75E-01 | -6.384 |
| LGALS2 | -0.07 | 6.723 | -0.446 | 6.56E-01 | 7.75E-01 | -6.384 |
| IGSF11 | -0.048 | 4.982 | -0.445 | 6.56E-01 | 7.75E-01 | -6.384 |
| PSMB9 | -0.066 | 9.067 | -0.445 | 6.56E-01 | 7.75E-01 | -6.384 |
| AURKC | 0.059 | 5.706 | 0.444 | 6.57E-01 | 7.75E-01 | -6.384 |
| PRKCZ | -0.047 | 9.689 | -0.445 | 6.57E-01 | 7.75E-01 | -6.384 |
| MLLT6 | -0.05 | 8.025 | -0.444 | 6.58E-01 | 7.76E-01 | -6.385 |
| EN1 | 0.054 | 4.813 | 0.443 | 6.58E-01 | 7.76E-01 | -6.385 |
| RAD9B | -0.046 | 4.89 | -0.444 | 6.58E-01 | 7.76E-01 | -6.385 |
| COL11A1 | 0.051 | 5.76 | 0.442 | 6.58E-01 | 7.76E-01 | -6.385 |
| MAP3K14 | 0.051 | 7.634 | 0.442 | 6.58E-01 | 7.76E-01 | -6.385 |
| SNRPD2 | -0.043 | 11.294 | -0.441 | 6.60E-01 | 7.78E-01 | -6.386 |
| ABCA13 | -0.074 | 5.605 | -0.441 | 6.60E-01 | 7.78E-01 | -6.386 |
| GALR3 | 0.079 | 8.977 | 0.439 | 6.60E-01 | 7.79E-01 | -6.387 |
| HOXC10 | -0.047 | 5.149 | -0.439 | 6.61E-01 | 7.79E-01 | -6.387 |
| RBMX2 | -0.038 | 7.824 | -0.439 | 6.61E-01 | 7.79E-01 | -6.387 |
| NDRG3 | -0.042 | 8.339 | -0.439 | 6.61E-01 | 7.79E-01 | -6.387 |
| CXADR | -0.056 | 5.152 | -0.438 | 6.62E-01 | 7.80E-01 | -6.387 |
| SMARCD2 | 0.046 | 9.235 | 0.436 | 6.62E-01 | 7.80E-01 | -6.388 |
| LRPPRC | 0.037 | 8.655 | 0.436 | 6.62E-01 | 7.80E-01 | -6.388 |
| KRTAP4-4 | 0.041 | 4.762 | 0.436 | 6.63E-01 | 7.80E-01 | -6.388 |
| DPPA2 | 0.042 | 5.614 | 0.435 | 6.63E-01 | 7.81E-01 | -6.388 |
| NOL3 | 0.044 | 8.411 | 0.435 | 6.63E-01 | 7.81E-01 | -6.388 |
| FTSJ2 | 0.028 | 8.253 | 0.435 | 6.63E-01 | 7.81E-01 | -6.388 |
| CPXM2 | -0.122 | 7.596 | -0.436 | 6.64E-01 | 7.81E-01 | -6.388 |
| PANX3 | 0.052 | 5.996 | 0.434 | 6.64E-01 | 7.81E-01 | -6.388 |
| EDA2R | 0.064 | 5.371 | 0.434 | 6.64E-01 | 7.81E-01 | -6.389 |
| GPR61 | 0.044 | 5.008 | 0.433 | 6.64E-01 | 7.81E-01 | -6.389 |
| SERAC1 | -0.057 | 5.748 | -0.433 | 6.65E-01 | 7.82E-01 | -6.389 |
| HABP4 | 0.038 | 7.669 | 0.431 | 6.66E-01 | 7.84E-01 | -6.39 |
| HIST1H2BK | -0.043 | 11.372 | -0.431 | 6.67E-01 | 7.84E-01 | -6.39 |
| CYP26B1 | 0.07 | 6.421 | 0.43 | 6.67E-01 | 7.84E-01 | -6.39 |
| CROCC | 0.035 | 6.613 | 0.43 | 6.67E-01 | 7.84E-01 | -6.39 |
| SMPD1 | -0.043 | 8.51 | -0.431 | 6.67E-01 | 7.84E-01 | -6.39 |
| CACNA1I | 0.042 | 6.581 | 0.429 | 6.68E-01 | 7.84E-01 | -6.391 |
| SLC15A4 | 0.035 | 8.71 | 0.429 | 6.68E-01 | 7.84E-01 | -6.391 |
| CNTN2 | 0.046 | 5.072 | 0.428 | 6.68E-01 | 7.84E-01 | -6.391 |
| SLC12A1 | -0.047 | 4.943 | -0.429 | 6.68E-01 | 7.84E-01 | -6.391 |
| SFRP5 | 0.049 | 4.795 | 0.428 | 6.68E-01 | 7.84E-01 | -6.391 |
| KLHL6 | -0.041 | 6.717 | -0.429 | 6.68E-01 | 7.84E-01 | -6.391 |
| RAP1GDS1 | -0.042 | 7.914 | -0.429 | 6.69E-01 | 7.85E-01 | -6.391 |
| ADAM8 | 0.056 | 8.348 | 0.427 | 6.69E-01 | 7.85E-01 | -6.391 |
| RBM15 | -0.04 | 7.521 | -0.428 | 6.69E-01 | 7.85E-01 | -6.391 |
| NRL | 0.053 | 5.977 | 0.427 | 6.69E-01 | 7.85E-01 | -6.392 |
| AKAP4 | -0.043 | 4.953 | -0.428 | 6.69E-01 | 7.85E-01 | -6.392 |
| ELOVL4 | 0.062 | 6.549 | 0.426 | 6.70E-01 | 7.85E-01 | -6.392 |
| FGF6 | 0.045 | 5.376 | 0.426 | 6.70E-01 | 7.85E-01 | -6.392 |
| PROS1 | -0.056 | 8.126 | -0.426 | 6.71E-01 | 7.86E-01 | -6.392 |
| PDGFD | -0.084 | 6.967 | -0.426 | 6.71E-01 | 7.86E-01 | -6.393 |
| SF3B4 | 0.068 | 8.299 | 0.425 | 6.71E-01 | 7.86E-01 | -6.393 |
| PARP10 | -0.039 | 8.288 | -0.425 | 6.72E-01 | 7.87E-01 | -6.393 |
| LDHA | -0.051 | 12.376 | -0.424 | 6.72E-01 | 7.87E-01 | -6.393 |
| IL1B | -0.139 | 8.548 | -0.424 | 6.72E-01 | 7.87E-01 | -6.393 |
| SCN4A | 0.057 | 5.614 | 0.423 | 6.72E-01 | 7.87E-01 | -6.393 |
| IFIT3 | -0.072 | 9.169 | -0.424 | 6.72E-01 | 7.87E-01 | -6.393 |
| NELL1 | 0.04 | 5.19 | 0.423 | 6.72E-01 | 7.87E-01 | -6.393 |
| GNGT2 | 0.047 | 6.051 | 0.422 | 6.72E-01 | 7.87E-01 | -6.394 |
| HHLA1 | 0.053 | 5.294 | 0.422 | 6.73E-01 | 7.87E-01 | -6.394 |
| SLC17A1 | 0.049 | 4.848 | 0.422 | 6.73E-01 | 7.87E-01 | -6.394 |
| TGM1 | -0.057 | 6.205 | -0.422 | 6.73E-01 | 7.88E-01 | -6.394 |
| AMPD1 | 0.045 | 4.737 | 0.421 | 6.73E-01 | 7.88E-01 | -6.394 |
| LHX1 | 0.058 | 6.107 | 0.421 | 6.74E-01 | 7.88E-01 | -6.394 |
| CCIN | 0.053 | 5.934 | 0.42 | 6.74E-01 | 7.88E-01 | -6.394 |
| FABP7 | -0.064 | 5.063 | -0.421 | 6.74E-01 | 7.88E-01 | -6.394 |
| MAD2L1BP | -0.029 | 9.308 | -0.421 | 6.74E-01 | 7.88E-01 | -6.395 |
| GABRB3 | -0.048 | 4.935 | -0.42 | 6.75E-01 | 7.88E-01 | -6.395 |
| AGMAT | -0.049 | 5.897 | -0.419 | 6.76E-01 | 7.89E-01 | -6.395 |
| TEX101 | 0.057 | 5.388 | 0.418 | 6.76E-01 | 7.89E-01 | -6.395 |
| CTH | 0.045 | 6.463 | 0.418 | 6.76E-01 | 7.89E-01 | -6.395 |
| TRIM63 | -0.068 | 5.801 | -0.419 | 6.76E-01 | 7.89E-01 | -6.395 |
| C19orf18 | 0.046 | 6.498 | 0.418 | 6.76E-01 | 7.89E-01 | -6.396 |
| FANCC | 0.035 | 7.103 | 0.417 | 6.76E-01 | 7.89E-01 | -6.396 |
| ARHGAP26 | -0.046 | 7.997 | -0.418 | 6.76E-01 | 7.89E-01 | -6.396 |
| UBQLN3 | 0.052 | 5.716 | 0.416 | 6.77E-01 | 7.90E-01 | -6.396 |
| IL4 | -0.05 | 4.822 | -0.417 | 6.77E-01 | 7.90E-01 | -6.396 |
| C10orf82 | 0.05 | 5.378 | 0.416 | 6.77E-01 | 7.90E-01 | -6.396 |
| UBAP1 | 0.031 | 9.937 | 0.415 | 6.77E-01 | 7.90E-01 | -6.396 |
| COL9A3 | -0.072 | 7.174 | -0.416 | 6.78E-01 | 7.90E-01 | -6.396 |
| ZBTB5 | 0.031 | 8.758 | 0.415 | 6.78E-01 | 7.90E-01 | -6.396 |
| MAGEE2 | -0.05 | 5.178 | -0.415 | 6.79E-01 | 7.91E-01 | -6.397 |
| SMAD1 | -0.041 | 7.778 | -0.415 | 6.79E-01 | 7.91E-01 | -6.397 |
| CDYL2 | -0.045 | 7.276 | -0.414 | 6.79E-01 | 7.91E-01 | -6.397 |
| TAS2R38 | -0.044 | 4.766 | -0.413 | 6.80E-01 | 7.92E-01 | -6.398 |
| NACA | -0.031 | 12.068 | -0.413 | 6.80E-01 | 7.93E-01 | -6.398 |
| ERCC3 | 0.03 | 8.846 | 0.412 | 6.80E-01 | 7.93E-01 | -6.398 |
| KLK1 | -0.057 | 5.56 | -0.412 | 6.81E-01 | 7.93E-01 | -6.398 |
| RHBDL2 | -0.048 | 5.289 | -0.412 | 6.81E-01 | 7.93E-01 | -6.398 |
| ALS2CR12 | 0.041 | 5.207 | 0.411 | 6.81E-01 | 7.93E-01 | -6.398 |
| NPFF | 0.037 | 7.445 | 0.411 | 6.81E-01 | 7.93E-01 | -6.398 |
| PRKD2 | 0.038 | 9.103 | 0.409 | 6.83E-01 | 7.94E-01 | -6.399 |
| DNAJB12 | 0.03 | 8.917 | 0.408 | 6.83E-01 | 7.94E-01 | -6.399 |
| PINK1 | -0.04 | 10.33 | -0.409 | 6.83E-01 | 7.94E-01 | -6.399 |
| COX7B2 | 0.092 | 5.469 | 0.408 | 6.83E-01 | 7.94E-01 | -6.399 |
| GLT8D2 | 0.04 | 6.588 | 0.408 | 6.83E-01 | 7.94E-01 | -6.399 |
| FNDC5 | -0.054 | 5.18 | -0.408 | 6.83E-01 | 7.95E-01 | -6.4 |
| KIF12 | -0.057 | 5.054 | -0.408 | 6.84E-01 | 7.95E-01 | -6.4 |
| SPEN | -0.047 | 9.412 | -0.407 | 6.85E-01 | 7.96E-01 | -6.4 |
| NISCH | 0.041 | 10.012 | 0.404 | 6.86E-01 | 7.97E-01 | -6.401 |
| RNF157 | 0.046 | 5.657 | 0.404 | 6.86E-01 | 7.97E-01 | -6.401 |
| CYP24A1 | -0.054 | 5.593 | -0.405 | 6.86E-01 | 7.97E-01 | -6.401 |
| PC | -0.044 | 7.758 | -0.404 | 6.86E-01 | 7.97E-01 | -6.401 |
| HIST1H1C | -0.065 | 11.54 | -0.404 | 6.87E-01 | 7.97E-01 | -6.401 |
| ARHGAP24 | -0.045 | 7.024 | -0.404 | 6.87E-01 | 7.97E-01 | -6.401 |
| ZNF195 | -0.05 | 8.018 | -0.404 | 6.87E-01 | 7.97E-01 | -6.401 |
| PAX3 | 0.036 | 4.987 | 0.402 | 6.88E-01 | 7.98E-01 | -6.402 |
| DFNA5 | -0.057 | 9.393 | -0.403 | 6.88E-01 | 7.98E-01 | -6.402 |
| PCCB | 0.035 | 9.357 | 0.401 | 6.88E-01 | 7.99E-01 | -6.402 |
| LOX | 0.064 | 7.11 | 0.401 | 6.88E-01 | 7.99E-01 | -6.402 |
| CYP4Z1 | 0.04 | 4.75 | 0.4 | 6.88E-01 | 7.99E-01 | -6.402 |
| HEBP1 | 0.039 | 10.798 | 0.4 | 6.89E-01 | 7.99E-01 | -6.402 |
| C19orf12 | 0.025 | 8.572 | 0.4 | 6.89E-01 | 7.99E-01 | -6.402 |
| UCHL1 | 0.092 | 8.079 | 0.4 | 6.89E-01 | 7.99E-01 | -6.403 |
| KLF12 | -0.042 | 6.625 | -0.401 | 6.89E-01 | 7.99E-01 | -6.403 |
| GUCY1B2 | 0.06 | 5.874 | 0.4 | 6.89E-01 | 7.99E-01 | -6.403 |
| IKBKB | -0.038 | 7.585 | -0.4 | 6.90E-01 | 7.99E-01 | -6.403 |
| BIK | 0.05 | 5.846 | 0.399 | 6.90E-01 | 7.99E-01 | -6.403 |
| KATNAL1 | -0.04 | 7.216 | -0.4 | 6.90E-01 | 7.99E-01 | -6.403 |
| BMP8A | -0.055 | 5.506 | -0.399 | 6.90E-01 | 7.99E-01 | -6.403 |
| SLAMF8 | 0.059 | 7.262 | 0.398 | 6.90E-01 | 8.00E-01 | -6.403 |
| LECT1 | 0.044 | 5.551 | 0.398 | 6.91E-01 | 8.00E-01 | -6.404 |
| SIN3A | 0.028 | 9.422 | 0.397 | 6.91E-01 | 8.00E-01 | -6.404 |
| UBP1 | -0.038 | 9.26 | -0.398 | 6.91E-01 | 8.00E-01 | -6.404 |
| POLR2A | 0.047 | 9.964 | 0.396 | 6.92E-01 | 8.01E-01 | -6.404 |
| AMFR | 0.049 | 9.788 | 0.395 | 6.92E-01 | 8.01E-01 | -6.404 |
| SPTBN1 | 0.044 | 7.664 | 0.395 | 6.92E-01 | 8.01E-01 | -6.404 |
| JAM3 | 0.048 | 9.56 | 0.395 | 6.93E-01 | 8.01E-01 | -6.405 |
| GNAS | -0.027 | 10.242 | -0.396 | 6.93E-01 | 8.01E-01 | -6.405 |
| RARRES1 | -0.059 | 6.413 | -0.396 | 6.93E-01 | 8.01E-01 | -6.405 |
| KCNA6 | -0.052 | 5.207 | -0.396 | 6.93E-01 | 8.01E-01 | -6.405 |
| RNF4 | -0.047 | 10.012 | -0.395 | 6.93E-01 | 8.01E-01 | -6.405 |
| PACSIN1 | 0.048 | 6.056 | 0.394 | 6.93E-01 | 8.01E-01 | -6.405 |
| RBM24 | 0.05 | 5.698 | 0.394 | 6.93E-01 | 8.01E-01 | -6.405 |
| RUNDC1 | -0.039 | 8.057 | -0.394 | 6.94E-01 | 8.02E-01 | -6.405 |
| ZNF134 | -0.039 | 7.087 | -0.394 | 6.94E-01 | 8.02E-01 | -6.405 |
| LHFP | 0.044 | 10.249 | 0.393 | 6.94E-01 | 8.02E-01 | -6.405 |
| IGF1 | 0.049 | 7.459 | 0.391 | 6.95E-01 | 8.03E-01 | -6.406 |
| SRGAP3 | -0.045 | 5.142 | -0.392 | 6.96E-01 | 8.03E-01 | -6.406 |
| NASP | -0.031 | 8.978 | -0.392 | 6.96E-01 | 8.03E-01 | -6.406 |
| TRIM54 | 0.038 | 5.231 | 0.39 | 6.96E-01 | 8.03E-01 | -6.406 |
| PSMC1 | -0.031 | 11.7 | -0.391 | 6.96E-01 | 8.03E-01 | -6.406 |
| ZNF502 | -0.04 | 7.585 | -0.391 | 6.96E-01 | 8.03E-01 | -6.406 |
| BRIP1 | 0.038 | 5.207 | 0.39 | 6.96E-01 | 8.03E-01 | -6.406 |
| WDR53 | 0.027 | 7.98 | 0.39 | 6.96E-01 | 8.03E-01 | -6.406 |
| XDH | 0.075 | 6.204 | 0.39 | 6.96E-01 | 8.03E-01 | -6.407 |
| RTP1 | 0.039 | 5.044 | 0.39 | 6.97E-01 | 8.03E-01 | -6.407 |
| DLGAP1 | -0.041 | 4.958 | -0.39 | 6.97E-01 | 8.03E-01 | -6.407 |
| HLA-DMB | -0.055 | 8.738 | -0.39 | 6.97E-01 | 8.03E-01 | -6.407 |
| PRDX1 | -0.029 | 13.003 | -0.39 | 6.97E-01 | 8.03E-01 | -6.407 |
| BTN1A1 | 0.044 | 4.864 | 0.388 | 6.97E-01 | 8.04E-01 | -6.407 |
| GNG3 | -0.042 | 4.934 | -0.389 | 6.98E-01 | 8.04E-01 | -6.407 |
| CYP4X1 | 0.05 | 5.846 | 0.388 | 6.98E-01 | 8.04E-01 | -6.407 |
| ADCY1 | -0.056 | 5.276 | -0.389 | 6.98E-01 | 8.04E-01 | -6.407 |
| CSRP3 | 0.031 | 4.429 | 0.387 | 6.98E-01 | 8.04E-01 | -6.407 |
| LRPAP1 | 0.034 | 10.39 | 0.387 | 6.98E-01 | 8.04E-01 | -6.407 |
| FLT3LG | 0.043 | 6.753 | 0.387 | 6.98E-01 | 8.04E-01 | -6.408 |
| LPP | 0.04 | 8.654 | 0.386 | 6.99E-01 | 8.05E-01 | -6.408 |
| VN1R4 | -0.037 | 4.996 | -0.387 | 6.99E-01 | 8.05E-01 | -6.408 |
| CHD9 | -0.046 | 8.281 | -0.387 | 6.99E-01 | 8.05E-01 | -6.408 |
| S100A8 | -0.063 | 9.917 | -0.385 | 7.00E-01 | 8.06E-01 | -6.409 |
| GRIA4 | 0.05 | 5.135 | 0.384 | 7.00E-01 | 8.06E-01 | -6.409 |
| AIP | 0.026 | 9.93 | 0.384 | 7.00E-01 | 8.06E-01 | -6.409 |
| PAX6 | -0.05 | 4.898 | -0.385 | 7.01E-01 | 8.06E-01 | -6.409 |
| FBXO2 | -0.073 | 6.632 | -0.384 | 7.01E-01 | 8.06E-01 | -6.409 |
| DAB1 | 0.046 | 6.518 | 0.383 | 7.01E-01 | 8.06E-01 | -6.409 |
| LRRN3 | -0.06 | 6.892 | -0.384 | 7.02E-01 | 8.07E-01 | -6.409 |
| TUB | 0.043 | 6.794 | 0.382 | 7.02E-01 | 8.07E-01 | -6.409 |
| CDON | -0.038 | 5.66 | -0.382 | 7.03E-01 | 8.08E-01 | -6.41 |
| ARID1A | -0.03 | 8.835 | -0.38 | 7.04E-01 | 8.09E-01 | -6.41 |
| IRS1 | 0.042 | 7.51 | 0.379 | 7.04E-01 | 8.09E-01 | -6.411 |
| DAAM1 | -0.041 | 8.243 | -0.379 | 7.05E-01 | 8.10E-01 | -6.411 |
| IRAK1 | 0.055 | 9.475 | 0.378 | 7.05E-01 | 8.10E-01 | -6.411 |
| SCNN1G | 0.035 | 5.062 | 0.377 | 7.06E-01 | 8.10E-01 | -6.411 |
| GCKR | 0.034 | 4.877 | 0.376 | 7.06E-01 | 8.11E-01 | -6.412 |
| ACRV1 | -0.037 | 5.087 | -0.376 | 7.07E-01 | 8.11E-01 | -6.412 |
| GPR132 | -0.042 | 6.183 | -0.376 | 7.07E-01 | 8.11E-01 | -6.412 |
| RTN3 | 0.026 | 9.217 | 0.375 | 7.07E-01 | 8.11E-01 | -6.412 |
| TCF7L2 | 0.046 | 7.163 | 0.375 | 7.08E-01 | 8.12E-01 | -6.412 |
| PAK1 | -0.051 | 9.427 | -0.375 | 7.08E-01 | 8.12E-01 | -6.412 |
| CBLL1 | 0.028 | 8.064 | 0.374 | 7.08E-01 | 8.12E-01 | -6.412 |
| CACNA1D | 0.049 | 5.152 | 0.374 | 7.08E-01 | 8.12E-01 | -6.412 |
| SLC2A3 | -0.056 | 9.022 | -0.374 | 7.08E-01 | 8.12E-01 | -6.413 |
| NCOR1 | -0.032 | 7.949 | -0.374 | 7.09E-01 | 8.12E-01 | -6.413 |
| SNX1 | -0.045 | 8.929 | -0.374 | 7.09E-01 | 8.12E-01 | -6.413 |
| RAD51C | 0.028 | 8.504 | 0.373 | 7.09E-01 | 8.12E-01 | -6.413 |
| HOXB2 | 0.046 | 7.951 | 0.372 | 7.09E-01 | 8.12E-01 | -6.413 |
| AVPR1B | -0.057 | 4.931 | -0.373 | 7.09E-01 | 8.13E-01 | -6.413 |
| TREML2 | -0.071 | 9.125 | -0.373 | 7.09E-01 | 8.13E-01 | -6.413 |
| WBSCR28 | -0.041 | 4.883 | -0.373 | 7.10E-01 | 8.13E-01 | -6.413 |
| PKD2L1 | 0.048 | 5.569 | 0.372 | 7.10E-01 | 8.13E-01 | -6.413 |
| SYP | 0.045 | 4.876 | 0.371 | 7.10E-01 | 8.13E-01 | -6.413 |
| ANKRD30A | 0.041 | 4.806 | 0.371 | 7.10E-01 | 8.13E-01 | -6.414 |
| SPAG6 | -0.039 | 4.854 | -0.372 | 7.10E-01 | 8.13E-01 | -6.414 |
| SLC14A1 | 0.054 | 6.251 | 0.371 | 7.10E-01 | 8.13E-01 | -6.414 |
| ATP8A2 | -0.053 | 5.725 | -0.371 | 7.11E-01 | 8.13E-01 | -6.414 |
| KCNJ15 | 0.039 | 6.981 | 0.369 | 7.12E-01 | 8.14E-01 | -6.414 |
| STARD7 | 0.027 | 10.155 | 0.369 | 7.12E-01 | 8.14E-01 | -6.414 |
| CTRL | -0.043 | 6.186 | -0.369 | 7.12E-01 | 8.14E-01 | -6.415 |
| CIDEA | -0.051 | 5.418 | -0.369 | 7.12E-01 | 8.14E-01 | -6.415 |
| PDLIM1 | -0.042 | 10.481 | -0.369 | 7.12E-01 | 8.14E-01 | -6.415 |
| NPTX2 | 0.06 | 4.891 | 0.368 | 7.12E-01 | 8.14E-01 | -6.415 |
| KIF3B | 0.029 | 8.24 | 0.368 | 7.13E-01 | 8.14E-01 | -6.415 |
| LY6D | -0.06 | 6.647 | -0.369 | 7.13E-01 | 8.14E-01 | -6.415 |
| CLEC4F | -0.049 | 5.112 | -0.369 | 7.13E-01 | 8.14E-01 | -6.415 |
| HTR2C | 0.047 | 4.908 | 0.367 | 7.13E-01 | 8.14E-01 | -6.415 |
| EPHA3 | -0.037 | 5.658 | -0.368 | 7.13E-01 | 8.14E-01 | -6.415 |
| PFDN5 | 0.038 | 12.467 | 0.367 | 7.14E-01 | 8.14E-01 | -6.415 |
| CHRNA6 | 0.038 | 5.642 | 0.366 | 7.14E-01 | 8.14E-01 | -6.415 |
| CHRNA10 | -0.04 | 6.243 | -0.367 | 7.14E-01 | 8.14E-01 | -6.415 |
| DUSP6 | 0.045 | 9.73 | 0.366 | 7.14E-01 | 8.15E-01 | -6.415 |
| CEBPB | -0.036 | 11.946 | -0.367 | 7.14E-01 | 8.15E-01 | -6.415 |
| NKX6-2 | 0.036 | 5.078 | 0.365 | 7.15E-01 | 8.15E-01 | -6.416 |
[truncated: 56,371 more chars]
