# Supplementary material for: Identifying molecular subgroups of patients with preeclampsia through bioinformatics
Source: Front Cardiovasc Med. 2024 Jun 3;11:1367578. doi: 10.3389/fcvm.2024.1367578 (PMC11180819; doi:10.3389/fcvm.2024.1367578)
Supplement: Supplementary file 5 [file Table5.docx]

**Supplementary Table S5.** Specific upregulation of differentially expressed genes in subgroups I and II

| Gene | subtype |
| --- | --- |
| IQGAP1 | I |
| USP38 | I |
| ACTR3 | I |
| RB1CC1 | I |
| NMD3 | I |
| PPP1R15B | I |
| VPS4B | I |
| RANBP6 | I |
| CLTC | I |
| CTSO | I |
| EEF1A1 | I |
| SLC33A1 | I |
| SPG20 | I |
| BTAF1 | I |
| FYTTD1 | I |
| OSBPL11 | I |
| CAPZA1 | I |
| NT5C2 | I |
| DHX15 | I |
| PHF3 | I |
| PPP1R12A | I |
| SEC24D | I |
| FNDC3A | I |
| ZNF140 | I |
| SCOC | I |
| PPM1D | I |
| ETNK1 | I |
| RAB18 | I |
| SLC35A5 | I |
| ELF1 | I |
| SNX14 | I |
| DEK | I |
| GOLPH3 | I |
| ANKRD10 | I |
| STAG1 | I |
| SNAP23 | I |
| RBM7 | I |
| UBE4A | I |
| ACBD3 | I |
| EIF2AK3 | I |
| ADNP | I |
| GMFB | I |
| ARID4A | I |
| RASA1 | I |
| NCK1 | I |
| CNOT8 | I |
| ZNF600 | I |
| IDI1 | I |
| PTPN12 | I |
| CHD1 | I |
| COPS2 | I |
| DLD | I |
| PHTF2 | I |
| TLK1 | I |
| ACTR6 | I |
| PPP4R2 | I |
| ZNF644 | I |
| GPD2 | I |
| ATP2C1 | I |
| USP14 | I |
| PERP | I |
| ZNRF2 | I |
| UBE2V2 | I |
| SDHD | I |
| TRIT1 | I |
| AMD1 | I |
| MAPK6 | I |
| SP3 | I |
| RAP2C | I |
| LAPTM4A | I |
| PDCD4 | I |
| IL1R1 | I |
| GALNT3 | I |
| NIPBL | I |
| KBTBD7 | I |
| PLSCR1 | I |
| SCFD1 | I |
| ADSS | I |
| PPP1CB | I |
| STX7 | I |
| BNIP2 | I |
| ATP11C | I |
| YRDC | I |
| DR1 | I |
| TSN | I |
| RAB14 | I |
| ZNF226 | I |
| GDI2 | I |
| PRKRA | I |
| GNA13 | I |
| CCNG1 | I |
| DNAJB4 | I |
| CORO1C | I |
| ZFR | I |
| RAB10 | I |
| PAIP2 | I |
| GOLGA4 | I |
| SLC39A10 | I |
| FZD6 | I |
| ALG5 | I |
| MATR3 | I |
| ATP6V1C1 | I |
| XPO1 | I |
| C1orf27 | I |
| SLC36A4 | I |
| SNX13 | I |
| ABI1 | I |
| GNAI3 | I |
| SLC40A1 | I |
| SOAT1 | I |
| EEA1 | I |
| SCYL2 | I |
| PCMT1 | I |
| DCTN4 | I |
| KIAA0232 | I |
| FOXJ3 | I |
| SKIL | I |
| WRB | I |
| PPT1 | I |
| IMPA1 | I |
| USP1 | I |
| GCA | I |
| UHRF2 | I |
| RPS6KB1 | I |
| MTMR6 | I |
| CD2AP | I |
| MALT1 | I |
| PDE8A | I |
| RAB33B | I |
| ANXA3 | I |
| NARS | I |
| ZBTB11 | I |
| PSMD12 | I |
| SPCS2 | I |
| ATP6V1A | I |
| RHOA | I |
| YES1 | I |
| MNDA | I |
| SDCBP | I |
| NAP1L1 | I |
| WDR44 | I |
| GNB4 | I |
| JAK2 | I |
| IL1RAP | I |
| APAF1 | I |
| BAG5 | I |
| SLC38A6 | I |
| SH3YL1 | I |
| SLK | I |
| PGBD2 | I |
| RAB21 | I |
| C6orf203 | I |
| FCHO2 | I |
| DMXL1 | I |
| APPBP2 | I |
| KIF5B | I |
| CGGBP1 | I |
| RPL7 | I |
| CHSY1 | I |
| PSIP1 | I |
| KIAA0196 | I |
| ZNF75A | I |
| RNF20 | I |
| STK38 | I |
| ARL6IP5 | I |
| GOLGA5 | I |
| PTP4A2 | I |
| VBP1 | I |
| LIFR | I |
| RABGGTB | I |
| SLMAP | I |
| DPYD | I |
| KIAA0907 | I |
| HCFC2 | I |
| TMED5 | I |
| GNAI1 | I |
| UBE3C | I |
| SLC35A3 | I |
| MBD4 | I |
| PNRC2 | I |
| RPGR | I |
| CLEC2D | I |
| GMNN | I |
| NOL7 | I |
| RAB8B | I |
| SELT | I |
| TOMM20 | I |
| LSM1 | I |
| HSF2 | I |
| ASF1A | I |
| SMAD4 | I |
| TCERG1 | I |
| TMEM33 | I |
| GLRX | I |
| UBE2N | I |
| PDGFC | I |
| TXNL1 | I |
| TRIP12 | I |
| SYNJ1 | I |
| TOPBP1 | I |
| LASP1 | I |
| GGH | I |
| GADD45G | I |
| HMGN3 | I |
| RAB28 | I |
| WBP4 | I |
| EIF4G2 | I |
| STAG2 | I |
| NFE2L2 | I |
| PON2 | I |
| MBD2 | I |
| C6orf62 | I |
| SLC35B3 | I |
| VPS29 | I |
| GCNT1 | I |
| SOCS5 | I |
| KHDRBS1 | I |
| B3GNT5 | I |
| MBNL1 | I |
| PTBP2 | I |
| CHUK | I |
| P4HA1 | I |
| WAC | I |
| MRPL50 | I |
| XPA | I |
| PKN2 | I |
| CSNK1A1 | I |
| DNAJC10 | I |
| PHF14 | I |
| RANBP2 | I |
| RAB9A | I |
| BACH1 | I |
| GTPBP4 | I |
| PPP2CA | I |
| KPNA3 | I |
| CNOT7 | I |
| MCTS1 | I |
| ELL2 | I |
| ARID4B | I |
| KLHL2 | I |
| EVI5 | I |
| WDR5B | I |
| PHYH | I |
| NET1 | I |
| CPEB2 | I |
| ARRDC3 | I |
| DEGS1 | I |
| ZNF431 | I |
| CLK1 | I |
| XRN1 | I |
| CHCHD7 | I |
| CLK4 | I |
| MAP3K8 | I |
| PRKD3 | I |
| NGLY1 | I |
| KCTD18 | I |
| OCIAD1 | I |
| GAB1 | I |
| SAP30L | I |
| LEPROTL1 | I |
| PAPOLA | I |
| SMARCA5 | I |
| AZI2 | I |
| SIAH1 | I |
| EVI2A | I |
| IPP | I |
| COMMD2 | I |
| TRNT1 | I |
| HSPH1 | I |
| BTK | I |
| YEATS4 | I |
| MINPP1 | I |
| ADD3 | I |
| OSBPL2 | I |
| IL18 | I |
| ARPC3 | I |
| CASP3 | I |
| ECHDC1 | I |
| FDX1 | I |
| C1GALT1 | I |
| OFD1 | I |
| SRPK2 | I |
| RABGAP1L | I |
| MTHFD2 | I |
| ANXA1 | I |
| ANP32A | I |
| PLEKHF2 | I |
| ZNF529 | I |
| NPC2 | I |
| PDCD6 | I |
| NUP43 | I |
| SP4 | I |
| RBBP4 | I |
| RHOB | I |
| PAN3 | I |
| GALNT7 | I |
| RBM25 | I |
| HERC4 | I |
| YAP1 | I |
| CCNT2 | I |
| CHFR | I |
| TRAM1 | I |
| RAF1 | I |
| STARD3NL | I |
| ACVR1 | I |
| FAM49B | I |
| MAPKAP1 | I |
| TUBGCP3 | I |
| C18orf25 | I |
| AHR | I |
| CPD | I |
| PIGH | I |
| C21orf91 | I |
| DAPP1 | I |
| RAB11A | I |
| TRPS1 | I |
| ARMCX5 | I |
| SLC39A7 | I |
| CASP7 | I |
| JRKL | I |
| MTMR9 | I |
| MCFD2 | I |
| ZNF608 | I |
| CHML | I |
| PCF11 | I |
| AMMECR1 | I |
| PIGN | I |
| STRN3 | I |
| CAST | I |
| F11R | I |
| TPD52 | I |
| SLC24A1 | I |
| FBXO11 | I |
| PRPF18 | I |
| TLR1 | I |
| PGM2 | I |
| ATAD1 | I |
| RDH10 | I |
| MDM4 | I |
| NFIL3 | I |
| ZNF569 | I |
| AGL | I |
| PNN | I |
| HTATSF1 | I |
| OSGEPL1 | I |
| FGFR1OP2 | I |
| KIAA1468 | I |
| OAT | I |
| C14orf142 | I |
| SLC30A9 | I |
| GDPD1 | I |
| BUB3 | I |
| ACRC | I |
| TGDS | I |
| ZFP36L1 | I |
| ANKRD12 | I |
| HDHD2 | I |
| THBS3 | I |
| CEBPZ | I |
| TRIM37 | I |
| RAP2A | I |
| POLR3F | I |
| RNGTT | I |
| PDCD10 | I |
| EIF1AX | I |
| RNF168 | I |
| INPP5F | I |
| PSMD14 | I |
| EIF2S1 | I |
| CDC42SE2 | I |
| CAPN7 | I |
| DOCK8 | I |
| LYPLA1 | I |
| NDUFA5 | I |
| CDC16 | I |
| ZNF30 | I |
| DENR | I |
| ARMC1 | I |
| E2F3 | I |
| RPL27A | I |
| RNF13 | I |
| PPA2 | I |
| LMNB1 | I |
| COMMD10 | I |
| NSUN2 | I |
| ITGB1BP1 | I |
| FGD6 | I |
| COG6 | I |
| ZNF267 | I |
| OXR1 | I |
| PRKRIR | I |
| SYAP1 | I |
| PRPF39 | I |
| SUV39H2 | I |
| TCEAL8 | I |
| MAN2B2 | I |
| SERPINB1 | I |
| TTF1 | I |
| HN1 | I |
| RCHY1 | I |
| ITGB3BP | I |
| BCCIP | I |
| RNF38 | I |
| DUSP12 | I |
| BZW1 | I |
| PKD2 | I |
| SAP30 | I |
| SBNO1 | I |
| ETF1 | I |
| MSH2 | I |
| CREBL2 | I |
| ARL1 | I |
| CPVL | I |
| ZNF248 | I |
| H2AFV | I |
| NCOA4 | I |
| ANLN | I |
| FANCD2 | I |
| ARHGAP5 | I |
| ZNF227 | I |
| BCL6 | I |
| CCNL1 | I |
| THUMPD1 | I |
| DLG5 | I |
| SNX16 | I |
| NDEL1 | I |
| ADK | I |
| MYLIP | I |
| ARL6IP6 | I |
| TRIM52 | I |
| ABCA1 | I |
| HEBP2 | I |
| DONSON | I |
| NUDT5 | I |
| CARS | I |
| CITED2 | I |
| RDX | I |
| PHLDB2 | I |
| SESTD1 | I |
| EHHADH | I |
| LBR | I |
| PIGK | I |
| PDK1 | I |
| RDH11 | I |
| BIRC3 | I |
| SH3GLB1 | I |
| SMPDL3A | I |
| SNX2 | I |
| ETFA | I |
| NUDCD2 | I |
| WWP1 | I |
| FXR1 | I |
| TCEB1 | I |
| VPS54 | I |
| PTGER2 | I |
| MRPL47 | I |
| RHOBTB3 | I |
| ACTR2 | I |
| GTF2A1 | I |
| RHEB | I |
| ZNF639 | I |
| FAM3C | I |
| ITGAV | I |
| PRKAA1 | I |
| SLC22A15 | I |
| SEMA3C | I |
| ATP1B3 | I |
| HIRA | I |
| MAT2B | I |
| NIPA1 | I |
| FUNDC1 | I |
| IFI16 | I |
| ASAH1 | I |
| WSB1 | I |
| RNF14 | I |
| CREM | I |
| TRIM56 | I |
| HS2ST1 | I |
| CUL3 | I |
| TMF1 | I |
| SUZ12 | I |
| MLX | I |
| NKAP | I |
| ATPIF1 | I |
| ATF4 | I |
| JDP2 | I |
| PSTPIP2 | I |
| ZNF354B | I |
| FOS | I |
| SMARCA2 | I |
| DNM1L | I |
| USP49 | I |
| ZNF350 | I |
| BRWD3 | I |
| HMGCS1 | I |
| RAB1A | I |
| KIF21A | I |
| GKAP1 | I |
| ANP32C | I |
| DPYS | I |
| TAF1A | I |
| ADAM9 | I |
| CTBS | I |
| SLC27A4 | I |
| RBBP9 | I |
| PIGA | I |
| ZNF430 | I |
| RNASEH1 | I |
| TARDBP | I |
| ZNF214 | I |
| ABHD8 | I |
| INPP5A | I |
| MYNN | I |
| MBTD1 | I |
| NBEA | I |
| CRYZL1 | I |
| GTF3C3 | I |
| SLC23A1 | I |
| ATM | I |
| UBE2A | I |
| ZZZ3 | I |
| IFI44 | I |
| NAT2 | I |
| CCPG1 | I |
| TARSL2 | I |
| ZNF274 | I |
| PTEN | I |
| ZBTB33 | I |
| PIK3CB | I |
| INCENP | I |
| ZNF367 | I |
| RPS6KA5 | I |
| ZCCHC9 | I |
| LAMP1 | I |
| ZNF429 | I |
| DDX27 | I |
| MARCKS | I |
| SNAPC1 | I |
| PPIL3 | I |
| NR2C1 | I |
| GCN1L1 | I |
| ATP5F1 | I |
| FUT8 | I |
| EPHB4 | I |
| MPP6 | I |
| SFPQ | I |
| MANEA | I |
| UVRAG | I |
| TMEM39A | I |
| SRP9 | I |
| USP16 | I |
| SNAPC5 | I |
| HDAC4 | I |
| MRPL19 | I |
| GBP3 | I |
| DDX3X | I |
| IL18R1 | I |
| LACTB2 | I |
| GSPT1 | I |
| TAF1B | I |
| MAP4K5 | I |
| TRUB1 | I |
| PPFIA1 | I |
| B4GALT6 | I |
| INSIG2 | I |
| ATP5O | I |
| SPRY2 | I |
| ARF6 | I |
| CDH11 | I |
| PPIG | I |
| RUFY2 | I |
| TCF12 | I |
| IFIH1 | I |
| PPP5C | I |
| EGLN3 | I |
| SGTB | I |
| C1D | I |
| FBXO33 | I |
| CPE | I |
| SENP7 | I |
| GALNS | I |
| VDAC2 | I |
| NDUFB5 | I |
| NUPL2 | I |
| TWSG1 | I |
| DDX20 | I |
| ZNF43 | I |
| TANK | I |
| SLBP | I |
| UQCRC2 | I |
| RIOK3 | I |
| FKBP14 | I |
| ABCE1 | I |
| NKIRAS1 | I |
| DZIP3 | I |
| CCNG2 | I |
| IMMT | I |
| MKLN1 | I |
| ZNF415 | I |
| MBD1 | I |
| OSTM1 | I |
| ABCD3 | I |
| RAD18 | I |
| GTF2F2 | I |
| AP3B1 | I |
| ZNF468 | I |
| SRP72 | I |
| CACNB4 | I |
| CUL4A | I |
| ZNF510 | I |
| RNF139 | I |
| SF3B1 | I |
| CALD1 | I |
| CARD6 | I |
| DNM3 | I |
| VDAC1 | I |
| ITGAE | I |
| MRPL15 | I |
| NCF2 | I |
| THAP9 | I |
| RAB40B | I |
| TRAF5 | I |
| CD1D | I |
| EXOSC8 | I |
| KIT | I |
| ARFGAP3 | I |
| LRBA | I |
| BCL2L2 | I |
| TERF1 | I |
| CFDP1 | I |
| UBE2H | I |
| SS18L1 | I |
| CBR4 | I |
| C4BPB | I |
| DCP2 | I |
| C9orf72 | I |
| MTRF1L | I |
| TSPYL5 | I |
| PSMA2 | I |
| CTBP2 | I |
| PEX13 | I |
| SPRED1 | I |
| SEC24C | I |
| FBXO3 | I |
| RABEP1 | I |
| PRKAB2 | I |
| PDE4DIP | I |
| LIMK2 | I |
| ANGPTL1 | I |
| IDS | I |
| TIMP2 | I |
| CD59 | I |
| HOMER1 | I |
| UTRN | I |
| KIDINS220 | I |
| L3MBTL3 | I |
| PDHA1 | I |
| SHFM1 | I |
| HECTD1 | I |
| ERO1L | I |
| PIK3R4 | I |
| IER2 | I |
| CREB5 | I |
| EEF1E1 | I |
| RANBP3 | I |
| NSUN6 | I |
| TRIM17 | I |
| ZNF595 | I |
| TIMM17A | I |
| TIMP3 | I |
| IL10RB | I |
| PHACTR2 | I |
| RNF130 | I |
| ZBED4 | I |
| RBM22 | I |
| SMUG1 | I |
| C1orf43 | I |
| PACSIN2 | I |
| BCL10 | I |
| ZNF382 | I |
| ZDHHC23 | I |
| RSBN1 | I |
| SUMF2 | I |
| SENP2 | I |
| HIBCH | I |
| ZCCHC6 | I |
| COX7B | I |
| NIPSNAP3B | I |
| LMO4 | I |
| RPL3 | I |
| ZNF26 | I |
| POLE3 | I |
| CUL2 | I |
| CPNE8 | I |
| RPL14 | I |
| HPSE | I |
| UQCRB | I |
| NCOA2 | I |
| CENPJ | I |
| GNAQ | I |
| ITCH | I |
| GFM2 | I |
| STIM2 | I |
| SAMD8 | I |
| POLR2C | I |
| ZFYVE21 | I |
| JMJD1C | I |
| HIVEP2 | I |
| THUMPD3 | I |
| NMT2 | I |
| SNRK | I |
| NAB1 | I |
| SH3BGRL | I |
| SEC61B | I |
| GPR87 | I |
| MCEE | I |
| ANKRD1 | I |
| CNOT6 | I |
| LDHB | I |
| LPPR4 | I |
| UPF3A | I |
| DIRAS3 | I |
| TUBGCP5 | I |
| ACTN1 | I |
| MFAP1 | I |
| PTGS2 | I |
| TRIM44 | I |
| INPPL1 | I |
| SNX3 | I |
| PDCD6IP | I |
| CD58 | I |
| TMEM14B | I |
| TNFRSF12A | I |
| POLK | I |
| MAGEH1 | I |
| ZNF623 | I |
| PCNA | I |
| CPSF6 | I |
| STAT5B | I |
| ENTPD4 | I |
| CSTF3 | I |
| RP2 | I |
| DBR1 | I |
| ATP6V1G1 | I |
| TDRD3 | I |
| KIAA1586 | I |
| TIGD7 | I |
| SCN4B | I |
| SPIN3 | I |
| VCL | I |
| POSTN | I |
| RBM11 | I |
| NUP160 | I |
| NDUFS2 | I |
| KDELC1 | I |
| ZCCHC2 | I |
| ARHGAP21 | I |
| TMCC1 | I |
| SURF6 | I |
| ZNF559 | I |
| BBS7 | I |
| RASA2 | I |
| RFC5 | I |
| LIMD1 | I |
| CGRRF1 | I |
| UBE2J2 | I |
| KLF7 | I |
| MRPL44 | I |
| MTBP | I |
| CCT6B | I |
| GGA1 | I |
| GALNT12 | I |
| SLC19A2 | I |
| VAMP3 | I |
| PIK3AP1 | I |
| DSCR3 | I |
| MDH1 | I |
| GPR65 | I |
| ZNF614 | I |
| PSMC5 | I |
| SNRPE | I |
| MTO1 | I |
| NUDT15 | I |
| PIGF | I |
| NRIP1 | I |
| PLCB1 | I |
| UNG | I |
| TTC19 | I |
| GNL2 | I |
| PSAP | I |
| PKIA | I |
| NID2 | I |
| B2M | I |
| HECTD2 | I |
| SCP2 | I |
| ZNF555 | I |
| C1GALT1C1 | I |
| KRAS | I |
| XRCC4 | I |
| UCHL5 | I |
| GRTP1 | I |
| THUMPD2 | I |
| PHF10 | I |
| SYNJ2 | I |
| PRSS23 | I |
| PSMC6 | I |
| AGPAT4 | I |
| SERP1 | I |
| MAPRE1 | I |
| TTK | I |
| OPN3 | I |
| KIAA1715 | I |
| PPIF | I |
| TRIM36 | I |
| GABPA | I |
| RER1 | I |
| REL | I |
| APOL4 | I |
| PFDN4 | I |
| STARD4 | I |
| TFAP2A | I |
| CALM1 | I |
| VRK2 | I |
| MARVELD2 | I |
| TOMM40 | I |
| ZNF230 | I |
| CRKL | I |
| IL13RA1 | I |
| ZNF398 | I |
| ADAMTS5 | I |
| TRIM23 | I |
| SPAG9 | I |
| C11orf30 | I |
| TFPI2 | I |
| ADM | I |
| KCTD3 | I |
| RSBN1L | I |
| CYP1B1 | I |
| HMGCR | I |
| RAPGEF4 | I |
| HCFC1 | I |
| MBP | I |
| FLNB | I |
| EIF5B | I |
| PDK4 | I |
| TIMM8A | I |
| ANKRD27 | I |
| SEPP1 | I |
| CASP1 | I |
| SOCS4 | I |
| GHITM | I |
| SMAD9 | I |
| NUBPL | I |
| CTHRC1 | I |
| RAB30 | I |
| IL17D | I |
| WDR37 | I |
| PTN | I |
| TAF5 | I |
| TGM1 | I |
| PLSCR4 | I |
| ACAD8 | I |
| C9orf41 | I |
| BIRC6 | I |
| PSMA5 | I |
| DUSP19 | I |
| ARV1 | I |
| AUP1 | I |
| RPP14 | I |
| ARNTL2 | I |
| MAP1LC3B | I |
| NCOA3 | I |
| COX11 | I |
| NCOR2 | I |
| THAP1 | I |
| FGF23 | I |
| DDX46 | I |
| VAMP1 | I |
| CAP2 | I |
| FAM19A2 | I |
| PDE7A | I |
| DCK | I |
| TAX1BP1 | I |
| DAZL | I |
| RBBP8 | I |
| GPR160 | I |
| MFAP3 | I |
| C9orf3 | I |
| KIAA1279 | I |
| TCEAL4 | I |
| COIL | I |
| KLF3 | I |
| BAZ2B | I |
| CENPE | I |
| ZCCHC12 | I |
| MTRF1 | I |
| AUH | I |
| ZNF304 | I |
| AMACR | I |
| CAP1 | I |
| PAICS | I |
| TSNAX | I |
| CHCHD1 | I |
| RABIF | I |
| NCOA1 | I |
| ITSN2 | I |
| SMURF2 | I |
| SIPA1L2 | I |
| RPS24 | I |
| RNF128 | I |
| GIMAP2 | I |
| ARHGEF4 | I |
| CACYBP | I |
| MPHOSPH9 | I |
| SNRPD1 | I |
| MAN1A1 | I |
| MEIS1 | I |
| ST13 | I |
| LRP12 | I |
| BHLHB9 | I |
| FMNL2 | I |
| EXTL2 | I |
| HBP1 | I |
| LRRN1 | I |
| VPS13A | I |
| P2RY10 | I |
| RECK | I |
| CDCA7 | I |
| P2RY14 | I |
| HOXD11 | I |
| DNAJC7 | I |
| TNFSF10 | I |
| PRDX3 | I |
| ZNF165 | I |
| HERPUD1 | I |
| S100A9 | I |
| ZDHHC17 | I |
| GLRX2 | I |
| RBM6 | I |
| ATE1 | I |
| STARD13 | I |
| SSB | I |
| UBR1 | I |
| BHMT2 | I |
| MRPL1 | I |
| ZBTB24 | I |
| EPSTI1 | I |
| VCP | I |
| LTB4R | I |
| CRYZ | I |
| INSR | I |
| SLC35B4 | I |
| NAP1L4 | I |
| C9orf43 | I |
| RECQL5 | I |
| SULF2 | I |
| PAFAH1B2 | I |
| PARN | I |
| FIGNL1 | I |
| ARFGAP1 | I |
| PHC3 | I |
| DEF6 | I |
| BARD1 | I |
| USP12 | I |
| SIDT2 | I |
| CD22 | I |
| HACE1 | I |
| ENPP4 | I |
| CABYR | I |
| MTIF3 | I |
| RPS23 | I |
| NKX2-2 | I |
| QPCT | I |
| CETN1 | I |
| KCMF1 | I |
| CLEC2B | I |
| TBCA | I |
| PTPRG | I |
| KYNU | I |
| UGCG | I |
| TAF7 | I |
| MET | I |
| GTPBP3 | I |
| PLAG1 | I |
| NDUFB3 | I |
| CXCL2 | I |
| MAN1A2 | I |
| TRIM4 | I |
| MGAM | I |
| MDM1 | I |
| NCOA6 | I |
| DECR1 | I |
| VRK1 | I |
| STXBP5 | I |
| RNF2 | I |
| ZNF222 | I |
| FDFT1 | I |
| INHBA | I |
| CLEC4E | I |
| FBXO4 | I |
| NCF4 | I |
| PTPRF | I |
| ZMYND11 | I |
| LZTFL1 | I |
| CALCRL | I |
| HSPB1 | I |
| CAMTA1 | I |
| GPR27 | I |
| ATP11A | I |
| ASPM | I |
| ZNF197 | I |
| SMAD5 | I |
| RGS18 | I |
| AKAP11 | I |
| PSMA3 | I |
| MRPL42 | I |
| CEBPE | I |
| PGGT1B | I |
| HSPA2 | I |
| IGF1R | I |
| ADAM12 | I |
| DHX57 | I |
| TEX14 | I |
| NEBL | I |
| LPIN1 | I |
| TUBB | I |
| MMP14 | I |
| GCH1 | I |
| BEX1 | I |
| MYO10 | I |
| BUB1 | I |
| CCNJ | I |
| SEPHS1 | I |
| TCEAL1 | I |
| TFPI | I |
| PRKY | I |
| LYST | I |
| BAMBI | I |
| ENSA | I |
| ITGB2 | I |
| SLC25A13 | I |
| ZNF491 | I |
| SERPINA3 | I |
| LILRA2 | I |
| UCP1 | I |
| SSBP1 | I |
| ETV7 | I |
| MED31 | I |
| RPE | I |
| PER2 | I |
| ACOX1 | I |
| RBX1 | I |
| RRAGB | I |
| TFG | I |
| AGA | I |
| ASPH | I |
| STYK1 | I |
| COL3A1 | I |
| CALML5 | I |
| C3orf14 | I |
| SPESP1 | I |
| IL15 | I |
| ICK | I |
| CCNB1 | I |
| USMG5 | I |
| ATXN7 | I |
| SLC38A1 | I |
| PCBP1 | I |
| CLDN12 | I |
| PTGES | I |
| DNAJC3 | I |
| STRN | I |
| KPNA1 | I |
| MTRR | I |
| SACS | I |
| AP4E1 | I |
| HELLS | I |
| AP4S1 | I |
| HTR7 | I |
| BRCA1 | I |
| ALCAM | I |
| FTH1 | I |
| MAP3K5 | I |
| ROBO1 | I |
| SLC26A7 | I |
| FGF18 | I |
| ARMC2 | I |
| SLC35D1 | I |
| HINT3 | I |
| HSD17B7 | I |
| PCDH9 | I |
| LPL | I |
| HTATIP2 | I |
| RALBP1 | I |
| HOXA10 | I |
| ATP8A1 | I |
| RAD51 | I |
| FXR2 | I |
| TLL1 | I |
| C12orf10 | II |
| CHCHD5 | II |
| ERAL1 | II |
| MRPL28 | II |
| MRPL2 | II |
| ERCC1 | II |
| S100A16 | II |
| TCEB2 | II |
| NDUFB7 | II |
| NDUFA11 | II |
| GRIN2C | II |
| SNCG | II |
| GRINA | II |
| PLCD1 | II |
| C6orf106 | II |
| C19orf24 | II |
| R3HDML | II |
| TFPT | II |
| CES2 | II |
| GSTK1 | II |
| ATOX1 | II |
| SERPINF1 | II |
| NARFL | II |
| SMARCC2 | II |
| STAP2 | II |
| NKIRAS2 | II |
| COL6A1 | II |
| POLR2I | II |
| GLIS1 | II |
| APEX2 | II |
| GPR62 | II |
| SNAPC2 | II |
| C9orf116 | II |
| TRIM26 | II |
| ARID1B | II |
| COX6A1 | II |
| CDH15 | II |
| ZNF574 | II |
| RTKN | II |
| TMEM9 | II |
| ALOXE3 | II |
| KIF17 | II |
| SIRT6 | II |
| CCS | II |
| OXER1 | II |
| TM6SF2 | II |
| NXPH4 | II |
| MC5R | II |
| KHSRP | II |
| KRTCAP2 | II |
| SLC43A1 | II |
| G6PC3 | II |
| BRF1 | II |
| DYRK1B | II |
| TRIM47 | II |
| RILP | II |
| RASL12 | II |
| CABP4 | II |
| PPIH | II |
| SDSL | II |
| UCKL1 | II |
| LCMT1 | II |
| UFD1L | II |
| CACNA1H | II |
| EMID1 | II |
| FBXO17 | II |
| BET1L | II |
| TMEM39B | II |
| TARBP2 | II |
| PRR3 | II |
| PRKCDBP | II |
| BARX2 | II |
| GRIK5 | II |
| PSENEN | II |
| HMBS | II |
| APLN | II |
| CUEDC2 | II |
| TRIM21 | II |
| SLC29A3 | II |
| MINK1 | II |
| CHRM1 | II |
| LAMC3 | II |
| ZNF213 | II |
| NOVA2 | II |
| A1BG | II |
| WDR24 | II |
| TYRO3 | II |
| BOK | II |
| ZNF324 | II |
| C9orf16 | II |
| CLDN3 | II |
| GGN | II |
| ACD | II |
| CIRH1A | II |
| C8A | II |
| LDHD | II |
| PCDHB4 | II |
| IDH3G | II |
| ALX3 | II |
| CNOT2 | II |
| SSSCA1 | II |
| VPS4A | II |
| DRAP1 | II |
| CHST10 | II |
| CYGB | II |
| RERE | II |
| CALML4 | II |
| GPSM3 | II |
| SLC13A4 | II |
| KCNJ8 | II |
| FUT5 | II |
| ANGPTL6 | II |
| LDOC1 | II |
| CHCHD6 | II |
| PCDH12 | II |
| TCF4 | II |
| NUP214 | II |
| NME4 | II |
| RRAS | II |
| DNAJC4 | II |
| NINJ2 | II |
| MRPS34 | II |
| AP1M2 | II |
| OR12D2 | II |
| TAF10 | II |
| WDTC1 | II |
| ZNF593 | II |
| WDR45 | II |
| FGD1 | II |
| PRPH | II |
| TNNC1 | II |
| ETHE1 | II |
| TCOF1 | II |
| CDH3 | II |
| MRPS27 | II |
| PLEKHF1 | II |
| INHBE | II |
| NDUFB11 | II |
| ATPAF2 | II |
| ALG3 | II |
| NUBP1 | II |
| KATNB1 | II |
| H2AFY2 | II |
| PLA2G2A | II |
| SLC41A3 | II |
| MPI | II |
| RUVBL1 | II |
| SMYD5 | II |
| ROBO4 | II |
| SUPT5H | II |
| CAD | II |
| MRPS12 | II |
| RNF121 | II |
| NR5A2 | II |
| SSTR2 | II |
| DPT | II |
| LYZL6 | II |
| LHFPL5 | II |
| CRB3 | II |
| FREM1 | II |
| PAK6 | II |
| TFF1 | II |
| GPX2 | II |
| EVI5L | II |
| SNX8 | II |
| CHPF | II |
| MRPS26 | II |
| SLC38A5 | II |
| RAB3IL1 | II |
| ACY3 | II |
| PA2G4 | II |
| SRC | II |
| CNGB1 | II |
| POU3F4 | II |
| DCPS | II |
| TRAPPC1 | II |
| TAOK2 | II |
| KIF1A | II |
| GPR12 | II |
| HCP5 | II |
| PDRG1 | II |
| SLC7A13 | II |
| MST1R | II |
| ABCC10 | II |
| CA11 | II |
| ADRB3 | II |
| TNC | II |
| MOV10 | II |
| NAGPA | II |
| TGFB1I1 | II |
| IL17F | II |
| TIAM1 | II |
| CLEC11A | II |
| IFRD2 | II |
| MLANA | II |
| ALS2CL | II |
| ARL2 | II |
| MRPL37 | II |
| PTPN23 | II |
| BCAS4 | II |
| DPM2 | II |
| GZMM | II |
| EEF1D | II |
| LAS1L | II |
| CNFN | II |
| COL4A1 | II |
| RPUSD3 | II |
| KRT15 | II |
| GPR3 | II |
| CIZ1 | II |
| MPG | II |
| APOA4 | II |
| COL5A3 | II |
| CRYBB1 | II |
| COX7A1 | II |
| ZNF71 | II |
| ST5 | II |
| CCDC12 | II |
| LMOD1 | II |
| FHL3 | II |
| EMILIN1 | II |
| ZMAT2 | II |
| LLGL1 | II |
| PYY | II |
| NCR2 | II |
| RFXANK | II |
| RORC | II |
| ZNF317 | II |
| ASB13 | II |
| IQGAP3 | II |
| CD34 | II |
| POLDIP2 | II |
| FXYD3 | II |
| BCL2L13 | II |
| TBXA2R | II |
| RRH | II |
| SSBP3 | II |
| SAMD1 | II |
| SLC29A1 | II |
| HIST1H2AJ | II |
| GYG2 | II |
| AP2B1 | II |
| FOXA2 | II |
| PPL | II |
| MAPT | II |
| HAND1 | II |
| PLAGL2 | II |
| AP3M2 | II |
| TAS1R1 | II |
| UMOD | II |
| POLR2L | II |
| XAB2 | II |
| SLC29A4 | II |
| FRAS1 | II |
| POLR3D | II |
| ZNF343 | II |
| PLXDC1 | II |
| CACNA1F | II |
| RAD52 | II |
| EXOSC10 | II |
| HCRT | II |
| HAPLN4 | II |
| SNCB | II |
| FMO1 | II |
| JAK3 | II |
| BNIP1 | II |
| BTNL2 | II |
| MAP1LC3A | II |
| TSSC1 | II |
| DDX51 | II |
| WNT9B | II |
| H1FX | II |
| COL4A2 | II |
| NRG4 | II |
| EIF2B5 | II |
| ACADVL | II |
| WWOX | II |
| P2RX2 | II |
| PCSK1N | II |
| OPN4 | II |
| TJP3 | II |
| MRPL23 | II |
| LPPR2 | II |
| KPNA6 | II |
| MSI2 | II |
| NDUFA7 | II |
| CMIP | II |
| HGFAC | II |
| LCN1 | II |
| PTTG1 | II |
| PYCRL | II |
| ACTR1A | II |
| FARS2 | II |
| TUFM | II |
| CD6 | II |
| STMN2 | II |
| ADIPOR1 | II |
| MASP2 | II |
| ZNF205 | II |
| PAX4 | II |
| NRM | II |
| RANBP10 | II |
| PPOX | II |
| HYDIN | II |
| GCM2 | II |
| PLA2G5 | II |
| FXYD6 | II |
| ASMTL | II |
| EYA3 | II |
| DLL4 | II |
| POLDIP3 | II |
| ITPKC | II |
| CLTB | II |
| POLRMT | II |
| TNFRSF4 | II |
| SOX9 | II |
| NUDT16L1 | II |
| OPA3 | II |
| TRPV5 | II |
| POMT1 | II |
| ZAR1 | II |
| PES1 | II |
| SCAMP4 | II |
| GPHN | II |
| GPR37 | II |
| MESP1 | II |
| E4F1 | II |
| NTHL1 | II |
| MCOLN1 | II |
| CRYBB2 | II |
| PTOV1 | II |
| GUCA1A | II |
| PFKM | II |
| FAM20C | II |
| PRM3 | II |
| PAFAH1B3 | II |
| RAB33A | II |
| KIRREL | II |
| PACSIN3 | II |
| KCTD17 | II |
| C14orf132 | II |
| MAPK12 | II |
| FZR1 | II |
| CSMD1 | II |
| ATP5G1 | II |
| MAF1 | II |
| SRM | II |
| SPRR3 | II |
| HMGCL | II |
| RNF26 | II |
| OR8B8 | II |
| SPRY3 | II |
| LOXL2 | II |
| MRPL20 | II |
| UNC13D | II |
| KRT16 | II |
| ASB16 | II |
| ZNF511 | II |
| ODF2 | II |
| NR2E3 | II |
| SYN1 | II |
| COPE | II |
| ZNRD1 | II |
| IPO4 | II |
| BMP2 | II |
| ISLR | II |
| ZDHHC19 | II |
| MOGAT3 | II |
| SOX7 | II |
| ST3GAL2 | II |
| RBM15B | II |
| SLITRK3 | II |
| CDC25A | II |
| SLC27A5 | II |
| BRD4 | II |
| LRP1 | II |
| TSHB | II |
| FCER1G | II |
| OAS2 | II |
| PCDHB6 | II |
| CYB561D2 | II |
| PCDHB1 | II |
| VRK3 | II |
| TXN2 | II |
| RGS6 | II |
| PUS3 | II |
| SCO2 | II |
| PIGQ | II |
| CYP2S1 | II |
| HAMP | II |
| SPTBN2 | II |
| BAP1 | II |
| SPON1 | II |
| ISYNA1 | II |
| FOXC1 | II |
| ZC3HC1 | II |
| GJA4 | II |
| DCBLD1 | II |
| JAM2 | II |
| KRT20 | II |
| ZNF395 | II |
| ZMYND15 | II |
| GSTM2 | II |
| STARD5 | II |
| DTX3 | II |
| CNGA1 | II |
| PHLDA3 | II |
| SLC13A5 | II |
| TACR1 | II |
| SCARB2 | II |
| ZCCHC5 | II |
| PTGIR | II |
| PTP4A3 | II |
| DDX24 | II |
| PCNXL2 | II |
| KLHL17 | II |
| XPNPEP2 | II |
| TRIM10 | II |
| HOXD1 | II |
| ZNF133 | II |
| CRELD1 | II |
| SOX15 | II |
| HIST1H2BJ | II |
| UAP1L1 | II |
| TRIM41 | II |
| FKBP8 | II |
| LAMA4 | II |
| SLC16A9 | II |
| OAZ3 | II |
| MCM5 | II |
| MRPS18A | II |
| PABPN1 | II |
| SULT1C2 | II |
| ZSCAN1 | II |
| SLC22A18 | II |
| CD7 | II |
| KLHDC3 | II |
| CABP5 | II |
| SCARF2 | II |
| MOSPD3 | II |
| AMHR2 | II |
| C1orf50 | II |
| CIB1 | II |
| PTPRD | II |
| ADD1 | II |
| ATP5D | II |
| SLC25A28 | II |
| CD79A | II |
| GPR50 | II |
| RPL8 | II |
| SYCP1 | II |
| KCTD13 | II |
| MAPKAPK3 | II |
| CRABP1 | II |
| NPR2 | II |
| GPR20 | II |
| DPAGT1 | II |
| RPUSD2 | II |
| ATP5I | II |
| WFDC9 | II |
| POLE4 | II |
| RENBP | II |
| CA5A | II |
| KCNN1 | II |
| CHRNB1 | II |
| TIMM50 | II |
| ARR3 | II |
| DPP7 | II |
| C10orf62 | II |
| FGD5 | II |
| EME1 | II |
| MYBPC2 | II |
| BYSL | II |
| TROAP | II |
| PPP2R2C | II |
| PCOLCE | II |
| ARHGEF17 | II |
| PSMF1 | II |
| SPATA2 | II |
| AIRE | II |
| ABCF1 | II |
| VPS33B | II |
| KLC2 | II |
| SIDT1 | II |
| MYOG | II |
| SERPINB5 | II |
| FMO3 | II |
| CDAN1 | II |
| HEXA | II |
| MRPS11 | II |
| SEZ6L2 | II |
| RPL36 | II |
| FHIT | II |
| XRCC3 | II |
| BCAN | II |
| PHOSPHO1 | II |
| ELF5 | II |
| PBOV1 | II |
| TIMM17B | II |
| POU2F2 | II |
| DNAL4 | II |
| SLC2A4RG | II |
| ACIN1 | II |
| IGSF8 | II |
| PTPRT | II |
| HOXC8 | II |
| APOE | II |
| YY1AP1 | II |
| MRPS15 | II |
| HK1 | II |
| PIWIL2 | II |
| HIST1H4A | II |
| SLC10A1 | II |
| FETUB | II |
| S100Z | II |
| SF3A2 | II |
| IGFBP5 | II |
| PPP1R13L | II |
| HYOU1 | II |
| HBE1 | II |
| GLTSCR1 | II |
| SOX21 | II |
| C1orf35 | II |
| ARID3A | II |
| SHANK1 | II |
| DPEP3 | II |
| TLN1 | II |
| KLK5 | II |
| CRLF2 | II |
| FBXO40 | II |
| PDCL2 | II |
| FBXO31 | II |
| WFDC8 | II |
| VGLL1 | II |
| HSD17B1 | II |
| UBE2L6 | II |
| PXMP4 | II |
| CD248 | II |
| WFDC1 | II |
| SH2D3A | II |
| CHDH | II |
| SULT2A1 | II |
| HMGA1 | II |
| SYNGR1 | II |
| SLIT3 | II |
| DEAF1 | II |
| PTCRA | II |
| POU1F1 | II |
| MYL6 | II |
| CAV1 | II |
| CLIC6 | II |
| KRTAP3-1 | II |
| ATIC | II |
| PSG2 | II |
| ZFYVE1 | II |
| GSTO2 | II |
| GYS1 | II |
| REG3A | II |
| CCL21 | II |
| PPRC1 | II |
| ARMC7 | II |
| MRPL22 | II |
| ST6GALNAC4 | II |
| MAFF | II |
| OR2H2 | II |
| MYO18B | II |
| OCRL | II |
| OR2H1 | II |
| F9 | II |
| SHMT2 | II |
| STRA6 | II |
| BSG | II |
| GFAP | II |
| EPB41L4A | II |
| MGC50722 | II |
| CDC25B | II |
| CPT1C | II |
| HDAC6 | II |
| HRC | II |
| LGALS4 | II |
| NODAL | II |
| RUVBL2 | II |
| SPACA3 | II |
| MICA | II |
| RPAP1 | II |
| SLC27A1 | II |
| FOLR1 | II |
| MARCKSL1 | II |
| CPB1 | II |
| CLPP | II |
| NDUFB2 | II |
| B3GNT4 | II |
| PTPN6 | II |
| ITGAL | II |
| RHEBL1 | II |
| DDX49 | II |
| ACAD9 | II |
| NAPA | II |
| FEZ1 | II |
| TBL1X | II |
| IFI27 | II |
| ST8SIA2 | II |
| SSR4 | II |
| TLN2 | II |
| CKB | II |
| RHBG | II |
| NES | II |
| GPX5 | II |
| GLB1L | II |
| C14orf93 | II |
| CCR10 | II |
| GPRC6A | II |
| DHDDS | II |
| SMO | II |
| ZBTB37 | II |
| PYGO2 | II |
| RASGRF2 | II |
| UPP2 | II |
| GSTM5 | II |
| UGT2B15 | II |
| NKX2-3 | II |
| DHX35 | II |
| SLC41A1 | II |
| PALM2 | II |
| UPK3B | II |
| WFDC3 | II |
| GPS2 | II |
| FGFRL1 | II |
| F5 | II |
| PKLR | II |
| DSC3 | II |
| NPAS1 | II |
| MPV17 | II |
| NUDT2 | II |
| LLGL2 | II |
| SRD5A2 | II |
| MUSK | II |
| WBSCR22 | II |
| TMC5 | II |
| KLF14 | II |
| HTR3A | II |
| SLCO3A1 | II |
| PIGO | II |
| TRIM11 | II |
| CDC42BPG | II |
| TTBK1 | II |
| UMODL1 | II |
| DUSP16 | II |
| CXCL16 | II |
| ANGPTL7 | II |
| KLF16 | II |
| CRMP1 | II |
| LZTS2 | II |
| RPS10 | II |
| SLC9A3R1 | II |
| SLC28A2 | II |
| THRA | II |
| SLC1A1 | II |
| COL4A5 | II |
| TBC1D13 | II |
| GNB1L | II |
| NEK6 | II |
| EXOSC5 | II |
| BRF2 | II |
| TM4SF5 | II |
| GSTA3 | II |
| SLC17A7 | II |
| ASXL1 | II |
| EDA | II |
| BRAF | II |
| SSH3 | II |
| ALPP | II |
| GSTO1 | II |
| RETNLB | II |
| GABRA3 | II |
| BAALC | II |
| NLGN2 | II |
| GSS | II |
| MAP2K5 | II |
| ENTPD5 | II |
| ZNF653 | II |
| MAP2K7 | II |
| TM4SF4 | II |
| HRAS | II |
| NDUFV3 | II |
| GNG13 | II |
| RTN2 | II |
| SCT | II |
| UNC5B | II |
| LEF1 | II |
| COL5A1 | II |
| CACNA1B | II |
| PCK2 | II |
| CDSN | II |
| CNKSR1 | II |
| C6orf15 | II |
| BCKDK | II |
| FBXO9 | II |
| OR51B5 | II |
| COMMD1 | II |
| FMOD | II |
| LTK | II |
| RTN4RL2 | II |
| TCP10L | II |
| ITGB5 | II |
| OCA2 | II |
| IL27RA | II |
| PEX11B | II |
| GABRA1 | II |
| KRTAP11-1 | II |
| HNF4A | II |
| SH2D2A | II |
| FHL5 | II |
| GAL | II |
| RNF40 | II |
| KRTAP4-5 | II |
| LSM10 | II |
| LENG1 | II |
| SERPINB6 | II |
| APOBEC3G | II |
| POLR3A | II |
| PRSS21 | II |
| C20orf144 | II |
| ABCF3 | II |
| KCNJ1 | II |
| PPP1R16A | II |
| PFKFB3 | II |
| MMP19 | II |
| FOLH1 | II |
| NUDT16 | II |
| ZNF541 | II |
| USP10 | II |
| GABRB2 | II |
| EIF2AK1 | II |
| DOLPP1 | II |
| CSF1R | II |
| MPP1 | II |
| HSD17B8 | II |
| TMEM41A | II |
| P2RY4 | II |
| HES4 | II |
| GPR25 | II |
| CNOT10 | II |
| ASTN2 | II |
| MRPL17 | II |
| CDC42BPB | II |
| GOT1 | II |
| APEH | II |
| CPSF1 | II |
| RHOF | II |
| ARRB1 | II |
| MMP26 | II |
| KPTN | II |
| IL9 | II |
| ASCC2 | II |
| PRPF4 | II |
| CHAF1A | II |
| FGF10 | II |
| PNKP | II |
| SLC25A23 | II |
| PKN3 | II |
| CPN1 | II |
| MOG | II |
| PHYHD1 | II |
| GEMIN6 | II |
| GP9 | II |
| OR3A2 | II |
| MVP | II |
| TIE1 | II |
| PARVA | II |
| KIF13B | II |
| TGM2 | II |
| CX3CL1 | II |
| NDUFA9 | II |
| DDO | II |
| OR4D2 | II |
| ATOH8 | II |
| GIT1 | II |
| SLC2A6 | II |
| CECR1 | II |
| TAF6 | II |
| MAX | II |
| FCN3 | II |
| GML | II |
| UTF1 | II |
| PMF1 | II |
| G6PC2 | II |
| PDGFB | II |
| CETP | II |
| PSMA7 | II |
| GTPBP2 | II |
| GEMIN5 | II |
| MYO1A | II |
| BIN1 | II |
| IMPDH2 | II |
| CD4 | II |
| OXA1L | II |
| HIST1H2AM | II |
| ADH1C | II |
| SRPX2 | II |
| ADSL | II |
| BCL6B | II |
| NYX | II |
| HMG20B | II |
| SDS | II |
| CNR2 | II |
| STC1 | II |
| DHODH | II |
| TBC1D22B | II |
| SLC6A15 | II |
| CYP3A43 | II |
| OR10H1 | II |
| ZNF232 | II |
| HOXA13 | II |
| LRCH3 | II |
| EFNA1 | II |
| UNC93B1 | II |
| F7 | II |
| POP5 | II |
| GABRA2 | II |
| NFKBIB | II |
| ADSSL1 | II |
| TBC1D20 | II |
| ATP1B2 | II |
| ACMSD | II |
| EFHD1 | II |
| ALDOC | II |
| FAM47B | II |
| SH3TC1 | II |
| NINJ1 | II |
| NPTXR | II |
| ITPKB | II |
| RAB2B | II |
| METTL1 | II |
| LSS | II |
| NEURL2 | II |
| TEX13A | II |
| QPRT | II |
| ZNF418 | II |
| ODC1 | II |
| LGR5 | II |
| PVRL2 | II |
| ICAM3 | II |
| VAT1 | II |
| GPR15 | II |
| HSPB2 | II |
| TIMM23 | II |
| CGN | II |
| CLN6 | II |
| POLL | II |
| SLC4A3 | II |
| FAM53C | II |
| LTB4R2 | II |
| INA | II |
| MPDZ | II |
| AKAP6 | II |
| ANK2 | II |
| ONECUT1 | II |
| KRT8 | II |
| PQLC2 | II |
| TRAF2 | II |
| KNDC1 | II |
| C2 | II |
| KRTAP1-5 | II |
| ZDHHC5 | II |
| LAMB3 | II |
| KRT9 | II |
| GTPBP1 | II |
| FGF21 | II |
| APH1A | II |
| MYD88 | II |
| GPR68 | II |
| CCDC7 | II |
| GRK6 | II |
| APCS | II |
| MC2R | II |
| LY86 | II |
| CACNA1C | II |
| CD86 | II |
| SLC6A17 | II |
| SLC22A11 | II |
| FCGR2B | II |
| HES5 | II |
| IRAK2 | II |
| LPAL2 | II |
| FGF3 | II |
| BFSP1 | II |
| CSTF2 | II |
| MAT1A | II |
| SLC4A1 | II |
| FTSJ1 | II |
| LTBP4 | II |
| SETMAR | II |
| DZIP1 | II |
| GPC1 | II |
| NAV1 | II |
| IRX3 | II |
| CPLX2 | II |
| CD84 | II |
| LAMP3 | II |
| ST3GAL3 | II |
| ZNF79 | II |
| NDN | II |
| RAI1 | II |
| KRTAP5-9 | II |
| CREB3L1 | II |
| TTC12 | II |
| PREB | II |
| SUFU | II |
| SIGIRR | II |
| PDK2 | II |
| GLIS2 | II |
| PRSS16 | II |
| MTX1 | II |
| LAMA1 | II |
| SYK | II |
| LRRC28 | II |
| CHST6 | II |
| RGS10 | II |
| PTDSS1 | II |
| CHIA | II |
| NKD2 | II |
| GABRP | II |
| CNIH2 | II |
| SORCS2 | II |
| CD79B | II |
| CACNB2 | II |
| CENPB | II |
| COMMD7 | II |
| IL22RA2 | II |
| VAC14 | II |
| ADAMTS2 | II |
| SPOCK2 | II |
| FHOD1 | II |
| PLTP | II |
| MYLK2 | II |
| PGLYRP2 | II |
| RLBP1 | II |
| HBD | II |
| SPTAN1 | II |
| ZCCHC3 | II |
| APEX1 | II |
| ABCC3 | II |
| UPB1 | II |
| ETV5 | II |
| STK16 | II |
| PARP16 | II |
| NR5A1 | II |
| NTRK2 | II |
| VIPR1 | II |
| BLCAP | II |
| WNK4 | II |
| GLI4 | II |
| MMP15 | II |
| KLK14 | II |
| B4GALT7 | II |
| PINX1 | II |
| PTGDS | II |
| FXYD7 | II |
| CDH6 | II |
| CDC42EP5 | II |
| SCNN1B | II |
| REM1 | II |
| MCOLN3 | II |
| SPATS2 | II |
| CALU | II |
| PEMT | II |
| KRT1 | II |
| ME3 | II |
| TRIM35 | II |
| CACNA2D1 | II |
| DHTKD1 | II |
| GMEB2 | II |
| ITM2C | II |
| LTC4S | II |
| COG7 | II |
| GNA15 | II |
| NFATC2IP | II |
| MOCS1 | II |
| MYOCD | II |
| GRM5 | II |
| JUN | II |
| ASF1B | II |
| GFRA1 | II |
| PEX11G | II |
| SFN | II |
| LHX5 | II |
| ZNF619 | II |
| PTPRM | II |
| ROR1 | II |
| MSN | II |
| TMPRSS4 | II |
| CHRNA2 | II |
| TRIB3 | II |
| RDH8 | II |
| MTSS1 | II |
| TTC17 | II |
| TGM4 | II |
| H6PD | II |
| MAN2B1 | II |
| KCNK7 | II |
| NR4A1 | II |
| PIAS4 | II |
| AQP8 | II |
| PCDHA9 | II |
| TGM3 | II |
| DLX4 | II |
| DCTN1 | II |
| PARVB | II |
| TPM2 | II |
| TYROBP | II |
| LMX1A | II |
| NDUFS7 | II |
| CTSG | II |
| PPP1R3F | II |
| TAF12 | II |
| PRKAB1 | II |
| ICAM5 | II |
| IGFBP6 | II |
| GNG5 | II |
| CYP7A1 | II |
| CECR5 | II |
| ERN1 | II |
| TCTA | II |
| RASGRP4 | II |
| SPHK2 | II |
| PPM1E | II |
| UNC13B | II |
| CACNG7 | II |
| STMN1 | II |
| MRPL24 | II |
| SLC34A1 | II |
| ELP4 | II |
| HIF1AN | II |
| POLR1D | II |
| VPS18 | II |
| SH3GL3 | II |
| CDH10 | II |
| CYP2W1 | II |
| COMMD9 | II |
| OTOS | II |
| SEMA6D | II |
| AMN | II |
