# Supplementary material for: Identifying molecular subgroups of patients with preeclampsia through bioinformatics
Source: Front Cardiovasc Med. 2024 Jun 3;11:1367578. doi: 10.3389/fcvm.2024.1367578 (PMC11180819; doi:10.3389/fcvm.2024.1367578)
Supplement: Supplementary file 6 [file Table6.docx]

**Supplementary Table S6.** KEGG enrichment pathway of gene modules

| KEGG_  Pathway | Term | PValue | Fold enrichment | Count | % | Genes |
| --- | --- | --- | --- | --- | --- | --- |
| Blue _1 | hsa05222:  Small cell lung cancer | 0.0002 | 2.5037 | 20 | 1.42 | LAMB3, CHUK, APAF1, LAMC3, MAX,  LAMA4, PTEN, TRAF2, PIK3CB, PTGS2,  GADD45G, COL4A2, COL4A1, CASP3,  TRAF5, COL4A5, ITGAV, E2F3, POLK, BIRC3 |
| Blue _2 | hsa04140:  Autophagy - animal | 0.0009 | 2.0420 | 25 | 1.77 | RAB1A, PRKAA1, PIK3R4, PTEN, PIK3CB,  TANK, C9ORF72, ZFYVE1, PPP2CA,  MAP1LC3B, MAP1LC3A, LAMP1, RB1CC1,  HRAS, SH3GLB1, UVRAG, EIF2AK3,  EIF2S1, RAB33B, ERN1, RPS6KB1, RRAGB,  RHEB, KRAS, RAF1 |
| Blue _3 | hsa05014:  Amyotrophic lateral sclerosis | 0.0012 | 1.5820 | 50 | 3.55 | COX7B, ACTR1A, MAP1LC3B, MAP1LC3A,  CASP3, KIF5B, RB1CC1, CASP1, NUP214,  APAF1, TRAF2, SDHD, ERN1, PSMA5,  PSMA3, NRG4, PSMA2, NDUFS2, MATR3,  VDAC1, UQCRC2, ATF4, RAB1A, PSMD12,  VCP, UQCRB, PSMD14, NDUFB5, DCTN1,  DCTN4, NDUFB3, PIK3R4, TANK, COX7A1,  C9ORF72, HDAC6, NUP160, KLC2,  NUP43, RANBP2, NDUFA5, EIF2AK3,  GRIN2C, EIF2S1, MAPK12, PSMC5, CCS,  PSMC6, TARDBP, DNAL4 |
| Blue _4 | hsa04932:  Non-alcoholic fatty liver disease | 0.0017 | 1.9319 | 26 | 1.84 | COX7B, PRKAA1, UQCRB, NDUFB5,  NDUFB3, PIK3CB, ADIPOR1, COX7A1,  CASP7, CASP3, MLX, PRKAB2, NDUFA5,  INSR, EIF2AK3, TRAF2, FOS, SDHD,  EIF2S1, PRKAB1, MAPK12, ERN1, ITCH,  NDUFS2, UQCRC2, ATF4 |
| Blue _5 | hsa05010:  Alzheimer disease | 0.0021 | 1.5296 | 51 | 3.62 | COX7B, CHRM1, PIK3CB, CASP7, CASP3,  KIF5B, RB1CC1, SLC39A7, HRAS, PSENEN,  APAF1, CHUK, SLC39A10, TRAF2, SDHD,  ERN1, PSMA5, PSMA3, PSMA2, PPIF,  NDUFS2, VDAC2, VDAC1, MAPT, UQCRC2,  RAF1, PLCB1, ATF4, PSMD12, LRP1,  UQCRB, PSMD14, NDUFB5, NDUFB3,  PIK3R4, PTGS2, COX7A1, APH1A, KLC2,  MAP2K7, NDUFA5, CSNK1A1, INSR,  FZD6, EIF2AK3, GRIN2C, EIF2S1, PSMC5,  PSMC6, KRAS, CALM1 |
| Blue _6 | hsa05022:  Pathways of neurodegeneration - multiple diseases | 0.0028 | 1.4517 | 60 | 4.26 | COX7B, CHRM1, UBE2L6, ZFYVE1,  ACTR1A, CASP7, MAP1LC3B, MAP1LC3A,  CASP3, KIF5B, RB1CC1, HRAS, GPR37,  APAF1, TRAF2, SDHD, ERN1, PSMA5,  PSMA3, PSMA2, PPIF, NDUFS2, VDAC2,  VDAC1, MAPT, UQCRC2, RAF1, PLCB1,  ATF4, RAB1A, PSMD12, VCP, UQCRB,  PSMD14, NDUFB5, DCTN1, DCTN4,  NDUFB3, PIK3R4, PTGS2, TANK,  COX7A1, C9ORF72, KLC2, MAP2K7,  SPTBN2, NDUFA5, CSNK1A1, FZD6,  EIF2AK3, GRIN2C, EIF2S1, MAPK12, PSMC5, CCS, PSMC6, KRAS, TARDBP, CALM1, DNAL4 |
| Blue _7 | hsa05134:  Legionellosis | 0.0030 | 2.6267 | 13 | 0.92 | RAB1A, VCP, APAF1, IL18, CXCL2, CLK4,  CLK1, EEF1A1, BCL2L13, CASP7, CASP3,  CASP1, MYD88 |
| Blue _8 | hsa05131:  Shigellosis | 0.0045 | 1.6320 | 35 | 2.48 | SRC, PIK3R4, BCL10, PIK3CB, MALT1, HK1,  CRKL, MAP1LC3B, MAP1LC3A, RPS6KA5,  CASP1, ACTR3, CAST, ACTR2, CHUK, IL1R1,  IL18, TRAF2, RHOA, MAPK12, RBX1,  RPS6KB1, RRAGB, ARPC3, TRAF5,  UBE2N, UBE2V2, ATM, VDAC1,  TLN2, PLCB1, TLN1, MYD88, PLCD1, ARF6 |
| Blue _9 | hsa00563:  Glycosylphosphatidylinositol (GPI)-anchor biosynthesis | 0.0054 | 3.5437 | 8 | 0.56 | DPM2, PIGO, PIGN, PIGQ, PIGA, PIGK, PIGF,  PIGH |
| Blue _10 | hsa05230:  Central carbon metabolism in cancer | 0.0065 | 2.3034 | 14 | 0.99 | PDHA1, PTEN, SIRT6, PIK3CB, HK1, LDHB,  SCO2, KIT, KRAS, RAF1, HRAS, MET, PFKM,  PDK1 |
| Blue _11 | hsa03250:  Viral life cycle - HIV-1 | 0.0071 | 2.3765 | 13 | 0.92 | RANBP2, CCNT2, CPSF6, PDCD6IP,  APOBEC3G, VPS4B, VPS4A, PSIP1, SUPT5H,  ELL2, CD4, XPO1, FEZ1 |
| Blue _12 | hsa05012:  Parkinson disease | 0.0081 | 1.5587 | 36 | 2.55 | COX7B, PSMD12, UQCRB, PSMD14,  NDUFB5, NDUFB3, GNAI3, UBE2L6,  COX7A1, GNAI1, KLC2, CASP3, KIF5B,  SLC39A7, GPR37, NDUFA5, APAF1,  EIF2AK3, SLC39A10, SDHD, EIF2S1, ERN1,  PSMA5, PSMC5, PSMA3, PSMC6, PSMA2,  PPIF, NDUFS2, VDAC2, VDAC1, UQCRC2,  MAPT, CALM1, ATF4, NFE2L2 |
| Blue _13 | hsa04510:  Focal adhesion | 0.0091 | 1.6453 | 29 | 2.05 | ITGB5, LAMC3, SRC, LAMA4, PTEN, PDGFB, TNC, PIK3CB, ARHGAP5, CRKL, PPP1CB, PDGFC, PAK6, ITGAV, HRAS, PPP1R12A, LAMB3, CAV1, PARVB, RHOA, COL4A2, COL4A1, COL6A1, COL4A5, TLN2, RAF1, TLN1, MET, BIRC3 |
| Blue _14 | hsa04115:  p53 signaling pathway | 0.0104 | 2.1789 | 14 | 0.99 | APAF1, SIAH1, PTEN, RCHY1, PPM1D,  GADD45G, CCNB1, PERP, CASP3, CCNG2,  CCNG1, MDM4, ATM, SFN |
| Blue _15 | hsa04120:  Ubiquitin mediated proteolysis | 0.0105 | 1.7843 | 22 | 1.56 | PIAS4, CUL3, CUL2, SIAH1, UBE4A,  WWP1, UBE2L6, BRCA1, UBE2A, RCHY1,  RBX1, CUL4A, HERC4, FZR1, ITCH,  CDC16, FBXO4, UBE2N, BIRC6, TRIP12,  TRIM37, BIRC3 |
| Brown _1 | hsa03010:  Ribosome | <0.0001 | 4.9545 | 13 | 5.75 | MRPS15, MRPS11, MRPS12, MRPS18A,  MRPL17, MRPL28, MRPL23, RPL8,  MRPL24, MRPL22, MRPL20, RPL36, RPS10 |
| Brown _2 | hsa05016:  Huntington disease | 0.0003 | 3.1199 | 15 | 6.64 | NDUFA9, NDUFA7, NDUFB7, NDUFA11,  NDUFB11, TUBB, NDUFB2, AP2B1,  COX6A1, PSMA7, NDUFS7, GNAQ, NDUFV3,  POLR2I, POLR2L |
| Brown _3 | hsa04723:  Retrograde endocannabinoid signaling | 0.0004 | 4.3004 | 10 | 4.42 | NDUFA9, NDUFA7, NDUFB7, GNG5,  NDUFS7, NDUFA11, NDUFB11, GNAQ,  NDUFB2, NDUFV3 |
| Brown _4 | hsa01100:  Metabolic pathways | 0.0009 | 1.6521 | 40 | 17.70 | ISYNA1, NDUFB7, NDUFA11, SHMT2,  NDUFB11, DPYS, HEXA, POMT1, NDUFB2,  NUDT2, COX6A1, FHIT, HSD17B8, SRM,  DHTKD1, HMGCL, UGCG, PTDSS1, ATIC,  NUDT16, NDUFV3, PCK2, NDUFA9,  GSTK1, NDUFA7, ADSL, IDH3G, GSTO1,  GSS, SETMAR, ASMTL, SYNJ2, DHODH,  NDUFS7, IMPDH2, ETHE1, HMBS,  PEMT, PAFAH1B3, SLC27A5 |
| Brown _5 | hsa00190:  Oxidative phosphorylation | 0.001136 | 4.2748 | 9 | 4.00 | NDUFA9, NDUFA7, NDUFB7, NDUFS7,  NDUFA11, NDUFB11, NDUFB2, NDUFV3,  COX6A1 |
| Brown _6 | hsa04932:  Non-alcoholic fatty liver disease | 0.0029 | 3.696 | 9 | 4.00 | NDUFA9, NDUFA7, NDUFB7, NDUFS7,  NDUFA11, NDUFB11, NDUFB2, NDUFV3,  COX6A1 |
| Brown _7 | hsa05012:  Parkinson disease | 0.0029 | 2.8713 | 12 | 5.31 | NDUFA9, NDUFA7, NDUFB7, NDUFS7,  NDUFA11, NDUFB11, TUBB, NDUFB2,  NDUFV3, COX6A1, PSMA7, TXN2 |
| Brown _8 | hsa03410:  Base excision repair | 0.0047 | 7.2326 | 5 | 2.21 | POLE4, NTHL1, MPG, PNKP, APEX1 |
| Brown _9 | hsa05208:  Chemical carcinogenesis - reactive oxygen species | 0.0080 | 2.8541 | 10 | 4.42 | NDUFA9, NDUFA7, NDUFB7, NDUFS7,  NDUFA11, GSTO1, NDUFB11, NDUFB2,  NDUFV3, COX6A1 |
| Brown _10 | hsa05020:  Prion disease | 0.0102 | 2.5645 | 11 | 4.87 | NDUFA9, NDUFA7, NDUFB7, NDUFS7,  NDUFA11, NDUFB11, TUBB, NDUFB2,  NDUFV3, COX6A1, PSMA7 |
| Brown _11 | hsa05415:  Diabetic cardiomyopathy | 0.0139 | 2.8218 | 9 | 3.98 | NDUFA9, NDUFA7, NDUFB7, NDUFS7,  NDUFA11, NDUFB11, NDUFB2, NDUFV3,  COX6A1 |
| Brown _12 | hsa04714:  Thermogenesis | 0.0284 | 2.469 | 9 | 3.98 | NDUFA9, NDUFA7, NDUFB7, NDUFS7,  NDUFA11, NDUFB11, NDUFB2, NDUFV3,  COX6A1 |
| Brown _13 | hsa05010:  Alzheimer disease | 0.03714 | 1.9890 | 12 | 5.31 | NDUFA9, NDUFA7, NDUFB7, NDUFS7,  NDUFA11, NDUFB11, GNAQ, TUBB,  NDUFB2, NDUFV3, COX6A1, PSMA7 |
| Brown _14 | hsa04146:  Peroxisome | 0.03888 | 3.8809 | 5 | 2.21 | GSTK1, HMGCL, PEX11B, PXMP4, MPV17 |
| Brown _15 | hsa00230:  Purine metabolism | 0.04952 | 2.9834 | 6 | 2.65 | ADSL, ATIC, IMPDH2, NUDT2, NUDT16, FHIT |
| Gray _1 | hsa04010:  MAPK signaling pathway | <0.0001 | 2.887 | 24 | 5.58 | NTRK2, JUN, CACNA2D1, CACNA1B,  HSPB1, BRAF, CACNA1C, HSPA2,  CACNA1F, FGF3, RASGRP4, IGF1R, CACNB2,  CACNG7, NR4A1, PPP5C, CACNB4, FGF18,  STMN1, FLNB, FGF23, FGF10, FGF21, MAP3K5 |
| Gray _2 | hsa04950:  Maturity onset diabetes of the young | <0.0001 | 9.781281 | 7 | 1.63 | NR5A2, PKLR, HNF4A, ONECUT1, PAX4,  FOXA2, NKX2-2 |
| Gray _3 | hsa04020:  Calcium signaling pathway | 0.0005 | 2.5848 | 18 | 4.19 | NTRK2, MYLK2, MCOLN3, CALML5,  CACNA1B, MST1R, CACNA1C,  CALML4, CACNA1F, FGF3, GRM5, HTR7,  LTB4R2, FGF18, HRC, FGF23, FGF10, FGF21 |
| Gray _4 | hsa05033:  Nicotine addiction | 0.0006 | 6.3578 | 7 | 1.62 | GABRA2, GABRB2, GABRP, GABRA1,  GABRA3, CACNA1B, SLC17A7 |
| Gray _5 | hsa04727:  GABAergic synapse | 0.0007 | 4.0821 | 10 | 2.33 | GABRA2, GABRB2, GABRP, GABRA1,  GABRA3, CACNA1B, CACNA1C, CACNA1F,  GPHN, GNG13 |
| Gray _6 | hsa04080:  Neuroactive ligand-receptor interaction | 0.0011 | 2.1778 | 22 | 5.12 | GABRA2, GABRB2, GABRP, GABRA1,  P2RY10, VIPR1, PTGIR, CHRNA2, GABRA3,  GPR50, SSTR2, LTB4R, PYY, GRM5, MC2R,  HTR7, GAL, P2RY4, LTB4R2, MC5R, CTSG,  HCRT |
| Gray _7 | hsa04662:  B cell receptor signaling pathway | 0.0020 | 3.8926 | 9 | 2.09 | CD79B, JUN, SYK, INPPL1, BTK, LILRA2,  FCGR2B, CD22, NFKBIB |
| Gray _8 | hsa05412:  Arrhythmogenic right ventricular cardiomyopathy | 0.0050 | 3.7746 | 8 | 1.86 | CACNG7, CACNB2, CACNB4, LAMA1,  CACNA2D1, LEF1, CACNA1C, CACNA1F |
| Gray _9 | hsa05224:  Breast cancer | 0.0071 | 2.7186 | 11 | 2.56 | JUN, FGF18, LEF1, WNT9B, BRAF, FGF23,  FGF3, HES5, FGF21, IGF1R, FGF10 |
| Gray _10 | hsa04723:  Retrograde endocannabinoid signaling | 0.0074 | 2.7002 | 11 | 2.59 | GABRA2, GABRB2, GABRP, GABRA1,  GRM5, GABRA3, CACNA1B, SLC17A7,  CACNA1C, CACNA1F, GNG13 |
| Gray _11 | hsa04744:  Phototransduction | 0.0076 | 6.2639 | 5 | 1.16 | CALML5, CNGA1, GUCA1A, CALML4, CNGB1 |
| Gray _12 | hsa04742:  Taste transduction | 0.0091 | 3.3796 | 8 | 1.86 | GABRA2, GABRA1, P2RY4, GABRA3,  HTR3A, TAS1R1, CACNA1C, GNG13 |
| Gray _13 | hsa04261:  Adrenergic signaling in cardiomyocytes | 0.0100 | 2.5950 | 11 | 2.56 | CACNG7, CACNB2, CACNB4, PPP2R2C,  CALML5, CREB3L1, CACNA2D1,  CALML4, CACNA1C, CACNA1F, RAPGEF4 |
| Gray _14 | hsa05218:  Melanoma | 0.0138 | 3.5321 | 7 | 1.63 | FGF18, BRAF, FGF23, FGF3, FGF21, IGF1R,  FGF10 |
| Gray _15 | hsa04921:  Oxytocin signaling pathway | 0.0256 | 2.3591 | 10 | 2.33 | CACNG7, MYLK2, CACNB2, JUN,  CACNB4, CALML5, CACNA2D1,  CALML4, CACNA1C, CACNA1F |
